# A unified computational view of DNA duplex, triplex, quadruplex and their donor–acceptor interactions

**DOI:** 10.1093/nar/gkab285

**Published:** 2021-04-24

**Authors:** Gyuri Park, Byunghwa Kang, Soyeon V Park, Donghwa Lee, Seung Soo Oh

**Affiliations:** Department of Materials Science and Engineering, Pohang University of Science and Technology (POSTECH), Pohang 37673, South Korea; Department of Materials Science and Engineering, Pohang University of Science and Technology (POSTECH), Pohang 37673, South Korea; Department of Materials Science and Engineering, Pohang University of Science and Technology (POSTECH), Pohang 37673, South Korea; Department of Materials Science and Engineering, Pohang University of Science and Technology (POSTECH), Pohang 37673, South Korea; Division of Advanced Materials Science, Pohang University of Science and Technology (POSTECH), Pohang 37673, South Korea; Institute for Convergence Research and Education in Advanced Technology (I-CREATE), Yonsei University, Incheon 21983, South Korea; Department of Materials Science and Engineering, Pohang University of Science and Technology (POSTECH), Pohang 37673, South Korea; Institute for Convergence Research and Education in Advanced Technology (I-CREATE), Yonsei University, Incheon 21983, South Korea; School of Interdisciplinary Bioscience and Bioengineering, Pohang University of Science and Technology (POSTECH), Pohang 37673, South Korea

## Abstract

DNA can assume various structures as a result of interactions at atomic and molecular levels (e.g., hydrogen bonds, π–π stacking interactions, and electrostatic potentials), so understanding of the consequences of these interactions could guide development of ways to produce elaborate programmable DNA for applications in bio- and nanotechnology. We conducted advanced *ab initio* calculations to investigate nucleobase model structures by componentizing their donor-acceptor interactions. By unifying computational conditions, we compared the independent interactions of DNA duplexes, triplexes, and quadruplexes, which led us to evaluate a stability trend among Watson–Crick and Hoogsteen base pairing, stacking, and even ion binding. For a realistic solution-like environment, the influence of water molecules was carefully considered, and the potassium-ion preference of G-quadruplex was first analyzed at an *ab initio* level by considering both base-base and ion-water interactions. We devised new structure factors including hydrogen bond length, glycosidic vector angle, and twist angle, which were highly effective for comparison between computationally-predicted and experimentally-determined structures; we clarified the function of phosphate backbone during nucleobase ordering. The simulated tendency of net interaction energies agreed well with that of real world, and this agreement validates the potential of *ab initio* study to guide programming of complicated DNA constructs.

## INTRODUCTION

Deoxyribonucleic acid (DNA) is a unique material as composed of nitrogenous bases (adenine (A), thymine (T), guanine (G) or cytosine (C)), sugar rings, and phosphate groups. DNA is programmable, so it can be rationally designed into molecular structures ranging from simple Watson–Crick base-pairing primers to DNA origami-based complex 3D constructs (1,2). Even spatial and temporal control of DNA nanostructures is achievable; sophisticated DNA molecular machines can perform a series of nanomechanical motions in a controllable manner, so this ability provides unprecedented applications in bio- and nanotechnology (3–6). Such active use of DNA requires fundamental understanding of DNA folding and its stabilization. In particular, DNA hybridization programming extensively exploits understanding of various donor-acceptor interactions of DNA, including hydrogen bonds, π–π stacking interactions, and electrostatic potentials (7).

Hydrogen bonds and π–π stacking are among the most important intra- and inter-molecular interactions in DNA (8–11). In general, nucleobases create specific hydrogen bonds between a purine (A or G) and a pyrimidine (C and T), which yield planar Watson–Crick base pairs (A–T; G–C). Between adjacent base pairs, π–π stacking interactions result in sequential stacking of base pairs. Therefore, hydrogen bonds and π–π stacking drive the formation of a duplex as a basic structure of DNA. However, the nucleobases can be influenced by different interactions due to pH and metal ions, so triplexes and quadruplexes are sometimes produced (12,13). For instance, C is protonated at slightly acidic pH to become a hydrogen-bond acceptor C^+^, which binds to the guanine of a G–C pair as a bond donor to yield a C^+^•G–C triad in a DNA triplex (14). The C^+^ can be also paired with a non-protonated form of C, and the resulting C–C^+^ pairs involve in formation of C-quadruplex, i.e., i-motif (15). Moreover, guanines can interact strongly with metal ions, and monovalent cations can influence a guanine-rich DNA strand to form a G-quadruplex (16,17). DNA should build thermodynamically-favored structures, which is a basic principle behind the spatiotemporal control of DNA nanoconstructs by modifying their environmental conditions.

Rational creation of advanced DNA structures requires in-depth understanding of DNA interactions under a variety of conditions; *ab initio* calculations can provide valuable insights in the behavior of proposed designs (18). This method provides reasonable values of dipole moments, charge distributions, and vibrational frequencies, so it can be useful to describe non-covalent molecular interactions (19,20). Importantly, all donor-acceptor interactions can be easily itemized, so that their independent functions in structure stabilization can be readily analyzed, and the relevant net energy is computed precisely. Therefore, the *ab initio* simulation has been exploited to interpret nucleic acid interactions. However, most previous studies have considered only interactions in gas phase (21–24), whereas actual DNA structures are in aqueous solutions. Some researchers have conducted phase-dependent simulation (25–27), but the calculation works used different computational conditions, making itemized DNA interactions not comparable to each other. Furthermore, the difference between computational and experimental results could not be well interpreted due to lack of effective analytical factors (26,28); in some calculations, the Watson–Crick base pair was less stable than a mismatch (MM) pair, i.e., a non-Watson–Crick base pair (26), and stacking of bases did not yield planarity (28). Therefore, there is a strong need for more realistic calculations of itemized DNA interactions under the unified conditions and analyses, making the computational results comparable with experimental ones.

**Scheme 1.**
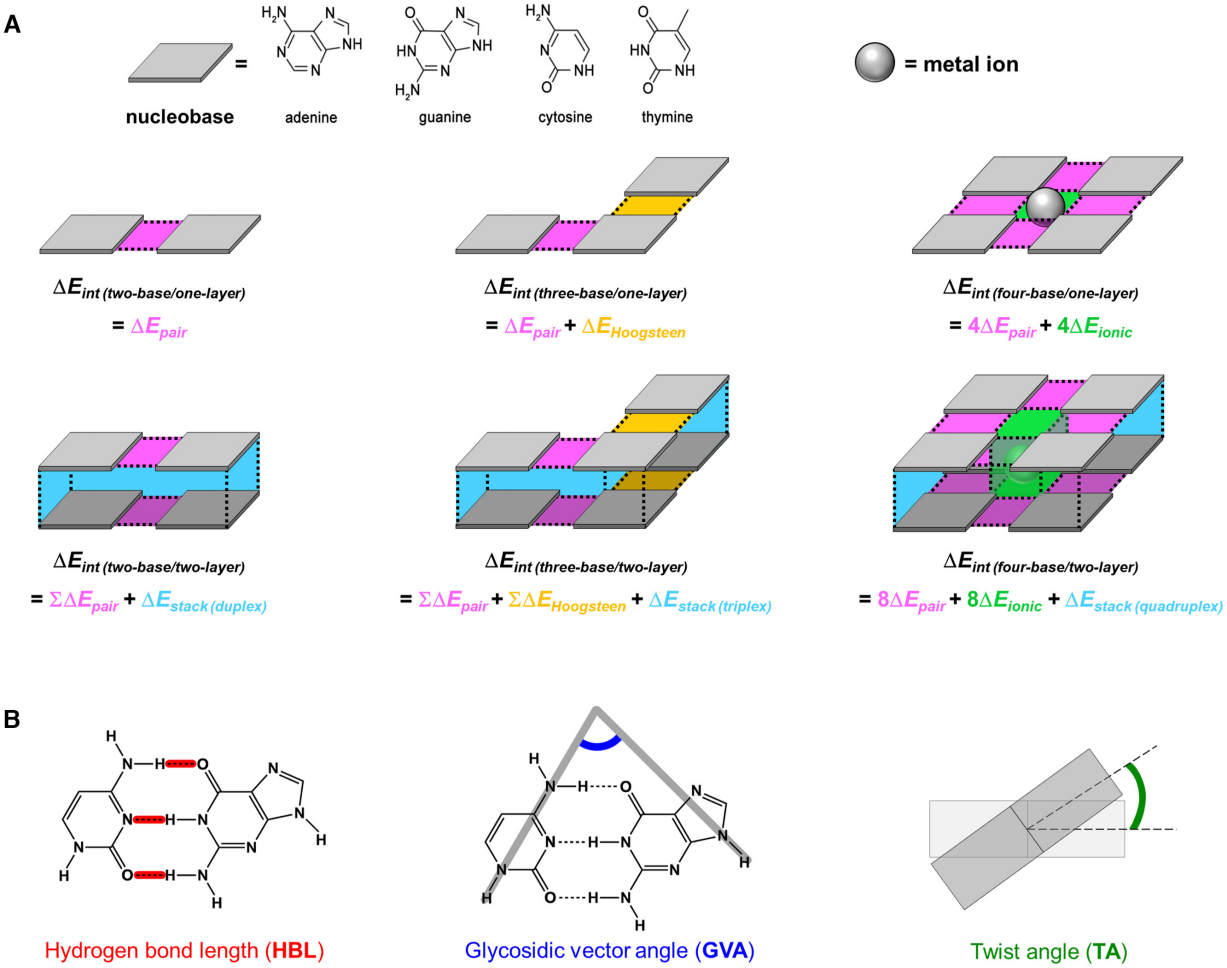
(**A**) Simulated nucleobase model structures and their itemized interactions. Planar base pair, triad, and tetrad and their double-layered structures are constructed for *ab initio* simulation. Gray tiles: nucleobases; spheres: monovalent metal ions. One-layer structures can have three different interactions: Watson–Crick or mismatch (MM) base-pairing (magenta), Hoogsteen base-pairing (yellow), and ionic bonding (green), which have interaction energies Δ*E_pair_*, Δ*E_Hoogsteen_*, and Δ*E_ionic_*, respectively. We note that one metal cation forms an ionic bond per nucleobase in the base tetrad. Two-layer structures additionally include a stacking interaction (cyan) which has energy Δ*E_stack_*. Overall interaction energy of a simulated structure is described as Δ*E_int_*. (**B**) Structural factors for conformational analysis. Hydrogen bond length (HBL, red) is defined as the length of hydrogen bonds of paired nucleobases. Glycosidic vector angle (GVA, blue) is an angle between two glycosidic vectors of nucleobases. Twist angle (TA, olive) is a rotation angle of one layer with respect to another. GVA and TA are obtained by vector dot-product calculations.

In this work, we conducted advanced *ab initio* calculations to simulate DNA duplexes, triplexes, and quadruplexes by using identical computation conditions and rationally itemized donor-acceptor interactions (Scheme 1a). To provide the fundamental information for designing and manipulating complicated DNA structures along with predictions of their potential stability and transient motions, we systematically analyzed stability orders of the itemized interactions and relevant structural changes. Specifically, we used the water continuum model to mimic the real environment in nature, and nucleobase interactions were specifically focused on; all related 3D structures were intensively investigated with important parameters such as numbers of bases and layers, and environmental effects (e.g., protonation, water solvation, and metal–ion interactions). Moreover, key interactions such as Watson–Crick and Hoogsteen base pairs, metal ionic bonds, and π–π stacking were independently componentized to facilitate identification of correlations among interactions and stabilizing structures. To fully understand G-quadruplex behaviors, we considered both base-base and ion-water interactions; to our best knowledge, we first provided the clue to the preference of potassium ion (K^+^) in G-quadruplex formation at an *ab initio* level. We also devised effective analytical factors, including hydrogen bond length (HBL), glycosidic vector angle (GVA), and twist angle (TA) (Scheme 1b). Using these structural factors, we thoroughly analyzed the relaxed structures by *ab initio* calculations and experimentally-determined structures by X-ray crystallography and nuclear magnetic resonance (NMR) spectroscopy, and those analyses were highly useful to identify how phosphate backbones influence nucleobase interactions. Owing to the unified computational view, various DNA constructs could be compared to each other to provide useful information, such as a stability trend among Watson–Crick base pairing, Hoogsteen base pairing, stacking, and metal-ion binding.

## MATERIALS AND METHODS

For our *ab initio* calculations, we used the Gaussian 09 (G09) program. To effectively investigate nucleobase interactions, DNA backbones including sugar rings and phosphate groups were replaced with hydrogen atoms. Based on the ideal structure of B-DNA, an initial model of two-layer structure was constructed with a parallel arrangement of base-pair layers, maintaining 3.4-Å stacking distance and 36° angle under its center of mass. Previous calculation and experimental determination reports have validated the feasibility of this model (24,29). The double-layer model was guided to initially have a fixed dihedral angle (Opt = ModRed), minimizing possible non-planarity caused by the absence of a backbone, and its optimization process was then followed. Every structure optimization and relaxation was conducted under M05–2X/6–31G(d,p)//M05–2X/6–31G(d,p) condition, and the basis set superposition error (BSSE) calculations were further corrected by the counterpoise (CP) method (30). The water solvent effect was realized by using a conductor-like screening model (COSMO) (31).

Shapes of DNA structures were determined by considering the numbers of bases and layers. The number of bases affects the hydrogen bonding among the nucleobases, whereas the number of layers represents π–π stacking interactions among the base-pair layers. Metal ions were added to construct G-tetrad or G-quadruplex structures in which the cations interact electrochemically with guanines.

All interactions are expressible in simple formulae. The binding energy Δ*E_bind_* is determined as the difference between the energy *E_complex_* of a complex system and the energy *E_sub_* of an isolated subsystem:

$$\begin{equation*}\Delta {\rm{}}{E_{bind}} = {E_{complex}} - {\rm{\Sigma }}{E_{sub}}\end{equation*}$$

The Δ*E_bind_* can be rewritten for a case of typical AB dimer, which is formed by binding monomer A and B, and Δ*E_bind_* can be defined:

$$\begin{eqnarray*} \Delta {E_{bind}}\left( {AB} \right) &=& E_{AB}^{AB}\left( {AB} \right) - E_A^A\left( A \right) - E_B^B\left( B \right) \nonumber \\ &=& E\left( {AB} \right) - E\left( A \right) - E\left( B \right) \end{eqnarray*}$$

In this formula, superscripts, subscripts, and symbols in parenthesis denote basis, geometry, and chemical system, respectively.

This binding energy formula can be used to derive the hydrogen bonding energy Δ*E_pair_* of Watson–Crick or MM base pairs:

$$\begin{equation*}\Delta {\rm{}}{E_{pair}} = {E_{complex}} - {\rm{\Sigma }}{E_{base}}\end{equation*}$$

*E_sub_* can be specifically defined; for instance, within a triad, *E_sub_* can be divided into the interaction energy Δ*E_pair_* of a base pair and the energy of all bases Σ*E_base_*, so the Hoogsteen base-pairing energy Δ*E_Hoogsteen_* can be obtained as

$$\begin{equation*}{\rm{\Delta }}{E_{Hoogsteen}} = {E_{complex}} - \left( {{\rm{\Delta }}{E_{pair}} + {\rm{\Sigma }}{E_{base}}} \right)\end{equation*}$$

Similarly, the interaction energy of G-tetrad (Δ*E_G-tetrad_*) is given by

$$\begin{eqnarray*}{\rm{\Delta }}{E_{G - tetrad}} &=& {E_{complex}} - \left( {4{\rm{\Delta }}{E_{pair}} + 4{E_{base}} + {E_{ion}}} \right) \nonumber \\ &=& 4{\rm{\Delta }}{E_{ionic}}\end{eqnarray*}$$

Δ*E_G-tetrad_* is four times the interaction energy Δ*E_ionic_* between a metal ion and a guanine because the monovalent cation forms one ionic bond per guanine.

The stacking interaction energy of a duplex is

$$\begin{equation*}{\rm{\Delta }}{E_{stack}} = {E_{complex}} - \left( {{\rm{\Sigma \Delta }}{E_{pair}} + {\rm{\Sigma }}{E_{base}}} \right)\end{equation*}$$

We can describe those of triplex and quadruplex in a similar way.

To obtain the CP-corrected Δ*E_bind_* ($\Delta E_{bind}^{CP}( {AB} )$), we additionally performed BSSE calculations (30). The basis of isolated monomer A and B is overlapped when the monomer A and B bind to each other to form a dimer AB. The AB can be further stabilized because of the basis overlap. To correct the additional binding interactions by BSSE, an extra basis overlap to each monomer should be removed, and the BSSE for monomer A (${E_{BSSE}}( A )$) and B (${E_{BSSE}}( B )$) can be described as:

\begin{equation*}\nonumber E_{BSSE}(A) = E_{AB}^{AB}( {\rm{A}} )-E_{AB}^{A}( {\rm{A}} )\end{equation*}

\begin{equation*}\nonumber E_{BSSE}(B) = E_{AB}^{AB}( {\rm{B}} )-E_{AB}^{B}( {\rm{B}} )\end{equation*}

$E_{AB}^{AB}( {\rm{A}} )\;$ and $E_{AB}^{AB}( B )$ are obtained from an optimized dimer geometry by assigning a ghost fragment to each monomer; $E_{AB}^A( {\rm{A}} )$ and $E_{AB}^B( B )$ are the energies of individual bases from the optimized complex geometry.

Then, the $\Delta E_{bind}^{CP}( {AB} )$ can be obtained by subtracting *E_BSSE_*(*A*) and *E_BSSE_*(*B*) from Δ*E_bind_*:

\begin{equation*}\nonumber \Delta E_{bind}^{CP}( {AB} ) = \Delta {E_{bind}}( {AB} ) - {E_{BSSE}}( A ) - {E_{BSSE}}( B )\end{equation*}

By this formula, we corrected the Δ*E_bind_* and acquired the $\Delta E_{bind}^{CP}( {AB} )$ values for all structures in our *ab initio* calculation study.

Finally, the overall interaction energy Δ*E_int_* of intermolecular interactions can be shown as

$$\begin{equation*}{\rm{\Delta }}{E_{int}} = {\rm{\Sigma \Delta }}{E_{pair}} + {\rm{\Sigma \Delta }}{E_{Hoogsteen}} + {\rm{\Sigma \Delta }}{E_{ionic}} + {\rm{\Delta }}{E_{stack}}\end{equation*}$$

In this formula, each interaction energy term, which is corrected by the CP method, can be zero depending on the type of structure; for example, in a two-base/two-layer structure, only ΣΔ*E_pair_* and Δ*E_stack_* are non-zero.

Without phosphate backbones, DNA structures in our calculations could be structurally distorted to find the most stable conformations. To identify the structural changes by the presence of the phosphate backbones, we compared the computationally-predicted DNA structures and the experimentally-determined structures. Among a number of DNA structures that have been determined by X-ray crystallography and solution NMR spectroscopy, we only considered the typical DNA helical structures; the DNA-containing complex structures (e.g., peptide nucleic acid–DNA, RNA–DNA and protein–DNA) and the DNA structures that possess nucleobase analogs were excluded. For G-quadruplexes, we analyzed both parallel and anti-parallel ones originated from *Oxytricha nova*, *Tetrahymena* and human telomeres. The PDB IDs of chosen DNA structures are 1ZF7, 1ZFB, 1ZFC, 1ZFH, 1ZFM (32), 1BWT (33), 1CS2 (34), 1D68 (35) for duplexes, 149D (36), 1BWG (37), 1D3X (38) for triplexes, and 1JB7 (39), 2AQY (40), 143D (41), 186D (42), 201D (43), 230D (44), 352D (45), 1JPQ (46), 1JRN (46), 1K8P (47), 1KF1 (47), 2GKU (48), 2JPZ (49) for G-quadruplexes.

## RESULTS AND DISCUSSION

### Overall *ab initio* calculation conditions and analytical factors

Using G09, we conducted every optimization and relaxation step under the M05–2X/6–31G(d,p)//M05–2X/6–31G(d,p) condition. The M05–2X method (50) is a specially-designed tool that describes non-covalent interactions of various biomolecular structures, making it possible to obtain calculation results of hydrogen bonding and base stacking interactions. Previously, this method has proven its suitability for nucleic acid calculations (51–53); specifically, hydrogen bonding and stacking patterns of DNA by M05–2X were comparable with those by second-order Møller–Plesset perturbation theory (MP2) (53,54), a relatively expensive method. The 6–31G(d,p) basis set (55,56) belongs to the double-zeta polarization group that represents reliable intra- and inter-molecular geometries (57), so DNA nucleobase structures can be described sufficiently well. The solvent effect of water phase was obtained using COSMO, which is useful to describe the Gibbs free energy, cavitation, internal energy, and entropy effect of the solvent (31). Computational results obtained using the COSMO solvation model agree well with experimental data when small molecules, such as nucleobases, are simulated (58,59). By applying the COSMO, we provided the appropriate solvation environment to DNA nucleobases during *ab initio* calculations. Our computational conditions yielded the calculation results remarkably close to those of triple-zeta basis set 6–311G(d,p) (Supplementary Figure S1a, b), and our COSMO model resulted in the interaction energy order that matches the general trend of nature better than that of integral equation formalism polarizable continuum model (IEFPCM) (Supplementary Figure S1c).

After the simulation, we systematically analyzed the relaxed structures by comparing structurally-important factors including HBL, GVA and TA (Scheme 1b). HBL is the length of hydrogen bonds between two adjacent nucleobases; it is involved directly in a pairing-interaction energy. As GVA is an angle between two glycosidic vectors of nucleobases, it is strongly correlated with structural integrity. If GVA deviates from the ideal value (72° for DNA duplexes), the DNA structure that contains the paired bases is assumed to be mechanically distorted. TA is a rotation angle of one layer with respect to another layer, and when twists occur within a helical DNA, torsional stress is expected. Using these structural factors, we systematically compared the relaxed DNA structures by *ab initio* calculations and the experimentally-determined ones by X-ray crystallography and NMR spectroscopy.

### Two-base/one-layer structures: Watson–Crick and MM base pairs

A duplex is the most basic structure among 3D DNA constructs, and base pairing is formed by interactions between nucleobases. We calculated the interaction energy Δ*E_pair_* of all possible two-base structures including the complementary Watson–Crick and non-complementary MM base pairs both in vacuum and in water (Supplementary Figures S2 and S3, Tables S1 and S2).

The Δ*E_pair_* order in the water phase suggests that the number of hydrogen bonds between nucleobases influences the stability of base pairs (Table 1, left). The G–C pair, which has the lowest Δ*E_pair_*, is highly stable due to its three interbase hydrogen bonds. In contrast, the C–C pair has a single hydrogen bond, so it is the least stable. Thus, we reconfirmed that the number of hydrogen bonds is a dominant factor to determine the degree of stability.

**Table 1.** Calculated interaction energy (kcal/mol) of base pair Δ*E_pair_*, Hoogsteen pair (in triad) Δ*E_Hoogsteen_*, and ionic bond (in tetrad) Δ*E_ionic_* in water phase. Hydrogen bonds are indicated by hyphens (-) for Watson–Crick or MM base pairs, and by bullets (•) for Hoogsteen base pairs. Ionic bonds between guanines and metal ions are represented by ellipsis (⋅⋅⋅). Δ*E* values are arranged in order of decreasing stability. However, Δ*E_Hoogsteen_* values are grouped whether the Hoogsteen base pair includes a flipped third base or not. The values in parenthesis indicate energy per hydrogen bond.

Pair	Δ*E_pair_*	Triad	Δ*E_Hoogsteen_*	Tetrad	Δ*E_ionic_*
**G–C**	–11.45 (–3.82)	**C^+^•G**	–11.04 (–5.52)	**G4⋅⋅⋅Li^+^**	–31.28 (–7.82)
**A–T**	–6.80 (–3.40)	**G•G**	–7.81 (–3.91)	**G4⋅⋅⋅Na^+^**	–27.68 (–6.92)
**G–T**	–6.77 (–3.38)	**T•A**	–6.50 (–3.25)	**G4⋅⋅⋅K^+^**	–20.26 (–5.07)
**G–G**	–6.75 (–3.37)	**A•A**	–1.69 (–0.84)		
**C–T**	–6.58 (–3.29)	**rC^+^•G**	–11.08 (–5.54)		
**T–T**	–6.43 (–3.21)	**rG•G**	–7.64 (–3.82)		
**A–G**	–6.41 (–3.20)	**rT•A**	–4.90 (–2.45)		
**A–C**	–3.39 (–1.70)	**rA•A**	–3.83 (–1.92)		
**A–A**	–3.25 (–1.62)				
**C–C**	–3.16				

**Figure 1.**
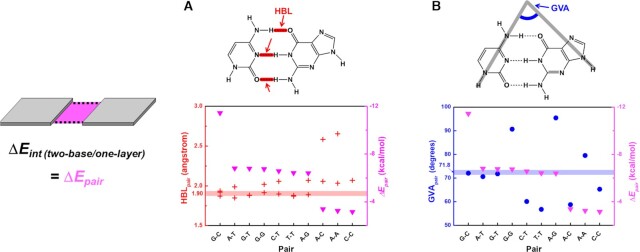
(**A**) HBL (red cross) and (**B**) GVA (blue circle) of Watson–Crick and MM base pairs, along with Δ*E_pair_* (magenta inverted triangle). Solid lines indicate the mean HBL and GVA of Watson–Crick base pairs obtained from X-ray crystallography and NMR measurements (PDB ID: 1ZF7, 1ZFB, 1ZFC, 1ZFH, 1ZFM, 1BWT, 1CS2 and 1D68).

The simulated HBL and GVA of Watson–Crick and wobble pairs are quite close to the experimentally-obtained values (PDB ID: 1ZF7, 1ZFB, 1ZFC, 1ZFH, 1ZFM, 1BWT, 1CS2, and 1D68) (Figure 1 and Table S2). Analysis of experimentally-determined structures indicates that the Watson–Crick base pairs have 1.90 Å mean HBL and ∼72° GVA (71.8°) (Supplementary Tables S3 and S4), and only the three most stable pairs (i.e., G–C, A–T and G–T) satisfy both ranges; importantly, their stability order is G–C > A–T > G–T in water (Table 1, left), which is consistent with the actual character of DNA (60). We note that when we conducted coupled cluster single-double excitation (CCSD) calculations (61–63), one of the most accurate and expensive calculations, the stability order was identical, justifying the reliability of our M05–2X method for optimizations (Supplementary Figure S1b). In our calculations, the G-T wobble pair has similar mean HBL and GVA values to those of the perfectly-matched A-T pair (Supplementary Table S2). Nevertheless, this non-Watson–Crick pair is less stable than the A–T pair due to the inconsistent pattern of hydrogen donor and acceptor. In Watson–Crick base pairs, the keto O4 of thymine and amino N2 of guanine participate in formation of intrabase hydrogen bonds for A–T and G–C pairs, respectively. However, in the G–T pair, they are not involved in those bonding patterns, so this pair has low stability (64).

Compared to the top three stable pairs, other MM pairs (e.g., G–G, C–T, T–T, A–G, A–C, A–A, and C–C) have GVAs that deviate significantly from the ideal value of ∼72° (Figure 1B). Especially, purine-purine pairs have GVA >> 72°, so steric hindrance between the large purines is anticipated. The effect of the steric hindrance in MM pairs also can be observed by long mean HBL values, which are 1.98 Å for A–G, 1.97 Å for G–G, and 2.34 Å for A–A (Supplementary Table S2). However, A–G and G–G have GVA close to 90°, so these purine-purine pairs have potential to seize each other in a square to form a tetrad (65). The T–T MM pair has a similar mean HBL to that of the G–T wobble pair (Supplementary Table S2), but the T–T MM pair is less stable than the G–T wobble pair. Moreover, as the T–T MM pair has the GVA << 72°, so the duplex would be further destabilized within a DNA double helix due to structural distortion. Thus, we conclude that for a duplex to be stable in nature, both HBL and GVA values should be in the appropriate range.

A material's phase has a critical influence on the Δ*E_pair_* order. For example, in vacuum, the Watson–Crick A–T pair is less stable than some MM pairs (Supplementary Table S1) as it has a deviated GVA = 67.4° (Supplementary Table S2). However, in an aqueous environment, the A–T pair has appropriate GVA = 70.6°, so the stability order of base pairs becomes the actual G–C > A–T > G–T. This result confirms that the vacuum condition does not represent the actual tendency of natural DNA, and that nucleobase simulation should be performed in an aqueous environment.

The Δ*E_pair_* order and the GVAs of base pairs would be highly useful for a duplex design. For example, single nucleotide polymorphism (SNP) detection requires to design DNA probes to selectively bind the SNP site (66), which would be benefited by our Δ*E_pair_* order analysis. Additionally, the GVAs involve in the width and depth of grooves in DNA, related to protein–DNA binding interactions, and by incorporating MM pairs, groove manipulation would be achievable for gene regulation, including transcriptional control (67).

### Two-base/two-layer structures: Watson–Crick base pair and stacking interactions

π–π stacking interaction has a strong influence on formation of double helix structures, i.e., a stack of base-pair layers. We next considered the stacking interaction for construction of DNA duplex, and two-layer structures of perfect-matched Watson–Crick base pairs were simulated (Supplementary Figures S4 and S5).

In vacuum, Δ*E_stack_* is affected by the degree of layer (non)planarity (Supplementary Figure S4). Even though all structures are initially constructed with parallel arrangements of two planar base pairs, the base-pair layers become nonplanar after structural optimization (Supplementary Figure S4, Table S2). The calculations in the gas phase cannot include the neutralization effect of the water continuum; additional hydrogen bonds can form between the layers, so the initial parallel structures are forced to be severely distorted. Compared to the mean GVA of single layers, those of the double layers are also decreased as a result of this structure distortion (Supplementary Table S2).

When the phase condition is changed from gas to water, the base-pair layers become planar (Supplementary Figure S5), and all the GVA are close to 71.8°, as expected from conventional Watson–Crick pairings (Supplementary Table S2). The introduction of the water continuum model successfully excludes the undesired dipole-dipole interactions in the gas by using Van der Waals interactions between solvent and solute. Formation of unwanted hydrogen bonds is interrupted between the base-pair layers, so the planar surfaces can be maintained even after optimization. In water, all calculated structures have relatively similar Δ*E_stack_* (Table 2, left); this result may occur because when two layers of purine-pyrimidine pairs are stacked on each other, the degree of layer overlapping would be quite similar under the influence of the π–π stacking.

**Table 2.** Calculated stacking interaction energy Δ*E_stack_* (kcal/mol) of duplex, triplex, quadruplex in water. Layers are divided by slash (/). Δ*E_stack_* is arranged in order of decreasing stability, but Δ*E_stack_* values of triplexes are grouped according to whether stacked third bases are parallel or antiparallel. Values in parenthesis: energy per ionic bond.

Duplex		Triplex		Quadruplex	
**G–C/C–G**	–5.46*	**A·A–T/A·A–T**	–11.87*	**G4/G4**	–13.16
**A–T/A–T**	–5.22	**C+·G–C/T·A–T**	–8.56*		
**G–C/A–T**	–5.21	**G·G–C/G·G–C**	–7.78*	**ΣΔ*E_ionic_***	
**A–T/T–A**	–5.05*	**T·A–T/T·A–T**	–7.37	**G4⋅⋅⋅Na^+^⋅⋅⋅G4**	–39.1 (–4.9)
**G–C/G–C**	–4.88	**C+·G–C/C+·G–C**	–3.77	**G4⋅⋅⋅K^+^⋅⋅⋅G4**	–37.0 (–4.6)
**G–C/T–A**	–4.04*	**rT·A–T/rT·A–T**	–13.07*	**G4⋅⋅⋅Li^+^⋅⋅⋅G4**	–32.8 (–4.1)
		**rA·A–T/rA·A–T**	–12.19		
		**rG·G–C/rG·G–C**	–7.07		
		**rC+·G–C/rC+·G–C**	–4.10*		

*If model structures further include sugar rings and phosphate backbones, additional torsional stress, *i.e.*, stability loss, is anticipated based on TA comparison between experimental and computational results.

Consequently, this reduces the influence of Δ*E_stack_* on the order of Δ*E_int_* in water; the influence of Δ*E_pair_* is significant when perfect-matched base pairs are layered (Supplementary Table S1). The Δ*E_int_* order mainly depends on the percent of G–C content in the entire structures, meaning that the number of hydrogen bonds dominantly contributes to determining the DNA stability, and that the influence of π–π interactions is relatively less important.

**Figure 2.**
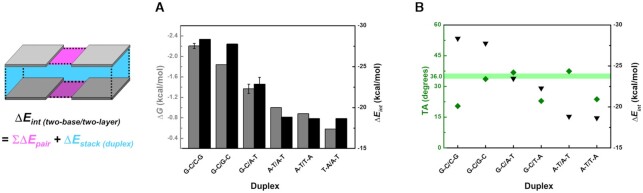
(**A**) Comparing thermodynamic Δ*G* (gray) (68) with calculated Δ*E_int_* (black). Thermodynamic Δ*G* results of duplexes show the trend, G–C/C–G = C–G/C–G and G–C/A–T = C–G/A–T = C–G/T–A = G–C/T–A. Compared to experimental nearest-neighbor models, our computational ones cannot have the directionality, so Δ*E_int_* of A–T/T–A and T–A/A–T are identical in simulation. (**B**) TA (olive diamonds) and Δ*E_int_* (black inverted triangles) of two-base/two-layer structures. The two-base/two-layer structures are ordered by stability of duplex, and Δ*E_int_* is the sum of 2Δ*E_pair_* (top and bottom layers) and Δ*E_stack (duplex)_*. Solid line: TA of duplex from crystallography and NMR data (PDB ID: 1ZF7, 1ZFB, 1ZFC, 1ZFH, 1ZFM, 1BWT, 1CS2 and 1D68).

The significance of our calculation results is that the stability order by Δ*E_int_* calculations shows an almost identical trend to those of nearest-neighbor thermodynamics results (68): G–C/C–G = C–G/G–C > G–C/G–C > G–C/A–T = C–G/T–A = C–G/A–T = G–C/T–A > A–T/A–T > A–T/T–A > T–A/A–T (Figure 2A). However, the experimental result shows a Δ*G* difference of A–T/T–A and T–A/A–T because the actual DNA structure has directionality as a result of the orientation of the 3′ and 5′ carbons along the DNA backbone, whereas this effect is not considered in our calculation model. Moreover, we did not consider several factors such as salts (cations and anions), pH, and strand-end effects, because these factors have much less influence on the stability of DNA than do the interactions between bases (8–11). Inclusion of these factors might enable correct prediction of the order of A–T/T–A and T–A/A–T.

The TA calculation results also support the anticipated Δ*E_int_* order (Figure 2b, Supplementary Table S5). Unlike actual DNA duplex structures, base-pair layers in our computational model do not have phosphate backbones, so the layers can rotate freely to find the most stable conformations. Compared to the ideal double helix that has TA = 36.0°, underrotated or overrotated helical structures must be less stable, so the TA value can be an indicator of potential torsional stress, showing the influence of the phosphate backbones to base-base and stacking interactions. The real Δ*E_int_* values of G–C/T–A and A–T/T–A are supposed to be higher than their calculated Δ*E_int_* due to their underrotation; this does not affect the Δ*E_int_* order, but their energy differences with G–C/A–T and A–T/A–T increase respectively. The torsional stress effect on G–C/C–G is not clear yet, so further investigation is ongoing. However, these TA calculations would be still useful information for precisely designing complex DNA structures, such as DNA origamis. Given that torsional stress of phosphate backbones is readily considered, the sum of TAs for all layers ideally determines the turns of helix; by calculating half or full turns of helices, the front and back of 2D patterns could be well distinguished (69), and even the curvature (70) or twist (71) of 3D DNA structures would be finely tuned based on the relation between TAs and DNA turns. Furthermore, as the TA values reflect the latent torsion stress, the TA calculations could be valuable for the design of DNA structures performing nanomechanical motions (72,73).

### Three-base/one-layer structures: Watson–Crick and Hoogsteen base pairs

The DNA duplex can be extended to construct a triplex when third neighboring bases are introduced to provide additional electron donor-acceptor interactions. The additional interactions can be found at a Hoogsteen edge, termed a Hoogsteen base pair. To analyze the Hoogsteen interaction, we conducted simulation of planar three-base structures by positioning the third bases near the optimized Watson–Crick base pairings. In this study, we chose six natural (C^+^•G–C, T•A–T, rT•A–T, rA•A–T, G•G–C and rG•G–C, where r indicates a flipped base) (Table 1, see notation) (12,74,75) and two unnatural (rC^+^•G–C and A•A–T) triads (Supplementary Figures S6 and S7). C^+^•G–C and T•A–T triads have been widely used to design DNA triple-stranded helices, wherein the C^+^•G–C requires a slightly acidic pH environment to protonate the third cytosine base (14). When a third strand is purine-rich, it can bind to a double helix in an antiparallel manner to form reversed Hoogsteen pairs, and three different triads (rT•A–T, rA•A–T and rG•G–C) that contain flipped third bases are found in the antiparallel triple helix (76). G•G–C triads are not common, but they have been discovered in several high-resolution crystallography studies (77). For comparison with the natural three-base structures, two unnatural triads (rC^+^•G–C and A•A–T) were also chosen.

**Figure 3.**
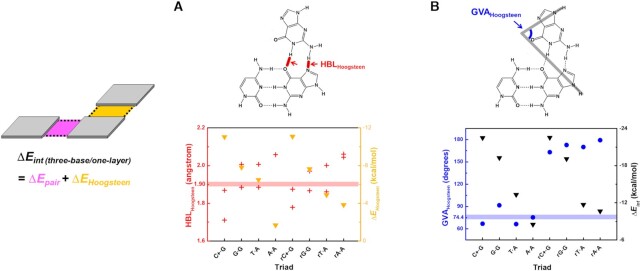
(**A**) HBL (red cross) and Δ*E_Hoogsteen_* (yellow inverted triangle) of Hoogsteen base pairs in simulated triads. Solid line indicates the NMR mean HBL of C^+^•G and T•A (PDB ID: 149D, 1BWG and 1D3X). (**B**) GVA of Hoogsteen base pairs (blue circle) and Δ*E_int_* of triads (black inverted triangle), which is the sum of Δ*E_pair_* and Δ*E_Hoogsteen_*. Solid line indicates the NMR mean GVA of C^+^•G and T•A.

Regardless of phase and base flipping, the stability order determined by Δ*E_Hoogsteen_* is (r)C^+^•G > (r)G•G > (r)T•A > (r)A•A (Table 1, middle). When we obtained the mean HBL of Hoogsteen base pairs from available solution NMR data (PDB ID: 149D, 1BWG and 1D3X), (r)C^+^•G and (r)A•A show somehow the difference between experimental determination and calculation. The calculated HBLs of (r)C^+^•G are shorter than the experimentally-determined ones (Figure 3A); when the (r)C^+^•G–C triad is connected to a phosphate backbone, the (r)C^+^•G is supposed to be under tensile stress, so the actual Δ*E_Hoogsteen_* is higher than the calculated one. The simulated (r)A•A shows longer HBLs, so compressive stress is expected in presence of the backbone, similarly decreasing the stability. On the contrary, the (r)G•G and (r)T•A show the relatively smaller difference between experimentally-observed and calculated HBLs, indicating that their Δ*E_Hoogsteen_* would be less relevant to the absence of the phosphate backbone. Interestingly, the calculated Δ*E_Hoogsteen_* of a triad is proportional to its mean HBL (Supplementary Table S2), whether the third base is flipped or not. Compared to the Hoogsteen pairs, Watson–Crick base pairs within the simulated triads have similar HBLs and GVAs to each other (Supplementary Tables S6 and S7, Figure S8), and they are quite close to the ideal values. This result suggests that the pre-existing Watson–Crick pairs are not significantly affected by the access of third bases during formation of Hoogsteen base pairs. Hoogsteen interactions do not much depend on Watson–Crick interactions, so Δ*E_Hoogsteen_* would be crucial to stabilize a triple helix.

Among the calculated triads that have unflipped third bases, C^+^•G can form the most stable Hoogsteen pair (Table 1), due to the molecular ion-molecular dipole interaction among nucleobases (78). In contrast, the A•A Hoogsteen base pair is energetically disfavored within the A•A–T triad. Interestingly, Δ*E_Hoogsteen_* of G•G–C is slightly lower than that of T•A–T even though the T•A–T is found much more frequently than G•G–C in nature. Although the HBLs of their Hoogsteen pairs are similar to each other, the G•G–C has large GVA > 90° as a result of its purine-purine pairing (Figure 3B). This large GVA can cause a severe structural distortion and therefore cannot be accepted in an actual triple helix. However, our simulated model freely accepts this distortion due to exclusion of the phosphate backbone. According to this structural freedom, Δ*E_Hoogsteen_* of G•G–C is comparable with that of T•A–T only in the calculation; in reality, the G•G Hoogsteen pair would be less stable than the T•A Hoogsteen pair.

Surprisingly, the most stable triad is rC^+^•G–C, which is an unnatural triad that has not been reported in nature (12,74,75). The stability of rC^+^•G–C is expected to be caused by the flipped third base; as a result, the oxygen atom of rC^+^ has a chance to approach the hydrogen atom of C and thereby causes formation of an additional hydrogen bond (Supplementary Figure S9). However, the actual formation of DNA triple helix must consider the backbone directionality of the third strand. The triads that contain flipped third base show calculated GVA closed to 180° (Figure 3b), which indicates that when a third strand is located within the major groove of double helix, its positioning can be disturbed by steric hindrance or helical under/overwinding. In addition, the rC^+^•G–C may cause electrostatically repulsive interactions with neighboring layers within the triple helix. The effect of third base flipping is strongly relevant to stacking interactions, so it is further evaluated in the next section.

### Three-base/two-layer structures: Hoogsteen base pairs and stacking interactions

In nature, a base triad alone is not stable, but its stacking structure, i.e., a triple helix, can be retained. In that configuration, π–π stacking interactions between the triad layers greatly contribute to formation of a stable triple-stranded helical structure. For next three-base/two-layer modeling, we first arranged the well-known triads (C^+^•G–C and T•A–T) to form C^+^•G–C/C^+^•G–C, C^+^•G–C/T•A–T and T•A–T/T•A–T triplexes. Second, to investigate a stacking effect of purine-purine Hoogsteen base pairs, we stacked G•G–C and A•A–T onto the same triads to construct G•G–C/G•G–C and A•A–T/A•A–T double layer structures. Finally, we simulated homolayers composed of triads that contain flipped third bases (rC^+^•G–C/rC^+^•G–C, rT•A–T/rT•A–T, rG•G–C/rG•G–C and rA•A–T/rA•A–T) to observe the effects of antiparallel strands in a triplex (Supplementary Figures S10 and S11).

**Figure 4.**
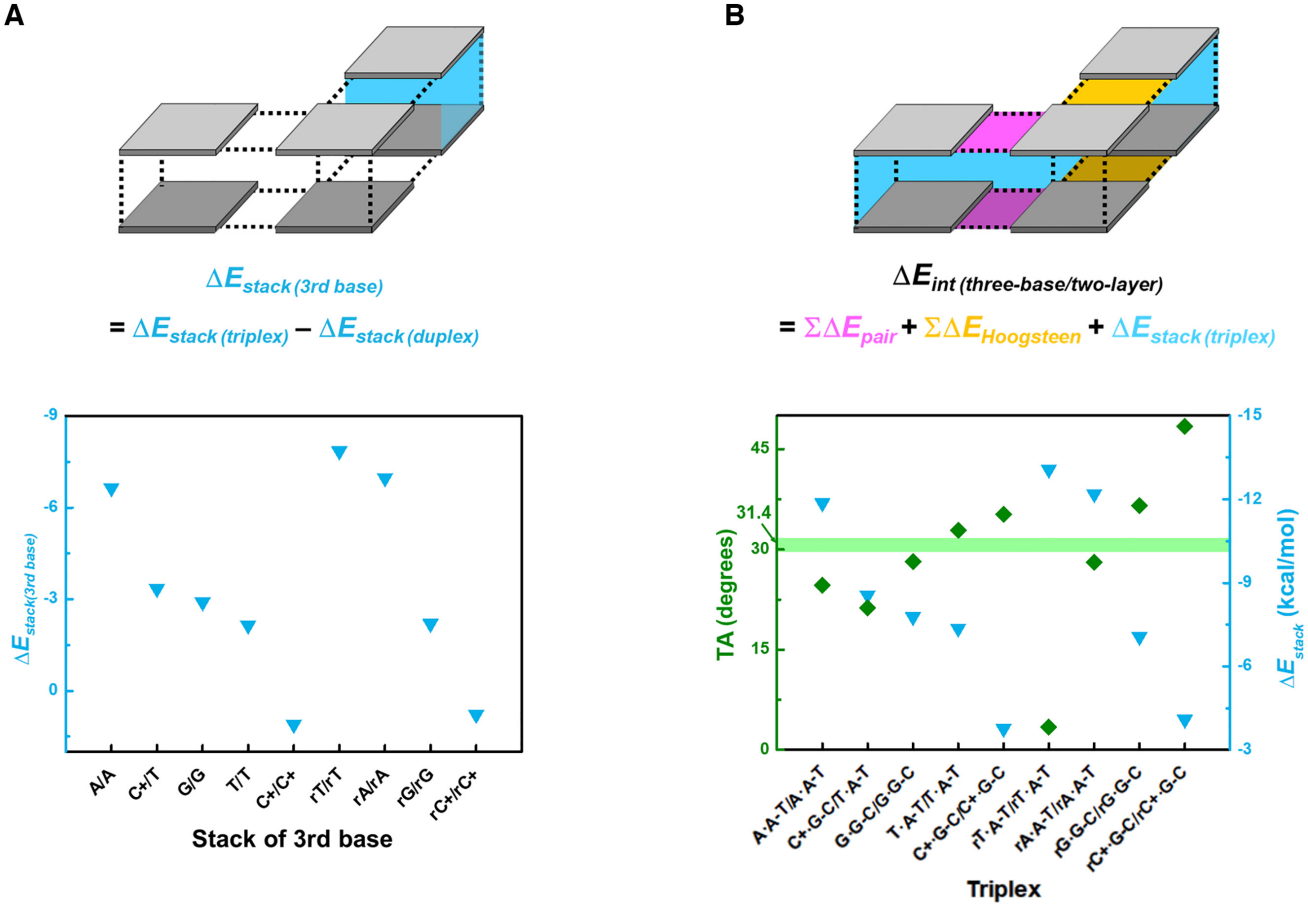
(**A**) Stacking interaction energy of third bases (Δ*E_stack (3rd base_*_)_), which is obtained by subtracting Δ*E_stack (duplex_*_)_ from Δ*E_stack (triplex_*_)_. (**B**) TA (olive diamonds) and Δ*E_stack (triplex_*_)_ (cyan inverted triangle) of triplexes. The mean TA of experimentally-determined C^+^•G–C/T•A–T and T•A–T/T•A–T is represented as solid line (PDB ID: 149D, 1BWG, and 1D3X).

According to the Δ*E_stack_* order in both phases, the C^+^•G–C/C^+^•G–C stacking is the least stable among the simulated triplexes (Table 2, middle; Supplementary Table S1). The stack instability is induced by strong charge-charge repulsion between C^+^ of each layer, and this repulsion distorts the triad layer into a nonplanar structure (Supplementary Figures S10 and S11). Therefore, when triple helices are rationally designed, successive C^+^•G–C layers should be avoided, and C^+^•G–C/T•A–T and T•A–T/T•A–T can be favored instead (79). C^+^•G–C/T•A–T triplexes have lower Δ*E_stack_* than T•A–T/T•A–T due to the different π–π stacking energy Δ*E_stack(__3rd base)_* between the third bases. Δ*E_stack(__3rd base)_* can be simply obtained by subtracting Δ*E_stack(duplex)_* from Δ*E_stack(triplex)_* (Figure 4A). The calculated Δ*E_stack(__3rd base)_* suggests that the C^+^/T stack of C^+^•G–C/T•A–T is more stable (–3.35 kcal/mol) than the T/T stack of T•A–T/T•A–T (–2.15 kcal/mol). These observations indicate that insertion of C^+^•G-C/T•A–T would stabilize the triple-stranded helix, in terms of stacking, better than insertion of T•A–T/T•A–T. However, the C^+^•G–C/T•A–T has smaller TA than the experimentally-determined value (Figure 4B, Supplementary Table S8), inferring that during triplex structure design, repetitive insertion of C^+^•G–C/T•A–T should be avoided because of undesired torsional stress.

Δ*E_stack(__3rd base)_* of the A/A stack in A•A–T/A•A–T (–6.65 kcal/mol) is the lowest among the triplexes that do not have flipped third bases. However, Δ*E_Hoogsteen_* of A•A Hoogsteen base pair (–1.69 kcal/mol) is significantly higher than the others (Table 1, middle), and the small TA = 24.6° of A•A–T/A•A–T indicates that this underrotated structure would be affected by torsional stress; this may be the reason that tandem repeats of A are not included in the third parallel strand of a natural triple helix, but can be only permitted by specifically-designed DNA structures in the laboratory (80).

Another triad stacking structure that contains purine-purine Hoogsteen pairs, G•G–C/G•G–C, has a value of Δ*E_stack(__3rd base)_* (–2.91 kcal/mol) that is higher than that of A•A–T/A•A–T, but is lower than that of T•A–T/T•A–T. Again, the calculated Δ*E_Hoogsteen_* value of G•G–C is comparable with that of naturally-occurring T•A–T, and the simulated TA of the G•G–C/G•G–C double layer has a negligible difference from the ideal TA value; it is therefore assumed to be unaffected by torsional stress. These observations suggest that G tandem repeats could be readily used to design a parallel triple-stranded helix if their steric hindrance is systematically compensated for GVA > 90° (Figure 3B).

Although rT•A–T/rT•A–T gives the most stable stacking interaction (Table 2, middle), such triad double layers are rare in naturally-occurring triple helices; the rarity may be caused by the preference for layer sliding as a phosphate backbone is absent in our computational model (81). During structure optimization, the rT•A–T triads slide preferentially to each other (Supplementary Figure S11) rather than to maintain the initial twist condition between layers (TA = 3.4°, Figure 4B). The highly decreased TA in *ab initio* calculations suggests that rT•A–T/rT•A–T double layers without the phosphodiester backbone may be stabilized at global minima. However, this layer sliding can allow the actual triple helix to underwind, making the helical structure destabilized due to torsional stress. Unlike the rT•A–T/rT•A–T, T•A–T/T•A–T shows no TA difference between the relaxed and experimentally-determined triplexes, indicating that T•A–T/T•A–T already has the preferred stacking structure only by base-base interactions. It would be the reason that when a poly-T strand binds to a DNA duplex, formation of parallel triple helix is more favored in nature than that of antiparallel one (82–84). Other double layers that include flipped third purine bases (e.g., rG•G–C/rG•G–C and rA•A–T/rA•A–T) display TA quite close to the ideal value of 31.4°. These observations may provide a clue to why formation of antiparallel triple helices requires a purine-rich third strand. Furthermore, using under- or overwinding information during triad stacking, optimal antiparallel triplexes can be readily designed by compensation of all torsional stress.

### Four-base/one-layer structures: G-tetrad and donor-acceptor bonds in metal ion binding

Nucleobases can form a quadruplex when other elements are involved in its formation. For instance, monovalent cations (M^+^) such as Na^+^ and K^+^ support nucleic acids to construct a G-quadruplex structure; briefly, addition of these ions affects interactions between guanine bases and thereby causes change in various factors (e.g., HBLs, GVAs and TAs). To investigate this ion-dependent formation of quadruplexes, we first focused on a guanine tetrad, which requires widely-accessible monovalent cations such as Li^+^, Na^+^, and K^+^ (Supplementary Figures S13 and S14). To clarify the effect of the metal ion, we simulated and compared G-tetrad structures both with and without metal ions (Supplementary Figure S12).

When monovalent metal cations are located in the guanine tetrads, ionic radii are crucial to determine whether the ion positions can be in-plane or out-of-plane with respect to the planar G-tetrad layers. Electrostatically, the G-tetrad tends to catch a monovalent cation in the center of itself due to partially negatively-charged oxygen atoms in the guanines (Supplementary Figure S15). Even when Li^+^ and Na^+^ are initially placed out of the preformed G-tetrads in *ab initio* calculations, the electrostatic charge-charge attraction draws the metal ions into an in-plane orientation, so the ions move into the center of the G-tetrad. In contrast, K^+^ is not in the center, but moves upwards along the central line perpendicular to the surface of the G-tetrad (Supplementary Figures S13 and S14); this observation is consistent with previous computational result (85). Compared to Li^+^ and Na^+^ that are readily accepted in the center of G-tetrad, the K^+^ has a longer ionic radius. Its relatively large ion size causes severe steric hindrance, so it remains above the G-tetrad.

**Figure 5.**
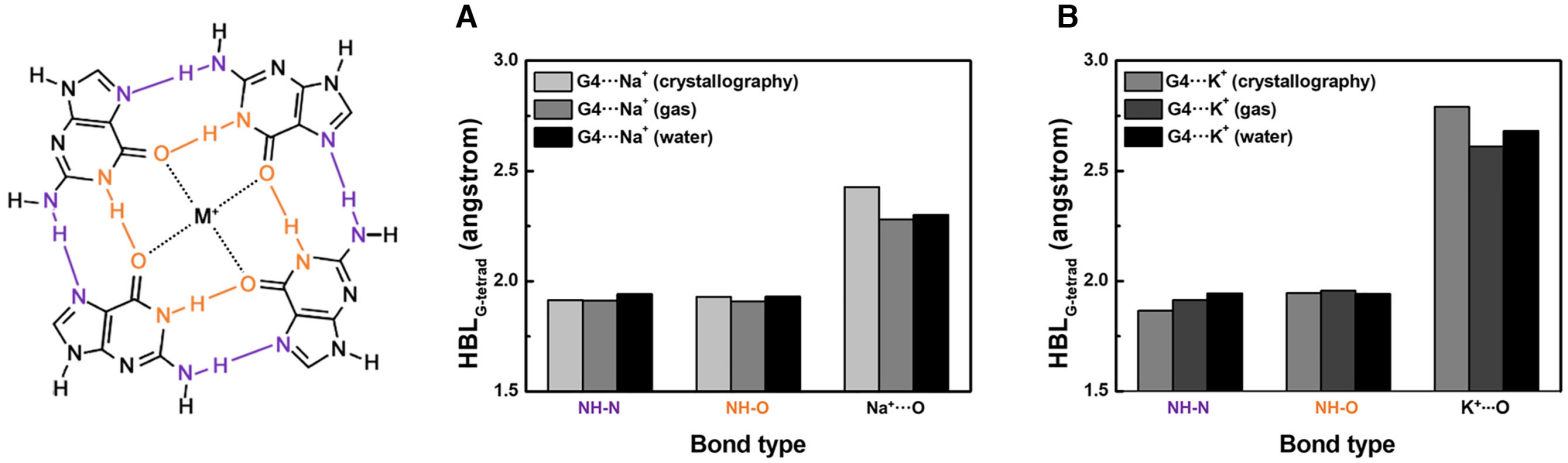
NH-N (purple), NH-O (orange), and M^+^⋅⋅⋅O (black) bonds of G-tetrad in crystallography and NMR data (light gray) and in gas- and water-phase calculations (dark gray and black, respectively). Mean bond length of G4⋅⋅⋅Na^+^ (**A**) and G4⋅⋅⋅K^+^ (**B**). The experimentally-determined bond length of Na^+^⋅⋅⋅O is obtained from in-plane G4⋅⋅⋅Na^+^ tetrads (PDB ID: 1JB7, 2AQY, 143D, 186D, 201D, 230D and 352D).

From our *ab initio* calculations of monovalent ion-containing G-tetrads (G4⋅⋅⋅M^+^) in both phases, the stability orders determined by Δ*E_ionic_* and Δ*E_int_* are G4⋅⋅⋅Li^+^ > G4⋅⋅⋅Na^+^ > G4⋅⋅⋅K^+^ (Table 1, right; Supplementary Table S1), which is the opposite of the natural behavior of G-quadruplexes: G4⋅⋅⋅K^+^ > G4⋅⋅⋅Na^+^ > G4⋅⋅⋅Li^+^ (86,87). Interestingly, this disagreement is not a result of our computational setup that excludes phosphodiester backbones which strongly affect the size of a G-quadruplex. In our calculations, G-tetrad can freely distort their size and conformation to captures target cation, so the G-tetrad structures in simulation may behave differently compared to the actual ones (39–46,48,49) (Figure 5, Supplementary Table S9). To confirm it, we analyzed the computed HBLs that are involved in the stability of a G-tetrad in the presence of metal ion. G4⋅⋅⋅M^+^ features two hydrogen bonds: NH-N and NH-O (Figure 5). The length of NH-N bond affects the size of the G-tetrad, whereas the NH–O bonds provide a space in which the metal ion is held. Mean computed HBLs of NH-N and NH-O are similar to the experimentally-determined in-plane G4⋅⋅⋅Na^+^ (1JB7, 2AQY, 143D, 186D, 201D, 230D and 352D) (Figure 5A). However, the simulated G4⋅⋅⋅K^+^ has longer NH-N mean HBL than crystallography and NMR spectroscopy measurements (1JPQ, 1JRN, 1K8P, 1KF1, 2GKU and 2JPZ), although NH-O bond lengths differ by < 2% from the experimentally-obtained counterparts (Figure 5B). The increased mean length of NH-N bonds in the optimized G4⋅⋅⋅K^+^ indicates that the simulated K^+^-containing G-tetrad is larger than a real one, so compressive stress can be exerted in presence of DNA backbones. Considering the energy loss by additional compression, the actual G4⋅⋅⋅K^+^ should be less stable than our calculations suggests, whereas the G4⋅⋅⋅Na^+^ would not be compressed, i.e., no further destabilization.

The length of the bond between a metal ion and a partially-charged oxygen of guanine (M^+^⋅⋅⋅O) was next considered, and the calculated M^+^⋅⋅⋅O lengths are shorter than the experimentally-determined ones (Figure 5). The difference is a result of stacking interactions between G-tetrad layers. An isolated G-tetrad experiences no π–π stacking interaction and importantly, provides only four oxygen atoms for a metal ion to form M^+^⋅⋅⋅O bonds. Therefore, in a double G-tetrad structure, i.e., a G-quadruplex, the metal ion can be located at its correct position; this change would narrow the difference between computed and measured M^+^⋅⋅⋅O lengths.

### Four-base/two-layer structures: G-quadruplex and donor–acceptor bonds in metal ion binding

Two or more G-tetrad layers can be stacked by π–π stacking interactions; the result is called a G-quadruplex. We further simulated G-quadruplex structures with monovalent cations, Li^+^, Na^+^, and K^+^, to investigate the stacking effect between the G-tetrads (Supplementary Figures S16 and S17). In constructing an initial simulation model, we positioned a metal ion in the center of the optimized G-quadruplex, so that the cation was out-of-plane with respect to both G-tetrad layers. This structural arrangement resembles the G-quadruplex formation reported in crystallography and NMR data (45,46). Simulated G-quadruplexes with metal ions (G4⋅⋅⋅M^+^⋅⋅⋅G4) show similar planarity and TA values to each other, so we focused on optimization of metal ion sites and alteration of relevant bond lengths.

Computational results of G4⋅⋅⋅M^+^⋅⋅⋅G4 reveal that the position of monovalent cation within an optimized G-quadruplex depends on the ion size (Supplementary Figure S17), as was observed in computational results of G4⋅⋅⋅M^+^. In water, Li^+^ does not remain in the center of G-quadruplex; in the two stacked G-tetrads, the Li^+^ occupies the central space in only one tetrad layer. Construction of out-of-plane G4⋅⋅⋅Li^+^⋅⋅⋅G4 requires long Li^+^⋅⋅⋅O bond lengths due to the small size of Li^+^, so its most stable arrangement is to have four Li^+^⋅⋅⋅O bonds by forming an in-plane G4⋅⋅⋅Li^+^ and an empty G-tetrad. In contrast, the large K^+^ is out-of-plane with respect to both G-tetrad layers, and being centered in the G-quadruplex is maintained. As a result, the optimal G4⋅⋅⋅K^+^⋅⋅⋅G4 structure has eight K^+^⋅⋅⋅O bonds and is further stabilized. The order of radius of metal ion is Li^+^ < Na^+^ < K^+^, so Na^+^ can be confined either in a G-tetrad or in a G-quadruplex; this inference has been confirmed by crystallography and NMR studies (39). G-quadruplexes that contain in-plane and out-of-plane Na^+^ have Δ*E_int_* = –105.8 and –106.3 kcal/mol, respectively (Supplementary Figure S18), and because of the similar energetic stability, we believe that both structures can be observable.

**Figure 6.**
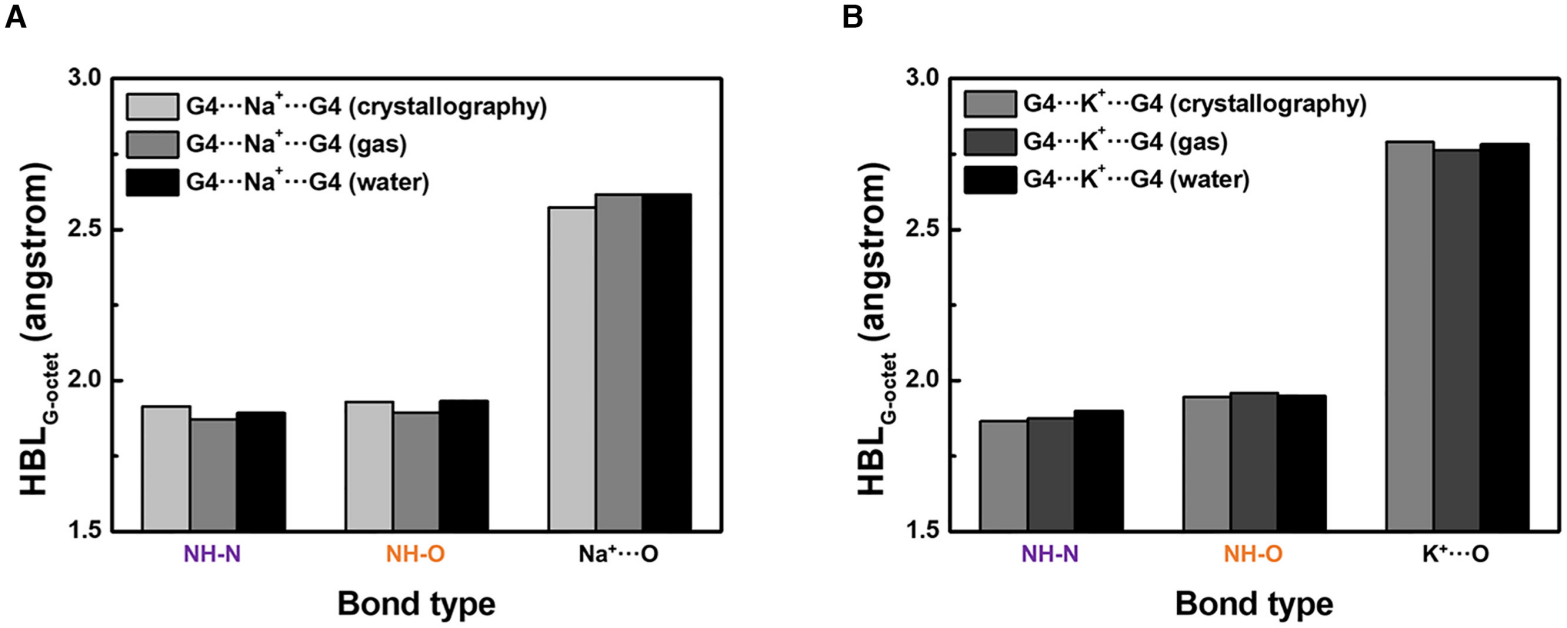
NH-N (purple), NH-O (orange), and M^+^⋅⋅⋅O (black) bonds of G-quadruplex. Mean bond length of G4⋅⋅⋅Na^+^⋅⋅⋅G4 (**A**) and G4⋅⋅⋅K^+^⋅⋅⋅G4 (**B**). The experimentally-determined bond length of Na^+^⋅⋅⋅O is originated from out-of-plane G4⋅⋅⋅Na^+^ quadruplexes (PDB ID: 1JB7, 2AQY, 143D, 186D, 201D, 230D and 352D).

Even though metal ions are located in optimal positions of G-quadruplexes, the stability orders determined by the overall interaction energy Δ*E_int_* of G4⋅⋅⋅M^+^⋅⋅⋅G4 in both phases are G4⋅⋅⋅Na^+^⋅⋅⋅G4 > G4⋅⋅⋅K^+^⋅⋅⋅G4 > G4⋅⋅⋅Li^+^⋅⋅⋅G4 (Supplementary Table S1), which does not match the general trend of nature. The formation energies of G-tetrads and their stacking are similar among three different monovalent cations (Supplementary Table S1), so the Δ*E_int_* values heavily rely on the sum of M^+^⋅⋅⋅O bond energies. The computed M^+^⋅⋅⋅O mean lengths of G4⋅⋅⋅M^+^⋅⋅⋅G4 structures are close to the experimentally-measured ones (39–46,48,49) (Figure 6A, B and Supplementary Table S9); this result indicates that the G-tetrad stacking yields the calculated ionic bonds that agree well with an actual G-quadruplex. In our calculations, both Na^+^- and K^+^-containing G-quadruplexes have eight M^+^⋅⋅⋅O bonds, but Δ*E_ionic_* of the Na^+^⋅⋅⋅O bond (-4.88 kcal/mol) is lower than that of K^+^⋅⋅⋅O bond (–4.63 kcal/mol). The in-plane Li^+^-containing G-quadruplex, which has only four Li^+^⋅⋅⋅O bonds, also has relatively higher Δ*E_ionic_* (–4.10 kcal/mol). In presence of K^+^, the calculated NH-N length of the G4⋅⋅⋅K^+^⋅⋅⋅G4 is even longer than the experimentally-determined length (Figure 6B). This means that if phosphate backbones are further included, the simulated G-quadruplex can be compressively stressed, as was observed for the G-tetrad.

To mimic the interaction of G-quadruplex accurately, metal-ion hydration becomes the key interaction to be considered. The COSMO water continuum was successfully applied to calculate a variety of base-base interactions, but the computation of interactions in a G-quadruplex demands an additional interaction among bases and metal ions. Metal cations are always coordinated with aqua ligands in water and therefore must be dehydrated before entering the G-quadruplex (88). According to the hydration property of metal ion in solution, we adopted a chemical equation wherein a hydrated metal ion reacts with a G-quadruplex to form a metal ion-containing G-quadruplex by the release of water molecules, and the net energy Δ*E_M+binding_* of this reaction determines whether the metal ion enters the G-quadruplex.

$$\begin{equation*}{{\rm{M}}^ + } + {\rm{n}}{{\rm{H}}_2}{\rm{O}}\mathop{-\!\!\!-\!\!\!-\!\!\!-\!\!\!-\!\!\!-\!\!\!-\!\!\!\longrightarrow}^{{{\Delta {E_{hydration}}}}} {\rm{M}}\left[ {{{\rm{H}}_2}{\rm{O}}} \right]_{\rm{n}}^ + ,\end{equation*}$$

$$\begin{equation*}8{\rm{G}} + {{\rm{M}}^ + }\mathop{-\!\!\!-\!\!\!-\!\!\!-\!\!\!\longrightarrow}^{{{\Delta {E_{ionic}}}}} {\rm{M}}\left[ {\rm{G}} \right]_8^ + ,\end{equation*}$$

$$\begin{equation*}{\rm{M}}\left[ {{{\rm{H}}_2}{\rm{O}}} \right]_{\rm{n}}^ + + 8{\rm{G}} \mathop{-\!\!\!-\!\!\!-\!\!\!-\!\!\!-\!\!\!-\!\!\!-\!\!\!-\!\!\!\longrightarrow}^{{\Delta {E_{{M^ + }binding}}}} {\rm{M}}\left[ {\rm{G}} \right]_8^ + + {\rm{n}}{{\rm{H}}_2}{\rm{O,}}\end{equation*}$$

$$\begin{equation*}\therefore {\rm{\Delta }}{E_{{M^ + }binding}} = {\rm{\Delta }}{E_{ionic}} - {\rm{\Delta }}{E_{hydration}}\end{equation*}$$

**Figure 7.**
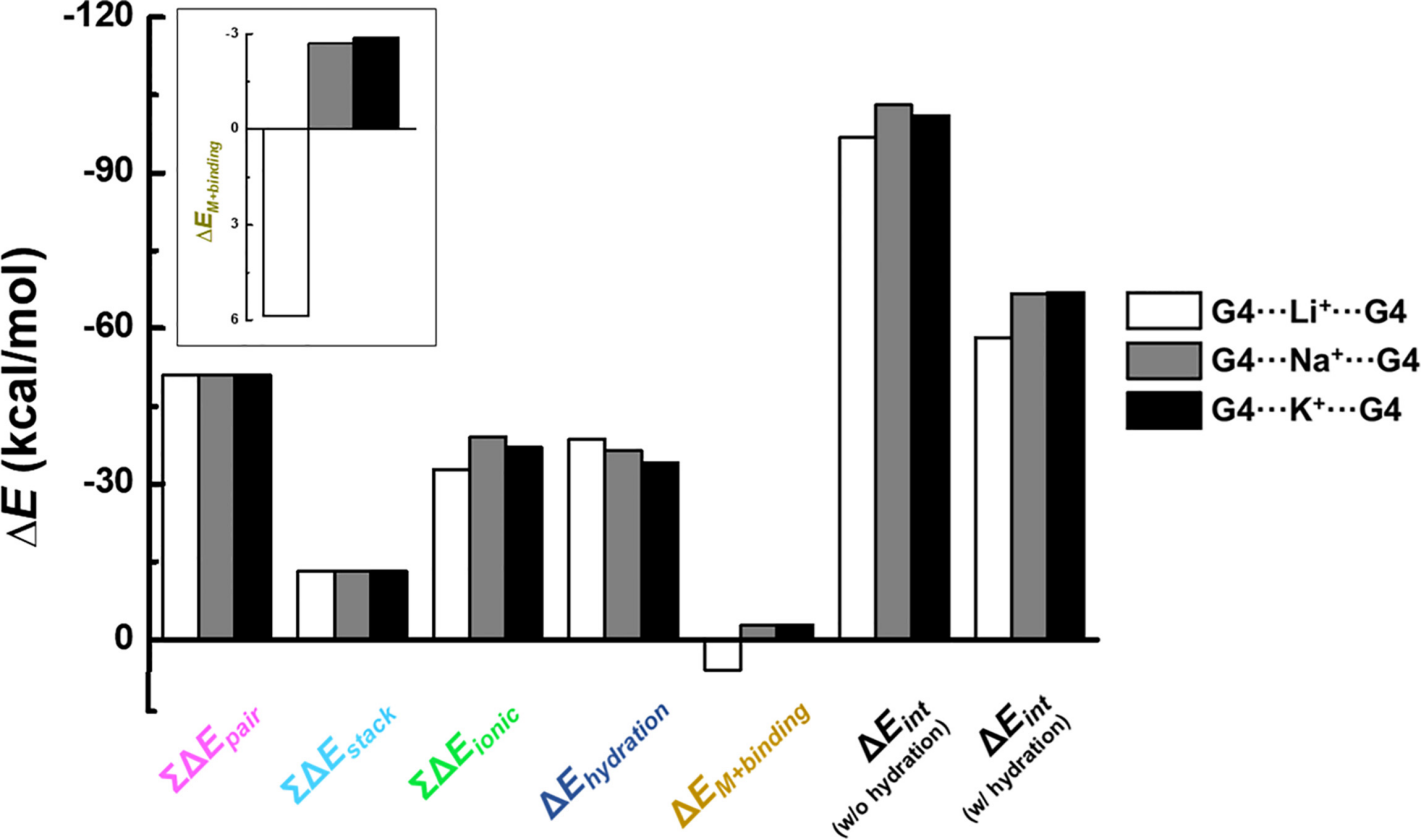
Energy components of G4⋅⋅⋅Li^+^⋅⋅⋅G4 (white columns), G4⋅⋅⋅Na^+^⋅⋅⋅G4 (gray columns), and G4⋅⋅⋅K^+^⋅⋅⋅G4 (black columns). Δ*E_int_* (w/o hydration) is summation of ΣΔ*E_pair_*, ΣΔ*E_stack_* and ΣΔ*E_ionic_*, and Δ*E_int_* (w/ hydration) is obtained by subtracting Δ*E_hydration_* from Δ*E_int_* (w/o hydration). Inset shows detail Δ*E_M+binding_*; specifically, Li^+^ shows a positive value because its ΣΔ*E_ionic_* value is higher than Δ*E_hydration_*value.

Δ*E_M+binding_* is the difference between Δ*E_ionic_* and the metal ion hydration energy Δ*E_hydration_*, so we next calculated Δ*E_hydration_* values. First, the coordination number of the monovalent metal ions should be identified because the Δ*E_hydration_* depends on the number of water molecules surrounding the metal ions. From previous computational and experimental studies (89–93), the coordination numbers by water are widely accepted to be 4 for Li^+^, 5 for Na^+^, and 6 for K^+^. We applied those numbers in our model of hydrated metal ion structures to calculate Δ*E_hydration_* values (Supplementary Figure S19; Table S10) and then obtained Δ*E_M+binding_* values of different Li^+^, Na^+^, and K^+^ within the G-quadruplex (Figure 7, inset). The calculated stability order of hydrated ions by Δ*E_hydration_* was Li^+^ > Na^+^ > K^+^, which is consistent with the observation that the affinity for water molecules decreases as ion size increases (85). Importantly, Δ*E_hydration_* values of Na^+^ and K^+^ differ by 2.22 kcal/mol, whereas their Δ*E_ionic_* values differ by only 0.25 kcal/mol. From these results, we can conclude that the stability order of G-quadruplexes by Δ*E_M+binding_* is G4⋅⋅⋅K^+^⋅⋅⋅G4 > G4⋅⋅⋅Na^+^⋅⋅⋅G4 > G4⋅⋅⋅Li^+^⋅⋅⋅G4. This result (with hydration) corrects the Δ*Eint* order to G4⋅⋅⋅K^+^⋅⋅⋅G4 > G4⋅⋅⋅Na^+^⋅⋅⋅G4 > G4⋅⋅⋅Li^+^⋅⋅⋅G4 (Figure 7; Supplementary Table S11), which agrees well with the actual stability tendency of G-quadruplexes in nature. This successful correction indicates that the reason why K^+^ binding to G-quadruplex is the strongest is that K^+^ is the most easily dehydrated.

Our calculation results reveal the importance of metal ion hydration in determining the G-quadruplex stability, which has been highlighted by previous experimental studies (94). Moreover, from this hydration-dependent stability, we infer that if different solvents or metal-binding ligands are introduced, G-quadruplex-favoring metal ions would be changed.

## CONCLUSION

In this study, we explored fundamental interactions of DNA duplexes, triplexes, and quadruplexes by calculating key interactions (*e.g*., hydrogen bonding, π-π stacking, ion binding, and metal-ion hydration) that strongly correlate with the actual DNA characteristics. To clearly identify the effect of each interaction, the involved interactions in molecular structure formation were componentized as independent terms in our *ab initio* calculations. In general, our simulated DNA structures and calculated energies agreed well with the actual behaviors of DNA; for example, the order in the interaction energy of simulated duplexes matches well with that obtained previously using nearest-neighbor thermodynamics (68). The agreement between computational and experimental results suggests that our improved *ab initio* calculations based on itemized interactions have potential to mimic natural DNA well.

The advantage of identical computation conditions in duplex, triplex and quadruplex calculations is striking. Regardless of the number of bases and layers, all calculated interaction energies are comparable among the simulated structures. Accordingly, we successfully obtained meaningful information:

First, Δ*E_pair_* of pairs and Δ*E_Hoogsteen_* of triads can be compared, and the resulting stability order is G–C (Δ*E_pair_* = –11.45 kcal/mol) > C^+^•G (Δ*E_Hoogsteen_* = –11.04 kcal/mol) > A–T (Δ*E_pair_* = -6.80 kcal/mol) > T•A (Δ*E_Hoogsteen_* = –6.50 kcal/mol). The energy difference between G–C and C^+^•G is attributed to the number of hydrogen bonds: the G–C Watson–Crick pair has three hydrogen bonds, but C^+^•G Hoogsteen pair has only two. In addition, the canonical A–T Watson–Crick pair shows the greater stability than the non-canonical T•A Hoogsteen pair.

Second, the simulated triplexes have a wider range of Δ*E_stack_* values (–3.77 to –13.07 kcal/mol) than do the duplexes (–4.04 to –5.46 kcal/mol). This observation suggests that in triple helices, the nucleobase pairs display strong sequence-dependence in structural designs, whereas in double-stranded helices, the sequence of bases is unconstrained.

Third, in G-tetrad and G-quadruplex structures that capture identical cations, the stability order by Δ*E_ionic_* is G4⋅⋅⋅Na^+^ (–6.92 kcal/mol) > G4⋅⋅⋅Na^+^⋅⋅⋅G4 (–4.88 kcal/mol), and G4⋅⋅⋅K^+^ (–5.07 kcal/mol) > G4⋅⋅⋅K^+^⋅⋅⋅G4 (–4.88 kcal/mol). Δ*E_ionic_* is affected by M^+^⋅⋅⋅O length, so the relatively large K^+^ that cannot be in-plane on a G-tetrad layer shows similar K^+^⋅⋅⋅O length in both the G-tetrad and the G-quadruplex; this result explains the prevalence of K^+^-containing G-quadruplex over G-tetrad in nature.

Fourth, the most encouraging result is the identification of metal ion hydration as an explanation of the K^+^ preference in G-quadruplex formation. When considering only the water continuum, the stability order by the calculated Δ*E_int_* is G4⋅⋅⋅Na^+^⋅⋅⋅G4 > G4⋅⋅⋅K^+^⋅⋅⋅G4 > G4⋅⋅⋅Li^+^⋅⋅⋅G4, while G4⋅⋅⋅K^+^⋅⋅⋅G4 > G4⋅⋅⋅Na^+^⋅⋅⋅G4 > G4⋅⋅⋅Li^+^⋅⋅⋅G4 is observed in nature. Addition of the dehydration effect successfully corrects the calculated order to the natural order. To our best knowledge, this consideration of both base-base and ion-water interactions has yielded the first successful correction of G-quadruplex ion preference by *ab initio* calculations.

Most donor-acceptor interactions in DNA construction are among nucleobases, but to be accurate, *ab initio* calculations must include structural components such as sugar rings and phosphate backbones, and also consider environmental conditions including surrounding ions and water molecules. Several analytical factors such as HBL, GVA, and TA were effective to indirectly examine the effect of DNA backbones, but an improvement of the simulation to consider the backbones is inevitably necessary. It is well known that the metal ions such as Na^+^ and Mg^2+^ neutralize negative charges of phosphodiester backbones (95,96), and the DNA backbones determine syn- and anti-type nucleoside, and strand directionality, resulting in formation of 3D complex DNA structures. Moreover, G-quadruplexes can show parallel, anti-parallel, or even hybrid topologies according to the length and the sequence of DNA strand, and the different G-quadruplex topologies can be also affected by syn- and anti-conformations of guanines (97). Furthermore, different shapes of DNA duplexes such as A-, B- or Z-form DNA are influenced by the syn- and anti-conformations, and even sugar puckers (98). Thus, only the calculations of backbone-including DNA structures will enable quantitative analysis of the effects of metal ions, backbone directionality, syn- and anti-nucleosides, and sugar puckers.

Additionally, studies of modified nucleobases have observed that hydration of hydrogen-bonding acceptors in minor grooves can influence the stability of helix (99,100). The relatively small system such as ours tend to be affected by this hydration effect, so investigation of nucleobase interactions in presence of water molecules can give more accurate and meaningful results. Continuing advances in understanding of DNA structures and environment effects will enable calculations, designs, and programming of complicated DNA constructs, for use in DNA nanotechnology, in the near future.

## Supplementary Material

gkab285_Supplemental_Files
