# Supplementary material for: A unified computational view of DNA duplex, triplex, quadruplex and their donor–acceptor interactions
Source: Nucleic Acids Res. 2021 Apr 24;49(9):4919–33. doi: 10.1093/nar/gkab285 (PMC8136788; doi:10.1093/nar/gkab285)
Supplement: gkab285_Supplemental_Files [file gkab285_supplemental_files.zip › Revised Cartesian coordinates.docx]

Cartesian coordinates of optimized structures in the gas phase by M05-2X/6-31G(d,p)// M05-2X/6-31G(d,p).

A-A

| 7 | 3.069 | -1.343 | -0.009 |
| --- | --- | --- | --- |
| 6 | 2.580 | -0.092 | -0.002 |
| 6 | 1.178 | 0.076 | 0.002 |
| 6 | 0.421 | -1.089 | 0.000 |
| 7 | 0.878 | -2.347 | -0.006 |
| 6 | 2.205 | -2.369 | -0.010 |
| 7 | -0.879 | -0.653 | 0.005 |
| 6 | -0.835 | 0.721 | 0.010 |
| 7 | 0.378 | 1.200 | 0.009 |
| 7 | 3.416 | 0.953 | -0.001 |
| 1 | 2.663 | -3.352 | -0.015 |
| 1 | -1.737 | 1.313 | 0.015 |
| 1 | 4.423 | 0.811 | -0.003 |
| 1 | 3.019 | 1.875 | 0.005 |
| 1 | -1.695 | -1.239 | 0.005 |
| 7 | 6.416 | 0.332 | -0.007 |
| 6 | 7.558 | 1.028 | -0.006 |
| 6 | 8.776 | 0.323 | 0.000 |
| 6 | 8.673 | -1.064 | 0.006 |
| 7 | 7.550 | -1.789 | 0.005 |
| 6 | 6.473 | -1.016 | -0.002 |
| 7 | 9.973 | -1.501 | 0.011 |
| 6 | 10.768 | -0.379 | 0.009 |
| 7 | 10.091 | 0.736 | 0.002 |
| 7 | 7.527 | 2.373 | -0.012 |
| 1 | 5.505 | -1.507 | -0.003 |
| 1 | 11.844 | -0.452 | 0.012 |
| 1 | 6.644 | 2.849 | -0.022 |
| 1 | 8.388 | 2.887 | -0.015 |
| 1 | 10.269 | -2.461 | 0.016 |

A-T

| 8 | 0.084 | -3.207 | 0.000 |
| --- | --- | --- | --- |
| 8 | 2.117 | 0.867 | 0.000 |
| 7 | 2.362 | -3.119 | 0.000 |
| 7 | 1.129 | -1.172 | 0.000 |
| 7 | -0.544 | 2.197 | 0.000 |
| 7 | -3.466 | 3.117 | 0.000 |
| 7 | -5.096 | 1.582 | 0.000 |
| 7 | -3.758 | -0.457 | 0.000 |
| 7 | -1.413 | 0.047 | 0.000 |
| 6 | 4.792 | -0.213 | 0.000 |
| 6 | 2.242 | -0.356 | 0.000 |
| 6 | 3.537 | -1.041 | 0.000 |
| 6 | 3.524 | -2.388 | 0.000 |
| 6 | 1.104 | -2.544 | 0.000 |
| 6 | -4.754 | 2.913 | 0.000 |
| 6 | -1.599 | 1.378 | 0.000 |
| 6 | -2.931 | 1.846 | 0.000 |
| 6 | -3.923 | 0.873 | 0.000 |
| 6 | -2.474 | -0.779 | 0.000 |
| 1 | 4.436 | -2.970 | 0.000 |
| 1 | 2.373 | -4.125 | 0.000 |
| 1 | 0.193 | -0.705 | 0.000 |
| 1 | 0.398 | 1.817 | 0.000 |
| 1 | -0.704 | 3.187 | 0.000 |
| 1 | -5.505 | 3.688 | 0.000 |
| 1 | -6.020 | 1.186 | 0.000 |
| 1 | -2.223 | -1.834 | 0.000 |
| 1 | 4.513 | 0.838 | 0.000 |
| 1 | 5.400 | -0.409 | -0.884 |
| 1 | 5.400 | -0.409 | 0.884 |

A-G

| 7 | 5.786 | 0.596 | 0.000 |
| --- | --- | --- | --- |
| 6 | 5.191 | 1.809 | 0.000 |
| 6 | 3.777 | 1.844 | 0.000 |
| 6 | 3.121 | 0.622 | 0.000 |
| 7 | 3.688 | -0.594 | 0.000 |
| 6 | 5.006 | -0.499 | 0.000 |
| 7 | 1.790 | 0.944 | 0.000 |
| 6 | 1.718 | 2.318 | 0.000 |
| 7 | 2.887 | 2.896 | 0.000 |
| 7 | 5.914 | 2.926 | 0.000 |
| 1 | 5.533 | -1.449 | 0.000 |
| 1 | 0.770 | 2.832 | 0.000 |
| 1 | 6.935 | 2.917 | 0.000 |
| 1 | 5.420 | 3.801 | 0.000 |
| 1 | 1.026 | 0.291 | 0.000 |
| 6 | 12.980 | 2.061 | 0.000 |
| 7 | 12.767 | 0.697 | 0.000 |
| 1 | 13.457 | -0.034 | 0.000 |
| 6 | 11.417 | 0.509 | 0.000 |
| 6 | 10.881 | 1.791 | 0.000 |
| 7 | 11.874 | 2.746 | 0.000 |
| 6 | 9.455 | 1.925 | 0.000 |
| 8 | 8.773 | 2.945 | 0.000 |
| 7 | 8.832 | 0.665 | 0.000 |
| 1 | 7.806 | 0.671 | 0.000 |
| 6 | 9.477 | -0.540 | 0.000 |
| 7 | 10.780 | -0.684 | 0.000 |
| 7 | 8.685 | -1.636 | 0.000 |
| 1 | 13.975 | 2.477 | 0.000 |
| 1 | 7.687 | -1.559 | 0.000 |
| 1 | 9.131 | -2.534 | 0.000 |

A-C

| 7 | 1.526 | -0.294 | 0.000 |
| --- | --- | --- | --- |
| 6 | 0.497 | -1.148 | 0.000 |
| 6 | -0.810 | -0.629 | 0.000 |
| 6 | -0.911 | 0.757 | 0.000 |
| 7 | 0.094 | 1.637 | 0.000 |
| 6 | 1.275 | 1.034 | 0.000 |
| 7 | -2.260 | 1.000 | 0.000 |
| 6 | -2.883 | -0.225 | 0.000 |
| 7 | -2.052 | -1.231 | 0.000 |
| 7 | 0.721 | -2.477 | 0.000 |
| 1 | 2.159 | 1.663 | 0.000 |
| 1 | -3.959 | -0.309 | 0.000 |
| 1 | 1.661 | -2.823 | 0.000 |
| 1 | -0.059 | -3.107 | 0.000 |
| 1 | -2.692 | 1.908 | 0.000 |
| 6 | 6.693 | 0.491 | 0.000 |
| 6 | 7.329 | 1.683 | 0.000 |
| 7 | 6.616 | 2.832 | 0.000 |
| 6 | 5.200 | 2.869 | 0.000 |
| 8 | 4.655 | 3.956 | 0.000 |
| 7 | 4.560 | 1.663 | 0.000 |
| 6 | 5.250 | 0.539 | 0.000 |
| 7 | 4.561 | -0.614 | 0.000 |
| 1 | 7.235 | -0.442 | 0.000 |
| 1 | 8.407 | 1.778 | 0.000 |
| 1 | 3.542 | -0.578 | 0.000 |
| 1 | 5.044 | -1.491 | 0.000 |
| 1 | 7.060 | 3.735 | 0.000 |

T-T

| 6 | -1.120 | -2.921 | 0.000 |
| --- | --- | --- | --- |
| 6 | -0.051 | -1.874 | 0.000 |
| 6 | -0.278 | -0.550 | 0.000 |
| 7 | 0.749 | 0.369 | 0.000 |
| 1 | 0.571 | 1.359 | 0.000 |
| 6 | 2.078 | 0.018 | 0.000 |
| 8 | 2.959 | 0.869 | 0.000 |
| 7 | 2.306 | -1.329 | 0.000 |
| 1 | 3.292 | -1.611 | 0.000 |
| 6 | 1.342 | -2.340 | 0.000 |
| 8 | 1.662 | -3.512 | 0.000 |
| 1 | -1.276 | -0.132 | 0.000 |
| 6 | 7.795 | -2.796 | 0.000 |
| 6 | 7.475 | -1.334 | 0.000 |
| 6 | 8.391 | -0.348 | 0.000 |
| 7 | 8.040 | 0.979 | 0.000 |
| 1 | 8.735 | 1.706 | 0.000 |
| 6 | 6.731 | 1.429 | 0.000 |
| 8 | 6.448 | 2.608 | 0.000 |
| 7 | 5.803 | 0.408 | 0.000 |
| 1 | 4.817 | 0.685 | 0.000 |
| 6 | 6.065 | -0.949 | 0.000 |
| 8 | 5.154 | -1.772 | 0.000 |
| 1 | 9.454 | -0.549 | 0.000 |
| 1 | -2.111 | -2.468 | 0.000 |
| 1 | -1.019 | -3.563 | 0.876 |
| 1 | -1.019 | -3.563 | -0.876 |
| 1 | 8.873 | -2.958 | 0.000 |
| 1 | 7.362 | -3.280 | -0.876 |
| 1 | 7.362 | -3.280 | 0.876 |

G-T

| 6 | -0.084 | 4.248 | -0.059 |
| --- | --- | --- | --- |
| 7 | 0.496 | 2.996 | -0.063 |
| 1 | 1.474 | 2.771 | -0.096 |
| 6 | -0.522 | 2.090 | -0.010 |
| 6 | -1.680 | 2.860 | 0.023 |
| 7 | -1.383 | 4.205 | -0.009 |
| 6 | -2.938 | 2.175 | 0.075 |
| 8 | -4.080 | 2.625 | 0.097 |
| 7 | -2.743 | 0.787 | 0.098 |
| 1 | -3.599 | 0.227 | 0.128 |
| 6 | -1.544 | 0.139 | 0.058 |
| 7 | -0.383 | 0.743 | -0.002 |
| 7 | -1.610 | -1.217 | 0.116 |
| 1 | 0.512 | 5.146 | -0.095 |
| 1 | -2.486 | -1.671 | -0.077 |
| 1 | -0.759 | -1.705 | -0.098 |
| 6 | -10.202 | 1.168 | -0.028 |
| 6 | -8.824 | 0.586 | -0.014 |
| 6 | -8.550 | -0.727 | -0.036 |
| 7 | -7.253 | -1.201 | -0.022 |
| 1 | -7.054 | -2.187 | -0.034 |
| 6 | -6.153 | -0.386 | 0.014 |
| 8 | -5.016 | -0.866 | 0.027 |
| 7 | -6.425 | 0.948 | 0.036 |
| 1 | -5.623 | 1.597 | 0.060 |
| 6 | -7.701 | 1.532 | 0.024 |
| 8 | -7.837 | 2.736 | 0.045 |
| 1 | -9.321 | -1.486 | -0.064 |
| 1 | -10.959 | 0.385 | -0.057 |
| 1 | -10.326 | 1.820 | -0.893 |
| 1 | -10.358 | 1.785 | 0.858 |

C-T

| 6 | 7.081 | 4.066 | 0.000 |
| --- | --- | --- | --- |
| 6 | 6.484 | 2.693 | 0.000 |
| 6 | 7.195 | 1.551 | 0.000 |
| 7 | 6.592 | 0.321 | 0.000 |
| 1 | 7.130 | -0.529 | 0.000 |
| 6 | 5.217 | 0.129 | 0.000 |
| 8 | 4.727 | -0.976 | 0.000 |
| 7 | 4.499 | 1.312 | 0.000 |
| 1 | 3.472 | 1.230 | 0.000 |
| 6 | 5.026 | 2.588 | 0.000 |
| 8 | 4.305 | 3.585 | 0.000 |
| 1 | 8.277 | 1.543 | 0.000 |
| 6 | -0.668 | 2.675 | 0.000 |
| 6 | -1.294 | 1.478 | 0.000 |
| 7 | -0.572 | 0.335 | 0.000 |
| 6 | 0.842 | 0.309 | 0.000 |
| 8 | 1.403 | -0.768 | 0.000 |
| 7 | 1.474 | 1.522 | 0.000 |
| 6 | 0.775 | 2.646 | 0.000 |
| 7 | 1.450 | 3.800 | 0.000 |
| 1 | -1.217 | 3.604 | 0.000 |
| 1 | -2.371 | 1.375 | 0.000 |
| 1 | 2.468 | 3.771 | 0.000 |
| 1 | 0.964 | 4.676 | 0.000 |
| 1 | -1.008 | -0.572 | 0.000 |
| 1 | 8.170 | 4.015 | 0.000 |
| 1 | 6.752 | 4.625 | 0.877 |
| 1 | 6.752 | 4.625 | -0.877 |

G-G

| 6 | 3.995 | 1.850 | 0.000 |
| --- | --- | --- | --- |
| 7 | 2.888 | 1.027 | 0.000 |
| 1 | 1.921 | 1.302 | 0.000 |
| 6 | 3.348 | -0.256 | 0.000 |
| 6 | 4.729 | -0.132 | 0.000 |
| 7 | 5.117 | 1.188 | 0.000 |
| 6 | 5.518 | -1.320 | 0.000 |
| 8 | 6.735 | -1.443 | 0.000 |
| 7 | 4.700 | -2.466 | 0.000 |
| 1 | 5.216 | -3.333 | 0.000 |
| 6 | 3.327 | -2.469 | 0.000 |
| 7 | 2.598 | -1.381 | 0.000 |
| 7 | 2.720 | -3.676 | 0.000 |
| 1 | 3.900 | 2.924 | 0.000 |
| 1 | 3.225 | -4.541 | 0.000 |
| 1 | 1.717 | -3.684 | 0.000 |
| 6 | 14.027 | -1.025 | 0.000 |
| 7 | 13.322 | 0.161 | 0.000 |
| 1 | 13.688 | 1.096 | 0.000 |
| 6 | 11.996 | -0.166 | 0.000 |
| 6 | 11.975 | -1.555 | 0.000 |
| 7 | 13.253 | -2.071 | 0.000 |
| 6 | 10.696 | -2.216 | 0.000 |
| 8 | 10.420 | -3.401 | 0.000 |
| 7 | 9.656 | -1.254 | 0.000 |
| 1 | 8.721 | -1.641 | 0.000 |
| 6 | 9.803 | 0.107 | 0.000 |
| 7 | 10.969 | 0.714 | 0.000 |
| 7 | 8.668 | 0.834 | 0.000 |
| 1 | 15.105 | -1.042 | 0.000 |
| 1 | 7.759 | 0.401 | 0.000 |
| 1 | 8.763 | 1.831 | 0.000 |

G-C

| 7 | -2.650 | 0.025 | 0.033 |
| --- | --- | --- | --- |
| 8 | 1.695 | 1.474 | 0.033 |
| 7 | -0.500 | 0.856 | 0.033 |
| 6 | -1.848 | 1.103 | 0.033 |
| 7 | -2.368 | 2.314 | 0.033 |
| 6 | -1.419 | 3.271 | 0.033 |
| 6 | -0.033 | 3.142 | 0.033 |
| 6 | 0.512 | 1.821 | 0.033 |
| 7 | -1.640 | 4.618 | 0.033 |
| 6 | -0.401 | 5.227 | 0.033 |
| 7 | 0.582 | 4.377 | 0.033 |
| 1 | -2.296 | -0.928 | 0.033 |
| 1 | -3.637 | 0.203 | 0.033 |
| 1 | -0.177 | -0.119 | 0.033 |
| 1 | -2.543 | 5.057 | 0.033 |
| 1 | -0.301 | 6.302 | 0.033 |
| 8 | -1.627 | -2.726 | 0.033 |
| 7 | 2.665 | -1.169 | 0.033 |
| 7 | 0.511 | -1.913 | 0.033 |
| 6 | 2.308 | -3.545 | 0.033 |
| 6 | -0.411 | -2.905 | 0.033 |
| 7 | 0.061 | -4.223 | 0.033 |
| 6 | 1.383 | -4.527 | 0.033 |
| 6 | 1.814 | -2.189 | 0.033 |
| 1 | 3.365 | -3.762 | 0.033 |
| 1 | -0.650 | -4.935 | 0.033 |
| 1 | 1.638 | -5.578 | 0.033 |
| 1 | 2.313 | -0.203 | 0.033 |
| 1 | 3.654 | -1.337 | 0.033 |

C-C

| 6 | 1.742 | 2.705 | 0.000 |
| --- | --- | --- | --- |
| 6 | 2.212 | 1.440 | 0.000 |
| 7 | 1.349 | 0.394 | 0.000 |
| 6 | -0.059 | 0.545 | 0.000 |
| 8 | -0.737 | -0.468 | 0.000 |
| 7 | -0.538 | 1.822 | 0.000 |
| 6 | 0.303 | 2.840 | 0.000 |
| 7 | -0.207 | 4.080 | 0.000 |
| 1 | 2.382 | 3.573 | 0.000 |
| 1 | 3.267 | 1.200 | 0.000 |
| 1 | -1.206 | 4.168 | 0.000 |
| 1 | 0.390 | 4.895 | 0.000 |
| 6 | 0.446 | 9.832 | 0.000 |
| 6 | 1.782 | 9.627 | 0.000 |
| 7 | 2.274 | 8.366 | 0.000 |
| 6 | 1.455 | 7.226 | 0.000 |
| 8 | 1.993 | 6.125 | 0.000 |
| 7 | 0.108 | 7.423 | 0.000 |
| 6 | -0.375 | 8.649 | 0.000 |
| 7 | -1.713 | 8.784 | 0.000 |
| 1 | 0.023 | 10.825 | 0.000 |
| 1 | 2.504 | 10.431 | 0.000 |
| 1 | -2.270 | 7.948 | 0.000 |
| 1 | -2.157 | 9.682 | 0.000 |
| 1 | 1.668 | -0.560 | 0.000 |
| 1 | 3.264 | 8.182 | 0.000 |

A-T/A-T

| 8 | -2.679 | -0.891 | -1.877 |
| --- | --- | --- | --- |
| 8 | -0.961 | 3.122 | -0.564 |
| 7 | -4.106 | 0.782 | -1.287 |
| 7 | -1.828 | 1.148 | -1.279 |
| 7 | 1.786 | 2.114 | -1.216 |
| 7 | 4.390 | 0.488 | -1.355 |
| 7 | 4.282 | -1.709 | -1.775 |
| 7 | 1.875 | -1.984 | -1.998 |
| 7 | 0.731 | 0.099 | -1.684 |
| 6 | -3.488 | 4.286 | -0.008 |
| 6 | -1.952 | 2.427 | -0.781 |
| 6 | -3.315 | 2.899 | -0.541 |
| 6 | -4.324 | 2.050 | -0.805 |
| 6 | -2.846 | 0.260 | -1.509 |
| 6 | 5.067 | -0.619 | -1.501 |
| 6 | 1.857 | 0.800 | -1.472 |
| 6 | 3.079 | 0.097 | -1.536 |
| 6 | 2.986 | -1.268 | -1.792 |
| 6 | 0.795 | -1.223 | -1.925 |
| 1 | -5.361 | 2.320 | -0.655 |
| 1 | -4.862 | 0.135 | -1.429 |
| 1 | -0.857 | 0.778 | -1.442 |
| 1 | 0.876 | 2.522 | -1.023 |
| 1 | 2.607 | 2.561 | -0.846 |
| 1 | 6.141 | -0.702 | -1.432 |
| 1 | 4.573 | -2.666 | -1.876 |
| 1 | -0.164 | -1.709 | -2.064 |
| 1 | -3.030 | 5.012 | -0.681 |
| 1 | -2.990 | 4.385 | 0.957 |
| 1 | -4.544 | 4.529 | 0.112 |
| 8 | -0.630 | -3.608 | 0.034 |
| 8 | -2.500 | 0.194 | 1.706 |
| 7 | -2.900 | -3.467 | 0.182 |
| 7 | -1.574 | -1.740 | 0.952 |
| 7 | 0.242 | 1.378 | 1.674 |
| 7 | 3.191 | 2.225 | 1.793 |
| 7 | 4.777 | 0.645 | 1.804 |
| 7 | 3.393 | -1.347 | 1.566 |
| 7 | 1.060 | -0.781 | 1.507 |
| 6 | -5.161 | -0.816 | 1.645 |
| 6 | -2.653 | -0.958 | 1.298 |
| 6 | -3.970 | -1.576 | 1.149 |
| 6 | -4.024 | -2.793 | 0.574 |
| 6 | -1.618 | -2.980 | 0.360 |
| 6 | 4.474 | 1.983 | 1.832 |
| 6 | 1.279 | 0.540 | 1.636 |
| 6 | 2.621 | 0.970 | 1.718 |
| 6 | 3.589 | -0.028 | 1.698 |
| 6 | 2.101 | -1.629 | 1.462 |
| 1 | -4.961 | -3.308 | 0.404 |
| 1 | -2.954 | -4.355 | -0.288 |
| 1 | -0.618 | -1.364 | 1.120 |
| 1 | -0.708 | 1.020 | 1.644 |
| 1 | 0.405 | 2.368 | 1.644 |
| 1 | 5.245 | 2.735 | 1.888 |
| 1 | 5.690 | 0.232 | 1.743 |
| 1 | 1.831 | -2.668 | 1.307 |
| 1 | -5.166 | 0.194 | 1.234 |
| 1 | -5.124 | -0.710 | 2.730 |
| 1 | -6.088 | -1.324 | 1.376 |

A-T/T-A

| 8 | 2.138 | -1.954 | 1.829 |
| --- | --- | --- | --- |
| 8 | 0.712 | 2.364 | 1.617 |
| 7 | 3.692 | -0.285 | 1.863 |
| 7 | 1.446 | 0.226 | 1.771 |
| 7 | -2.108 | 1.437 | 1.507 |
| 7 | -4.831 | 0.040 | 1.195 |
| 7 | -4.903 | -2.198 | 1.128 |
| 7 | -2.535 | -2.709 | 1.364 |
| 7 | -1.226 | -0.706 | 1.513 |
| 6 | 3.370 | 3.476 | 1.571 |
| 6 | 1.661 | 1.586 | 1.694 |
| 6 | 3.062 | 2.010 | 1.692 |
| 6 | 4.002 | 1.047 | 1.755 |
| 6 | 2.395 | -0.764 | 1.821 |
| 6 | -5.598 | -1.013 | 1.088 |
| 6 | -2.287 | 0.114 | 1.447 |
| 6 | -3.560 | -0.486 | 1.318 |
| 6 | -3.578 | -1.877 | 1.280 |
| 6 | -1.399 | -2.038 | 1.467 |
| 1 | 5.059 | 1.273 | 1.710 |
| 1 | 4.407 | -0.991 | 1.828 |
| 1 | 0.462 | -0.104 | 1.695 |
| 1 | -1.167 | 1.824 | 1.528 |
| 1 | -2.907 | 2.033 | 1.382 |
| 1 | -6.672 | -0.991 | 0.989 |
| 1 | -5.274 | -3.131 | 1.082 |
| 1 | -0.481 | -2.614 | 1.527 |
| 1 | 2.456 | 4.006 | 1.310 |
| 1 | 4.121 | 3.661 | 0.801 |
| 1 | 3.744 | 3.886 | 2.511 |
| 8 | -2.146 | -1.865 | -1.907 |
| 8 | -0.708 | 2.441 | -1.557 |
| 7 | -3.694 | -0.192 | -1.859 |
| 7 | -1.445 | 0.308 | -1.773 |
| 7 | 2.107 | 1.503 | -1.457 |
| 7 | 4.827 | 0.094 | -1.175 |
| 7 | 4.899 | -2.144 | -1.191 |
| 7 | 2.533 | -2.646 | -1.468 |
| 7 | 1.225 | -0.638 | -1.554 |
| 6 | -3.348 | 3.553 | -1.434 |
| 6 | -1.656 | 1.665 | -1.649 |
| 6 | -3.055 | 2.092 | -1.613 |
| 6 | -4.001 | 1.136 | -1.696 |
| 6 | -2.399 | -0.676 | -1.851 |
| 6 | 5.593 | -0.961 | -1.100 |
| 6 | 2.286 | 0.178 | -1.447 |
| 6 | 3.557 | -0.426 | -1.329 |
| 6 | 3.575 | -1.817 | -1.343 |
| 6 | 1.398 | -1.971 | -1.556 |
| 1 | -5.056 | 1.363 | -1.630 |
| 1 | -4.411 | -0.897 | -1.844 |
| 1 | -0.462 | -0.027 | -1.716 |
| 1 | 1.166 | 1.891 | -1.472 |
| 1 | 2.905 | 2.095 | -1.314 |
| 1 | 6.666 | -0.944 | -0.990 |
| 1 | 5.270 | -3.078 | -1.179 |
| 1 | 0.481 | -2.544 | -1.644 |
| 1 | -2.513 | 4.028 | -0.924 |
| 1 | -3.497 | 4.066 | -2.387 |
| 1 | -4.258 | 3.696 | -0.849 |

G-C/A-T

| 8 | -3.153 | 1.407 | 1.819 |
| --- | --- | --- | --- |
| 8 | -1.952 | -2.681 | 0.190 |
| 7 | -4.773 | -0.132 | 1.386 |
| 7 | -2.552 | -0.662 | 1.041 |
| 7 | 0.950 | -2.126 | 0.965 |
| 7 | 3.666 | -1.004 | 1.856 |
| 7 | 3.806 | 1.135 | 2.503 |
| 7 | 1.483 | 1.814 | 2.237 |
| 7 | 0.131 | -0.005 | 1.450 |
| 6 | -4.616 | -3.627 | -0.051 |
| 6 | -2.842 | -1.919 | 0.547 |
| 6 | -4.264 | -2.269 | 0.471 |
| 6 | -5.157 | -1.362 | 0.909 |
| 6 | -3.455 | 0.286 | 1.443 |
| 6 | 4.453 | -0.055 | 2.280 |
| 6 | 1.159 | -0.873 | 1.384 |
| 6 | 2.428 | -0.399 | 1.787 |
| 6 | 2.490 | 0.933 | 2.182 |
| 6 | 0.342 | 1.259 | 1.860 |
| 1 | -6.222 | -1.553 | 0.911 |
| 1 | -5.447 | 0.539 | 1.713 |
| 1 | -1.533 | -0.408 | 1.131 |
| 1 | 0.061 | -2.345 | 0.529 |
| 1 | 1.758 | -2.663 | 0.691 |
| 1 | 5.512 | -0.162 | 2.461 |
| 1 | 4.207 | 2.004 | 2.807 |
| 1 | -0.542 | 1.886 | 1.875 |
| 1 | -4.154 | -4.400 | 0.565 |
| 1 | -4.230 | -3.762 | -1.063 |
| 1 | -5.696 | -3.776 | -0.059 |
| 7 | 2.158 | 2.745 | -0.986 |
| 8 | 0.656 | -1.453 | -2.061 |
| 7 | 1.504 | 0.599 | -1.526 |
| 6 | 2.509 | 1.453 | -1.157 |
| 7 | 3.765 | 1.081 | -0.998 |
| 6 | 3.928 | -0.242 | -1.203 |
| 6 | 2.982 | -1.203 | -1.540 |
| 6 | 1.636 | -0.774 | -1.748 |
| 7 | 5.093 | -0.940 | -1.065 |
| 6 | 4.792 | -2.268 | -1.276 |
| 7 | 3.540 | -2.465 | -1.569 |
| 1 | 1.181 | 2.990 | -0.832 |
| 1 | 2.855 | 3.309 | -0.535 |
| 1 | 0.538 | 0.953 | -1.567 |
| 1 | 5.973 | -0.548 | -0.783 |
| 1 | 5.546 | -3.036 | -1.202 |
| 8 | -0.672 | 3.425 | -0.569 |
| 7 | -2.013 | -0.581 | -2.296 |
| 7 | -1.312 | 1.427 | -1.477 |
| 6 | -3.694 | 1.028 | -1.692 |
| 6 | -1.550 | 2.651 | -0.947 |
| 7 | -2.883 | 3.057 | -0.835 |
| 6 | -3.919 | 2.255 | -1.171 |
| 6 | -2.315 | 0.625 | -1.821 |
| 1 | -4.503 | 0.370 | -1.971 |
| 1 | -3.024 | 3.934 | -0.361 |
| 1 | -4.911 | 2.652 | -1.004 |
| 1 | -1.040 | -0.908 | -2.247 |
| 1 | -2.741 | -1.251 | -2.463 |

G-C/T-A

| 8 | -2.208 | -0.745 | -1.876 |
| --- | --- | --- | --- |
| 8 | 0.139 | 3.110 | -1.310 |
| 7 | -3.326 | 1.105 | -1.128 |
| 7 | -1.076 | 1.221 | -1.649 |
| 7 | 2.646 | 1.557 | -1.680 |
| 7 | 4.851 | -0.367 | -0.732 |
| 7 | 4.410 | -2.557 | -0.614 |
| 7 | 2.085 | -2.560 | -1.331 |
| 7 | 1.317 | -0.337 | -1.801 |
| 6 | -2.132 | 4.634 | -0.467 |
| 6 | -0.955 | 2.540 | -1.273 |
| 6 | -2.183 | 3.181 | -0.826 |
| 6 | -3.289 | 2.412 | -0.729 |
| 6 | -2.206 | 0.437 | -1.576 |
| 6 | 5.311 | -1.548 | -0.407 |
| 6 | 2.510 | 0.228 | -1.557 |
| 6 | 3.565 | -0.622 | -1.171 |
| 6 | 3.260 | -1.975 | -1.079 |
| 6 | 1.167 | -1.665 | -1.673 |
| 1 | -4.221 | 2.796 | -0.335 |
| 1 | -4.096 | 0.508 | -0.839 |
| 1 | -0.208 | 0.702 | -1.869 |
| 1 | 1.814 | 2.144 | -1.702 |
| 1 | 3.530 | 1.962 | -1.432 |
| 1 | 6.299 | -1.742 | -0.020 |
| 1 | 4.522 | -3.528 | -0.381 |
| 1 | 0.162 | -2.028 | -1.862 |
| 1 | -1.951 | 5.246 | -1.353 |
| 1 | -1.306 | 4.837 | 0.217 |
| 1 | -3.065 | 4.957 | -0.005 |
| 7 | 0.343 | -2.907 | 1.342 |
| 8 | -2.561 | 0.621 | 1.772 |
| 7 | -1.201 | -1.201 | 1.556 |
| 6 | -0.914 | -2.473 | 1.127 |
| 7 | -1.798 | -3.265 | 0.552 |
| 6 | -2.992 | -2.669 | 0.405 |
| 6 | -3.402 | -1.407 | 0.814 |
| 6 | -2.430 | -0.556 | 1.421 |
| 7 | -4.067 | -3.183 | -0.262 |
| 6 | -5.035 | -2.209 | -0.278 |
| 7 | -4.676 | -1.128 | 0.358 |
| 1 | 1.083 | -2.239 | 1.553 |
| 1 | 0.607 | -3.698 | 0.780 |
| 1 | -0.423 | -0.604 | 1.861 |
| 1 | -4.085 | -4.071 | -0.732 |
| 1 | -5.983 | -2.359 | -0.771 |
| 8 | 2.555 | -1.040 | 1.729 |
| 7 | -0.395 | 2.439 | 1.988 |
| 7 | 1.055 | 0.678 | 1.897 |
| 6 | 1.925 | 2.929 | 1.626 |
| 6 | 2.300 | 0.167 | 1.727 |
| 7 | 3.350 | 1.064 | 1.528 |
| 6 | 3.157 | 2.404 | 1.461 |
| 6 | 0.851 | 1.992 | 1.835 |
| 1 | 1.742 | 3.990 | 1.560 |
| 1 | 4.228 | 0.646 | 1.250 |
| 1 | 4.035 | 3.006 | 1.268 |
| 1 | -1.174 | 1.775 | 2.015 |
| 1 | -0.591 | 3.411 | 1.842 |

G-C/G-C

| 7 | -0.094 | -2.439 | 1.426 |
| --- | --- | --- | --- |
| 8 | -2.630 | 1.362 | 1.700 |
| 7 | -1.471 | -0.601 | 1.598 |
| 6 | -1.319 | -1.918 | 1.249 |
| 7 | -2.300 | -2.664 | 0.770 |
| 6 | -3.448 | -1.970 | 0.646 |
| 6 | -3.717 | -0.646 | 0.963 |
| 6 | -2.639 | 0.155 | 1.453 |
| 7 | -4.601 | -2.431 | 0.077 |
| 6 | -5.479 | -1.374 | 0.029 |
| 7 | -4.983 | -0.284 | 0.546 |
| 1 | 0.706 | -1.851 | 1.648 |
| 1 | 0.097 | -3.300 | 0.945 |
| 1 | -0.624 | -0.068 | 1.829 |
| 1 | -4.739 | -3.357 | -0.288 |
| 1 | -6.466 | -1.474 | -0.394 |
| 8 | 2.349 | -0.901 | 2.003 |
| 7 | -0.183 | 2.854 | 1.354 |
| 7 | 1.042 | 0.963 | 1.751 |
| 6 | 2.201 | 3.037 | 1.227 |
| 6 | 2.228 | 0.317 | 1.839 |
| 7 | 3.392 | 1.081 | 1.736 |
| 6 | 3.373 | 2.402 | 1.406 |
| 6 | 1.002 | 2.259 | 1.435 |
| 1 | 2.147 | 4.079 | 0.953 |
| 1 | 4.250 | 0.559 | 1.793 |
| 1 | 4.334 | 2.889 | 1.305 |
| 1 | -1.034 | 2.302 | 1.484 |
| 1 | -0.278 | 3.700 | 0.817 |
| 7 | -0.660 | -2.043 | -1.957 |
| 8 | 1.108 | 2.177 | -1.586 |
| 7 | 0.126 | 0.128 | -1.792 |
| 6 | -0.946 | -0.725 | -1.806 |
| 7 | -2.193 | -0.325 | -1.704 |
| 6 | -2.293 | 1.008 | -1.512 |
| 6 | -1.281 | 1.962 | -1.487 |
| 6 | 0.064 | 1.512 | -1.628 |
| 7 | -3.438 | 1.695 | -1.234 |
| 6 | -3.064 | 2.998 | -1.006 |
| 7 | -1.788 | 3.208 | -1.168 |
| 1 | 0.262 | -2.360 | -1.669 |
| 1 | -1.419 | -2.635 | -1.656 |
| 1 | 1.068 | -0.274 | -1.754 |
| 1 | -4.300 | 1.258 | -0.929 |
| 1 | -3.787 | 3.745 | -0.721 |
| 8 | 1.914 | -2.991 | -0.766 |
| 7 | 3.735 | 1.110 | -1.613 |
| 7 | 2.806 | -0.934 | -1.224 |
| 6 | 5.133 | -0.591 | -0.638 |
| 6 | 2.854 | -2.200 | -0.739 |
| 7 | 4.069 | -2.636 | -0.199 |
| 6 | 5.169 | -1.852 | -0.148 |
| 6 | 3.868 | -0.141 | -1.166 |
| 1 | 6.005 | 0.045 | -0.629 |
| 1 | 4.057 | -3.562 | 0.195 |
| 1 | 6.056 | -2.290 | 0.289 |
| 1 | 2.790 | 1.489 | -1.752 |
| 1 | 4.504 | 1.749 | -1.535 |

G-C/C-G

| 7 | 0.217 | -2.411 | 1.729 |
| --- | --- | --- | --- |
| 8 | -2.518 | 1.270 | 1.682 |
| 7 | -1.242 | -0.625 | 1.702 |
| 6 | -1.018 | -1.956 | 1.460 |
| 7 | -1.944 | -2.781 | 1.002 |
| 6 | -3.114 | -2.155 | 0.781 |
| 6 | -3.457 | -0.828 | 1.003 |
| 6 | -2.442 | 0.054 | 1.486 |
| 7 | -4.227 | -2.711 | 0.218 |
| 6 | -5.155 | -1.708 | 0.082 |
| 7 | -4.733 | -0.564 | 0.542 |
| 1 | 0.993 | -1.764 | 1.874 |
| 1 | 0.437 | -3.312 | 1.341 |
| 1 | -0.437 | -0.027 | 1.941 |
| 1 | -4.305 | -3.662 | -0.098 |
| 1 | -6.122 | -1.886 | -0.362 |
| 8 | 2.463 | -0.641 | 1.890 |
| 7 | -0.252 | 3.027 | 1.785 |
| 7 | 1.084 | 1.180 | 1.862 |
| 6 | 2.068 | 3.297 | 1.215 |
| 6 | 2.283 | 0.564 | 1.695 |
| 7 | 3.359 | 1.336 | 1.263 |
| 6 | 3.237 | 2.653 | 0.992 |
| 6 | 0.960 | 2.483 | 1.628 |
| 1 | 1.957 | 4.354 | 1.028 |
| 1 | 4.163 | 0.815 | 0.923 |
| 1 | 4.116 | 3.141 | 0.597 |
| 1 | -1.064 | 2.415 | 1.890 |
| 1 | -0.400 | 3.986 | 1.532 |
| 7 | -0.225 | -2.382 | -1.734 |
| 8 | 2.524 | 1.289 | -1.680 |
| 7 | 1.241 | -0.601 | -1.705 |
| 6 | 1.012 | -1.933 | -1.467 |
| 7 | 1.937 | -2.763 | -1.015 |
| 6 | 3.109 | -2.143 | -0.796 |
| 6 | 3.458 | -0.817 | -1.015 |
| 6 | 2.444 | 0.072 | -1.490 |
| 7 | 4.222 | -2.707 | -0.241 |
| 6 | 5.156 | -1.709 | -0.104 |
| 7 | 4.737 | -0.560 | -0.558 |
| 1 | -0.998 | -1.731 | -1.881 |
| 1 | -0.449 | -3.281 | -1.346 |
| 1 | 0.438 | -0.001 | -1.938 |
| 1 | 4.297 | -3.659 | 0.072 |
| 1 | 6.124 | -1.893 | 0.334 |
| 8 | -2.467 | -0.610 | -1.897 |
| 7 | 0.257 | 3.049 | -1.758 |
| 7 | -1.083 | 1.206 | -1.853 |
| 6 | -2.062 | 3.321 | -1.189 |
| 6 | -2.284 | 0.592 | -1.691 |
| 7 | -3.358 | 1.363 | -1.252 |
| 6 | -3.233 | 2.677 | -0.971 |
| 6 | -0.956 | 2.507 | -1.608 |
| 1 | -1.949 | 4.376 | -0.994 |
| 1 | -4.162 | 0.839 | -0.915 |
| 1 | -4.111 | 3.165 | -0.572 |
| 1 | 1.068 | 2.437 | -1.870 |
| 1 | 0.408 | 4.006 | -1.500 |

C^+^•G-C

| 7 | -2.675 | -0.018 | 0.000 |
| --- | --- | --- | --- |
| 8 | 1.717 | 1.325 | 0.000 |
| 7 | -0.492 | 0.733 | 0.000 |
| 6 | -1.844 | 1.020 | 0.000 |
| 7 | -2.326 | 2.255 | 0.000 |
| 6 | -1.366 | 3.181 | 0.000 |
| 6 | 0.011 | 2.998 | 0.000 |
| 6 | 0.517 | 1.674 | 0.000 |
| 7 | -1.538 | 4.541 | 0.000 |
| 6 | -0.300 | 5.119 | 0.000 |
| 7 | 0.651 | 4.221 | 0.000 |
| 1 | -2.359 | -0.990 | 0.000 |
| 1 | -3.656 | 0.197 | 0.000 |
| 1 | -0.195 | -0.259 | 0.000 |
| 1 | -2.426 | 5.015 | 0.000 |
| 1 | -0.134 | 6.184 | 0.000 |
| 8 | -1.811 | -2.706 | 0.000 |
| 7 | 2.594 | -1.505 | 0.000 |
| 7 | 0.385 | -2.066 | 0.000 |
| 6 | 2.041 | -3.840 | 0.000 |
| 6 | -0.615 | -2.988 | 0.000 |
| 7 | -0.252 | -4.334 | 0.000 |
| 6 | 1.038 | -4.746 | 0.000 |
| 6 | 1.656 | -2.455 | 0.000 |
| 1 | 3.077 | -4.142 | 0.000 |
| 1 | -1.019 | -4.988 | 0.000 |
| 1 | 1.206 | -5.814 | 0.000 |
| 1 | 2.315 | -0.527 | 0.000 |
| 1 | 3.565 | -1.755 | 0.000 |
| 6 | 5.552 | 4.762 | 0.000 |
| 6 | 5.634 | 6.113 | 0.000 |
| 7 | 4.523 | 6.891 | 0.000 |
| 6 | 3.233 | 6.374 | 0.000 |
| 8 | 2.248 | 7.076 | 0.000 |
| 7 | 3.174 | 4.993 | 0.000 |
| 1 | 2.141 | 4.603 | 0.000 |
| 6 | 4.244 | 4.179 | 0.000 |
| 7 | 4.052 | 2.874 | 0.000 |
| 1 | 6.436 | 4.144 | 0.000 |
| 1 | 6.581 | 6.634 | 0.000 |
| 1 | 3.119 | 2.433 | 0.000 |
| 1 | 4.856 | 2.271 | 0.000 |
| 1 | 4.583 | 7.898 | 0.000 |

rC^+^•G-C

| 7 | -2.656 | -0.042 | 0.000 |
| --- | --- | --- | --- |
| 8 | 1.642 | 1.551 | 0.000 |
| 7 | -0.522 | 0.838 | 0.000 |
| 6 | -1.888 | 1.041 | 0.000 |
| 7 | -2.443 | 2.247 | 0.000 |
| 6 | -1.540 | 3.227 | 0.000 |
| 6 | -0.153 | 3.133 | 0.000 |
| 6 | 0.423 | 1.839 | 0.000 |
| 7 | -1.795 | 4.573 | 0.000 |
| 6 | -0.590 | 5.220 | 0.000 |
| 7 | 0.420 | 4.391 | 0.000 |
| 1 | -2.283 | -0.998 | 0.000 |
| 1 | -3.648 | 0.114 | 0.000 |
| 1 | -0.163 | -0.138 | 0.000 |
| 1 | -2.711 | 4.992 | 0.000 |
| 1 | -0.519 | 6.296 | 0.000 |
| 8 | -1.670 | -2.662 | 0.000 |
| 7 | 2.671 | -1.242 | 0.000 |
| 7 | 0.489 | -1.908 | 0.000 |
| 6 | 2.235 | -3.598 | 0.000 |
| 6 | -0.459 | -2.879 | 0.000 |
| 7 | -0.030 | -4.205 | 0.000 |
| 6 | 1.281 | -4.553 | 0.000 |
| 6 | 1.784 | -2.231 | 0.000 |
| 1 | 3.285 | -3.845 | 0.000 |
| 1 | -0.764 | -4.896 | 0.000 |
| 1 | 1.501 | -5.611 | 0.000 |
| 1 | 2.363 | -0.276 | 0.000 |
| 1 | 3.659 | -1.414 | 0.000 |
| 6 | 5.508 | 4.671 | 0.000 |
| 6 | 6.499 | 3.752 | 0.000 |
| 7 | 6.227 | 2.423 | 0.000 |
| 6 | 4.940 | 1.904 | 0.000 |
| 8 | 4.726 | 0.716 | 0.000 |
| 7 | 3.938 | 2.863 | 0.000 |
| 1 | 2.956 | 2.457 | 0.000 |
| 6 | 4.155 | 4.197 | 0.000 |
| 7 | 3.132 | 5.021 | 0.000 |
| 1 | 5.719 | 5.728 | 0.000 |
| 1 | 7.544 | 4.027 | 0.000 |
| 1 | 2.124 | 4.720 | 0.000 |
| 1 | 3.326 | 6.008 | 0.000 |
| 1 | 6.964 | 1.734 | 0.000 |

T•A-T

| 8 | 0.036 | -3.295 | -0.003 |
| --- | --- | --- | --- |
| 8 | 2.111 | 0.757 | 0.001 |
| 7 | 2.314 | -3.230 | -0.002 |
| 7 | 1.102 | -1.270 | -0.001 |
| 7 | -0.569 | 2.140 | 0.002 |
| 7 | -3.537 | 3.011 | 0.002 |
| 7 | -5.132 | 1.465 | 0.000 |
| 7 | -3.762 | -0.552 | -0.001 |
| 7 | -1.423 | -0.015 | 0.000 |
| 6 | 4.775 | -0.349 | 0.001 |
| 6 | 2.223 | -0.465 | 0.000 |
| 6 | 3.512 | -1.165 | 0.000 |
| 6 | 3.484 | -2.511 | -0.001 |
| 6 | 1.063 | -2.641 | -0.002 |
| 6 | -4.827 | 2.798 | 0.002 |
| 6 | -1.617 | 1.320 | 0.001 |
| 6 | -2.963 | 1.759 | 0.001 |
| 6 | -3.943 | 0.775 | 0.000 |
| 6 | -2.472 | -0.853 | -0.001 |
| 1 | 4.390 | -3.104 | -0.001 |
| 1 | 2.314 | -4.236 | -0.003 |
| 1 | 0.174 | -0.789 | 0.000 |
| 1 | 0.364 | 1.740 | 0.002 |
| 1 | -0.683 | 3.147 | 0.003 |
| 1 | -5.568 | 3.583 | 0.002 |
| 1 | -6.047 | 1.050 | 0.000 |
| 1 | -2.207 | -1.905 | -0.002 |
| 1 | 4.505 | 0.705 | 0.002 |
| 1 | 5.381 | -0.549 | -0.883 |
| 1 | 5.381 | -0.551 | 0.885 |
| 6 | 0.300 | 7.915 | 0.000 |
| 6 | -1.132 | 7.482 | 0.000 |
| 6 | -2.186 | 8.319 | -0.002 |
| 7 | -3.486 | 7.870 | -0.002 |
| 1 | -4.263 | 8.509 | -0.003 |
| 6 | -3.825 | 6.530 | 0.000 |
| 8 | -4.982 | 6.146 | 0.000 |
| 7 | -2.742 | 5.690 | 0.001 |
| 1 | -2.965 | 4.674 | 0.002 |
| 6 | -1.408 | 6.044 | 0.001 |
| 8 | -0.531 | 5.188 | 0.003 |
| 1 | -2.067 | 9.395 | -0.003 |
| 1 | 0.380 | 9.002 | -0.002 |
| 1 | 0.816 | 7.519 | -0.876 |
| 1 | 0.815 | 7.521 | 0.877 |

rT•A-T

| 8 | 0.084 | -3.371 | 0.000 |
| --- | --- | --- | --- |
| 8 | 2.219 | 0.650 | 0.000 |
| 7 | 2.363 | -3.340 | 0.000 |
| 7 | 1.179 | -1.363 | 0.000 |
| 7 | -0.441 | 2.067 | 0.000 |
| 7 | -3.396 | 2.982 | 0.000 |
| 7 | -5.013 | 1.461 | 0.000 |
| 7 | -3.674 | -0.576 | 0.000 |
| 7 | -1.328 | -0.074 | 0.000 |
| 6 | 4.866 | -0.496 | 0.000 |
| 6 | 2.313 | -0.574 | 0.000 |
| 6 | 3.591 | -1.293 | 0.000 |
| 6 | 3.544 | -2.639 | 0.000 |
| 6 | 1.120 | -2.733 | 0.000 |
| 6 | -4.688 | 2.789 | 0.000 |
| 6 | -1.501 | 1.263 | 0.000 |
| 6 | -2.840 | 1.722 | 0.000 |
| 6 | -3.835 | 0.753 | 0.000 |
| 6 | -2.389 | -0.897 | 0.000 |
| 1 | 4.441 | -3.244 | 0.000 |
| 1 | 2.348 | -4.345 | 0.000 |
| 1 | 0.258 | -0.868 | 0.000 |
| 1 | 0.486 | 1.653 | 0.000 |
| 1 | -0.540 | 3.075 | 0.000 |
| 1 | -5.417 | 3.586 | 0.000 |
| 1 | -5.935 | 1.060 | 0.000 |
| 1 | -2.139 | -1.953 | 0.000 |
| 1 | 4.612 | 0.562 | 0.000 |
| 1 | 5.469 | -0.706 | -0.884 |
| 1 | 5.469 | -0.706 | 0.884 |
| 6 | -4.415 | 8.955 | 0.000 |
| 6 | -3.299 | 7.958 | 0.000 |
| 6 | -1.991 | 8.274 | 0.000 |
| 7 | -1.000 | 7.319 | 0.000 |
| 1 | -0.026 | 7.570 | 0.000 |
| 6 | -1.261 | 5.965 | 0.000 |
| 8 | -0.366 | 5.131 | 0.000 |
| 7 | -2.590 | 5.655 | 0.000 |
| 1 | -2.824 | 4.641 | 0.000 |
| 6 | -3.659 | 6.537 | 0.000 |
| 8 | -4.810 | 6.124 | 0.000 |
| 1 | -1.646 | 9.300 | 0.000 |
| 1 | -4.030 | 9.975 | 0.000 |
| 1 | -5.049 | 8.813 | 0.877 |
| 1 | -5.049 | 8.813 | -0.876 |

A•A-T

| 8 | 0.418 | -2.820 | -0.466 |
| --- | --- | --- | --- |
| 8 | 1.521 | 1.448 | 0.700 |
| 7 | 2.604 | -2.182 | -0.555 |
| 7 | 0.982 | -0.685 | 0.133 |
| 7 | -1.366 | 1.973 | 1.101 |
| 7 | -4.266 | 2.562 | 0.255 |
| 7 | -5.510 | 0.899 | -0.582 |
| 7 | -3.887 | -0.918 | -0.527 |
| 7 | -1.781 | -0.177 | 0.349 |
| 6 | 4.292 | 1.118 | 0.191 |
| 6 | 1.886 | 0.338 | 0.307 |
| 6 | 3.282 | 0.028 | 0.011 |
| 6 | 3.570 | -1.220 | -0.404 |
| 6 | 1.260 | -1.959 | -0.306 |
| 6 | -5.434 | 2.223 | -0.218 |
| 6 | -2.200 | 1.087 | 0.557 |
| 6 | -3.532 | 1.395 | 0.198 |
| 6 | -4.283 | 0.347 | -0.322 |
| 6 | -2.624 | -1.086 | -0.170 |
| 1 | 4.579 | -1.531 | -0.641 |
| 1 | 2.829 | -3.109 | -0.875 |
| 1 | -0.039 | -0.493 | 0.296 |
| 1 | -0.395 | 1.701 | 1.185 |
| 1 | -1.550 | 2.968 | 0.996 |
| 1 | -6.277 | 2.887 | -0.327 |
| 1 | -6.301 | 0.415 | -0.969 |
| 1 | -2.192 | -2.070 | -0.317 |
| 7 | -1.143 | 4.836 | 0.400 |
| 6 | 0.098 | 5.311 | 0.222 |
| 6 | 0.241 | 6.688 | -0.056 |
| 6 | -0.935 | 7.424 | -0.129 |
| 7 | -2.184 | 6.971 | 0.032 |
| 6 | -2.190 | 5.671 | 0.291 |
| 7 | -0.522 | 8.701 | -0.408 |
| 6 | 0.850 | 8.665 | -0.487 |
| 7 | 1.349 | 7.477 | -0.284 |
| 7 | 1.162 | 4.500 | 0.310 |
| 1 | -3.157 | 5.199 | 0.431 |
| 1 | 1.425 | 9.553 | -0.699 |
| 1 | 1.073 | 3.503 | 0.436 |
| 1 | 2.071 | 4.895 | 0.153 |
| 1 | -1.121 | 9.498 | -0.530 |
| 1 | 4.278 | 1.484 | 1.219 |
| 1 | 4.059 | 1.965 | -0.456 |
| 1 | 5.295 | 0.762 | -0.044 |

rA•A-T

| 8 | 0.126 | -3.247 | 0.000 |
| --- | --- | --- | --- |
| 8 | 2.004 | 0.903 | 0.000 |
| 7 | 2.398 | -3.072 | 0.000 |
| 7 | 1.091 | -1.172 | 0.000 |
| 7 | -0.748 | 2.068 | 0.000 |
| 7 | -3.746 | 2.852 | 0.000 |
| 7 | -5.280 | 1.230 | 0.000 |
| 7 | -3.838 | -0.729 | 0.000 |
| 7 | -1.523 | -0.109 | 0.000 |
| 6 | 4.716 | -0.078 | 0.000 |
| 6 | 2.172 | -0.315 | 0.000 |
| 6 | 3.492 | -0.951 | 0.000 |
| 6 | 3.531 | -2.298 | 0.000 |
| 6 | 1.120 | -2.545 | 0.000 |
| 6 | -5.022 | 2.575 | 0.000 |
| 6 | -1.768 | 1.218 | 0.000 |
| 6 | -3.128 | 1.616 | 0.000 |
| 6 | -4.066 | 0.591 | 0.000 |
| 6 | -2.539 | -0.985 | 0.000 |
| 1 | 4.465 | -2.845 | 0.000 |
| 1 | 2.447 | -4.077 | 0.000 |
| 1 | 0.132 | -0.750 | 0.000 |
| 1 | 0.196 | 1.696 | 0.000 |
| 1 | -0.889 | 3.073 | 0.000 |
| 1 | -5.817 | 3.304 | 0.000 |
| 1 | -6.177 | 0.778 | 0.000 |
| 1 | -2.235 | -2.027 | 0.000 |
| 7 | -2.478 | 8.058 | 0.000 |
| 6 | -2.269 | 6.732 | 0.000 |
| 6 | -0.935 | 6.269 | 0.000 |
| 6 | 0.052 | 7.249 | 0.000 |
| 7 | -0.125 | 8.573 | 0.000 |
| 6 | -1.419 | 8.876 | 0.000 |
| 7 | 1.235 | 6.550 | 0.000 |
| 6 | 0.913 | 5.219 | 0.000 |
| 7 | -0.376 | 5.008 | 0.000 |
| 7 | -3.325 | 5.909 | 0.000 |
| 1 | -1.656 | 9.935 | 0.000 |
| 1 | 1.666 | 4.446 | 0.000 |
| 1 | -4.231 | 6.341 | 0.000 |
| 1 | -3.238 | 4.899 | 0.000 |
| 1 | 2.155 | 6.956 | 0.000 |
| 1 | 4.398 | 0.963 | 0.000 |
| 1 | 5.331 | -0.250 | -0.884 |
| 1 | 5.331 | -0.250 | 0.884 |

G•G-C

| 7 | -2.685 | -0.079 | 0.000 |
| --- | --- | --- | --- |
| 8 | 1.525 | 1.726 | 0.000 |
| 7 | -0.605 | 0.910 | 0.000 |
| 6 | -1.972 | 1.052 | 0.000 |
| 7 | -2.587 | 2.224 | 0.000 |
| 6 | -1.724 | 3.251 | 0.000 |
| 6 | -0.335 | 3.223 | 0.000 |
| 6 | 0.307 | 1.955 | 0.000 |
| 7 | -2.037 | 4.583 | 0.000 |
| 6 | -0.849 | 5.280 | 0.000 |
| 7 | 0.192 | 4.497 | 0.000 |
| 1 | -2.259 | -1.008 | 0.000 |
| 1 | -3.683 | 0.024 | 0.000 |
| 1 | -0.202 | -0.045 | 0.000 |
| 1 | -2.968 | 4.961 | 0.000 |
| 1 | -0.825 | 6.358 | 0.000 |
| 8 | -1.522 | -2.699 | 0.000 |
| 7 | 2.681 | -0.919 | 0.000 |
| 7 | 0.568 | -1.770 | 0.000 |
| 6 | 2.455 | -3.305 | 0.000 |
| 6 | -0.294 | -2.811 | 0.000 |
| 7 | 0.245 | -4.100 | 0.000 |
| 6 | 1.584 | -4.334 | 0.000 |
| 6 | 1.892 | -1.976 | 0.000 |
| 1 | 3.523 | -3.459 | 0.000 |
| 1 | -0.427 | -4.848 | 0.000 |
| 1 | 1.893 | -5.370 | 0.000 |
| 1 | 2.271 | 0.012 | 0.000 |
| 1 | 3.689 | -0.953 | 0.000 |
| 6 | 8.590 | 2.975 | 0.000 |
| 7 | 7.904 | 4.173 | 0.000 |
| 1 | 8.285 | 5.102 | 0.000 |
| 6 | 6.573 | 3.866 | 0.000 |
| 6 | 6.531 | 2.477 | 0.000 |
| 7 | 7.802 | 1.940 | 0.000 |
| 6 | 5.247 | 1.842 | 0.000 |
| 8 | 4.976 | 0.647 | 0.000 |
| 7 | 4.217 | 2.801 | 0.000 |
| 1 | 3.267 | 2.430 | 0.000 |
| 6 | 4.382 | 4.160 | 0.000 |
| 7 | 5.558 | 4.754 | 0.000 |
| 7 | 3.265 | 4.911 | 0.000 |
| 1 | 9.668 | 2.943 | 0.000 |
| 1 | 2.319 | 4.550 | 0.000 |
| 1 | 3.407 | 5.904 | 0.000 |

rG•G-C

| 7 | -2.651 | 0.051 | 0.000 |
| --- | --- | --- | --- |
| 8 | 1.790 | 1.220 | 0.000 |
| 7 | -0.446 | 0.723 | 0.000 |
| 6 | -1.778 | 1.068 | 0.000 |
| 7 | -2.214 | 2.314 | 0.000 |
| 6 | -1.209 | 3.207 | 0.000 |
| 6 | 0.158 | 2.965 | 0.000 |
| 6 | 0.614 | 1.621 | 0.000 |
| 7 | -1.317 | 4.570 | 0.000 |
| 6 | -0.041 | 5.087 | 0.000 |
| 7 | 0.860 | 4.146 | 0.000 |
| 1 | -2.365 | -0.925 | 0.000 |
| 1 | -3.623 | 0.298 | 0.000 |
| 1 | -0.190 | -0.274 | 0.000 |
| 1 | -2.180 | 5.087 | 0.000 |
| 1 | 0.189 | 6.142 | 0.000 |
| 8 | -1.817 | -2.737 | 0.000 |
| 7 | 2.587 | -1.530 | 0.000 |
| 7 | 0.380 | -2.097 | 0.000 |
| 6 | 2.040 | -3.868 | 0.000 |
| 6 | -0.620 | -3.015 | 0.000 |
| 7 | -0.254 | -4.365 | 0.000 |
| 6 | 1.039 | -4.774 | 0.000 |
| 6 | 1.654 | -2.479 | 0.000 |
| 1 | 3.076 | -4.168 | 0.000 |
| 1 | -1.020 | -5.018 | 0.000 |
| 1 | 1.209 | -5.842 | 0.000 |
| 1 | 2.311 | -0.543 | 0.000 |
| 1 | 3.559 | -1.777 | 0.000 |
| 6 | 6.634 | 8.179 | 0.000 |
| 7 | 7.032 | 6.858 | 0.000 |
| 1 | 7.968 | 6.496 | 0.000 |
| 6 | 5.894 | 6.102 | 0.000 |
| 6 | 4.855 | 7.027 | 0.000 |
| 7 | 5.341 | 8.317 | 0.000 |
| 6 | 3.507 | 6.526 | 0.000 |
| 8 | 2.442 | 7.129 | 0.000 |
| 7 | 3.512 | 5.120 | 0.000 |
| 1 | 2.575 | 4.693 | 0.000 |
| 6 | 4.606 | 4.310 | 0.000 |
| 7 | 5.848 | 4.750 | 0.000 |
| 7 | 4.368 | 2.979 | 0.000 |
| 1 | 7.352 | 8.984 | 0.000 |
| 1 | 3.440 | 2.577 | 0.000 |
| 1 | 5.173 | 2.384 | 0.000 |

C^+^•G-C/C^+^•G-C

| 7 | -1.359 | 1.486 | -2.756 |
| --- | --- | --- | --- |
| 8 | 1.195 | -1.709 | -0.658 |
| 7 | -0.028 | -0.060 | -1.680 |
| 6 | -0.160 | 1.182 | -2.273 |
| 7 | 0.839 | 2.053 | -2.377 |
| 6 | 1.978 | 1.590 | -1.862 |
| 6 | 2.217 | 0.357 | -1.266 |
| 6 | 1.143 | -0.561 | -1.157 |
| 7 | 3.185 | 2.238 | -1.813 |
| 6 | 4.083 | 1.403 | -1.215 |
| 7 | 3.537 | 0.264 | -0.869 |
| 1 | -2.172 | 0.867 | -2.665 |
| 1 | -1.461 | 2.392 | -3.179 |
| 1 | -0.871 | -0.661 | -1.580 |
| 1 | 3.363 | 3.175 | -2.138 |
| 1 | 5.116 | 1.668 | -1.055 |
| 8 | -3.611 | -0.103 | -2.342 |
| 7 | -1.250 | -3.341 | -0.158 |
| 7 | -2.390 | -1.720 | -1.287 |
| 6 | -3.584 | -3.711 | -0.590 |
| 6 | -3.537 | -1.215 | -1.815 |
| 7 | -4.680 | -1.999 | -1.763 |
| 6 | -4.709 | -3.201 | -1.145 |
| 6 | -2.401 | -2.907 | -0.688 |
| 1 | -3.579 | -4.675 | -0.105 |
| 1 | -5.526 | -1.530 | -2.058 |
| 1 | -5.660 | -3.716 | -1.131 |
| 1 | -0.407 | -2.792 | -0.291 |
| 1 | -1.196 | -4.262 | 0.235 |
| 6 | 5.749 | -4.078 | -0.777 |
| 6 | 7.006 | -3.705 | -0.440 |
| 7 | 7.285 | -2.442 | -0.025 |
| 6 | 6.329 | -1.445 | 0.059 |
| 8 | 6.586 | -0.310 | 0.404 |
| 7 | 5.051 | -1.847 | -0.280 |
| 1 | 4.360 | -1.014 | -0.406 |
| 6 | 4.720 | -3.088 | -0.692 |
| 7 | 3.462 | -3.339 | -0.993 |
| 1 | 5.529 | -5.083 | -1.101 |
| 1 | 7.843 | -4.389 | -0.481 |
| 1 | 2.702 | -2.651 | -0.871 |
| 1 | 3.219 | -4.250 | -1.345 |
| 1 | 8.228 | -2.162 | 0.204 |
| 7 | -4.697 | 2.409 | -0.901 |
| 8 | -1.214 | 0.396 | 1.307 |
| 7 | -2.898 | 1.471 | 0.198 |
| 6 | -3.436 | 2.536 | -0.495 |
| 7 | -2.766 | 3.654 | -0.742 |
| 6 | -1.527 | 3.629 | -0.250 |
| 6 | -0.890 | 2.604 | 0.441 |
| 6 | -1.618 | 1.416 | 0.704 |
| 7 | -0.587 | 4.620 | -0.345 |
| 6 | 0.552 | 4.175 | 0.262 |
| 7 | 0.405 | 2.970 | 0.748 |
| 1 | -5.191 | 1.519 | -0.885 |
| 1 | -5.077 | 3.176 | -1.427 |
| 1 | -3.503 | 0.656 | 0.380 |
| 1 | -0.728 | 5.517 | -0.783 |
| 1 | 1.458 | 4.756 | 0.331 |
| 8 | -6.332 | 0.023 | -0.458 |
| 7 | -3.229 | -1.364 | 2.592 |
| 7 | -4.757 | -0.641 | 1.059 |
| 6 | -5.537 | -1.907 | 2.977 |
| 6 | -6.030 | -0.540 | 0.596 |
| 7 | -7.042 | -1.127 | 1.353 |
| 6 | -6.802 | -1.783 | 2.515 |
| 6 | -4.503 | -1.289 | 2.194 |
| 1 | -5.321 | -2.425 | 3.898 |
| 1 | -7.979 | -0.995 | 1.004 |
| 1 | -7.662 | -2.188 | 3.031 |
| 1 | -2.516 | -0.842 | 2.088 |
| 1 | -3.013 | -1.744 | 3.495 |
| 6 | 3.708 | -0.131 | 2.698 |
| 6 | 4.833 | 0.617 | 2.600 |
| 7 | 4.803 | 1.871 | 2.089 |
| 6 | 3.645 | 2.474 | 1.621 |
| 8 | 3.642 | 3.570 | 1.103 |
| 7 | 2.504 | 1.715 | 1.780 |
| 1 | 1.599 | 2.194 | 1.392 |
| 6 | 2.484 | 0.452 | 2.242 |
| 7 | 1.339 | -0.200 | 2.262 |
| 1 | 3.726 | -1.125 | 3.115 |
| 1 | 5.802 | 0.258 | 2.913 |
| 1 | 0.451 | 0.179 | 1.896 |
| 1 | 1.338 | -1.159 | 2.561 |
| 1 | 5.649 | 2.413 | 1.990 |

C^+^•G-C/T•A-T

| 8 | -3.905 | -1.724 | 2.222 |
| --- | --- | --- | --- |
| 8 | -2.239 | 2.355 | 1.085 |
| 7 | -5.289 | 0.089 | 2.322 |
| 7 | -3.087 | 0.341 | 1.695 |
| 7 | 0.505 | 1.173 | 1.011 |
| 7 | 3.166 | -0.381 | 1.397 |
| 7 | 3.100 | -2.516 | 2.007 |
| 7 | 0.691 | -2.840 | 2.157 |
| 7 | -0.492 | -0.817 | 1.641 |
| 6 | -4.699 | 3.742 | 1.528 |
| 6 | -3.205 | 1.694 | 1.469 |
| 6 | -4.525 | 2.266 | 1.717 |
| 6 | -5.501 | 1.428 | 2.123 |
| 6 | -4.078 | -0.525 | 2.088 |
| 6 | 3.878 | -1.438 | 1.697 |
| 6 | 0.617 | -0.103 | 1.382 |
| 6 | 1.852 | -0.781 | 1.528 |
| 6 | 1.789 | -2.116 | 1.913 |
| 6 | -0.403 | -2.108 | 2.003 |
| 1 | -6.506 | 1.773 | 2.325 |
| 1 | -6.049 | -0.530 | 2.550 |
| 1 | -2.145 | -0.081 | 1.594 |
| 1 | -0.419 | 1.600 | 0.985 |
| 1 | 1.323 | 1.735 | 0.823 |
| 1 | 4.957 | -1.457 | 1.715 |
| 1 | 3.414 | -3.437 | 2.264 |
| 1 | -1.358 | -2.591 | 2.188 |
| 1 | -4.074 | 4.293 | 2.233 |
| 1 | -5.738 | 4.035 | 1.679 |
| 1 | -4.385 | 4.037 | 0.526 |
| 6 | 4.070 | 5.439 | -0.614 |
| 6 | 4.741 | 4.208 | -0.086 |
| 6 | 6.055 | 4.119 | 0.206 |
| 7 | 6.634 | 2.962 | 0.670 |
| 1 | 7.626 | 2.907 | 0.842 |
| 6 | 5.924 | 1.802 | 0.895 |
| 8 | 6.446 | 0.748 | 1.218 |
| 7 | 4.573 | 1.941 | 0.691 |
| 1 | 4.001 | 1.098 | 0.913 |
| 6 | 3.931 | 3.014 | 0.118 |
| 8 | 2.741 | 2.921 | -0.205 |
| 1 | 6.730 | 4.956 | 0.079 |
| 1 | 4.790 | 6.245 | -0.750 |
| 1 | 3.293 | 5.774 | 0.074 |
| 1 | 3.587 | 5.230 | -1.569 |
| 7 | -4.682 | -2.538 | -0.713 |
| 8 | -0.714 | -0.380 | -1.544 |
| 7 | -2.645 | -1.519 | -1.093 |
| 6 | -3.352 | -2.656 | -0.763 |
| 7 | -2.784 | -3.830 | -0.543 |
| 6 | -1.457 | -3.794 | -0.699 |
| 6 | -0.660 | -2.718 | -1.063 |
| 6 | -1.277 | -1.457 | -1.261 |
| 7 | -0.580 | -4.832 | -0.522 |
| 6 | 0.678 | -4.362 | -0.767 |
| 7 | 0.665 | -3.097 | -1.100 |
| 1 | -5.135 | -1.626 | -0.698 |
| 1 | -5.163 | -3.323 | -0.310 |
| 1 | -3.157 | -0.629 | -1.209 |
| 1 | -0.832 | -5.762 | -0.231 |
| 1 | 1.568 | -4.965 | -0.697 |
| 8 | -5.971 | 0.055 | -0.717 |
| 7 | -2.160 | 2.075 | -2.207 |
| 7 | -4.047 | 1.030 | -1.473 |
| 6 | -4.220 | 3.302 | -2.313 |
| 6 | -5.378 | 1.012 | -1.215 |
| 7 | -6.114 | 2.154 | -1.535 |
| 6 | -5.549 | 3.262 | -2.075 |
| 6 | -3.470 | 2.117 | -1.982 |
| 1 | -3.747 | 4.172 | -2.739 |
| 1 | -7.104 | 2.095 | -1.358 |
| 1 | -6.213 | 4.088 | -2.292 |
| 1 | -1.630 | 1.254 | -1.919 |
| 1 | -1.676 | 2.899 | -2.512 |
| 6 | 4.427 | 0.047 | -1.745 |
| 6 | 5.462 | -0.808 | -1.595 |
| 7 | 5.253 | -2.132 | -1.368 |
| 6 | 3.989 | -2.700 | -1.292 |
| 8 | 3.823 | -3.880 | -1.064 |
| 7 | 2.949 | -1.812 | -1.478 |
| 1 | 1.962 | -2.279 | -1.351 |
| 6 | 3.100 | -0.480 | -1.621 |
| 7 | 2.039 | 0.296 | -1.651 |
| 1 | 4.580 | 1.096 | -1.934 |
| 1 | 6.493 | -0.488 | -1.636 |
| 1 | 1.081 | -0.070 | -1.565 |
| 1 | 2.173 | 1.294 | -1.542 |
| 1 | 6.021 | -2.774 | -1.250 |

T•A-T/T•A-T

| 8 | 4.232 | -2.291 | -1.886 |
| --- | --- | --- | --- |
| 8 | 3.002 | 2.064 | -1.337 |
| 7 | 5.841 | -0.679 | -1.948 |
| 7 | 3.629 | -0.100 | -1.629 |
| 7 | 0.071 | 1.199 | -1.321 |
| 7 | -2.685 | -0.181 | -1.634 |
| 7 | -2.775 | -2.382 | -1.937 |
| 7 | -0.394 | -2.898 | -1.983 |
| 7 | 0.932 | -0.922 | -1.673 |
| 6 | 5.648 | 3.082 | -1.606 |
| 6 | 3.900 | 1.247 | -1.532 |
| 6 | 5.307 | 1.625 | -1.666 |
| 6 | 6.205 | 0.637 | -1.846 |
| 6 | 4.535 | -1.114 | -1.819 |
| 6 | -3.469 | -1.217 | -1.784 |
| 6 | -0.126 | -0.096 | -1.548 |
| 6 | -1.405 | -0.692 | -1.676 |
| 6 | -1.440 | -2.068 | -1.869 |
| 6 | 0.750 | -2.236 | -1.878 |
| 1 | 7.266 | 0.834 | -1.940 |
| 1 | 6.529 | -1.412 | -1.969 |
| 1 | 2.627 | -0.392 | -1.576 |
| 1 | 1.021 | 1.542 | -1.221 |
| 1 | -0.707 | 1.830 | -1.178 |
| 1 | -4.547 | -1.162 | -1.790 |
| 1 | -3.168 | -3.309 | -1.880 |
| 1 | 1.668 | -2.811 | -1.956 |
| 1 | 5.213 | 3.612 | -2.455 |
| 1 | 6.729 | 3.231 | -1.617 |
| 1 | 5.229 | 3.531 | -0.705 |
| 6 | -3.344 | 5.754 | -0.067 |
| 6 | -4.094 | 4.504 | -0.402 |
| 6 | -5.434 | 4.387 | -0.390 |
| 7 | -6.073 | 3.209 | -0.705 |
| 1 | -7.076 | 3.144 | -0.714 |
| 6 | -5.404 | 2.053 | -1.056 |
| 8 | -5.998 | 1.017 | -1.321 |
| 7 | -4.043 | 2.184 | -1.069 |
| 1 | -3.492 | 1.330 | -1.311 |
| 6 | -3.312 | 3.324 | -0.779 |
| 8 | -2.090 | 3.319 | -0.839 |
| 1 | -6.084 | 5.213 | -0.130 |
| 1 | -4.026 | 6.562 | 0.195 |
| 1 | -2.731 | 6.065 | -0.914 |
| 1 | -2.664 | 5.574 | 0.767 |
| 8 | 5.792 | -0.662 | 1.134 |
| 8 | 2.047 | 1.803 | 1.967 |
| 7 | 6.013 | 1.535 | 1.691 |
| 7 | 3.918 | 0.572 | 1.588 |
| 7 | 0.356 | -0.624 | 1.805 |
| 7 | -1.020 | -3.319 | 1.177 |
| 7 | 0.218 | -5.053 | 0.550 |
| 7 | 2.434 | -4.057 | 0.665 |
| 7 | 2.322 | -1.746 | 1.301 |
| 6 | 3.442 | 4.250 | 2.266 |
| 6 | 3.266 | 1.760 | 1.861 |
| 6 | 4.119 | 2.938 | 2.029 |
| 6 | 5.450 | 2.764 | 1.951 |
| 6 | 5.270 | 0.396 | 1.446 |
| 6 | -1.036 | -4.560 | 0.769 |
| 6 | 0.980 | -1.743 | 1.453 |
| 6 | 0.313 | -2.967 | 1.203 |
| 6 | 1.104 | -4.036 | 0.805 |
| 6 | 2.958 | -2.869 | 0.929 |
| 1 | 6.150 | 3.579 | 2.078 |
| 1 | 7.007 | 1.424 | 1.590 |
| 1 | 3.324 | -0.282 | 1.463 |
| 1 | 0.892 | 0.235 | 1.886 |
| 1 | -0.648 | -0.598 | 1.931 |
| 1 | -1.934 | -5.137 | 0.611 |
| 1 | 0.461 | -5.954 | 0.176 |
| 1 | 4.032 | -2.770 | 0.826 |
| 1 | 2.800 | 4.491 | 1.417 |
| 1 | 4.171 | 5.049 | 2.402 |
| 1 | 2.801 | 4.194 | 3.147 |
| 6 | -4.991 | 1.323 | 2.263 |
| 6 | -4.864 | -0.066 | 1.718 |
| 6 | -5.864 | -0.746 | 1.129 |
| 7 | -5.693 | -2.021 | 0.639 |
| 1 | -6.444 | -2.505 | 0.176 |
| 6 | -4.490 | -2.692 | 0.677 |
| 8 | -4.350 | -3.802 | 0.174 |
| 7 | -3.488 | -2.001 | 1.295 |
| 1 | -2.551 | -2.463 | 1.307 |
| 6 | -3.556 | -0.711 | 1.795 |
| 8 | -2.564 | -0.168 | 2.264 |
| 1 | -6.847 | -0.322 | 0.984 |
| 1 | -5.995 | 1.714 | 2.098 |
| 1 | -4.266 | 1.984 | 1.785 |
| 1 | -4.775 | 1.335 | 3.332 |

rC^+^•G-C/rC^+^•G-C

| 7 | -1.188 | -2.344 | 1.842 |
| --- | --- | --- | --- |
| 8 | 2.395 | 0.373 | 0.973 |
| 7 | 0.665 | -1.041 | 1.420 |
| 6 | 0.123 | -2.291 | 1.633 |
| 7 | 0.837 | -3.413 | 1.605 |
| 6 | 2.133 | -3.190 | 1.370 |
| 6 | 2.773 | -1.982 | 1.120 |
| 6 | 1.992 | -0.797 | 1.150 |
| 7 | 3.132 | -4.126 | 1.318 |
| 6 | 4.304 | -3.463 | 1.064 |
| 7 | 4.129 | -2.174 | 0.931 |
| 1 | -1.764 | -1.498 | 1.941 |
| 1 | -1.570 | -3.243 | 2.078 |
| 1 | 0.055 | -0.200 | 1.509 |
| 1 | 3.023 | -5.107 | 1.521 |
| 1 | 5.249 | -3.978 | 0.999 |
| 8 | -2.762 | -0.053 | 2.069 |
| 7 | 0.776 | 2.800 | 1.598 |
| 7 | -0.963 | 1.340 | 1.840 |
| 6 | -1.408 | 3.725 | 1.945 |
| 6 | -2.285 | 1.086 | 2.000 |
| 7 | -3.154 | 2.167 | 2.065 |
| 6 | -2.729 | 3.452 | 2.050 |
| 6 | -0.529 | 2.601 | 1.796 |
| 1 | -1.037 | 4.738 | 1.939 |
| 1 | -4.138 | 1.943 | 2.093 |
| 1 | -3.492 | 4.215 | 2.111 |
| 1 | 1.364 | 2.008 | 1.366 |
| 1 | 1.120 | 3.725 | 1.414 |
| 6 | 6.920 | 2.013 | -0.014 |
| 6 | 6.566 | 3.200 | -0.556 |
| 7 | 5.265 | 3.506 | -0.797 |
| 6 | 4.214 | 2.659 | -0.494 |
| 8 | 3.054 | 2.960 | -0.685 |
| 7 | 4.596 | 1.446 | 0.051 |
| 1 | 3.773 | 0.857 | 0.384 |
| 6 | 5.876 | 1.084 | 0.292 |
| 7 | 6.117 | -0.108 | 0.792 |
| 1 | 7.953 | 1.764 | 0.172 |
| 1 | 7.293 | 3.955 | -0.825 |
| 1 | 5.377 | -0.827 | 0.902 |
| 1 | 7.069 | -0.362 | 0.997 |
| 1 | 5.003 | 4.393 | -1.202 |
| 7 | -5.388 | 1.760 | -0.206 |
| 8 | -1.706 | -0.853 | -0.995 |
| 7 | -3.611 | 0.364 | -0.711 |
| 6 | -4.928 | 0.507 | -0.336 |
| 7 | -5.728 | -0.509 | -0.071 |
| 6 | -5.105 | -1.685 | -0.154 |
| 6 | -3.773 | -1.941 | -0.456 |
| 6 | -2.931 | -0.834 | -0.736 |
| 7 | -5.656 | -2.917 | 0.071 |
| 6 | -4.668 | -3.845 | -0.094 |
| 7 | -3.522 | -3.298 | -0.406 |
| 1 | -4.849 | 2.567 | -0.534 |
| 1 | -6.386 | 1.842 | -0.106 |
| 1 | -3.041 | 1.210 | -0.895 |
| 1 | -6.618 | -3.089 | 0.317 |
| 1 | -4.850 | -4.901 | 0.024 |
| 8 | -3.834 | 3.995 | -0.843 |
| 7 | 0.022 | 1.603 | -1.371 |
| 7 | -1.939 | 2.752 | -1.152 |
| 6 | 0.109 | 3.986 | -1.590 |
| 6 | -2.633 | 3.916 | -1.118 |
| 7 | -1.949 | 5.096 | -1.388 |
| 6 | -0.611 | 5.128 | -1.612 |
| 6 | -0.621 | 2.763 | -1.369 |
| 1 | 1.175 | 3.974 | -1.748 |
| 1 | -2.513 | 5.932 | -1.402 |
| 1 | -0.177 | 6.100 | -1.803 |
| 1 | -0.492 | 0.737 | -1.246 |
| 1 | 1.023 | 1.561 | -1.461 |
| 6 | 1.074 | -4.912 | -1.790 |
| 6 | 2.223 | -4.300 | -2.147 |
| 7 | 2.327 | -2.946 | -2.146 |
| 6 | 1.286 | -2.097 | -1.803 |
| 8 | 1.416 | -0.893 | -1.796 |
| 7 | 0.106 | -2.742 | -1.471 |
| 1 | -0.698 | -2.087 | -1.246 |
| 6 | -0.044 | -4.087 | -1.430 |
| 7 | -1.196 | -4.602 | -1.081 |
| 1 | 0.981 | -5.986 | -1.794 |
| 1 | 3.104 | -4.848 | -2.451 |
| 1 | -2.053 | -4.040 | -0.817 |
| 1 | -1.277 | -5.605 | -1.075 |
| 1 | 3.186 | -2.482 | -2.401 |

rT•A-T/rT•A-T

| 8 | -4.591 | -3.279 | 0.838 |
| --- | --- | --- | --- |
| 8 | -3.208 | 0.920 | 1.937 |
| 7 | -6.159 | -1.702 | 1.338 |
| 7 | -3.912 | -1.178 | 1.428 |
| 7 | -0.358 | 0.011 | 1.685 |
| 7 | 2.392 | -1.372 | 1.385 |
| 7 | 2.484 | -3.565 | 1.069 |
| 7 | 0.101 | -4.093 | 1.081 |
| 7 | -1.225 | -2.120 | 1.413 |
| 6 | -5.836 | 1.949 | 2.269 |
| 6 | -4.140 | 0.139 | 1.762 |
| 6 | -5.543 | 0.531 | 1.893 |
| 6 | -6.479 | -0.409 | 1.657 |
| 6 | -4.858 | -2.139 | 1.168 |
| 6 | 3.182 | -2.397 | 1.201 |
| 6 | -0.164 | -1.290 | 1.495 |
| 6 | 1.114 | -1.887 | 1.372 |
| 6 | 1.146 | -3.259 | 1.174 |
| 6 | -1.044 | -3.433 | 1.210 |
| 1 | -7.538 | -0.194 | 1.723 |
| 1 | -6.864 | -2.362 | 1.060 |
| 1 | -2.922 | -1.497 | 1.375 |
| 1 | -1.308 | 0.368 | 1.729 |
| 1 | 0.412 | 0.668 | 1.644 |
| 1 | 4.257 | -2.336 | 1.137 |
| 1 | 2.869 | -4.484 | 0.936 |
| 1 | -1.960 | -4.007 | 1.126 |
| 1 | -5.368 | 2.635 | 1.562 |
| 1 | -5.423 | 2.178 | 3.253 |
| 1 | -6.911 | 2.134 | 2.287 |
| 6 | 7.264 | 2.188 | 1.822 |
| 6 | 5.772 | 2.250 | 1.709 |
| 6 | 5.052 | 3.388 | 1.681 |
| 7 | 3.680 | 3.389 | 1.586 |
| 1 | 3.150 | 4.244 | 1.601 |
| 6 | 2.924 | 2.232 | 1.563 |
| 8 | 1.705 | 2.256 | 1.555 |
| 7 | 3.668 | 1.085 | 1.556 |
| 1 | 3.130 | 0.188 | 1.514 |
| 6 | 5.046 | 0.979 | 1.632 |
| 8 | 5.592 | -0.117 | 1.627 |
| 1 | 5.515 | 4.365 | 1.736 |
| 1 | 7.700 | 3.187 | 1.834 |
| 1 | 7.682 | 1.622 | 0.988 |
| 1 | 7.553 | 1.663 | 2.734 |
| 8 | -5.722 | -0.371 | -1.394 |
| 8 | -2.583 | 2.933 | -1.174 |
| 7 | -6.402 | 1.801 | -1.306 |
| 7 | -4.152 | 1.291 | -1.266 |
| 7 | -0.386 | 0.920 | -1.527 |
| 7 | 1.440 | -1.548 | -1.889 |
| 7 | 0.528 | -3.569 | -2.043 |
| 7 | -1.825 | -2.988 | -1.828 |
| 7 | -2.117 | -0.624 | -1.533 |
| 6 | -4.473 | 5.046 | -1.072 |
| 6 | -3.767 | 2.617 | -1.194 |
| 6 | -4.852 | 3.601 | -1.163 |
| 6 | -6.114 | 3.143 | -1.230 |
| 6 | -5.433 | 0.813 | -1.327 |
| 6 | 1.676 | -2.828 | -2.015 |
| 6 | -0.801 | -0.339 | -1.621 |
| 6 | 0.068 | -1.442 | -1.809 |
| 6 | -0.521 | -2.696 | -1.895 |
| 6 | -2.546 | -1.894 | -1.638 |
| 1 | -6.971 | 3.804 | -1.224 |
| 1 | -7.349 | 1.469 | -1.369 |
| 1 | -3.383 | 0.578 | -1.321 |
| 1 | -1.069 | 1.649 | -1.350 |
| 1 | 0.599 | 1.156 | -1.558 |
| 1 | 2.665 | -3.255 | -2.078 |
| 1 | 0.447 | -4.571 | -2.028 |
| 1 | -3.619 | -2.022 | -1.553 |
| 1 | -3.838 | 5.324 | -1.915 |
| 1 | -3.894 | 5.228 | -0.165 |
| 1 | -5.357 | 5.684 | -1.065 |
| 6 | 7.410 | -0.586 | -1.210 |
| 6 | 6.118 | 0.134 | -1.425 |
| 6 | 5.996 | 1.469 | -1.532 |
| 7 | 4.771 | 2.089 | -1.652 |
| 1 | 4.690 | 3.090 | -1.709 |
| 6 | 3.577 | 1.402 | -1.652 |
| 8 | 2.499 | 1.979 | -1.712 |
| 7 | 3.714 | 0.046 | -1.579 |
| 1 | 2.831 | -0.503 | -1.639 |
| 6 | 4.893 | -0.671 | -1.465 |
| 8 | 4.876 | -1.894 | -1.412 |
| 1 | 6.852 | 2.131 | -1.515 |
| 1 | 8.259 | 0.091 | -1.310 |
| 1 | 7.515 | -1.403 | -1.924 |
| 1 | 7.413 | -1.016 | -0.206 |

G•G-C/G•G-C

| 7 | 3.555 | 3.116 | 1.065 |
| --- | --- | --- | --- |
| 8 | 0.088 | 0.524 | 2.572 |
| 7 | 1.747 | 1.878 | 1.776 |
| 6 | 2.251 | 3.091 | 1.371 |
| 7 | 1.530 | 4.197 | 1.299 |
| 6 | 0.249 | 3.991 | 1.652 |
| 6 | -0.361 | 2.820 | 2.084 |
| 6 | 0.439 | 1.648 | 2.177 |
| 7 | -0.749 | 4.924 | 1.683 |
| 6 | -1.888 | 4.284 | 2.131 |
| 7 | -1.693 | 3.024 | 2.383 |
| 1 | 4.132 | 2.274 | 1.005 |
| 1 | 3.895 | 3.971 | 0.666 |
| 1 | 2.378 | 1.058 | 1.752 |
| 1 | -0.644 | 5.896 | 1.452 |
| 1 | -2.824 | 4.808 | 2.241 |
| 8 | 5.261 | 0.818 | 0.838 |
| 7 | 1.717 | -1.792 | 2.054 |
| 7 | 3.474 | -0.454 | 1.484 |
| 6 | 3.768 | -2.868 | 1.454 |
| 6 | 4.744 | -0.282 | 1.055 |
| 7 | 5.514 | -1.425 | 0.838 |
| 6 | 5.029 | -2.680 | 1.013 |
| 6 | 2.976 | -1.683 | 1.665 |
| 1 | 3.347 | -3.850 | 1.604 |
| 1 | 6.430 | -1.262 | 0.458 |
| 1 | 5.703 | -3.492 | 0.781 |
| 1 | 1.172 | -0.941 | 2.168 |
| 1 | 1.223 | -2.679 | 2.099 |
| 6 | -3.690 | -4.341 | -0.599 |
| 7 | -4.370 | -3.242 | -0.142 |
| 1 | -5.110 | -2.730 | -0.622 |
| 6 | -3.585 | -2.672 | 0.812 |
| 6 | -2.485 | -3.516 | 0.933 |
| 7 | -2.563 | -4.556 | 0.026 |
| 6 | -1.417 | -3.125 | 1.793 |
| 8 | -0.353 | -3.708 | 2.015 |
| 7 | -1.687 | -1.883 | 2.385 |
| 1 | -0.952 | -1.484 | 2.951 |
| 6 | -2.805 | -1.109 | 2.170 |
| 7 | -3.811 | -1.489 | 1.421 |
| 7 | -2.834 | 0.080 | 2.818 |
| 1 | -4.080 | -4.953 | -1.397 |
| 1 | -1.940 | 0.529 | 2.977 |
| 1 | -3.544 | 0.706 | 2.474 |
| 7 | 4.473 | 0.269 | -2.217 |
| 8 | 0.033 | 0.653 | -1.177 |
| 7 | 2.252 | 0.556 | -1.681 |
| 6 | 3.458 | 1.118 | -2.024 |
| 7 | 3.638 | 2.419 | -2.191 |
| 6 | 2.504 | 3.114 | -2.020 |
| 6 | 1.233 | 2.651 | -1.707 |
| 6 | 1.069 | 1.253 | -1.496 |
| 7 | 2.355 | 4.469 | -2.135 |
| 6 | 1.030 | 4.754 | -1.902 |
| 7 | 0.326 | 3.690 | -1.646 |
| 1 | 4.386 | -0.739 | -2.073 |
| 1 | 5.377 | 0.688 | -2.329 |
| 1 | 2.197 | -0.471 | -1.559 |
| 1 | 3.086 | 5.117 | -2.374 |
| 1 | 0.653 | 5.763 | -1.938 |
| 8 | 4.303 | -2.577 | -1.878 |
| 7 | -0.187 | -2.260 | -1.053 |
| 7 | 2.068 | -2.360 | -1.429 |
| 6 | 0.833 | -4.413 | -0.955 |
| 6 | 3.198 | -3.074 | -1.616 |
| 7 | 3.120 | -4.459 | -1.507 |
| 6 | 1.968 | -5.107 | -1.161 |
| 6 | 0.912 | -2.983 | -1.148 |
| 1 | -0.096 | -4.873 | -0.648 |
| 1 | 3.974 | -4.956 | -1.691 |
| 1 | 2.030 | -6.182 | -1.063 |
| 1 | -0.168 | -1.253 | -1.181 |
| 1 | -1.090 | -2.690 | -0.927 |
| 6 | -6.930 | -0.301 | -1.209 |
| 7 | -6.712 | 1.049 | -1.088 |
| 1 | -7.400 | 1.771 | -0.966 |
| 6 | -5.359 | 1.246 | -1.143 |
| 6 | -4.825 | -0.021 | -1.312 |
| 7 | -5.823 | -0.975 | -1.347 |
| 6 | -3.396 | -0.153 | -1.372 |
| 8 | -2.721 | -1.162 | -1.525 |
| 7 | -2.776 | 1.099 | -1.227 |
| 1 | -1.753 | 1.068 | -1.216 |
| 6 | -3.415 | 2.300 | -1.093 |
| 7 | -4.727 | 2.433 | -1.045 |
| 7 | -2.638 | 3.396 | -1.000 |
| 1 | -7.922 | -0.723 | -1.185 |
| 1 | -1.639 | 3.391 | -1.189 |
| 1 | -3.131 | 4.269 | -0.994 |

rG•G-C/rG•G-C

| 7 | -2.580 | -3.146 | 1.543 |
| --- | --- | --- | --- |
| 8 | -0.776 | 1.063 | 1.876 |
| 7 | -1.591 | -1.070 | 1.716 |
| 6 | -1.439 | -2.433 | 1.614 |
| 7 | -0.274 | -3.045 | 1.605 |
| 6 | 0.759 | -2.189 | 1.752 |
| 6 | 0.706 | -0.811 | 1.929 |
| 6 | -0.551 | -0.163 | 1.848 |
| 7 | 2.088 | -2.496 | 1.787 |
| 6 | 2.769 | -1.317 | 1.973 |
| 7 | 1.971 | -0.289 | 2.076 |
| 1 | -3.484 | -2.705 | 1.394 |
| 1 | -2.471 | -4.119 | 1.327 |
| 1 | -2.536 | -0.668 | 1.657 |
| 1 | 2.510 | -3.352 | 1.443 |
| 1 | 3.848 | -1.266 | 2.024 |
| 8 | -5.220 | -1.879 | 1.212 |
| 7 | -3.361 | 2.222 | 1.971 |
| 7 | -4.257 | 0.157 | 1.605 |
| 6 | -5.755 | 2.038 | 1.918 |
| 6 | -5.315 | -0.675 | 1.445 |
| 7 | -6.593 | -0.125 | 1.572 |
| 6 | -6.802 | 1.195 | 1.772 |
| 6 | -4.440 | 1.454 | 1.831 |
| 1 | -5.895 | 3.094 | 2.096 |
| 1 | -7.354 | -0.761 | 1.398 |
| 1 | -7.832 | 1.520 | 1.819 |
| 1 | -2.423 | 1.805 | 1.924 |
| 1 | -3.462 | 3.194 | 2.196 |
| 6 | 6.673 | 3.506 | -1.078 |
| 7 | 5.411 | 3.989 | -1.327 |
| 1 | 5.123 | 4.577 | -2.089 |
| 6 | 4.580 | 3.444 | -0.388 |
| 6 | 5.410 | 2.685 | 0.420 |
| 7 | 6.709 | 2.715 | -0.040 |
| 6 | 4.800 | 1.834 | 1.404 |
| 8 | 5.309 | 0.975 | 2.108 |
| 7 | 3.414 | 2.071 | 1.468 |
| 1 | 2.893 | 1.373 | 2.010 |
| 6 | 2.697 | 2.836 | 0.601 |
| 7 | 3.232 | 3.567 | -0.359 |
| 7 | 1.354 | 2.848 | 0.773 |
| 1 | 7.517 | 3.776 | -1.691 |
| 1 | 0.865 | 2.131 | 1.299 |
| 1 | 0.847 | 3.298 | 0.034 |
| 7 | -4.897 | -1.979 | -1.846 |
| 8 | -0.818 | 0.098 | -1.444 |
| 7 | -2.810 | -1.021 | -1.595 |
| 6 | -3.571 | -2.162 | -1.695 |
| 7 | -3.067 | -3.380 | -1.691 |
| 6 | -1.724 | -3.377 | -1.608 |
| 6 | -0.864 | -2.288 | -1.568 |
| 6 | -1.422 | -0.983 | -1.535 |
| 7 | -0.902 | -4.467 | -1.532 |
| 6 | 0.390 | -4.003 | -1.445 |
| 7 | 0.442 | -2.703 | -1.476 |
| 1 | -5.313 | -1.080 | -1.620 |
| 1 | -5.448 | -2.798 | -1.659 |
| 1 | -3.284 | -0.111 | -1.523 |
| 1 | -1.200 | -5.427 | -1.537 |
| 1 | 1.254 | -4.642 | -1.343 |
| 8 | -6.138 | 0.685 | -1.347 |
| 7 | -2.023 | 2.666 | -1.318 |
| 7 | -4.061 | 1.644 | -1.365 |
| 6 | -3.969 | 4.069 | -1.405 |
| 6 | -5.414 | 1.680 | -1.391 |
| 7 | -6.024 | 2.937 | -1.478 |
| 6 | -5.317 | 4.095 | -1.478 |
| 6 | -3.348 | 2.767 | -1.363 |
| 1 | -3.385 | 4.976 | -1.406 |
| 1 | -7.029 | 2.927 | -1.533 |
| 1 | -5.887 | 5.011 | -1.540 |
| 1 | -1.579 | 1.741 | -1.308 |
| 1 | -1.457 | 3.492 | -1.386 |
| 6 | 7.169 | -1.517 | 0.169 |
| 7 | 6.647 | -0.346 | -0.327 |
| 1 | 7.018 | 0.593 | -0.218 |
| 6 | 5.371 | -0.625 | -0.708 |
| 6 | 5.203 | -1.986 | -0.453 |
| 7 | 6.346 | -2.525 | 0.100 |
| 6 | 3.912 | -2.555 | -0.684 |
| 8 | 3.500 | -3.699 | -0.459 |
| 7 | 3.023 | -1.594 | -1.181 |
| 1 | 2.063 | -1.935 | -1.332 |
| 6 | 3.291 | -0.271 | -1.373 |
| 7 | 4.471 | 0.268 | -1.177 |
| 7 | 2.262 | 0.501 | -1.818 |
| 1 | 8.171 | -1.559 | 0.566 |
| 1 | 1.307 | 0.212 | -1.641 |
| 1 | 2.455 | 1.489 | -1.756 |

A•A-T/A•A-T

| 8 | -5.658 | 2.268 | -0.936 |
| --- | --- | --- | --- |
| 8 | -1.175 | 2.943 | -0.523 |
| 8 | -1.412 | -0.263 | 2.841 |
| 8 | -3.514 | -3.659 | 0.638 |
| 7 | -4.911 | 4.281 | -0.170 |
| 7 | -3.407 | 2.653 | -0.822 |
| 7 | -0.967 | 0.017 | -0.736 |
| 7 | -0.894 | -2.942 | -1.455 |
| 7 | -2.702 | -3.896 | -2.371 |
| 7 | -4.155 | -1.942 | -2.560 |
| 7 | -3.075 | 0.026 | -1.712 |
| 7 | 1.885 | -0.161 | -1.542 |
| 7 | 4.023 | -1.258 | -1.589 |
| 7 | 5.837 | 0.344 | -1.863 |
| 7 | 4.658 | 2.243 | -2.000 |
| 7 | 1.610 | 2.137 | -1.815 |
| 7 | 0.739 | 1.624 | 1.479 |
| 7 | 2.473 | 4.008 | 0.670 |
| 7 | 4.702 | 3.817 | 0.741 |
| 7 | 4.905 | 1.489 | 1.430 |
| 7 | 2.747 | 0.498 | 1.811 |
| 7 | -0.188 | -3.613 | 1.891 |
| 7 | 0.677 | -5.282 | 0.397 |
| 7 | 3.033 | -4.882 | -0.103 |
| 7 | 3.392 | -3.064 | 1.153 |
| 7 | 1.063 | -1.837 | 2.713 |
| 7 | -2.498 | -1.965 | 1.795 |
| 7 | -4.491 | -1.598 | 0.685 |
| 6 | -1.413 | 5.574 | 0.487 |
| 6 | -2.320 | 3.400 | -0.437 |
| 6 | -2.588 | 4.734 | 0.088 |
| 6 | -3.877 | 5.108 | 0.195 |
| 6 | -4.725 | 3.007 | -0.670 |
| 6 | -1.437 | -4.059 | -1.858 |
| 6 | -1.940 | -0.627 | -1.386 |
| 6 | -1.867 | -1.999 | -1.696 |
| 6 | -3.007 | -2.569 | -2.251 |
| 6 | -4.093 | -0.652 | -2.270 |
| 6 | 2.411 | 1.059 | -1.726 |
| 6 | 3.813 | 1.164 | -1.839 |
| 6 | 4.523 | -0.029 | -1.749 |
| 6 | 2.700 | -1.227 | -1.489 |
| 6 | 5.849 | 1.710 | -1.999 |
| 6 | 3.616 | 4.606 | 0.463 |
| 6 | 4.088 | 0.494 | 1.754 |
| 6 | 4.216 | 2.594 | 1.123 |
| 6 | 2.834 | 2.743 | 1.093 |
| 6 | 2.078 | 1.610 | 1.467 |
| 6 | -0.272 | -4.708 | 1.122 |
| 6 | 1.836 | -4.615 | 0.509 |
| 6 | 2.084 | -3.484 | 1.278 |
| 6 | 0.981 | -2.949 | 1.978 |
| 6 | 3.920 | -3.919 | 0.322 |
| 6 | -3.496 | -2.500 | 1.014 |
| 6 | -4.477 | -0.281 | 1.068 |
| 6 | -3.496 | 0.226 | 1.842 |
| 6 | -2.393 | -0.655 | 2.203 |
| 6 | -3.435 | 1.638 | 2.336 |
| 1 | -4.168 | 6.076 | 0.581 |
| 1 | -5.871 | 4.562 | -0.061 |
| 1 | -3.252 | 1.674 | -1.192 |
| 1 | -1.062 | 1.019 | -0.631 |
| 1 | -0.019 | -0.347 | -0.791 |
| 1 | -0.963 | -5.026 | -1.790 |
| 1 | -3.340 | -4.622 | -2.644 |
| 1 | -4.974 | -0.054 | -2.480 |
| 1 | 2.196 | -2.176 | -1.342 |
| 1 | 6.774 | 2.254 | -2.105 |
| 1 | 0.638 | 2.051 | -1.564 |
| 1 | 2.030 | 3.041 | -1.675 |
| 1 | 6.629 | -0.269 | -1.794 |
| 1 | -0.738 | 5.710 | -0.358 |
| 1 | -0.834 | 5.082 | 1.270 |
| 1 | -1.739 | 6.550 | 0.846 |
| 1 | -4.299 | 2.210 | 1.996 |
| 1 | -3.410 | 1.656 | 3.427 |
| 1 | -2.519 | 2.124 | 1.994 |
| 1 | 5.667 | 4.032 | 0.565 |
| 1 | 0.250 | 2.426 | 1.116 |
| 1 | 0.210 | 0.822 | 1.794 |
| 1 | 3.731 | 5.621 | 0.116 |
| 1 | 4.545 | -0.460 | 1.994 |
| 1 | -1.260 | -5.154 | 1.079 |
| 1 | 3.212 | -5.634 | -0.744 |
| 1 | 4.934 | -3.886 | -0.040 |
| 1 | 1.829 | -1.195 | 2.528 |
| 1 | 0.193 | -1.428 | 3.032 |
| 1 | -1.692 | -2.601 | 1.972 |
| 1 | -5.170 | -1.940 | 0.025 |
| 1 | -5.292 | 0.325 | 0.690 |

rA•A-T/rA•A-T

| 8 | 5.522 | 0.156 | -1.383 |
| --- | --- | --- | --- |
| 8 | 1.457 | -1.887 | -1.319 |
| 8 | 3.141 | -1.948 | 1.660 |
| 8 | 4.107 | 2.501 | 1.420 |
| 7 | 5.433 | -2.118 | -1.473 |
| 7 | 3.483 | -0.884 | -1.417 |
| 7 | 0.076 | 0.909 | -1.422 |
| 7 | -0.611 | 3.912 | -1.723 |
| 7 | 1.050 | 5.392 | -1.884 |
| 7 | 2.966 | 3.911 | -1.695 |
| 7 | 2.280 | 1.627 | -1.441 |
| 7 | -5.320 | 3.165 | 0.440 |
| 7 | -6.240 | 0.946 | 0.273 |
| 7 | -4.739 | -0.561 | -0.910 |
| 7 | -3.058 | 0.843 | -1.364 |
| 7 | -3.291 | 3.786 | -0.462 |
| 7 | -4.404 | -2.446 | 1.962 |
| 7 | -1.878 | -2.943 | 0.291 |
| 7 | -2.051 | -4.011 | -1.661 |
| 7 | -4.432 | -4.537 | -1.642 |
| 7 | -5.518 | -3.628 | 0.307 |
| 7 | 0.909 | 0.944 | 1.726 |
| 7 | -0.525 | 2.853 | 1.950 |
| 7 | -2.836 | 2.184 | 2.325 |
| 7 | -2.625 | -0.035 | 2.221 |
| 7 | 0.171 | -1.253 | 1.736 |
| 7 | 3.634 | 0.268 | 1.573 |
| 7 | 5.818 | 1.015 | 1.651 |
| 6 | 2.532 | -4.526 | -1.229 |
| 6 | 2.683 | -2.000 | -1.353 |
| 6 | 3.367 | -3.292 | -1.350 |
| 6 | 4.710 | -3.285 | -1.421 |
| 6 | 4.854 | -0.864 | -1.420 |
| 6 | -0.300 | 5.173 | -1.866 |
| 6 | 0.961 | 1.902 | -1.494 |
| 6 | 0.604 | 3.264 | -1.638 |
| 6 | 1.655 | 4.170 | -1.734 |
| 6 | 3.186 | 2.616 | -1.536 |
| 6 | -4.218 | 2.844 | -0.262 |
| 6 | -4.073 | 1.513 | -0.712 |
| 6 | -5.123 | 0.651 | -0.405 |
| 6 | -6.247 | 2.219 | 0.649 |
| 6 | -3.495 | -0.387 | -1.454 |
| 6 | -1.237 | -3.349 | -0.774 |
| 6 | -5.474 | -4.302 | -0.853 |
| 6 | -3.311 | -4.026 | -1.118 |
| 6 | -3.179 | -3.355 | 0.093 |
| 6 | -4.372 | -3.141 | 0.817 |
| 6 | 0.647 | 2.260 | 1.784 |
| 6 | -1.507 | 1.951 | 2.083 |
| 6 | -1.395 | 0.565 | 2.032 |
| 6 | -0.094 | 0.050 | 1.822 |
| 6 | -3.452 | 0.965 | 2.380 |
| 6 | 4.481 | 1.349 | 1.535 |
| 6 | 6.262 | -0.273 | 1.782 |
| 6 | 5.421 | -1.325 | 1.831 |
| 6 | 3.990 | -1.056 | 1.690 |
| 6 | 5.850 | -2.747 | 2.024 |
| 1 | 5.293 | -4.196 | -1.428 |
| 1 | 6.438 | -2.123 | -1.467 |
| 1 | 2.999 | 0.053 | -1.396 |
| 1 | 0.421 | -0.034 | -1.290 |
| 1 | -0.922 | 1.088 | -1.441 |
| 1 | -1.010 | 5.980 | -1.962 |
| 1 | 1.528 | 6.272 | -1.971 |
| 1 | 4.218 | 2.291 | -1.482 |
| 1 | -7.116 | 2.543 | 1.212 |
| 1 | -2.955 | -1.205 | -1.904 |
| 1 | -3.488 | 4.704 | -0.105 |
| 1 | -2.417 | 3.606 | -0.949 |
| 1 | -5.254 | -1.425 | -0.824 |
| 1 | 1.799 | -4.578 | -2.037 |
| 1 | 1.986 | -4.511 | -0.284 |
| 1 | 3.151 | -5.422 | -1.261 |
| 1 | 5.428 | -3.380 | 1.242 |
| 1 | 6.937 | -2.832 | 2.014 |
| 1 | 5.478 | -3.133 | 2.974 |
| 1 | -1.786 | -4.417 | -2.541 |
| 1 | -3.631 | -1.822 | 2.184 |
| 1 | -5.318 | -2.244 | 2.329 |
| 1 | -0.193 | -3.158 | -0.978 |
| 1 | -6.429 | -4.684 | -1.198 |
| 1 | 1.519 | 2.897 | 1.675 |
| 1 | -3.294 | 3.081 | 2.320 |
| 1 | -4.518 | 0.875 | 2.525 |
| 1 | -0.593 | -1.898 | 1.574 |
| 1 | 1.128 | -1.546 | 1.573 |
| 1 | 2.611 | 0.494 | 1.544 |
| 1 | 6.458 | 1.783 | 1.544 |
| 1 | 7.335 | -0.390 | 1.859 |

G4

| 8 | -1.096 | -2.721 | 0.000 |
| --- | --- | --- | --- |
| 8 | -2.721 | 1.096 | 0.000 |
| 8 | 2.721 | -1.096 | 0.000 |
| 8 | 1.096 | 2.721 | 0.000 |
| 7 | 3.320 | -3.958 | 0.000 |
| 7 | 1.797 | -5.687 | 0.000 |
| 7 | 1.079 | -3.419 | 0.000 |
| 7 | -1.797 | -5.706 | 0.000 |
| 7 | -0.115 | -7.176 | 0.000 |
| 7 | -3.958 | -3.319 | 0.000 |
| 7 | -5.687 | -1.797 | 0.000 |
| 7 | -3.419 | -1.078 | 0.000 |
| 7 | -5.706 | 1.797 | 0.000 |
| 7 | -7.176 | 0.115 | 0.000 |
| 7 | 3.958 | 3.320 | 0.000 |
| 7 | 5.687 | 1.797 | 0.000 |
| 7 | 3.419 | 1.079 | 0.000 |
| 7 | 5.706 | -1.797 | 0.000 |
| 7 | 7.176 | -0.115 | 0.000 |
| 7 | -3.319 | 3.958 | 0.000 |
| 7 | -1.797 | 5.687 | 0.000 |
| 7 | -1.078 | 3.419 | 0.000 |
| 7 | 1.797 | 5.706 | 0.000 |
| 7 | 0.115 | 7.176 | 0.000 |
| 6 | 2.055 | -4.391 | 0.000 |
| 6 | -0.291 | -3.657 | 0.000 |
| 6 | -0.583 | -5.051 | 0.000 |
| 6 | 0.478 | -5.945 | 0.000 |
| 6 | -1.479 | -6.969 | 0.000 |
| 6 | -4.390 | -2.055 | 0.000 |
| 6 | -3.656 | 0.292 | 0.000 |
| 6 | -5.051 | 0.583 | 0.000 |
| 6 | -5.945 | -0.478 | 0.000 |
| 6 | -6.969 | 1.479 | 0.000 |
| 6 | 4.390 | 2.055 | 0.000 |
| 6 | 3.656 | -0.291 | 0.000 |
| 6 | 5.050 | -0.584 | 0.000 |
| 6 | 5.944 | 0.478 | 0.000 |
| 6 | 6.968 | -1.479 | 0.000 |
| 6 | -2.055 | 4.391 | 0.000 |
| 6 | 0.292 | 3.657 | 0.000 |
| 6 | 0.584 | 5.051 | 0.000 |
| 6 | -0.478 | 5.945 | 0.000 |
| 6 | 1.479 | 6.969 | 0.000 |
| 1 | 4.049 | -4.646 | 0.000 |
| 1 | 3.548 | -2.973 | 0.000 |
| 1 | -2.175 | -7.792 | 0.000 |
| 1 | 1.375 | -2.445 | 0.000 |
| 1 | -4.646 | -4.048 | 0.000 |
| 1 | -2.973 | -3.548 | 0.000 |
| 1 | -7.792 | 2.175 | 0.000 |
| 1 | -2.444 | -1.374 | 0.000 |
| 1 | 4.646 | 4.048 | 0.000 |
| 1 | 2.973 | 3.548 | 0.000 |
| 1 | 7.792 | -2.175 | 0.000 |
| 1 | 2.444 | 1.375 | 0.000 |
| 1 | -4.048 | 4.646 | 0.000 |
| 1 | -3.548 | 2.973 | 0.000 |
| 1 | 2.175 | 7.792 | 0.000 |
| 1 | -1.374 | 2.445 | 0.000 |
| 1 | -8.056 | -0.371 | 0.000 |
| 1 | -0.370 | 8.056 | 0.000 |
| 1 | 8.056 | 0.370 | 0.000 |
| 1 | 0.370 | -8.056 | 0.000 |

G4∙∙∙Li^+^

| 8 | -1.039 | -1.632 | 0.530 |
| --- | --- | --- | --- |
| 8 | 1.632 | -1.039 | -0.531 |
| 8 | -1.632 | 1.039 | -0.531 |
| 8 | 1.039 | 1.632 | 0.530 |
| 7 | -5.050 | -0.918 | -1.578 |
| 7 | -4.718 | -3.044 | -0.754 |
| 7 | -3.069 | -1.359 | -0.479 |
| 7 | -1.958 | -4.551 | 0.968 |
| 7 | -3.968 | -5.131 | 0.203 |
| 7 | 0.918 | -5.050 | 1.578 |
| 7 | 3.044 | -4.717 | 0.755 |
| 7 | 1.359 | -3.069 | 0.478 |
| 7 | 4.551 | -1.958 | -0.968 |
| 7 | 5.131 | -3.968 | -0.202 |
| 7 | -0.918 | 5.050 | 1.578 |
| 7 | -3.044 | 4.717 | 0.755 |
| 7 | -1.359 | 3.069 | 0.478 |
| 7 | -4.551 | 1.958 | -0.968 |
| 7 | -5.131 | 3.968 | -0.202 |
| 7 | 5.050 | 0.917 | -1.578 |
| 7 | 4.718 | 3.044 | -0.754 |
| 7 | 3.069 | 1.359 | -0.478 |
| 7 | 1.958 | 4.551 | 0.968 |
| 7 | 3.968 | 5.131 | 0.203 |
| 6 | -4.293 | -1.806 | -0.924 |
| 6 | -2.133 | -2.130 | 0.184 |
| 6 | -2.570 | -3.464 | 0.374 |
| 6 | -3.827 | -3.811 | -0.114 |
| 6 | -2.820 | -5.521 | 0.847 |
| 6 | 1.806 | -4.292 | 0.924 |
| 6 | 2.130 | -2.133 | -0.184 |
| 6 | 3.464 | -2.570 | -0.375 |
| 6 | 3.811 | -3.826 | 0.114 |
| 6 | 5.521 | -2.820 | -0.847 |
| 6 | -1.806 | 4.292 | 0.924 |
| 6 | -2.130 | 2.133 | -0.184 |
| 6 | -3.465 | 2.570 | -0.374 |
| 6 | -3.811 | 3.826 | 0.114 |
| 6 | -5.521 | 2.820 | -0.846 |
| 6 | 4.293 | 1.806 | -0.924 |
| 6 | 2.133 | 2.130 | 0.184 |
| 6 | 2.570 | 3.464 | 0.374 |
| 6 | 3.827 | 3.811 | -0.114 |
| 6 | 2.820 | 5.521 | 0.847 |
| 3 | 0.000 | 0.000 | -0.001 |
| 1 | -5.962 | -1.240 | -1.849 |
| 1 | -4.859 | 0.081 | -1.521 |
| 1 | -2.682 | -6.530 | 1.201 |
| 1 | -2.770 | -0.395 | -0.660 |
| 1 | 1.240 | -5.962 | 1.850 |
| 1 | -0.081 | -4.858 | 1.521 |
| 1 | 6.530 | -2.682 | -1.200 |
| 1 | 0.395 | -2.769 | 0.659 |
| 1 | -1.240 | 5.962 | 1.849 |
| 1 | 0.081 | 4.858 | 1.521 |
| 1 | -6.531 | 2.682 | -1.200 |
| 1 | -0.395 | 2.769 | 0.659 |
| 1 | 5.962 | 1.240 | -1.849 |
| 1 | 4.859 | -0.082 | -1.521 |
| 1 | 2.682 | 6.530 | 1.200 |
| 1 | 2.770 | 0.395 | -0.660 |
| 1 | 4.773 | 5.698 | -0.007 |
| 1 | 5.698 | -4.773 | 0.008 |
| 1 | -4.773 | -5.698 | -0.007 |
| 1 | -5.699 | 4.773 | 0.008 |

G4∙∙∙Na^+^

| 11 | 0.000 | 0.000 | -0.002 |
| --- | --- | --- | --- |
| 8 | -0.560 | -2.213 | 0.000 |
| 8 | 2.213 | -0.560 | 0.001 |
| 8 | -2.213 | 0.560 | 0.001 |
| 8 | 0.560 | 2.213 | 0.001 |
| 7 | -5.156 | -2.178 | 0.007 |
| 7 | -4.170 | -4.250 | 0.003 |
| 7 | -2.843 | -2.278 | 0.003 |
| 7 | -0.746 | -5.299 | 0.001 |
| 7 | -2.774 | -6.219 | 0.001 |
| 7 | 2.179 | -5.155 | -0.001 |
| 7 | 4.250 | -4.170 | -0.004 |
| 7 | 2.278 | -2.843 | -0.001 |
| 7 | 5.300 | -0.746 | -0.001 |
| 7 | 6.219 | -2.774 | -0.005 |
| 7 | -2.179 | 5.155 | 0.000 |
| 7 | -4.251 | 4.170 | -0.005 |
| 7 | -2.278 | 2.843 | -0.001 |
| 7 | -5.299 | 0.746 | -0.001 |
| 7 | -6.219 | 2.773 | -0.006 |
| 7 | 5.156 | 2.178 | 0.006 |
| 7 | 4.170 | 4.250 | 0.003 |
| 7 | 2.843 | 2.278 | 0.002 |
| 7 | 0.747 | 5.300 | 0.001 |
| 7 | 2.774 | 6.219 | 0.001 |
| 6 | -4.056 | -2.934 | 0.004 |
| 6 | -1.603 | -2.898 | 0.001 |
| 6 | -1.711 | -4.312 | 0.002 |
| 6 | -2.985 | -4.870 | 0.002 |
| 6 | -1.416 | -6.417 | 0.001 |
| 6 | 2.934 | -4.056 | -0.002 |
| 6 | 2.898 | -1.603 | 0.000 |
| 6 | 4.312 | -1.711 | -0.001 |
| 6 | 4.870 | -2.985 | -0.003 |
| 6 | 6.418 | -1.416 | -0.003 |
| 6 | -2.934 | 4.056 | -0.002 |
| 6 | -2.898 | 1.603 | 0.000 |
| 6 | -4.312 | 1.710 | -0.001 |
| 6 | -4.870 | 2.985 | -0.004 |
| 6 | -6.418 | 1.415 | -0.004 |
| 6 | 4.056 | 2.934 | 0.003 |
| 6 | 1.603 | 2.898 | 0.002 |
| 6 | 1.711 | 4.312 | 0.002 |
| 6 | 2.985 | 4.870 | 0.002 |
| 6 | 1.416 | 6.418 | 0.001 |
| 1 | -6.027 | -2.678 | 0.005 |
| 1 | -5.147 | -1.157 | 0.003 |
| 1 | -0.986 | -7.406 | 0.000 |
| 1 | -2.819 | -1.253 | 0.003 |
| 1 | 2.678 | -6.027 | -0.005 |
| 1 | 1.157 | -5.147 | -0.003 |
| 1 | 7.407 | -0.986 | -0.003 |
| 1 | 1.253 | -2.819 | 0.000 |
| 1 | -2.678 | 6.027 | -0.004 |
| 1 | -1.158 | 5.147 | -0.001 |
| 1 | -7.406 | 0.985 | -0.004 |
| 1 | -1.254 | 2.819 | 0.001 |
| 1 | 6.027 | 2.678 | 0.004 |
| 1 | 5.147 | 1.157 | 0.002 |
| 1 | 0.986 | 7.406 | 0.001 |
| 1 | 2.819 | 1.253 | 0.003 |
| 1 | -6.923 | 3.493 | -0.007 |
| 1 | -3.494 | -6.922 | 0.003 |
| 1 | 6.922 | -3.494 | -0.005 |
| 1 | 3.494 | 6.922 | 0.003 |

G4∙∙∙K^+^

| 19 | 0.000 | 0.000 | 1.465 |
| --- | --- | --- | --- |
| 8 | -2.061 | -1.173 | 0.373 |
| 8 | 1.207 | -2.089 | 0.473 |
| 8 | -1.207 | 2.089 | 0.473 |
| 8 | 2.061 | 1.173 | 0.374 |
| 7 | -5.124 | 2.253 | 0.247 |
| 7 | -5.989 | 0.127 | 0.164 |
| 7 | -3.640 | 0.475 | 0.267 |
| 7 | -4.452 | -3.110 | 0.157 |
| 7 | -6.500 | -2.234 | 0.099 |
| 7 | -2.237 | -4.958 | -0.552 |
| 7 | -0.118 | -5.806 | -0.792 |
| 7 | -0.451 | -3.567 | -0.067 |
| 7 | 3.129 | -4.382 | -0.278 |
| 7 | 2.240 | -6.316 | -0.935 |
| 7 | 2.237 | 4.958 | -0.552 |
| 7 | 0.118 | 5.806 | -0.792 |
| 7 | 0.451 | 3.567 | -0.067 |
| 7 | -3.129 | 4.382 | -0.278 |
| 7 | -2.240 | 6.316 | -0.935 |
| 7 | 5.124 | -2.253 | 0.247 |
| 7 | 5.989 | -0.127 | 0.164 |
| 7 | 3.640 | -0.475 | 0.267 |
| 7 | 4.453 | 3.110 | 0.157 |
| 7 | 6.500 | 2.234 | 0.099 |
| 6 | -4.941 | 0.930 | 0.217 |
| 6 | -3.263 | -0.863 | 0.277 |
| 6 | -4.379 | -1.732 | 0.188 |
| 6 | -5.649 | -1.168 | 0.151 |
| 6 | -5.728 | -3.371 | 0.102 |
| 6 | -0.913 | -4.800 | -0.475 |
| 6 | 0.889 | -3.225 | 0.077 |
| 6 | 1.752 | -4.296 | -0.269 |
| 6 | 1.179 | -5.495 | -0.677 |
| 6 | 3.382 | -5.596 | -0.680 |
| 6 | 0.913 | 4.800 | -0.475 |
| 6 | -0.889 | 3.225 | 0.077 |
| 6 | -1.752 | 4.296 | -0.269 |
| 6 | -1.179 | 5.495 | -0.677 |
| 6 | -3.382 | 5.596 | -0.680 |
| 6 | 4.941 | -0.930 | 0.217 |
| 6 | 3.263 | 0.863 | 0.277 |
| 6 | 4.379 | 1.732 | 0.188 |
| 6 | 5.649 | 1.167 | 0.151 |
| 6 | 5.728 | 3.371 | 0.102 |
| 1 | -6.078 | 2.556 | 0.165 |
| 1 | -4.374 | 2.935 | 0.127 |
| 1 | -6.166 | -4.355 | 0.062 |
| 1 | -2.874 | 1.155 | 0.306 |
| 1 | -2.549 | -5.868 | -0.841 |
| 1 | -2.922 | -4.266 | -0.249 |
| 1 | 4.363 | -6.023 | -0.810 |
| 1 | -1.126 | -2.815 | 0.103 |
| 1 | 2.549 | 5.868 | -0.841 |
| 1 | 2.922 | 4.266 | -0.249 |
| 1 | -4.363 | 6.023 | -0.810 |
| 1 | 1.126 | 2.815 | 0.104 |
| 1 | 6.078 | -2.556 | 0.165 |
| 1 | 4.374 | -2.935 | 0.127 |
| 1 | 6.167 | 4.355 | 0.062 |
| 1 | 2.874 | -1.155 | 0.306 |
| 1 | 2.177 | -7.269 | -1.251 |
| 1 | 7.504 | 2.179 | 0.064 |
| 1 | -2.177 | 7.269 | -1.251 |
| 1 | -7.504 | -2.179 | 0.064 |

G4-G4

| 8 | 0.194 | -2.977 | 1.218 |
| --- | --- | --- | --- |
| 8 | 2.977 | 0.194 | 1.218 |
| 8 | -2.977 | -0.194 | 1.218 |
| 8 | -0.194 | 2.977 | 1.218 |
| 7 | -4.357 | -2.662 | 1.685 |
| 7 | -3.496 | -4.802 | 1.708 |
| 7 | -2.074 | -2.908 | 1.474 |
| 7 | -0.129 | -6.027 | 1.433 |
| 7 | -2.192 | -6.840 | 1.693 |
| 7 | 2.662 | -4.357 | 1.685 |
| 7 | 4.802 | -3.496 | 1.708 |
| 7 | 2.908 | -2.074 | 1.474 |
| 7 | 6.027 | -0.129 | 1.433 |
| 7 | 6.840 | -2.192 | 1.693 |
| 7 | -2.662 | 4.357 | 1.685 |
| 7 | -4.802 | 3.496 | 1.708 |
| 7 | -2.908 | 2.074 | 1.474 |
| 7 | -6.027 | 0.129 | 1.433 |
| 7 | -6.840 | 2.192 | 1.693 |
| 7 | 4.357 | 2.662 | 1.685 |
| 7 | 3.496 | 4.802 | 1.708 |
| 7 | 2.074 | 2.908 | 1.474 |
| 7 | 0.129 | 6.027 | 1.433 |
| 7 | 2.192 | 6.840 | 1.693 |
| 6 | -3.313 | -3.496 | 1.615 |
| 6 | -0.867 | -3.590 | 1.373 |
| 6 | -1.049 | -4.999 | 1.480 |
| 6 | -2.340 | -5.483 | 1.636 |
| 6 | -0.845 | -7.106 | 1.561 |
| 6 | 3.496 | -3.313 | 1.615 |
| 6 | 3.590 | -0.867 | 1.373 |
| 6 | 4.999 | -1.049 | 1.480 |
| 6 | 5.483 | -2.340 | 1.636 |
| 6 | 7.106 | -0.845 | 1.561 |
| 6 | -3.496 | 3.313 | 1.615 |
| 6 | -3.590 | 0.867 | 1.373 |
| 6 | -4.999 | 1.049 | 1.480 |
| 6 | -5.483 | 2.340 | 1.636 |
| 6 | -7.106 | 0.845 | 1.561 |
| 6 | 3.313 | 3.496 | 1.615 |
| 6 | 0.867 | 3.590 | 1.373 |
| 6 | 1.049 | 4.999 | 1.480 |
| 6 | 2.340 | 5.483 | 1.636 |
| 6 | 0.845 | 7.106 | 1.561 |
| 1 | -5.272 | -3.067 | 1.609 |
| 1 | -4.238 | -1.677 | 1.484 |
| 1 | -0.463 | -8.115 | 1.578 |
| 1 | -2.036 | -1.894 | 1.381 |
| 1 | 3.067 | -5.272 | 1.609 |
| 1 | 1.677 | -4.238 | 1.484 |
| 1 | 8.115 | -0.463 | 1.578 |
| 1 | 1.894 | -2.036 | 1.381 |
| 1 | -3.067 | 5.272 | 1.609 |
| 1 | -1.677 | 4.238 | 1.484 |
| 1 | -8.115 | 0.463 | 1.578 |
| 1 | -1.894 | 2.036 | 1.381 |
| 1 | 5.272 | 3.067 | 1.609 |
| 1 | 4.238 | 1.677 | 1.484 |
| 1 | 0.463 | 8.115 | 1.578 |
| 1 | 2.036 | 1.894 | 1.381 |
| 1 | 7.502 | -2.939 | 1.807 |
| 1 | 2.939 | 7.502 | 1.807 |
| 1 | -7.502 | 2.939 | 1.807 |
| 1 | -2.939 | -7.502 | 1.807 |
| 8 | -1.114 | -2.765 | -1.733 |
| 8 | 2.765 | -1.114 | -1.733 |
| 8 | -2.765 | 1.114 | -1.733 |
| 8 | 1.114 | 2.765 | -1.733 |
| 7 | -5.125 | -0.556 | -1.568 |
| 7 | -5.248 | -2.856 | -1.439 |
| 7 | -3.154 | -1.744 | -1.639 |
| 7 | -2.699 | -5.389 | -1.534 |
| 7 | -4.923 | -5.254 | -1.364 |
| 7 | 0.556 | -5.125 | -1.568 |
| 7 | 2.856 | -5.248 | -1.439 |
| 7 | 1.744 | -3.154 | -1.639 |
| 7 | 5.389 | -2.699 | -1.534 |
| 7 | 5.254 | -4.923 | -1.364 |
| 7 | -0.556 | 5.125 | -1.568 |
| 7 | -2.856 | 5.248 | -1.439 |
| 7 | -1.744 | 3.154 | -1.639 |
| 7 | -5.389 | 2.699 | -1.534 |
| 7 | -5.254 | 4.923 | -1.364 |
| 7 | 5.125 | 0.556 | -1.568 |
| 7 | 5.248 | 2.856 | -1.439 |
| 7 | 3.154 | 1.744 | -1.639 |
| 7 | 2.699 | 5.389 | -1.534 |
| 7 | 4.923 | 5.254 | -1.364 |
| 6 | -4.528 | -1.750 | -1.537 |
| 6 | -2.342 | -2.871 | -1.661 |
| 6 | -3.106 | -4.072 | -1.567 |
| 6 | -4.486 | -3.963 | -1.456 |
| 6 | -3.807 | -6.061 | -1.409 |
| 6 | 1.750 | -4.528 | -1.537 |
| 6 | 2.871 | -2.342 | -1.661 |
| 6 | 4.072 | -3.106 | -1.567 |
| 6 | 3.963 | -4.486 | -1.456 |
| 6 | 6.061 | -3.807 | -1.409 |
| 6 | -1.750 | 4.528 | -1.537 |
| 6 | -2.871 | 2.342 | -1.661 |
| 6 | -4.072 | 3.106 | -1.567 |
| 6 | -3.963 | 4.486 | -1.456 |
| 6 | -6.061 | 3.807 | -1.409 |
| 6 | 4.528 | 1.750 | -1.537 |
| 6 | 2.342 | 2.871 | -1.661 |
| 6 | 3.106 | 4.072 | -1.567 |
| 6 | 4.486 | 3.963 | -1.456 |
| 6 | 3.807 | 6.061 | -1.409 |
| 1 | -6.082 | -0.515 | -1.270 |
| 1 | -4.574 | 0.293 | -1.575 |
| 1 | -3.885 | -7.136 | -1.353 |
| 1 | -2.683 | -0.840 | -1.649 |
| 1 | 0.515 | -6.082 | -1.270 |
| 1 | -0.293 | -4.574 | -1.575 |
| 1 | 7.136 | -3.885 | -1.353 |
| 1 | 0.840 | -2.683 | -1.649 |
| 1 | -0.515 | 6.082 | -1.270 |
| 1 | 0.293 | 4.574 | -1.575 |
| 1 | -7.136 | 3.885 | -1.353 |
| 1 | -0.840 | 2.683 | -1.649 |
| 1 | 6.082 | 0.515 | -1.270 |
| 1 | 4.574 | -0.293 | -1.575 |
| 1 | 3.885 | 7.136 | -1.353 |
| 1 | 2.683 | 0.840 | -1.649 |
| 1 | 5.536 | -5.880 | -1.251 |
| 1 | 5.880 | 5.536 | -1.251 |
| 1 | -5.536 | 5.880 | -1.251 |
| 1 | -5.880 | -5.536 | -1.251 |

G4∙∙∙Li^+^∙∙∙G4

| 8 | 1.908 | -1.272 | -1.254 |
| --- | --- | --- | --- |
| 8 | -1.424 | -1.790 | -1.306 |
| 8 | 1.403 | 1.766 | -1.169 |
| 8 | -1.922 | 1.308 | -1.554 |
| 7 | 5.452 | 1.649 | -1.493 |
| 7 | 5.979 | -0.579 | -1.644 |
| 7 | 3.718 | 0.119 | -1.416 |
| 7 | 3.972 | -3.541 | -1.574 |
| 7 | 6.121 | -2.988 | -1.733 |
| 7 | 1.612 | -5.192 | -1.899 |
| 7 | -0.592 | -5.813 | -1.840 |
| 7 | 0.029 | -3.525 | -1.611 |
| 7 | -3.613 | -3.968 | -1.373 |
| 7 | -2.985 | -6.077 | -1.657 |
| 7 | -1.590 | 5.147 | -2.030 |
| 7 | 0.613 | 5.774 | -1.888 |
| 7 | -0.015 | 3.503 | -1.607 |
| 7 | 3.614 | 3.925 | -1.250 |
| 7 | 3.003 | 6.031 | -1.630 |
| 7 | -5.286 | -1.809 | -1.350 |
| 7 | -5.946 | 0.369 | -1.455 |
| 7 | -3.606 | -0.234 | -1.462 |
| 7 | -4.117 | 3.386 | -1.674 |
| 7 | -6.267 | 2.755 | -1.584 |
| 6 | 5.068 | 0.370 | -1.523 |
| 6 | 3.141 | -1.140 | -1.405 |
| 6 | 4.105 | -2.168 | -1.558 |
| 6 | 5.444 | -1.804 | -1.653 |
| 6 | 5.189 | -3.994 | -1.680 |
| 6 | 0.326 | -4.860 | -1.783 |
| 6 | -1.249 | -3.010 | -1.462 |
| 6 | -2.240 | -4.029 | -1.507 |
| 6 | -1.830 | -5.342 | -1.686 |
| 6 | -4.021 | -5.207 | -1.465 |
| 6 | -0.299 | 4.826 | -1.841 |
| 6 | 1.253 | 2.976 | -1.380 |
| 6 | 2.249 | 3.992 | -1.429 |
| 6 | 1.850 | 5.301 | -1.670 |
| 6 | 4.028 | 5.156 | -1.375 |
| 6 | -4.928 | -0.553 | -1.419 |
| 6 | -3.110 | 1.063 | -1.532 |
| 6 | -4.178 | 2.053 | -1.583 |
| 6 | -5.525 | 1.597 | -1.524 |
| 6 | -5.388 | 3.783 | -1.659 |
| 1 | 6.432 | 1.816 | -1.631 |
| 1 | 4.799 | 2.425 | -1.388 |
| 1 | 5.471 | -5.033 | -1.726 |
| 1 | 3.046 | 0.890 | -1.332 |
| 1 | 1.794 | -6.168 | -2.049 |
| 1 | 2.391 | -4.538 | -1.785 |
| 1 | -5.045 | -5.537 | -1.403 |
| 1 | 0.783 | -2.832 | -1.558 |
| 1 | -1.781 | 6.112 | -2.230 |
| 1 | -2.349 | 4.488 | -1.934 |
| 1 | 5.052 | 5.485 | -1.292 |
| 1 | -0.766 | 2.815 | -1.595 |
| 1 | -6.280 | -1.973 | -1.317 |
| 1 | -4.604 | -2.624 | -1.325 |
| 1 | -5.706 | 4.812 | -1.702 |
| 1 | -2.850 | -0.971 | -1.390 |
| 1 | -7.274 | 2.817 | -1.574 |
| 1 | -3.046 | -7.074 | -1.783 |
| 1 | 7.117 | -3.086 | -1.836 |
| 1 | 3.075 | 7.021 | -1.792 |
| 8 | 2.246 | 0.376 | 1.271 |
| 8 | 0.504 | -2.110 | 1.122 |
| 8 | -0.402 | 2.341 | 1.261 |
| 8 | -2.313 | -0.278 | 1.262 |
| 7 | 2.435 | 4.938 | 1.831 |
| 7 | 4.435 | 3.825 | 1.968 |
| 7 | 2.412 | 2.636 | 1.585 |
| 7 | 5.302 | 0.368 | 1.637 |
| 7 | 6.321 | 2.315 | 1.993 |
| 7 | 5.047 | -2.546 | 1.683 |
| 7 | 3.857 | -4.508 | 1.636 |
| 7 | 2.749 | -2.411 | 1.459 |
| 7 | 0.370 | -5.198 | 1.236 |
| 7 | 2.273 | -6.321 | 1.482 |
| 7 | -4.999 | 2.532 | 1.360 |
| 7 | -3.903 | 4.551 | 1.342 |
| 7 | -2.677 | 2.515 | 1.359 |
| 7 | -0.430 | 5.423 | 1.243 |
| 7 | -2.402 | 6.454 | 1.263 |
| 7 | -2.427 | -4.853 | 1.747 |
| 7 | -4.424 | -3.767 | 1.999 |
| 7 | -2.430 | -2.545 | 1.542 |
| 7 | -5.352 | -0.321 | 1.770 |
| 7 | -6.326 | -2.284 | 2.152 |
| 6 | 3.125 | 3.793 | 1.799 |
| 6 | 2.965 | 1.372 | 1.489 |
| 6 | 4.372 | 1.387 | 1.664 |
| 6 | 4.991 | 2.612 | 1.888 |
| 6 | 6.448 | 0.958 | 1.835 |
| 6 | 3.878 | -3.187 | 1.598 |
| 6 | 1.458 | -2.893 | 1.298 |
| 6 | 1.421 | -4.313 | 1.350 |
| 6 | 2.621 | -4.999 | 1.507 |
| 6 | 0.914 | -6.381 | 1.319 |
| 6 | -3.842 | 3.236 | 1.380 |
| 6 | -1.394 | 3.068 | 1.305 |
| 6 | -1.444 | 4.495 | 1.293 |
| 6 | -2.682 | 5.118 | 1.307 |
| 6 | -1.035 | 6.579 | 1.228 |
| 6 | -3.120 | -3.715 | 1.772 |
| 6 | -3.001 | -1.275 | 1.500 |
| 6 | -4.407 | -1.326 | 1.741 |
| 6 | -4.996 | -2.560 | 1.976 |
| 6 | -6.480 | -0.927 | 2.025 |
| 3 | 0.745 | -0.252 | 0.087 |
| 1 | 2.971 | 5.764 | 2.030 |
| 1 | 1.440 | 5.014 | 1.630 |
| 1 | 7.407 | 0.467 | 1.879 |
| 1 | 1.391 | 2.671 | 1.481 |
| 1 | 5.852 | -3.120 | 1.857 |
| 1 | 5.124 | -1.528 | 1.665 |
| 1 | 0.385 | -7.319 | 1.267 |
| 1 | 2.822 | -1.389 | 1.457 |
| 1 | -5.799 | 3.082 | 1.627 |
| 1 | -5.022 | 1.538 | 1.611 |
| 1 | -0.551 | 7.542 | 1.193 |
| 1 | -2.691 | 1.485 | 1.399 |
| 1 | -2.943 | -5.680 | 1.991 |
| 1 | -1.423 | -4.907 | 1.549 |
| 1 | -7.440 | -0.449 | 2.136 |
| 1 | -1.414 | -2.557 | 1.394 |
| 1 | -7.033 | -2.955 | 2.405 |
| 1 | 2.908 | -7.093 | 1.600 |
| 1 | 7.054 | 2.976 | 2.189 |
| 1 | -3.077 | 7.200 | 1.305 |

G4∙∙∙Na^+^∙∙∙G4

| 8 | -1.187 | 1.953 | 1.275 |
| --- | --- | --- | --- |
| 8 | -1.953 | -1.186 | 1.275 |
| 8 | 1.953 | 1.186 | 1.275 |
| 8 | 1.186 | -1.953 | 1.275 |
| 7 | 2.100 | 5.136 | 1.728 |
| 7 | -0.055 | 5.915 | 1.742 |
| 7 | 0.393 | 3.588 | 1.517 |
| 7 | -3.218 | 4.269 | 1.435 |
| 7 | -2.433 | 6.338 | 1.660 |
| 7 | -5.137 | 2.100 | 1.727 |
| 7 | -5.916 | -0.055 | 1.740 |
| 7 | -3.589 | 0.393 | 1.516 |
| 7 | -4.270 | -3.218 | 1.434 |
| 7 | -6.338 | -2.433 | 1.658 |
| 7 | 5.137 | -2.100 | 1.727 |
| 7 | 5.915 | 0.055 | 1.740 |
| 7 | 3.589 | -0.393 | 1.516 |
| 7 | 4.269 | 3.218 | 1.434 |
| 7 | 6.338 | 2.433 | 1.658 |
| 7 | -2.100 | -5.136 | 1.728 |
| 7 | 0.055 | -5.915 | 1.742 |
| 7 | -0.393 | -3.588 | 1.517 |
| 7 | 3.218 | -4.269 | 1.436 |
| 7 | 2.433 | -6.337 | 1.661 |
| 6 | 0.786 | 4.897 | 1.662 |
| 6 | -0.925 | 3.153 | 1.422 |
| 6 | -1.842 | 4.242 | 1.507 |
| 6 | -1.332 | 5.525 | 1.649 |
| 6 | -3.535 | 5.530 | 1.528 |
| 6 | -4.898 | 0.786 | 1.661 |
| 6 | -3.153 | -0.925 | 1.421 |
| 6 | -4.242 | -1.842 | 1.506 |
| 6 | -5.525 | -1.332 | 1.647 |
| 6 | -5.530 | -3.535 | 1.526 |
| 6 | 4.898 | -0.786 | 1.661 |
| 6 | 3.153 | 0.925 | 1.422 |
| 6 | 4.242 | 1.842 | 1.506 |
| 6 | 5.525 | 1.332 | 1.647 |
| 6 | 5.530 | 3.535 | 1.526 |
| 6 | -0.786 | -4.897 | 1.662 |
| 6 | 0.925 | -3.153 | 1.422 |
| 6 | 1.842 | -4.242 | 1.507 |
| 6 | 1.332 | -5.525 | 1.649 |
| 6 | 3.535 | -5.530 | 1.528 |
| 1 | 2.360 | 6.099 | 1.838 |
| 1 | 2.816 | 4.418 | 1.604 |
| 1 | -4.537 | 5.929 | 1.505 |
| 1 | 1.091 | 2.838 | 1.453 |
| 1 | -6.100 | 2.360 | 1.836 |
| 1 | -4.419 | 2.816 | 1.603 |
| 1 | -5.929 | -4.537 | 1.503 |
| 1 | -2.838 | 1.091 | 1.453 |
| 1 | 6.100 | -2.360 | 1.837 |
| 1 | 4.419 | -2.816 | 1.604 |
| 1 | 5.929 | 4.537 | 1.503 |
| 1 | 2.838 | -1.091 | 1.453 |
| 1 | -2.360 | -6.099 | 1.838 |
| 1 | -2.816 | -4.418 | 1.603 |
| 1 | 4.537 | -5.928 | 1.506 |
| 1 | -1.091 | -2.838 | 1.453 |
| 1 | 7.337 | 2.419 | 1.771 |
| 1 | -2.419 | 7.336 | 1.774 |
| 1 | -7.337 | -2.419 | 1.771 |
| 1 | 2.419 | -7.336 | 1.774 |
| 11 | 0.000 | 0.000 | -0.009 |
| 8 | 0.542 | 2.218 | -1.279 |
| 8 | -2.218 | 0.542 | -1.280 |
| 8 | 2.218 | -0.542 | -1.280 |
| 8 | -0.542 | -2.218 | -1.279 |
| 7 | 5.117 | 2.147 | -1.731 |
| 7 | 4.144 | 4.222 | -1.740 |
| 7 | 2.816 | 2.259 | -1.520 |
| 7 | 0.744 | 5.293 | -1.431 |
| 7 | 2.761 | 6.201 | -1.654 |
| 7 | -2.147 | 5.118 | -1.730 |
| 7 | -4.221 | 4.144 | -1.739 |
| 7 | -2.259 | 2.816 | -1.520 |
| 7 | -5.293 | 0.744 | -1.432 |
| 7 | -6.201 | 2.761 | -1.655 |
| 7 | 2.147 | -5.118 | -1.729 |
| 7 | 4.222 | -4.144 | -1.739 |
| 7 | 2.259 | -2.816 | -1.520 |
| 7 | 5.293 | -0.744 | -1.432 |
| 7 | 6.201 | -2.761 | -1.654 |
| 7 | -5.117 | -2.147 | -1.732 |
| 7 | -4.144 | -4.222 | -1.740 |
| 7 | -2.816 | -2.259 | -1.520 |
| 7 | -0.744 | -5.293 | -1.431 |
| 7 | -2.761 | -6.201 | -1.654 |
| 6 | 4.019 | 2.907 | -1.664 |
| 6 | 1.576 | 2.883 | -1.424 |
| 6 | 1.698 | 4.301 | -1.504 |
| 6 | 2.965 | 4.848 | -1.645 |
| 6 | 1.411 | 6.409 | -1.521 |
| 6 | -2.907 | 4.019 | -1.663 |
| 6 | -2.883 | 1.576 | -1.424 |
| 6 | -4.301 | 1.698 | -1.505 |
| 6 | -4.848 | 2.965 | -1.646 |
| 6 | -6.409 | 1.411 | -1.523 |
| 6 | 2.907 | -4.020 | -1.662 |
| 6 | 2.883 | -1.576 | -1.424 |
| 6 | 4.301 | -1.698 | -1.505 |
| 6 | 4.848 | -2.965 | -1.645 |
| 6 | 6.409 | -1.411 | -1.522 |
| 6 | -4.019 | -2.907 | -1.664 |
| 6 | -1.576 | -2.883 | -1.424 |
| 6 | -1.698 | -4.301 | -1.505 |
| 6 | -2.965 | -4.848 | -1.646 |
| 6 | -1.411 | -6.409 | -1.522 |
| 1 | 5.982 | 2.643 | -1.839 |
| 1 | 5.115 | 1.133 | -1.604 |
| 1 | 0.984 | 7.399 | -1.496 |
| 1 | 2.779 | 1.234 | -1.460 |
| 1 | -2.643 | 5.982 | -1.837 |
| 1 | -1.133 | 5.116 | -1.603 |
| 1 | -7.399 | 0.984 | -1.499 |
| 1 | -1.234 | 2.779 | -1.459 |
| 1 | 2.644 | -5.982 | -1.837 |
| 1 | 1.133 | -5.116 | -1.603 |
| 1 | 7.399 | -0.984 | -1.498 |
| 1 | 1.234 | -2.779 | -1.459 |
| 1 | -5.982 | -2.643 | -1.840 |
| 1 | -5.115 | -1.133 | -1.605 |
| 1 | -0.984 | -7.399 | -1.497 |
| 1 | -2.779 | -1.234 | -1.460 |
| 1 | 6.898 | -3.478 | -1.767 |
| 1 | 3.477 | 6.898 | -1.767 |
| 1 | -6.898 | 3.477 | -1.768 |
| 1 | -3.477 | -6.898 | -1.768 |

G4∙∙∙K^+^∙∙∙G4

| 8 | 2.322 | -0.548 | 1.388 |
| --- | --- | --- | --- |
| 8 | 0.548 | 2.322 | 1.388 |
| 8 | -0.548 | -2.322 | 1.388 |
| 8 | -2.322 | 0.548 | 1.388 |
| 7 | 2.106 | -5.130 | 1.713 |
| 7 | 4.213 | -4.222 | 1.709 |
| 7 | 2.290 | -2.829 | 1.556 |
| 7 | 5.381 | -0.843 | 1.481 |
| 7 | 6.234 | -2.893 | 1.633 |
| 7 | 5.130 | 2.106 | 1.713 |
| 7 | 4.222 | 4.213 | 1.710 |
| 7 | 2.829 | 2.290 | 1.556 |
| 7 | 0.843 | 5.381 | 1.481 |
| 7 | 2.893 | 6.234 | 1.633 |
| 7 | -5.130 | -2.106 | 1.713 |
| 7 | -4.222 | -4.213 | 1.710 |
| 7 | -2.829 | -2.290 | 1.556 |
| 7 | -0.843 | -5.381 | 1.481 |
| 7 | -2.893 | -6.234 | 1.633 |
| 7 | -2.106 | 5.130 | 1.713 |
| 7 | -4.213 | 4.222 | 1.709 |
| 7 | -2.290 | 2.829 | 1.556 |
| 7 | -5.381 | 0.843 | 1.481 |
| 7 | -6.234 | 2.893 | 1.633 |
| 6 | 2.902 | -4.055 | 1.658 |
| 6 | 2.954 | -1.607 | 1.487 |
| 6 | 4.366 | -1.773 | 1.541 |
| 6 | 4.876 | -3.060 | 1.638 |
| 6 | 6.478 | -1.544 | 1.537 |
| 6 | 4.055 | 2.902 | 1.658 |
| 6 | 1.607 | 2.954 | 1.487 |
| 6 | 1.773 | 4.366 | 1.541 |
| 6 | 3.060 | 4.876 | 1.638 |
| 6 | 1.544 | 6.478 | 1.537 |
| 6 | -4.055 | -2.902 | 1.658 |
| 6 | -1.607 | -2.954 | 1.487 |
| 6 | -1.773 | -4.366 | 1.541 |
| 6 | -3.060 | -4.876 | 1.638 |
| 6 | -1.544 | -6.478 | 1.537 |
| 6 | -2.902 | 4.055 | 1.658 |
| 6 | -2.954 | 1.607 | 1.487 |
| 6 | -4.366 | 1.773 | 1.541 |
| 6 | -4.876 | 3.060 | 1.638 |
| 6 | -6.478 | 1.544 | 1.537 |
| 1 | 2.575 | -6.014 | 1.786 |
| 1 | 1.092 | -5.105 | 1.611 |
| 1 | 7.480 | -1.146 | 1.511 |
| 1 | 1.266 | -2.773 | 1.523 |
| 1 | 6.014 | 2.575 | 1.786 |
| 1 | 5.105 | 1.092 | 1.611 |
| 1 | 1.146 | 7.480 | 1.512 |
| 1 | 2.773 | 1.266 | 1.523 |
| 1 | -6.014 | -2.575 | 1.786 |
| 1 | -5.105 | -1.092 | 1.611 |
| 1 | -1.146 | -7.480 | 1.512 |
| 1 | -2.773 | -1.266 | 1.523 |
| 1 | -2.575 | 6.014 | 1.786 |
| 1 | -1.092 | 5.105 | 1.611 |
| 1 | -7.480 | 1.146 | 1.512 |
| 1 | -1.266 | 2.773 | 1.523 |
| 1 | 3.630 | 6.913 | 1.721 |
| 1 | -6.913 | 3.630 | 1.721 |
| 1 | -3.630 | -6.913 | 1.721 |
| 1 | 6.913 | -3.630 | 1.721 |
| 19 | 0.000 | 0.000 | -0.005 |
| 8 | 1.257 | -2.029 | -1.396 |
| 8 | 2.029 | 1.257 | -1.396 |
| 8 | -2.029 | -1.257 | -1.396 |
| 8 | -1.257 | 2.029 | -1.396 |
| 7 | -2.133 | -5.120 | -1.717 |
| 7 | 0.000 | -5.966 | -1.706 |
| 7 | -0.377 | -3.621 | -1.562 |
| 7 | 3.214 | -4.399 | -1.479 |
| 7 | 2.369 | -6.453 | -1.623 |
| 7 | 5.120 | -2.133 | -1.717 |
| 7 | 5.966 | 0.000 | -1.706 |
| 7 | 3.621 | -0.377 | -1.562 |
| 7 | 4.399 | 3.214 | -1.479 |
| 7 | 6.453 | 2.369 | -1.623 |
| 7 | -5.120 | 2.133 | -1.717 |
| 7 | -5.966 | 0.000 | -1.705 |
| 7 | -3.621 | 0.377 | -1.562 |
| 7 | -4.399 | -3.214 | -1.479 |
| 7 | -6.453 | -2.369 | -1.623 |
| 7 | 2.133 | 5.120 | -1.717 |
| 7 | 0.000 | 5.966 | -1.705 |
| 7 | 0.377 | 3.621 | -1.562 |
| 7 | -3.214 | 4.399 | -1.479 |
| 7 | -2.369 | 6.453 | -1.623 |
| 6 | -0.810 | -4.921 | -1.660 |
| 6 | 0.956 | -3.225 | -1.493 |
| 6 | 1.838 | -4.341 | -1.542 |
| 6 | 1.291 | -5.612 | -1.634 |
| 6 | 3.495 | -5.670 | -1.529 |
| 6 | 4.921 | -0.810 | -1.660 |
| 6 | 3.225 | 0.956 | -1.493 |
| 6 | 4.341 | 1.838 | -1.541 |
| 6 | 5.612 | 1.291 | -1.634 |
| 6 | 5.670 | 3.495 | -1.528 |
| 6 | -4.921 | 0.810 | -1.660 |
| 6 | -3.225 | -0.956 | -1.493 |
| 6 | -4.341 | -1.838 | -1.541 |
| 6 | -5.612 | -1.291 | -1.634 |
| 6 | -5.670 | -3.495 | -1.528 |
| 6 | 0.810 | 4.921 | -1.660 |
| 6 | -0.956 | 3.225 | -1.493 |
| 6 | -1.838 | 4.341 | -1.542 |
| 6 | -1.291 | 5.612 | -1.634 |
| 6 | -3.495 | 5.670 | -1.529 |
| 1 | -2.424 | -6.078 | -1.784 |
| 1 | -2.833 | -4.386 | -1.615 |
| 1 | 4.485 | -6.096 | -1.499 |
| 1 | -1.062 | -2.858 | -1.532 |
| 1 | 6.078 | -2.424 | -1.784 |
| 1 | 4.386 | -2.833 | -1.615 |
| 1 | 6.096 | 4.485 | -1.499 |
| 1 | 2.858 | -1.062 | -1.532 |
| 1 | -6.078 | 2.424 | -1.784 |
| 1 | -4.386 | 2.833 | -1.615 |
| 1 | -6.096 | -4.485 | -1.499 |
| 1 | -2.858 | 1.062 | -1.532 |
| 1 | 2.424 | 6.078 | -1.784 |
| 1 | 2.833 | 4.386 | -1.615 |
| 1 | -4.485 | 6.096 | -1.499 |
| 1 | 1.062 | 2.858 | -1.532 |
| 1 | 7.455 | 2.329 | -1.705 |
| 1 | -2.329 | 7.455 | -1.705 |
| 1 | -7.455 | -2.329 | -1.705 |
| 1 | 2.329 | -7.455 | -1.705 |

Cartesian coordinates of optimized structures in the water phase (COSMO) by M05-2X/6-31G(d,p)// M05-2X/6-31G(d,p).

A-A

| 7 | 3.029 | -1.359 | 0.000 |
| --- | --- | --- | --- |
| 6 | 2.581 | -0.088 | 0.000 |
| 6 | 1.183 | 0.123 | 0.000 |
| 6 | 0.385 | -1.017 | 0.000 |
| 7 | 0.808 | -2.292 | 0.000 |
| 6 | 2.135 | -2.358 | 0.000 |
| 7 | -0.898 | -0.544 | 0.000 |
| 6 | -0.818 | 0.822 | 0.000 |
| 7 | 0.414 | 1.269 | 0.000 |
| 7 | 3.461 | 0.917 | 0.000 |
| 1 | 2.561 | -3.356 | 0.000 |
| 1 | -1.706 | 1.434 | 0.000 |
| 1 | 4.461 | 0.720 | 0.000 |
| 1 | 3.130 | 1.866 | 0.000 |
| 1 | -1.744 | -1.094 | 0.000 |
| 7 | 6.406 | 0.129 | 0.000 |
| 6 | 7.490 | 0.926 | 0.000 |
| 6 | 8.761 | 0.313 | 0.000 |
| 6 | 8.776 | -1.079 | 0.000 |
| 7 | 7.707 | -1.892 | 0.000 |
| 6 | 6.574 | -1.204 | 0.000 |
| 7 | 10.101 | -1.412 | 0.000 |
| 6 | 10.807 | -0.240 | 0.000 |
| 7 | 10.043 | 0.825 | 0.000 |
| 7 | 7.331 | 2.254 | 0.000 |
| 1 | 5.651 | -1.773 | 0.000 |
| 1 | 11.886 | -0.237 | 0.000 |
| 1 | 6.406 | 2.650 | 0.000 |
| 1 | 8.131 | 2.862 | 0.000 |
| 1 | 10.489 | -2.344 | 0.000 |

A-T

| 7 | -1.496 | 1.316 | 0.000 |
| --- | --- | --- | --- |
| 6 | -2.570 | 0.511 | 0.000 |
| 7 | -3.850 | 0.849 | 0.000 |
| 6 | -4.004 | 2.185 | 0.000 |
| 6 | -2.992 | 3.140 | 0.000 |
| 6 | -1.666 | 2.653 | 0.000 |
| 7 | -5.164 | 2.907 | 0.000 |
| 6 | -4.810 | 4.228 | 0.000 |
| 7 | -3.514 | 4.418 | 0.000 |
| 7 | -0.594 | 3.447 | 0.000 |
| 1 | -2.341 | -0.550 | 0.000 |
| 1 | -6.104 | 2.539 | 0.000 |
| 1 | -5.556 | 5.007 | 0.000 |
| 1 | -0.713 | 4.446 | 0.000 |
| 1 | 0.337 | 3.042 | 0.000 |
| 7 | 1.116 | 0.088 | 0.000 |
| 6 | 1.104 | -1.284 | 0.000 |
| 7 | 2.350 | -1.855 | 0.000 |
| 6 | 3.510 | -1.125 | 0.000 |
| 6 | 3.516 | 0.225 | 0.000 |
| 6 | 2.230 | 0.902 | 0.000 |
| 8 | 2.103 | 2.131 | 0.000 |
| 8 | 0.068 | -1.940 | 0.000 |
| 6 | 4.762 | 1.057 | 0.000 |
| 1 | 0.186 | 0.547 | 0.000 |
| 1 | 2.385 | -2.864 | 0.000 |
| 1 | 4.421 | -1.709 | 0.000 |
| 1 | 5.646 | 0.421 | 0.000 |
| 1 | 4.792 | 1.703 | 0.880 |
| 1 | 4.792 | 1.703 | -0.880 |

A-G

| 7 | 5.791 | 0.777 | 0.000 |
| --- | --- | --- | --- |
| 6 | 5.095 | 1.935 | 0.000 |
| 6 | 3.684 | 1.856 | 0.000 |
| 6 | 3.130 | 0.581 | 0.000 |
| 7 | 3.803 | -0.582 | 0.000 |
| 6 | 5.113 | -0.383 | 0.000 |
| 7 | 1.779 | 0.787 | 0.000 |
| 6 | 1.584 | 2.141 | 0.000 |
| 7 | 2.702 | 2.825 | 0.000 |
| 7 | 5.746 | 3.098 | 0.000 |
| 1 | 5.724 | -1.279 | 0.000 |
| 1 | 0.591 | 2.563 | 0.000 |
| 1 | 6.763 | 3.119 | 0.000 |
| 1 | 5.221 | 3.956 | 0.000 |
| 1 | 1.061 | 0.079 | 0.000 |
| 6 | 12.893 | 2.679 | 0.000 |
| 7 | 12.810 | 1.308 | 0.000 |
| 1 | 13.583 | 0.660 | 0.000 |
| 6 | 11.486 | 0.988 | 0.000 |
| 6 | 10.825 | 2.209 | 0.000 |
| 7 | 11.722 | 3.260 | 0.000 |
| 6 | 9.400 | 2.206 | 0.000 |
| 8 | 8.646 | 3.187 | 0.000 |
| 7 | 8.882 | 0.910 | 0.000 |
| 1 | 7.856 | 0.837 | 0.000 |
| 6 | 9.635 | -0.239 | 0.000 |
| 7 | 10.955 | -0.252 | 0.000 |
| 7 | 8.967 | -1.402 | 0.000 |
| 1 | 13.847 | 3.183 | 0.000 |
| 1 | 7.965 | -1.446 | 0.000 |
| 1 | 9.497 | -2.256 | 0.000 |

A-C

| 7 | 1.732 | -0.104 | 0.000 |
| --- | --- | --- | --- |
| 6 | 0.750 | -1.023 | 0.000 |
| 6 | -0.585 | -0.566 | 0.000 |
| 6 | -0.765 | 0.814 | 0.000 |
| 7 | 0.200 | 1.749 | 0.000 |
| 6 | 1.407 | 1.200 | 0.000 |
| 7 | -2.121 | 0.988 | 0.000 |
| 6 | -2.682 | -0.260 | 0.000 |
| 7 | -1.797 | -1.226 | 0.000 |
| 7 | 1.064 | -2.324 | 0.000 |
| 1 | 2.257 | 1.874 | 0.000 |
| 1 | -3.753 | -0.392 | 0.000 |
| 1 | 2.029 | -2.608 | 0.000 |
| 1 | 0.341 | -3.022 | 0.000 |
| 1 | -2.616 | 1.867 | 0.000 |
| 6 | 6.925 | 0.443 | 0.000 |
| 6 | 7.596 | 1.617 | 0.000 |
| 7 | 6.916 | 2.790 | 0.000 |
| 6 | 5.523 | 2.857 | 0.000 |
| 8 | 4.982 | 3.967 | 0.000 |
| 7 | 4.835 | 1.687 | 0.000 |
| 6 | 5.490 | 0.527 | 0.000 |
| 7 | 4.762 | -0.593 | 0.000 |
| 1 | 7.436 | -0.508 | 0.000 |
| 1 | 8.675 | 1.688 | 0.000 |
| 1 | 3.746 | -0.525 | 0.000 |
| 1 | 5.210 | -1.493 | 0.000 |
| 1 | 7.411 | 3.670 | 0.000 |

T-T

| 6 | -1.162 | -2.952 | 0.000 |
| --- | --- | --- | --- |
| 6 | -0.113 | -1.882 | 0.000 |
| 6 | -0.385 | -0.562 | 0.000 |
| 7 | 0.603 | 0.391 | 0.000 |
| 1 | 0.364 | 1.372 | 0.000 |
| 6 | 1.933 | 0.087 | 0.000 |
| 8 | 2.808 | 0.956 | 0.000 |
| 7 | 2.210 | -1.252 | 0.000 |
| 1 | 3.203 | -1.511 | 0.000 |
| 6 | 1.285 | -2.290 | 0.000 |
| 8 | 1.669 | -3.455 | 0.000 |
| 1 | -1.395 | -0.175 | 0.000 |
| 6 | 7.739 | -2.842 | 0.000 |
| 6 | 7.349 | -1.396 | 0.000 |
| 6 | 8.232 | -0.375 | 0.000 |
| 7 | 7.839 | 0.938 | 0.000 |
| 1 | 8.528 | 1.675 | 0.000 |
| 6 | 6.526 | 1.332 | 0.000 |
| 8 | 6.177 | 2.504 | 0.000 |
| 7 | 5.631 | 0.286 | 0.000 |
| 1 | 4.640 | 0.546 | 0.000 |
| 6 | 5.936 | -1.060 | 0.000 |
| 8 | 5.034 | -1.905 | 0.000 |
| 1 | 9.302 | -0.534 | 0.000 |
| 1 | -2.158 | -2.510 | 0.000 |
| 1 | -1.059 | -3.590 | 0.880 |
| 1 | -1.059 | -3.590 | -0.880 |
| 1 | 8.823 | -2.944 | 0.000 |
| 1 | 7.337 | -3.348 | -0.880 |
| 1 | 7.337 | -3.348 | 0.880 |

G-T

| 6 | -0.377 | 4.667 | 0.000 |
| --- | --- | --- | --- |
| 7 | 0.277 | 3.460 | 0.000 |
| 1 | 1.275 | 3.315 | 0.000 |
| 6 | -0.679 | 2.488 | 0.000 |
| 6 | -1.887 | 3.179 | 0.000 |
| 7 | -1.677 | 4.546 | 0.000 |
| 6 | -3.091 | 2.419 | 0.000 |
| 8 | -4.260 | 2.830 | 0.000 |
| 7 | -2.834 | 1.050 | 0.000 |
| 1 | -3.660 | 0.438 | 0.000 |
| 6 | -1.595 | 0.467 | 0.000 |
| 7 | -0.467 | 1.154 | 0.000 |
| 7 | -1.569 | -0.875 | 0.000 |
| 1 | 0.168 | 5.598 | 0.000 |
| 1 | -2.414 | -1.419 | 0.000 |
| 1 | -0.679 | -1.344 | 0.000 |
| 6 | -10.403 | 1.342 | 0.000 |
| 6 | -9.016 | 0.779 | 0.000 |
| 6 | -8.733 | -0.539 | 0.000 |
| 7 | -7.443 | -1.013 | 0.000 |
| 1 | -7.268 | -2.007 | 0.000 |
| 6 | -6.350 | -0.197 | 0.000 |
| 8 | -5.198 | -0.644 | 0.000 |
| 7 | -6.632 | 1.139 | 0.000 |
| 1 | -5.829 | 1.788 | 0.000 |
| 6 | -7.898 | 1.715 | 0.000 |
| 8 | -8.016 | 2.935 | 0.000 |
| 1 | -9.501 | -1.300 | 0.000 |
| 1 | -11.142 | 0.542 | 0.000 |
| 1 | -10.561 | 1.969 | -0.879 |
| 1 | -10.561 | 1.969 | 0.879 |

C-T

| 6 | 7.176 | 4.076 | 0.000 |
| --- | --- | --- | --- |
| 6 | 6.525 | 2.727 | 0.000 |
| 6 | 7.203 | 1.561 | 0.000 |
| 7 | 6.568 | 0.348 | 0.000 |
| 1 | 7.105 | -0.507 | 0.000 |
| 6 | 5.205 | 0.205 | 0.000 |
| 8 | 4.655 | -0.887 | 0.000 |
| 7 | 4.515 | 1.397 | 0.000 |
| 1 | 3.487 | 1.335 | 0.000 |
| 6 | 5.073 | 2.662 | 0.000 |
| 8 | 4.354 | 3.665 | 0.000 |
| 1 | 8.284 | 1.516 | 0.000 |
| 6 | -0.686 | 2.642 | 0.000 |
| 6 | -1.332 | 1.455 | 0.000 |
| 7 | -0.626 | 0.299 | 0.000 |
| 6 | 0.767 | 0.259 | 0.000 |
| 8 | 1.325 | -0.841 | 0.000 |
| 7 | 1.434 | 1.445 | 0.000 |
| 6 | 0.750 | 2.593 | 0.000 |
| 7 | 1.440 | 3.732 | 0.000 |
| 1 | -1.215 | 3.583 | 0.000 |
| 1 | -2.409 | 1.361 | 0.000 |
| 1 | 2.456 | 3.709 | 0.000 |
| 1 | 0.960 | 4.615 | 0.000 |
| 1 | -1.099 | -0.592 | 0.000 |
| 1 | 8.260 | 3.975 | 0.000 |
| 1 | 6.875 | 4.648 | 0.880 |
| 1 | 6.875 | 4.648 | -0.880 |

G-G

| 6 | 7.167 | -0.095 | 0.262 |
| --- | --- | --- | --- |
| 7 | 5.931 | -0.683 | 0.262 |
| 1 | 5.043 | -0.205 | 0.262 |
| 6 | 6.124 | -2.034 | 0.262 |
| 6 | 7.503 | -2.194 | 0.262 |
| 7 | 8.140 | -0.970 | 0.262 |
| 6 | 8.034 | -3.497 | 0.262 |
| 8 | 9.219 | -3.867 | 0.262 |
| 7 | 7.023 | -4.470 | 0.262 |
| 1 | 7.335 | -5.434 | 0.262 |
| 6 | 5.671 | -4.208 | 0.262 |
| 7 | 5.170 | -2.988 | 0.262 |
| 7 | 4.853 | -5.268 | 0.262 |
| 1 | 7.283 | 0.977 | 0.262 |
| 1 | 5.195 | -6.213 | 0.262 |
| 1 | 3.861 | -5.104 | 0.262 |
| 6 | 16.262 | -2.829 | 0.262 |
| 7 | 15.693 | -1.580 | 0.262 |
| 1 | 16.182 | -0.697 | 0.262 |
| 6 | 14.339 | -1.755 | 0.262 |
| 6 | 14.161 | -3.132 | 0.262 |
| 7 | 15.377 | -3.790 | 0.262 |
| 6 | 12.825 | -3.643 | 0.262 |
| 8 | 12.460 | -4.819 | 0.262 |
| 7 | 11.882 | -2.608 | 0.262 |
| 1 | 10.898 | -2.925 | 0.262 |
| 6 | 12.168 | -1.264 | 0.262 |
| 7 | 13.404 | -0.783 | 0.262 |
| 7 | 11.133 | -0.416 | 0.262 |
| 1 | 17.334 | -2.956 | 0.262 |
| 1 | 10.140 | -0.702 | 0.262 |
| 1 | 11.361 | 0.564 | 0.262 |

G-C

| 7 | -2.607 | 0.018 | 0.033 |
| --- | --- | --- | --- |
| 8 | 1.713 | 1.556 | 0.033 |
| 7 | -0.471 | 0.878 | 0.033 |
| 6 | -1.827 | 1.104 | 0.033 |
| 7 | -2.365 | 2.312 | 0.033 |
| 6 | -1.437 | 3.291 | 0.033 |
| 6 | -0.052 | 3.172 | 0.033 |
| 6 | 0.512 | 1.862 | 0.033 |
| 7 | -1.670 | 4.634 | 0.033 |
| 6 | -0.446 | 5.257 | 0.033 |
| 7 | 0.553 | 4.415 | 0.033 |
| 1 | -2.228 | -0.928 | 0.033 |
| 1 | -3.602 | 0.161 | 0.033 |
| 1 | -0.136 | -0.099 | 0.033 |
| 1 | -2.573 | 5.084 | 0.033 |
| 1 | -0.365 | 6.333 | 0.033 |
| 8 | -1.630 | -2.702 | 0.033 |
| 7 | 2.691 | -1.214 | 0.033 |
| 7 | 0.517 | -1.922 | 0.033 |
| 6 | 2.297 | -3.576 | 0.033 |
| 6 | -0.407 | -2.912 | 0.033 |
| 7 | 0.040 | -4.224 | 0.033 |
| 6 | 1.357 | -4.548 | 0.033 |
| 6 | 1.822 | -2.221 | 0.033 |
| 1 | 3.352 | -3.800 | 0.033 |
| 1 | -0.667 | -4.945 | 0.033 |
| 1 | 1.590 | -5.604 | 0.033 |
| 1 | 2.359 | -0.252 | 0.033 |
| 1 | 3.680 | -1.399 | 0.033 |

C-C

| 6 | 1.438 | 3.083 | 0.000 |
| --- | --- | --- | --- |
| 6 | 2.031 | 1.868 | 0.000 |
| 7 | 1.277 | 0.740 | 0.000 |
| 6 | -0.118 | 0.762 | 0.000 |
| 8 | -0.725 | -0.315 | 0.000 |
| 7 | -0.734 | 1.972 | 0.000 |
| 6 | 0.000 | 3.084 | 0.000 |
| 7 | -0.640 | 4.257 | 0.000 |
| 1 | 1.997 | 4.008 | 0.000 |
| 1 | 3.103 | 1.726 | 0.000 |
| 1 | -1.646 | 4.253 | 0.000 |
| 1 | -0.144 | 5.145 | 0.000 |
| 6 | 0.443 | 9.477 | 0.000 |
| 6 | 1.793 | 9.562 | 0.000 |
| 7 | 2.546 | 8.435 | 0.000 |
| 6 | 1.995 | 7.158 | 0.000 |
| 8 | 2.755 | 6.183 | 0.000 |
| 7 | 0.640 | 7.053 | 0.000 |
| 6 | -0.112 | 8.154 | 0.000 |
| 7 | -1.440 | 8.006 | 0.000 |
| 1 | -0.187 | 10.353 | 0.000 |
| 1 | 2.332 | 10.499 | 0.000 |
| 1 | -1.846 | 7.086 | 0.000 |
| 1 | -2.050 | 8.806 | 0.000 |
| 1 | 1.714 | -0.169 | 0.000 |
| 1 | 3.554 | 8.493 | 0.000 |

A-T/A-T

| 8 | 2.815 | -1.247 | 2.037 |
| --- | --- | --- | --- |
| 8 | 1.426 | 2.807 | 0.508 |
| 7 | 4.388 | 0.332 | 1.533 |
| 7 | 2.149 | 0.804 | 1.287 |
| 7 | -1.419 | 2.101 | 1.292 |
| 7 | -4.175 | 0.787 | 1.640 |
| 7 | -4.297 | -1.412 | 2.035 |
| 7 | -1.920 | -1.967 | 2.056 |
| 7 | -0.568 | -0.024 | 1.664 |
| 6 | 4.079 | 3.841 | 0.156 |
| 6 | 2.376 | 2.076 | 0.804 |
| 6 | 3.770 | 2.474 | 0.685 |
| 6 | 4.708 | 1.581 | 1.065 |
| 6 | 3.099 | -0.118 | 1.647 |
| 6 | -4.968 | -0.236 | 1.843 |
| 6 | -1.625 | 0.801 | 1.539 |
| 6 | -2.910 | 0.241 | 1.699 |
| 6 | -2.964 | -1.128 | 1.944 |
| 6 | -0.769 | -1.329 | 1.907 |
| 1 | 5.767 | 1.796 | 1.018 |
| 1 | 5.119 | -0.318 | 1.784 |
| 1 | 1.160 | 0.505 | 1.397 |
| 1 | -0.488 | 2.401 | 1.020 |
| 1 | -2.208 | 2.673 | 1.040 |
| 1 | -6.046 | -0.196 | 1.866 |
| 1 | -4.712 | -2.317 | 2.200 |
| 1 | 0.135 | -1.922 | 1.982 |
| 1 | 3.618 | 4.608 | 0.780 |
| 1 | 3.683 | 3.958 | -0.854 |
| 1 | 5.156 | 4.005 | 0.132 |
| 8 | 0.676 | -3.477 | -0.194 |
| 8 | 2.129 | 0.421 | -2.046 |
| 7 | 2.915 | -3.193 | -0.539 |
| 7 | 1.432 | -1.530 | -1.118 |
| 7 | -0.649 | 1.486 | -1.908 |
| 7 | -3.643 | 2.179 | -1.851 |
| 7 | -5.139 | 0.535 | -1.590 |
| 7 | -3.631 | -1.370 | -1.352 |
| 7 | -1.333 | -0.693 | -1.526 |
| 6 | 4.892 | -0.337 | -2.031 |
| 6 | 2.420 | -0.691 | -1.591 |
| 6 | 3.780 | -1.197 | -1.512 |
| 6 | 3.959 | -2.426 | -0.981 |
| 6 | 1.609 | -2.784 | -0.586 |
| 6 | -4.915 | 1.872 | -1.768 |
| 6 | -1.635 | 0.607 | -1.719 |
| 6 | -3.001 | 0.966 | -1.713 |
| 6 | -3.915 | -0.069 | -1.539 |
| 6 | -2.323 | -1.582 | -1.350 |
| 1 | 4.937 | -2.877 | -0.882 |
| 1 | 3.087 | -4.104 | -0.139 |
| 1 | 0.449 | -1.218 | -1.219 |
| 1 | 0.318 | 1.176 | -1.914 |
| 1 | -0.866 | 2.462 | -2.016 |
| 1 | -5.733 | 2.573 | -1.832 |
| 1 | -6.037 | 0.089 | -1.480 |
| 1 | -1.995 | -2.602 | -1.182 |
| 1 | 4.877 | 0.641 | -1.549 |
| 1 | 4.782 | -0.174 | -3.105 |
| 1 | 5.856 | -0.809 | -1.846 |

A-T/T-A

| 8 | -2.372 | -2.276 | -1.618 |
| --- | --- | --- | --- |
| 8 | -0.939 | 2.050 | -1.717 |
| 7 | -3.922 | -0.606 | -1.765 |
| 7 | -1.680 | -0.093 | -1.696 |
| 7 | 1.870 | 1.107 | -1.885 |
| 7 | 4.639 | -0.165 | -1.438 |
| 7 | 4.765 | -2.321 | -0.854 |
| 7 | 2.395 | -2.896 | -0.848 |
| 7 | 1.033 | -0.994 | -1.386 |
| 6 | -3.588 | 3.160 | -1.747 |
| 6 | -1.897 | 1.269 | -1.723 |
| 6 | -3.286 | 1.692 | -1.762 |
| 6 | -4.232 | 0.729 | -1.780 |
| 6 | -2.637 | -1.079 | -1.686 |
| 6 | 5.433 | -1.158 | -1.125 |
| 6 | 2.086 | -0.169 | -1.550 |
| 6 | 3.375 | -0.714 | -1.364 |
| 6 | 3.434 | -2.059 | -1.006 |
| 6 | 1.242 | -2.278 | -1.056 |
| 1 | -5.289 | 0.956 | -1.805 |
| 1 | -4.657 | -1.298 | -1.749 |
| 1 | -0.699 | -0.412 | -1.612 |
| 1 | 0.922 | 1.470 | -1.876 |
| 1 | 2.648 | 1.743 | -1.928 |
| 1 | 6.510 | -1.108 | -1.076 |
| 1 | 5.181 | -3.205 | -0.599 |
| 1 | 0.341 | -2.873 | -0.952 |
| 1 | -2.975 | 3.681 | -2.483 |
| 1 | -3.363 | 3.590 | -0.767 |
| 1 | -4.639 | 3.337 | -1.971 |
| 8 | 2.359 | -1.510 | 2.225 |
| 8 | 0.954 | 2.655 | 1.048 |
| 7 | 3.926 | 0.089 | 1.774 |
| 7 | 1.684 | 0.589 | 1.634 |
| 7 | -1.910 | 1.782 | 1.522 |
| 7 | -4.655 | 0.396 | 1.421 |
| 7 | -4.751 | -1.841 | 1.460 |
| 7 | -2.373 | -2.360 | 1.641 |
| 7 | -1.042 | -0.362 | 1.641 |
| 6 | 3.593 | 3.684 | 0.646 |
| 6 | 1.906 | 1.895 | 1.249 |
| 6 | 3.298 | 2.289 | 1.102 |
| 6 | 4.241 | 1.362 | 1.370 |
| 6 | 2.639 | -0.361 | 1.900 |
| 6 | -5.435 | -0.657 | 1.388 |
| 6 | -2.106 | 0.460 | 1.557 |
| 6 | -3.386 | -0.135 | 1.515 |
| 6 | -3.424 | -1.528 | 1.547 |
| 6 | -1.231 | -1.691 | 1.681 |
| 1 | 5.300 | 1.568 | 1.282 |
| 1 | 4.659 | -0.583 | 1.948 |
| 1 | 0.699 | 0.272 | 1.689 |
| 1 | -0.968 | 2.143 | 1.413 |
| 1 | -2.698 | 2.393 | 1.385 |
| 1 | -6.511 | -0.637 | 1.314 |
| 1 | -5.153 | -2.766 | 1.450 |
| 1 | -0.321 | -2.278 | 1.757 |
| 1 | 3.169 | 4.415 | 1.337 |
| 1 | 4.668 | 3.845 | 0.578 |
| 1 | 3.147 | 3.864 | -0.335 |

G-C/A-T

| 8 | -3.067 | 1.193 | 2.024 |
| --- | --- | --- | --- |
| 8 | -1.720 | -2.826 | 0.369 |
| 7 | -4.653 | -0.276 | 1.286 |
| 7 | -2.424 | -0.844 | 1.216 |
| 7 | 1.115 | -2.246 | 1.274 |
| 7 | 3.899 | -1.016 | 1.725 |
| 7 | 4.065 | 1.156 | 2.240 |
| 7 | 1.702 | 1.767 | 2.243 |
| 7 | 0.312 | -0.117 | 1.717 |
| 6 | -4.364 | -3.676 | -0.343 |
| 6 | -2.660 | -2.066 | 0.622 |
| 6 | -4.050 | -2.375 | 0.329 |
| 6 | -4.980 | -1.462 | 0.682 |
| 6 | -3.361 | 0.104 | 1.541 |
| 6 | 4.712 | -0.025 | 1.997 |
| 6 | 1.351 | -0.963 | 1.579 |
| 6 | 2.646 | -0.444 | 1.788 |
| 6 | 2.728 | 0.910 | 2.106 |
| 6 | 0.540 | 1.168 | 2.032 |
| 1 | -6.037 | -1.613 | 0.508 |
| 1 | -5.372 | 0.399 | 1.501 |
| 1 | -1.434 | -0.590 | 1.401 |
| 1 | 0.185 | -2.501 | 0.955 |
| 1 | 1.893 | -2.822 | 0.998 |
| 1 | 5.788 | -0.092 | 2.038 |
| 1 | 4.497 | 2.041 | 2.459 |
| 1 | -0.351 | 1.781 | 2.113 |
| 1 | -4.014 | -4.516 | 0.259 |
| 1 | -3.861 | -3.736 | -1.311 |
| 1 | -5.438 | -3.776 | -0.498 |
| 7 | 1.874 | 2.803 | -1.018 |
| 8 | 0.481 | -1.490 | -1.840 |
| 7 | 1.271 | 0.610 | -1.405 |
| 6 | 2.270 | 1.528 | -1.204 |
| 7 | 3.555 | 1.225 | -1.227 |
| 6 | 3.771 | -0.088 | -1.452 |
| 6 | 2.836 | -1.097 | -1.658 |
| 6 | 1.455 | -0.746 | -1.655 |
| 7 | 4.979 | -0.716 | -1.514 |
| 6 | 4.731 | -2.048 | -1.730 |
| 7 | 3.456 | -2.320 | -1.826 |
| 1 | 0.915 | 2.993 | -0.732 |
| 1 | 2.588 | 3.432 | -0.689 |
| 1 | 0.288 | 0.923 | -1.341 |
| 1 | 5.883 | -0.285 | -1.393 |
| 1 | 5.536 | -2.762 | -1.810 |
| 8 | -0.880 | 3.401 | -0.211 |
| 7 | -2.259 | -0.495 | -2.160 |
| 7 | -1.539 | 1.442 | -1.183 |
| 6 | -3.898 | 1.194 | -1.701 |
| 6 | -1.773 | 2.678 | -0.681 |
| 7 | -3.074 | 3.154 | -0.702 |
| 6 | -4.110 | 2.429 | -1.192 |
| 6 | -2.546 | 0.713 | -1.678 |
| 1 | -4.702 | 0.591 | -2.095 |
| 1 | -3.229 | 4.075 | -0.318 |
| 1 | -5.083 | 2.897 | -1.151 |
| 1 | -1.312 | -0.861 | -2.075 |
| 1 | -2.993 | -1.088 | -2.508 |

G-C/T-A

| 8 | -1.871 | -1.166 | -2.552 |
| --- | --- | --- | --- |
| 8 | -0.766 | 2.813 | -0.647 |
| 7 | -3.542 | 0.321 | -2.084 |
| 7 | -1.352 | 0.848 | -1.615 |
| 7 | 2.127 | 2.110 | -0.960 |
| 7 | 4.929 | 0.832 | -0.905 |
| 7 | 5.150 | -1.330 | -1.437 |
| 7 | 2.830 | -1.876 | -1.960 |
| 7 | 1.395 | 0.021 | -1.640 |
| 6 | -3.492 | 3.675 | -0.342 |
| 6 | -1.665 | 2.072 | -1.063 |
| 6 | -3.078 | 2.404 | -1.019 |
| 6 | -3.947 | 1.512 | -1.537 |
| 6 | -2.232 | -0.078 | -2.113 |
| 6 | 5.760 | -0.173 | -1.036 |
| 6 | 2.399 | 0.838 | -1.266 |
| 6 | 3.703 | 0.294 | -1.239 |
| 6 | 3.820 | -1.052 | -1.578 |
| 6 | 1.660 | -1.256 | -1.960 |
| 1 | -5.016 | 1.677 | -1.538 |
| 1 | -4.224 | -0.340 | -2.424 |
| 1 | -0.301 | 0.548 | -1.630 |
| 1 | 1.158 | 2.401 | -0.861 |
| 1 | 2.856 | 2.688 | -0.578 |
| 1 | 6.824 | -0.132 | -0.862 |
| 1 | 5.601 | -2.218 | -1.604 |
| 1 | 0.794 | -1.839 | -2.255 |
| 1 | -2.957 | 4.528 | -0.755 |
| 1 | -3.259 | 3.620 | 0.722 |
| 1 | -4.563 | 3.836 | -0.454 |
| 7 | -0.198 | -2.980 | 0.498 |
| 8 | -1.980 | 0.982 | 1.964 |
| 7 | -1.184 | -1.032 | 1.232 |
| 6 | -1.330 | -2.296 | 0.716 |
| 7 | -2.503 | -2.833 | 0.422 |
| 6 | -3.530 | -1.996 | 0.672 |
| 6 | -3.493 | -0.709 | 1.200 |
| 6 | -2.221 | -0.147 | 1.510 |
| 7 | -4.852 | -2.240 | 0.448 |
| 6 | -5.542 | -1.119 | 0.841 |
| 7 | -4.764 | -0.176 | 1.300 |
| 1 | 0.707 | -2.631 | 0.810 |
| 1 | -0.287 | -3.932 | 0.188 |
| 1 | -0.233 | -0.685 | 1.433 |
| 1 | -5.249 | -3.086 | 0.069 |
| 1 | -6.617 | -1.067 | 0.762 |
| 8 | 2.412 | -2.041 | 1.342 |
| 7 | 0.709 | 2.077 | 2.341 |
| 7 | 1.528 | 0.007 | 1.834 |
| 6 | 3.088 | 1.785 | 2.390 |
| 6 | 2.561 | -0.850 | 1.661 |
| 7 | 3.847 | -0.373 | 1.854 |
| 6 | 4.104 | 0.913 | 2.203 |
| 6 | 1.760 | 1.278 | 2.184 |
| 1 | 3.259 | 2.812 | 2.670 |
| 1 | 4.603 | -1.024 | 1.701 |
| 1 | 5.146 | 1.177 | 2.315 |
| 1 | -0.230 | 1.714 | 2.190 |
| 1 | 0.836 | 3.039 | 2.605 |

G-C/G-C

| 7 | -0.434 | 1.179 | 2.660 |
| --- | --- | --- | --- |
| 8 | 1.344 | -2.518 | 0.609 |
| 7 | 0.545 | -0.645 | 1.649 |
| 6 | 0.695 | 0.558 | 2.296 |
| 7 | 1.870 | 1.095 | 2.576 |
| 6 | 2.899 | 0.332 | 2.156 |
| 6 | 2.858 | -0.895 | 1.502 |
| 6 | 1.583 | -1.454 | 1.199 |
| 7 | 4.227 | 0.611 | 2.286 |
| 6 | 4.917 | -0.431 | 1.717 |
| 7 | 4.132 | -1.358 | 1.234 |
| 1 | -1.355 | 0.835 | 2.390 |
| 1 | -0.346 | 2.109 | 3.032 |
| 1 | -0.411 | -0.966 | 1.429 |
| 1 | 4.625 | 1.424 | 2.731 |
| 1 | 5.996 | -0.443 | 1.695 |
| 8 | -3.110 | 0.272 | 2.032 |
| 7 | -1.362 | -3.310 | -0.206 |
| 7 | -2.200 | -1.521 | 0.943 |
| 6 | -3.740 | -3.044 | -0.158 |
| 6 | -3.244 | -0.770 | 1.369 |
| 7 | -4.525 | -1.190 | 1.050 |
| 6 | -4.767 | -2.291 | 0.296 |
| 6 | -2.419 | -2.614 | 0.202 |
| 1 | -3.899 | -3.927 | -0.758 |
| 1 | -5.285 | -0.606 | 1.366 |
| 1 | -5.806 | -2.513 | 0.094 |
| 1 | -0.422 | -3.015 | 0.051 |
| 1 | -1.487 | -4.137 | -0.765 |
| 7 | 1.215 | 2.996 | -0.320 |
| 8 | 0.352 | -1.075 | -2.244 |
| 7 | 0.879 | 0.928 | -1.278 |
| 6 | 1.760 | 1.894 | -0.856 |
| 7 | 3.072 | 1.791 | -0.984 |
| 6 | 3.444 | 0.635 | -1.572 |
| 6 | 2.640 | -0.405 | -2.025 |
| 6 | 1.228 | -0.273 | -1.887 |
| 7 | 4.717 | 0.237 | -1.854 |
| 6 | 4.633 | -1.001 | -2.440 |
| 7 | 3.402 | -1.423 | -2.564 |
| 1 | 0.224 | 3.041 | -0.086 |
| 1 | 1.847 | 3.656 | 0.101 |
| 1 | -0.133 | 1.093 | -1.152 |
| 1 | 5.562 | 0.752 | -1.660 |
| 1 | 5.517 | -1.534 | -2.753 |
| 8 | -1.625 | 3.259 | 0.278 |
| 7 | -2.498 | -0.405 | -2.312 |
| 7 | -2.024 | 1.419 | -1.015 |
| 6 | -4.340 | 0.929 | -1.560 |
| 6 | -2.418 | 2.501 | -0.302 |
| 7 | -3.774 | 2.775 | -0.224 |
| 6 | -4.711 | 2.009 | -0.837 |
| 6 | -2.934 | 0.648 | -1.623 |
| 1 | -5.061 | 0.296 | -2.055 |
| 1 | -4.046 | 3.582 | 0.317 |
| 1 | -5.739 | 2.318 | -0.709 |
| 1 | -1.509 | -0.647 | -2.297 |
| 1 | -3.157 | -1.039 | -2.731 |

G-C/C-G

| 7 | 0.422 | -2.441 | -1.829 |
| --- | --- | --- | --- |
| 8 | 1.908 | 1.879 | -1.435 |
| 7 | 1.252 | -0.306 | -1.588 |
| 6 | 1.489 | -1.659 | -1.623 |
| 7 | 2.692 | -2.193 | -1.489 |
| 6 | 3.653 | -1.263 | -1.321 |
| 6 | 3.527 | 0.122 | -1.305 |
| 6 | 2.223 | 0.680 | -1.441 |
| 7 | 4.984 | -1.492 | -1.138 |
| 6 | 5.595 | -0.266 | -1.029 |
| 7 | 4.755 | 0.730 | -1.125 |
| 1 | -0.526 | -2.068 | -1.862 |
| 1 | 0.564 | -3.435 | -1.768 |
| 1 | 0.278 | 0.026 | -1.666 |
| 1 | 5.435 | -2.393 | -1.099 |
| 1 | 6.660 | -0.182 | -0.880 |
| 8 | -2.292 | -1.476 | -1.999 |
| 7 | -0.866 | 2.835 | -1.491 |
| 7 | -1.544 | 0.668 | -1.749 |
| 6 | -3.218 | 2.426 | -1.689 |
| 6 | -2.519 | -0.259 | -1.905 |
| 7 | -3.832 | 0.177 | -1.970 |
| 6 | -4.174 | 1.483 | -1.849 |
| 6 | -1.861 | 1.964 | -1.642 |
| 1 | -3.459 | 3.473 | -1.594 |
| 1 | -4.544 | -0.534 | -2.062 |
| 1 | -5.231 | 1.706 | -1.891 |
| 1 | 0.098 | 2.509 | -1.460 |
| 1 | -1.064 | 3.817 | -1.399 |
| 7 | -0.427 | -2.625 | 1.579 |
| 8 | -1.907 | 1.714 | 1.636 |
| 7 | -1.254 | -0.475 | 1.558 |
| 6 | -1.492 | -1.824 | 1.446 |
| 7 | -2.693 | -2.339 | 1.244 |
| 6 | -3.652 | -1.395 | 1.167 |
| 6 | -3.526 | -0.016 | 1.302 |
| 6 | -2.222 | 0.522 | 1.510 |
| 7 | -4.981 | -1.602 | 0.947 |
| 6 | -5.590 | -0.370 | 0.965 |
| 7 | -4.752 | 0.609 | 1.178 |
| 1 | 0.520 | -2.257 | 1.661 |
| 1 | -0.566 | -3.606 | 1.407 |
| 1 | -0.280 | -0.154 | 1.679 |
| 1 | -5.432 | -2.493 | 0.808 |
| 1 | -6.654 | -0.270 | 0.816 |
| 8 | 2.288 | -1.682 | 1.873 |
| 7 | 0.867 | 2.660 | 1.778 |
| 7 | 1.543 | 0.478 | 1.827 |
| 6 | 3.220 | 2.231 | 1.920 |
| 6 | 2.517 | -0.461 | 1.888 |
| 7 | 3.832 | -0.035 | 1.978 |
| 6 | 4.176 | 1.277 | 1.981 |
| 6 | 1.861 | 1.778 | 1.839 |
| 1 | 3.462 | 3.283 | 1.924 |
| 1 | 4.542 | -0.753 | 1.998 |
| 1 | 5.233 | 1.493 | 2.036 |
| 1 | -0.097 | 2.339 | 1.721 |
| 1 | 1.066 | 3.646 | 1.776 |

C^+^•G-C

| 7 | -2.714 | -0.041 | 0.000 |
| --- | --- | --- | --- |
| 8 | 1.656 | 1.342 | 0.000 |
| 7 | -0.546 | 0.739 | 0.000 |
| 6 | -1.895 | 1.013 | 0.000 |
| 7 | -2.391 | 2.242 | 0.000 |
| 6 | -1.435 | 3.186 | 0.000 |
| 6 | -0.056 | 3.013 | 0.000 |
| 6 | 0.460 | 1.690 | 0.000 |
| 7 | -1.617 | 4.539 | 0.000 |
| 6 | -0.381 | 5.122 | 0.000 |
| 7 | 0.582 | 4.237 | 0.000 |
| 1 | -2.370 | -1.001 | 0.000 |
| 1 | -3.703 | 0.141 | 0.000 |
| 1 | -0.239 | -0.249 | 0.000 |
| 1 | -2.504 | 5.022 | 0.000 |
| 1 | -0.243 | 6.191 | 0.000 |
| 8 | -1.824 | -2.774 | 0.000 |
| 7 | 2.558 | -1.472 | 0.000 |
| 7 | 0.355 | -2.085 | 0.000 |
| 6 | 2.062 | -3.814 | 0.000 |
| 6 | -0.610 | -3.035 | 0.000 |
| 7 | -0.220 | -4.364 | 0.000 |
| 6 | 1.082 | -4.745 | 0.000 |
| 6 | 1.646 | -2.440 | 0.000 |
| 1 | 3.107 | -4.084 | 0.000 |
| 1 | -0.958 | -5.053 | 0.000 |
| 1 | 1.269 | -5.809 | 0.000 |
| 1 | 2.267 | -0.499 | 0.000 |
| 1 | 3.538 | -1.699 | 0.000 |
| 6 | 5.605 | 4.744 | 0.000 |
| 6 | 5.702 | 6.093 | 0.000 |
| 7 | 4.600 | 6.886 | 0.000 |
| 6 | 3.316 | 6.392 | 0.000 |
| 8 | 2.326 | 7.103 | 0.000 |
| 7 | 3.234 | 5.014 | 0.000 |
| 1 | 2.244 | 4.644 | 0.000 |
| 6 | 4.296 | 4.175 | 0.000 |
| 7 | 4.087 | 2.878 | 0.000 |
| 1 | 6.476 | 4.108 | 0.000 |
| 1 | 6.651 | 6.611 | 0.000 |
| 1 | 3.153 | 2.462 | 0.000 |
| 1 | 4.882 | 2.257 | 0.000 |
| 1 | 4.695 | 7.893 | 0.000 |

rC^+^•G-C

| 7 | -2.707 | -0.056 | 0.000 |
| --- | --- | --- | --- |
| 8 | 1.595 | 1.513 | 0.000 |
| 7 | -0.576 | 0.819 | 0.000 |
| 6 | -1.936 | 1.033 | 0.000 |
| 7 | -2.484 | 2.239 | 0.000 |
| 6 | -1.569 | 3.224 | 0.000 |
| 6 | -0.183 | 3.113 | 0.000 |
| 6 | 0.384 | 1.813 | 0.000 |
| 7 | -1.811 | 4.567 | 0.000 |
| 6 | -0.598 | 5.201 | 0.000 |
| 7 | 0.405 | 4.363 | 0.000 |
| 1 | -2.322 | -1.001 | 0.000 |
| 1 | -3.703 | 0.081 | 0.000 |
| 1 | -0.225 | -0.155 | 0.000 |
| 1 | -2.717 | 5.011 | 0.000 |
| 1 | -0.520 | 6.276 | 0.000 |
| 8 | -1.704 | -2.749 | 0.000 |
| 7 | 2.618 | -1.259 | 0.000 |
| 7 | 0.443 | -1.965 | 0.000 |
| 6 | 2.224 | -3.619 | 0.000 |
| 6 | -0.479 | -2.956 | 0.000 |
| 7 | -0.032 | -4.268 | 0.000 |
| 6 | 1.286 | -4.591 | 0.000 |
| 6 | 1.749 | -2.264 | 0.000 |
| 1 | 3.280 | -3.842 | 0.000 |
| 1 | -0.739 | -4.988 | 0.000 |
| 1 | 1.519 | -5.647 | 0.000 |
| 1 | 2.294 | -0.297 | 0.000 |
| 1 | 3.609 | -1.430 | 0.000 |
| 6 | 5.585 | 4.717 | 0.000 |
| 6 | 6.554 | 3.774 | 0.000 |
| 7 | 6.255 | 2.449 | 0.000 |
| 6 | 4.970 | 1.964 | 0.000 |
| 8 | 4.706 | 0.775 | 0.000 |
| 7 | 3.990 | 2.941 | 0.000 |
| 1 | 3.024 | 2.571 | 0.000 |
| 6 | 4.227 | 4.277 | 0.000 |
| 7 | 3.214 | 5.109 | 0.000 |
| 1 | 5.814 | 5.771 | 0.000 |
| 1 | 7.606 | 4.018 | 0.000 |
| 1 | 2.230 | 4.797 | 0.000 |
| 1 | 3.404 | 6.100 | 0.000 |
| 1 | 6.994 | 1.758 | 0.000 |

T•A-T

| 8 | -0.307 | 5.077 | -0.035 |
| --- | --- | --- | --- |
| 8 | -4.680 | 6.329 | -0.011 |
| 8 | 2.139 | 0.742 | -0.087 |
| 8 | 0.105 | -3.323 | 0.147 |
| 7 | -2.477 | 5.734 | -0.023 |
| 7 | -3.076 | 7.954 | 0.016 |
| 7 | -1.457 | -0.044 | -0.027 |
| 7 | -3.810 | -0.524 | -0.013 |
| 7 | -5.136 | 1.527 | -0.029 |
| 7 | -3.501 | 3.037 | -0.049 |
| 7 | -0.561 | 2.091 | -0.053 |
| 7 | 1.152 | -1.297 | 0.027 |
| 7 | 2.387 | -3.237 | 0.130 |
| 6 | -1.124 | 6.002 | -0.018 |
| 6 | -3.497 | 6.651 | -0.006 |
| 6 | -1.753 | 8.316 | 0.024 |
| 6 | -0.754 | 7.408 | 0.008 |
| 6 | 0.701 | 7.765 | 0.018 |
| 6 | -2.527 | -0.854 | -0.014 |
| 6 | -3.969 | 0.811 | -0.026 |
| 6 | -2.961 | 1.768 | -0.039 |
| 6 | -1.628 | 1.295 | -0.040 |
| 6 | -4.798 | 2.847 | -0.043 |
| 6 | 1.141 | -2.668 | 0.105 |
| 6 | 3.547 | -2.509 | 0.081 |
| 6 | 3.553 | -1.161 | 0.005 |
| 6 | 2.266 | -0.484 | -0.023 |
| 6 | 4.798 | -0.330 | -0.049 |
| 1 | 1.197 | 7.335 | 0.890 |
| 1 | 1.199 | 7.370 | -0.870 |
| 1 | 0.828 | 8.847 | 0.039 |
| 1 | -1.568 | 9.382 | 0.043 |
| 1 | -2.771 | 4.743 | -0.038 |
| 1 | -3.796 | 8.661 | 0.028 |
| 1 | 5.682 | -0.966 | -0.019 |
| 1 | 4.824 | 0.265 | -0.963 |
| 1 | 4.834 | 0.363 | 0.794 |
| 1 | -2.294 | -1.913 | -0.001 |
| 1 | -6.073 | 1.151 | -0.022 |
| 1 | -5.538 | 3.631 | -0.047 |
| 1 | -0.654 | 3.100 | -0.057 |
| 1 | 0.363 | 1.670 | -0.060 |
| 1 | 0.223 | -0.837 | 0.006 |
| 1 | 2.423 | -4.244 | 0.189 |
| 1 | 4.457 | -3.093 | 0.109 |

rT•A-T

| 8 | 0.176 | -3.388 | 0.000 |
| --- | --- | --- | --- |
| 8 | 2.138 | 0.713 | 0.000 |
| 7 | 2.457 | -3.270 | 0.000 |
| 7 | 1.199 | -1.346 | 0.000 |
| 7 | -0.579 | 2.015 | 0.000 |
| 7 | -3.537 | 2.905 | 0.000 |
| 7 | -5.142 | 1.362 | 0.000 |
| 7 | -3.776 | -0.662 | 0.000 |
| 7 | -1.433 | -0.137 | 0.000 |
| 6 | 4.851 | -0.339 | 0.000 |
| 6 | 2.299 | -0.511 | 0.000 |
| 6 | 3.597 | -1.170 | 0.000 |
| 6 | 3.605 | -2.521 | 0.000 |
| 6 | 1.203 | -2.717 | 0.000 |
| 6 | -4.830 | 2.689 | 0.000 |
| 6 | -1.631 | 1.199 | 0.000 |
| 6 | -2.972 | 1.647 | 0.000 |
| 6 | -3.961 | 0.670 | 0.000 |
| 6 | -2.487 | -0.967 | 0.000 |
| 1 | 4.522 | -3.094 | 0.000 |
| 1 | 2.509 | -4.278 | 0.000 |
| 1 | 0.264 | -0.899 | 0.000 |
| 1 | 0.352 | 1.612 | 0.000 |
| 1 | -0.691 | 3.022 | 0.000 |
| 1 | -5.586 | 3.458 | 0.000 |
| 1 | -6.071 | 0.968 | 0.000 |
| 1 | -2.233 | -2.022 | 0.000 |
| 1 | 4.583 | 0.715 | 0.000 |
| 1 | 5.458 | -0.541 | -0.883 |
| 1 | 5.458 | -0.541 | 0.883 |
| 6 | -4.235 | 9.026 | 0.000 |
| 6 | -3.179 | 7.963 | 0.000 |
| 6 | -1.854 | 8.218 | 0.000 |
| 7 | -0.913 | 7.217 | 0.000 |
| 1 | 0.070 | 7.444 | 0.000 |
| 6 | -1.236 | 5.889 | 0.000 |
| 8 | -0.385 | 5.000 | 0.000 |
| 7 | -2.579 | 5.634 | 0.000 |
| 1 | -2.860 | 4.639 | 0.000 |
| 6 | -3.601 | 6.570 | 0.000 |
| 8 | -4.774 | 6.200 | 0.000 |
| 1 | -1.454 | 9.223 | 0.000 |
| 1 | -3.780 | 10.016 | 0.000 |
| 1 | -4.874 | 8.931 | 0.880 |
| 1 | -4.874 | 8.931 | -0.880 |

A•A-T

| 8 | 0.353 | -3.147 | -0.002 |
| --- | --- | --- | --- |
| 8 | 1.814 | 1.158 | 0.001 |
| 7 | 2.603 | -2.759 | -0.001 |
| 7 | 1.125 | -0.998 | 0.000 |
| 7 | -1.031 | 2.088 | 0.002 |
| 7 | -4.059 | 2.668 | 0.002 |
| 7 | -5.494 | 0.953 | 0.001 |
| 7 | -3.921 | -0.914 | 0.000 |
| 7 | -1.648 | -0.141 | 0.001 |
| 6 | 4.632 | 0.435 | 0.001 |
| 6 | 2.119 | -0.039 | 0.000 |
| 6 | 3.486 | -0.539 | 0.000 |
| 6 | 3.653 | -1.880 | -0.001 |
| 6 | 1.293 | -2.360 | -0.001 |
| 6 | -5.320 | 2.309 | 0.002 |
| 6 | -1.990 | 1.166 | 0.001 |
| 6 | -3.371 | 1.472 | 0.001 |
| 6 | -4.248 | 0.391 | 0.001 |
| 6 | -2.606 | -1.080 | 0.000 |
| 1 | 4.633 | -2.339 | -0.001 |
| 1 | 2.773 | -3.754 | -0.002 |
| 1 | 0.141 | -0.672 | 0.000 |
| 1 | -0.062 | 1.787 | 0.001 |
| 1 | -1.243 | 3.083 | 0.002 |
| 1 | -6.162 | 2.983 | 0.002 |
| 1 | -6.376 | 0.463 | 0.001 |
| 1 | -2.241 | -2.101 | 0.000 |
| 7 | -1.175 | 5.140 | 0.001 |
| 6 | -0.011 | 5.815 | 0.000 |
| 6 | -0.065 | 7.226 | -0.001 |
| 6 | -1.334 | 7.797 | -0.001 |
| 7 | -2.507 | 7.143 | 0.000 |
| 6 | -2.329 | 5.829 | 0.001 |
| 7 | -1.108 | 9.145 | -0.002 |
| 6 | 0.248 | 9.321 | -0.003 |
| 7 | 0.918 | 8.195 | -0.002 |
| 7 | 1.148 | 5.148 | 0.000 |
| 1 | -3.222 | 5.214 | 0.002 |
| 1 | 0.684 | 10.308 | -0.004 |
| 1 | 1.162 | 4.142 | 0.000 |
| 1 | 2.019 | 5.650 | -0.001 |
| 1 | -1.806 | 9.874 | -0.002 |
| 1 | 4.242 | 1.450 | 0.001 |
| 1 | 5.259 | 0.307 | -0.883 |
| 1 | 5.258 | 0.306 | 0.884 |

rA•A-T

| 8 | 0.156 | -3.308 | 0.000 |
| --- | --- | --- | --- |
| 8 | 2.050 | 0.825 | 0.000 |
| 7 | 2.434 | -3.153 | 0.000 |
| 7 | 1.144 | -1.250 | 0.000 |
| 7 | -0.696 | 2.050 | 0.000 |
| 7 | -3.663 | 2.907 | 0.000 |
| 7 | -5.247 | 1.334 | 0.000 |
| 7 | -3.854 | -0.669 | 0.000 |
| 7 | -1.519 | -0.112 | 0.000 |
| 6 | 4.779 | -0.184 | 0.000 |
| 6 | 2.230 | -0.397 | 0.000 |
| 6 | 3.539 | -1.035 | 0.000 |
| 6 | 3.569 | -2.386 | 0.000 |
| 6 | 1.171 | -2.621 | 0.000 |
| 6 | -4.952 | 2.666 | 0.000 |
| 6 | -1.736 | 1.221 | 0.000 |
| 6 | -3.083 | 1.654 | 0.000 |
| 6 | -4.057 | 0.660 | 0.000 |
| 6 | -2.561 | -0.957 | 0.000 |
| 1 | 4.496 | -2.944 | 0.000 |
| 1 | 2.501 | -4.160 | 0.000 |
| 1 | 0.199 | -0.822 | 0.000 |
| 1 | 0.242 | 1.662 | 0.000 |
| 1 | -0.826 | 3.058 | 0.000 |
| 1 | -5.726 | 3.417 | 0.000 |
| 1 | -6.170 | 0.927 | 0.000 |
| 1 | -2.292 | -2.008 | 0.000 |
| 7 | -2.570 | 8.103 | 0.000 |
| 6 | -2.356 | 6.773 | 0.000 |
| 6 | -1.017 | 6.318 | 0.000 |
| 6 | -0.030 | 7.298 | 0.000 |
| 7 | -0.219 | 8.627 | 0.000 |
| 6 | -1.513 | 8.927 | 0.000 |
| 7 | 1.152 | 6.607 | 0.000 |
| 6 | 0.838 | 5.279 | 0.000 |
| 7 | -0.454 | 5.057 | 0.000 |
| 7 | -3.401 | 5.943 | 0.000 |
| 1 | -1.754 | 9.985 | 0.000 |
| 1 | 1.601 | 4.516 | 0.000 |
| 1 | -4.327 | 6.338 | 0.000 |
| 1 | -3.290 | 4.934 | 0.000 |
| 1 | 2.081 | 7.001 | 0.000 |
| 1 | 4.495 | 0.866 | 0.000 |
| 1 | 5.389 | -0.376 | -0.883 |
| 1 | 5.390 | -0.377 | 0.883 |

G•G-C

| 7 | -2.647 | -0.074 | 0.000 |
| --- | --- | --- | --- |
| 8 | 1.609 | 1.618 | 0.000 |
| 7 | -0.542 | 0.863 | 0.000 |
| 6 | -1.907 | 1.038 | 0.000 |
| 7 | -2.489 | 2.228 | 0.000 |
| 6 | -1.600 | 3.239 | 0.000 |
| 6 | -0.212 | 3.168 | 0.000 |
| 6 | 0.394 | 1.883 | 0.000 |
| 7 | -1.878 | 4.575 | 0.000 |
| 6 | -0.680 | 5.241 | 0.000 |
| 7 | 0.346 | 4.431 | 0.000 |
| 1 | -2.236 | -1.007 | 0.000 |
| 1 | -3.646 | 0.036 | 0.000 |
| 1 | -0.167 | -0.100 | 0.000 |
| 1 | -2.795 | 4.995 | 0.000 |
| 1 | -0.633 | 6.318 | 0.000 |
| 8 | -1.577 | -2.750 | 0.000 |
| 7 | 2.691 | -1.119 | 0.000 |
| 7 | 0.543 | -1.899 | 0.000 |
| 6 | 2.378 | -3.493 | 0.000 |
| 6 | -0.347 | -2.919 | 0.000 |
| 7 | 0.143 | -4.215 | 0.000 |
| 6 | 1.471 | -4.495 | 0.000 |
| 6 | 1.859 | -2.154 | 0.000 |
| 1 | 3.440 | -3.682 | 0.000 |
| 1 | -0.538 | -4.960 | 0.000 |
| 1 | 1.738 | -5.542 | 0.000 |
| 1 | 2.331 | -0.170 | 0.000 |
| 1 | 3.688 | -1.244 | 0.000 |
| 6 | 8.512 | 3.019 | 0.000 |
| 7 | 7.883 | 4.240 | 0.000 |
| 1 | 8.328 | 5.145 | 0.000 |
| 6 | 6.540 | 4.000 | 0.000 |
| 6 | 6.428 | 2.615 | 0.000 |
| 7 | 7.674 | 2.016 | 0.000 |
| 6 | 5.121 | 2.040 | 0.000 |
| 8 | 4.812 | 0.846 | 0.000 |
| 7 | 4.130 | 3.028 | 0.000 |
| 1 | 3.168 | 2.677 | 0.000 |
| 6 | 4.351 | 4.384 | 0.000 |
| 7 | 5.558 | 4.924 | 0.000 |
| 7 | 3.270 | 5.175 | 0.000 |
| 1 | 9.588 | 2.943 | 0.000 |
| 1 | 2.313 | 4.829 | 0.000 |
| 1 | 3.432 | 6.167 | 0.000 |

rG•G-C

| 7 | -2.625 | 0.017 | 0.000 |
| --- | --- | --- | --- |
| 8 | 1.757 | 1.362 | 0.000 |
| 7 | -0.452 | 0.777 | 0.000 |
| 6 | -1.798 | 1.065 | 0.000 |
| 7 | -2.282 | 2.298 | 0.000 |
| 6 | -1.315 | 3.234 | 0.000 |
| 6 | 0.062 | 3.047 | 0.000 |
| 6 | 0.565 | 1.718 | 0.000 |
| 7 | -1.481 | 4.589 | 0.000 |
| 6 | -0.234 | 5.155 | 0.000 |
| 7 | 0.718 | 4.260 | 0.000 |
| 1 | -2.287 | -0.946 | 0.000 |
| 1 | -3.613 | 0.205 | 0.000 |
| 1 | -0.156 | -0.213 | 0.000 |
| 1 | -2.362 | 5.082 | 0.000 |
| 1 | -0.084 | 6.223 | 0.000 |
| 8 | -1.754 | -2.726 | 0.000 |
| 7 | 2.638 | -1.457 | 0.000 |
| 7 | 0.430 | -2.053 | 0.000 |
| 6 | 2.125 | -3.795 | 0.000 |
| 6 | -0.542 | -2.996 | 0.000 |
| 7 | -0.162 | -4.328 | 0.000 |
| 6 | 1.137 | -4.718 | 0.000 |
| 6 | 1.718 | -2.418 | 0.000 |
| 1 | 3.167 | -4.072 | 0.000 |
| 1 | -0.903 | -5.014 | 0.000 |
| 1 | 1.317 | -5.784 | 0.000 |
| 1 | 2.355 | -0.481 | 0.000 |
| 1 | 3.616 | -1.691 | 0.000 |
| 6 | 6.744 | 8.030 | 0.000 |
| 7 | 7.088 | 6.701 | 0.000 |
| 1 | 8.023 | 6.323 | 0.000 |
| 6 | 5.927 | 5.985 | 0.000 |
| 6 | 4.920 | 6.944 | 0.000 |
| 7 | 5.451 | 8.220 | 0.000 |
| 6 | 3.562 | 6.499 | 0.000 |
| 8 | 2.531 | 7.177 | 0.000 |
| 7 | 3.486 | 5.105 | 0.000 |
| 1 | 2.527 | 4.724 | 0.000 |
| 6 | 4.551 | 4.242 | 0.000 |
| 7 | 5.813 | 4.640 | 0.000 |
| 7 | 4.263 | 2.932 | 0.000 |
| 1 | 7.496 | 8.803 | 0.000 |
| 1 | 3.317 | 2.567 | 0.000 |
| 1 | 5.035 | 2.289 | 0.000 |

C^+^•G-C/C^+^•G-C

| 7 | -1.226 | -3.242 | 2.199 |
| --- | --- | --- | --- |
| 8 | 1.276 | 0.464 | 1.215 |
| 7 | 0.114 | -1.434 | 1.708 |
| 6 | 0.009 | -2.780 | 1.970 |
| 7 | 1.050 | -3.595 | 2.025 |
| 6 | 2.211 | -2.967 | 1.781 |
| 6 | 2.421 | -1.620 | 1.504 |
| 6 | 1.293 | -0.762 | 1.452 |
| 7 | 3.456 | -3.526 | 1.747 |
| 6 | 4.346 | -2.529 | 1.456 |
| 7 | 3.763 | -1.367 | 1.305 |
| 1 | -2.045 | -2.668 | 2.001 |
| 1 | -1.330 | -4.239 | 2.281 |
| 1 | -0.743 | -0.850 | 1.713 |
| 1 | 3.678 | -4.499 | 1.900 |
| 1 | 5.404 | -2.718 | 1.371 |
| 8 | -3.532 | -1.586 | 1.655 |
| 7 | -1.004 | 2.200 | 2.041 |
| 7 | -2.241 | 0.290 | 1.827 |
| 6 | -3.394 | 2.408 | 2.106 |
| 6 | -3.431 | -0.354 | 1.763 |
| 7 | -4.587 | 0.406 | 1.815 |
| 6 | -4.571 | 1.751 | 1.994 |
| 6 | -2.201 | 1.618 | 1.987 |
| 1 | -3.345 | 3.477 | 2.245 |
| 1 | -5.463 | -0.090 | 1.730 |
| 1 | -5.534 | 2.240 | 2.036 |
| 1 | -0.181 | 1.656 | 1.800 |
| 1 | -0.930 | 3.203 | 2.034 |
| 6 | 5.335 | 3.483 | 0.408 |
| 6 | 4.653 | 4.617 | 0.131 |
| 7 | 3.294 | 4.648 | 0.141 |
| 6 | 2.518 | 3.557 | 0.434 |
| 8 | 1.297 | 3.597 | 0.458 |
| 7 | 3.227 | 2.405 | 0.713 |
| 1 | 2.636 | 1.584 | 0.931 |
| 6 | 4.581 | 2.309 | 0.708 |
| 7 | 5.144 | 1.154 | 0.968 |
| 1 | 6.413 | 3.448 | 0.397 |
| 1 | 5.142 | 5.549 | -0.114 |
| 1 | 4.605 | 0.285 | 1.116 |
| 1 | 6.151 | 1.095 | 0.955 |
| 1 | 2.801 | 5.506 | -0.068 |
| 7 | -5.010 | -1.181 | -1.353 |
| 8 | -0.468 | -0.620 | -1.476 |
| 7 | -2.716 | -0.990 | -1.405 |
| 6 | -3.825 | -1.797 | -1.299 |
| 7 | -3.760 | -3.115 | -1.180 |
| 6 | -2.497 | -3.568 | -1.133 |
| 6 | -1.316 | -2.839 | -1.231 |
| 6 | -1.409 | -1.434 | -1.377 |
| 7 | -2.092 | -4.862 | -0.975 |
| 6 | -0.722 | -4.866 | -0.982 |
| 7 | -0.217 | -3.670 | -1.137 |
| 1 | -5.094 | -0.164 | -1.357 |
| 1 | -5.828 | -1.746 | -1.199 |
| 1 | -2.849 | 0.035 | -1.443 |
| 1 | -2.690 | -5.667 | -0.870 |
| 1 | -0.158 | -5.778 | -0.871 |
| 8 | -5.340 | 1.692 | -1.290 |
| 7 | -0.803 | 2.268 | -1.251 |
| 7 | -3.068 | 1.941 | -1.297 |
| 6 | -2.197 | 4.149 | -0.770 |
| 6 | -4.332 | 2.408 | -1.168 |
| 7 | -4.508 | 3.753 | -0.884 |
| 6 | -3.468 | 4.600 | -0.679 |
| 6 | -2.026 | 2.763 | -1.107 |
| 1 | -1.344 | 4.791 | -0.608 |
| 1 | -5.460 | 4.079 | -0.801 |
| 1 | -3.724 | 5.623 | -0.441 |
| 1 | -0.675 | 1.272 | -1.405 |
| 1 | 0.003 | 2.813 | -0.984 |
| 6 | 4.506 | -1.653 | -2.014 |
| 6 | 4.919 | -0.384 | -2.225 |
| 7 | 4.046 | 0.659 | -2.206 |
| 6 | 2.700 | 0.513 | -1.979 |
| 8 | 1.921 | 1.451 | -1.961 |
| 7 | 2.293 | -0.792 | -1.770 |
| 1 | 1.274 | -0.896 | -1.621 |
| 6 | 3.116 | -1.870 | -1.766 |
| 7 | 2.614 | -3.060 | -1.541 |
| 1 | 5.191 | -2.486 | -2.020 |
| 1 | 5.951 | -0.128 | -2.417 |
| 1 | 1.609 | -3.224 | -1.372 |
| 1 | 3.236 | -3.855 | -1.541 |
| 1 | 4.372 | 1.601 | -2.377 |

C^+^•G-C/T•A-T

| 8 | 4.609 | -1.845 | -2.188 |
| --- | --- | --- | --- |
| 8 | 2.579 | 2.117 | -1.241 |
| 7 | 5.894 | 0.035 | -2.036 |
| 7 | 3.619 | 0.155 | -1.708 |
| 7 | -0.129 | 0.829 | -1.577 |
| 7 | -2.643 | -0.963 | -1.667 |
| 7 | -2.417 | -3.151 | -2.000 |
| 7 | 0.015 | -3.299 | -2.160 |
| 7 | 1.041 | -1.147 | -1.879 |
| 6 | 5.020 | 3.629 | -1.255 |
| 6 | 3.633 | 1.513 | -1.465 |
| 6 | 4.938 | 2.153 | -1.502 |
| 6 | 6.006 | 1.377 | -1.785 |
| 6 | 4.703 | -0.639 | -1.986 |
| 6 | -3.268 | -2.110 | -1.785 |
| 6 | -0.133 | -0.492 | -1.745 |
| 6 | -1.308 | -1.275 | -1.804 |
| 6 | -1.147 | -2.641 | -2.008 |
| 6 | 1.048 | -2.474 | -2.076 |
| 1 | 7.012 | 1.772 | -1.837 |
| 1 | 6.718 | -0.519 | -2.218 |
| 1 | 2.695 | -0.314 | -1.723 |
| 1 | 0.764 | 1.300 | -1.461 |
| 1 | -0.988 | 1.345 | -1.426 |
| 1 | -4.337 | -2.240 | -1.722 |
| 1 | -2.676 | -4.121 | -2.110 |
| 1 | 2.036 | -2.912 | -2.173 |
| 1 | 4.477 | 4.179 | -2.027 |
| 1 | 6.059 | 3.958 | -1.255 |
| 1 | 4.571 | 3.883 | -0.294 |
| 6 | -3.966 | 5.143 | -0.700 |
| 6 | -4.520 | 3.759 | -0.855 |
| 6 | -5.835 | 3.460 | -0.787 |
| 7 | -6.307 | 2.180 | -0.928 |
| 1 | -7.297 | 1.994 | -0.855 |
| 6 | -5.491 | 1.101 | -1.138 |
| 8 | -5.908 | -0.050 | -1.228 |
| 7 | -4.158 | 1.414 | -1.231 |
| 1 | -3.529 | 0.606 | -1.396 |
| 6 | -3.592 | 2.665 | -1.098 |
| 8 | -2.369 | 2.806 | -1.181 |
| 1 | -6.592 | 4.213 | -0.614 |
| 1 | -4.770 | 5.857 | -0.527 |
| 1 | -3.417 | 5.439 | -1.596 |
| 1 | -3.270 | 5.184 | 0.139 |
| 7 | 3.889 | -2.822 | 1.024 |
| 8 | 0.345 | 0.024 | 1.607 |
| 7 | 2.052 | -1.463 | 1.312 |
| 6 | 2.557 | -2.721 | 1.082 |
| 7 | 1.800 | -3.799 | 0.946 |
| 6 | 0.488 | -3.528 | 1.052 |
| 6 | -0.119 | -2.297 | 1.267 |
| 6 | 0.708 | -1.152 | 1.410 |
| 7 | -0.545 | -4.417 | 0.962 |
| 6 | -1.707 | -3.714 | 1.116 |
| 7 | -1.492 | -2.437 | 1.303 |
| 1 | 4.489 | -1.997 | 0.997 |
| 1 | 4.269 | -3.717 | 0.767 |
| 1 | 2.711 | -0.670 | 1.403 |
| 1 | -0.466 | -5.409 | 0.796 |
| 1 | -2.677 | -4.183 | 1.080 |
| 8 | 5.647 | -0.539 | 1.043 |
| 7 | 2.217 | 2.220 | 2.264 |
| 7 | 3.909 | 0.808 | 1.662 |
| 6 | 4.462 | 3.022 | 2.493 |
| 6 | 5.225 | 0.540 | 1.489 |
| 7 | 6.143 | 1.522 | 1.828 |
| 6 | 5.771 | 2.730 | 2.318 |
| 6 | 3.521 | 2.000 | 2.132 |
| 1 | 4.135 | 3.975 | 2.878 |
| 1 | 7.118 | 1.300 | 1.690 |
| 1 | 6.572 | 3.419 | 2.550 |
| 1 | 1.551 | 1.496 | 2.006 |
| 1 | 1.883 | 3.101 | 2.617 |
| 6 | -4.640 | 1.431 | 2.088 |
| 6 | -5.826 | 0.777 | 2.106 |
| 7 | -5.906 | -0.559 | 1.877 |
| 6 | -4.811 | -1.339 | 1.588 |
| 8 | -4.884 | -2.531 | 1.344 |
| 7 | -3.608 | -0.664 | 1.605 |
| 1 | -2.764 | -1.281 | 1.451 |
| 6 | -3.466 | 0.662 | 1.827 |
| 7 | -2.263 | 1.191 | 1.790 |
| 1 | -4.570 | 2.493 | 2.267 |
| 1 | -6.764 | 1.278 | 2.299 |
| 1 | -1.414 | 0.640 | 1.640 |
| 1 | -2.161 | 2.182 | 1.947 |
| 1 | -6.805 | -1.020 | 1.861 |

T•A-T/T•A-T

| 8 | -4.816 | -3.035 | 0.359 |
| --- | --- | --- | --- |
| 8 | -3.472 | 1.038 | 1.889 |
| 7 | -6.374 | -1.384 | 0.598 |
| 7 | -4.169 | -0.984 | 1.128 |
| 7 | -0.633 | 0.114 | 1.665 |
| 7 | 2.129 | -1.267 | 1.703 |
| 7 | 2.239 | -3.490 | 1.735 |
| 7 | -0.143 | -4.026 | 1.683 |
| 7 | -1.489 | -2.038 | 1.645 |
| 6 | -6.098 | 2.186 | 1.807 |
| 6 | -4.403 | 0.325 | 1.500 |
| 6 | -5.778 | 0.782 | 1.395 |
| 6 | -6.696 | -0.099 | 0.942 |
| 6 | -5.101 | -1.883 | 0.670 |
| 6 | 2.922 | -2.311 | 1.730 |
| 6 | -0.429 | -1.202 | 1.660 |
| 6 | 0.855 | -1.795 | 1.685 |
| 6 | 0.905 | -3.184 | 1.700 |
| 6 | -1.290 | -3.364 | 1.649 |
| 1 | -7.741 | 0.160 | 0.829 |
| 1 | -7.085 | -2.010 | 0.249 |
| 1 | -3.211 | -1.353 | 1.261 |
| 1 | -1.587 | 0.462 | 1.655 |
| 1 | 0.136 | 0.761 | 1.536 |
| 1 | 3.999 | -2.274 | 1.735 |
| 1 | 2.645 | -4.414 | 1.746 |
| 1 | -2.195 | -3.962 | 1.616 |
| 1 | -5.915 | 2.326 | 2.874 |
| 1 | -7.143 | 2.416 | 1.597 |
| 1 | -5.465 | 2.895 | 1.271 |
| 6 | 2.376 | 4.974 | 0.867 |
| 6 | 3.177 | 3.744 | 1.165 |
| 6 | 4.508 | 3.747 | 1.393 |
| 7 | 5.213 | 2.600 | 1.654 |
| 1 | 6.211 | 2.639 | 1.796 |
| 6 | 4.634 | 1.359 | 1.704 |
| 8 | 5.268 | 0.331 | 1.915 |
| 7 | 3.277 | 1.366 | 1.498 |
| 1 | 2.811 | 0.444 | 1.553 |
| 6 | 2.489 | 2.464 | 1.221 |
| 8 | 1.274 | 2.328 | 1.048 |
| 1 | 5.097 | 4.654 | 1.376 |
| 1 | 3.017 | 5.854 | 0.859 |
| 1 | 1.593 | 5.114 | 1.615 |
| 1 | 1.887 | 4.886 | -0.105 |
| 8 | -5.137 | -0.112 | -2.056 |
| 8 | -1.643 | 2.505 | -0.774 |
| 7 | -5.487 | 2.130 | -1.789 |
| 7 | -3.399 | 1.230 | -1.431 |
| 7 | 0.256 | 0.284 | -1.521 |
| 7 | 1.803 | -2.380 | -1.546 |
| 7 | 0.676 | -4.301 | -1.622 |
| 7 | -1.609 | -3.442 | -1.672 |
| 7 | -1.643 | -1.043 | -1.586 |
| 6 | -3.181 | 4.926 | -0.707 |
| 6 | -2.839 | 2.439 | -1.073 |
| 6 | -3.732 | 3.586 | -1.089 |
| 6 | -5.014 | 3.373 | -1.455 |
| 6 | -4.706 | 1.003 | -1.779 |
| 6 | 1.892 | -3.688 | -1.557 |
| 6 | -0.298 | -0.926 | -1.570 |
| 6 | 0.449 | -2.126 | -1.603 |
| 6 | -0.272 | -3.314 | -1.643 |
| 6 | -2.206 | -2.260 | -1.637 |
| 1 | -5.745 | 4.169 | -1.501 |
| 1 | -6.458 | 2.005 | -2.035 |
| 1 | -2.770 | 0.403 | -1.452 |
| 1 | -0.341 | 1.086 | -1.352 |
| 1 | 1.263 | 0.403 | -1.518 |
| 1 | 2.812 | -4.249 | -1.513 |
| 1 | 0.509 | -5.296 | -1.626 |
| 1 | -3.290 | -2.261 | -1.644 |
| 1 | -2.797 | 4.904 | 0.315 |
| 1 | -3.955 | 5.689 | -0.775 |
| 1 | -2.353 | 5.202 | -1.362 |
| 6 | 5.450 | 2.742 | -1.974 |
| 6 | 5.425 | 1.303 | -1.560 |
| 6 | 6.524 | 0.598 | -1.213 |
| 7 | 6.468 | -0.725 | -0.863 |
| 1 | 7.309 | -1.215 | -0.596 |
| 6 | 5.302 | -1.441 | -0.823 |
| 8 | 5.254 | -2.626 | -0.507 |
| 7 | 4.188 | -0.721 | -1.174 |
| 1 | 3.307 | -1.254 | -1.228 |
| 6 | 4.149 | 0.609 | -1.542 |
| 8 | 3.073 | 1.143 | -1.828 |
| 1 | 7.512 | 1.037 | -1.196 |
| 1 | 6.453 | 3.154 | -1.864 |
| 1 | 4.758 | 3.327 | -1.366 |
| 1 | 5.139 | 2.848 | -3.016 |

rC^+^•G-C/rC^+^•G-C

| 7 | -1.226 | -3.242 | 2.199 |
| --- | --- | --- | --- |
| 8 | 1.276 | 0.464 | 1.215 |
| 7 | 0.114 | -1.434 | 1.708 |
| 6 | 0.009 | -2.780 | 1.970 |
| 7 | 1.050 | -3.595 | 2.025 |
| 6 | 2.211 | -2.967 | 1.781 |
| 6 | 2.421 | -1.620 | 1.504 |
| 6 | 1.293 | -0.762 | 1.452 |
| 7 | 3.456 | -3.526 | 1.747 |
| 6 | 4.346 | -2.529 | 1.456 |
| 7 | 3.763 | -1.367 | 1.305 |
| 1 | -2.045 | -2.668 | 2.001 |
| 1 | -1.330 | -4.239 | 2.281 |
| 1 | -0.743 | -0.850 | 1.713 |
| 1 | 3.678 | -4.499 | 1.900 |
| 1 | 5.404 | -2.718 | 1.371 |
| 8 | -3.532 | -1.586 | 1.655 |
| 7 | -1.004 | 2.200 | 2.041 |
| 7 | -2.241 | 0.290 | 1.827 |
| 6 | -3.394 | 2.408 | 2.106 |
| 6 | -3.431 | -0.354 | 1.763 |
| 7 | -4.587 | 0.406 | 1.815 |
| 6 | -4.571 | 1.751 | 1.994 |
| 6 | -2.201 | 1.618 | 1.987 |
| 1 | -3.345 | 3.477 | 2.245 |
| 1 | -5.463 | -0.090 | 1.730 |
| 1 | -5.534 | 2.240 | 2.036 |
| 1 | -0.181 | 1.656 | 1.800 |
| 1 | -0.930 | 3.203 | 2.034 |
| 6 | 5.335 | 3.483 | 0.408 |
| 6 | 4.653 | 4.617 | 0.131 |
| 7 | 3.294 | 4.648 | 0.141 |
| 6 | 2.518 | 3.557 | 0.434 |
| 8 | 1.297 | 3.597 | 0.458 |
| 7 | 3.227 | 2.405 | 0.713 |
| 1 | 2.636 | 1.584 | 0.931 |
| 6 | 4.581 | 2.309 | 0.708 |
| 7 | 5.144 | 1.154 | 0.968 |
| 1 | 6.413 | 3.448 | 0.397 |
| 1 | 5.142 | 5.549 | -0.114 |
| 1 | 4.605 | 0.285 | 1.116 |
| 1 | 6.151 | 1.095 | 0.955 |
| 1 | 2.801 | 5.506 | -0.068 |
| 7 | -5.010 | -1.181 | -1.353 |
| 8 | -0.468 | -0.620 | -1.476 |
| 7 | -2.716 | -0.990 | -1.405 |
| 6 | -3.825 | -1.797 | -1.299 |
| 7 | -3.760 | -3.115 | -1.180 |
| 6 | -2.497 | -3.568 | -1.133 |
| 6 | -1.316 | -2.839 | -1.231 |
| 6 | -1.409 | -1.434 | -1.377 |
| 7 | -2.092 | -4.862 | -0.975 |
| 6 | -0.722 | -4.866 | -0.982 |
| 7 | -0.217 | -3.670 | -1.137 |
| 1 | -5.094 | -0.164 | -1.357 |
| 1 | -5.828 | -1.746 | -1.199 |
| 1 | -2.849 | 0.035 | -1.443 |
| 1 | -2.690 | -5.667 | -0.870 |
| 1 | -0.158 | -5.778 | -0.871 |
| 8 | -5.340 | 1.692 | -1.290 |
| 7 | -0.803 | 2.268 | -1.251 |
| 7 | -3.068 | 1.941 | -1.297 |
| 6 | -2.197 | 4.149 | -0.770 |
| 6 | -4.332 | 2.408 | -1.168 |
| 7 | -4.508 | 3.753 | -0.884 |
| 6 | -3.468 | 4.600 | -0.679 |
| 6 | -2.026 | 2.763 | -1.107 |
| 1 | -1.344 | 4.791 | -0.608 |
| 1 | -5.460 | 4.079 | -0.801 |
| 1 | -3.724 | 5.623 | -0.441 |
| 1 | -0.675 | 1.272 | -1.405 |
| 1 | 0.003 | 2.813 | -0.984 |
| 6 | 4.506 | -1.653 | -2.014 |
| 6 | 4.919 | -0.384 | -2.225 |
| 7 | 4.046 | 0.659 | -2.206 |
| 6 | 2.700 | 0.513 | -1.979 |
| 8 | 1.921 | 1.451 | -1.961 |
| 7 | 2.293 | -0.792 | -1.770 |
| 1 | 1.274 | -0.896 | -1.621 |
| 6 | 3.116 | -1.870 | -1.766 |
| 7 | 2.614 | -3.060 | -1.541 |
| 1 | 5.191 | -2.486 | -2.020 |
| 1 | 5.951 | -0.128 | -2.417 |
| 1 | 1.609 | -3.224 | -1.372 |
| 1 | 3.236 | -3.855 | -1.541 |
| 1 | 4.372 | 1.601 | -2.377 |

rT•A-T/rT•A-T

| 8 | -4.346 | 2.241 | -1.821 |
| --- | --- | --- | --- |
| 8 | -2.078 | -1.705 | -1.803 |
| 7 | -5.523 | 0.299 | -2.072 |
| 7 | -3.235 | 0.246 | -1.822 |
| 7 | 0.524 | -0.203 | -1.461 |
| 7 | 2.894 | 1.729 | -1.066 |
| 7 | 2.519 | 3.923 | -1.014 |
| 7 | 0.109 | 3.944 | -1.405 |
| 7 | -0.762 | 1.717 | -1.619 |
| 6 | -4.417 | -3.318 | -2.147 |
| 6 | -3.167 | -1.130 | -1.892 |
| 6 | -4.433 | -1.822 | -2.076 |
| 6 | -5.551 | -1.070 | -2.155 |
| 6 | -4.367 | 1.017 | -1.898 |
| 6 | 3.432 | 2.915 | -0.916 |
| 6 | 0.441 | 1.125 | -1.456 |
| 6 | 1.554 | 1.978 | -1.275 |
| 6 | 1.301 | 3.344 | -1.247 |
| 6 | -0.857 | 3.055 | -1.588 |
| 1 | -6.535 | -1.502 | -2.284 |
| 1 | -6.379 | 0.830 | -2.128 |
| 1 | -2.341 | 0.763 | -1.716 |
| 1 | -0.324 | -0.746 | -1.584 |
| 1 | 1.416 | -0.677 | -1.380 |
| 1 | 4.479 | 3.101 | -0.738 |
| 1 | 2.711 | 4.911 | -0.933 |
| 1 | -1.861 | 3.442 | -1.723 |
| 1 | -3.930 | -3.725 | -1.260 |
| 1 | -3.854 | -3.659 | -3.018 |
| 1 | -5.431 | -3.710 | -2.207 |
| 6 | 8.379 | -1.079 | -1.232 |
| 6 | 6.909 | -1.361 | -1.291 |
| 6 | 6.385 | -2.595 | -1.446 |
| 7 | 5.031 | -2.817 | -1.500 |
| 1 | 4.673 | -3.755 | -1.606 |
| 6 | 4.107 | -1.815 | -1.412 |
| 8 | 2.894 | -2.025 | -1.456 |
| 7 | 4.639 | -0.564 | -1.265 |
| 1 | 3.966 | 0.218 | -1.188 |
| 6 | 5.984 | -0.243 | -1.172 |
| 8 | 6.326 | 0.926 | -0.996 |
| 1 | 6.999 | -3.481 | -1.535 |
| 1 | 8.948 | -2.000 | -1.353 |
| 1 | 8.646 | -0.623 | -0.277 |
| 1 | 8.666 | -0.380 | -2.020 |
| 8 | -6.204 | 1.547 | 0.932 |
| 8 | -3.737 | -2.267 | 1.218 |
| 7 | -7.289 | -0.463 | 0.939 |
| 7 | -4.993 | -0.383 | 1.087 |
| 7 | -1.238 | -0.581 | 1.435 |
| 7 | 1.013 | 1.475 | 1.919 |
| 7 | 0.521 | 3.646 | 1.969 |
| 7 | -1.888 | 3.535 | 1.584 |
| 7 | -2.634 | 1.264 | 1.350 |
| 6 | -6.026 | -4.010 | 1.279 |
| 6 | -4.860 | -1.755 | 1.155 |
| 6 | -6.098 | -2.519 | 1.148 |
| 6 | -7.253 | -1.831 | 1.038 |
| 6 | -6.167 | 0.322 | 0.987 |
| 6 | 1.488 | 2.689 | 2.060 |
| 6 | -1.399 | 0.739 | 1.497 |
| 6 | -0.340 | 1.649 | 1.719 |
| 6 | -0.666 | 3.000 | 1.746 |
| 6 | -2.803 | 2.594 | 1.394 |
| 1 | -8.219 | -2.317 | 1.020 |
| 1 | -8.174 | 0.016 | 0.862 |
| 1 | -4.129 | 0.190 | 1.150 |
| 1 | -2.050 | -1.170 | 1.283 |
| 1 | -0.317 | -1.001 | 1.483 |
| 1 | 2.526 | 2.931 | 2.224 |
| 1 | 0.660 | 4.643 | 2.036 |
| 1 | -3.826 | 2.926 | 1.254 |
| 1 | -5.510 | -4.288 | 2.200 |
| 1 | -5.474 | -4.450 | 0.448 |
| 1 | -7.028 | -4.437 | 1.297 |
| 6 | 6.631 | -0.961 | 2.193 |
| 6 | 5.200 | -1.337 | 1.955 |
| 6 | 4.767 | -2.605 | 1.794 |
| 7 | 3.442 | -2.911 | 1.598 |
| 1 | 3.154 | -3.871 | 1.479 |
| 6 | 2.455 | -1.965 | 1.558 |
| 8 | 1.267 | -2.250 | 1.409 |
| 7 | 2.899 | -0.680 | 1.705 |
| 1 | 2.175 | 0.058 | 1.725 |
| 6 | 4.208 | -0.272 | 1.904 |
| 8 | 4.469 | 0.925 | 2.021 |
| 1 | 5.436 | -3.455 | 1.809 |
| 1 | 7.279 | -1.831 | 2.085 |
| 1 | 6.758 | -0.549 | 3.197 |
| 1 | 6.945 | -0.194 | 1.484 |

G•G-C/G•G-C

| 7 | 5.066 | 1.055 | -1.902 |
| --- | --- | --- | --- |
| 8 | 0.501 | 1.145 | -1.537 |
| 7 | 2.778 | 1.184 | -1.669 |
| 6 | 3.999 | 1.817 | -1.636 |
| 7 | 4.146 | 3.107 | -1.377 |
| 6 | 2.973 | 3.724 | -1.141 |
| 6 | 1.692 | 3.183 | -1.172 |
| 6 | 1.556 | 1.802 | -1.464 |
| 7 | 2.787 | 5.034 | -0.813 |
| 6 | 1.438 | 5.227 | -0.657 |
| 7 | 0.745 | 4.140 | -0.868 |
| 1 | 4.991 | 0.047 | -2.035 |
| 1 | 5.972 | 1.480 | -1.807 |
| 1 | 2.750 | 0.164 | -1.836 |
| 1 | 3.507 | 5.732 | -0.702 |
| 1 | 1.033 | 6.191 | -0.389 |
| 8 | 4.928 | -1.814 | -2.277 |
| 7 | 0.430 | -1.787 | -1.460 |
| 7 | 2.672 | -1.764 | -1.921 |
| 6 | 1.591 | -3.873 | -1.371 |
| 6 | 3.862 | -2.398 | -2.016 |
| 7 | 3.895 | -3.768 | -1.808 |
| 6 | 2.787 | -4.488 | -1.491 |
| 6 | 1.571 | -2.452 | -1.583 |
| 1 | 0.692 | -4.408 | -1.106 |
| 1 | 4.796 | -4.218 | -1.883 |
| 1 | 2.933 | -5.548 | -1.344 |
| 1 | 0.423 | -0.777 | -1.565 |
| 1 | -0.395 | -2.235 | -1.081 |
| 6 | -6.436 | 0.047 | -1.543 |
| 7 | -6.268 | 1.334 | -1.094 |
| 1 | -6.996 | 1.969 | -0.804 |
| 6 | -4.931 | 1.599 | -1.129 |
| 6 | -4.351 | 0.428 | -1.599 |
| 7 | -5.309 | -0.531 | -1.862 |
| 6 | -2.928 | 0.376 | -1.716 |
| 8 | -2.226 | -0.568 | -2.083 |
| 7 | -2.342 | 1.591 | -1.343 |
| 1 | -1.320 | 1.612 | -1.398 |
| 6 | -3.019 | 2.720 | -0.952 |
| 7 | -4.332 | 2.764 | -0.800 |
| 7 | -2.289 | 3.829 | -0.758 |
| 1 | -7.416 | -0.400 | -1.609 |
| 1 | -1.271 | 3.822 | -0.752 |
| 1 | -2.764 | 4.614 | -0.347 |
| 7 | 3.968 | 1.718 | 1.740 |
| 8 | -0.135 | -0.296 | 1.506 |
| 7 | 1.863 | 0.794 | 1.630 |
| 6 | 2.647 | 1.905 | 1.838 |
| 7 | 2.159 | 3.099 | 2.137 |
| 6 | 0.815 | 3.105 | 2.201 |
| 6 | -0.063 | 2.046 | 1.996 |
| 6 | 0.481 | 0.771 | 1.694 |
| 7 | 0.012 | 4.166 | 2.498 |
| 6 | -1.284 | 3.722 | 2.460 |
| 7 | -1.371 | 2.452 | 2.166 |
| 1 | 4.365 | 0.834 | 1.425 |
| 1 | 4.554 | 2.534 | 1.802 |
| 1 | 2.325 | -0.116 | 1.461 |
| 1 | 0.316 | 5.109 | 2.693 |
| 1 | -2.114 | 4.382 | 2.657 |
| 8 | 5.148 | -0.814 | 0.995 |
| 7 | 1.189 | -2.928 | 1.852 |
| 7 | 3.153 | -1.841 | 1.427 |
| 6 | 3.202 | -4.229 | 1.860 |
| 6 | 4.496 | -1.839 | 1.253 |
| 7 | 5.171 | -3.043 | 1.371 |
| 6 | 4.541 | -4.207 | 1.674 |
| 6 | 2.511 | -2.980 | 1.710 |
| 1 | 2.671 | -5.139 | 2.094 |
| 1 | 6.171 | -3.020 | 1.234 |
| 1 | 5.169 | -5.084 | 1.748 |
| 1 | 0.694 | -2.055 | 1.689 |
| 1 | 0.661 | -3.769 | 2.012 |
| 6 | -5.819 | -4.144 | -0.032 |
| 7 | -6.282 | -2.991 | 0.554 |
| 1 | -7.247 | -2.748 | 0.721 |
| 6 | -5.195 | -2.223 | 0.844 |
| 6 | -4.106 | -2.974 | 0.415 |
| 7 | -4.517 | -4.175 | -0.132 |
| 6 | -2.802 | -2.424 | 0.572 |
| 8 | -1.716 | -2.937 | 0.267 |
| 7 | -2.848 | -1.157 | 1.155 |
| 1 | -1.939 | -0.707 | 1.297 |
| 6 | -3.989 | -0.493 | 1.535 |
| 7 | -5.202 | -1.001 | 1.411 |
| 7 | -3.827 | 0.717 | 2.096 |
| 1 | -6.496 | -4.919 | -0.356 |
| 1 | -2.947 | 1.228 | 2.049 |
| 1 | -4.670 | 1.248 | 2.242 |

rG•G-C/rG•G-C

| 7 | 3.490 | -3.356 | -1.861 |
| --- | --- | --- | --- |
| 8 | 1.116 | 0.491 | -1.104 |
| 7 | 2.214 | -1.478 | -1.467 |
| 6 | 2.265 | -2.828 | -1.721 |
| 7 | 1.190 | -3.586 | -1.857 |
| 6 | 0.045 | -2.886 | -1.740 |
| 6 | -0.112 | -1.526 | -1.501 |
| 6 | 1.055 | -0.730 | -1.337 |
| 7 | -1.227 | -3.372 | -1.841 |
| 6 | -2.082 | -2.318 | -1.667 |
| 7 | -1.450 | -1.192 | -1.460 |
| 1 | 4.318 | -2.843 | -1.561 |
| 1 | 3.540 | -4.360 | -1.915 |
| 1 | 3.106 | -0.957 | -1.394 |
| 1 | -1.489 | -4.332 | -2.010 |
| 1 | -3.152 | -2.438 | -1.705 |
| 8 | 5.898 | -1.933 | -1.086 |
| 7 | 3.641 | 2.001 | -1.653 |
| 7 | 4.737 | 0.009 | -1.405 |
| 6 | 6.033 | 2.030 | -1.772 |
| 6 | 5.879 | -0.706 | -1.292 |
| 7 | 7.084 | -0.037 | -1.411 |
| 6 | 7.161 | 1.295 | -1.655 |
| 6 | 4.790 | 1.331 | -1.606 |
| 1 | 6.057 | 3.093 | -1.956 |
| 1 | 7.923 | -0.593 | -1.324 |
| 1 | 8.156 | 1.708 | -1.745 |
| 1 | 2.765 | 1.521 | -1.467 |
| 1 | 3.642 | 3.001 | -1.765 |
| 6 | -6.497 | 3.850 | -1.188 |
| 7 | -5.323 | 4.382 | -0.717 |
| 1 | -5.195 | 5.307 | -0.335 |
| 6 | -4.364 | 3.422 | -0.852 |
| 6 | -5.023 | 2.339 | -1.418 |
| 7 | -6.361 | 2.622 | -1.618 |
| 6 | -4.280 | 1.139 | -1.647 |
| 8 | -4.678 | 0.068 | -2.112 |
| 7 | -2.944 | 1.289 | -1.267 |
| 1 | -2.362 | 0.442 | -1.356 |
| 6 | -2.390 | 2.414 | -0.719 |
| 7 | -3.067 | 3.526 | -0.489 |
| 7 | -1.088 | 2.342 | -0.381 |
| 1 | -7.412 | 4.421 | -1.187 |
| 1 | -0.486 | 1.595 | -0.711 |
| 1 | -0.649 | 3.212 | -0.131 |
| 7 | 4.448 | -1.523 | 1.582 |
| 8 | 0.266 | 0.263 | 2.203 |
| 7 | 2.309 | -0.709 | 1.885 |
| 6 | 3.131 | -1.781 | 1.627 |
| 7 | 2.689 | -3.016 | 1.457 |
| 6 | 1.349 | -3.103 | 1.542 |
| 6 | 0.437 | -2.088 | 1.808 |
| 6 | 0.928 | -0.767 | 1.988 |
| 7 | 0.585 | -4.220 | 1.370 |
| 6 | -0.724 | -3.847 | 1.515 |
| 7 | -0.854 | -2.574 | 1.785 |
| 1 | 4.773 | -0.556 | 1.558 |
| 1 | 5.009 | -2.192 | 1.075 |
| 1 | 2.725 | 0.235 | 1.912 |
| 1 | 0.922 | -5.143 | 1.139 |
| 1 | -1.537 | -4.548 | 1.405 |
| 8 | 5.549 | 1.152 | 1.523 |
| 7 | 1.364 | 2.963 | 1.858 |
| 7 | 3.444 | 2.022 | 1.712 |
| 6 | 3.196 | 4.408 | 1.325 |
| 6 | 4.775 | 2.122 | 1.482 |
| 7 | 5.297 | 3.372 | 1.187 |
| 6 | 4.528 | 4.485 | 1.106 |
| 6 | 2.669 | 3.109 | 1.635 |
| 1 | 2.555 | 5.274 | 1.268 |
| 1 | 6.289 | 3.418 | 1.010 |
| 1 | 5.041 | 5.405 | 0.862 |
| 1 | 0.977 | 2.042 | 2.049 |
| 1 | 0.756 | 3.764 | 1.845 |
| 6 | -7.540 | -1.015 | -0.008 |
| 7 | -6.970 | 0.131 | 0.489 |
| 1 | -7.352 | 1.064 | 0.433 |
| 6 | -5.728 | -0.202 | 0.938 |
| 6 | -5.616 | -1.566 | 0.699 |
| 7 | -6.761 | -2.059 | 0.104 |
| 6 | -4.371 | -2.203 | 0.989 |
| 8 | -4.052 | -3.383 | 0.811 |
| 7 | -3.449 | -1.298 | 1.517 |
| 1 | -2.515 | -1.693 | 1.705 |
| 6 | -3.669 | 0.036 | 1.732 |
| 7 | -4.813 | 0.639 | 1.467 |
| 7 | -2.652 | 0.738 | 2.275 |
| 1 | -8.532 | -1.010 | -0.432 |
| 1 | -1.698 | 0.392 | 2.223 |
| 1 | -2.765 | 1.739 | 2.250 |

A•A-T/A•A-T

| 8 | 4.887 | -2.377 | -1.925 |
| --- | --- | --- | --- |
| 8 | 0.467 | -1.779 | -1.031 |
| 8 | 0.828 | -2.009 | 2.271 |
| 8 | 5.045 | -0.638 | 1.242 |
| 7 | 3.490 | -4.184 | -2.024 |
| 7 | 2.664 | -2.103 | -1.484 |
| 7 | 0.823 | 1.069 | -1.528 |
| 7 | 1.420 | 4.084 | -1.578 |
| 7 | 3.543 | 4.776 | -1.429 |
| 7 | 4.677 | 2.618 | -1.276 |
| 7 | 3.111 | 0.798 | -1.335 |
| 7 | -2.196 | 1.334 | -1.303 |
| 7 | -4.103 | 2.801 | -1.207 |
| 7 | -6.179 | 1.598 | -1.658 |
| 7 | -5.361 | -0.456 | -2.003 |
| 7 | -2.360 | -0.943 | -1.776 |
| 7 | -2.135 | -2.327 | 1.368 |
| 7 | -5.049 | -3.023 | 0.776 |
| 7 | -6.624 | -1.445 | 0.971 |
| 7 | -5.229 | 0.460 | 1.594 |
| 7 | -2.910 | -0.165 | 1.753 |
| 7 | 2.153 | 1.472 | 1.939 |
| 7 | 2.822 | 3.776 | 1.846 |
| 7 | 0.893 | 5.254 | 1.605 |
| 7 | -0.750 | 3.737 | 1.546 |
| 7 | -0.038 | 0.752 | 1.822 |
| 7 | 2.935 | -1.351 | 1.749 |
| 7 | 4.576 | -2.872 | 1.201 |
| 6 | -0.259 | -4.484 | -1.604 |
| 6 | 1.368 | -2.560 | -1.368 |
| 6 | 1.146 | -3.964 | -1.652 |
| 6 | 2.227 | -4.712 | -1.962 |
| 6 | 3.764 | -2.855 | -1.826 |
| 6 | 2.223 | 5.119 | -1.541 |
| 6 | 2.037 | 1.611 | -1.443 |
| 6 | 2.271 | 3.002 | -1.481 |
| 6 | 3.597 | 3.411 | -1.388 |
| 6 | 4.338 | 1.337 | -1.261 |
| 6 | -2.943 | 0.252 | -1.592 |
| 6 | -4.337 | 0.426 | -1.720 |
| 6 | -4.826 | 1.710 | -1.505 |
| 6 | -2.808 | 2.517 | -1.134 |
| 6 | -6.438 | 0.288 | -1.953 |
| 6 | -6.327 | -2.747 | 0.679 |
| 6 | -3.941 | 0.693 | 1.807 |
| 6 | -5.442 | -0.825 | 1.266 |
| 6 | -4.479 | -1.823 | 1.147 |
| 6 | -3.146 | -1.450 | 1.427 |
| 6 | 3.045 | 2.473 | 1.938 |
| 6 | 1.509 | 4.040 | 1.723 |
| 6 | 0.472 | 3.112 | 1.688 |
| 6 | 0.837 | 1.753 | 1.815 |
| 6 | -0.448 | 5.012 | 1.495 |
| 6 | 4.246 | -1.554 | 1.385 |
| 6 | 3.687 | -3.901 | 1.358 |
| 6 | 2.401 | -3.695 | 1.719 |
| 6 | 1.978 | -2.324 | 1.935 |
| 6 | 1.401 | -4.793 | 1.919 |
| 1 | 2.159 | -5.769 | -2.179 |
| 1 | 4.273 | -4.771 | -2.274 |
| 1 | 2.834 | -1.087 | -1.368 |
| 1 | 0.723 | 0.078 | -1.333 |
| 1 | -0.018 | 1.626 | -1.475 |
| 1 | 1.915 | 6.152 | -1.591 |
| 1 | 4.324 | 5.413 | -1.382 |
| 1 | 5.145 | 0.619 | -1.171 |
| 1 | -2.146 | 3.344 | -0.898 |
| 1 | -7.446 | -0.059 | -2.121 |
| 1 | -1.401 | -1.093 | -1.494 |
| 1 | -2.955 | -1.753 | -1.830 |
| 1 | -6.861 | 2.338 | -1.575 |
| 1 | -0.726 | -4.245 | -0.648 |
| 1 | -0.271 | -5.564 | -1.746 |
| 1 | -0.865 | -4.020 | -2.386 |
| 1 | 1.832 | -5.754 | 1.639 |
| 1 | 1.083 | -4.839 | 2.962 |
| 1 | 0.512 | -4.616 | 1.312 |
| 1 | -7.537 | -1.018 | 0.938 |
| 1 | -2.337 | -3.296 | 1.189 |
| 1 | -1.198 | -2.077 | 1.651 |
| 1 | -7.099 | -3.449 | 0.403 |
| 1 | -3.672 | 1.715 | 2.060 |
| 1 | 4.081 | 2.162 | 2.022 |
| 1 | 1.342 | 6.157 | 1.576 |
| 1 | -1.156 | 5.818 | 1.385 |
| 1 | -1.036 | 0.889 | 1.740 |
| 1 | 0.294 | -0.193 | 1.983 |
| 1 | 2.660 | -0.359 | 1.871 |
| 1 | 5.523 | -3.060 | 0.904 |
| 1 | 4.087 | -4.889 | 1.173 |

rA•A-T/rA•A-T

| 8 | 5.240 | -0.413 | -1.978 |
| --- | --- | --- | --- |
| 8 | 1.478 | -2.608 | -0.668 |
| 8 | 3.129 | -1.039 | 1.955 |
| 8 | 5.225 | 2.631 | 0.262 |
| 7 | 5.359 | -2.664 | -1.604 |
| 7 | 3.366 | -1.546 | -1.334 |
| 7 | -0.181 | -0.222 | -1.463 |
| 7 | -1.438 | 2.574 | -1.764 |
| 7 | -0.105 | 4.344 | -2.040 |
| 7 | 2.074 | 3.246 | -1.972 |
| 7 | 1.850 | 0.879 | -1.637 |
| 7 | -6.337 | 0.833 | -0.699 |
| 7 | -6.628 | -1.498 | -1.232 |
| 7 | -4.522 | -2.537 | -1.901 |
| 7 | -3.161 | -0.775 | -1.733 |
| 7 | -4.308 | 1.940 | -0.873 |
| 7 | -4.172 | 0.369 | 2.369 |
| 7 | -1.873 | -1.443 | 1.420 |
| 7 | -2.313 | -3.551 | 0.834 |
| 7 | -4.717 | -3.607 | 1.255 |
| 7 | -5.527 | -1.485 | 2.055 |
| 7 | 1.763 | 2.325 | 1.425 |
| 7 | 0.835 | 4.526 | 1.181 |
| 7 | -1.597 | 4.494 | 1.384 |
| 7 | -1.926 | 2.315 | 1.732 |
| 7 | 0.505 | 0.419 | 1.817 |
| 7 | 4.199 | 0.776 | 1.112 |
| 7 | 6.468 | 0.771 | 0.718 |
| 6 | 2.765 | -5.165 | -0.457 |
| 6 | 2.683 | -2.677 | -0.940 |
| 6 | 3.454 | -3.906 | -0.887 |
| 6 | 4.759 | -3.837 | -1.229 |
| 6 | 4.696 | -1.466 | -1.661 |
| 6 | -1.381 | 3.875 | -1.924 |
| 6 | 0.501 | 0.911 | -1.618 |
| 6 | -0.116 | 2.174 | -1.770 |
| 6 | 0.730 | 3.266 | -1.936 |
| 6 | 2.541 | 2.017 | -1.809 |
| 6 | -5.021 | 0.815 | -0.986 |
| 6 | -4.448 | -0.410 | -1.394 |
| 6 | -5.312 | -1.497 | -1.490 |
| 6 | -7.041 | -0.299 | -0.837 |
| 6 | -3.253 | -2.051 | -2.022 |
| 6 | -1.373 | -2.571 | 0.972 |
| 6 | -5.652 | -2.767 | 1.686 |
| 6 | -3.512 | -3.016 | 1.220 |
| 6 | -3.219 | -1.704 | 1.578 |
| 6 | -4.311 | -0.912 | 2.002 |
| 6 | 1.828 | 3.650 | 1.223 |
| 6 | -0.354 | 3.925 | 1.366 |
| 6 | -0.578 | 2.569 | 1.584 |
| 6 | 0.563 | 1.734 | 1.608 |
| 6 | -2.494 | 3.490 | 1.603 |
| 6 | 5.294 | 1.474 | 0.666 |
| 6 | 6.548 | -0.521 | 1.164 |
| 6 | 5.469 | -1.205 | 1.604 |
| 6 | 4.187 | -0.521 | 1.583 |
| 6 | 5.523 | -2.607 | 2.128 |
| 1 | 5.407 | -4.704 | -1.223 |
| 1 | 6.341 | -2.647 | -1.836 |
| 1 | 2.822 | -0.663 | -1.405 |
| 1 | 0.331 | -1.073 | -1.257 |
| 1 | -1.196 | -0.225 | -1.451 |
| 1 | -2.233 | 4.536 | -1.963 |
| 1 | 0.169 | 5.309 | -2.150 |
| 1 | 3.619 | 1.900 | -1.810 |
| 1 | -8.096 | -0.216 | -0.594 |
| 1 | -2.432 | -2.682 | -2.325 |
| 1 | -4.781 | 2.784 | -0.599 |
| 1 | -3.329 | 1.980 | -1.139 |
| 1 | -4.813 | -3.491 | -2.049 |
| 1 | 1.922 | -5.386 | -1.114 |
| 1 | 2.373 | -5.059 | 0.556 |
| 1 | 3.458 | -6.005 | -0.478 |
| 1 | 6.527 | -3.017 | 2.016 |
| 1 | 5.249 | -2.633 | 3.184 |
| 1 | 4.820 | -3.245 | 1.590 |
| 1 | -2.150 | -4.491 | 0.505 |
| 1 | -3.313 | 0.875 | 2.177 |
| 1 | -5.013 | 0.896 | 2.540 |
| 1 | -0.337 | -2.738 | 0.716 |
| 1 | -6.659 | -3.167 | 1.745 |
| 1 | 2.829 | 4.038 | 1.070 |
| 1 | -1.814 | 5.472 | 1.257 |
| 1 | -3.554 | 3.679 | 1.658 |
| 1 | -0.388 | -0.064 | 1.805 |
| 1 | 1.365 | -0.118 | 1.788 |
| 1 | 3.311 | 1.309 | 1.163 |
| 1 | 7.293 | 1.246 | 0.385 |
| 1 | 7.540 | -0.953 | 1.145 |

G4

| 8 | -2.130 | -0.973 | 0.000 |
| --- | --- | --- | --- |
| 8 | 0.973 | -2.129 | 0.000 |
| 8 | -0.973 | 2.129 | 0.000 |
| 8 | 2.130 | 0.973 | 0.000 |
| 7 | -4.887 | 2.702 | 0.000 |
| 7 | -5.940 | 0.659 | 0.000 |
| 7 | -3.566 | 0.802 | 0.000 |
| 7 | -4.693 | -2.702 | 0.000 |
| 7 | -6.657 | -1.655 | 0.000 |
| 7 | -2.702 | -4.886 | 0.000 |
| 7 | -0.659 | -5.939 | 0.000 |
| 7 | -0.802 | -3.565 | 0.000 |
| 7 | 2.702 | -4.692 | 0.000 |
| 7 | 1.655 | -6.655 | 0.000 |
| 7 | 2.702 | 4.886 | 0.000 |
| 7 | 0.659 | 5.939 | 0.000 |
| 7 | 0.802 | 3.565 | 0.000 |
| 7 | -2.702 | 4.692 | 0.000 |
| 7 | -1.655 | 6.656 | 0.000 |
| 7 | 4.887 | -2.702 | 0.000 |
| 7 | 5.940 | -0.660 | 0.000 |
| 7 | 3.566 | -0.802 | 0.000 |
| 7 | 4.693 | 2.702 | 0.000 |
| 7 | 6.657 | 1.654 | 0.000 |
| 6 | -4.821 | 1.368 | 0.000 |
| 6 | -3.299 | -0.566 | 0.000 |
| 6 | -4.498 | -1.335 | 0.000 |
| 6 | -5.715 | -0.667 | 0.000 |
| 6 | -5.991 | -2.852 | 0.000 |
| 6 | -1.368 | -4.819 | 0.000 |
| 6 | 0.566 | -3.298 | 0.000 |
| 6 | 1.335 | -4.497 | 0.000 |
| 6 | 0.667 | -5.714 | 0.000 |
| 6 | 2.852 | -5.990 | 0.000 |
| 6 | 1.368 | 4.819 | 0.000 |
| 6 | -0.566 | 3.298 | 0.000 |
| 6 | -1.335 | 4.497 | 0.000 |
| 6 | -0.667 | 5.714 | 0.000 |
| 6 | -2.852 | 5.990 | 0.000 |
| 6 | 4.821 | -1.368 | 0.000 |
| 6 | 3.300 | 0.566 | 0.000 |
| 6 | 4.498 | 1.335 | 0.000 |
| 6 | 5.715 | 0.667 | 0.000 |
| 6 | 5.992 | 2.852 | 0.000 |
| 1 | -5.806 | 3.109 | 0.000 |
| 1 | -4.070 | 3.314 | 0.000 |
| 1 | -6.518 | -3.793 | 0.000 |
| 1 | -2.737 | 1.406 | 0.000 |
| 1 | -3.109 | -5.805 | 0.000 |
| 1 | -3.315 | -4.069 | 0.000 |
| 1 | 3.793 | -6.517 | 0.000 |
| 1 | -1.406 | -2.736 | 0.000 |
| 1 | 3.109 | 5.805 | 0.000 |
| 1 | 3.315 | 4.069 | 0.000 |
| 1 | -3.793 | 6.517 | 0.000 |
| 1 | 1.406 | 2.736 | 0.000 |
| 1 | 5.806 | -3.109 | 0.000 |
| 1 | 4.070 | -3.315 | 0.000 |
| 1 | 6.518 | 3.793 | 0.000 |
| 1 | 2.737 | -1.406 | 0.000 |
| 1 | 7.657 | 1.527 | 0.000 |
| 1 | 1.527 | -7.656 | 0.000 |
| 1 | -7.657 | -1.527 | 0.000 |
| 1 | -1.527 | 7.656 | 0.000 |

G4∙∙∙Li^+^

| 8 | 1.751 | 0.719 | -0.688 |
| --- | --- | --- | --- |
| 8 | -0.724 | 1.745 | 0.682 |
| 8 | 0.722 | -1.745 | 0.690 |
| 8 | -1.750 | -0.719 | -0.686 |
| 7 | 4.654 | -1.944 | 1.660 |
| 7 | 5.579 | -0.059 | 0.695 |
| 7 | 3.271 | -0.546 | 0.453 |
| 7 | 4.135 | 2.604 | -1.231 |
| 7 | 6.126 | 2.024 | -0.414 |
| 7 | 1.949 | 4.652 | -1.650 |
| 7 | 0.059 | 5.575 | -0.691 |
| 7 | 0.548 | 3.267 | -0.448 |
| 7 | -2.610 | 4.126 | 1.223 |
| 7 | -2.031 | 6.118 | 0.408 |
| 7 | -1.944 | -4.643 | -1.659 |
| 7 | -0.058 | -5.571 | -0.696 |
| 7 | -0.546 | -3.264 | -0.446 |
| 7 | 2.608 | -4.130 | 1.229 |
| 7 | 2.028 | -6.119 | 0.407 |
| 7 | -4.655 | 1.942 | 1.662 |
| 7 | -5.579 | 0.055 | 0.697 |
| 7 | -3.271 | 0.544 | 0.454 |
| 7 | -4.136 | -2.605 | -1.233 |
| 7 | -6.126 | -2.026 | -0.413 |
| 6 | 4.532 | -0.834 | 0.920 |
| 6 | 2.932 | 0.559 | -0.302 |
| 6 | 4.037 | 1.409 | -0.545 |
| 6 | 5.275 | 1.034 | -0.026 |
| 6 | 5.395 | 2.937 | -1.128 |
| 6 | 0.836 | 4.529 | -0.913 |
| 6 | -0.560 | 2.927 | 0.302 |
| 6 | -1.411 | 4.031 | 0.544 |
| 6 | -1.037 | 5.269 | 0.026 |
| 6 | -2.945 | 5.385 | 1.117 |
| 6 | -0.833 | -4.524 | -0.918 |
| 6 | 0.560 | -2.926 | 0.306 |
| 6 | 1.410 | -4.032 | 0.547 |
| 6 | 1.036 | -5.267 | 0.024 |
| 6 | 2.942 | -5.389 | 1.120 |
| 6 | -4.532 | 0.831 | 0.921 |
| 6 | -2.932 | -0.561 | -0.302 |
| 6 | -4.037 | -1.412 | -0.544 |
| 6 | -5.274 | -1.037 | -0.024 |
| 6 | -5.396 | -2.938 | -1.129 |
| 3 | 0.001 | 0.003 | 0.001 |
| 1 | 5.594 | -2.216 | 1.900 |
| 1 | 3.939 | -2.670 | 1.625 |
| 1 | 5.848 | 3.824 | -1.541 |
| 1 | 2.485 | -1.160 | 0.686 |
| 1 | 2.222 | 5.592 | -1.886 |
| 1 | 2.674 | 3.937 | -1.616 |
| 1 | -3.835 | 5.836 | 1.527 |
| 1 | 1.165 | 2.482 | -0.678 |
| 1 | -2.214 | -5.582 | -1.904 |
| 1 | -2.670 | -3.929 | -1.622 |
| 1 | 3.831 | -5.842 | 1.531 |
| 1 | -1.160 | -2.476 | -0.677 |
| 1 | -5.595 | 2.212 | 1.900 |
| 1 | -3.943 | 2.670 | 1.622 |
| 1 | -5.849 | -3.824 | -1.544 |
| 1 | -2.485 | 1.160 | 0.683 |
| 1 | -7.114 | -2.078 | -0.209 |
| 1 | -2.085 | 7.106 | 0.204 |
| 1 | 7.114 | 2.076 | -0.211 |
| 1 | 2.081 | -7.106 | 0.200 |

G4∙∙∙Na^+^

| 11 | 0.000 | -0.001 | 0.016 |
| --- | --- | --- | --- |
| 8 | 1.393 | -1.833 | 0.002 |
| 8 | -1.834 | -1.391 | -0.006 |
| 8 | 1.832 | 1.392 | -0.006 |
| 8 | -1.393 | 1.831 | 0.001 |
| 7 | 5.609 | -0.016 | -0.123 |
| 7 | 5.506 | -2.314 | -0.146 |
| 7 | 3.522 | -1.008 | -0.058 |
| 7 | 2.747 | -4.602 | -0.084 |
| 7 | 4.972 | -4.675 | -0.164 |
| 7 | -0.016 | -5.607 | 0.097 |
| 7 | -2.315 | -5.505 | 0.143 |
| 7 | -1.009 | -3.520 | 0.046 |
| 7 | -4.604 | -2.746 | 0.096 |
| 7 | -4.676 | -4.970 | 0.177 |
| 7 | 0.018 | 5.610 | 0.093 |
| 7 | 2.316 | 5.506 | 0.142 |
| 7 | 1.009 | 3.522 | 0.045 |
| 7 | 4.602 | 2.745 | 0.098 |
| 7 | 4.676 | 4.969 | 0.178 |
| 7 | -5.610 | 0.016 | -0.123 |
| 7 | -5.506 | 2.315 | -0.145 |
| 7 | -3.523 | 1.007 | -0.059 |
| 7 | -2.746 | 4.602 | -0.084 |
| 7 | -4.971 | 4.676 | -0.162 |
| 6 | 4.893 | -1.141 | -0.108 |
| 6 | 2.618 | -2.059 | -0.042 |
| 6 | 3.262 | -3.321 | -0.083 |
| 6 | 4.652 | -3.350 | -0.132 |
| 6 | 3.797 | -5.380 | -0.133 |
| 6 | -1.142 | -4.891 | 0.095 |
| 6 | -2.060 | -2.617 | 0.039 |
| 6 | -3.322 | -3.260 | 0.087 |
| 6 | -3.351 | -4.651 | 0.136 |
| 6 | -5.380 | -3.796 | 0.150 |
| 6 | 1.142 | 4.893 | 0.093 |
| 6 | 2.059 | 2.618 | 0.039 |
| 6 | 3.322 | 3.260 | 0.087 |
| 6 | 3.351 | 4.650 | 0.136 |
| 6 | 5.380 | 3.794 | 0.152 |
| 6 | -4.894 | 1.141 | -0.107 |
| 6 | -2.618 | 2.058 | -0.043 |
| 6 | -3.261 | 3.321 | -0.083 |
| 6 | -4.651 | 3.350 | -0.132 |
| 6 | -3.796 | 5.379 | -0.132 |
| 1 | 6.610 | -0.113 | -0.130 |
| 1 | 5.195 | 0.914 | -0.042 |
| 1 | 3.782 | -6.458 | -0.148 |
| 1 | 3.108 | -0.071 | -0.037 |
| 1 | -0.113 | -6.608 | 0.116 |
| 1 | 0.915 | -5.192 | 0.034 |
| 1 | -6.458 | -3.781 | 0.172 |
| 1 | -0.072 | -3.107 | 0.017 |
| 1 | 0.115 | 6.611 | 0.114 |
| 1 | -0.913 | 5.195 | 0.032 |
| 1 | 6.458 | 3.778 | 0.175 |
| 1 | 0.072 | 3.109 | 0.015 |
| 1 | -6.611 | 0.114 | -0.129 |
| 1 | -5.196 | -0.914 | -0.043 |
| 1 | -3.780 | 6.457 | -0.147 |
| 1 | -3.109 | 0.070 | -0.037 |
| 1 | 5.067 | 5.899 | 0.220 |
| 1 | 5.902 | -5.066 | -0.204 |
| 1 | -5.066 | -5.900 | 0.219 |
| 1 | -5.901 | 5.066 | -0.201 |

G4∙∙∙K^+^

| 8 | -1.243 | -1.972 | 0.138 |
| --- | --- | --- | --- |
| 8 | -1.974 | 1.242 | 0.125 |
| 8 | 1.977 | -1.244 | 0.156 |
| 8 | 1.245 | 1.973 | 0.137 |
| 7 | 1.972 | -5.246 | -0.096 |
| 7 | -0.207 | -5.971 | -0.205 |
| 7 | 0.295 | -3.656 | 0.002 |
| 7 | -3.338 | -4.226 | -0.103 |
| 7 | -2.602 | -6.321 | -0.277 |
| 7 | -5.247 | -1.975 | -0.077 |
| 7 | -5.976 | 0.205 | -0.162 |
| 7 | -3.658 | -0.296 | 0.006 |
| 7 | -4.233 | 3.337 | -0.068 |
| 7 | -6.330 | 2.600 | -0.212 |
| 7 | 5.247 | 1.972 | -0.104 |
| 7 | 5.975 | -0.208 | -0.187 |
| 7 | 3.659 | 0.295 | 0.009 |
| 7 | 4.233 | -3.338 | -0.061 |
| 7 | 6.327 | -2.603 | -0.235 |
| 7 | -1.972 | 5.242 | -0.141 |
| 7 | 0.208 | 5.971 | -0.214 |
| 7 | -0.294 | 3.655 | -0.016 |
| 7 | 3.339 | 4.231 | -0.072 |
| 7 | 2.603 | 6.325 | -0.253 |
| 6 | 0.665 | -4.979 | -0.105 |
| 6 | -1.014 | -3.190 | 0.026 |
| 6 | -1.957 | -4.245 | -0.083 |
| 6 | -1.483 | -5.547 | -0.191 |
| 6 | -3.683 | -5.481 | -0.221 |
| 6 | -4.983 | -0.667 | -0.083 |
| 6 | -3.193 | 1.013 | 0.031 |
| 6 | -4.251 | 1.956 | -0.057 |
| 6 | -5.554 | 1.481 | -0.146 |
| 6 | -5.490 | 3.682 | -0.163 |
| 6 | 4.982 | 0.664 | -0.098 |
| 6 | 3.195 | -1.015 | 0.045 |
| 6 | 4.251 | -1.958 | -0.054 |
| 6 | 5.553 | -1.483 | -0.162 |
| 6 | 5.488 | -3.684 | -0.171 |
| 6 | -0.664 | 4.978 | -0.127 |
| 6 | 1.016 | 3.191 | 0.025 |
| 6 | 1.959 | 4.248 | -0.071 |
| 6 | 1.484 | 5.549 | -0.183 |
| 6 | 3.685 | 5.486 | -0.183 |
| 1 | 2.240 | -6.210 | -0.202 |
| 1 | 2.696 | -4.527 | -0.063 |
| 1 | -4.694 | -5.854 | -0.270 |
| 1 | 1.021 | -2.935 | 0.060 |
| 1 | -6.212 | -2.243 | -0.173 |
| 1 | -4.527 | -2.698 | -0.069 |
| 1 | -5.865 | 4.692 | -0.201 |
| 1 | -2.936 | -1.021 | 0.049 |
| 1 | 6.212 | 2.240 | -0.204 |
| 1 | 4.528 | 2.696 | -0.070 |
| 1 | 5.863 | -4.695 | -0.210 |
| 1 | 2.937 | 1.020 | 0.058 |
| 1 | -2.240 | 6.207 | -0.235 |
| 1 | -2.695 | 4.524 | -0.093 |
| 1 | 4.695 | 5.861 | -0.218 |
| 1 | -1.019 | 2.933 | 0.033 |
| 1 | 2.630 | 7.330 | -0.338 |
| 1 | -7.336 | 2.627 | -0.283 |
| 1 | -2.630 | -7.326 | -0.365 |
| 1 | 7.333 | -2.630 | -0.319 |
| 19 | -0.004 | 0.004 | 1.460 |

G4-G4

| 8 | 0.626 | -2.230 | 1.456 |
| --- | --- | --- | --- |
| 8 | 2.236 | 0.631 | 1.452 |
| 8 | -2.234 | -0.625 | 1.451 |
| 8 | -0.626 | 2.237 | 1.452 |
| 7 | -3.391 | -4.444 | 1.752 |
| 7 | -1.513 | -5.771 | 1.723 |
| 7 | -1.330 | -3.404 | 1.591 |
| 7 | 1.981 | -5.001 | 1.502 |
| 7 | 0.678 | -6.801 | 1.632 |
| 7 | 4.445 | -3.387 | 1.770 |
| 7 | 5.774 | -1.510 | 1.738 |
| 7 | 3.408 | -1.326 | 1.599 |
| 7 | 5.008 | 1.984 | 1.499 |
| 7 | 6.806 | 0.680 | 1.640 |
| 7 | -4.445 | 3.393 | 1.765 |
| 7 | -5.773 | 1.515 | 1.740 |
| 7 | -3.406 | 1.332 | 1.596 |
| 7 | -5.005 | -1.978 | 1.506 |
| 7 | -6.804 | -0.676 | 1.648 |
| 7 | 3.392 | 4.448 | 1.754 |
| 7 | 1.514 | 5.776 | 1.724 |
| 7 | 1.331 | 3.409 | 1.591 |
| 7 | -1.980 | 5.008 | 1.498 |
| 7 | -0.677 | 6.807 | 1.631 |
| 6 | -2.061 | -4.566 | 1.681 |
| 6 | 0.060 | -3.329 | 1.529 |
| 6 | 0.656 | -4.620 | 1.564 |
| 6 | -0.171 | -5.733 | 1.646 |
| 6 | 1.953 | -6.307 | 1.542 |
| 6 | 4.570 | -2.057 | 1.694 |
| 6 | 3.334 | 0.064 | 1.530 |
| 6 | 4.626 | 0.659 | 1.565 |
| 6 | 5.737 | -0.168 | 1.655 |
| 6 | 6.314 | 1.955 | 1.544 |
| 6 | -4.568 | 2.063 | 1.692 |
| 6 | -3.332 | -0.058 | 1.530 |
| 6 | -4.624 | -0.654 | 1.569 |
| 6 | -5.736 | 0.173 | 1.659 |
| 6 | -6.311 | -1.950 | 1.552 |
| 6 | 2.062 | 4.572 | 1.681 |
| 6 | -0.059 | 3.335 | 1.527 |
| 6 | -0.655 | 4.627 | 1.561 |
| 6 | 0.172 | 5.739 | 1.646 |
| 6 | -1.951 | 6.314 | 1.539 |
| 1 | -3.921 | -5.298 | 1.720 |
| 1 | -3.876 | -3.556 | 1.611 |
| 1 | 2.812 | -6.957 | 1.511 |
| 1 | -1.814 | -2.501 | 1.555 |
| 1 | 5.299 | -3.918 | 1.740 |
| 1 | 3.559 | -3.871 | 1.625 |
| 1 | 6.965 | 2.814 | 1.513 |
| 1 | 2.504 | -1.810 | 1.562 |
| 1 | -5.299 | 3.924 | 1.738 |
| 1 | -3.558 | 3.877 | 1.619 |
| 1 | -6.962 | -2.809 | 1.522 |
| 1 | -2.503 | 1.816 | 1.556 |
| 1 | 3.923 | 5.302 | 1.724 |
| 1 | 3.877 | 3.561 | 1.613 |
| 1 | -2.810 | 6.965 | 1.508 |
| 1 | 1.815 | 2.506 | 1.554 |
| 1 | -0.414 | 7.780 | 1.677 |
| 1 | 7.779 | 0.418 | 1.690 |
| 1 | 0.416 | -7.774 | 1.677 |
| 1 | -7.777 | -0.413 | 1.697 |
| 8 | -1.130 | -2.026 | -1.491 |
| 8 | 2.022 | -1.134 | -1.488 |
| 8 | -2.024 | 1.128 | -1.485 |
| 8 | 1.129 | 2.020 | -1.491 |
| 7 | -5.542 | -0.765 | -1.755 |
| 7 | -5.146 | -3.031 | -1.714 |
| 7 | -3.347 | -1.480 | -1.607 |
| 7 | -2.122 | -4.946 | -1.509 |
| 7 | -4.316 | -5.306 | -1.618 |
| 7 | 0.764 | -5.546 | -1.760 |
| 7 | 3.030 | -5.148 | -1.710 |
| 7 | 1.477 | -3.350 | -1.610 |
| 7 | 4.943 | -2.125 | -1.490 |
| 7 | 5.304 | -4.319 | -1.600 |
| 7 | -0.765 | 5.539 | -1.772 |
| 7 | -3.031 | 5.142 | -1.718 |
| 7 | -1.478 | 3.344 | -1.612 |
| 7 | -4.944 | 2.120 | -1.492 |
| 7 | -5.305 | 4.313 | -1.608 |
| 7 | 5.541 | 0.760 | -1.764 |
| 7 | 5.144 | 3.026 | -1.720 |
| 7 | 3.346 | 1.474 | -1.611 |
| 7 | 2.121 | 4.940 | -1.506 |
| 7 | 4.315 | 5.301 | -1.620 |
| 6 | -4.685 | -1.790 | -1.683 |
| 6 | -2.307 | -2.406 | -1.550 |
| 6 | -2.795 | -3.743 | -1.572 |
| 6 | -4.166 | -3.950 | -1.642 |
| 6 | -3.063 | -5.853 | -1.536 |
| 6 | 1.788 | -4.688 | -1.685 |
| 6 | 2.403 | -2.311 | -1.546 |
| 6 | 3.740 | -2.798 | -1.561 |
| 6 | 3.947 | -4.169 | -1.631 |
| 6 | 5.850 | -3.066 | -1.513 |
| 6 | -1.790 | 4.682 | -1.692 |
| 6 | -2.405 | 2.305 | -1.546 |
| 6 | -3.741 | 2.792 | -1.562 |
| 6 | -3.949 | 4.163 | -1.637 |
| 6 | -5.851 | 3.060 | -1.518 |
| 6 | 4.684 | 1.785 | -1.689 |
| 6 | 2.306 | 2.401 | -1.551 |
| 6 | 2.794 | 3.737 | -1.572 |
| 6 | 4.164 | 3.944 | -1.645 |
| 6 | 3.062 | 5.847 | -1.534 |
| 1 | -6.520 | -0.995 | -1.704 |
| 1 | -5.257 | 0.204 | -1.615 |
| 1 | -2.911 | -6.920 | -1.500 |
| 1 | -3.054 | -0.497 | -1.578 |
| 1 | 0.994 | -6.524 | -1.708 |
| 1 | -0.206 | -5.260 | -1.626 |
| 1 | 6.917 | -2.914 | -1.469 |
| 1 | 0.495 | -3.057 | -1.585 |
| 1 | -0.995 | 6.517 | -1.721 |
| 1 | 0.205 | 5.255 | -1.631 |
| 1 | -6.918 | 2.909 | -1.476 |
| 1 | -0.496 | 3.051 | -1.587 |
| 1 | 6.519 | 0.991 | -1.712 |
| 1 | 5.256 | -0.209 | -1.620 |
| 1 | 2.910 | 6.914 | -1.496 |
| 1 | 3.053 | 0.492 | -1.582 |
| 1 | 5.187 | 5.806 | -1.656 |
| 1 | 5.809 | -5.192 | -1.633 |
| 1 | -5.189 | -5.811 | -1.653 |
| 1 | -5.810 | 5.186 | -1.645 |

G4∙∙∙Li^+^∙∙∙G4

| 8 | 1.994 | 1.201 | 1.469 |
| --- | --- | --- | --- |
| 8 | -1.201 | 1.993 | 1.470 |
| 8 | 1.202 | -1.992 | 1.471 |
| 8 | -1.993 | -1.200 | 1.469 |
| 7 | 5.179 | -2.102 | 1.739 |
| 7 | 5.970 | 0.057 | 1.717 |
| 7 | 3.636 | -0.385 | 1.597 |
| 7 | 4.313 | 3.227 | 1.493 |
| 7 | 6.392 | 2.441 | 1.619 |
| 7 | 2.103 | 5.177 | 1.739 |
| 7 | -0.056 | 5.969 | 1.723 |
| 7 | 0.386 | 3.635 | 1.599 |
| 7 | -3.226 | 4.313 | 1.506 |
| 7 | -2.440 | 6.392 | 1.633 |
| 7 | -2.103 | -5.177 | 1.739 |
| 7 | 0.056 | -5.968 | 1.724 |
| 7 | -0.386 | -3.634 | 1.599 |
| 7 | 3.226 | -4.311 | 1.506 |
| 7 | 2.440 | -6.390 | 1.633 |
| 7 | -5.178 | 2.103 | 1.740 |
| 7 | -5.970 | -0.056 | 1.718 |
| 7 | -3.635 | 0.386 | 1.598 |
| 7 | -4.312 | -3.226 | 1.493 |
| 7 | -6.392 | -2.440 | 1.620 |
| 6 | 4.950 | -0.786 | 1.679 |
| 6 | 3.203 | 0.936 | 1.535 |
| 6 | 4.293 | 1.849 | 1.559 |
| 6 | 5.584 | 1.342 | 1.639 |
| 6 | 5.581 | 3.541 | 1.528 |
| 6 | 0.787 | 4.949 | 1.681 |
| 6 | -0.936 | 3.203 | 1.539 |
| 6 | -1.848 | 4.293 | 1.568 |
| 6 | -1.341 | 5.583 | 1.649 |
| 6 | -3.541 | 5.581 | 1.544 |
| 6 | -0.787 | -4.948 | 1.681 |
| 6 | 0.936 | -3.202 | 1.540 |
| 6 | 1.848 | -4.292 | 1.568 |
| 6 | 1.341 | -5.582 | 1.649 |
| 6 | 3.541 | -5.580 | 1.544 |
| 6 | -4.950 | 0.788 | 1.679 |
| 6 | -3.203 | -0.935 | 1.536 |
| 6 | -4.293 | -1.848 | 1.559 |
| 6 | -5.583 | -1.341 | 1.640 |
| 6 | -5.581 | -3.540 | 1.528 |
| 1 | 6.141 | -2.394 | 1.723 |
| 1 | 4.446 | -2.803 | 1.623 |
| 1 | 5.984 | 4.541 | 1.494 |
| 1 | 2.892 | -1.091 | 1.571 |
| 1 | 2.395 | 6.140 | 1.723 |
| 1 | 2.803 | 4.445 | 1.618 |
| 1 | -4.540 | 5.984 | 1.513 |
| 1 | 1.091 | 2.891 | 1.570 |
| 1 | -2.395 | -6.139 | 1.723 |
| 1 | -2.803 | -4.444 | 1.618 |
| 1 | 4.541 | -5.983 | 1.514 |
| 1 | -1.091 | -2.890 | 1.570 |
| 1 | -6.141 | 2.395 | 1.724 |
| 1 | -4.445 | 2.804 | 1.624 |
| 1 | -5.984 | -4.540 | 1.494 |
| 1 | -2.891 | 1.092 | 1.571 |
| 1 | -7.400 | -2.442 | 1.661 |
| 1 | -2.442 | 7.400 | 1.676 |
| 1 | 7.400 | 2.443 | 1.661 |
| 1 | 2.443 | -7.398 | 1.676 |
| 8 | 2.052 | -0.594 | -1.473 |
| 8 | 0.593 | 2.052 | -1.471 |
| 8 | -0.593 | -2.053 | -1.470 |
| 8 | -2.053 | 0.593 | -1.472 |
| 7 | 2.188 | -5.182 | -1.754 |
| 7 | 4.228 | -4.123 | -1.708 |
| 7 | 2.205 | -2.875 | -1.594 |
| 7 | 5.141 | -0.673 | -1.461 |
| 7 | 6.142 | -2.654 | -1.598 |
| 7 | 5.180 | 2.187 | -1.770 |
| 7 | 4.122 | 4.227 | -1.719 |
| 7 | 2.874 | 2.205 | -1.601 |
| 7 | 0.673 | 5.141 | -1.456 |
| 7 | 2.653 | 6.142 | -1.600 |
| 7 | -5.180 | -2.188 | -1.768 |
| 7 | -4.122 | -4.228 | -1.718 |
| 7 | -2.874 | -2.206 | -1.600 |
| 7 | -0.673 | -5.143 | -1.456 |
| 7 | -2.654 | -6.143 | -1.600 |
| 7 | -2.188 | 5.181 | -1.757 |
| 7 | -4.228 | 4.122 | -1.710 |
| 7 | -2.206 | 2.874 | -1.595 |
| 7 | -5.142 | 0.672 | -1.461 |
| 7 | -6.142 | 2.653 | -1.598 |
| 6 | 2.907 | -4.061 | -1.678 |
| 6 | 2.773 | -1.614 | -1.525 |
| 6 | 4.191 | -1.670 | -1.535 |
| 6 | 4.804 | -2.916 | -1.620 |
| 6 | 6.288 | -1.297 | -1.498 |
| 6 | 4.059 | 2.906 | -1.689 |
| 6 | 1.613 | 2.773 | -1.527 |
| 6 | 1.669 | 4.191 | -1.535 |
| 6 | 2.915 | 4.804 | -1.625 |
| 6 | 1.298 | 6.288 | -1.495 |
| 6 | -4.060 | -2.907 | -1.687 |
| 6 | -1.613 | -2.774 | -1.526 |
| 6 | -1.669 | -4.192 | -1.534 |
| 6 | -2.916 | -4.805 | -1.625 |
| 6 | -1.298 | -6.289 | -1.495 |
| 6 | -2.907 | 4.060 | -1.679 |
| 6 | -2.773 | 1.613 | -1.525 |
| 6 | -4.191 | 1.669 | -1.534 |
| 6 | -4.805 | 2.915 | -1.621 |
| 6 | -6.289 | 1.297 | -1.498 |
| 3 | 0.000 | -0.001 | -1.354 |
| 1 | 2.696 | -6.049 | -1.714 |
| 1 | 1.175 | -5.172 | -1.613 |
| 1 | 7.260 | -0.831 | -1.457 |
| 1 | 1.182 | -2.882 | -1.572 |
| 1 | 6.047 | 2.695 | -1.732 |
| 1 | 5.170 | 1.174 | -1.626 |
| 1 | 0.831 | 7.260 | -1.450 |
| 1 | 2.880 | 1.182 | -1.580 |
| 1 | -6.047 | -2.696 | -1.731 |
| 1 | -5.171 | -1.175 | -1.625 |
| 1 | -0.832 | -7.261 | -1.451 |
| 1 | -2.880 | -1.183 | -1.579 |
| 1 | -2.696 | 6.048 | -1.716 |
| 1 | -1.175 | 5.171 | -1.614 |
| 1 | -7.260 | 0.830 | -1.456 |
| 1 | -1.183 | 2.880 | -1.573 |
| 1 | -6.885 | 3.335 | -1.639 |
| 1 | 3.335 | 6.884 | -1.643 |
| 1 | 6.885 | -3.336 | -1.638 |
| 1 | -3.336 | -6.886 | -1.643 |

G4∙∙∙Na^+^∙∙∙G4

| 8 | 1.385 | -1.765 | 1.347 |
| --- | --- | --- | --- |
| 8 | 1.767 | 1.387 | 1.343 |
| 8 | -1.767 | -1.387 | 1.343 |
| 8 | -1.385 | 1.766 | 1.345 |
| 7 | -1.371 | -5.424 | 1.741 |
| 7 | 0.885 | -5.863 | 1.750 |
| 7 | 0.081 | -3.635 | 1.538 |
| 7 | 3.754 | -3.739 | 1.469 |
| 7 | 3.303 | -5.908 | 1.675 |
| 7 | 5.425 | -1.368 | 1.740 |
| 7 | 5.866 | 0.887 | 1.740 |
| 7 | 3.636 | 0.083 | 1.536 |
| 7 | 3.742 | 3.756 | 1.454 |
| 7 | 5.911 | 3.305 | 1.655 |
| 7 | -5.426 | 1.368 | 1.737 |
| 7 | -5.866 | -0.888 | 1.740 |
| 7 | -3.637 | -0.084 | 1.534 |
| 7 | -3.742 | -3.757 | 1.455 |
| 7 | -5.911 | -3.305 | 1.658 |
| 7 | 1.370 | 5.424 | 1.746 |
| 7 | -0.886 | 5.864 | 1.752 |
| 7 | -0.082 | 3.635 | 1.539 |
| 7 | -3.755 | 3.739 | 1.466 |
| 7 | -3.304 | 5.908 | 1.673 |
| 6 | -0.109 | -4.992 | 1.671 |
| 6 | 1.315 | -3.002 | 1.461 |
| 6 | 2.389 | -3.930 | 1.529 |
| 6 | 2.091 | -5.281 | 1.659 |
| 6 | 4.263 | -4.939 | 1.556 |
| 6 | 4.994 | -0.107 | 1.667 |
| 6 | 3.004 | 1.317 | 1.455 |
| 6 | 3.932 | 2.392 | 1.518 |
| 6 | 5.284 | 2.093 | 1.646 |
| 6 | 4.943 | 4.265 | 1.535 |
| 6 | -4.995 | 0.106 | 1.665 |
| 6 | -3.004 | -1.318 | 1.454 |
| 6 | -3.933 | -2.392 | 1.518 |
| 6 | -5.284 | -2.094 | 1.647 |
| 6 | -4.943 | -4.266 | 1.538 |
| 6 | 0.108 | 4.993 | 1.674 |
| 6 | -1.316 | 3.002 | 1.460 |
| 6 | -2.390 | 3.930 | 1.528 |
| 6 | -2.092 | 5.282 | 1.659 |
| 6 | -4.264 | 4.940 | 1.552 |
| 1 | -1.507 | -6.421 | 1.757 |
| 1 | -2.172 | -4.807 | 1.594 |
| 1 | 5.314 | -5.180 | 1.542 |
| 1 | -0.727 | -3.006 | 1.476 |
| 1 | 6.422 | -1.505 | 1.759 |
| 1 | 4.807 | -2.170 | 1.601 |
| 1 | 5.184 | 5.316 | 1.517 |
| 1 | 3.007 | -0.724 | 1.477 |
| 1 | -6.423 | 1.504 | 1.754 |
| 1 | -4.809 | 2.170 | 1.596 |
| 1 | -5.184 | -5.317 | 1.521 |
| 1 | -3.008 | 0.724 | 1.476 |
| 1 | 1.505 | 6.421 | 1.762 |
| 1 | 2.171 | 4.808 | 1.597 |
| 1 | -5.315 | 5.181 | 1.537 |
| 1 | 0.726 | 3.007 | 1.478 |
| 1 | -6.905 | -3.465 | 1.734 |
| 1 | 3.463 | -6.901 | 1.754 |
| 1 | 6.904 | 3.464 | 1.732 |
| 1 | -3.463 | 6.901 | 1.752 |
| 11 | -0.001 | 0.000 | -0.004 |
| 8 | -0.265 | -2.224 | -1.345 |
| 8 | 2.227 | -0.267 | -1.351 |
| 8 | -2.227 | 0.266 | -1.352 |
| 8 | 0.266 | 2.224 | -1.345 |
| 7 | -4.798 | -2.879 | -1.751 |
| 7 | -3.507 | -4.780 | -1.752 |
| 7 | -2.507 | -2.632 | -1.544 |
| 7 | 0.024 | -5.296 | -1.451 |
| 7 | -1.824 | -6.517 | -1.663 |
| 7 | 2.882 | -4.802 | -1.737 |
| 7 | 4.784 | -3.510 | -1.739 |
| 7 | 2.635 | -2.510 | -1.538 |
| 7 | 5.298 | 0.023 | -1.460 |
| 7 | 6.519 | -1.826 | -1.660 |
| 7 | -2.881 | 4.801 | -1.737 |
| 7 | -4.783 | 3.510 | -1.743 |
| 7 | -2.635 | 2.509 | -1.540 |
| 7 | -5.298 | -0.022 | -1.458 |
| 7 | -6.519 | 1.826 | -1.662 |
| 7 | 4.799 | 2.879 | -1.753 |
| 7 | 3.508 | 4.780 | -1.750 |
| 7 | 2.508 | 2.632 | -1.543 |
| 7 | -0.023 | 5.296 | -1.450 |
| 7 | 1.825 | 6.517 | -1.661 |
| 6 | -3.599 | -3.461 | -1.677 |
| 6 | -1.186 | -3.053 | -1.458 |
| 6 | -1.078 | -4.469 | -1.520 |
| 6 | -2.241 | -5.218 | -1.654 |
| 6 | -0.461 | -6.506 | -1.537 |
| 6 | 3.465 | -3.602 | -1.666 |
| 6 | 3.056 | -1.188 | -1.460 |
| 6 | 4.471 | -1.080 | -1.523 |
| 6 | 5.221 | -2.244 | -1.649 |
| 6 | 6.508 | -0.462 | -1.542 |
| 6 | -3.464 | 3.601 | -1.668 |
| 6 | -3.055 | 1.188 | -1.461 |
| 6 | -4.471 | 1.080 | -1.522 |
| 6 | -5.220 | 2.244 | -1.651 |
| 6 | -6.509 | 0.463 | -1.542 |
| 6 | 3.600 | 3.461 | -1.677 |
| 6 | 1.187 | 3.053 | -1.458 |
| 6 | 1.079 | 4.469 | -1.519 |
| 6 | 2.242 | 5.218 | -1.653 |
| 6 | 0.462 | 6.506 | -1.535 |
| 1 | -5.597 | -3.491 | -1.764 |
| 1 | -4.932 | -1.876 | -1.602 |
| 1 | 0.115 | -7.418 | -1.517 |
| 1 | -2.635 | -1.617 | -1.487 |
| 1 | 3.494 | -5.600 | -1.747 |
| 1 | 1.879 | -4.935 | -1.591 |
| 1 | 7.420 | 0.114 | -1.524 |
| 1 | 1.620 | -2.638 | -1.479 |
| 1 | -3.493 | 5.600 | -1.746 |
| 1 | -1.878 | 4.934 | -1.589 |
| 1 | -7.421 | -0.113 | -1.524 |
| 1 | -1.619 | 2.637 | -1.481 |
| 1 | 5.597 | 3.490 | -1.768 |
| 1 | 4.933 | 1.876 | -1.608 |
| 1 | -0.114 | 7.418 | -1.514 |
| 1 | 2.636 | 1.617 | -1.486 |
| 1 | -7.335 | 2.414 | -1.742 |
| 1 | -2.411 | -7.333 | -1.748 |
| 1 | 7.336 | -2.413 | -1.742 |
| 1 | 2.412 | 7.333 | -1.746 |

G4∙∙∙K^+^∙∙∙G4

| 8 | -0.643 | 2.229 | -1.536 |
| --- | --- | --- | --- |
| 8 | 2.229 | 0.643 | -1.536 |
| 8 | -2.230 | -0.643 | -1.536 |
| 8 | 0.643 | -2.229 | -1.536 |
| 7 | -5.232 | 2.026 | -1.725 |
| 7 | -4.319 | 4.137 | -1.694 |
| 7 | -2.928 | 2.211 | -1.611 |
| 7 | -0.932 | 5.297 | -1.529 |
| 7 | -2.984 | 6.157 | -1.609 |
| 7 | 2.026 | 5.232 | -1.726 |
| 7 | 4.137 | 4.319 | -1.695 |
| 7 | 2.210 | 2.928 | -1.612 |
| 7 | 5.296 | 0.932 | -1.530 |
| 7 | 6.156 | 2.984 | -1.610 |
| 7 | -2.026 | -5.232 | -1.725 |
| 7 | -4.137 | -4.319 | -1.694 |
| 7 | -2.211 | -2.928 | -1.611 |
| 7 | -5.297 | -0.932 | -1.529 |
| 7 | -6.157 | -2.984 | -1.609 |
| 7 | 5.232 | -2.026 | -1.726 |
| 7 | 4.319 | -4.137 | -1.695 |
| 7 | 2.928 | -2.210 | -1.612 |
| 7 | 0.932 | -5.296 | -1.530 |
| 7 | 2.984 | -6.157 | -1.610 |
| 6 | -4.161 | 2.823 | -1.670 |
| 6 | -1.706 | 2.872 | -1.573 |
| 6 | -1.867 | 4.283 | -1.582 |
| 6 | -3.154 | 4.803 | -1.634 |
| 6 | -1.637 | 6.397 | -1.543 |
| 6 | 2.823 | 4.161 | -1.670 |
| 6 | 2.872 | 1.706 | -1.574 |
| 6 | 4.283 | 1.866 | -1.583 |
| 6 | 4.803 | 3.154 | -1.635 |
| 6 | 6.397 | 1.637 | -1.544 |
| 6 | -2.823 | -4.161 | -1.670 |
| 6 | -2.873 | -1.706 | -1.573 |
| 6 | -4.283 | -1.866 | -1.582 |
| 6 | -4.803 | -3.154 | -1.633 |
| 6 | -6.397 | -1.637 | -1.543 |
| 6 | 4.160 | -2.823 | -1.671 |
| 6 | 1.706 | -2.872 | -1.574 |
| 6 | 1.866 | -4.283 | -1.583 |
| 6 | 3.154 | -4.803 | -1.634 |
| 6 | 1.637 | -6.397 | -1.544 |
| 1 | -6.131 | 2.476 | -1.681 |
| 1 | -5.179 | 1.013 | -1.615 |
| 1 | -1.241 | 7.399 | -1.510 |
| 1 | -2.872 | 1.187 | -1.597 |
| 1 | 2.476 | 6.131 | -1.682 |
| 1 | 1.013 | 5.178 | -1.616 |
| 1 | 7.399 | 1.241 | -1.511 |
| 1 | 1.187 | 2.872 | -1.597 |
| 1 | -2.476 | -6.131 | -1.681 |
| 1 | -1.013 | -5.178 | -1.616 |
| 1 | -7.400 | -1.241 | -1.509 |
| 1 | -1.188 | -2.872 | -1.597 |
| 1 | 6.130 | -2.476 | -1.683 |
| 1 | 5.178 | -1.013 | -1.617 |
| 1 | 1.241 | -7.399 | -1.510 |
| 1 | 2.872 | -1.187 | -1.597 |
| 1 | 6.852 | 3.715 | -1.633 |
| 1 | 3.715 | -6.852 | -1.632 |
| 1 | -6.852 | -3.715 | -1.631 |
| 1 | -3.715 | 6.852 | -1.631 |
| 19 | 0.000 | 0.000 | 0.001 |
| 8 | -2.034 | 1.117 | 1.534 |
| 8 | 1.117 | 2.034 | 1.534 |
| 8 | -1.116 | -2.034 | 1.534 |
| 8 | 2.035 | -1.116 | 1.534 |
| 7 | -5.124 | -2.282 | 1.727 |
| 7 | -5.979 | -0.146 | 1.696 |
| 7 | -3.632 | -0.518 | 1.611 |
| 7 | -4.413 | 3.074 | 1.529 |
| 7 | -6.470 | 2.224 | 1.612 |
| 7 | -2.282 | 5.124 | 1.726 |
| 7 | -0.146 | 5.979 | 1.695 |
| 7 | -0.518 | 3.632 | 1.611 |
| 7 | 3.074 | 4.413 | 1.528 |
| 7 | 2.224 | 6.470 | 1.611 |
| 7 | 2.282 | -5.124 | 1.726 |
| 7 | 0.146 | -5.979 | 1.695 |
| 7 | 0.518 | -3.632 | 1.611 |
| 7 | -3.074 | -4.413 | 1.529 |
| 7 | -2.224 | -6.470 | 1.611 |
| 7 | 5.124 | 2.282 | 1.725 |
| 7 | 5.979 | 0.146 | 1.695 |
| 7 | 3.632 | 0.518 | 1.611 |
| 7 | 4.413 | -3.074 | 1.528 |
| 7 | 6.470 | -2.224 | 1.610 |
| 6 | -4.935 | -0.960 | 1.671 |
| 6 | -3.240 | 0.816 | 1.572 |
| 6 | -4.353 | 1.696 | 1.582 |
| 6 | -5.630 | 1.150 | 1.635 |
| 6 | -5.690 | 3.349 | 1.545 |
| 6 | -0.960 | 4.935 | 1.671 |
| 6 | 0.816 | 3.240 | 1.572 |
| 6 | 1.696 | 4.353 | 1.581 |
| 6 | 1.150 | 5.630 | 1.634 |
| 6 | 3.349 | 5.690 | 1.544 |
| 6 | 0.960 | -4.935 | 1.670 |
| 6 | -0.816 | -3.240 | 1.572 |
| 6 | -1.696 | -4.353 | 1.582 |
| 6 | -1.150 | -5.630 | 1.635 |
| 6 | -3.349 | -5.690 | 1.545 |
| 6 | 4.935 | 0.960 | 1.670 |
| 6 | 3.240 | -0.816 | 1.572 |
| 6 | 4.354 | -1.696 | 1.581 |
| 6 | 5.630 | -1.150 | 1.634 |
| 6 | 5.691 | -3.349 | 1.543 |
| 1 | -6.076 | -2.603 | 1.684 |
| 1 | -4.367 | -2.957 | 1.617 |
| 1 | -6.123 | 4.336 | 1.512 |
| 1 | -2.866 | -1.199 | 1.598 |
| 1 | -2.603 | 6.076 | 1.683 |
| 1 | -2.957 | 4.367 | 1.616 |
| 1 | 4.337 | 6.123 | 1.511 |
| 1 | -1.199 | 2.867 | 1.598 |
| 1 | 2.604 | -6.076 | 1.683 |
| 1 | 2.957 | -4.367 | 1.616 |
| 1 | -4.336 | -6.123 | 1.512 |
| 1 | 1.199 | -2.867 | 1.597 |
| 1 | 6.076 | 2.604 | 1.682 |
| 1 | 4.367 | 2.957 | 1.616 |
| 1 | 6.123 | -4.337 | 1.510 |
| 1 | 2.867 | 1.199 | 1.597 |
| 1 | 2.197 | 7.478 | 1.635 |
| 1 | 7.479 | -2.197 | 1.634 |
| 1 | -2.197 | -7.478 | 1.635 |
| 1 | -7.478 | 2.197 | 1.636 |

Cartesian coordinates of optimized structures in the gas phase by M05-2X/6-311G(d,p)// M05-2X/6-311G(d,p).

A-T

| 8 | 0.112 | -3.239 | 0.000 |
| --- | --- | --- | --- |
| 8 | 2.097 | 0.845 | 0.000 |
| 7 | 2.385 | -3.130 | 0.000 |
| 7 | 1.132 | -1.198 | 0.000 |
| 7 | -0.550 | 2.196 | 0.000 |
| 7 | -3.463 | 3.121 | 0.000 |
| 7 | -5.098 | 1.598 | 0.000 |
| 7 | -3.767 | -0.443 | 0.000 |
| 7 | -1.424 | 0.048 | 0.000 |
| 6 | 4.783 | -0.204 | 0.000 |
| 6 | 2.236 | -0.370 | 0.000 |
| 6 | 3.536 | -1.041 | 0.000 |
| 6 | 3.537 | -2.385 | 0.000 |
| 6 | 1.120 | -2.570 | 0.000 |
| 6 | -4.749 | 2.928 | 0.000 |
| 6 | -1.605 | 1.377 | 0.000 |
| 6 | -2.932 | 1.849 | 0.000 |
| 6 | -3.929 | 0.884 | 0.000 |
| 6 | -2.488 | -0.772 | 0.000 |
| 1 | 4.455 | -2.956 | 0.000 |
| 1 | 2.410 | -4.135 | 0.000 |
| 1 | 0.198 | -0.738 | 0.000 |
| 1 | 0.392 | 1.819 | 0.000 |
| 1 | -0.713 | 3.186 | 0.000 |
| 1 | -5.494 | 3.706 | 0.000 |
| 1 | -6.023 | 1.206 | 0.000 |
| 1 | -2.245 | -1.828 | 0.000 |
| 1 | 4.498 | 0.845 | 0.000 |
| 1 | 5.391 | -0.394 | -0.884 |
| 1 | 5.391 | -0.394 | 0.884 |

G-T

| 6 | -0.096 | 4.250 | -0.053 |
| --- | --- | --- | --- |
| 7 | 0.498 | 3.004 | -0.057 |
| 1 | 1.479 | 2.787 | -0.089 |
| 6 | -0.509 | 2.087 | -0.009 |
| 6 | -1.674 | 2.842 | 0.023 |
| 7 | -1.390 | 4.189 | -0.006 |
| 6 | -2.923 | 2.144 | 0.072 |
| 8 | -4.064 | 2.578 | 0.094 |
| 7 | -2.711 | 0.757 | 0.092 |
| 1 | -3.563 | 0.192 | 0.124 |
| 6 | -1.506 | 0.125 | 0.054 |
| 7 | -0.357 | 0.744 | -0.002 |
| 7 | -1.552 | -1.233 | 0.110 |
| 1 | 0.488 | 5.154 | -0.086 |
| 1 | -2.419 | -1.699 | -0.092 |
| 1 | -0.693 | -1.708 | -0.100 |
| 6 | -10.228 | 1.167 | -0.030 |
| 6 | -8.847 | 0.596 | -0.015 |
| 6 | -8.564 | -0.712 | -0.034 |
| 7 | -7.266 | -1.180 | -0.019 |
| 1 | -7.065 | -2.164 | -0.030 |
| 6 | -6.171 | -0.359 | 0.017 |
| 8 | -5.036 | -0.827 | 0.031 |
| 7 | -6.452 | 0.972 | 0.037 |
| 1 | -5.650 | 1.619 | 0.061 |
| 6 | -7.731 | 1.549 | 0.023 |
| 8 | -7.873 | 2.746 | 0.043 |
| 1 | -9.330 | -1.474 | -0.063 |
| 1 | -10.977 | 0.378 | -0.060 |
| 1 | -10.356 | 1.817 | -0.895 |
| 1 | -10.390 | 1.782 | 0.855 |

G-C

| 7 | -2.681 | 0.033 | 0.033 |
| --- | --- | --- | --- |
| 8 | 1.669 | 1.448 | 0.033 |
| 7 | -0.525 | 0.847 | 0.033 |
| 6 | -1.869 | 1.103 | 0.033 |
| 7 | -2.375 | 2.317 | 0.033 |
| 6 | -1.422 | 3.267 | 0.033 |
| 6 | -0.040 | 3.128 | 0.033 |
| 6 | 0.496 | 1.805 | 0.033 |
| 7 | -1.634 | 4.614 | 0.033 |
| 6 | -0.391 | 5.216 | 0.033 |
| 7 | 0.582 | 4.359 | 0.033 |
| 1 | -2.333 | -0.920 | 0.033 |
| 1 | -3.665 | 0.218 | 0.033 |
| 1 | -0.203 | -0.128 | 0.033 |
| 1 | -2.535 | 5.058 | 0.033 |
| 1 | -0.283 | 6.287 | 0.033 |
| 8 | -1.604 | -2.695 | 0.033 |
| 7 | 2.691 | -1.174 | 0.033 |
| 7 | 0.534 | -1.903 | 0.033 |
| 6 | 2.316 | -3.543 | 0.033 |
| 6 | -0.396 | -2.886 | 0.033 |
| 7 | 0.068 | -4.207 | 0.033 |
| 6 | 1.387 | -4.518 | 0.033 |
| 6 | 1.831 | -2.186 | 0.033 |
| 1 | 3.370 | -3.767 | 0.033 |
| 1 | -0.645 | -4.917 | 0.033 |
| 1 | 1.636 | -5.569 | 0.033 |
| 1 | 2.344 | -0.207 | 0.033 |
| 1 | 3.677 | -1.350 | 0.033 |

C-C

| 6 | 1.715 | 2.724 | 0.000 |
| --- | --- | --- | --- |
| 6 | 2.228 | 1.479 | 0.000 |
| 7 | 1.403 | 0.404 | 0.000 |
| 6 | -0.009 | 0.505 | 0.000 |
| 8 | -0.650 | -0.525 | 0.000 |
| 7 | -0.528 | 1.764 | 0.000 |
| 6 | 0.274 | 2.808 | 0.000 |
| 7 | -0.279 | 4.029 | 0.000 |
| 1 | 2.322 | 3.614 | 0.000 |
| 1 | 3.289 | 1.277 | 0.000 |
| 1 | -1.280 | 4.082 | 0.000 |
| 1 | 0.290 | 4.863 | 0.000 |
| 6 | 0.484 | 9.844 | 0.000 |
| 6 | 1.810 | 9.598 | 0.000 |
| 7 | 2.264 | 8.325 | 0.000 |
| 6 | 1.411 | 7.210 | 0.000 |
| 8 | 1.911 | 6.098 | 0.000 |
| 7 | 0.074 | 7.451 | 0.000 |
| 6 | -0.372 | 8.688 | 0.000 |
| 7 | -1.705 | 8.864 | 0.000 |
| 1 | 0.092 | 10.847 | 0.000 |
| 1 | 2.555 | 10.381 | 0.000 |
| 1 | -2.289 | 8.048 | 0.000 |
| 1 | -2.118 | 9.776 | 0.000 |
| 1 | 1.759 | -0.536 | 0.000 |
| 1 | 3.248 | 8.115 | 0.000 |

A-T/A-T

| 8 | -2.731 | -0.985 | -1.871 |
| --- | --- | --- | --- |
| 8 | -1.025 | 3.040 | -0.620 |
| 7 | -4.160 | 0.691 | -1.309 |
| 7 | -1.886 | 1.057 | -1.297 |
| 7 | 1.746 | 2.089 | -1.233 |
| 7 | 4.373 | 0.517 | -1.382 |
| 7 | 4.311 | -1.678 | -1.801 |
| 7 | 1.910 | -2.001 | -2.003 |
| 7 | 0.724 | 0.054 | -1.685 |
| 6 | -3.553 | 4.215 | -0.093 |
| 6 | -2.012 | 2.346 | -0.825 |
| 6 | -3.375 | 2.821 | -0.601 |
| 6 | -4.380 | 1.970 | -0.858 |
| 6 | -2.901 | 0.166 | -1.521 |
| 6 | 5.074 | -0.570 | -1.533 |
| 6 | 1.837 | 0.777 | -1.483 |
| 6 | 3.069 | 0.098 | -1.550 |
| 6 | 3.006 | -1.265 | -1.806 |
| 6 | 0.816 | -1.266 | -1.923 |
| 1 | -5.417 | 2.243 | -0.722 |
| 1 | -4.919 | 0.047 | -1.448 |
| 1 | -0.917 | 0.697 | -1.453 |
| 1 | 0.834 | 2.488 | -1.039 |
| 1 | 2.569 | 2.558 | -0.897 |
| 1 | 6.149 | -0.628 | -1.474 |
| 1 | 4.621 | -2.628 | -1.902 |
| 1 | -0.130 | -1.775 | -2.055 |
| 1 | -3.078 | 4.927 | -0.767 |
| 1 | -3.075 | 4.324 | 0.880 |
| 1 | -4.610 | 4.463 | 0.001 |
| 8 | -0.689 | -3.579 | 0.045 |
| 8 | -2.430 | 0.238 | 1.790 |
| 7 | -2.947 | -3.386 | 0.231 |
| 7 | -1.568 | -1.698 | 0.989 |
| 7 | 0.307 | 1.407 | 1.703 |
| 7 | 3.251 | 2.232 | 1.789 |
| 7 | 4.829 | 0.651 | 1.781 |
| 7 | 3.432 | -1.333 | 1.548 |
| 7 | 1.105 | -0.760 | 1.519 |
| 6 | -5.117 | -0.686 | 1.734 |
| 6 | -2.620 | -0.896 | 1.366 |
| 6 | -3.953 | -1.475 | 1.222 |
| 6 | -4.046 | -2.683 | 0.641 |
| 6 | -1.651 | -2.933 | 0.390 |
| 6 | 4.530 | 1.989 | 1.819 |
| 6 | 1.333 | 0.558 | 1.648 |
| 6 | 2.676 | 0.981 | 1.711 |
| 6 | 3.638 | -0.018 | 1.681 |
| 6 | 2.141 | -1.610 | 1.459 |
| 1 | -4.998 | -3.169 | 0.475 |
| 1 | -3.035 | -4.262 | -0.253 |
| 1 | -0.604 | -1.345 | 1.147 |
| 1 | -0.649 | 1.065 | 1.702 |
| 1 | 0.487 | 2.394 | 1.702 |
| 1 | 5.302 | 2.738 | 1.871 |
| 1 | 5.740 | 0.232 | 1.725 |
| 1 | 1.869 | -2.647 | 1.309 |
| 1 | -5.080 | 0.333 | 1.351 |
| 1 | -5.081 | -0.613 | 2.821 |
| 1 | -6.060 | -1.151 | 1.448 |

G-C/A-T

| 8 | -3.187 | 1.348 | 1.835 |
| --- | --- | --- | --- |
| 8 | -1.929 | -2.701 | 0.186 |
| 7 | -4.786 | -0.191 | 1.353 |
| 7 | -2.559 | -0.696 | 1.033 |
| 7 | 0.982 | -2.166 | 0.935 |
| 7 | 3.696 | -1.047 | 1.809 |
| 7 | 3.834 | 1.071 | 2.507 |
| 7 | 1.506 | 1.740 | 2.286 |
| 7 | 0.155 | -0.063 | 1.474 |
| 6 | -4.579 | -3.661 | -0.132 |
| 6 | -2.829 | -1.952 | 0.523 |
| 6 | -4.246 | -2.308 | 0.413 |
| 6 | -5.149 | -1.417 | 0.849 |
| 6 | -3.473 | 0.238 | 1.438 |
| 6 | 4.484 | -0.110 | 2.247 |
| 6 | 1.185 | -0.922 | 1.377 |
| 6 | 2.454 | -0.450 | 1.769 |
| 6 | 2.516 | 0.870 | 2.197 |
| 6 | 0.365 | 1.190 | 1.909 |
| 1 | -6.212 | -1.615 | 0.829 |
| 1 | -5.473 | 0.466 | 1.678 |
| 1 | -1.548 | -0.441 | 1.137 |
| 1 | 0.089 | -2.388 | 0.508 |
| 1 | 1.790 | -2.695 | 0.651 |
| 1 | 5.544 | -0.217 | 2.411 |
| 1 | 4.234 | 1.935 | 2.828 |
| 1 | -0.518 | 1.815 | 1.947 |
| 1 | -4.131 | -4.437 | 0.488 |
| 1 | -4.167 | -3.783 | -1.133 |
| 1 | -5.657 | -3.812 | -0.169 |
| 7 | 2.200 | 2.782 | -0.918 |
| 8 | 0.598 | -1.352 | -2.061 |
| 7 | 1.494 | 0.664 | -1.492 |
| 6 | 2.520 | 1.489 | -1.124 |
| 7 | 3.767 | 1.085 | -0.997 |
| 6 | 3.902 | -0.233 | -1.234 |
| 6 | 2.933 | -1.167 | -1.572 |
| 6 | 1.594 | -0.707 | -1.748 |
| 7 | 5.056 | -0.953 | -1.138 |
| 6 | 4.727 | -2.271 | -1.372 |
| 7 | 3.468 | -2.436 | -1.641 |
| 1 | 1.232 | 3.044 | -0.747 |
| 1 | 2.917 | 3.326 | -0.475 |
| 1 | 0.534 | 1.033 | -1.520 |
| 1 | 5.948 | -0.583 | -0.866 |
| 1 | 5.465 | -3.054 | -1.329 |
| 8 | -0.633 | 3.435 | -0.493 |
| 7 | -2.072 | -0.490 | -2.299 |
| 7 | -1.323 | 1.478 | -1.431 |
| 6 | -3.710 | 1.139 | -1.646 |
| 6 | -1.528 | 2.695 | -0.877 |
| 7 | -2.852 | 3.125 | -0.743 |
| 6 | -3.904 | 2.357 | -1.100 |
| 6 | -2.343 | 0.709 | -1.792 |
| 1 | -4.533 | 0.511 | -1.944 |
| 1 | -2.975 | 3.999 | -0.260 |
| 1 | -4.886 | 2.772 | -0.922 |
| 1 | -1.107 | -0.838 | -2.267 |
| 1 | -2.818 | -1.130 | -2.496 |

G-C/T-A

| 8 | -2.220 | -0.745 | -1.886 |
| --- | --- | --- | --- |
| 8 | 0.157 | 3.080 | -1.337 |
| 7 | -3.318 | 1.114 | -1.148 |
| 7 | -1.071 | 1.205 | -1.670 |
| 7 | 2.673 | 1.545 | -1.648 |
| 7 | 4.864 | -0.385 | -0.703 |
| 7 | 4.427 | -2.573 | -0.614 |
| 7 | 2.110 | -2.568 | -1.351 |
| 7 | 1.344 | -0.346 | -1.805 |
| 6 | -2.093 | 4.628 | -0.492 |
| 6 | -0.938 | 2.524 | -1.297 |
| 6 | -2.158 | 3.176 | -0.849 |
| 6 | -3.268 | 2.421 | -0.753 |
| 6 | -2.208 | 0.433 | -1.594 |
| 6 | 5.325 | -1.565 | -0.390 |
| 6 | 2.533 | 0.216 | -1.544 |
| 6 | 3.582 | -0.635 | -1.154 |
| 6 | 3.280 | -1.988 | -1.082 |
| 6 | 1.196 | -1.673 | -1.692 |
| 1 | -4.195 | 2.813 | -0.358 |
| 1 | -4.097 | 0.529 | -0.860 |
| 1 | -0.208 | 0.685 | -1.893 |
| 1 | 1.847 | 2.137 | -1.692 |
| 1 | 3.559 | 1.944 | -1.402 |
| 1 | 6.309 | -1.761 | 0.002 |
| 1 | 4.538 | -3.547 | -0.394 |
| 1 | 0.196 | -2.038 | -1.897 |
| 1 | -1.919 | 5.237 | -1.380 |
| 1 | -1.258 | 4.823 | 0.182 |
| 1 | -3.018 | 4.955 | -0.020 |
| 7 | 0.306 | -2.935 | 1.340 |
| 8 | -2.529 | 0.642 | 1.736 |
| 7 | -1.205 | -1.201 | 1.536 |
| 6 | -0.944 | -2.483 | 1.129 |
| 7 | -1.845 | -3.266 | 0.577 |
| 6 | -3.030 | -2.655 | 0.428 |
| 6 | -3.412 | -1.379 | 0.815 |
| 6 | -2.425 | -0.538 | 1.403 |
| 7 | -4.118 | -3.163 | -0.220 |
| 6 | -5.071 | -2.174 | -0.245 |
| 7 | -4.687 | -1.091 | 0.364 |
| 1 | 1.059 | -2.279 | 1.533 |
| 1 | 0.548 | -3.752 | 0.805 |
| 1 | -0.420 | -0.609 | 1.833 |
| 1 | -4.153 | -4.060 | -0.670 |
| 1 | -6.024 | -2.316 | -0.726 |
| 8 | 2.496 | -1.039 | 1.702 |
| 7 | -0.389 | 2.478 | 1.989 |
| 7 | 1.031 | 0.698 | 1.879 |
| 6 | 1.941 | 2.934 | 1.666 |
| 6 | 2.266 | 0.167 | 1.719 |
| 7 | 3.333 | 1.050 | 1.552 |
| 6 | 3.164 | 2.393 | 1.510 |
| 6 | 0.850 | 2.014 | 1.844 |
| 1 | 1.778 | 3.998 | 1.619 |
| 1 | 4.212 | 0.626 | 1.293 |
| 1 | 4.053 | 2.983 | 1.342 |
| 1 | -1.178 | 1.826 | 2.004 |
| 1 | -0.566 | 3.459 | 1.883 |

G-C/G-C

| 7 | -0.122 | -2.466 | 1.446 |
| --- | --- | --- | --- |
| 8 | -2.604 | 1.365 | 1.679 |
| 7 | -1.473 | -0.608 | 1.596 |
| 6 | -1.338 | -1.928 | 1.262 |
| 7 | -2.326 | -2.661 | 0.788 |
| 6 | -3.464 | -1.957 | 0.654 |
| 6 | -3.715 | -0.627 | 0.954 |
| 6 | -2.631 | 0.163 | 1.443 |
| 7 | -4.620 | -2.410 | 0.087 |
| 6 | -5.484 | -1.342 | 0.025 |
| 7 | -4.976 | -0.257 | 0.530 |
| 1 | 0.687 | -1.890 | 1.666 |
| 1 | 0.054 | -3.336 | 0.976 |
| 1 | -0.621 | -0.080 | 1.825 |
| 1 | -4.767 | -3.339 | -0.266 |
| 1 | -6.470 | -1.434 | -0.399 |
| 8 | 2.313 | -0.909 | 2.000 |
| 7 | -0.164 | 2.871 | 1.356 |
| 7 | 1.035 | 0.965 | 1.745 |
| 6 | 2.217 | 3.026 | 1.244 |
| 6 | 2.210 | 0.305 | 1.840 |
| 7 | 3.382 | 1.056 | 1.742 |
| 6 | 3.379 | 2.379 | 1.427 |
| 6 | 1.010 | 2.262 | 1.441 |
| 1 | 2.177 | 4.068 | 0.976 |
| 1 | 4.237 | 0.532 | 1.822 |
| 1 | 4.344 | 2.856 | 1.336 |
| 1 | -1.023 | 2.332 | 1.476 |
| 1 | -0.242 | 3.730 | 0.837 |
| 7 | -0.681 | -2.051 | -1.959 |
| 8 | 1.121 | 2.143 | -1.562 |
| 7 | 0.123 | 0.110 | -1.779 |
| 6 | -0.955 | -0.732 | -1.804 |
| 7 | -2.195 | -0.321 | -1.705 |
| 6 | -2.286 | 1.011 | -1.517 |
| 6 | -1.267 | 1.955 | -1.482 |
| 6 | 0.073 | 1.494 | -1.614 |
| 7 | -3.427 | 1.709 | -1.254 |
| 6 | -3.045 | 3.010 | -1.028 |
| 7 | -1.768 | 3.206 | -1.176 |
| 1 | 0.237 | -2.379 | -1.673 |
| 1 | -1.448 | -2.642 | -1.680 |
| 1 | 1.063 | -0.296 | -1.746 |
| 1 | -4.296 | 1.281 | -0.959 |
| 1 | -3.763 | 3.764 | -0.753 |
| 8 | 1.906 | -2.962 | -0.781 |
| 7 | 3.761 | 1.116 | -1.607 |
| 7 | 2.815 | -0.919 | -1.225 |
| 6 | 5.142 | -0.601 | -0.645 |
| 6 | 2.851 | -2.187 | -0.750 |
| 7 | 4.062 | -2.634 | -0.212 |
| 6 | 5.168 | -1.862 | -0.163 |
| 6 | 3.882 | -0.137 | -1.167 |
| 1 | 6.019 | 0.027 | -0.635 |
| 1 | 4.047 | -3.562 | 0.176 |
| 1 | 6.052 | -2.309 | 0.269 |
| 1 | 2.820 | 1.502 | -1.746 |
| 1 | 4.537 | 1.747 | -1.530 |

C^+^•G-C

| 7 | -2.695 | -0.015 | 0.000 |
| --- | --- | --- | --- |
| 8 | 1.693 | 1.331 | 0.000 |
| 7 | -0.512 | 0.735 | 0.000 |
| 6 | -1.862 | 1.021 | 0.000 |
| 7 | -2.341 | 2.254 | 0.000 |
| 6 | -1.384 | 3.180 | 0.000 |
| 6 | -0.010 | 2.998 | 0.000 |
| 6 | 0.496 | 1.676 | 0.000 |
| 7 | -1.557 | 4.540 | 0.000 |
| 6 | -0.318 | 5.117 | 0.000 |
| 7 | 0.631 | 4.221 | 0.000 |
| 1 | -2.381 | -0.986 | 0.000 |
| 1 | -3.676 | 0.201 | 0.000 |
| 1 | -0.211 | -0.255 | 0.000 |
| 1 | -2.445 | 5.012 | 0.000 |
| 1 | -0.155 | 6.181 | 0.000 |
| 8 | -1.783 | -2.687 | 0.000 |
| 7 | 2.611 | -1.488 | 0.000 |
| 7 | 0.406 | -2.054 | 0.000 |
| 6 | 2.060 | -3.822 | 0.000 |
| 6 | -0.594 | -2.972 | 0.000 |
| 7 | -0.229 | -4.318 | 0.000 |
| 6 | 1.060 | -4.727 | 0.000 |
| 6 | 1.675 | -2.439 | 0.000 |
| 1 | 3.095 | -4.123 | 0.000 |
| 1 | -0.993 | -4.974 | 0.000 |
| 1 | 1.230 | -5.794 | 0.000 |
| 1 | 2.329 | -0.512 | 0.000 |
| 1 | 3.581 | -1.737 | 0.000 |
| 6 | 5.547 | 4.734 | 0.000 |
| 6 | 5.643 | 6.081 | 0.000 |
| 7 | 4.542 | 6.871 | 0.000 |
| 6 | 3.246 | 6.370 | 0.000 |
| 8 | 2.273 | 7.075 | 0.000 |
| 7 | 3.177 | 4.988 | 0.000 |
| 1 | 2.152 | 4.607 | 0.000 |
| 6 | 4.236 | 4.164 | 0.000 |
| 7 | 4.035 | 2.863 | 0.000 |
| 1 | 6.424 | 4.108 | 0.000 |
| 1 | 6.595 | 6.591 | 0.000 |
| 1 | 3.099 | 2.426 | 0.000 |
| 1 | 4.834 | 2.254 | 0.000 |
| 1 | 4.616 | 7.877 | 0.000 |

T•A-T

| 8 | 0.068 | -3.324 | 0.006 |
| --- | --- | --- | --- |
| 8 | 2.092 | 0.740 | -0.004 |
| 7 | 2.341 | -3.236 | 0.004 |
| 7 | 1.107 | -1.293 | 0.001 |
| 7 | -0.584 | 2.137 | -0.004 |
| 7 | -3.551 | 3.004 | -0.003 |
| 7 | -5.141 | 1.457 | 0.001 |
| 7 | -3.768 | -0.554 | 0.004 |
| 7 | -1.434 | -0.017 | 0.001 |
| 6 | 4.769 | -0.333 | -0.003 |
| 6 | 2.220 | -0.474 | -0.001 |
| 6 | 3.514 | -1.159 | -0.001 |
| 6 | 3.501 | -2.503 | 0.002 |
| 6 | 1.082 | -2.663 | 0.004 |
| 6 | -4.837 | 2.790 | -0.002 |
| 6 | -1.629 | 1.316 | -0.002 |
| 6 | -2.974 | 1.752 | -0.001 |
| 6 | -3.952 | 0.769 | 0.002 |
| 6 | -2.480 | -0.855 | 0.003 |
| 1 | 4.413 | -3.084 | 0.002 |
| 1 | 2.356 | -4.241 | 0.006 |
| 1 | 0.180 | -0.819 | 0.001 |
| 1 | 0.350 | 1.741 | -0.004 |
| 1 | -0.700 | 3.142 | -0.005 |
| 1 | -5.581 | 3.571 | -0.003 |
| 1 | -6.055 | 1.039 | 0.003 |
| 1 | -2.218 | -1.907 | 0.005 |
| 1 | 4.492 | 0.718 | -0.005 |
| 1 | 5.374 | -0.530 | -0.888 |
| 1 | 5.375 | -0.527 | 0.881 |
| 6 | 0.310 | 7.896 | 0.005 |
| 6 | -1.126 | 7.480 | 0.002 |
| 6 | -2.167 | 8.328 | 0.004 |
| 7 | -3.473 | 7.897 | 0.001 |
| 1 | -4.239 | 8.548 | 0.003 |
| 6 | -3.830 | 6.563 | -0.004 |
| 8 | -4.986 | 6.194 | -0.006 |
| 7 | -2.758 | 5.711 | -0.007 |
| 1 | -2.988 | 4.700 | -0.010 |
| 6 | -1.419 | 6.047 | -0.003 |
| 8 | -0.559 | 5.182 | -0.005 |
| 1 | -2.034 | 9.401 | 0.008 |
| 1 | 0.401 | 8.981 | 0.009 |
| 1 | 0.821 | 7.499 | -0.872 |
| 1 | 0.819 | 7.492 | 0.880 |

rT•A-T

| 8 | 0.120 | -3.405 | 0.000 |
| --- | --- | --- | --- |
| 8 | 2.198 | 0.632 | 0.000 |
| 7 | 2.394 | -3.347 | 0.000 |
| 7 | 1.186 | -1.388 | 0.000 |
| 7 | -0.459 | 2.059 | 0.000 |
| 7 | -3.414 | 2.967 | 0.000 |
| 7 | -5.025 | 1.443 | 0.000 |
| 7 | -3.680 | -0.587 | 0.000 |
| 7 | -1.339 | -0.083 | 0.000 |
| 6 | 4.860 | -0.478 | 0.000 |
| 6 | 2.310 | -0.584 | 0.000 |
| 6 | 3.595 | -1.287 | 0.000 |
| 6 | 3.564 | -2.630 | 0.000 |
| 6 | 1.143 | -2.758 | 0.000 |
| 6 | -4.703 | 2.772 | 0.000 |
| 6 | -1.516 | 1.253 | 0.000 |
| 6 | -2.854 | 1.708 | 0.000 |
| 6 | -3.845 | 0.738 | 0.000 |
| 6 | -2.396 | -0.906 | 0.000 |
| 1 | 4.468 | -3.223 | 0.000 |
| 1 | 2.395 | -4.352 | 0.000 |
| 1 | 0.265 | -0.902 | 0.000 |
| 1 | 0.470 | 1.651 | 0.000 |
| 1 | -0.560 | 3.066 | 0.000 |
| 1 | -5.435 | 3.564 | 0.000 |
| 1 | -5.944 | 1.038 | 0.000 |
| 1 | -2.148 | -1.961 | 0.000 |
| 1 | 4.597 | 0.577 | 0.000 |
| 1 | 5.463 | -0.681 | -0.884 |
| 1 | 5.463 | -0.681 | 0.884 |
| 6 | -4.388 | 8.992 | 0.000 |
| 6 | -3.288 | 7.979 | 0.000 |
| 6 | -1.980 | 8.276 | 0.000 |
| 7 | -1.000 | 7.309 | 0.000 |
| 1 | -0.025 | 7.550 | 0.000 |
| 6 | -1.277 | 5.960 | 0.000 |
| 8 | -0.400 | 5.116 | 0.000 |
| 7 | -2.611 | 5.668 | 0.000 |
| 1 | -2.853 | 4.660 | 0.000 |
| 6 | -3.668 | 6.564 | 0.000 |
| 8 | -4.818 | 6.168 | 0.000 |
| 1 | -1.621 | 9.296 | 0.000 |
| 1 | -3.987 | 10.004 | 0.000 |
| 1 | -5.023 | 8.859 | 0.876 |
| 1 | -5.023 | 8.859 | -0.876 |

rA•A-T

| 8 | 0.149 | -3.273 | 0.000 |
| --- | --- | --- | --- |
| 8 | 1.998 | 0.876 | 0.000 |
| 7 | 2.416 | -3.088 | 0.000 |
| 7 | 1.099 | -1.198 | 0.000 |
| 7 | -0.749 | 2.071 | 0.000 |
| 7 | -3.739 | 2.861 | 0.000 |
| 7 | -5.277 | 1.248 | 0.000 |
| 7 | -3.841 | -0.713 | 0.000 |
| 7 | -1.528 | -0.103 | 0.000 |
| 6 | 4.717 | -0.087 | 0.000 |
| 6 | 2.175 | -0.335 | 0.000 |
| 6 | 3.496 | -0.963 | 0.000 |
| 6 | 3.542 | -2.306 | 0.000 |
| 6 | 1.134 | -2.570 | 0.000 |
| 6 | -5.014 | 2.591 | 0.000 |
| 6 | -1.768 | 1.222 | 0.000 |
| 6 | -3.124 | 1.624 | 0.000 |
| 6 | -4.066 | 0.604 | 0.000 |
| 6 | -2.545 | -0.975 | 0.000 |
| 1 | 4.479 | -2.847 | 0.000 |
| 1 | 2.474 | -4.092 | 0.000 |
| 1 | 0.144 | -0.778 | 0.000 |
| 1 | 0.195 | 1.700 | 0.000 |
| 1 | -0.891 | 3.076 | 0.000 |
| 1 | -5.805 | 3.323 | 0.000 |
| 1 | -6.175 | 0.797 | 0.000 |
| 1 | -2.249 | -2.017 | 0.000 |
| 7 | -2.484 | 8.062 | 0.000 |
| 6 | -2.276 | 6.739 | 0.000 |
| 6 | -0.944 | 6.277 | 0.000 |
| 6 | 0.042 | 7.254 | 0.000 |
| 7 | -0.137 | 8.575 | 0.000 |
| 6 | -1.428 | 8.881 | 0.000 |
| 7 | 1.224 | 6.557 | 0.000 |
| 6 | 0.902 | 5.226 | 0.000 |
| 7 | -0.384 | 5.016 | 0.000 |
| 7 | -3.331 | 5.916 | 0.000 |
| 1 | -1.665 | 9.938 | 0.000 |
| 1 | 1.655 | 4.453 | 0.000 |
| 1 | -4.238 | 6.345 | 0.000 |
| 1 | -3.242 | 4.906 | 0.000 |
| 1 | 2.143 | 6.964 | 0.000 |
| 1 | 4.399 | 0.952 | 0.000 |
| 1 | 5.330 | -0.258 | -0.884 |
| 1 | 5.330 | -0.258 | 0.884 |

rG•G-C

| 7 | -2.675 | 0.055 | 0.000 |
| --- | --- | --- | --- |
| 8 | 1.763 | 1.216 | 0.000 |
| 7 | -0.468 | 0.724 | 0.000 |
| 6 | -1.798 | 1.069 | 0.000 |
| 7 | -2.229 | 2.313 | 0.000 |
| 6 | -1.225 | 3.204 | 0.000 |
| 6 | 0.139 | 2.963 | 0.000 |
| 6 | 0.594 | 1.619 | 0.000 |
| 7 | -1.333 | 4.567 | 0.000 |
| 6 | -0.057 | 5.083 | 0.000 |
| 7 | 0.841 | 4.144 | 0.000 |
| 1 | -2.390 | -0.920 | 0.000 |
| 1 | -3.645 | 0.304 | 0.000 |
| 1 | -0.210 | -0.272 | 0.000 |
| 1 | -2.196 | 5.082 | 0.000 |
| 1 | 0.171 | 6.138 | 0.000 |
| 8 | -1.786 | -2.715 | 0.000 |
| 7 | 2.609 | -1.516 | 0.000 |
| 7 | 0.404 | -2.084 | 0.000 |
| 6 | 2.060 | -3.851 | 0.000 |
| 6 | -0.596 | -2.997 | 0.000 |
| 7 | -0.231 | -4.347 | 0.000 |
| 6 | 1.061 | -4.755 | 0.000 |
| 6 | 1.676 | -2.463 | 0.000 |
| 1 | 3.095 | -4.152 | 0.000 |
| 1 | -0.994 | -5.002 | 0.000 |
| 1 | 1.231 | -5.821 | 0.000 |
| 1 | 2.332 | -0.531 | 0.000 |
| 1 | 3.580 | -1.764 | 0.000 |
| 6 | 6.650 | 8.156 | 0.000 |
| 7 | 7.037 | 6.832 | 0.000 |
| 1 | 7.970 | 6.461 | 0.000 |
| 6 | 5.895 | 6.085 | 0.000 |
| 6 | 4.864 | 7.015 | 0.000 |
| 7 | 5.361 | 8.301 | 0.000 |
| 6 | 3.514 | 6.525 | 0.000 |
| 8 | 2.458 | 7.130 | 0.000 |
| 7 | 3.511 | 5.117 | 0.000 |
| 1 | 2.574 | 4.695 | 0.000 |
| 6 | 4.598 | 4.301 | 0.000 |
| 7 | 5.838 | 4.735 | 0.000 |
| 7 | 4.352 | 2.973 | 0.000 |
| 1 | 7.371 | 8.956 | 0.000 |
| 1 | 3.422 | 2.577 | 0.000 |
| 1 | 5.152 | 2.372 | 0.000 |

C^+^•G-C/C^+^•G-C

| 7 | -1.358 | 1.770 | -2.828 |
| --- | --- | --- | --- |
| 8 | 0.870 | -1.624 | -0.689 |
| 7 | -0.180 | 0.111 | -1.745 |
| 6 | -0.198 | 1.364 | -2.326 |
| 7 | 0.868 | 2.149 | -2.393 |
| 6 | 1.956 | 1.588 | -1.873 |
| 6 | 2.080 | 0.337 | -1.283 |
| 6 | 0.930 | -0.485 | -1.192 |
| 7 | 3.217 | 2.120 | -1.825 |
| 6 | 4.037 | 1.196 | -1.242 |
| 7 | 3.388 | 0.114 | -0.900 |
| 1 | -2.185 | 1.167 | -2.883 |
| 1 | -1.364 | 2.670 | -3.276 |
| 1 | -1.068 | -0.426 | -1.685 |
| 1 | 3.480 | 3.037 | -2.149 |
| 1 | 5.093 | 1.354 | -1.101 |
| 8 | -3.601 | 0.114 | -2.932 |
| 7 | -1.803 | -2.874 | -0.007 |
| 7 | -2.658 | -1.386 | -1.503 |
| 6 | -4.028 | -3.258 | -0.822 |
| 6 | -3.660 | -0.936 | -2.303 |
| 7 | -4.796 | -1.724 | -2.418 |
| 6 | -4.989 | -2.831 | -1.670 |
| 6 | -2.820 | -2.488 | -0.785 |
| 1 | -4.153 | -4.150 | -0.230 |
| 1 | -5.534 | -1.340 | -2.987 |
| 1 | -5.932 | -3.340 | -1.797 |
| 1 | -0.919 | -2.383 | -0.072 |
| 1 | -1.864 | -3.728 | 0.514 |
| 6 | 5.119 | -4.478 | -0.794 |
| 6 | 6.420 | -4.245 | -0.516 |
| 7 | 6.860 | -3.013 | -0.153 |
| 6 | 6.032 | -1.909 | -0.064 |
| 8 | 6.428 | -0.808 | 0.230 |
| 7 | 4.703 | -2.174 | -0.342 |
| 1 | 4.106 | -1.285 | -0.447 |
| 6 | 4.215 | -3.377 | -0.706 |
| 7 | 2.929 | -3.492 | -0.957 |
| 1 | 4.774 | -5.458 | -1.080 |
| 1 | 7.169 | -5.022 | -0.566 |
| 1 | 2.250 | -2.720 | -0.847 |
| 1 | 2.574 | -4.381 | -1.268 |
| 1 | 7.838 | -2.843 | 0.033 |
| 7 | -4.622 | 2.325 | -0.614 |
| 8 | -0.968 | 0.449 | 1.418 |
| 7 | -2.733 | 1.445 | 0.372 |
| 6 | -3.346 | 2.495 | -0.276 |
| 7 | -2.732 | 3.634 | -0.549 |
| 6 | -1.465 | 3.648 | -0.141 |
| 6 | -0.756 | 2.645 | 0.507 |
| 6 | -1.430 | 1.442 | 0.819 |
| 7 | -0.566 | 4.670 | -0.286 |
| 6 | 0.619 | 4.262 | 0.257 |
| 7 | 0.540 | 3.055 | 0.747 |
| 1 | -5.090 | 1.426 | -0.534 |
| 1 | -5.055 | 3.070 | -1.129 |
| 1 | -3.288 | 0.609 | 0.616 |
| 1 | -0.760 | 5.561 | -0.713 |
| 1 | 1.506 | 4.874 | 0.275 |
| 8 | -5.914 | -0.232 | -0.100 |
| 7 | -2.806 | -1.237 | 3.066 |
| 7 | -4.345 | -0.722 | 1.472 |
| 6 | -5.052 | -2.034 | 3.378 |
| 6 | -5.597 | -0.769 | 0.956 |
| 7 | -6.563 | -1.465 | 1.677 |
| 6 | -6.297 | -2.070 | 2.860 |
| 6 | -4.064 | -1.317 | 2.624 |
| 1 | -4.819 | -2.507 | 4.318 |
| 1 | -7.495 | -1.448 | 1.294 |
| 1 | -7.124 | -2.566 | 3.346 |
| 1 | -2.136 | -0.692 | 2.532 |
| 1 | -2.573 | -1.565 | 3.985 |
| 6 | 3.993 | 0.016 | 2.596 |
| 6 | 5.100 | 0.775 | 2.440 |
| 7 | 5.034 | 2.019 | 1.910 |
| 6 | 3.849 | 2.605 | 1.488 |
| 8 | 3.806 | 3.693 | 0.973 |
| 7 | 2.729 | 1.825 | 1.695 |
| 1 | 1.810 | 2.281 | 1.349 |
| 6 | 2.747 | 0.570 | 2.176 |
| 7 | 1.619 | -0.104 | 2.244 |
| 1 | 4.044 | -0.971 | 3.021 |
| 1 | 6.085 | 0.428 | 2.715 |
| 1 | 0.709 | 0.253 | 1.909 |
| 1 | 1.646 | -1.051 | 2.577 |
| 1 | 5.863 | 2.583 | 1.797 |

C^+^•G-C/T•A-T

| 8 | -3.921 | -1.657 | 2.325 |
| --- | --- | --- | --- |
| 8 | -2.209 | 2.349 | 1.054 |
| 7 | -5.280 | 0.172 | 2.369 |
| 7 | -3.082 | 0.371 | 1.719 |
| 7 | 0.534 | 1.161 | 0.988 |
| 7 | 3.179 | -0.419 | 1.349 |
| 7 | 3.095 | -2.543 | 1.991 |
| 7 | 0.686 | -2.835 | 2.166 |
| 7 | -0.479 | -0.811 | 1.641 |
| 6 | -4.647 | 3.784 | 1.457 |
| 6 | -3.180 | 1.719 | 1.456 |
| 6 | -4.491 | 2.314 | 1.689 |
| 6 | -5.472 | 1.506 | 2.128 |
| 6 | -4.080 | -0.468 | 2.151 |
| 6 | 3.880 | -1.477 | 1.661 |
| 6 | 0.634 | -0.113 | 1.363 |
| 6 | 1.861 | -0.804 | 1.496 |
| 6 | 1.788 | -2.130 | 1.902 |
| 6 | -0.401 | -2.096 | 2.015 |
| 1 | -6.471 | 1.871 | 2.324 |
| 1 | -6.047 | -0.424 | 2.631 |
| 1 | -2.149 | -0.064 | 1.619 |
| 1 | -0.384 | 1.600 | 0.960 |
| 1 | 1.355 | 1.707 | 0.772 |
| 1 | 4.958 | -1.508 | 1.678 |
| 1 | 3.400 | -3.461 | 2.266 |
| 1 | -1.356 | -2.567 | 2.219 |
| 1 | -4.013 | 4.347 | 2.142 |
| 1 | -5.681 | 4.093 | 1.605 |
| 1 | -4.334 | 4.047 | 0.447 |
| 6 | 4.042 | 5.413 | -0.641 |
| 6 | 4.725 | 4.209 | -0.072 |
| 6 | 6.014 | 4.177 | 0.317 |
| 7 | 6.610 | 3.046 | 0.817 |
| 1 | 7.586 | 3.040 | 1.065 |
| 6 | 5.942 | 1.853 | 0.983 |
| 8 | 6.479 | 0.829 | 1.347 |
| 7 | 4.606 | 1.931 | 0.671 |
| 1 | 4.053 | 1.075 | 0.863 |
| 6 | 3.956 | 2.984 | 0.072 |
| 8 | 2.795 | 2.846 | -0.315 |
| 1 | 6.655 | 5.044 | 0.244 |
| 1 | 4.725 | 6.259 | -0.690 |
| 1 | 3.184 | 5.688 | -0.028 |
| 1 | 3.670 | 5.203 | -1.643 |
| 7 | -4.705 | -2.527 | -0.618 |
| 8 | -0.738 | -0.427 | -1.564 |
| 7 | -2.668 | -1.535 | -1.056 |
| 6 | -3.379 | -2.658 | -0.695 |
| 7 | -2.815 | -3.829 | -0.464 |
| 6 | -1.494 | -3.810 | -0.643 |
| 6 | -0.695 | -2.748 | -1.036 |
| 6 | -1.303 | -1.488 | -1.250 |
| 7 | -0.623 | -4.851 | -0.463 |
| 6 | 0.634 | -4.394 | -0.737 |
| 7 | 0.627 | -3.138 | -1.089 |
| 1 | -5.155 | -1.615 | -0.631 |
| 1 | -5.191 | -3.305 | -0.210 |
| 1 | -3.171 | -0.643 | -1.186 |
| 1 | -0.877 | -5.773 | -0.149 |
| 1 | 1.519 | -5.005 | -0.671 |
| 8 | -5.937 | 0.093 | -0.685 |
| 7 | -2.140 | 2.037 | -2.280 |
| 7 | -4.024 | 1.034 | -1.488 |
| 6 | -4.187 | 3.280 | -2.379 |
| 6 | -5.348 | 1.033 | -1.210 |
| 7 | -6.077 | 2.174 | -1.542 |
| 6 | -5.509 | 3.262 | -2.118 |
| 6 | -3.445 | 2.098 | -2.034 |
| 1 | -3.712 | 4.134 | -2.833 |
| 1 | -7.064 | 2.133 | -1.350 |
| 1 | -6.166 | 4.089 | -2.343 |
| 1 | -1.616 | 1.216 | -1.985 |
| 1 | -1.654 | 2.846 | -2.616 |
| 6 | 4.387 | 0.024 | -1.793 |
| 6 | 5.427 | -0.824 | -1.673 |
| 7 | 5.233 | -2.150 | -1.448 |
| 6 | 3.976 | -2.728 | -1.346 |
| 8 | 3.820 | -3.901 | -1.113 |
| 7 | 2.930 | -1.846 | -1.518 |
| 1 | 1.956 | -2.307 | -1.379 |
| 6 | 3.066 | -0.514 | -1.663 |
| 7 | 2.002 | 0.252 | -1.693 |
| 1 | 4.528 | 1.075 | -1.978 |
| 1 | 6.454 | -0.498 | -1.738 |
| 1 | 1.045 | -0.116 | -1.603 |
| 1 | 2.136 | 1.252 | -1.596 |
| 1 | 6.010 | -2.784 | -1.349 |

T•A-T/T•A-T

| 8 | -3.292 | -2.381 | 2.031 |
| --- | --- | --- | --- |
| 8 | -1.369 | 1.729 | 1.922 |
| 7 | -4.644 | -0.547 | 2.041 |
| 7 | -2.351 | -0.307 | 2.042 |
| 7 | 1.338 | 0.463 | 1.555 |
| 7 | 3.835 | -1.325 | 1.153 |
| 7 | 3.600 | -3.531 | 1.149 |
| 7 | 1.200 | -3.701 | 1.517 |
| 7 | 0.195 | -1.539 | 1.731 |
| 6 | -3.840 | 3.148 | 1.900 |
| 6 | -2.396 | 1.069 | 1.974 |
| 6 | -3.732 | 1.658 | 1.976 |
| 6 | -4.779 | 0.818 | 2.005 |
| 6 | -3.414 | -1.175 | 2.035 |
| 6 | 4.445 | -2.471 | 1.017 |
| 6 | 1.346 | -0.866 | 1.553 |
| 6 | 2.509 | -1.647 | 1.365 |
| 6 | 2.341 | -3.023 | 1.358 |
| 6 | 0.179 | -2.876 | 1.701 |
| 1 | -5.800 | 1.179 | 2.000 |
| 1 | -5.442 | -1.140 | 1.874 |
| 1 | -1.414 | -0.747 | 1.982 |
| 1 | 0.455 | 0.952 | 1.671 |
| 1 | 2.193 | 0.996 | 1.474 |
| 1 | 5.497 | -2.579 | 0.810 |
| 1 | 3.825 | -4.501 | 1.023 |
| 1 | -0.800 | -3.320 | 1.841 |
| 1 | -3.318 | 3.615 | 2.734 |
| 1 | -4.884 | 3.461 | 1.910 |
| 1 | -3.371 | 3.503 | 0.981 |
| 6 | 5.283 | 4.717 | 1.037 |
| 6 | 5.803 | 3.338 | 0.782 |
| 6 | 7.036 | 3.060 | 0.331 |
| 7 | 7.460 | 1.771 | 0.092 |
| 1 | 8.377 | 1.581 | -0.274 |
| 6 | 6.663 | 0.666 | 0.299 |
| 8 | 7.021 | -0.459 | -0.005 |
| 7 | 5.444 | 0.954 | 0.842 |
| 1 | 4.805 | 0.142 | 0.986 |
| 6 | 4.903 | 2.213 | 1.040 |
| 8 | 3.749 | 2.345 | 1.401 |
| 1 | 7.762 | 3.833 | 0.120 |
| 1 | 6.049 | 5.465 | 0.840 |
| 1 | 4.950 | 4.809 | 2.070 |
| 1 | 4.418 | 4.917 | 0.405 |
| 8 | -6.901 | -0.926 | -0.041 |
| 8 | -3.996 | 2.237 | -1.504 |
| 7 | -7.660 | 1.190 | -0.368 |
| 7 | -5.492 | 0.639 | -0.923 |
| 7 | -1.704 | 0.246 | -1.316 |
| 7 | 0.094 | -2.252 | -1.513 |
| 7 | -0.811 | -4.269 | -1.328 |
| 7 | -3.148 | -3.655 | -1.038 |
| 7 | -3.433 | -1.277 | -1.045 |
| 6 | -5.886 | 4.357 | -1.425 |
| 6 | -5.140 | 1.948 | -1.197 |
| 6 | -6.213 | 2.937 | -1.088 |
| 6 | -7.420 | 2.507 | -0.688 |
| 6 | -6.690 | 0.209 | -0.427 |
| 6 | 0.328 | -3.535 | -1.499 |
| 6 | -2.124 | -1.013 | -1.233 |
| 6 | -1.266 | -2.131 | -1.325 |
| 6 | -1.852 | -3.382 | -1.210 |
| 6 | -3.859 | -2.548 | -0.956 |
| 1 | -8.266 | 3.172 | -0.585 |
| 1 | -8.558 | 0.887 | -0.034 |
| 1 | -4.719 | -0.072 | -0.965 |
| 1 | -2.390 | 0.991 | -1.328 |
| 1 | -0.729 | 0.472 | -1.472 |
| 1 | 1.307 | -3.972 | -1.604 |
| 1 | -0.882 | -5.267 | -1.234 |
| 1 | -4.924 | -2.658 | -0.793 |
| 1 | -5.104 | 4.731 | -0.765 |
| 1 | -6.765 | 4.992 | -1.332 |
| 1 | -5.503 | 4.424 | -2.443 |
| 6 | 3.314 | 3.044 | -2.044 |
| 6 | 3.424 | 1.553 | -2.027 |
| 6 | 4.580 | 0.872 | -2.069 |
| 7 | 4.641 | -0.501 | -2.016 |
| 1 | 5.527 | -0.962 | -1.876 |
| 6 | 3.520 | -1.294 | -1.900 |
| 8 | 3.578 | -2.506 | -1.822 |
| 7 | 2.346 | -0.591 | -1.884 |
| 1 | 1.484 | -1.157 | -1.770 |
| 6 | 2.189 | 0.781 | -1.916 |
| 8 | 1.080 | 1.285 | -1.859 |
| 1 | 5.537 | 1.371 | -2.146 |
| 1 | 4.298 | 3.505 | -2.130 |
| 1 | 2.838 | 3.385 | -1.124 |
| 1 | 2.690 | 3.373 | -2.875 |

rT•A-T/rT•A-T

| 8 | -4.417 | -3.196 | 0.714 |
| --- | --- | --- | --- |
| 8 | -3.820 | 1.195 | 1.726 |
| 7 | -6.246 | -1.871 | 0.971 |
| 7 | -4.129 | -1.002 | 1.262 |
| 7 | -0.825 | 0.748 | 1.677 |
| 7 | 2.107 | -0.186 | 1.915 |
| 7 | 2.575 | -2.357 | 1.780 |
| 7 | 0.322 | -3.254 | 1.546 |
| 7 | -1.323 | -1.512 | 1.569 |
| 6 | -6.595 | 1.761 | 1.943 |
| 6 | -4.594 | 0.266 | 1.533 |
| 6 | -6.047 | 0.421 | 1.566 |
| 6 | -6.795 | -0.653 | 1.264 |
| 6 | -4.885 | -2.108 | 0.958 |
| 6 | 3.053 | -1.085 | 1.910 |
| 6 | -0.420 | -0.519 | 1.669 |
| 6 | 0.937 | -0.904 | 1.764 |
| 6 | 1.209 | -2.262 | 1.684 |
| 6 | -0.917 | -2.788 | 1.508 |
| 1 | -7.876 | -0.613 | 1.246 |
| 1 | -6.816 | -2.647 | 0.683 |
| 1 | -3.103 | -1.161 | 1.313 |
| 1 | -1.816 | 0.949 | 1.596 |
| 1 | -0.161 | 1.482 | 1.473 |
| 1 | 4.104 | -0.862 | 2.006 |
| 1 | 3.133 | -3.174 | 1.591 |
| 1 | -1.715 | -3.512 | 1.393 |
| 1 | -6.123 | 2.542 | 1.348 |
| 1 | -6.374 | 1.986 | 2.987 |
| 1 | -7.675 | 1.795 | 1.800 |
| 6 | 6.422 | 3.955 | 0.389 |
| 6 | 4.958 | 3.642 | 0.355 |
| 6 | 4.057 | 4.307 | -0.391 |
| 7 | 2.721 | 4.016 | -0.359 |
| 1 | 2.066 | 4.472 | -0.970 |
| 6 | 2.188 | 3.017 | 0.428 |
| 8 | 1.003 | 2.758 | 0.424 |
| 7 | 3.104 | 2.371 | 1.214 |
| 1 | 2.731 | 1.531 | 1.686 |
| 6 | 4.476 | 2.543 | 1.196 |
| 8 | 5.213 | 1.806 | 1.825 |
| 1 | 4.342 | 5.115 | -1.050 |
| 1 | 6.666 | 4.761 | -0.302 |
| 1 | 7.020 | 3.078 | 0.136 |
| 1 | 6.723 | 4.249 | 1.394 |
| 8 | -5.392 | -0.368 | -1.681 |
| 8 | -1.888 | 2.496 | -1.242 |
| 7 | -5.807 | 1.853 | -1.441 |
| 7 | -3.638 | 1.084 | -1.510 |
| 7 | 0.119 | 0.257 | -1.515 |
| 7 | 1.607 | -2.450 | -1.443 |
| 7 | 0.447 | -4.337 | -1.566 |
| 7 | -1.802 | -3.437 | -1.738 |
| 7 | -1.794 | -1.043 | -1.677 |
| 6 | -3.506 | 4.799 | -0.888 |
| 6 | -3.097 | 2.336 | -1.290 |
| 6 | -4.054 | 3.432 | -1.141 |
| 6 | -5.358 | 3.136 | -1.230 |
| 6 | -4.967 | 0.763 | -1.554 |
| 6 | 1.671 | -3.753 | -1.422 |
| 6 | -0.454 | -0.942 | -1.595 |
| 6 | 0.267 | -2.156 | -1.582 |
| 6 | -0.476 | -3.326 | -1.649 |
| 6 | -2.380 | -2.248 | -1.738 |
| 1 | -6.129 | 3.888 | -1.136 |
| 1 | -6.788 | 1.638 | -1.453 |
| 1 | -2.958 | 0.295 | -1.588 |
| 1 | -0.464 | 1.082 | -1.419 |
| 1 | 1.123 | 0.354 | -1.455 |
| 1 | 2.583 | -4.313 | -1.295 |
| 1 | 0.229 | -5.315 | -1.500 |
| 1 | -3.462 | -2.229 | -1.788 |
| 1 | -2.842 | 5.097 | -1.699 |
| 1 | -2.914 | 4.798 | 0.027 |
| 1 | -4.307 | 5.530 | -0.795 |
| 6 | 7.397 | -2.228 | 0.287 |
| 6 | 6.314 | -1.368 | -0.284 |
| 6 | 6.453 | -0.063 | -0.572 |
| 7 | 5.431 | 0.690 | -1.093 |
| 1 | 5.525 | 1.687 | -1.198 |
| 6 | 4.157 | 0.201 | -1.272 |
| 8 | 3.227 | 0.913 | -1.608 |
| 7 | 4.041 | -1.140 | -1.039 |
| 1 | 3.097 | -1.556 | -1.190 |
| 6 | 5.008 | -1.976 | -0.511 |
| 8 | 4.741 | -3.138 | -0.238 |
| 1 | 7.379 | 0.467 | -0.404 |
| 1 | 8.318 | -1.661 | 0.411 |
| 1 | 7.588 | -3.081 | -0.364 |
| 1 | 7.095 | -2.627 | 1.256 |

rG•G-C/rG•G-C

| 7 | -2.542 | -3.212 | 1.532 |
| --- | --- | --- | --- |
| 8 | -0.740 | 0.982 | 1.953 |
| 7 | -1.553 | -1.141 | 1.743 |
| 6 | -1.403 | -2.501 | 1.617 |
| 7 | -0.240 | -3.108 | 1.595 |
| 6 | 0.793 | -2.259 | 1.752 |
| 6 | 0.743 | -0.885 | 1.947 |
| 6 | -0.513 | -0.237 | 1.893 |
| 7 | 2.121 | -2.567 | 1.777 |
| 6 | 2.806 | -1.392 | 1.974 |
| 7 | 2.009 | -0.367 | 2.094 |
| 1 | -3.450 | -2.771 | 1.421 |
| 1 | -2.437 | -4.186 | 1.321 |
| 1 | -2.496 | -0.733 | 1.700 |
| 1 | 2.538 | -3.424 | 1.434 |
| 1 | 3.883 | -1.343 | 2.017 |
| 8 | -5.151 | -1.880 | 1.289 |
| 7 | -3.284 | 2.217 | 1.987 |
| 7 | -4.188 | 0.153 | 1.651 |
| 6 | -5.675 | 2.047 | 1.891 |
| 6 | -5.246 | -0.677 | 1.494 |
| 7 | -6.522 | -0.116 | 1.588 |
| 6 | -6.723 | 1.209 | 1.754 |
| 6 | -4.365 | 1.453 | 1.844 |
| 1 | -5.811 | 3.105 | 2.044 |
| 1 | -7.286 | -0.749 | 1.424 |
| 1 | -7.751 | 1.541 | 1.775 |
| 1 | -2.352 | 1.788 | 1.974 |
| 1 | -3.383 | 3.193 | 2.192 |
| 6 | 6.602 | 3.554 | -1.071 |
| 7 | 5.334 | 4.041 | -1.276 |
| 1 | 5.025 | 4.650 | -2.013 |
| 6 | 4.528 | 3.468 | -0.334 |
| 6 | 5.376 | 2.689 | 0.430 |
| 7 | 6.663 | 2.737 | -0.061 |
| 6 | 4.794 | 1.812 | 1.406 |
| 8 | 5.319 | 0.944 | 2.074 |
| 7 | 3.407 | 2.035 | 1.501 |
| 1 | 2.902 | 1.323 | 2.037 |
| 6 | 2.671 | 2.826 | 0.676 |
| 7 | 3.183 | 3.585 | -0.269 |
| 7 | 1.332 | 2.825 | 0.875 |
| 1 | 7.431 | 3.842 | -1.696 |
| 1 | 0.857 | 2.096 | 1.396 |
| 1 | 0.803 | 3.303 | 0.171 |
| 7 | -4.936 | -1.971 | -1.835 |
| 8 | -0.870 | 0.109 | -1.410 |
| 7 | -2.854 | -1.011 | -1.569 |
| 6 | -3.610 | -2.150 | -1.690 |
| 7 | -3.101 | -3.363 | -1.708 |
| 6 | -1.761 | -3.358 | -1.628 |
| 6 | -0.906 | -2.270 | -1.566 |
| 6 | -1.466 | -0.969 | -1.513 |
| 7 | -0.936 | -4.447 | -1.581 |
| 6 | 0.356 | -3.981 | -1.492 |
| 7 | 0.403 | -2.683 | -1.493 |
| 1 | -5.358 | -1.075 | -1.612 |
| 1 | -5.487 | -2.797 | -1.685 |
| 1 | -3.326 | -0.102 | -1.493 |
| 1 | -1.233 | -5.407 | -1.606 |
| 1 | 1.220 | -4.621 | -1.413 |
| 8 | -6.129 | 0.718 | -1.344 |
| 7 | -2.024 | 2.694 | -1.312 |
| 7 | -4.061 | 1.676 | -1.353 |
| 6 | -3.967 | 4.095 | -1.429 |
| 6 | -5.411 | 1.710 | -1.393 |
| 7 | -6.019 | 2.965 | -1.504 |
| 6 | -5.311 | 4.121 | -1.514 |
| 6 | -3.347 | 2.795 | -1.365 |
| 1 | -3.382 | 5.000 | -1.437 |
| 1 | -7.023 | 2.958 | -1.572 |
| 1 | -5.879 | 5.036 | -1.593 |
| 1 | -1.584 | 1.767 | -1.298 |
| 1 | -1.457 | 3.516 | -1.408 |
| 6 | 7.167 | -1.487 | 0.064 |
| 7 | 6.629 | -0.312 | -0.405 |
| 1 | 6.997 | 0.627 | -0.289 |
| 6 | 5.349 | -0.593 | -0.767 |
| 6 | 5.193 | -1.956 | -0.531 |
| 7 | 6.349 | -2.495 | -0.007 |
| 6 | 3.904 | -2.531 | -0.747 |
| 8 | 3.508 | -3.677 | -0.541 |
| 7 | 2.998 | -1.566 | -1.207 |
| 1 | 2.040 | -1.908 | -1.350 |
| 6 | 3.255 | -0.240 | -1.384 |
| 7 | 4.433 | 0.300 | -1.200 |
| 7 | 2.213 | 0.532 | -1.794 |
| 1 | 8.174 | -1.530 | 0.443 |
| 1 | 1.264 | 0.229 | -1.619 |
| 1 | 2.395 | 1.520 | -1.725 |

rA•A-T/rA•A-T

| 8 | -5.342 | -0.242 | -1.625 |
| --- | --- | --- | --- |
| 8 | -1.436 | 2.072 | -1.453 |
| 8 | -3.207 | 1.605 | 1.576 |
| 8 | -4.685 | -2.683 | 1.259 |
| 7 | -5.400 | 2.025 | -1.775 |
| 7 | -3.381 | 0.928 | -1.592 |
| 7 | 0.187 | -0.593 | -1.553 |
| 7 | 1.122 | -3.539 | -1.360 |
| 7 | -0.411 | -5.148 | -1.217 |
| 7 | -2.436 | -3.822 | -1.317 |
| 7 | -1.949 | -1.487 | -1.493 |
| 7 | 6.124 | -2.383 | -0.407 |
| 7 | 6.687 | -0.051 | -0.443 |
| 7 | 4.829 | 1.314 | -1.217 |
| 7 | 3.296 | -0.275 | -1.545 |
| 7 | 4.070 | -3.219 | -1.041 |
| 7 | 4.153 | 1.467 | 2.017 |
| 7 | 1.645 | 2.601 | 0.654 |
| 7 | 1.773 | 4.550 | -0.414 |
| 7 | 4.083 | 5.145 | 0.082 |
| 7 | 5.177 | 3.435 | 1.367 |
| 7 | -1.315 | -1.557 | 1.731 |
| 7 | -0.069 | -3.597 | 1.847 |
| 7 | 2.325 | -3.172 | 1.888 |
| 7 | 2.331 | -0.948 | 1.781 |
| 7 | -0.361 | 0.549 | 1.615 |
| 7 | -3.955 | -0.530 | 1.439 |
| 7 | -6.212 | -1.000 | 1.326 |
| 6 | -2.682 | 4.630 | -1.517 |
| 6 | -2.658 | 2.096 | -1.537 |
| 6 | -3.426 | 3.336 | -1.605 |
| 6 | -4.758 | 3.237 | -1.735 |
| 6 | -4.744 | 0.813 | -1.659 |
| 6 | 0.914 | -4.819 | -1.235 |
| 6 | -0.612 | -1.656 | -1.490 |
| 6 | -0.142 | -2.986 | -1.412 |
| 6 | -1.112 | -3.977 | -1.321 |
| 6 | -2.767 | -2.547 | -1.406 |
| 6 | 4.881 | -2.167 | -0.860 |
| 6 | 4.479 | -0.841 | -1.109 |
| 6 | 5.442 | 0.136 | -0.886 |
| 6 | 6.935 | -1.334 | -0.231 |
| 6 | 3.548 | 1.003 | -1.586 |
| 6 | 1.002 | 3.465 | -0.082 |
| 6 | 5.110 | 4.608 | 0.729 |
| 6 | 3.006 | 4.360 | 0.158 |
| 6 | 2.903 | 3.142 | 0.817 |
| 6 | 4.081 | 2.662 | 1.424 |
| 6 | -1.186 | -2.889 | 1.794 |
| 6 | 1.006 | -2.802 | 1.837 |
| 6 | 1.031 | -1.417 | 1.771 |
| 6 | -0.223 | -0.769 | 1.702 |
| 6 | 3.065 | -2.023 | 1.842 |
| 6 | -4.923 | -1.499 | 1.332 |
| 6 | -6.501 | 0.334 | 1.414 |
| 6 | -5.546 | 1.273 | 1.528 |
| 6 | -4.154 | 0.829 | 1.522 |
| 6 | -5.811 | 2.738 | 1.680 |
| 1 | -5.398 | 4.105 | -1.805 |
| 1 | -6.402 | 1.965 | -1.825 |
| 1 | -2.833 | 0.034 | -1.537 |
| 1 | -0.234 | 0.326 | -1.510 |
| 1 | 1.196 | -0.692 | -1.517 |
| 1 | 1.685 | -5.570 | -1.164 |
| 1 | -0.817 | -6.056 | -1.077 |
| 1 | -3.824 | -2.312 | -1.407 |
| 1 | 7.926 | -1.574 | 0.134 |
| 1 | 2.841 | 1.764 | -1.872 |
| 1 | 4.469 | -4.127 | -0.887 |
| 1 | 3.094 | -3.115 | -1.300 |
| 1 | 5.227 | 2.231 | -1.097 |
| 1 | -1.901 | 4.669 | -2.277 |
| 1 | -2.203 | 4.721 | -0.541 |
| 1 | -3.353 | 5.476 | -1.655 |
| 1 | -6.872 | 2.956 | 1.561 |
| 1 | -5.492 | 3.083 | 2.664 |
| 1 | -5.238 | 3.304 | 0.947 |
| 1 | 1.500 | 5.345 | -0.966 |
| 1 | 3.395 | 0.792 | 1.949 |
| 1 | 5.061 | 1.175 | 2.332 |
| 1 | -0.010 | 3.340 | -0.436 |
| 1 | 6.025 | 5.186 | 0.731 |
| 1 | -2.122 | -3.436 | 1.782 |
| 1 | 2.670 | -4.114 | 1.907 |
| 1 | 4.144 | -2.043 | 1.852 |
| 1 | 0.446 | 1.139 | 1.445 |
| 1 | -1.292 | 0.948 | 1.563 |
| 1 | -2.976 | -0.884 | 1.498 |
| 1 | -6.933 | -1.684 | 1.174 |
| 1 | -7.553 | 0.584 | 1.398 |

G4∙∙∙Na^+^

| 11 | 0.000 | 0.000 | -0.005 |
| --- | --- | --- | --- |
| 8 | -0.559 | -2.219 | -0.002 |
| 8 | 2.219 | -0.559 | -0.001 |
| 8 | -2.219 | 0.559 | 0.000 |
| 8 | 0.559 | 2.219 | -0.001 |
| 7 | -5.149 | -2.177 | 0.010 |
| 7 | -4.162 | -4.245 | 0.006 |
| 7 | -2.838 | -2.279 | 0.003 |
| 7 | -0.747 | -5.302 | 0.000 |
| 7 | -2.773 | -6.216 | 0.003 |
| 7 | 2.177 | -5.149 | -0.001 |
| 7 | 4.246 | -4.162 | -0.004 |
| 7 | 2.279 | -2.838 | -0.002 |
| 7 | 5.302 | -0.747 | -0.003 |
| 7 | 6.216 | -2.773 | -0.006 |
| 7 | -2.178 | 5.149 | 0.000 |
| 7 | -4.246 | 4.162 | -0.005 |
| 7 | -2.279 | 2.838 | -0.001 |
| 7 | -5.302 | 0.747 | -0.003 |
| 7 | -6.216 | 2.773 | -0.007 |
| 7 | 5.149 | 2.177 | 0.009 |
| 7 | 4.162 | 4.246 | 0.006 |
| 7 | 2.838 | 2.279 | 0.003 |
| 7 | 0.747 | 5.302 | 0.001 |
| 7 | 2.773 | 6.216 | 0.004 |
| 6 | -4.051 | -2.932 | 0.006 |
| 6 | -1.598 | -2.900 | 0.001 |
| 6 | -1.709 | -4.311 | 0.002 |
| 6 | -2.982 | -4.867 | 0.004 |
| 6 | -1.415 | -6.417 | 0.001 |
| 6 | 2.933 | -4.051 | -0.003 |
| 6 | 2.900 | -1.598 | -0.001 |
| 6 | 4.311 | -1.709 | -0.002 |
| 6 | 4.868 | -2.982 | -0.004 |
| 6 | 6.417 | -1.415 | -0.005 |
| 6 | -2.933 | 4.050 | -0.003 |
| 6 | -2.900 | 1.598 | -0.001 |
| 6 | -4.311 | 1.709 | -0.002 |
| 6 | -4.868 | 2.981 | -0.005 |
| 6 | -6.417 | 1.414 | -0.006 |
| 6 | 4.051 | 2.932 | 0.005 |
| 6 | 1.598 | 2.900 | 0.001 |
| 6 | 1.709 | 4.311 | 0.002 |
| 6 | 2.982 | 4.867 | 0.004 |
| 6 | 1.415 | 6.417 | 0.002 |
| 1 | -6.020 | -2.675 | 0.008 |
| 1 | -5.140 | -1.157 | 0.004 |
| 1 | -0.987 | -7.405 | 0.000 |
| 1 | -2.814 | -1.254 | 0.003 |
| 1 | 2.675 | -6.020 | -0.005 |
| 1 | 1.157 | -5.140 | -0.004 |
| 1 | 7.406 | -0.987 | -0.005 |
| 1 | 1.255 | -2.814 | -0.001 |
| 1 | -2.675 | 6.020 | -0.005 |
| 1 | -1.158 | 5.140 | -0.002 |
| 1 | -7.405 | 0.987 | -0.006 |
| 1 | -1.255 | 2.814 | 0.000 |
| 1 | 6.021 | 2.674 | 0.007 |
| 1 | 5.140 | 1.157 | 0.002 |
| 1 | 0.988 | 7.406 | 0.002 |
| 1 | 2.814 | 1.255 | 0.003 |
| 1 | -6.917 | 3.494 | -0.008 |
| 1 | -3.495 | -6.917 | 0.006 |
| 1 | 6.917 | -3.495 | -0.006 |
| 1 | 3.495 | 6.917 | 0.006 |

G4∙∙∙K^+^

| 19 | 0.000 | 0.001 | 1.387 |
| --- | --- | --- | --- |
| 8 | 2.107 | 1.166 | 0.390 |
| 8 | -1.167 | 2.107 | 0.391 |
| 8 | 1.167 | -2.108 | 0.394 |
| 8 | -2.108 | -1.166 | 0.392 |
| 7 | 5.032 | -2.337 | -0.134 |
| 7 | 5.937 | -0.237 | -0.281 |
| 7 | 3.612 | -0.523 | 0.092 |
| 7 | 4.516 | 3.037 | -0.049 |
| 7 | 6.509 | 2.106 | -0.374 |
| 7 | 2.337 | 5.028 | -0.158 |
| 7 | 0.237 | 5.932 | -0.306 |
| 7 | 0.522 | 3.610 | 0.082 |
| 7 | -3.038 | 4.514 | -0.059 |
| 7 | -2.106 | 6.505 | -0.399 |
| 7 | -2.337 | -5.028 | -0.157 |
| 7 | -0.237 | -5.931 | -0.308 |
| 7 | -0.522 | -3.610 | 0.084 |
| 7 | 3.038 | -4.514 | -0.060 |
| 7 | 2.106 | -6.504 | -0.403 |
| 7 | -5.032 | 2.337 | -0.139 |
| 7 | -5.936 | 0.237 | -0.284 |
| 7 | -3.612 | 0.523 | 0.091 |
| 7 | -4.516 | -3.037 | -0.046 |
| 7 | -6.509 | -2.106 | -0.374 |
| 6 | 4.883 | -1.012 | -0.114 |
| 6 | 3.278 | 0.824 | 0.164 |
| 6 | 4.402 | 1.662 | -0.034 |
| 6 | 5.638 | 1.064 | -0.235 |
| 6 | 5.779 | 3.264 | -0.255 |
| 6 | 1.011 | 4.880 | -0.134 |
| 6 | -0.825 | 3.276 | 0.158 |
| 6 | -1.662 | 4.400 | -0.045 |
| 6 | -1.065 | 5.634 | -0.256 |
| 6 | -3.265 | 5.776 | -0.273 |
| 6 | -1.011 | -4.880 | -0.133 |
| 6 | 0.825 | -3.276 | 0.160 |
| 6 | 1.662 | -4.399 | -0.046 |
| 6 | 1.064 | -5.634 | -0.258 |
| 6 | 3.264 | -5.776 | -0.276 |
| 6 | -4.883 | 1.011 | -0.117 |
| 6 | -3.278 | -0.825 | 0.165 |
| 6 | -4.402 | -1.662 | -0.033 |
| 6 | -5.638 | -1.064 | -0.236 |
| 6 | -5.780 | -3.264 | -0.253 |
| 1 | 5.965 | -2.666 | -0.304 |
| 1 | 4.272 | -3.011 | -0.049 |
| 1 | 6.239 | 4.235 | -0.330 |
| 1 | 2.834 | -1.182 | 0.186 |
| 1 | 2.666 | 5.960 | -0.332 |
| 1 | 3.011 | 4.269 | -0.061 |
| 1 | -4.235 | 6.236 | -0.349 |
| 1 | 1.181 | 2.832 | 0.179 |
| 1 | -2.666 | -5.960 | -0.332 |
| 1 | -3.011 | -4.270 | -0.058 |
| 1 | 4.235 | -6.235 | -0.354 |
| 1 | -1.182 | -2.832 | 0.182 |
| 1 | -5.965 | 2.666 | -0.309 |
| 1 | -4.272 | 3.011 | -0.052 |
| 1 | -6.239 | -4.235 | -0.327 |
| 1 | -2.834 | 1.182 | 0.185 |
| 1 | -2.021 | 7.493 | -0.564 |
| 1 | -7.498 | -2.021 | -0.533 |
| 1 | 2.021 | -7.492 | -0.569 |
| 1 | 7.499 | 2.020 | -0.532 |

G4∙∙∙Na^+^∙∙∙G4

| 8 | -1.180 | 1.972 | 1.278 |
| --- | --- | --- | --- |
| 8 | -1.972 | -1.180 | 1.278 |
| 8 | 1.972 | 1.180 | 1.278 |
| 8 | 1.180 | -1.972 | 1.278 |
| 7 | 2.144 | 5.112 | 1.714 |
| 7 | -0.002 | 5.909 | 1.746 |
| 7 | 0.419 | 3.583 | 1.518 |
| 7 | -3.181 | 4.306 | 1.442 |
| 7 | -2.372 | 6.362 | 1.672 |
| 7 | -5.113 | 2.144 | 1.713 |
| 7 | -5.909 | -0.002 | 1.744 |
| 7 | -3.584 | 0.419 | 1.517 |
| 7 | -4.307 | -3.181 | 1.440 |
| 7 | -6.362 | -2.373 | 1.669 |
| 7 | 5.113 | -2.144 | 1.713 |
| 7 | 5.909 | 0.002 | 1.745 |
| 7 | 3.583 | -0.419 | 1.518 |
| 7 | 4.307 | 3.181 | 1.440 |
| 7 | 6.362 | 2.373 | 1.669 |
| 7 | -2.144 | -5.112 | 1.714 |
| 7 | 0.002 | -5.909 | 1.746 |
| 7 | -0.419 | -3.583 | 1.518 |
| 7 | 3.181 | -4.306 | 1.442 |
| 7 | 2.372 | -6.362 | 1.672 |
| 6 | 0.827 | 4.886 | 1.661 |
| 6 | -0.904 | 3.163 | 1.425 |
| 6 | -1.805 | 4.262 | 1.512 |
| 6 | -1.282 | 5.536 | 1.656 |
| 6 | -3.484 | 5.567 | 1.537 |
| 6 | -4.886 | 0.827 | 1.660 |
| 6 | -3.164 | -0.904 | 1.424 |
| 6 | -4.263 | -1.805 | 1.510 |
| 6 | -5.537 | -1.282 | 1.654 |
| 6 | -5.568 | -3.484 | 1.535 |
| 6 | 4.886 | -0.827 | 1.660 |
| 6 | 3.163 | 0.904 | 1.425 |
| 6 | 4.262 | 1.805 | 1.510 |
| 6 | 5.537 | 1.282 | 1.655 |
| 6 | 5.568 | 3.484 | 1.535 |
| 6 | -0.827 | -4.886 | 1.661 |
| 6 | 0.904 | -3.163 | 1.425 |
| 6 | 1.805 | -4.262 | 1.512 |
| 6 | 1.282 | -5.536 | 1.656 |
| 6 | 3.484 | -5.567 | 1.538 |
| 1 | 2.415 | 6.069 | 1.843 |
| 1 | 2.852 | 4.386 | 1.606 |
| 1 | -4.480 | 5.977 | 1.516 |
| 1 | 1.109 | 2.825 | 1.454 |
| 1 | -6.070 | 2.415 | 1.842 |
| 1 | -4.387 | 2.852 | 1.606 |
| 1 | -5.978 | -4.480 | 1.513 |
| 1 | -2.826 | 1.109 | 1.454 |
| 1 | 6.069 | -2.415 | 1.842 |
| 1 | 4.387 | -2.852 | 1.606 |
| 1 | 5.978 | 4.480 | 1.514 |
| 1 | 2.826 | -1.109 | 1.454 |
| 1 | -2.415 | -6.069 | 1.843 |
| 1 | -2.852 | -4.386 | 1.606 |
| 1 | 4.479 | -5.977 | 1.516 |
| 1 | -1.109 | -2.825 | 1.454 |
| 1 | 7.361 | 2.345 | 1.780 |
| 1 | -2.345 | 7.360 | 1.782 |
| 1 | -7.361 | -2.345 | 1.779 |
| 1 | 2.345 | -7.360 | 1.783 |
| 11 | 0.000 | 0.000 | -0.006 |
| 8 | 0.561 | 2.229 | -1.279 |
| 8 | -2.228 | 0.561 | -1.279 |
| 8 | 2.229 | -0.561 | -1.279 |
| 8 | -0.561 | -2.229 | -1.279 |
| 7 | 5.131 | 2.097 | -1.716 |
| 7 | 4.178 | 4.178 | -1.746 |
| 7 | 2.831 | 2.236 | -1.519 |
| 7 | 0.798 | 5.294 | -1.438 |
| 7 | 2.824 | 6.175 | -1.668 |
| 7 | -2.097 | 5.132 | -1.714 |
| 7 | -4.178 | 4.179 | -1.745 |
| 7 | -2.236 | 2.831 | -1.519 |
| 7 | -5.294 | 0.798 | -1.440 |
| 7 | -6.175 | 2.824 | -1.669 |
| 7 | 2.097 | -5.132 | -1.714 |
| 7 | 4.178 | -4.179 | -1.745 |
| 7 | 2.236 | -2.831 | -1.519 |
| 7 | 5.294 | -0.798 | -1.440 |
| 7 | 6.175 | -2.824 | -1.669 |
| 7 | -5.131 | -2.097 | -1.716 |
| 7 | -4.178 | -4.178 | -1.746 |
| 7 | -2.831 | -2.236 | -1.520 |
| 7 | -0.798 | -5.294 | -1.439 |
| 7 | -2.823 | -6.175 | -1.669 |
| 6 | 4.041 | 2.868 | -1.662 |
| 6 | 1.599 | 2.875 | -1.425 |
| 6 | 1.739 | 4.289 | -1.510 |
| 6 | 3.010 | 4.820 | -1.655 |
| 6 | 1.476 | 6.400 | -1.533 |
| 6 | -2.868 | 4.041 | -1.661 |
| 6 | -2.875 | 1.599 | -1.425 |
| 6 | -4.289 | 1.739 | -1.511 |
| 6 | -4.820 | 3.010 | -1.655 |
| 6 | -6.400 | 1.476 | -1.534 |
| 6 | 2.868 | -4.041 | -1.661 |
| 6 | 2.875 | -1.599 | -1.425 |
| 6 | 4.289 | -1.739 | -1.511 |
| 6 | 4.820 | -3.010 | -1.655 |
| 6 | 6.400 | -1.476 | -1.534 |
| 6 | -4.041 | -2.868 | -1.662 |
| 6 | -1.599 | -2.875 | -1.425 |
| 6 | -1.739 | -4.289 | -1.510 |
| 6 | -3.010 | -4.820 | -1.655 |
| 6 | -1.476 | -6.400 | -1.533 |
| 1 | 6.000 | 2.581 | -1.844 |
| 1 | 5.118 | 1.083 | -1.606 |
| 1 | 1.062 | 7.394 | -1.510 |
| 1 | 2.783 | 1.212 | -1.458 |
| 1 | -2.581 | 6.000 | -1.842 |
| 1 | -1.083 | 5.119 | -1.604 |
| 1 | -7.394 | 1.062 | -1.512 |
| 1 | -1.212 | 2.783 | -1.457 |
| 1 | 2.581 | -6.000 | -1.842 |
| 1 | 1.083 | -5.119 | -1.604 |
| 1 | 7.394 | -1.062 | -1.512 |
| 1 | 1.213 | -2.783 | -1.457 |
| 1 | -6.000 | -2.581 | -1.845 |
| 1 | -5.118 | -1.083 | -1.606 |
| 1 | -1.062 | -7.394 | -1.510 |
| 1 | -2.783 | -1.213 | -1.458 |
| 1 | 6.862 | -3.549 | -1.779 |
| 1 | 3.549 | 6.862 | -1.779 |
| 1 | -6.861 | 3.549 | -1.780 |
| 1 | -3.549 | -6.862 | -1.780 |

G4∙∙∙K^+^∙∙∙G4

| 8 | -2.367 | -0.392 | -1.382 |
| --- | --- | --- | --- |
| 8 | -0.392 | 2.367 | -1.382 |
| 8 | 0.392 | -2.367 | -1.382 |
| 8 | 2.367 | 0.392 | -1.382 |
| 7 | -2.425 | -4.975 | -1.696 |
| 7 | -4.468 | -3.935 | -1.716 |
| 7 | -2.469 | -2.667 | -1.551 |
| 7 | -5.431 | -0.498 | -1.487 |
| 7 | -6.405 | -2.490 | -1.648 |
| 7 | -4.975 | 2.425 | -1.697 |
| 7 | -3.935 | 4.468 | -1.717 |
| 7 | -2.667 | 2.469 | -1.551 |
| 7 | -0.498 | 5.431 | -1.488 |
| 7 | -2.490 | 6.405 | -1.648 |
| 7 | 4.975 | -2.425 | -1.697 |
| 7 | 3.935 | -4.468 | -1.716 |
| 7 | 2.667 | -2.468 | -1.551 |
| 7 | 0.498 | -5.431 | -1.488 |
| 7 | 2.490 | -6.405 | -1.648 |
| 7 | 2.425 | 4.975 | -1.696 |
| 7 | 4.468 | 3.935 | -1.716 |
| 7 | 2.469 | 2.667 | -1.551 |
| 7 | 5.431 | 0.498 | -1.487 |
| 7 | 6.405 | 2.490 | -1.648 |
| 6 | -3.152 | -3.852 | -1.656 |
| 6 | -3.057 | -1.407 | -1.486 |
| 6 | -4.474 | -1.488 | -1.545 |
| 6 | -5.061 | -2.738 | -1.647 |
| 6 | -6.567 | -1.129 | -1.548 |
| 6 | -3.852 | 3.152 | -1.656 |
| 6 | -1.407 | 3.057 | -1.486 |
| 6 | -1.488 | 4.474 | -1.545 |
| 6 | -2.738 | 5.061 | -1.647 |
| 6 | -1.129 | 6.567 | -1.549 |
| 6 | 3.852 | -3.152 | -1.656 |
| 6 | 1.407 | -3.057 | -1.486 |
| 6 | 1.488 | -4.474 | -1.545 |
| 6 | 2.738 | -5.061 | -1.647 |
| 6 | 1.129 | -6.567 | -1.549 |
| 6 | 3.152 | 3.852 | -1.656 |
| 6 | 3.057 | 1.407 | -1.486 |
| 6 | 4.474 | 1.488 | -1.545 |
| 6 | 5.061 | 2.738 | -1.647 |
| 6 | 6.567 | 1.129 | -1.549 |
| 1 | -2.946 | -5.827 | -1.794 |
| 1 | -1.411 | -5.013 | -1.613 |
| 1 | -7.541 | -0.671 | -1.526 |
| 1 | -1.443 | -2.674 | -1.513 |
| 1 | -5.827 | 2.946 | -1.794 |
| 1 | -5.013 | 1.411 | -1.613 |
| 1 | -0.671 | 7.541 | -1.526 |
| 1 | -2.674 | 1.443 | -1.513 |
| 1 | 5.827 | -2.946 | -1.794 |
| 1 | 5.013 | -1.411 | -1.613 |
| 1 | 0.671 | -7.541 | -1.526 |
| 1 | 2.674 | -1.443 | -1.513 |
| 1 | 2.946 | 5.827 | -1.794 |
| 1 | 1.411 | 5.013 | -1.613 |
| 1 | 7.541 | 0.671 | -1.526 |
| 1 | 1.443 | 2.674 | -1.513 |
| 1 | -3.185 | 7.126 | -1.734 |
| 1 | 7.126 | 3.185 | -1.734 |
| 1 | 3.185 | -7.126 | -1.734 |
| 1 | -7.126 | -3.185 | -1.733 |
| 19 | 0.000 | 0.000 | -0.002 |
| 8 | -1.392 | -1.950 | 1.379 |
| 8 | -1.950 | 1.392 | 1.379 |
| 8 | 1.950 | -1.392 | 1.379 |
| 8 | 1.392 | 1.950 | 1.379 |
| 7 | 1.807 | -5.231 | 1.705 |
| 7 | -0.372 | -5.940 | 1.719 |
| 7 | 0.144 | -3.630 | 1.554 |
| 7 | -3.484 | -4.193 | 1.483 |
| 7 | -2.763 | -6.290 | 1.646 |
| 7 | -5.231 | -1.807 | 1.705 |
| 7 | -5.940 | 0.372 | 1.719 |
| 7 | -3.630 | -0.144 | 1.554 |
| 7 | -4.193 | 3.484 | 1.483 |
| 7 | -6.290 | 2.763 | 1.646 |
| 7 | 5.231 | 1.807 | 1.705 |
| 7 | 5.940 | -0.372 | 1.719 |
| 7 | 3.630 | 0.144 | 1.554 |
| 7 | 4.193 | -3.484 | 1.483 |
| 7 | 6.290 | -2.763 | 1.646 |
| 7 | -1.807 | 5.231 | 1.705 |
| 7 | 0.372 | 5.940 | 1.719 |
| 7 | -0.144 | 3.630 | 1.554 |
| 7 | 3.484 | 4.193 | 1.483 |
| 7 | 2.763 | 6.290 | 1.646 |
| 6 | 0.500 | -4.951 | 1.659 |
| 6 | -1.162 | -3.155 | 1.485 |
| 6 | -2.107 | -4.215 | 1.543 |
| 6 | -1.637 | -5.514 | 1.647 |
| 6 | -3.840 | -5.442 | 1.544 |
| 6 | -4.951 | -0.500 | 1.660 |
| 6 | -3.155 | 1.162 | 1.485 |
| 6 | -4.215 | 2.107 | 1.543 |
| 6 | -5.514 | 1.637 | 1.647 |
| 6 | -5.442 | 3.840 | 1.544 |
| 6 | 4.951 | 0.500 | 1.660 |
| 6 | 3.155 | -1.162 | 1.485 |
| 6 | 4.215 | -2.107 | 1.543 |
| 6 | 5.514 | -1.637 | 1.647 |
| 6 | 5.442 | -3.840 | 1.544 |
| 6 | -0.500 | 4.951 | 1.659 |
| 6 | 1.162 | 3.155 | 1.485 |
| 6 | 2.107 | 4.215 | 1.543 |
| 6 | 1.637 | 5.514 | 1.647 |
| 6 | 3.840 | 5.442 | 1.544 |
| 1 | 2.041 | -6.201 | 1.799 |
| 1 | 2.552 | -4.541 | 1.617 |
| 1 | -4.853 | -5.808 | 1.519 |
| 1 | 0.874 | -2.909 | 1.516 |
| 1 | -6.201 | -2.041 | 1.800 |
| 1 | -4.541 | -2.552 | 1.618 |
| 1 | -5.808 | 4.853 | 1.518 |
| 1 | -2.909 | -0.874 | 1.516 |
| 1 | 6.201 | 2.041 | 1.799 |
| 1 | 4.541 | 2.552 | 1.618 |
| 1 | 5.808 | -4.853 | 1.519 |
| 1 | 2.909 | 0.874 | 1.516 |
| 1 | -2.041 | 6.201 | 1.799 |
| 1 | -2.552 | 4.541 | 1.617 |
| 1 | 4.853 | 5.808 | 1.519 |
| 1 | -0.874 | 2.909 | 1.516 |
| 1 | -7.291 | 2.781 | 1.732 |
| 1 | 2.781 | 7.291 | 1.732 |
| 1 | 7.291 | -2.781 | 1.732 |
| 1 | -2.781 | -7.291 | 1.732 |

Cartesian coordinates of optimized structures in the water phase (COSMO) by M05-2X/6-311G(d,p)// M05-2X/6-311G(d,p).

A-T

| 7 | -1.515 | 1.311 | 0.000 |
| --- | --- | --- | --- |
| 6 | -2.595 | 0.517 | 0.000 |
| 7 | -3.870 | 0.867 | 0.000 |
| 6 | -4.015 | 2.201 | 0.000 |
| 6 | -2.995 | 3.144 | 0.000 |
| 6 | -1.675 | 2.648 | 0.000 |
| 7 | -5.168 | 2.933 | 0.000 |
| 6 | -4.802 | 4.250 | 0.000 |
| 7 | -3.507 | 4.425 | 0.000 |
| 7 | -0.601 | 3.438 | 0.000 |
| 1 | -2.381 | -0.545 | 0.000 |
| 1 | -6.110 | 2.573 | 0.000 |
| 1 | -5.539 | 5.036 | 0.000 |
| 1 | -0.718 | 4.436 | 0.000 |
| 1 | 0.328 | 3.032 | 0.000 |
| 7 | 1.122 | 0.055 | 0.000 |
| 6 | 1.129 | -1.316 | 0.000 |
| 7 | 2.382 | -1.867 | 0.000 |
| 6 | 3.530 | -1.121 | 0.000 |
| 6 | 3.518 | 0.226 | 0.000 |
| 6 | 2.224 | 0.885 | 0.000 |
| 8 | 2.079 | 2.106 | 0.000 |
| 8 | 0.107 | -1.984 | 0.000 |
| 6 | 4.752 | 1.073 | 0.000 |
| 1 | 0.190 | 0.504 | 0.000 |
| 1 | 2.434 | -2.875 | 0.000 |
| 1 | 4.448 | -1.692 | 0.000 |
| 1 | 5.642 | 0.446 | 0.000 |
| 1 | 4.775 | 1.717 | 0.879 |
| 1 | 4.775 | 1.717 | -0.879 |

G-T

| 6 | -0.376 | 4.626 | 0.000 |
| --- | --- | --- | --- |
| 7 | 0.295 | 3.428 | 0.000 |
| 1 | 1.295 | 3.299 | 0.000 |
| 6 | -0.643 | 2.442 | 0.000 |
| 6 | -1.861 | 3.111 | 0.000 |
| 7 | -1.671 | 4.479 | 0.000 |
| 6 | -3.054 | 2.336 | 0.000 |
| 8 | -4.220 | 2.738 | 0.000 |
| 7 | -2.778 | 0.971 | 0.000 |
| 1 | -3.595 | 0.355 | 0.000 |
| 6 | -1.529 | 0.409 | 0.000 |
| 7 | -0.417 | 1.114 | 0.000 |
| 7 | -1.475 | -0.931 | 0.000 |
| 1 | 0.153 | 5.564 | 0.000 |
| 1 | -2.304 | -1.498 | 0.000 |
| 1 | -0.574 | -1.375 | 0.000 |
| 6 | -10.326 | 1.198 | 0.000 |
| 6 | -8.933 | 0.652 | 0.000 |
| 6 | -8.641 | -0.661 | 0.000 |
| 7 | -7.350 | -1.127 | 0.000 |
| 1 | -7.169 | -2.119 | 0.000 |
| 6 | -6.263 | -0.306 | 0.000 |
| 8 | -5.112 | -0.735 | 0.000 |
| 7 | -6.558 | 1.028 | 0.000 |
| 1 | -5.756 | 1.669 | 0.000 |
| 6 | -7.825 | 1.595 | 0.000 |
| 8 | -7.952 | 2.810 | 0.000 |
| 1 | -9.403 | -1.426 | 0.000 |
| 1 | -11.052 | 0.388 | 0.000 |
| 1 | -10.492 | 1.822 | -0.879 |
| 1 | -10.492 | 1.822 | 0.879 |

G-C

| 7 | -2.637 | 0.028 | 0.033 |
| --- | --- | --- | --- |
| 8 | 1.688 | 1.532 | 0.033 |
| 7 | -0.495 | 0.872 | 0.033 |
| 6 | -1.847 | 1.107 | 0.033 |
| 7 | -2.372 | 2.318 | 0.033 |
| 6 | -1.440 | 3.289 | 0.033 |
| 6 | -0.057 | 3.160 | 0.033 |
| 6 | 0.496 | 1.849 | 0.033 |
| 7 | -1.664 | 4.633 | 0.033 |
| 6 | -0.436 | 5.248 | 0.033 |
| 7 | 0.554 | 4.400 | 0.033 |
| 1 | -2.265 | -0.920 | 0.033 |
| 1 | -3.630 | 0.178 | 0.033 |
| 1 | -0.161 | -0.103 | 0.033 |
| 1 | -2.563 | 5.088 | 0.033 |
| 1 | -0.347 | 6.322 | 0.033 |
| 8 | -1.608 | -2.681 | 0.033 |
| 7 | 2.711 | -1.218 | 0.033 |
| 7 | 0.538 | -1.918 | 0.033 |
| 6 | 2.306 | -3.577 | 0.033 |
| 6 | -0.391 | -2.900 | 0.033 |
| 7 | 0.049 | -4.213 | 0.033 |
| 6 | 1.363 | -4.542 | 0.033 |
| 6 | 1.839 | -2.221 | 0.033 |
| 1 | 3.359 | -3.806 | 0.033 |
| 1 | -0.659 | -4.932 | 0.033 |
| 1 | 1.591 | -5.598 | 0.033 |
| 1 | 2.380 | -0.257 | 0.033 |
| 1 | 3.699 | -1.405 | 0.033 |

C-C

| 6 | 3.586 | 0.842 | 0.000 |
| --- | --- | --- | --- |
| 6 | 4.328 | 1.969 | 0.000 |
| 7 | 3.723 | 3.181 | 0.000 |
| 6 | 2.338 | 3.334 | 0.000 |
| 8 | 1.871 | 4.473 | 0.000 |
| 7 | 1.579 | 2.211 | 0.000 |
| 6 | 2.160 | 1.015 | 0.000 |
| 7 | 1.374 | -0.063 | 0.000 |
| 1 | 4.034 | -0.138 | 0.000 |
| 1 | 5.408 | 1.972 | 0.000 |
| 1 | 0.361 | 0.022 | 0.000 |
| 1 | 1.780 | -0.983 | 0.000 |
| 6 | -3.696 | -1.678 | 0.000 |
| 6 | -4.436 | -0.549 | 0.000 |
| 7 | -3.831 | 0.661 | 0.000 |
| 6 | -2.448 | 0.816 | 0.000 |
| 8 | -1.975 | 1.950 | 0.000 |
| 7 | -1.691 | -0.312 | 0.000 |
| 6 | -2.273 | -1.507 | 0.000 |
| 7 | -1.489 | -2.588 | 0.000 |
| 1 | -4.145 | -2.658 | 0.000 |
| 1 | -5.516 | -0.546 | 0.000 |
| 1 | -0.489 | -2.482 | 0.000 |
| 1 | -1.882 | -3.513 | 0.000 |
| 1 | 4.274 | 4.025 | 0.000 |
| 1 | -4.382 | 1.506 | 0.000 |

A-T/A-T

| 8 | 2.866 | -1.055 | 2.105 |
| --- | --- | --- | --- |
| 8 | 1.402 | 2.866 | 0.346 |
| 7 | 4.405 | 0.500 | 1.469 |
| 7 | 2.159 | 0.925 | 1.228 |
| 7 | -1.441 | 2.214 | 1.145 |
| 7 | -4.193 | 0.932 | 1.569 |
| 7 | -4.324 | -1.233 | 2.103 |
| 7 | -1.950 | -1.790 | 2.159 |
| 7 | -0.591 | 0.117 | 1.649 |
| 6 | 4.032 | 3.911 | -0.107 |
| 6 | 2.363 | 2.170 | 0.669 |
| 6 | 3.747 | 2.577 | 0.507 |
| 6 | 4.700 | 1.722 | 0.924 |
| 6 | 3.126 | 0.043 | 1.635 |
| 6 | -4.991 | -0.070 | 1.835 |
| 6 | -1.645 | 0.934 | 1.474 |
| 6 | -2.930 | 0.388 | 1.663 |
| 6 | -2.991 | -0.961 | 1.994 |
| 6 | -0.798 | -1.168 | 1.971 |
| 1 | 5.754 | 1.948 | 0.846 |
| 1 | 5.149 | -0.120 | 1.754 |
| 1 | 1.177 | 0.624 | 1.363 |
| 1 | -0.511 | 2.507 | 0.869 |
| 1 | -2.232 | 2.777 | 0.881 |
| 1 | -6.067 | -0.024 | 1.854 |
| 1 | -4.741 | -2.125 | 2.320 |
| 1 | 0.099 | -1.763 | 2.084 |
| 1 | 3.568 | 4.709 | 0.474 |
| 1 | 3.624 | 3.961 | -1.117 |
| 1 | 5.105 | 4.086 | -0.152 |
| 8 | 0.725 | -3.560 | 0.048 |
| 8 | 2.122 | 0.210 | -2.067 |
| 7 | 2.955 | -3.261 | -0.295 |
| 7 | 1.450 | -1.673 | -1.004 |
| 7 | -0.637 | 1.292 | -1.984 |
| 7 | -3.616 | 2.020 | -1.961 |
| 7 | -5.130 | 0.415 | -1.615 |
| 7 | -3.644 | -1.490 | -1.276 |
| 7 | -1.342 | -0.857 | -1.490 |
| 6 | 4.897 | -0.488 | -1.965 |
| 6 | 2.428 | -0.854 | -1.529 |
| 6 | 3.794 | -1.325 | -1.396 |
| 6 | 3.988 | -2.508 | -0.781 |
| 6 | 1.644 | -2.882 | -0.383 |
| 6 | -4.889 | 1.737 | -1.864 |
| 6 | -1.629 | 0.433 | -1.749 |
| 6 | -2.987 | 0.809 | -1.757 |
| 6 | -3.914 | -0.201 | -1.530 |
| 6 | -2.341 | -1.721 | -1.265 |
| 1 | 4.972 | -2.931 | -0.639 |
| 1 | 3.142 | -4.139 | 0.167 |
| 1 | 0.467 | -1.383 | -1.134 |
| 1 | 0.326 | 0.973 | -1.992 |
| 1 | -0.848 | 2.261 | -2.148 |
| 1 | -5.696 | 2.444 | -1.965 |
| 1 | -6.032 | -0.015 | -1.484 |
| 1 | -2.031 | -2.736 | -1.049 |
| 1 | 4.856 | 0.525 | -1.563 |
| 1 | 4.803 | -0.413 | -3.049 |
| 1 | 5.866 | -0.923 | -1.726 |

G-C/A-T

| 8 | -3.171 | 1.194 | 2.040 |
| --- | --- | --- | --- |
| 8 | -1.737 | -2.783 | 0.385 |
| 7 | -4.718 | -0.293 | 1.275 |
| 7 | -2.481 | -0.818 | 1.224 |
| 7 | 1.084 | -2.215 | 1.298 |
| 7 | 3.865 | -0.996 | 1.749 |
| 7 | 4.042 | 1.173 | 2.251 |
| 7 | 1.683 | 1.796 | 2.236 |
| 7 | 0.286 | -0.080 | 1.717 |
| 6 | -4.351 | -3.667 | -0.382 |
| 6 | -2.689 | -2.042 | 0.624 |
| 6 | -4.067 | -2.369 | 0.307 |
| 6 | -5.015 | -1.479 | 0.655 |
| 6 | -3.439 | 0.108 | 1.548 |
| 6 | 4.682 | -0.013 | 2.020 |
| 6 | 1.320 | -0.931 | 1.592 |
| 6 | 2.615 | -0.416 | 1.798 |
| 6 | 2.705 | 0.936 | 2.109 |
| 6 | 0.520 | 1.204 | 2.022 |
| 1 | -6.066 | -1.647 | 0.465 |
| 1 | -5.454 | 0.363 | 1.489 |
| 1 | -1.500 | -0.552 | 1.419 |
| 1 | 0.155 | -2.476 | 0.983 |
| 1 | 1.863 | -2.798 | 1.041 |
| 1 | 5.756 | -0.088 | 2.069 |
| 1 | 4.477 | 2.057 | 2.466 |
| 1 | -0.363 | 1.826 | 2.097 |
| 1 | -3.995 | -4.506 | 0.217 |
| 1 | -3.836 | -3.708 | -1.343 |
| 1 | -5.420 | -3.783 | -0.551 |
| 7 | 1.964 | 2.787 | -1.059 |
| 8 | 0.488 | -1.477 | -1.843 |
| 7 | 1.315 | 0.605 | -1.421 |
| 6 | 2.330 | 1.503 | -1.221 |
| 7 | 3.605 | 1.168 | -1.222 |
| 6 | 3.796 | -0.150 | -1.424 |
| 6 | 2.843 | -1.139 | -1.625 |
| 6 | 1.473 | -0.758 | -1.650 |
| 7 | 4.990 | -0.804 | -1.460 |
| 6 | 4.716 | -2.134 | -1.657 |
| 7 | 3.440 | -2.377 | -1.766 |
| 1 | 1.003 | 3.007 | -0.801 |
| 1 | 2.686 | 3.411 | -0.741 |
| 1 | 0.339 | 0.938 | -1.380 |
| 1 | 5.901 | -0.390 | -1.333 |
| 1 | 5.505 | -2.866 | -1.715 |
| 8 | -0.800 | 3.416 | -0.320 |
| 7 | -2.253 | -0.493 | -2.165 |
| 7 | -1.497 | 1.453 | -1.239 |
| 6 | -3.869 | 1.202 | -1.666 |
| 6 | -1.710 | 2.693 | -0.745 |
| 7 | -3.009 | 3.169 | -0.721 |
| 6 | -4.061 | 2.441 | -1.167 |
| 6 | -2.519 | 0.720 | -1.688 |
| 1 | -4.685 | 0.597 | -2.025 |
| 1 | -3.151 | 4.094 | -0.342 |
| 1 | -5.031 | 2.909 | -1.094 |
| 1 | -1.307 | -0.862 | -2.106 |
| 1 | -3.001 | -1.087 | -2.479 |

G-C/T-A

| 8 | -2.383 | -0.821 | -2.506 |
| --- | --- | --- | --- |
| 8 | -1.055 | 2.889 | -0.254 |
| 7 | -3.976 | 0.604 | -1.716 |
| 7 | -1.750 | 1.044 | -1.360 |
| 7 | 1.807 | 2.315 | -0.944 |
| 7 | 4.612 | 1.096 | -1.220 |
| 7 | 4.818 | -1.042 | -1.834 |
| 7 | 2.467 | -1.623 | -2.116 |
| 7 | 1.039 | 0.234 | -1.609 |
| 6 | -3.749 | 3.739 | 0.375 |
| 6 | -1.997 | 2.206 | -0.655 |
| 6 | -3.395 | 2.539 | -0.451 |
| 6 | -4.314 | 1.720 | -1.000 |
| 6 | -2.684 | 0.198 | -1.902 |
| 6 | 5.443 | 0.119 | -1.469 |
| 6 | 2.062 | 1.057 | -1.314 |
| 6 | 3.367 | 0.539 | -1.429 |
| 6 | 3.476 | -0.792 | -1.819 |
| 6 | 1.293 | -1.027 | -1.985 |
| 1 | -5.375 | 1.899 | -0.903 |
| 1 | -4.697 | 0.007 | -2.095 |
| 1 | -0.761 | 0.769 | -1.480 |
| 1 | 0.851 | 2.587 | -0.736 |
| 1 | 2.566 | 2.893 | -0.624 |
| 1 | 6.517 | 0.181 | -1.408 |
| 1 | 5.265 | -1.913 | -2.075 |
| 1 | 0.417 | -1.625 | -2.207 |
| 1 | -2.956 | 4.483 | 0.306 |
| 1 | -3.861 | 3.466 | 1.428 |
| 1 | -4.683 | 4.182 | 0.031 |
| 7 | 0.242 | -3.134 | 0.342 |
| 8 | -1.715 | 0.624 | 2.079 |
| 7 | -0.825 | -1.269 | 1.172 |
| 6 | -0.912 | -2.490 | 0.553 |
| 7 | -2.054 | -3.029 | 0.169 |
| 6 | -3.117 | -2.263 | 0.474 |
| 6 | -3.143 | -1.039 | 1.130 |
| 6 | -1.902 | -0.458 | 1.517 |
| 7 | -4.424 | -2.545 | 0.212 |
| 6 | -5.171 | -1.511 | 0.720 |
| 7 | -4.441 | -0.589 | 1.282 |
| 1 | 1.134 | -2.781 | 0.681 |
| 1 | 0.194 | -4.044 | -0.083 |
| 1 | 0.110 | -0.909 | 1.416 |
| 1 | -4.773 | -3.365 | -0.260 |
| 1 | -6.246 | -1.504 | 0.639 |
| 8 | 2.819 | -2.170 | 1.254 |
| 7 | 0.978 | 1.854 | 2.356 |
| 7 | 1.867 | -0.176 | 1.803 |
| 6 | 3.361 | 1.683 | 2.247 |
| 6 | 2.929 | -0.979 | 1.571 |
| 7 | 4.196 | -0.438 | 1.694 |
| 6 | 4.406 | 0.862 | 2.016 |
| 6 | 2.053 | 1.107 | 2.131 |
| 1 | 3.494 | 2.722 | 2.505 |
| 1 | 4.976 | -1.052 | 1.511 |
| 1 | 5.436 | 1.180 | 2.069 |
| 1 | 0.048 | 1.454 | 2.262 |
| 1 | 1.078 | 2.830 | 2.578 |

G-C/G-C

| 7 | -0.244 | -0.982 | -2.182 |
| --- | --- | --- | --- |
| 8 | 1.771 | 2.737 | -0.436 |
| 7 | 0.856 | 0.862 | -1.352 |
| 6 | 0.925 | -0.386 | -1.918 |
| 7 | 2.062 | -1.000 | -2.183 |
| 6 | 3.140 | -0.272 | -1.832 |
| 6 | 3.179 | 0.995 | -1.264 |
| 6 | 1.944 | 1.638 | -0.969 |
| 7 | 4.445 | -0.637 | -1.958 |
| 6 | 5.205 | 0.400 | -1.474 |
| 7 | 4.483 | 1.398 | -1.048 |
| 1 | -1.132 | -0.492 | -2.097 |
| 1 | -0.214 | -1.883 | -2.628 |
| 1 | -0.075 | 1.259 | -1.155 |
| 1 | 4.786 | -1.507 | -2.336 |
| 1 | 6.281 | 0.350 | -1.468 |
| 8 | -2.810 | 0.355 | -1.938 |
| 7 | -0.859 | 3.692 | 0.484 |
| 7 | -1.802 | 2.016 | -0.748 |
| 6 | -3.245 | 3.541 | 0.466 |
| 6 | -2.885 | 1.349 | -1.205 |
| 7 | -4.138 | 1.806 | -0.833 |
| 6 | -4.313 | 2.871 | -0.014 |
| 6 | -1.954 | 3.071 | 0.059 |
| 1 | -3.350 | 4.392 | 1.119 |
| 1 | -4.933 | 1.292 | -1.183 |
| 1 | -5.335 | 3.133 | 0.217 |
| 1 | 0.059 | 3.365 | 0.195 |
| 1 | -0.933 | 4.484 | 1.099 |
| 7 | 0.934 | -3.391 | 0.257 |
| 8 | -0.047 | 0.691 | 2.087 |
| 7 | 0.535 | -1.323 | 1.188 |
| 6 | 1.441 | -2.277 | 0.802 |
| 7 | 2.744 | -2.147 | 0.963 |
| 6 | 3.083 | -0.972 | 1.525 |
| 6 | 2.250 | 0.055 | 1.952 |
| 6 | 0.848 | -0.104 | 1.781 |
| 7 | 4.343 | -0.530 | 1.792 |
| 6 | 4.225 | 0.721 | 2.345 |
| 7 | 2.986 | 1.107 | 2.462 |
| 1 | -0.044 | -3.445 | -0.021 |
| 1 | 1.591 | -4.043 | -0.137 |
| 1 | -0.468 | -1.489 | 1.015 |
| 1 | 5.202 | -1.020 | 1.592 |
| 1 | 5.093 | 1.288 | 2.637 |
| 8 | -1.859 | -3.558 | -0.542 |
| 7 | -2.924 | 0.156 | 1.883 |
| 7 | -2.360 | -1.712 | 0.696 |
| 6 | -4.691 | -1.060 | 0.822 |
| 6 | -2.688 | -2.753 | -0.102 |
| 7 | -4.021 | -2.921 | -0.436 |
| 6 | -4.998 | -2.100 | 0.020 |
| 6 | -3.304 | -0.877 | 1.135 |
| 1 | -5.443 | -0.383 | 1.194 |
| 1 | -4.248 | -3.703 | -1.032 |
| 1 | -6.004 | -2.332 | -0.297 |
| 1 | -1.934 | 0.332 | 2.034 |
| 1 | -3.605 | 0.815 | 2.219 |

C^+^•G-C

| 7 | -2.741 | -0.050 | 0.000 |
| --- | --- | --- | --- |
| 8 | 1.612 | 1.365 | 0.000 |
| 7 | -0.580 | 0.746 | 0.000 |
| 6 | -1.929 | 1.009 | 0.000 |
| 7 | -2.429 | 2.232 | 0.000 |
| 6 | -1.483 | 3.184 | 0.000 |
| 6 | -0.105 | 3.022 | 0.000 |
| 6 | 0.418 | 1.704 | 0.000 |
| 7 | -1.677 | 4.534 | 0.000 |
| 6 | -0.444 | 5.126 | 0.000 |
| 7 | 0.525 | 4.251 | 0.000 |
| 1 | -2.391 | -1.006 | 0.000 |
| 1 | -3.731 | 0.124 | 0.000 |
| 1 | -0.264 | -0.238 | 0.000 |
| 1 | -2.567 | 5.009 | 0.000 |
| 1 | -0.321 | 6.195 | 0.000 |
| 8 | -1.785 | -2.767 | 0.000 |
| 7 | 2.575 | -1.425 | 0.000 |
| 7 | 0.381 | -2.061 | 0.000 |
| 6 | 2.102 | -3.769 | 0.000 |
| 6 | -0.574 | -3.018 | 0.000 |
| 7 | -0.171 | -4.342 | 0.000 |
| 6 | 1.134 | -4.708 | 0.000 |
| 6 | 1.673 | -2.401 | 0.000 |
| 1 | 3.149 | -4.027 | 0.000 |
| 1 | -0.899 | -5.041 | 0.000 |
| 1 | 1.333 | -5.769 | 0.000 |
| 1 | 2.273 | -0.456 | 0.000 |
| 1 | 3.557 | -1.643 | 0.000 |
| 6 | 5.607 | 4.677 | 0.000 |
| 6 | 5.743 | 6.020 | 0.000 |
| 7 | 4.665 | 6.844 | 0.000 |
| 6 | 3.368 | 6.390 | 0.000 |
| 8 | 2.404 | 7.125 | 0.000 |
| 7 | 3.248 | 5.015 | 0.000 |
| 1 | 2.261 | 4.668 | 0.000 |
| 6 | 4.284 | 4.146 | 0.000 |
| 7 | 4.043 | 2.857 | 0.000 |
| 1 | 6.458 | 4.017 | 0.000 |
| 1 | 6.705 | 6.509 | 0.000 |
| 1 | 3.098 | 2.462 | 0.000 |
| 1 | 4.822 | 2.218 | 0.000 |
| 1 | 4.792 | 7.847 | 0.000 |

T•A-T

|  |  |  |  |
| --- | --- | --- | --- |
| 8 | -2.281 | -1.826 | 0.151 |
| 8 | -5.586 | 1.265 | -0.197 |
| 7 | -3.945 | -0.304 | -0.028 |
| 7 | -6.147 | -0.941 | -0.199 |
| 6 | -3.481 | -1.602 | 0.042 |
| 6 | -5.252 | 0.095 | -0.139 |
| 6 | -5.778 | -2.259 | -0.137 |
| 6 | -4.494 | -2.645 | -0.019 |
| 6 | -4.049 | -4.072 | 0.048 |
| 1 | -3.369 | -4.302 | -0.773 |
| 1 | -3.516 | -4.264 | 0.979 |
| 1 | -4.908 | -4.739 | -0.010 |
| 1 | -6.592 | -2.967 | -0.190 |
| 1 | -3.247 | 0.457 | 0.034 |
| 1 | -7.120 | -0.690 | -0.289 |
| 8 | 2.709 | -1.835 | 0.223 |
| 8 | 5.289 | 1.872 | -0.229 |
| 7 | 1.621 | 1.657 | 0.079 |
| 7 | 0.881 | 3.938 | 0.042 |
| 7 | -1.550 | 4.091 | 0.081 |
| 7 | -2.070 | 1.932 | 0.135 |
| 7 | 0.204 | -0.171 | 0.118 |
| 7 | 4.020 | -0.008 | -0.016 |
| 7 | 6.302 | -0.164 | -0.238 |
| 6 | 1.800 | 2.985 | 0.047 |
| 6 | -0.356 | 3.423 | 0.070 |
| 6 | -0.696 | 2.076 | 0.100 |
| 6 | 0.370 | 1.149 | 0.100 |
| 6 | -2.537 | 3.153 | 0.111 |
| 6 | 5.210 | 0.658 | -0.160 |
| 6 | 6.217 | -1.527 | -0.168 |
| 6 | 5.047 | -2.179 | -0.018 |
| 6 | 3.841 | -1.374 | 0.071 |
| 6 | 4.949 | -3.674 | 0.085 |
| 1 | 5.641 | -4.153 | -0.604 |
| 1 | 5.178 | -4.013 | 1.096 |
| 1 | 3.937 | -3.996 | -0.151 |
| 1 | 2.835 | 3.307 | 0.036 |
| 1 | -1.679 | 5.092 | 0.068 |
| 1 | -3.579 | 3.422 | 0.121 |
| 1 | -0.719 | -0.593 | 0.146 |
| 1 | 1.022 | -0.770 | 0.144 |
| 1 | 3.170 | 0.581 | 0.035 |
| 1 | 7.201 | 0.283 | -0.350 |
| 1 | 7.161 | -2.048 | -0.247 |

rT•A-T

| 8 | 0.214 | -3.419 | 0.000 |
| --- | --- | --- | --- |
| 8 | 2.115 | 0.699 | 0.000 |
| 7 | 2.487 | -3.271 | 0.000 |
| 7 | 1.204 | -1.368 | 0.000 |
| 7 | -0.598 | 2.007 | 0.000 |
| 7 | -3.556 | 2.888 | 0.000 |
| 7 | -5.154 | 1.340 | 0.000 |
| 7 | -3.781 | -0.675 | 0.000 |
| 7 | -1.445 | -0.146 | 0.000 |
| 6 | 4.841 | -0.316 | 0.000 |
| 6 | 2.293 | -0.518 | 0.000 |
| 6 | 3.597 | -1.160 | 0.000 |
| 6 | 3.622 | -2.507 | 0.000 |
| 6 | 1.227 | -2.737 | 0.000 |
| 6 | -4.846 | 2.668 | 0.000 |
| 6 | -1.646 | 1.187 | 0.000 |
| 6 | -2.987 | 1.631 | 0.000 |
| 6 | -3.972 | 0.652 | 0.000 |
| 6 | -2.494 | -0.978 | 0.000 |
| 1 | 4.547 | -3.067 | 0.000 |
| 1 | 2.555 | -4.278 | 0.000 |
| 1 | 0.267 | -0.930 | 0.000 |
| 1 | 0.335 | 1.609 | 0.000 |
| 1 | -0.715 | 3.013 | 0.000 |
| 1 | -5.607 | 3.431 | 0.000 |
| 1 | -6.080 | 0.942 | 0.000 |
| 1 | -2.241 | -2.032 | 0.000 |
| 1 | 4.564 | 0.735 | 0.000 |
| 1 | 5.448 | -0.511 | -0.883 |
| 1 | 5.448 | -0.511 | 0.883 |
| 6 | -4.201 | 9.061 | 0.000 |
| 6 | -3.163 | 7.982 | 0.000 |
| 6 | -1.838 | 8.216 | 0.000 |
| 7 | -0.912 | 7.203 | 0.000 |
| 1 | 0.073 | 7.416 | 0.000 |
| 6 | -1.254 | 5.881 | 0.000 |
| 8 | -0.422 | 4.981 | 0.000 |
| 7 | -2.601 | 5.646 | 0.000 |
| 1 | -2.892 | 4.656 | 0.000 |
| 6 | -3.607 | 6.597 | 0.000 |
| 8 | -4.781 | 6.248 | 0.000 |
| 1 | -1.424 | 9.214 | 0.000 |
| 1 | -3.729 | 10.042 | 0.000 |
| 1 | -4.840 | 8.977 | 0.879 |
| 1 | -4.840 | 8.977 | -0.879 |

rA•A-T

| 8 | 0.199 | -3.352 | 0.000 |
| --- | --- | --- | --- |
| 8 | 2.027 | 0.800 | 0.000 |
| 7 | 2.469 | -3.163 | 0.000 |
| 7 | 1.151 | -1.282 | 0.000 |
| 7 | -0.702 | 2.041 | 0.000 |
| 7 | -3.655 | 2.916 | 0.000 |
| 7 | -5.250 | 1.360 | 0.000 |
| 7 | -3.872 | -0.651 | 0.000 |
| 7 | -1.537 | -0.117 | 0.000 |
| 6 | 4.770 | -0.167 | 0.000 |
| 6 | 2.225 | -0.414 | 0.000 |
| 6 | 3.541 | -1.033 | 0.000 |
| 6 | 3.590 | -2.380 | 0.000 |
| 6 | 1.199 | -2.652 | 0.000 |
| 6 | -4.944 | 2.689 | 0.000 |
| 6 | -1.744 | 1.216 | 0.000 |
| 6 | -3.085 | 1.658 | 0.000 |
| 6 | -4.067 | 0.676 | 0.000 |
| 6 | -2.584 | -0.951 | 0.000 |
| 1 | 4.525 | -2.922 | 0.000 |
| 1 | 2.552 | -4.169 | 0.000 |
| 1 | 0.205 | -0.864 | 0.000 |
| 1 | 0.235 | 1.651 | 0.000 |
| 1 | -0.830 | 3.048 | 0.000 |
| 1 | -5.711 | 3.446 | 0.000 |
| 1 | -6.176 | 0.960 | 0.000 |
| 1 | -2.331 | -2.004 | 0.000 |
| 7 | -2.562 | 8.108 | 0.000 |
| 6 | -2.356 | 6.779 | 0.000 |
| 6 | -1.022 | 6.319 | 0.000 |
| 6 | -0.031 | 7.291 | 0.000 |
| 7 | -0.215 | 8.618 | 0.000 |
| 6 | -1.505 | 8.927 | 0.000 |
| 7 | 1.147 | 6.594 | 0.000 |
| 6 | 0.826 | 5.269 | 0.000 |
| 7 | -0.465 | 5.056 | 0.000 |
| 7 | -3.405 | 5.953 | 0.000 |
| 1 | -1.740 | 9.984 | 0.000 |
| 1 | 1.585 | 4.503 | 0.000 |
| 1 | -4.329 | 6.350 | 0.000 |
| 1 | -3.295 | 4.944 | 0.000 |
| 1 | 2.076 | 6.985 | 0.000 |
| 1 | 4.475 | 0.879 | 0.000 |
| 1 | 5.380 | -0.352 | -0.883 |
| 1 | 5.380 | -0.352 | 0.883 |

rG•G-C

| 7 | -2.638 | -0.002 | 0.000 |
| --- | --- | --- | --- |
| 8 | 1.695 | 1.441 | 0.000 |
| 7 | -0.489 | 0.814 | 0.000 |
| 6 | -1.836 | 1.064 | 0.000 |
| 7 | -2.347 | 2.285 | 0.000 |
| 6 | -1.407 | 3.242 | 0.000 |
| 6 | -0.026 | 3.091 | 0.000 |
| 6 | 0.499 | 1.779 | 0.000 |
| 7 | -1.610 | 4.590 | 0.000 |
| 6 | -0.380 | 5.189 | 0.000 |
| 7 | 0.596 | 4.326 | 0.000 |
| 1 | -2.275 | -0.955 | 0.000 |
| 1 | -3.629 | 0.162 | 0.000 |
| 1 | -0.164 | -0.173 | 0.000 |
| 1 | -2.504 | 5.055 | 0.000 |
| 1 | -0.268 | 6.260 | 0.000 |
| 8 | -1.665 | -2.705 | 0.000 |
| 7 | 2.632 | -1.182 | 0.000 |
| 7 | 0.469 | -1.914 | 0.000 |
| 6 | 2.260 | -3.545 | 0.000 |
| 6 | -0.446 | -2.905 | 0.000 |
| 7 | 0.012 | -4.213 | 0.000 |
| 6 | 1.329 | -4.522 | 0.000 |
| 6 | 1.778 | -2.196 | 0.000 |
| 1 | 3.314 | -3.763 | 0.000 |
| 1 | -0.688 | -4.940 | 0.000 |
| 1 | 1.573 | -5.573 | 0.000 |
| 1 | 2.288 | -0.214 | 0.000 |
| 1 | 3.621 | -1.364 | 0.000 |
| 6 | 6.795 | 7.811 | 0.000 |
| 7 | 7.102 | 6.471 | 0.000 |
| 1 | 8.030 | 6.074 | 0.000 |
| 6 | 5.928 | 5.783 | 0.000 |
| 6 | 4.944 | 6.761 | 0.000 |
| 7 | 5.507 | 8.021 | 0.000 |
| 6 | 3.575 | 6.355 | 0.000 |
| 8 | 2.574 | 7.077 | 0.000 |
| 7 | 3.460 | 4.971 | 0.000 |
| 1 | 2.493 | 4.610 | 0.000 |
| 6 | 4.509 | 4.083 | 0.000 |
| 7 | 5.774 | 4.446 | 0.000 |
| 7 | 4.181 | 2.786 | 0.000 |
| 1 | 7.564 | 8.566 | 0.000 |
| 1 | 3.219 | 2.447 | 0.000 |
| 1 | 4.940 | 2.126 | 0.000 |

C^+^•G-C/C+•G-C

| 7 | -0.981 | -3.081 | 2.165 |
| --- | --- | --- | --- |
| 8 | 0.630 | 1.129 | 1.378 |
| 7 | -0.087 | -0.997 | 1.776 |
| 6 | 0.115 | -2.335 | 2.014 |
| 7 | 1.314 | -2.884 | 2.108 |
| 6 | 2.305 | -2.004 | 1.903 |
| 6 | 2.203 | -0.646 | 1.635 |
| 6 | 0.912 | -0.063 | 1.574 |
| 7 | 3.646 | -2.260 | 1.907 |
| 6 | 4.290 | -1.084 | 1.641 |
| 7 | 3.456 | -0.092 | 1.476 |
| 1 | -1.913 | -2.700 | 2.007 |
| 1 | -0.856 | -4.071 | 2.284 |
| 1 | -1.053 | -0.630 | 1.759 |
| 1 | 4.081 | -3.158 | 2.058 |
| 1 | 5.363 | -1.014 | 1.580 |
| 8 | -3.607 | -1.970 | 1.711 |
| 7 | -2.044 | 2.306 | 1.928 |
| 7 | -2.796 | 0.155 | 1.808 |
| 6 | -4.413 | 1.955 | 1.968 |
| 6 | -3.800 | -0.750 | 1.773 |
| 7 | -5.101 | -0.281 | 1.808 |
| 6 | -5.400 | 1.037 | 1.911 |
| 6 | -3.070 | 1.459 | 1.897 |
| 1 | -4.616 | 3.011 | 2.044 |
| 1 | -5.836 | -0.972 | 1.765 |
| 1 | -6.450 | 1.288 | 1.935 |
| 1 | -1.103 | 1.952 | 1.788 |
| 1 | -2.207 | 3.298 | 1.936 |
| 6 | 4.085 | 4.621 | -0.310 |
| 6 | 5.429 | 4.645 | -0.442 |
| 7 | 6.198 | 3.583 | -0.092 |
| 6 | 5.681 | 2.416 | 0.417 |
| 8 | 6.365 | 1.461 | 0.722 |
| 7 | 4.308 | 2.411 | 0.549 |
| 1 | 3.919 | 1.511 | 0.922 |
| 6 | 3.493 | 3.436 | 0.214 |
| 7 | 2.197 | 3.308 | 0.379 |
| 1 | 3.470 | 5.461 | -0.586 |
| 1 | 5.962 | 5.501 | -0.829 |
| 1 | 1.761 | 2.461 | 0.755 |
| 1 | 1.596 | 4.076 | 0.123 |
| 1 | 7.202 | 3.623 | -0.199 |
| 7 | -4.960 | -1.896 | -1.414 |
| 8 | -0.773 | -0.049 | -1.399 |
| 7 | -2.820 | -1.051 | -1.397 |
| 6 | -3.650 | -2.144 | -1.342 |
| 7 | -3.208 | -3.386 | -1.247 |
| 6 | -1.870 | -3.458 | -1.177 |
| 6 | -0.953 | -2.418 | -1.220 |
| 6 | -1.440 | -1.096 | -1.342 |
| 7 | -1.112 | -4.585 | -1.041 |
| 6 | 0.199 | -4.197 | -1.005 |
| 7 | 0.335 | -2.903 | -1.114 |
| 1 | -5.328 | -0.947 | -1.395 |
| 1 | -5.584 | -2.677 | -1.306 |
| 1 | -3.235 | -0.107 | -1.442 |
| 1 | -1.457 | -5.531 | -0.970 |
| 1 | 1.004 | -4.904 | -0.900 |
| 8 | -6.001 | 0.797 | -1.281 |
| 7 | -1.844 | 2.672 | -1.436 |
| 7 | -3.911 | 1.700 | -1.368 |
| 6 | -3.729 | 4.101 | -1.076 |
| 6 | -5.253 | 1.783 | -1.240 |
| 7 | -5.816 | 3.032 | -1.046 |
| 6 | -5.072 | 4.162 | -0.963 |
| 6 | -3.159 | 2.803 | -1.293 |
| 1 | -3.108 | 4.980 | -1.014 |
| 1 | -6.820 | 3.072 | -0.959 |
| 1 | -5.615 | 5.082 | -0.802 |
| 1 | -1.437 | 1.741 | -1.483 |
| 1 | -1.243 | 3.469 | -1.318 |
| 6 | 4.384 | 0.093 | -1.910 |
| 6 | 5.386 | -0.803 | -1.793 |
| 7 | 5.144 | -2.100 | -1.468 |
| 6 | 3.881 | -2.604 | -1.266 |
| 8 | 3.661 | -3.769 | -1.007 |
| 7 | 2.872 | -1.670 | -1.373 |
| 1 | 1.908 | -2.058 | -1.241 |
| 6 | 3.055 | -0.364 | -1.674 |
| 7 | 2.014 | 0.429 | -1.738 |
| 1 | 4.565 | 1.124 | -2.170 |
| 1 | 6.423 | -0.543 | -1.942 |
| 1 | 1.054 | 0.109 | -1.581 |
| 1 | 2.155 | 1.401 | -1.967 |
| 1 | 5.908 | -2.758 | -1.395 |

C^+^•G-C/T•A-T

| 8 | -3.935 | -1.842 | 2.440 |
| --- | --- | --- | --- |
| 8 | -2.260 | 2.216 | 1.295 |
| 7 | -5.367 | -0.077 | 2.279 |
| 7 | -3.130 | 0.200 | 1.838 |
| 7 | 0.530 | 1.071 | 1.242 |
| 7 | 3.188 | -0.485 | 1.443 |
| 7 | 3.146 | -2.662 | 1.898 |
| 7 | 0.741 | -2.995 | 2.117 |
| 7 | -0.463 | -0.954 | 1.761 |
| 6 | -4.785 | 3.567 | 1.476 |
| 6 | -3.252 | 1.552 | 1.591 |
| 6 | -4.590 | 2.102 | 1.711 |
| 6 | -5.583 | 1.255 | 2.042 |
| 6 | -4.133 | -0.660 | 2.202 |
| 6 | 3.907 | -1.561 | 1.638 |
| 6 | 0.644 | -0.227 | 1.516 |
| 6 | 1.881 | -0.905 | 1.587 |
| 6 | 1.838 | -2.262 | 1.881 |
| 6 | -0.356 | -2.261 | 2.036 |
| 1 | -6.609 | 1.578 | 2.144 |
| 1 | -6.138 | -0.677 | 2.531 |
| 1 | -2.181 | -0.208 | 1.781 |
| 1 | -0.397 | 1.480 | 1.189 |
| 1 | 1.332 | 1.616 | 0.950 |
| 1 | 4.983 | -1.605 | 1.604 |
| 1 | 3.489 | -3.593 | 2.082 |
| 1 | -1.298 | -2.768 | 2.215 |
| 1 | -4.250 | 4.151 | 2.227 |
| 1 | -5.843 | 3.821 | 1.523 |
| 1 | -4.395 | 3.854 | 0.500 |
| 6 | 3.732 | 5.476 | -0.606 |
| 6 | 4.474 | 4.309 | -0.034 |
| 6 | 5.800 | 4.303 | 0.204 |
| 7 | 6.451 | 3.214 | 0.719 |
| 1 | 7.449 | 3.244 | 0.865 |
| 6 | 5.818 | 2.045 | 1.040 |
| 8 | 6.401 | 1.063 | 1.471 |
| 7 | 4.462 | 2.070 | 0.829 |
| 1 | 3.961 | 1.195 | 1.048 |
| 6 | 3.734 | 3.103 | 0.281 |
| 8 | 2.526 | 2.964 | 0.077 |
| 1 | 6.429 | 5.157 | -0.004 |
| 1 | 4.413 | 6.305 | -0.789 |
| 1 | 2.950 | 5.806 | 0.078 |
| 1 | 3.252 | 5.201 | -1.546 |
| 7 | -4.680 | -2.393 | -0.721 |
| 8 | -0.649 | -0.408 | -1.601 |
| 7 | -2.609 | -1.467 | -1.124 |
| 6 | -3.357 | -2.570 | -0.787 |
| 7 | -2.831 | -3.761 | -0.561 |
| 6 | -1.499 | -3.784 | -0.732 |
| 6 | -0.665 | -2.740 | -1.103 |
| 6 | -1.238 | -1.459 | -1.303 |
| 7 | -0.668 | -4.855 | -0.575 |
| 6 | 0.603 | -4.432 | -0.848 |
| 7 | 0.644 | -3.168 | -1.173 |
| 1 | -5.104 | -1.468 | -0.793 |
| 1 | -5.227 | -3.163 | -0.378 |
| 1 | -3.087 | -0.561 | -1.260 |
| 1 | -0.941 | -5.788 | -0.305 |
| 1 | 1.453 | -5.090 | -0.793 |
| 8 | -5.927 | 0.187 | -0.985 |
| 7 | -1.986 | 2.153 | -2.171 |
| 7 | -3.940 | 1.141 | -1.561 |
| 6 | -3.998 | 3.436 | -2.342 |
| 6 | -5.280 | 1.162 | -1.385 |
| 7 | -5.957 | 2.336 | -1.671 |
| 6 | -5.333 | 3.440 | -2.150 |
| 6 | -3.304 | 2.224 | -2.017 |
| 1 | -3.471 | 4.298 | -2.717 |
| 1 | -6.956 | 2.331 | -1.534 |
| 1 | -5.962 | 4.294 | -2.352 |
| 1 | -1.496 | 1.292 | -1.951 |
| 1 | -1.472 | 2.946 | -2.514 |
| 6 | 4.520 | 0.068 | -1.746 |
| 6 | 5.557 | -0.794 | -1.693 |
| 7 | 5.362 | -2.131 | -1.546 |
| 6 | 4.116 | -2.706 | -1.467 |
| 8 | 3.942 | -3.901 | -1.344 |
| 7 | 3.069 | -1.810 | -1.528 |
| 1 | 2.122 | -2.252 | -1.428 |
| 6 | 3.204 | -0.468 | -1.635 |
| 7 | 2.132 | 0.286 | -1.637 |
| 1 | 4.667 | 1.127 | -1.873 |
| 1 | 6.586 | -0.476 | -1.760 |
| 1 | 1.185 | -0.097 | -1.574 |
| 1 | 2.239 | 1.290 | -1.616 |
| 1 | 6.151 | -2.760 | -1.504 |

T•A-T/T•A-T

| 8 | 4.861 | -3.058 | -0.346 |
| --- | --- | --- | --- |
| 8 | 3.454 | 0.962 | -1.925 |
| 7 | 6.381 | -1.377 | -0.567 |
| 7 | 4.180 | -1.033 | -1.137 |
| 7 | 0.626 | 0.036 | -1.685 |
| 7 | -2.131 | -1.346 | -1.689 |
| 7 | -2.236 | -3.568 | -1.691 |
| 7 | 0.145 | -4.096 | -1.646 |
| 7 | 1.488 | -2.111 | -1.644 |
| 6 | 6.042 | 2.181 | -1.780 |
| 6 | 4.390 | 0.278 | -1.512 |
| 6 | 5.748 | 0.770 | -1.377 |
| 6 | 6.677 | -0.086 | -0.907 |
| 6 | 5.124 | -1.906 | -0.658 |
| 6 | -2.920 | -2.390 | -1.699 |
| 6 | 0.427 | -1.279 | -1.661 |
| 6 | -0.856 | -1.872 | -1.667 |
| 6 | -0.903 | -3.259 | -1.666 |
| 6 | 1.291 | -3.436 | -1.632 |
| 1 | 7.710 | 0.200 | -0.772 |
| 1 | 7.101 | -1.983 | -0.203 |
| 1 | 3.231 | -1.418 | -1.277 |
| 1 | 1.577 | 0.389 | -1.694 |
| 1 | -0.145 | 0.682 | -1.565 |
| 1 | -3.997 | -2.358 | -1.701 |
| 1 | -2.639 | -4.493 | -1.688 |
| 1 | 2.194 | -4.034 | -1.605 |
| 1 | 5.870 | 2.319 | -2.849 |
| 1 | 7.077 | 2.431 | -1.556 |
| 1 | 5.388 | 2.873 | -1.251 |
| 6 | -2.312 | 4.921 | -0.975 |
| 6 | -3.141 | 3.704 | -1.242 |
| 6 | -4.473 | 3.729 | -1.439 |
| 7 | -5.204 | 2.594 | -1.668 |
| 1 | -6.203 | 2.652 | -1.797 |
| 6 | -4.653 | 1.342 | -1.710 |
| 8 | -5.309 | 0.328 | -1.897 |
| 7 | -3.294 | 1.324 | -1.526 |
| 1 | -2.842 | 0.397 | -1.570 |
| 6 | -2.480 | 2.414 | -1.294 |
| 8 | -1.269 | 2.258 | -1.152 |
| 1 | -5.044 | 4.647 | -1.422 |
| 1 | -2.939 | 5.810 | -0.953 |
| 1 | -1.551 | 5.045 | -1.746 |
| 1 | -1.796 | 4.829 | -0.018 |
| 8 | 5.164 | -0.022 | 2.062 |
| 8 | 1.641 | 2.512 | 0.735 |
| 7 | 5.475 | 2.218 | 1.781 |
| 7 | 3.415 | 1.275 | 1.397 |
| 7 | -0.248 | 0.304 | 1.531 |
| 7 | -1.799 | -2.348 | 1.574 |
| 7 | -0.680 | -4.271 | 1.654 |
| 7 | 1.606 | -3.417 | 1.689 |
| 7 | 1.650 | -1.023 | 1.588 |
| 6 | 3.129 | 4.962 | 0.672 |
| 6 | 2.832 | 2.473 | 1.041 |
| 6 | 3.700 | 3.636 | 1.065 |
| 6 | 4.979 | 3.450 | 1.442 |
| 6 | 4.720 | 1.077 | 1.769 |
| 6 | -1.894 | -3.654 | 1.591 |
| 6 | 0.307 | -0.904 | 1.580 |
| 6 | -0.443 | -2.098 | 1.621 |
| 6 | 0.271 | -3.288 | 1.665 |
| 6 | 2.207 | -2.240 | 1.642 |
| 1 | 5.692 | 4.261 | 1.494 |
| 1 | 6.443 | 2.118 | 2.047 |
| 1 | 2.802 | 0.441 | 1.421 |
| 1 | 0.344 | 1.107 | 1.353 |
| 1 | -1.255 | 0.420 | 1.536 |
| 1 | -2.815 | -4.212 | 1.556 |
| 1 | -0.516 | -5.266 | 1.658 |
| 1 | 3.290 | -2.251 | 1.644 |
| 1 | 2.758 | 4.930 | -0.353 |
| 1 | 3.888 | 5.739 | 0.746 |
| 1 | 2.290 | 5.226 | 1.317 |
| 6 | -5.396 | 2.813 | 1.948 |
| 6 | -5.398 | 1.366 | 1.563 |
| 6 | -6.507 | 0.678 | 1.230 |
| 7 | -6.477 | -0.651 | 0.904 |
| 1 | -7.327 | -1.127 | 0.643 |
| 6 | -5.327 | -1.389 | 0.873 |
| 8 | -5.302 | -2.573 | 0.576 |
| 7 | -4.199 | -0.684 | 1.212 |
| 1 | -3.325 | -1.227 | 1.265 |
| 6 | -4.137 | 0.648 | 1.562 |
| 8 | -3.056 | 1.162 | 1.845 |
| 1 | -7.485 | 1.135 | 1.204 |
| 1 | -6.390 | 3.239 | 1.828 |
| 1 | -4.695 | 3.370 | 1.326 |
| 1 | -5.083 | 2.935 | 2.985 |

rT•A-T/rT•A-T

| 8 | -4.380 | 2.289 | -1.826 |
| --- | --- | --- | --- |
| 8 | -2.065 | -1.616 | -1.814 |
| 7 | -5.528 | 0.339 | -2.089 |
| 7 | -3.244 | 0.316 | -1.829 |
| 7 | 0.524 | -0.122 | -1.402 |
| 7 | 2.895 | 1.800 | -1.001 |
| 7 | 2.525 | 3.993 | -0.940 |
| 7 | 0.118 | 4.018 | -1.336 |
| 7 | -0.758 | 1.799 | -1.559 |
| 6 | -4.378 | -3.258 | -2.187 |
| 6 | -3.158 | -1.058 | -1.906 |
| 6 | -4.412 | -1.765 | -2.099 |
| 6 | -5.536 | -1.029 | -2.184 |
| 6 | -4.385 | 1.071 | -1.908 |
| 6 | 3.435 | 2.982 | -0.846 |
| 6 | 0.441 | 1.205 | -1.394 |
| 6 | 1.555 | 2.052 | -1.208 |
| 6 | 1.306 | 3.417 | -1.176 |
| 6 | -0.849 | 3.135 | -1.525 |
| 1 | -6.511 | -1.473 | -2.323 |
| 1 | -6.392 | 0.856 | -2.154 |
| 1 | -2.359 | 0.840 | -1.706 |
| 1 | -0.321 | -0.666 | -1.542 |
| 1 | 1.417 | -0.595 | -1.333 |
| 1 | 4.481 | 3.168 | -0.667 |
| 1 | 2.717 | 4.979 | -0.856 |
| 1 | -1.849 | 3.528 | -1.665 |
| 1 | -3.891 | -3.673 | -1.305 |
| 1 | -3.811 | -3.581 | -3.060 |
| 1 | -5.388 | -3.658 | -2.254 |
| 6 | 8.383 | -1.098 | -1.310 |
| 6 | 6.908 | -1.347 | -1.353 |
| 6 | 6.359 | -2.564 | -1.522 |
| 7 | 5.001 | -2.760 | -1.562 |
| 1 | 4.625 | -3.689 | -1.681 |
| 6 | 4.099 | -1.744 | -1.441 |
| 8 | 2.887 | -1.927 | -1.467 |
| 7 | 4.657 | -0.507 | -1.279 |
| 1 | 3.999 | 0.282 | -1.177 |
| 6 | 6.009 | -0.213 | -1.210 |
| 8 | 6.376 | 0.942 | -1.031 |
| 1 | 6.954 | -3.460 | -1.634 |
| 1 | 8.929 | -2.029 | -1.451 |
| 1 | 8.671 | -0.663 | -0.352 |
| 1 | 8.674 | -0.395 | -2.091 |
| 8 | -6.251 | 1.489 | 0.942 |
| 8 | -3.750 | -2.291 | 1.219 |
| 7 | -7.306 | -0.529 | 0.860 |
| 7 | -5.021 | -0.423 | 1.082 |
| 7 | -1.244 | -0.624 | 1.506 |
| 7 | 1.008 | 1.431 | 1.981 |
| 7 | 0.516 | 3.599 | 2.022 |
| 7 | -1.890 | 3.485 | 1.634 |
| 7 | -2.638 | 1.220 | 1.410 |
| 6 | -6.014 | -4.064 | 1.167 |
| 6 | -4.872 | -1.796 | 1.127 |
| 6 | -6.098 | -2.572 | 1.063 |
| 6 | -7.255 | -1.897 | 0.930 |
| 6 | -6.199 | 0.269 | 0.964 |
| 6 | 1.481 | 2.643 | 2.118 |
| 6 | -1.405 | 0.696 | 1.560 |
| 6 | -0.345 | 1.604 | 1.777 |
| 6 | -0.670 | 2.953 | 1.799 |
| 6 | -2.805 | 2.548 | 1.448 |
| 1 | -8.212 | -2.394 | 0.869 |
| 1 | -8.195 | -0.061 | 0.769 |
| 1 | -4.164 | 0.152 | 1.176 |
| 1 | -2.054 | -1.213 | 1.347 |
| 1 | -0.323 | -1.042 | 1.549 |
| 1 | 2.517 | 2.889 | 2.286 |
| 1 | 0.654 | 4.596 | 2.085 |
| 1 | -3.825 | 2.884 | 1.308 |
| 1 | -5.538 | -4.354 | 2.104 |
| 1 | -5.420 | -4.478 | 0.354 |
| 1 | -7.011 | -4.500 | 1.131 |
| 6 | 6.654 | -1.101 | 2.111 |
| 6 | 5.211 | -1.440 | 1.897 |
| 6 | 4.752 | -2.692 | 1.714 |
| 7 | 3.419 | -2.971 | 1.548 |
| 1 | 3.112 | -3.922 | 1.412 |
| 6 | 2.449 | -2.009 | 1.562 |
| 8 | 1.258 | -2.266 | 1.435 |
| 7 | 2.918 | -0.737 | 1.734 |
| 1 | 2.207 | 0.009 | 1.779 |
| 6 | 4.238 | -0.359 | 1.911 |
| 8 | 4.524 | 0.824 | 2.061 |
| 1 | 5.406 | -3.552 | 1.686 |
| 1 | 7.279 | -1.977 | 1.952 |
| 1 | 6.814 | -0.732 | 3.125 |
| 1 | 6.967 | -0.314 | 1.425 |

rG•G-C/rG•G-C

| 7 | -3.470 | -3.260 | 1.972 |
| --- | --- | --- | --- |
| 8 | -0.948 | 0.532 | 1.519 |
| 7 | -2.125 | -1.408 | 1.722 |
| 6 | -2.230 | -2.770 | 1.859 |
| 7 | -1.185 | -3.576 | 1.902 |
| 6 | -0.015 | -2.920 | 1.810 |
| 6 | 0.195 | -1.554 | 1.684 |
| 6 | -0.936 | -0.702 | 1.632 |
| 7 | 1.236 | -3.464 | 1.827 |
| 6 | 2.131 | -2.436 | 1.708 |
| 7 | 1.545 | -1.273 | 1.622 |
| 1 | -4.290 | -2.675 | 1.819 |
| 1 | -3.570 | -4.260 | 1.976 |
| 1 | -2.987 | -0.839 | 1.693 |
| 1 | 1.459 | -4.445 | 1.902 |
| 1 | 3.194 | -2.608 | 1.686 |
| 8 | -5.813 | -1.593 | 1.554 |
| 7 | -3.325 | 2.229 | 1.733 |
| 7 | -4.538 | 0.295 | 1.617 |
| 6 | -5.696 | 2.419 | 1.477 |
| 6 | -5.717 | -0.360 | 1.522 |
| 7 | -6.871 | 0.393 | 1.386 |
| 6 | -6.860 | 1.747 | 1.361 |
| 6 | -4.507 | 1.631 | 1.607 |
| 1 | -5.653 | 3.496 | 1.463 |
| 1 | -7.740 | -0.113 | 1.299 |
| 1 | -7.819 | 2.230 | 1.247 |
| 1 | -2.478 | 1.669 | 1.720 |
| 1 | -3.258 | 3.230 | 1.659 |
| 6 | 6.640 | 3.750 | 1.110 |
| 7 | 5.449 | 4.308 | 0.716 |
| 1 | 5.310 | 5.245 | 0.371 |
| 6 | 4.488 | 3.355 | 0.875 |
| 6 | 5.161 | 2.249 | 1.369 |
| 7 | 6.510 | 2.515 | 1.508 |
| 6 | 4.419 | 1.054 | 1.604 |
| 8 | 4.830 | -0.031 | 2.008 |
| 7 | 3.064 | 1.230 | 1.310 |
| 1 | 2.475 | 0.396 | 1.439 |
| 6 | 2.493 | 2.383 | 0.845 |
| 7 | 3.175 | 3.484 | 0.599 |
| 7 | 1.168 | 2.353 | 0.615 |
| 1 | 7.559 | 4.311 | 1.080 |
| 1 | 0.576 | 1.608 | 0.968 |
| 1 | 0.735 | 3.234 | 0.398 |
| 7 | -4.525 | -1.865 | -1.501 |
| 8 | -0.447 | 0.197 | -1.777 |
| 7 | -2.436 | -0.908 | -1.633 |
| 6 | -3.202 | -2.043 | -1.518 |
| 7 | -2.691 | -3.258 | -1.432 |
| 6 | -1.348 | -3.263 | -1.471 |
| 6 | -0.490 | -2.180 | -1.599 |
| 6 | -1.052 | -0.881 | -1.681 |
| 7 | -0.527 | -4.349 | -1.386 |
| 6 | 0.762 | -3.893 | -1.454 |
| 7 | 0.826 | -2.597 | -1.586 |
| 1 | -4.953 | -0.946 | -1.597 |
| 1 | -5.102 | -2.677 | -1.370 |
| 1 | -2.912 | 0.009 | -1.653 |
| 1 | -0.817 | -5.308 | -1.273 |
| 1 | 1.607 | -4.558 | -1.398 |
| 8 | -5.773 | 0.719 | -1.867 |
| 7 | -1.753 | 2.815 | -1.354 |
| 7 | -3.747 | 1.735 | -1.635 |
| 6 | -3.729 | 4.155 | -1.512 |
| 6 | -5.091 | 1.748 | -1.785 |
| 7 | -5.726 | 2.976 | -1.854 |
| 6 | -5.065 | 4.148 | -1.704 |
| 6 | -3.074 | 2.881 | -1.503 |
| 1 | -3.173 | 5.071 | -1.395 |
| 1 | -6.729 | 2.964 | -1.969 |
| 1 | -5.665 | 5.044 | -1.748 |
| 1 | -1.276 | 1.922 | -1.438 |
| 1 | -1.210 | 3.660 | -1.301 |
| 6 | 7.653 | -0.688 | -0.632 |
| 7 | 6.964 | 0.416 | -1.065 |
| 1 | 7.324 | 1.354 | -1.145 |
| 6 | 5.681 | 0.024 | -1.293 |
| 6 | 5.663 | -1.330 | -0.992 |
| 7 | 6.909 | -1.758 | -0.577 |
| 6 | 4.421 | -2.025 | -1.073 |
| 8 | 4.191 | -3.208 | -0.826 |
| 7 | 3.389 | -1.177 | -1.478 |
| 1 | 2.461 | -1.618 | -1.547 |
| 6 | 3.518 | 0.153 | -1.772 |
| 7 | 4.660 | 0.806 | -1.698 |
| 7 | 2.407 | 0.791 | -2.186 |
| 1 | 8.699 | -0.633 | -0.378 |
| 1 | 1.484 | 0.406 | -2.010 |
| 1 | 2.476 | 1.794 | -2.235 |

rA•A-T/rA•A-T

| 8 | 5.275 | -0.500 | -1.895 |
| --- | --- | --- | --- |
| 8 | 1.497 | -2.700 | -0.680 |
| 8 | 3.075 | -0.901 | 1.986 |
| 8 | 5.274 | 2.697 | 0.302 |
| 7 | 5.388 | -2.748 | -1.538 |
| 7 | 3.395 | -1.631 | -1.287 |
| 7 | -0.153 | -0.299 | -1.473 |
| 7 | -1.430 | 2.475 | -1.830 |
| 7 | -0.116 | 4.252 | -2.120 |
| 7 | 2.070 | 3.178 | -1.998 |
| 7 | 1.870 | 0.820 | -1.621 |
| 7 | -6.337 | 0.735 | -0.800 |
| 7 | -6.587 | -1.619 | -1.211 |
| 7 | -4.460 | -2.661 | -1.795 |
| 7 | -3.128 | -0.876 | -1.700 |
| 7 | -4.324 | 1.862 | -1.008 |
| 7 | -4.220 | 0.479 | 2.286 |
| 7 | -1.872 | -1.329 | 1.485 |
| 7 | -2.252 | -3.474 | 1.018 |
| 7 | -4.659 | -3.560 | 1.411 |
| 7 | -5.527 | -1.420 | 2.072 |
| 7 | 1.729 | 2.460 | 1.398 |
| 7 | 0.787 | 4.640 | 1.075 |
| 7 | -1.648 | 4.596 | 1.233 |
| 7 | -1.962 | 2.427 | 1.638 |
| 7 | 0.473 | 0.560 | 1.817 |
| 7 | 4.194 | 0.879 | 1.148 |
| 7 | 6.459 | 0.806 | 0.756 |
| 6 | 2.771 | -5.266 | -0.507 |
| 6 | 2.700 | -2.768 | -0.933 |
| 6 | 3.467 | -3.999 | -0.893 |
| 6 | 4.776 | -3.926 | -1.204 |
| 6 | 4.728 | -1.550 | -1.595 |
| 6 | -1.387 | 3.770 | -2.014 |
| 6 | 0.522 | 0.838 | -1.627 |
| 6 | -0.105 | 2.090 | -1.806 |
| 6 | 0.729 | 3.186 | -1.981 |
| 6 | 2.549 | 1.960 | -1.803 |
| 6 | -5.019 | 0.723 | -1.066 |
| 6 | -4.425 | -0.512 | -1.398 |
| 6 | -5.269 | -1.614 | -1.451 |
| 6 | -7.024 | -0.410 | -0.886 |
| 6 | -3.196 | -2.164 | -1.923 |
| 6 | -1.339 | -2.464 | 1.110 |
| 6 | -5.619 | -2.723 | 1.778 |
| 6 | -3.469 | -2.948 | 1.356 |
| 6 | -3.214 | -1.613 | 1.640 |
| 6 | -4.326 | -0.823 | 2.001 |
| 6 | 1.786 | 3.778 | 1.162 |
| 6 | -0.399 | 4.038 | 1.252 |
| 6 | -0.614 | 2.690 | 1.503 |
| 6 | 0.532 | 1.867 | 1.573 |
| 6 | -2.538 | 3.590 | 1.467 |
| 6 | 5.309 | 1.544 | 0.704 |
| 6 | 6.497 | -0.491 | 1.187 |
| 6 | 5.400 | -1.144 | 1.618 |
| 6 | 4.143 | -0.419 | 1.612 |
| 6 | 5.409 | -2.554 | 2.120 |
| 1 | 5.420 | -4.794 | -1.201 |
| 1 | 6.373 | -2.733 | -1.758 |
| 1 | 2.857 | -0.748 | -1.357 |
| 1 | 0.358 | -1.148 | -1.259 |
| 1 | -1.168 | -0.307 | -1.474 |
| 1 | -2.246 | 4.419 | -2.080 |
| 1 | 0.149 | 5.217 | -2.244 |
| 1 | 3.627 | 1.860 | -1.787 |
| 1 | -8.081 | -0.330 | -0.663 |
| 1 | -2.362 | -2.797 | -2.180 |
| 1 | -4.809 | 2.712 | -0.773 |
| 1 | -3.339 | 1.898 | -1.248 |
| 1 | -4.736 | -3.626 | -1.898 |
| 1 | 1.938 | -5.468 | -1.180 |
| 1 | 2.366 | -5.187 | 0.503 |
| 1 | 3.464 | -6.105 | -0.541 |
| 1 | 6.397 | -2.994 | 1.997 |
| 1 | 5.138 | -2.587 | 3.176 |
| 1 | 4.683 | -3.158 | 1.576 |
| 1 | -2.063 | -4.427 | 0.746 |
| 1 | -3.362 | 0.988 | 2.100 |
| 1 | -5.070 | 0.998 | 2.435 |
| 1 | -0.297 | -2.619 | 0.878 |
| 1 | -6.615 | -3.142 | 1.849 |
| 1 | 2.784 | 4.176 | 1.023 |
| 1 | -1.870 | 5.568 | 1.078 |
| 1 | -3.600 | 3.770 | 1.499 |
| 1 | -0.419 | 0.074 | 1.813 |
| 1 | 1.335 | 0.024 | 1.827 |
| 1 | 3.320 | 1.430 | 1.187 |
| 1 | 7.299 | 1.254 | 0.422 |
| 1 | 7.473 | -0.956 | 1.160 |

G4∙∙∙Na^+^

| 11 | 0.000 | 0.000 | -0.044 |
| --- | --- | --- | --- |
| 8 | -1.333 | -1.891 | -0.013 |
| 8 | 1.895 | -1.325 | -0.044 |
| 8 | -1.894 | 1.325 | -0.042 |
| 8 | 1.332 | 1.891 | -0.013 |
| 7 | -5.597 | -0.203 | 0.129 |
| 7 | -5.418 | -2.493 | 0.192 |
| 7 | -3.482 | -1.130 | 0.057 |
| 7 | -2.598 | -4.697 | 0.124 |
| 7 | -4.815 | -4.835 | 0.237 |
| 7 | 0.203 | -5.588 | -0.108 |
| 7 | 2.494 | -5.414 | -0.159 |
| 7 | 1.132 | -3.474 | -0.072 |
| 7 | 4.701 | -2.595 | -0.129 |
| 7 | 4.837 | -4.814 | -0.198 |
| 7 | -0.202 | 5.589 | -0.108 |
| 7 | -2.493 | 5.414 | -0.161 |
| 7 | -1.132 | 3.475 | -0.071 |
| 7 | -4.700 | 2.596 | -0.129 |
| 7 | -4.836 | 4.814 | -0.201 |
| 7 | 5.596 | 0.202 | 0.124 |
| 7 | 5.417 | 2.492 | 0.191 |
| 7 | 3.481 | 1.129 | 0.054 |
| 7 | 2.598 | 4.696 | 0.125 |
| 7 | 4.814 | 4.834 | 0.239 |
| 6 | -4.845 | -1.304 | 0.125 |
| 6 | -2.545 | -2.152 | 0.046 |
| 6 | -3.150 | -3.431 | 0.111 |
| 6 | -4.535 | -3.502 | 0.182 |
| 6 | -3.620 | -5.504 | 0.198 |
| 6 | 1.305 | -4.839 | -0.113 |
| 6 | 2.156 | -2.538 | -0.075 |
| 6 | 3.434 | -3.146 | -0.117 |
| 6 | 3.504 | -4.532 | -0.159 |
| 6 | 5.507 | -3.619 | -0.177 |
| 6 | -1.304 | 4.839 | -0.113 |
| 6 | -2.155 | 2.539 | -0.074 |
| 6 | -3.433 | 3.146 | -0.118 |
| 6 | -3.503 | 4.533 | -0.160 |
| 6 | -5.506 | 3.620 | -0.179 |
| 6 | 4.844 | 1.303 | 0.123 |
| 6 | 2.544 | 2.152 | 0.045 |
| 6 | 3.149 | 3.430 | 0.111 |
| 6 | 4.534 | 3.502 | 0.182 |
| 6 | 3.619 | 5.503 | 0.201 |
| 1 | -6.593 | -0.332 | 0.159 |
| 1 | -5.218 | 0.739 | 0.029 |
| 1 | -3.573 | -6.579 | 0.228 |
| 1 | -3.100 | -0.181 | 0.014 |
| 1 | 0.330 | -6.586 | -0.119 |
| 1 | -0.739 | -5.205 | -0.029 |
| 1 | 6.583 | -3.574 | -0.198 |
| 1 | 0.184 | -3.091 | -0.044 |
| 1 | -0.328 | 6.586 | -0.121 |
| 1 | 0.740 | 5.205 | -0.028 |
| 1 | -6.582 | 3.575 | -0.200 |
| 1 | -0.183 | 3.092 | -0.044 |
| 1 | 6.592 | 0.331 | 0.156 |
| 1 | 5.218 | -0.740 | 0.025 |
| 1 | 3.572 | 6.579 | 0.232 |
| 1 | 3.098 | 0.181 | 0.011 |
| 1 | -5.251 | 5.733 | -0.236 |
| 1 | -5.732 | -5.250 | 0.294 |
| 1 | 5.251 | -5.732 | -0.233 |
| 1 | 5.731 | 5.249 | 0.297 |

G4∙∙∙K^+^

| 8 | -1.292 | -1.986 | 0.389 |
| --- | --- | --- | --- |
| 8 | -1.946 | 1.238 | 0.213 |
| 8 | 1.946 | -1.238 | 0.212 |
| 8 | 1.293 | 1.986 | 0.389 |
| 7 | 1.919 | -5.037 | -0.800 |
| 7 | -0.261 | -5.752 | -0.965 |
| 7 | 0.242 | -3.561 | -0.211 |
| 7 | -3.387 | -4.137 | -0.298 |
| 7 | -2.655 | -6.108 | -1.022 |
| 7 | -5.248 | -1.933 | 0.462 |
| 7 | -5.956 | 0.256 | 0.393 |
| 7 | -3.648 | -0.273 | 0.311 |
| 7 | -4.182 | 3.361 | 0.193 |
| 7 | -6.287 | 2.653 | 0.320 |
| 7 | 5.248 | 1.933 | 0.461 |
| 7 | 5.956 | -0.257 | 0.393 |
| 7 | 3.648 | 0.273 | 0.310 |
| 7 | 4.182 | -3.362 | 0.193 |
| 7 | 6.287 | -2.654 | 0.321 |
| 7 | -1.919 | 5.037 | -0.799 |
| 7 | 0.261 | 5.752 | -0.964 |
| 7 | -0.242 | 3.562 | -0.210 |
| 7 | 3.388 | 4.137 | -0.299 |
| 7 | 2.655 | 6.108 | -1.022 |
| 6 | 0.610 | -4.808 | -0.659 |
| 6 | -1.064 | -3.134 | -0.020 |
| 6 | -2.007 | -4.140 | -0.344 |
| 6 | -1.536 | -5.366 | -0.793 |
| 6 | -3.735 | -5.324 | -0.710 |
| 6 | -4.975 | -0.626 | 0.379 |
| 6 | -3.167 | 1.027 | 0.249 |
| 6 | -4.214 | 1.982 | 0.247 |
| 6 | -5.523 | 1.525 | 0.327 |
| 6 | -5.435 | 3.723 | 0.237 |
| 6 | 4.975 | 0.626 | 0.379 |
| 6 | 3.167 | -1.027 | 0.249 |
| 6 | 4.214 | -1.982 | 0.247 |
| 6 | 5.522 | -1.525 | 0.327 |
| 6 | 5.434 | -3.723 | 0.238 |
| 6 | -0.610 | 4.808 | -0.658 |
| 6 | 1.065 | 3.134 | -0.019 |
| 6 | 2.007 | 4.141 | -0.344 |
| 6 | 1.536 | 5.366 | -0.793 |
| 6 | 3.735 | 5.324 | -0.710 |
| 1 | 2.186 | -5.972 | -1.056 |
| 1 | 2.632 | -4.415 | -0.421 |
| 1 | -4.744 | -5.688 | -0.807 |
| 1 | 0.967 | -2.859 | -0.037 |
| 1 | -6.220 | -2.188 | 0.414 |
| 1 | -4.552 | -2.647 | 0.246 |
| 1 | -5.800 | 4.736 | 0.211 |
| 1 | -2.934 | -1.007 | 0.319 |
| 1 | 6.221 | 2.188 | 0.414 |
| 1 | 4.552 | 2.647 | 0.246 |
| 1 | 5.800 | -4.736 | 0.212 |
| 1 | 2.934 | 1.007 | 0.318 |
| 1 | -2.186 | 5.972 | -1.054 |
| 1 | -2.632 | 4.415 | -0.420 |
| 1 | 4.744 | 5.688 | -0.808 |
| 1 | -0.966 | 2.859 | -0.036 |
| 1 | 2.683 | 7.059 | -1.357 |
| 1 | -7.294 | 2.692 | 0.367 |
| 1 | -2.683 | -7.059 | -1.357 |
| 1 | 7.293 | -2.692 | 0.369 |
| 19 | 0.000 | 0.000 | 1.593 |

G4∙∙∙Na^+^∙∙∙G4

| 8 | 1.089 | 1.956 | -1.366 |
| --- | --- | --- | --- |
| 8 | 1.974 | -1.091 | -1.365 |
| 8 | -1.975 | 1.083 | -1.361 |
| 8 | -1.089 | -1.959 | -1.367 |
| 7 | -2.194 | 5.147 | -1.704 |
| 7 | -0.031 | 5.921 | -1.738 |
| 7 | -0.486 | 3.600 | -1.538 |
| 7 | 3.126 | 4.262 | -1.491 |
| 7 | 2.351 | 6.335 | -1.682 |
| 7 | 5.162 | 2.196 | -1.695 |
| 7 | 5.939 | 0.035 | -1.727 |
| 7 | 3.617 | 0.486 | -1.534 |
| 7 | 4.284 | -3.125 | -1.484 |
| 7 | 6.356 | -2.346 | -1.669 |
| 7 | -5.165 | -2.201 | -1.701 |
| 7 | -5.940 | -0.039 | -1.736 |
| 7 | -3.619 | -0.493 | -1.536 |
| 7 | -4.281 | 3.119 | -1.487 |
| 7 | -6.354 | 2.343 | -1.680 |
| 7 | 2.200 | -5.145 | -1.699 |
| 7 | 0.039 | -5.922 | -1.739 |
| 7 | 0.490 | -3.601 | -1.537 |
| 7 | -3.122 | -4.269 | -1.494 |
| 7 | -2.342 | -6.340 | -1.687 |
| 6 | -0.882 | 4.912 | -1.658 |
| 6 | 0.830 | 3.162 | -1.473 |
| 6 | 1.748 | 4.242 | -1.541 |
| 6 | 1.249 | 5.531 | -1.662 |
| 6 | 3.448 | 5.523 | -1.574 |
| 6 | 4.929 | 0.884 | -1.650 |
| 6 | 3.181 | -0.831 | -1.469 |
| 6 | 4.263 | -1.746 | -1.535 |
| 6 | 5.551 | -1.246 | -1.652 |
| 6 | 5.546 | -3.444 | -1.563 |
| 6 | -4.931 | -0.889 | -1.656 |
| 6 | -3.181 | 0.824 | -1.469 |
| 6 | -4.262 | 1.741 | -1.539 |
| 6 | -5.550 | 1.242 | -1.660 |
| 6 | -5.543 | 3.440 | -1.571 |
| 6 | 0.888 | -4.912 | -1.656 |
| 6 | -0.828 | -3.165 | -1.473 |
| 6 | -1.743 | -4.247 | -1.543 |
| 6 | -1.242 | -5.535 | -1.665 |
| 6 | -3.441 | -5.531 | -1.580 |
| 1 | -2.486 | 6.109 | -1.736 |
| 1 | -2.891 | 4.410 | -1.590 |
| 1 | 4.449 | 5.921 | -1.565 |
| 1 | -1.189 | 2.857 | -1.480 |
| 1 | 6.123 | 2.491 | -1.727 |
| 1 | 4.421 | 2.890 | -1.586 |
| 1 | 5.945 | -4.445 | -1.553 |
| 1 | 2.873 | 1.188 | -1.478 |
| 1 | -6.126 | -2.494 | -1.736 |
| 1 | -4.425 | -2.896 | -1.590 |
| 1 | -5.940 | 4.441 | -1.563 |
| 1 | -2.875 | -1.196 | -1.476 |
| 1 | 2.493 | -6.106 | -1.731 |
| 1 | 2.896 | -4.406 | -1.585 |
| 1 | -4.441 | -5.930 | -1.574 |
| 1 | 1.192 | -2.857 | -1.478 |
| 1 | -7.360 | 2.348 | -1.758 |
| 1 | 2.356 | 7.340 | -1.759 |
| 1 | 7.362 | -2.350 | -1.745 |
| 1 | -2.345 | -7.346 | -1.767 |
| 11 | -0.004 | -0.003 | -0.031 |
| 8 | -0.610 | 2.173 | 1.328 |
| 8 | 2.170 | 0.616 | 1.325 |
| 8 | -2.173 | -0.605 | 1.329 |
| 8 | 0.606 | -2.173 | 1.323 |
| 7 | -5.183 | 2.072 | 1.705 |
| 7 | -4.217 | 4.154 | 1.753 |
| 7 | -2.884 | 2.205 | 1.519 |
| 7 | -0.823 | 5.246 | 1.489 |
| 7 | -2.844 | 6.142 | 1.707 |
| 7 | 2.077 | 5.191 | 1.697 |
| 7 | 4.157 | 4.221 | 1.746 |
| 7 | 2.205 | 2.890 | 1.514 |
| 7 | 5.242 | 0.824 | 1.487 |
| 7 | 6.143 | 2.843 | 1.703 |
| 7 | -2.074 | -5.179 | 1.713 |
| 7 | -4.155 | -4.211 | 1.758 |
| 7 | -2.205 | -2.879 | 1.523 |
| 7 | -5.246 | -0.818 | 1.490 |
| 7 | -6.142 | -2.838 | 1.711 |
| 7 | 5.179 | -2.073 | 1.717 |
| 7 | 4.211 | -4.154 | 1.760 |
| 7 | 2.879 | -2.205 | 1.522 |
| 7 | 0.819 | -5.247 | 1.483 |
| 7 | 2.839 | -6.142 | 1.708 |
| 6 | -4.095 | 2.841 | 1.657 |
| 6 | -1.648 | 2.836 | 1.451 |
| 6 | -1.774 | 4.248 | 1.538 |
| 6 | -3.042 | 4.793 | 1.675 |
| 6 | -1.497 | 6.358 | 1.589 |
| 6 | 2.844 | 4.101 | 1.651 |
| 6 | 2.834 | 1.654 | 1.447 |
| 6 | 4.246 | 1.777 | 1.534 |
| 6 | 4.795 | 3.044 | 1.669 |
| 6 | 6.356 | 1.496 | 1.587 |
| 6 | -2.842 | -4.090 | 1.663 |
| 6 | -2.836 | -1.644 | 1.454 |
| 6 | -4.248 | -1.769 | 1.540 |
| 6 | -4.794 | -3.036 | 1.679 |
| 6 | -6.358 | -1.492 | 1.591 |
| 6 | 4.090 | -2.841 | 1.664 |
| 6 | 1.644 | -2.836 | 1.449 |
| 6 | 1.769 | -4.248 | 1.535 |
| 6 | 3.037 | -4.793 | 1.677 |
| 6 | 1.493 | -6.358 | 1.585 |
| 1 | -6.071 | 2.541 | 1.760 |
| 1 | -5.151 | 1.058 | 1.589 |
| 1 | -1.080 | 7.351 | 1.586 |
| 1 | -2.846 | 1.183 | 1.452 |
| 1 | 2.547 | 6.078 | 1.748 |
| 1 | 1.063 | 5.160 | 1.581 |
| 1 | 7.348 | 1.076 | 1.585 |
| 1 | 1.183 | 2.856 | 1.447 |
| 1 | -2.544 | -6.066 | 1.767 |
| 1 | -1.060 | -5.149 | 1.594 |
| 1 | -7.351 | -1.074 | 1.587 |
| 1 | -1.183 | -2.842 | 1.455 |
| 1 | 6.065 | -2.544 | 1.773 |
| 1 | 5.148 | -1.059 | 1.600 |
| 1 | 1.076 | -7.351 | 1.580 |
| 1 | 2.842 | -1.183 | 1.455 |
| 1 | -6.849 | -3.552 | 1.801 |
| 1 | -3.559 | 6.848 | 1.796 |
| 1 | 6.850 | 3.557 | 1.792 |
| 1 | 3.554 | -6.847 | 1.800 |

G4∙∙∙K^+^∙∙∙G4

| 0.071 | 2.319 | -1.585 |
| --- | --- | --- |
| 2.318 | -0.075 | -1.586 |
| -2.321 | 0.071 | -1.587 |
| -0.074 | -2.319 | -1.588 |
| -4.364 | 3.498 | -1.690 |
| -2.860 | 5.237 | -1.646 |
| -2.113 | 2.985 | -1.618 |
| 0.716 | 5.324 | -1.526 |
| -0.980 | 6.761 | -1.549 |
| 3.495 | 4.361 | -1.691 |
| 5.235 | 2.857 | -1.647 |
| 2.983 | 2.110 | -1.618 |
| 5.322 | -0.719 | -1.528 |
| 6.759 | 0.977 | -1.551 |
| -3.500 | -4.363 | -1.691 |
| -5.240 | -2.859 | -1.644 |
| -2.988 | -2.112 | -1.619 |
| -5.327 | 0.717 | -1.525 |
| -6.763 | -0.979 | -1.546 |
| 4.359 | -3.498 | -1.693 |
| 2.856 | -5.238 | -1.644 |
| 2.109 | -2.986 | -1.619 |
| -0.720 | -5.325 | -1.525 |
| 0.976 | -6.761 | -1.545 |
| -3.104 | 3.940 | -1.645 |
| -0.749 | 3.245 | -1.589 |
| -0.481 | 4.639 | -1.558 |
| -1.550 | 5.522 | -1.586 |
| 0.378 | 6.584 | -1.515 |
| 3.938 | 3.101 | -1.644 |
| 3.243 | 0.746 | -1.590 |
| 4.637 | 0.478 | -1.559 |
| 5.520 | 1.547 | -1.588 |
| 6.581 | -0.381 | -1.516 |
| -3.943 | -3.103 | -1.644 |
| -3.248 | -0.748 | -1.589 |
| -4.641 | -0.479 | -1.557 |
| -5.525 | -1.549 | -1.584 |
| -6.586 | 0.379 | -1.511 |
| 3.099 | -3.941 | -1.644 |
| 0.745 | -3.246 | -1.590 |
| 0.477 | -4.639 | -1.558 |
| 1.545 | -5.523 | -1.584 |
| -0.382 | -6.584 | -1.511 |
| -5.092 | 4.190 | -1.638 |
| -4.615 | 2.512 | -1.610 |
| 1.057 | 7.419 | -1.502 |
| -2.380 | 1.996 | -1.628 |
| 4.186 | 5.089 | -1.639 |
| 2.509 | 4.609 | -1.611 |
| 7.417 | -1.060 | -1.508 |
| 1.994 | 2.376 | -1.629 |
| -4.192 | -5.091 | -1.639 |
| -2.514 | -4.612 | -1.611 |
| -7.422 | 1.058 | -1.498 |
| -1.999 | -2.378 | -1.629 |
| 5.088 | -4.188 | -1.642 |
| 4.607 | -2.511 | -1.613 |
| -1.062 | -7.419 | -1.500 |
| 2.374 | -1.997 | -1.630 |
| 7.641 | 1.466 | -1.563 |
| 1.464 | -7.644 | -1.555 |
| -7.645 | -1.468 | -1.555 |
| -1.469 | 7.643 | -1.560 |
| -0.003 | -0.001 | -0.034 |
| -1.307 | 1.958 | 1.418 |
| 1.957 | 1.309 | 1.421 |
| -1.953 | -1.313 | 1.415 |
| 1.317 | -1.951 | 1.417 |
| -5.541 | 0.219 | 1.674 |
| -5.397 | 2.514 | 1.659 |
| -3.445 | 1.174 | 1.508 |
| -2.601 | 4.750 | 1.587 |
| -4.818 | 4.865 | 1.699 |
| 0.219 | 5.542 | 1.672 |
| 2.513 | 5.399 | 1.657 |
| 1.174 | 3.446 | 1.508 |
| 4.750 | 2.604 | 1.586 |
| 4.864 | 4.821 | 1.697 |
| -0.218 | -5.546 | 1.673 |
| -2.514 | -5.401 | 1.658 |
| -1.171 | -3.451 | 1.506 |
| -4.745 | -2.602 | 1.589 |
| -4.864 | -4.819 | 1.701 |
| 5.552 | -0.217 | 1.668 |
| 5.406 | -2.512 | 1.653 |
| 3.456 | -1.169 | 1.504 |
| 2.606 | -4.742 | 1.588 |
| 4.823 | -4.863 | 1.698 |
| -4.807 | 1.331 | 1.608 |
| -2.518 | 2.208 | 1.498 |
| -3.140 | 3.479 | 1.582 |
| -4.525 | 3.534 | 1.650 |
| -3.630 | 5.546 | 1.660 |
| 1.331 | 4.809 | 1.607 |
| 2.207 | 2.519 | 1.498 |
| 3.479 | 3.142 | 1.582 |
| 3.533 | 4.527 | 1.649 |
| 5.546 | 3.633 | 1.658 |
| -1.331 | -4.813 | 1.607 |
| -2.204 | -2.523 | 1.495 |
| -3.475 | -3.143 | 1.582 |
| -3.533 | -4.528 | 1.650 |
| -5.544 | -3.629 | 1.663 |
| 4.818 | -1.329 | 1.603 |
| 2.528 | -2.201 | 1.495 |
| 3.148 | -3.473 | 1.580 |
| 4.533 | -3.531 | 1.647 |
| 3.633 | -5.541 | 1.663 |
| -6.540 | 0.334 | 1.674 |
| -5.154 | -0.721 | 1.584 |
| -3.594 | 6.622 | 1.684 |
| -3.051 | 0.228 | 1.483 |
| 0.333 | 6.541 | 1.671 |
| -0.722 | 5.156 | 1.582 |
| 6.622 | 3.598 | 1.682 |
| 0.228 | 3.053 | 1.484 |
| -0.329 | -6.545 | 1.672 |
| 0.718 | -5.151 | 1.583 |
| -6.620 | -3.590 | 1.690 |
| -0.226 | -3.056 | 1.481 |
| 6.551 | -0.327 | 1.665 |
| 5.157 | 0.720 | 1.580 |
| 3.593 | -6.617 | 1.689 |
| 3.061 | -0.223 | 1.481 |
| 5.271 | 5.742 | 1.761 |
| 5.742 | -5.273 | 1.763 |
| -5.274 | -5.738 | 1.766 |
| -5.739 | 5.272 | 1.764 |

Cartesian coordinates of optimized structures in the water phase (IEEPCM) by M05-2X/6-31G(d,p).

A-T

| 7 | -1.472 | 1.326 | 0.000 |
| --- | --- | --- | --- |
| 6 | -2.540 | 0.512 | 0.000 |
| 7 | -3.822 | 0.842 | 0.000 |
| 6 | -3.983 | 2.176 | 0.000 |
| 6 | -2.978 | 3.139 | 0.000 |
| 6 | -1.650 | 2.661 | 0.000 |
| 7 | -5.147 | 2.892 | 0.000 |
| 6 | -4.799 | 4.217 | 0.000 |
| 7 | -3.505 | 4.413 | 0.000 |
| 7 | -0.584 | 3.464 | 0.000 |
| 1 | -2.299 | -0.545 | 0.000 |
| 1 | -6.082 | 2.518 | 0.000 |
| 1 | -5.549 | 4.993 | 0.000 |
| 1 | -0.716 | 4.460 | 0.000 |
| 1 | 0.352 | 3.068 | 0.000 |
| 7 | 1.100 | 0.084 | 0.000 |
| 6 | 1.085 | -1.288 | 0.000 |
| 7 | 2.336 | -1.857 | 0.000 |
| 6 | 3.496 | -1.126 | 0.000 |
| 6 | 3.501 | 0.223 | 0.000 |
| 6 | 2.212 | 0.900 | 0.000 |
| 8 | 2.085 | 2.127 | 0.000 |
| 8 | 0.054 | -1.946 | 0.000 |
| 6 | 4.742 | 1.061 | 0.000 |
| 1 | 0.168 | 0.546 | 0.000 |
| 1 | 2.366 | -2.865 | 0.000 |
| 1 | 4.407 | -1.708 | 0.000 |
| 1 | 5.631 | 0.432 | 0.000 |
| 1 | 4.768 | 1.707 | 0.879 |
| 1 | 4.768 | 1.707 | -0.879 |

G-T

| 6 | -0.317 | 4.603 | 0.000 |
| --- | --- | --- | --- |
| 7 | 0.306 | 3.378 | 0.000 |
| 1 | 1.298 | 3.205 | 0.000 |
| 6 | -0.673 | 2.430 | 0.000 |
| 6 | -1.863 | 3.152 | 0.000 |
| 7 | -1.619 | 4.511 | 0.000 |
| 6 | -3.089 | 2.422 | 0.000 |
| 8 | -4.249 | 2.855 | 0.000 |
| 7 | -2.860 | 1.049 | 0.000 |
| 1 | -3.695 | 0.453 | 0.000 |
| 6 | -1.635 | 0.438 | 0.000 |
| 7 | -0.492 | 1.092 | 0.000 |
| 7 | -1.653 | -0.907 | 0.000 |
| 1 | 0.250 | 5.520 | 0.000 |
| 1 | -2.517 | -1.420 | 0.000 |
| 1 | -0.779 | -1.399 | 0.000 |
| 6 | -10.282 | 1.150 | 0.000 |
| 6 | -8.883 | 0.617 | 0.000 |
| 6 | -8.573 | -0.693 | 0.000 |
| 7 | -7.271 | -1.138 | 0.000 |
| 1 | -7.066 | -2.125 | 0.000 |
| 6 | -6.196 | -0.300 | 0.000 |
| 8 | -5.037 | -0.725 | 0.000 |
| 7 | -6.506 | 1.030 | 0.000 |
| 1 | -5.722 | 1.695 | 0.000 |
| 6 | -7.787 | 1.581 | 0.000 |
| 8 | -7.934 | 2.794 | 0.000 |
| 1 | -9.324 | -1.471 | 0.000 |
| 1 | -11.005 | 0.336 | 0.000 |
| 1 | -10.452 | 1.774 | -0.879 |
| 1 | -10.452 | 1.774 | 0.879 |

G-C

| 7 | -2.615 | 0.013 | 0.033 |
| --- | --- | --- | --- |
| 8 | 1.710 | 1.532 | 0.033 |
| 7 | -0.476 | 0.867 | 0.033 |
| 6 | -1.830 | 1.098 | 0.033 |
| 7 | -2.365 | 2.306 | 0.033 |
| 6 | -1.433 | 3.280 | 0.033 |
| 6 | -0.047 | 3.160 | 0.033 |
| 6 | 0.514 | 1.848 | 0.033 |
| 7 | -1.664 | 4.624 | 0.033 |
| 6 | -0.437 | 5.245 | 0.033 |
| 7 | 0.559 | 4.402 | 0.033 |
| 1 | -2.244 | -0.937 | 0.033 |
| 1 | -3.607 | 0.168 | 0.033 |
| 1 | -0.140 | -0.109 | 0.033 |
| 1 | -2.566 | 5.072 | 0.033 |
| 1 | -0.353 | 6.320 | 0.033 |
| 8 | -1.629 | -2.690 | 0.033 |
| 7 | 2.690 | -1.201 | 0.033 |
| 7 | 0.520 | -1.910 | 0.033 |
| 6 | 2.297 | -3.566 | 0.033 |
| 6 | -0.408 | -2.897 | 0.033 |
| 7 | 0.040 | -4.212 | 0.033 |
| 6 | 1.357 | -4.536 | 0.033 |
| 6 | 1.823 | -2.210 | 0.033 |
| 1 | 3.351 | -3.795 | 0.033 |
| 1 | -0.671 | -4.927 | 0.033 |
| 1 | 1.592 | -5.591 | 0.033 |
| 1 | 2.355 | -0.238 | 0.033 |
| 1 | 3.678 | -1.384 | 0.033 |

C-C

| 6 | 3.409 | 1.005 | 0.000 |
| --- | --- | --- | --- |
| 6 | 4.431 | 1.889 | 0.000 |
| 7 | 4.176 | 3.220 | 0.000 |
| 6 | 2.878 | 3.748 | 0.000 |
| 8 | 2.748 | 4.972 | 0.000 |
| 7 | 1.841 | 2.870 | 0.000 |
| 6 | 2.080 | 1.561 | 0.000 |
| 7 | 1.029 | 0.738 | 0.000 |
| 1 | 3.577 | -0.061 | 0.000 |
| 1 | 5.471 | 1.596 | 0.000 |
| 1 | 0.078 | 1.103 | 0.000 |
| 1 | 1.149 | -0.259 | 0.000 |
| 6 | -3.857 | -1.941 | 0.000 |
| 6 | -4.481 | -0.741 | 0.000 |
| 7 | -3.753 | 0.402 | 0.000 |
| 6 | -2.361 | 0.409 | 0.000 |
| 8 | -1.781 | 1.502 | 0.000 |
| 7 | -1.716 | -0.784 | 0.000 |
| 6 | -2.421 | -1.910 | 0.000 |
| 7 | -1.743 | -3.064 | 0.000 |
| 1 | -4.407 | -2.869 | 0.000 |
| 1 | -5.556 | -0.628 | 0.000 |
| 1 | -0.739 | -3.039 | 0.000 |
| 1 | -2.213 | -3.951 | 0.000 |
| 1 | 4.927 | 3.891 | 0.000 |
| 1 | -4.204 | 1.304 | 0.000 |

A-T/A-T

| 8 | -2.698 | -0.964 | -1.985 |
| --- | --- | --- | --- |
| 8 | -1.102 | 3.028 | -0.483 |
| 7 | -4.186 | 0.654 | -1.368 |
| 7 | -1.922 | 1.054 | -1.245 |
| 7 | 1.684 | 2.151 | -1.205 |
| 7 | 4.355 | 0.657 | -1.446 |
| 7 | 4.348 | -1.545 | -1.851 |
| 7 | 1.943 | -1.936 | -1.997 |
| 7 | 0.711 | 0.093 | -1.656 |
| 6 | -3.691 | 4.146 | -0.005 |
| 6 | -2.085 | 2.327 | -0.739 |
| 6 | -3.458 | 2.770 | -0.546 |
| 6 | -4.443 | 1.910 | -0.877 |
| 6 | -2.917 | 0.166 | -1.564 |
| 6 | 5.086 | -0.416 | -1.614 |
| 6 | 1.812 | 0.842 | -1.469 |
| 6 | 3.062 | 0.198 | -1.577 |
| 6 | 3.033 | -1.171 | -1.828 |
| 6 | 0.832 | -1.223 | -1.899 |
| 1 | -5.490 | 2.159 | -0.775 |
| 1 | -4.942 | 0.024 | -1.585 |
| 1 | -0.947 | 0.715 | -1.390 |
| 1 | 0.762 | 2.499 | -0.956 |
| 1 | 2.492 | 2.634 | -0.849 |
| 1 | 6.163 | -0.449 | -1.580 |
| 1 | 4.704 | -2.475 | -1.997 |
| 1 | -0.107 | -1.752 | -2.011 |
| 1 | -3.240 | 4.896 | -0.657 |
| 1 | -3.231 | 4.253 | 0.979 |
| 1 | -4.757 | 4.351 | 0.083 |
| 8 | -0.752 | -3.566 | -0.111 |
| 8 | -2.352 | 0.200 | 1.886 |
| 7 | -3.004 | -3.346 | 0.177 |
| 7 | -1.573 | -1.704 | 0.926 |
| 7 | 0.371 | 1.356 | 1.721 |
| 7 | 3.338 | 2.149 | 1.794 |
| 7 | 4.896 | 0.543 | 1.763 |
| 7 | 3.466 | -1.425 | 1.562 |
| 7 | 1.142 | -0.818 | 1.539 |
| 6 | -5.084 | -0.643 | 1.806 |
| 6 | -2.596 | -0.904 | 1.392 |
| 6 | -3.942 | -1.442 | 1.257 |
| 6 | -4.078 | -2.636 | 0.643 |
| 6 | -1.705 | -2.918 | 0.296 |
| 6 | 4.620 | 1.882 | 1.814 |
| 6 | 1.393 | 0.501 | 1.659 |
| 6 | 2.744 | 0.906 | 1.714 |
| 6 | 3.696 | -0.107 | 1.677 |
| 6 | 2.168 | -1.681 | 1.488 |
| 1 | -5.042 | -3.101 | 0.489 |
| 1 | -3.138 | -4.228 | -0.292 |
| 1 | -0.603 | -1.380 | 1.082 |
| 1 | -0.585 | 1.012 | 1.700 |
| 1 | 0.546 | 2.345 | 1.705 |
| 1 | 5.409 | 2.615 | 1.871 |
| 1 | 5.809 | 0.120 | 1.729 |
| 1 | 1.881 | -2.720 | 1.363 |
| 1 | -5.077 | 0.368 | 1.396 |
| 1 | -5.003 | -0.551 | 2.891 |
| 1 | -6.035 | -1.117 | 1.567 |

G-C/A-T

| 8 | 3.073 | -1.378 | 1.864 |
| --- | --- | --- | --- |
| 8 | 1.768 | 2.775 | 0.532 |
| 7 | 4.674 | 0.147 | 1.296 |
| 7 | 2.446 | 0.719 | 1.211 |
| 7 | -1.092 | 2.127 | 1.262 |
| 7 | -3.857 | 0.889 | 1.767 |
| 7 | -4.010 | -1.291 | 2.256 |
| 7 | -1.648 | -1.901 | 2.192 |
| 7 | -0.274 | -0.006 | 1.664 |
| 6 | 4.424 | 3.676 | -0.040 |
| 6 | 2.696 | 1.991 | 0.735 |
| 6 | 4.097 | 2.319 | 0.503 |
| 6 | 5.017 | 1.380 | 0.801 |
| 6 | 3.373 | -0.254 | 1.483 |
| 6 | -4.662 | -0.105 | 2.044 |
| 6 | -1.316 | 0.838 | 1.559 |
| 6 | -2.604 | 0.315 | 1.792 |
| 6 | -2.675 | -1.043 | 2.093 |
| 6 | -0.491 | -1.298 | 1.962 |
| 1 | 6.078 | 1.545 | 0.669 |
| 1 | 5.382 | -0.548 | 1.473 |
| 1 | 1.450 | 0.455 | 1.355 |
| 1 | -0.168 | 2.384 | 0.924 |
| 1 | -1.880 | 2.681 | 0.971 |
| 1 | -5.736 | -0.037 | 2.112 |
| 1 | -4.434 | -2.178 | 2.472 |
| 1 | 0.404 | -1.909 | 2.005 |
| 1 | 4.058 | 4.456 | 0.630 |
| 1 | 3.941 | 3.828 | -1.007 |
| 1 | 5.499 | 3.794 | -0.162 |
| 7 | -1.919 | -2.708 | -1.074 |
| 8 | -0.521 | 1.586 | -1.879 |
| 7 | -1.312 | -0.514 | -1.454 |
| 6 | -2.311 | -1.426 | -1.227 |
| 7 | -3.593 | -1.115 | -1.195 |
| 6 | -3.804 | 0.204 | -1.381 |
| 6 | -2.871 | 1.212 | -1.602 |
| 6 | -1.493 | 0.850 | -1.668 |
| 7 | -5.008 | 0.842 | -1.372 |
| 6 | -4.758 | 2.180 | -1.557 |
| 7 | -3.487 | 2.444 | -1.704 |
| 1 | -0.952 | -2.912 | -0.821 |
| 1 | -2.628 | -3.325 | -0.715 |
| 1 | -0.329 | -0.832 | -1.417 |
| 1 | -5.904 | 0.414 | -1.209 |
| 1 | -5.558 | 2.902 | -1.577 |
| 8 | 0.844 | -3.347 | -0.434 |
| 7 | 2.206 | 0.646 | -2.184 |
| 7 | 1.493 | -1.345 | -1.324 |
| 6 | 3.859 | -1.044 | -1.769 |
| 6 | 1.731 | -2.595 | -0.858 |
| 7 | 3.041 | -3.055 | -0.875 |
| 6 | 4.076 | -2.297 | -1.307 |
| 6 | 2.500 | -0.581 | -1.757 |
| 1 | 4.667 | -0.418 | -2.116 |
| 1 | 3.194 | -3.981 | -0.509 |
| 1 | 5.056 | -2.749 | -1.261 |
| 1 | 1.254 | 0.999 | -2.090 |
| 1 | 2.938 | 1.263 | -2.489 |

G-C/T-A

| 8 | -2.069 | -0.904 | -2.507 |
| --- | --- | --- | --- |
| 8 | -0.850 | 2.930 | -0.382 |
| 7 | -3.705 | 0.533 | -1.816 |
| 7 | -1.488 | 1.029 | -1.441 |
| 7 | 2.019 | 2.276 | -0.842 |
| 7 | 4.811 | 0.994 | -0.959 |
| 7 | 5.008 | -1.133 | -1.628 |
| 7 | 2.669 | -1.645 | -2.102 |
| 7 | 1.255 | 0.235 | -1.627 |
| 6 | -3.539 | 3.763 | 0.143 |
| 6 | -1.767 | 2.210 | -0.785 |
| 6 | -3.177 | 2.529 | -0.623 |
| 6 | -4.077 | 1.672 | -1.146 |
| 6 | -2.397 | 0.140 | -1.959 |
| 6 | 5.634 | 0.001 | -1.180 |
| 6 | 2.273 | 1.023 | -1.236 |
| 6 | 3.573 | 0.476 | -1.282 |
| 6 | 3.673 | -0.847 | -1.706 |
| 6 | 1.503 | -1.022 | -2.029 |
| 1 | -5.143 | 1.829 | -1.063 |
| 1 | -4.402 | -0.106 | -2.164 |
| 1 | -0.490 | 0.756 | -1.529 |
| 1 | 1.056 | 2.553 | -0.670 |
| 1 | 2.768 | 2.807 | -0.432 |
| 1 | 6.703 | 0.031 | -1.038 |
| 1 | 5.449 | -2.007 | -1.866 |
| 1 | 0.622 | -1.582 | -2.324 |
| 1 | -3.085 | 4.647 | -0.309 |
| 1 | -3.165 | 3.689 | 1.166 |
| 1 | -4.619 | 3.897 | 0.173 |
| 7 | -0.044 | -3.081 | 0.226 |
| 8 | -1.847 | 0.762 | 1.959 |
| 7 | -1.040 | -1.185 | 1.076 |
| 6 | -1.180 | -2.417 | 0.488 |
| 7 | -2.347 | -2.948 | 0.171 |
| 6 | -3.378 | -2.137 | 0.478 |
| 6 | -3.353 | -0.888 | 1.093 |
| 6 | -2.083 | -0.332 | 1.430 |
| 7 | -4.699 | -2.382 | 0.248 |
| 6 | -5.402 | -1.301 | 0.727 |
| 7 | -4.631 | -0.383 | 1.243 |
| 1 | 0.860 | -2.766 | 0.578 |
| 1 | -0.139 | -4.010 | -0.143 |
| 1 | -0.090 | -0.844 | 1.298 |
| 1 | -5.084 | -3.207 | -0.184 |
| 1 | -6.477 | -1.259 | 0.664 |
| 8 | 2.517 | -2.232 | 1.239 |
| 7 | 0.821 | 1.861 | 2.347 |
| 7 | 1.635 | -0.198 | 1.793 |
| 6 | 3.201 | 1.563 | 2.381 |
| 6 | 2.665 | -1.056 | 1.598 |
| 7 | 3.954 | -0.588 | 1.818 |
| 6 | 4.215 | 0.691 | 2.185 |
| 6 | 1.870 | 1.064 | 2.168 |
| 1 | 3.380 | 2.585 | 2.676 |
| 1 | 4.702 | -1.245 | 1.655 |
| 1 | 5.256 | 0.954 | 2.303 |
| 1 | -0.119 | 1.516 | 2.160 |
| 1 | 0.956 | 2.829 | 2.579 |

G-C/G-C

| 7 | -0.571 | -0.874 | -2.284 |
| --- | --- | --- | --- |
| 8 | 1.269 | 3.048 | -0.771 |
| 7 | 0.448 | 1.061 | -1.544 |
| 6 | 0.576 | -0.229 | -1.997 |
| 7 | 1.739 | -0.825 | -2.179 |
| 6 | 2.780 | -0.031 | -1.855 |
| 6 | 2.765 | 1.286 | -1.406 |
| 6 | 1.499 | 1.911 | -1.202 |
| 7 | 4.100 | -0.367 | -1.898 |
| 6 | 4.810 | 0.729 | -1.470 |
| 7 | 4.047 | 1.743 | -1.167 |
| 1 | -1.462 | -0.464 | -2.009 |
| 1 | -0.528 | -1.882 | -2.261 |
| 1 | -0.497 | 1.411 | -1.330 |
| 1 | 4.473 | -1.269 | -2.145 |
| 1 | 5.886 | 0.710 | -1.407 |
| 8 | -3.213 | 0.154 | -1.647 |
| 7 | -1.295 | 3.774 | 0.374 |
| 7 | -2.227 | 1.973 | -0.676 |
| 6 | -3.607 | 3.315 | 0.806 |
| 6 | -3.278 | 1.154 | -0.921 |
| 7 | -4.493 | 1.455 | -0.318 |
| 6 | -4.652 | 2.503 | 0.528 |
| 6 | -2.361 | 3.009 | 0.157 |
| 1 | -3.704 | 4.153 | 1.477 |
| 1 | -5.256 | 0.826 | -0.518 |
| 1 | -5.637 | 2.637 | 0.951 |
| 1 | -0.400 | 3.540 | -0.056 |
| 1 | -1.351 | 4.551 | 1.008 |
| 7 | 1.311 | -3.341 | 0.090 |
| 8 | 0.300 | 0.725 | 1.950 |
| 7 | 0.907 | -1.267 | 1.014 |
| 6 | 1.820 | -2.217 | 0.632 |
| 7 | 3.121 | -2.094 | 0.809 |
| 6 | 3.448 | -0.933 | 1.413 |
| 6 | 2.610 | 0.087 | 1.854 |
| 6 | 1.206 | -0.061 | 1.644 |
| 7 | 4.704 | -0.510 | 1.733 |
| 6 | 4.576 | 0.719 | 2.335 |
| 7 | 3.335 | 1.112 | 2.429 |
| 1 | 0.360 | -3.352 | -0.274 |
| 1 | 1.984 | -3.950 | -0.344 |
| 1 | -0.094 | -1.442 | 0.828 |
| 1 | 5.562 | -1.005 | 1.551 |
| 1 | 5.438 | 1.267 | 2.680 |
| 8 | -1.425 | -3.429 | -0.940 |
| 7 | -2.509 | -0.094 | 1.984 |
| 7 | -1.930 | -1.788 | 0.568 |
| 6 | -4.266 | -1.505 | 1.162 |
| 6 | -2.259 | -2.797 | -0.268 |
| 7 | -3.595 | -3.144 | -0.379 |
| 6 | -4.573 | -2.522 | 0.326 |
| 6 | -2.882 | -1.130 | 1.236 |
| 1 | -5.022 | -0.990 | 1.734 |
| 1 | -3.812 | -3.901 | -1.008 |
| 1 | -5.579 | -2.885 | 0.176 |
| 1 | -1.534 | 0.208 | 1.985 |
| 1 | -3.191 | 0.433 | 2.500 |

C^+^•G-C

| 7 | -2.712 | -0.037 | 0.000 |
| --- | --- | --- | --- |
| 8 | 1.664 | 1.330 | 0.000 |
| 7 | -0.540 | 0.732 | 0.000 |
| 6 | -1.888 | 1.014 | 0.000 |
| 7 | -2.379 | 2.244 | 0.000 |
| 6 | -1.419 | 3.182 | 0.000 |
| 6 | -0.042 | 3.001 | 0.000 |
| 6 | 0.468 | 1.678 | 0.000 |
| 7 | -1.591 | 4.539 | 0.000 |
| 6 | -0.350 | 5.114 | 0.000 |
| 7 | 0.604 | 4.220 | 0.000 |
| 1 | -2.375 | -1.001 | 0.000 |
| 1 | -3.697 | 0.156 | 0.000 |
| 1 | -0.236 | -0.259 | 0.000 |
| 1 | -2.473 | 5.026 | 0.000 |
| 1 | -0.196 | 6.180 | 0.000 |
| 8 | -1.814 | -2.741 | 0.000 |
| 7 | 2.572 | -1.459 | 0.000 |
| 7 | 0.369 | -2.062 | 0.000 |
| 6 | 2.065 | -3.801 | 0.000 |
| 6 | -0.605 | -3.005 | 0.000 |
| 7 | -0.220 | -4.338 | 0.000 |
| 6 | 1.080 | -4.726 | 0.000 |
| 6 | 1.656 | -2.424 | 0.000 |
| 1 | 3.106 | -4.081 | 0.000 |
| 1 | -0.964 | -5.019 | 0.000 |
| 1 | 1.264 | -5.790 | 0.000 |
| 1 | 2.282 | -0.485 | 0.000 |
| 1 | 3.550 | -1.686 | 0.000 |
| 6 | 5.587 | 4.735 | 0.000 |
| 6 | 5.679 | 6.085 | 0.000 |
| 7 | 4.574 | 6.874 | 0.000 |
| 6 | 3.289 | 6.374 | 0.000 |
| 8 | 2.299 | 7.080 | 0.000 |
| 7 | 3.216 | 4.995 | 0.000 |
| 1 | 2.218 | 4.621 | 0.000 |
| 6 | 4.279 | 4.163 | 0.000 |
| 7 | 4.070 | 2.864 | 0.000 |
| 1 | 6.464 | 4.109 | 0.000 |
| 1 | 6.627 | 6.602 | 0.000 |
| 1 | 3.136 | 2.444 | 0.000 |
| 1 | 4.863 | 2.243 | 0.000 |
| 1 | 4.659 | 7.880 | 0.000 |

T•A-T

| 8 | -0.331 | 5.093 | 0.009 |
| --- | --- | --- | --- |
| 8 | -4.711 | 6.322 | 0.022 |
| 8 | 2.153 | 0.725 | -0.012 |
| 8 | 0.074 | -3.323 | -0.017 |
| 7 | -2.504 | 5.739 | 0.015 |
| 7 | -3.114 | 7.957 | 0.024 |
| 7 | -1.415 | -0.026 | -0.004 |
| 7 | -3.767 | -0.507 | 0.002 |
| 7 | -5.091 | 1.545 | 0.011 |
| 7 | -3.456 | 3.053 | 0.009 |
| 7 | -0.515 | 2.108 | -0.002 |
| 7 | 1.144 | -1.306 | -0.015 |
| 7 | 2.357 | -3.262 | -0.025 |
| 6 | -1.152 | 6.013 | 0.013 |
| 6 | -3.532 | 6.649 | 0.021 |
| 6 | -1.792 | 8.324 | 0.022 |
| 6 | -0.789 | 7.423 | 0.017 |
| 6 | 0.665 | 7.781 | 0.015 |
| 6 | -2.484 | -0.836 | -0.003 |
| 6 | -3.924 | 0.827 | 0.006 |
| 6 | -2.916 | 1.785 | 0.005 |
| 6 | -1.583 | 1.313 | -0.001 |
| 6 | -4.752 | 2.866 | 0.013 |
| 6 | 1.113 | -2.678 | -0.019 |
| 6 | 3.526 | -2.544 | -0.026 |
| 6 | 3.546 | -1.196 | -0.023 |
| 6 | 2.266 | -0.503 | -0.016 |
| 6 | 4.797 | -0.372 | -0.024 |
| 1 | 1.164 | 7.365 | 0.891 |
| 1 | 1.160 | 7.371 | -0.867 |
| 1 | 0.795 | 8.863 | 0.018 |
| 1 | -1.610 | 9.390 | 0.025 |
| 1 | -2.790 | 4.745 | 0.013 |
| 1 | -3.839 | 8.658 | 0.028 |
| 1 | 4.834 | 0.271 | 0.856 |
| 1 | 5.679 | -1.011 | -0.029 |
| 1 | 4.828 | 0.276 | -0.902 |
| 1 | -2.245 | -1.895 | -0.006 |
| 1 | -6.025 | 1.168 | 0.013 |
| 1 | -5.484 | 3.658 | 0.017 |
| 1 | -0.606 | 3.118 | 0.000 |
| 1 | 0.408 | 1.684 | -0.006 |
| 1 | 0.219 | -0.832 | -0.011 |
| 1 | 2.375 | -4.270 | -0.028 |
| 1 | 4.430 | -3.138 | -0.031 |

rT•A-T

| 8 | 0.136 | -3.382 | 0.000 |
| --- | --- | --- | --- |
| 8 | 2.161 | 0.688 | 0.000 |
| 7 | 2.418 | -3.299 | 0.000 |
| 7 | 1.189 | -1.356 | 0.000 |
| 7 | -0.519 | 2.038 | 0.000 |
| 7 | -3.474 | 2.936 | 0.000 |
| 7 | -5.085 | 1.403 | 0.000 |
| 7 | -3.729 | -0.627 | 0.000 |
| 7 | -1.385 | -0.109 | 0.000 |
| 6 | 4.853 | -0.401 | 0.000 |
| 6 | 2.302 | -0.537 | 0.000 |
| 6 | 3.591 | -1.218 | 0.000 |
| 6 | 3.578 | -2.568 | 0.000 |
| 6 | 1.169 | -2.727 | 0.000 |
| 6 | -4.767 | 2.730 | 0.000 |
| 6 | -1.574 | 1.227 | 0.000 |
| 6 | -2.915 | 1.677 | 0.000 |
| 6 | -3.907 | 0.703 | 0.000 |
| 6 | -2.441 | -0.937 | 0.000 |
| 1 | 4.487 | -3.153 | 0.000 |
| 1 | 2.448 | -4.306 | 0.000 |
| 1 | 0.260 | -0.890 | 0.000 |
| 1 | 0.410 | 1.628 | 0.000 |
| 1 | -0.625 | 3.046 | 0.000 |
| 1 | -5.512 | 3.510 | 0.000 |
| 1 | -6.014 | 1.011 | 0.000 |
| 1 | -2.185 | -1.991 | 0.000 |
| 1 | 4.592 | 0.655 | 0.000 |
| 1 | 5.459 | -0.606 | -0.883 |
| 1 | 5.459 | -0.606 | 0.883 |
| 6 | -4.305 | 9.002 | 0.000 |
| 6 | -3.229 | 7.960 | 0.000 |
| 6 | -1.910 | 8.236 | 0.000 |
| 7 | -0.951 | 7.251 | 0.000 |
| 1 | 0.028 | 7.489 | 0.000 |
| 6 | -1.252 | 5.915 | 0.000 |
| 8 | -0.388 | 5.041 | 0.000 |
| 7 | -2.591 | 5.641 | 0.000 |
| 1 | -2.856 | 4.641 | 0.000 |
| 6 | -3.631 | 6.558 | 0.000 |
| 8 | -4.795 | 6.169 | 0.000 |
| 1 | -1.529 | 9.247 | 0.000 |
| 1 | -3.875 | 10.002 | 0.000 |
| 1 | -4.943 | 8.893 | 0.879 |
| 1 | -4.943 | 8.893 | -0.879 |

rA•A-T

| 8 | 0.140 | -3.308 | 0.000 |
| --- | --- | --- | --- |
| 8 | 2.020 | 0.832 | 0.000 |
| 7 | 2.418 | -3.144 | 0.000 |
| 7 | 1.119 | -1.245 | 0.000 |
| 7 | -0.704 | 2.066 | 0.000 |
| 7 | -3.681 | 2.894 | 0.000 |
| 7 | -5.247 | 1.305 | 0.000 |
| 7 | -3.836 | -0.684 | 0.000 |
| 7 | -1.508 | -0.102 | 0.000 |
| 6 | 4.747 | -0.160 | 0.000 |
| 6 | 2.202 | -0.388 | 0.000 |
| 6 | 3.515 | -1.022 | 0.000 |
| 6 | 3.550 | -2.371 | 0.000 |
| 6 | 1.149 | -2.617 | 0.000 |
| 6 | -4.966 | 2.641 | 0.000 |
| 6 | -1.736 | 1.228 | 0.000 |
| 6 | -3.088 | 1.647 | 0.000 |
| 6 | -4.050 | 0.642 | 0.000 |
| 6 | -2.540 | -0.959 | 0.000 |
| 1 | 4.480 | -2.923 | 0.000 |
| 1 | 2.484 | -4.149 | 0.000 |
| 1 | 0.172 | -0.816 | 0.000 |
| 1 | 0.236 | 1.681 | 0.000 |
| 1 | -0.837 | 3.073 | 0.000 |
| 1 | -5.748 | 3.383 | 0.000 |
| 1 | -6.164 | 0.886 | 0.000 |
| 1 | -2.256 | -2.006 | 0.000 |
| 7 | -2.557 | 8.081 | 0.000 |
| 6 | -2.330 | 6.754 | 0.000 |
| 6 | -0.988 | 6.311 | 0.000 |
| 6 | -0.011 | 7.301 | 0.000 |
| 7 | -0.211 | 8.627 | 0.000 |
| 6 | -1.508 | 8.913 | 0.000 |
| 7 | 1.178 | 6.621 | 0.000 |
| 6 | 0.876 | 5.289 | 0.000 |
| 7 | -0.413 | 5.055 | 0.000 |
| 7 | -3.369 | 5.915 | 0.000 |
| 1 | -1.760 | 9.969 | 0.000 |
| 1 | 1.646 | 4.534 | 0.000 |
| 1 | -4.293 | 6.311 | 0.000 |
| 1 | -3.258 | 4.905 | 0.000 |
| 1 | 2.101 | 7.025 | 0.000 |
| 1 | 4.448 | 0.886 | 0.000 |
| 1 | 5.360 | -0.344 | -0.883 |
| 1 | 5.360 | -0.344 | 0.883 |

rG•G-C

| 7 | -2.631 | 0.021 | 0.000 |
| --- | --- | --- | --- |
| 8 | 1.765 | 1.328 | 0.000 |
| 7 | -0.449 | 0.760 | 0.000 |
| 6 | -1.793 | 1.062 | 0.000 |
| 7 | -2.266 | 2.298 | 0.000 |
| 6 | -1.291 | 3.224 | 0.000 |
| 6 | 0.083 | 3.024 | 0.000 |
| 6 | 0.576 | 1.692 | 0.000 |
| 7 | -1.442 | 4.582 | 0.000 |
| 6 | -0.188 | 5.136 | 0.000 |
| 7 | 0.751 | 4.229 | 0.000 |
| 1 | -2.305 | -0.946 | 0.000 |
| 1 | -3.614 | 0.226 | 0.000 |
| 1 | -0.159 | -0.233 | 0.000 |
| 1 | -2.317 | 5.082 | 0.000 |
| 1 | -0.016 | 6.200 | 0.000 |
| 8 | -1.752 | -2.702 | 0.000 |
| 7 | 2.644 | -1.456 | 0.000 |
| 7 | 0.437 | -2.042 | 0.000 |
| 6 | 2.119 | -3.794 | 0.000 |
| 6 | -0.545 | -2.976 | 0.000 |
| 7 | -0.171 | -4.313 | 0.000 |
| 6 | 1.126 | -4.711 | 0.000 |
| 6 | 1.721 | -2.414 | 0.000 |
| 1 | 3.158 | -4.082 | 0.000 |
| 1 | -0.920 | -4.988 | 0.000 |
| 1 | 1.302 | -5.777 | 0.000 |
| 1 | 2.363 | -0.479 | 0.000 |
| 1 | 3.620 | -1.694 | 0.000 |
| 6 | 6.726 | 8.038 | 0.000 |
| 7 | 7.070 | 6.707 | 0.000 |
| 1 | 8.002 | 6.325 | 0.000 |
| 6 | 5.908 | 5.992 | 0.000 |
| 6 | 4.902 | 6.952 | 0.000 |
| 7 | 5.435 | 8.227 | 0.000 |
| 6 | 3.541 | 6.506 | 0.000 |
| 8 | 2.508 | 7.175 | 0.000 |
| 7 | 3.473 | 5.108 | 0.000 |
| 1 | 2.514 | 4.724 | 0.000 |
| 6 | 4.538 | 4.249 | 0.000 |
| 7 | 5.798 | 4.646 | 0.000 |
| 7 | 4.252 | 2.937 | 0.000 |
| 1 | 7.478 | 8.811 | 0.000 |
| 1 | 3.309 | 2.563 | 0.000 |
| 1 | 5.029 | 2.301 | 0.000 |

C^+^•G-C/C^+^•G-C

| 7 | -1.892 | -3.050 | 2.020 |
| --- | --- | --- | --- |
| 8 | -0.026 | 1.115 | 1.573 |
| 7 | -0.876 | -0.994 | 1.795 |
| 6 | -0.752 | -2.358 | 1.943 |
| 7 | 0.412 | -2.984 | 2.026 |
| 6 | 1.454 | -2.144 | 1.940 |
| 6 | 1.435 | -0.766 | 1.777 |
| 6 | 0.181 | -0.107 | 1.700 |
| 7 | 2.780 | -2.473 | 1.988 |
| 6 | 3.498 | -1.319 | 1.853 |
| 7 | 2.722 | -0.273 | 1.726 |
| 1 | -2.805 | -2.623 | 1.848 |
| 1 | -1.808 | -4.051 | 2.032 |
| 1 | -1.828 | -0.584 | 1.743 |
| 1 | 3.161 | -3.402 | 2.073 |
| 1 | 4.575 | -1.292 | 1.853 |
| 8 | -4.460 | -1.928 | 1.586 |
| 7 | -2.718 | 2.296 | 1.724 |
| 7 | -3.551 | 0.169 | 1.658 |
| 6 | -5.095 | 2.041 | 1.589 |
| 6 | -4.592 | -0.696 | 1.594 |
| 7 | -5.875 | -0.171 | 1.547 |
| 6 | -6.120 | 1.160 | 1.529 |
| 6 | -3.773 | 1.487 | 1.655 |
| 1 | -5.260 | 3.107 | 1.578 |
| 1 | -6.628 | -0.838 | 1.478 |
| 1 | -7.156 | 1.459 | 1.468 |
| 1 | -1.778 | 1.911 | 1.677 |
| 1 | -2.842 | 3.292 | 1.666 |
| 6 | 3.800 | 4.403 | 0.373 |
| 6 | 5.152 | 4.331 | 0.362 |
| 7 | 5.807 | 3.188 | 0.694 |
| 6 | 5.161 | 2.028 | 1.063 |
| 8 | 5.746 | 0.997 | 1.342 |
| 7 | 3.784 | 2.125 | 1.079 |
| 1 | 3.295 | 1.215 | 1.366 |
| 6 | 3.080 | 3.231 | 0.754 |
| 7 | 1.766 | 3.182 | 0.788 |
| 1 | 3.280 | 5.309 | 0.107 |
| 1 | 5.776 | 5.169 | 0.089 |
| 1 | 1.239 | 2.352 | 1.084 |
| 1 | 1.243 | 4.012 | 0.560 |
| 1 | 6.816 | 3.150 | 0.678 |
| 7 | -4.021 | -2.377 | -1.575 |
| 8 | -0.169 | 0.096 | -1.421 |
| 7 | -2.043 | -1.205 | -1.435 |
| 6 | -2.694 | -2.419 | -1.427 |
| 7 | -2.069 | -3.582 | -1.318 |
| 6 | -0.736 | -3.448 | -1.236 |
| 6 | 0.012 | -2.279 | -1.276 |
| 6 | -0.673 | -1.043 | -1.385 |
| 7 | 0.189 | -4.446 | -1.105 |
| 6 | 1.425 | -3.862 | -1.068 |
| 7 | 1.358 | -2.561 | -1.175 |
| 1 | -4.529 | -1.493 | -1.532 |
| 1 | -4.516 | -3.236 | -1.412 |
| 1 | -2.595 | -0.329 | -1.480 |
| 1 | -0.006 | -5.432 | -1.036 |
| 1 | 2.336 | -4.428 | -0.963 |
| 8 | -5.432 | 0.124 | -1.605 |
| 7 | -1.618 | 2.630 | -1.417 |
| 7 | -3.501 | 1.345 | -1.543 |
| 6 | -3.723 | 3.762 | -1.601 |
| 6 | -4.847 | 1.215 | -1.631 |
| 7 | -5.606 | 2.370 | -1.759 |
| 6 | -5.060 | 3.610 | -1.730 |
| 6 | -2.942 | 2.559 | -1.520 |
| 1 | -3.261 | 4.737 | -1.577 |
| 1 | -6.604 | 2.243 | -1.829 |
| 1 | -5.747 | 4.440 | -1.813 |
| 1 | -1.074 | 1.774 | -1.349 |
| 1 | -1.156 | 3.522 | -1.393 |
| 6 | 4.869 | 0.927 | -2.013 |
| 6 | 5.989 | 0.195 | -1.802 |
| 7 | 5.925 | -1.097 | -1.393 |
| 6 | 4.739 | -1.763 | -1.171 |
| 8 | 4.683 | -2.923 | -0.808 |
| 7 | 3.609 | -1.000 | -1.388 |
| 1 | 2.700 | -1.538 | -1.263 |
| 6 | 3.613 | 0.296 | -1.766 |
| 7 | 2.465 | 0.929 | -1.880 |
| 1 | 4.916 | 1.955 | -2.337 |
| 1 | 6.981 | 0.598 | -1.944 |
| 1 | 1.557 | 0.490 | -1.683 |
| 1 | 2.465 | 1.882 | -2.206 |
| 1 | 6.769 | -1.627 | -1.232 |

C^+^•G-C/T•A-T

| 8 | 5.010 | -2.858 | -0.387 |
| --- | --- | --- | --- |
| 8 | 3.654 | 1.341 | -1.511 |
| 7 | 6.582 | -1.227 | -0.654 |
| 7 | 4.358 | -0.754 | -0.998 |
| 7 | 0.760 | 0.517 | -1.211 |
| 7 | -1.968 | -0.828 | -1.759 |
| 7 | -2.051 | -3.015 | -2.149 |
| 7 | 0.318 | -3.568 | -1.921 |
| 7 | 1.627 | -1.618 | -1.425 |
| 6 | 6.322 | 2.371 | -1.781 |
| 6 | 4.595 | 0.574 | -1.296 |
| 6 | 5.994 | 0.978 | -1.342 |
| 6 | 6.919 | 0.057 | -1.003 |
| 6 | 5.294 | -1.695 | -0.661 |
| 6 | -2.744 | -1.848 | -2.032 |
| 6 | 0.568 | -0.782 | -1.437 |
| 6 | -0.699 | -1.358 | -1.688 |
| 6 | -0.730 | -2.728 | -1.923 |
| 6 | 1.446 | -2.927 | -1.657 |
| 1 | 7.978 | 0.274 | -0.995 |
| 1 | 7.295 | -1.894 | -0.406 |
| 1 | 3.374 | -1.074 | -1.053 |
| 1 | 1.713 | 0.861 | -1.148 |
| 1 | -0.017 | 1.165 | -1.160 |
| 1 | -3.815 | -1.793 | -2.143 |
| 1 | -2.441 | -3.923 | -2.347 |
| 1 | 2.350 | -3.524 | -1.610 |
| 1 | 6.037 | 2.519 | -2.825 |
| 1 | 7.389 | 2.566 | -1.681 |
| 1 | 5.770 | 3.103 | -1.192 |
| 6 | -2.615 | 5.216 | -0.252 |
| 6 | -3.329 | 3.974 | -0.688 |
| 6 | -4.661 | 3.898 | -0.895 |
| 7 | -5.278 | 2.739 | -1.293 |
| 1 | -6.278 | 2.709 | -1.416 |
| 6 | -4.607 | 1.558 | -1.486 |
| 8 | -5.167 | 0.510 | -1.787 |
| 7 | -3.251 | 1.652 | -1.301 |
| 1 | -2.716 | 0.775 | -1.458 |
| 6 | -2.545 | 2.771 | -0.917 |
| 8 | -1.318 | 2.714 | -0.788 |
| 1 | -5.318 | 4.746 | -0.759 |
| 1 | -3.314 | 6.044 | -0.142 |
| 1 | -1.852 | 5.494 | -0.980 |
| 1 | -2.112 | 5.055 | 0.703 |
| 7 | 3.327 | -1.982 | 1.913 |
| 8 | -0.653 | 0.294 | 1.793 |
| 7 | 1.282 | -0.916 | 1.855 |
| 6 | 1.999 | -2.087 | 1.756 |
| 7 | 1.445 | -3.270 | 1.549 |
| 6 | 0.111 | -3.199 | 1.416 |
| 6 | -0.700 | -2.076 | 1.511 |
| 6 | -0.090 | -0.816 | 1.731 |
| 7 | -0.749 | -4.222 | 1.128 |
| 6 | -2.006 | -3.692 | 1.035 |
| 7 | -2.015 | -2.404 | 1.260 |
| 1 | 3.769 | -1.062 | 1.947 |
| 1 | 3.872 | -2.732 | 1.515 |
| 1 | 1.801 | -0.021 | 1.884 |
| 1 | -0.493 | -5.182 | 0.961 |
| 1 | -2.871 | -4.286 | 0.792 |
| 8 | 4.719 | 0.530 | 1.977 |
| 7 | 0.812 | 2.745 | 1.138 |
| 7 | 2.741 | 1.619 | 1.620 |
| 6 | 2.877 | 3.937 | 0.900 |
| 6 | 4.092 | 1.559 | 1.689 |
| 7 | 4.811 | 2.714 | 1.418 |
| 6 | 4.220 | 3.871 | 1.027 |
| 6 | 2.140 | 2.745 | 1.219 |
| 1 | 2.377 | 4.842 | 0.593 |
| 1 | 5.815 | 2.641 | 1.490 |
| 1 | 4.877 | 4.707 | 0.832 |
| 1 | 0.301 | 1.885 | 1.319 |
| 1 | 0.318 | 3.481 | 0.663 |
| 6 | -5.740 | 0.938 | 1.565 |
| 6 | -6.755 | 0.210 | 1.040 |
| 7 | -6.549 | -1.023 | 0.517 |
| 6 | -5.308 | -1.619 | 0.457 |
| 8 | -5.120 | -2.720 | -0.026 |
| 7 | -4.287 | -0.871 | 1.011 |
| 1 | -3.363 | -1.388 | 1.068 |
| 6 | -4.435 | 0.362 | 1.542 |
| 7 | -3.375 | 0.984 | 2.012 |
| 1 | -5.902 | 1.921 | 1.975 |
| 1 | -7.773 | 0.572 | 1.012 |
| 1 | -2.424 | 0.607 | 1.925 |
| 1 | -3.487 | 1.899 | 2.415 |
| 1 | -7.311 | -1.544 | 0.109 |

T•A-T/T•A-T

| 8 | -4.738 | -3.075 | 0.796 |
| --- | --- | --- | --- |
| 8 | -3.189 | 1.074 | 1.855 |
| 7 | -6.243 | -1.389 | 1.111 |
| 7 | -3.986 | -0.987 | 1.336 |
| 7 | -0.382 | 0.069 | 1.737 |
| 7 | 2.329 | -1.395 | 1.550 |
| 7 | 2.371 | -3.616 | 1.449 |
| 7 | -0.028 | -4.079 | 1.502 |
| 7 | -1.307 | -2.051 | 1.626 |
| 6 | -5.789 | 2.268 | 1.957 |
| 6 | -4.162 | 0.350 | 1.627 |
| 6 | -5.538 | 0.825 | 1.640 |
| 6 | -6.511 | -0.072 | 1.373 |
| 6 | -4.971 | -1.902 | 1.057 |
| 6 | 3.091 | -2.457 | 1.466 |
| 6 | -0.221 | -1.250 | 1.647 |
| 6 | 1.042 | -1.883 | 1.582 |
| 6 | 1.045 | -3.271 | 1.515 |
| 6 | -1.153 | -3.381 | 1.552 |
| 1 | -7.558 | 0.199 | 1.360 |
| 1 | -6.988 | -2.026 | 0.882 |
| 1 | -3.020 | -1.359 | 1.376 |
| 1 | -1.325 | 0.446 | 1.730 |
| 1 | 0.407 | 0.699 | 1.641 |
| 1 | 4.166 | -2.441 | 1.397 |
| 1 | 2.743 | -4.549 | 1.383 |
| 1 | -2.081 | -3.942 | 1.521 |
| 1 | -5.472 | 2.498 | 2.976 |
| 1 | -6.847 | 2.505 | 1.858 |
| 1 | -5.219 | 2.912 | 1.286 |
| 6 | 2.861 | 4.844 | 1.594 |
| 6 | 3.616 | 3.550 | 1.622 |
| 6 | 4.959 | 3.459 | 1.716 |
| 7 | 5.617 | 2.255 | 1.729 |
| 1 | 6.623 | 2.221 | 1.781 |
| 6 | 4.972 | 1.045 | 1.640 |
| 8 | 5.562 | -0.028 | 1.632 |
| 7 | 3.606 | 1.147 | 1.563 |
| 1 | 3.092 | 0.248 | 1.526 |
| 6 | 2.860 | 2.308 | 1.546 |
| 8 | 1.631 | 2.256 | 1.482 |
| 1 | 5.595 | 4.331 | 1.782 |
| 1 | 3.544 | 5.690 | 1.662 |
| 1 | 2.154 | 4.893 | 2.424 |
| 1 | 2.286 | 4.930 | 0.671 |
| 8 | -5.408 | -0.330 | -1.712 |
| 8 | -2.023 | 2.667 | -1.194 |
| 7 | -5.911 | 1.898 | -1.675 |
| 7 | -3.730 | 1.196 | -1.451 |
| 7 | -0.001 | 0.467 | -1.553 |
| 7 | 1.641 | -2.141 | -1.675 |
| 7 | 0.586 | -4.100 | -1.738 |
| 7 | -1.729 | -3.330 | -1.650 |
| 7 | -1.846 | -0.935 | -1.532 |
| 6 | -3.734 | 4.964 | -1.263 |
| 6 | -3.233 | 2.478 | -1.332 |
| 6 | -4.216 | 3.551 | -1.393 |
| 6 | -5.508 | 3.205 | -1.567 |
| 6 | -5.042 | 0.834 | -1.619 |
| 6 | 1.781 | -3.442 | -1.720 |
| 6 | -0.509 | -0.764 | -1.577 |
| 6 | 0.278 | -1.936 | -1.653 |
| 6 | -0.399 | -3.150 | -1.683 |
| 6 | -2.367 | -2.171 | -1.568 |
| 1 | -6.301 | 3.938 | -1.627 |
| 1 | -6.886 | 1.670 | -1.792 |
| 1 | -3.038 | 0.417 | -1.437 |
| 1 | -0.635 | 1.247 | -1.415 |
| 1 | 1.001 | 0.625 | -1.556 |
| 1 | 2.729 | -3.957 | -1.733 |
| 1 | 0.453 | -5.098 | -1.741 |
| 1 | -3.450 | -2.208 | -1.520 |
| 1 | -3.236 | 5.111 | -0.303 |
| 1 | -4.565 | 5.663 | -1.337 |
| 1 | -3.009 | 5.195 | -2.045 |
| 6 | 5.210 | 2.986 | -1.906 |
| 6 | 5.218 | 1.507 | -1.667 |
| 6 | 6.340 | 0.768 | -1.527 |
| 7 | 6.310 | -0.588 | -1.338 |
| 1 | 7.164 | -1.104 | -1.199 |
| 6 | 5.146 | -1.307 | -1.238 |
| 8 | 5.120 | -2.516 | -1.046 |
| 7 | 4.011 | -0.551 | -1.388 |
| 1 | 3.121 | -1.077 | -1.398 |
| 6 | 3.942 | 0.810 | -1.599 |
| 8 | 2.848 | 1.368 | -1.716 |
| 1 | 7.327 | 1.207 | -1.564 |
| 1 | 6.223 | 3.386 | -1.869 |
| 1 | 4.603 | 3.492 | -1.154 |
| 1 | 4.779 | 3.215 | -2.882 |

rT•A-T/rT•A-T

| 8 | -4.710 | -3.147 | 0.883 |
| --- | --- | --- | --- |
| 8 | -3.133 | 0.998 | 1.906 |
| 7 | -6.206 | -1.500 | 1.393 |
| 7 | -3.945 | -1.064 | 1.421 |
| 7 | -0.317 | 0.016 | 1.689 |
| 7 | 2.388 | -1.430 | 1.364 |
| 7 | 2.429 | -3.637 | 1.104 |
| 7 | 0.034 | -4.111 | 1.203 |
| 7 | -1.243 | -2.098 | 1.495 |
| 6 | -5.731 | 2.147 | 2.270 |
| 6 | -4.113 | 0.264 | 1.753 |
| 6 | -5.489 | 0.715 | 1.902 |
| 6 | -6.468 | -0.193 | 1.708 |
| 6 | -4.937 | -1.988 | 1.203 |
| 6 | 3.149 | -2.481 | 1.187 |
| 6 | -0.159 | -1.297 | 1.527 |
| 6 | 1.102 | -1.923 | 1.394 |
| 6 | 1.105 | -3.302 | 1.233 |
| 6 | -1.090 | -3.421 | 1.336 |
| 1 | -7.516 | 0.059 | 1.799 |
| 1 | -6.959 | -2.143 | 1.209 |
| 1 | -2.973 | -1.420 | 1.370 |
| 1 | -1.260 | 0.390 | 1.721 |
| 1 | 0.468 | 0.651 | 1.606 |
| 1 | 4.223 | -2.451 | 1.098 |
| 1 | 2.802 | -4.564 | 0.973 |
| 1 | -2.018 | -3.982 | 1.304 |
| 1 | -5.254 | 2.814 | 1.551 |
| 1 | -5.308 | 2.368 | 3.252 |
| 1 | -6.799 | 2.362 | 2.292 |
| 6 | 7.281 | 2.187 | 1.838 |
| 6 | 5.789 | 2.232 | 1.720 |
| 6 | 5.067 | 3.370 | 1.670 |
| 7 | 3.696 | 3.371 | 1.567 |
| 1 | 3.185 | 4.239 | 1.559 |
| 6 | 2.948 | 2.222 | 1.529 |
| 8 | 1.721 | 2.231 | 1.484 |
| 7 | 3.688 | 1.073 | 1.545 |
| 1 | 3.157 | 0.182 | 1.493 |
| 6 | 5.066 | 0.966 | 1.655 |
| 8 | 5.597 | -0.141 | 1.685 |
| 1 | 5.525 | 4.349 | 1.707 |
| 1 | 7.699 | 3.193 | 1.851 |
| 1 | 7.712 | 1.635 | 1.001 |
| 1 | 7.578 | 1.670 | 2.753 |
| 8 | -5.703 | -0.421 | -1.451 |
| 8 | -2.549 | 2.849 | -1.177 |
| 7 | -6.385 | 1.758 | -1.364 |
| 7 | -4.142 | 1.240 | -1.303 |
| 7 | -0.385 | 0.802 | -1.588 |
| 7 | 1.444 | -1.664 | -1.911 |
| 7 | 0.539 | -3.695 | -2.004 |
| 7 | -1.819 | -3.113 | -1.759 |
| 7 | -2.111 | -0.742 | -1.526 |
| 6 | -4.444 | 4.995 | -1.116 |
| 6 | -3.746 | 2.559 | -1.223 |
| 6 | -4.816 | 3.547 | -1.211 |
| 6 | -6.085 | 3.094 | -1.285 |
| 6 | -5.429 | 0.770 | -1.378 |
| 6 | 1.681 | -2.947 | -2.022 |
| 6 | -0.796 | -0.464 | -1.635 |
| 6 | 0.074 | -1.567 | -1.802 |
| 6 | -0.510 | -2.827 | -1.853 |
| 6 | -2.537 | -2.013 | -1.590 |
| 1 | -6.938 | 3.759 | -1.284 |
| 1 | -7.343 | 1.450 | -1.414 |
| 1 | -3.391 | 0.518 | -1.339 |
| 1 | -1.073 | 1.528 | -1.415 |
| 1 | 0.600 | 1.037 | -1.629 |
| 1 | 2.662 | -3.385 | -2.108 |
| 1 | 0.482 | -4.700 | -2.040 |
| 1 | -3.610 | -2.136 | -1.486 |
| 1 | -3.807 | 5.283 | -1.954 |
| 1 | -3.883 | 5.185 | -0.200 |
| 1 | -5.334 | 5.622 | -1.119 |
| 6 | 7.448 | -0.366 | -1.263 |
| 6 | 6.107 | 0.273 | -1.439 |
| 6 | 5.907 | 1.602 | -1.530 |
| 7 | 4.650 | 2.151 | -1.648 |
| 1 | 4.530 | 3.148 | -1.719 |
| 6 | 3.508 | 1.397 | -1.686 |
| 8 | 2.390 | 1.901 | -1.785 |
| 7 | 3.716 | 0.050 | -1.601 |
| 1 | 2.872 | -0.545 | -1.658 |
| 6 | 4.936 | -0.598 | -1.479 |
| 8 | 4.980 | -1.823 | -1.424 |
| 1 | 6.719 | 2.317 | -1.509 |
| 1 | 8.244 | 0.374 | -1.335 |
| 1 | 7.610 | -1.134 | -2.021 |
| 1 | 7.498 | -0.846 | -0.284 |

rG•G-C/rG•G-C

| 7 | 3.411 | -3.450 | -1.799 |
| --- | --- | --- | --- |
| 8 | 1.155 | 0.491 | -1.164 |
| 7 | 2.197 | -1.521 | -1.457 |
| 6 | 2.204 | -2.882 | -1.650 |
| 7 | 1.108 | -3.616 | -1.725 |
| 6 | -0.015 | -2.880 | -1.621 |
| 6 | -0.129 | -1.507 | -1.448 |
| 6 | 1.061 | -0.737 | -1.340 |
| 7 | -1.305 | -3.329 | -1.680 |
| 6 | -2.124 | -2.239 | -1.550 |
| 7 | -1.453 | -1.127 | -1.410 |
| 1 | 4.258 | -2.951 | -1.528 |
| 1 | 3.426 | -4.455 | -1.795 |
| 1 | 3.102 | -1.017 | -1.415 |
| 1 | -1.597 | -4.286 | -1.796 |
| 1 | -3.199 | -2.318 | -1.575 |
| 8 | 5.838 | -2.021 | -1.075 |
| 7 | 3.658 | 1.940 | -1.718 |
| 7 | 4.716 | -0.065 | -1.447 |
| 6 | 6.052 | 1.921 | -1.848 |
| 6 | 5.842 | -0.800 | -1.310 |
| 7 | 7.061 | -0.157 | -1.438 |
| 6 | 7.165 | 1.167 | -1.711 |
| 6 | 4.796 | 1.250 | -1.668 |
| 1 | 6.102 | 2.978 | -2.061 |
| 1 | 7.883 | -0.731 | -1.331 |
| 1 | 8.166 | 1.561 | -1.809 |
| 1 | 2.774 | 1.473 | -1.529 |
| 1 | 3.671 | 2.931 | -1.885 |
| 6 | -6.464 | 3.860 | -0.885 |
| 7 | -5.270 | 4.367 | -0.443 |
| 1 | -5.128 | 5.246 | 0.027 |
| 6 | -4.307 | 3.441 | -0.729 |
| 6 | -4.982 | 2.412 | -1.369 |
| 7 | -6.336 | 2.684 | -1.444 |
| 6 | -4.245 | 1.234 | -1.720 |
| 8 | -4.649 | 0.195 | -2.238 |
| 7 | -2.894 | 1.369 | -1.382 |
| 1 | -2.332 | 0.511 | -1.486 |
| 6 | -2.329 | 2.440 | -0.747 |
| 7 | -2.999 | 3.525 | -0.401 |
| 7 | -1.019 | 2.345 | -0.458 |
| 1 | -7.387 | 4.403 | -0.765 |
| 1 | -0.428 | 1.581 | -0.769 |
| 1 | -0.580 | 3.149 | -0.048 |
| 7 | 4.422 | -1.479 | 1.515 |
| 8 | 0.277 | 0.315 | 2.335 |
| 7 | 2.302 | -0.660 | 1.920 |
| 6 | 3.108 | -1.735 | 1.621 |
| 7 | 2.655 | -2.967 | 1.464 |
| 6 | 1.319 | -3.047 | 1.603 |
| 6 | 0.426 | -2.032 | 1.920 |
| 6 | 0.927 | -0.714 | 2.085 |
| 7 | 0.540 | -4.158 | 1.444 |
| 6 | -0.761 | -3.777 | 1.641 |
| 7 | -0.866 | -2.510 | 1.939 |
| 1 | 4.741 | -0.511 | 1.466 |
| 1 | 4.952 | -2.143 | 0.969 |
| 1 | 2.716 | 0.286 | 1.916 |
| 1 | 0.861 | -5.070 | 1.160 |
| 1 | -1.593 | -4.456 | 1.541 |
| 8 | 5.463 | 1.201 | 1.371 |
| 7 | 1.301 | 3.012 | 1.918 |
| 7 | 3.369 | 2.071 | 1.665 |
| 6 | 3.122 | 4.468 | 1.364 |
| 6 | 4.692 | 2.169 | 1.383 |
| 7 | 5.206 | 3.425 | 1.093 |
| 6 | 4.442 | 4.546 | 1.086 |
| 6 | 2.600 | 3.162 | 1.654 |
| 1 | 2.488 | 5.341 | 1.360 |
| 1 | 6.191 | 3.466 | 0.884 |
| 1 | 4.947 | 5.471 | 0.848 |
| 1 | 0.932 | 2.088 | 2.135 |
| 1 | 0.717 | 3.821 | 2.038 |
| 6 | -7.352 | -1.144 | -0.358 |
| 7 | -6.815 | 0.039 | 0.089 |
| 1 | -7.088 | 0.970 | -0.204 |
| 6 | -5.633 | -0.271 | 0.692 |
| 6 | -5.533 | -1.655 | 0.606 |
| 7 | -6.625 | -2.186 | -0.056 |
| 6 | -4.331 | -2.277 | 1.070 |
| 8 | -4.010 | -3.466 | 1.032 |
| 7 | -3.443 | -1.328 | 1.590 |
| 1 | -2.516 | -1.704 | 1.842 |
| 6 | -3.646 | 0.023 | 1.629 |
| 7 | -4.752 | 0.608 | 1.216 |
| 7 | -2.650 | 0.773 | 2.157 |
| 1 | -8.289 | -1.167 | -0.891 |
| 1 | -1.699 | 0.418 | 2.198 |
| 1 | -2.741 | 1.760 | 1.979 |

rA•A-T/rA•A-T

| 8 | 5.206 | -0.414 | -1.923 |
| --- | --- | --- | --- |
| 8 | 1.399 | -2.612 | -0.754 |
| 8 | 3.111 | -1.062 | 1.933 |
| 8 | 5.245 | 2.596 | 0.260 |
| 7 | 5.303 | -2.670 | -1.577 |
| 7 | 3.308 | -1.545 | -1.342 |
| 7 | -0.218 | -0.181 | -1.449 |
| 7 | -1.406 | 2.651 | -1.740 |
| 7 | -0.025 | 4.385 | -2.008 |
| 7 | 2.123 | 3.231 | -1.943 |
| 7 | 1.836 | 0.872 | -1.618 |
| 7 | -6.317 | 0.910 | -0.670 |
| 7 | -6.641 | -1.425 | -1.164 |
| 7 | -4.556 | -2.496 | -1.843 |
| 7 | -3.172 | -0.749 | -1.714 |
| 7 | -4.281 | 1.993 | -0.882 |
| 7 | -4.125 | 0.350 | 2.301 |
| 7 | -1.847 | -1.494 | 1.359 |
| 7 | -2.311 | -3.604 | 0.798 |
| 7 | -4.711 | -3.634 | 1.242 |
| 7 | -5.494 | -1.497 | 2.028 |
| 7 | 1.779 | 2.288 | 1.421 |
| 7 | 0.886 | 4.506 | 1.210 |
| 7 | -1.545 | 4.508 | 1.415 |
| 7 | -1.908 | 2.327 | 1.720 |
| 7 | 0.496 | 0.392 | 1.775 |
| 7 | 4.199 | 0.751 | 1.107 |
| 7 | 6.470 | 0.727 | 0.725 |
| 6 | 2.668 | -5.171 | -0.527 |
| 6 | 2.610 | -2.677 | -0.985 |
| 6 | 3.377 | -3.912 | -0.922 |
| 6 | 4.690 | -3.845 | -1.229 |
| 6 | 4.646 | -1.464 | -1.635 |
| 6 | -1.314 | 3.949 | -1.894 |
| 6 | 0.488 | 0.936 | -1.600 |
| 6 | -0.096 | 2.216 | -1.748 |
| 6 | 0.781 | 3.284 | -1.909 |
| 6 | 2.558 | 1.991 | -1.783 |
| 6 | -5.006 | 0.872 | -0.968 |
| 6 | -4.450 | -0.365 | -1.364 |
| 6 | -5.329 | -1.441 | -1.436 |
| 6 | -7.034 | -0.216 | -0.782 |
| 6 | -3.281 | -2.026 | -1.984 |
| 6 | -1.360 | -2.628 | 0.918 |
| 6 | -5.634 | -2.781 | 1.673 |
| 6 | -3.503 | -3.056 | 1.194 |
| 6 | -3.193 | -1.743 | 1.535 |
| 6 | -4.275 | -0.936 | 1.955 |
| 6 | 1.865 | 3.614 | 1.240 |
| 6 | -0.309 | 3.918 | 1.384 |
| 6 | -0.557 | 2.563 | 1.576 |
| 6 | 0.571 | 1.709 | 1.589 |
| 6 | -2.457 | 3.512 | 1.616 |
| 6 | 5.299 | 1.441 | 0.663 |
| 6 | 6.537 | -0.569 | 1.162 |
| 6 | 5.452 | -1.244 | 1.595 |
| 6 | 4.173 | -0.549 | 1.571 |
| 6 | 5.488 | -2.650 | 2.111 |
| 1 | 5.332 | -4.715 | -1.215 |
| 1 | 6.290 | -2.648 | -1.780 |
| 1 | 2.769 | -0.653 | -1.408 |
| 1 | 0.279 | -1.039 | -1.238 |
| 1 | -1.233 | -0.164 | -1.420 |
| 1 | -2.148 | 4.633 | -1.930 |
| 1 | 0.277 | 5.341 | -2.103 |
| 1 | 3.633 | 1.844 | -1.776 |
| 1 | -8.085 | -0.118 | -0.529 |
| 1 | -2.470 | -2.669 | -2.287 |
| 1 | -4.755 | 2.839 | -0.619 |
| 1 | -3.297 | 2.023 | -1.131 |
| 1 | -4.857 | -3.450 | -1.955 |
| 1 | 1.840 | -5.375 | -1.207 |
| 1 | 2.249 | -5.075 | 0.477 |
| 1 | 3.352 | -6.018 | -0.541 |
| 1 | 6.485 | -3.075 | 1.997 |
| 1 | 5.214 | -2.678 | 3.167 |
| 1 | 4.774 | -3.274 | 1.573 |
| 1 | -2.161 | -4.549 | 0.484 |
| 1 | -3.270 | 0.855 | 2.081 |
| 1 | -4.968 | 0.880 | 2.442 |
| 1 | -0.329 | -2.805 | 0.647 |
| 1 | -6.644 | -3.170 | 1.744 |
| 1 | 2.873 | 3.984 | 1.089 |
| 1 | -1.743 | 5.490 | 1.310 |
| 1 | -3.514 | 3.717 | 1.681 |
| 1 | -0.399 | -0.087 | 1.727 |
| 1 | 1.355 | -0.149 | 1.744 |
| 1 | 3.312 | 1.288 | 1.150 |
| 1 | 7.293 | 1.194 | 0.379 |
| 1 | 7.524 | -1.011 | 1.144 |

G4∙∙∙Na^+^

| 11 | 0.000 | 0.000 | -0.346 |
| --- | --- | --- | --- |
| 8 | 1.377 | -1.835 | -0.166 |
| 8 | 1.826 | 1.359 | 0.019 |
| 8 | -1.826 | -1.359 | 0.020 |
| 8 | -1.377 | 1.835 | -0.166 |
| 7 | -1.552 | -5.283 | 0.621 |
| 7 | 0.684 | -5.829 | 0.681 |
| 7 | -0.014 | -3.604 | 0.226 |
| 7 | 3.653 | -3.874 | 0.214 |
| 7 | 3.101 | -5.981 | 0.678 |
| 7 | 5.326 | -1.554 | -0.573 |
| 7 | 5.869 | 0.684 | -0.579 |
| 7 | 3.622 | -0.023 | -0.265 |
| 7 | 3.880 | 3.644 | -0.204 |
| 7 | 6.014 | 3.102 | -0.543 |
| 7 | -5.326 | 1.554 | -0.573 |
| 7 | -5.869 | -0.684 | -0.579 |
| 7 | -3.622 | 0.023 | -0.264 |
| 7 | -3.880 | -3.644 | -0.205 |
| 7 | -6.013 | -3.102 | -0.544 |
| 7 | 1.552 | 5.283 | 0.620 |
| 7 | -0.684 | 5.830 | 0.681 |
| 7 | 0.014 | 3.604 | 0.226 |
| 7 | -3.653 | 3.874 | 0.214 |
| 7 | -3.101 | 5.981 | 0.678 |
| 6 | -0.268 | -4.928 | 0.506 |
| 6 | 1.248 | -3.048 | 0.087 |
| 6 | 2.279 | -4.003 | 0.267 |
| 6 | 1.917 | -5.315 | 0.555 |
| 6 | 4.106 | -5.073 | 0.464 |
| 6 | 4.962 | -0.271 | -0.466 |
| 6 | 3.054 | 1.237 | -0.152 |
| 6 | 4.017 | 2.272 | -0.261 |
| 6 | 5.344 | 1.915 | -0.471 |
| 6 | 5.091 | 4.102 | -0.376 |
| 6 | -4.962 | 0.271 | -0.466 |
| 6 | -3.054 | -1.237 | -0.152 |
| 6 | -4.017 | -2.272 | -0.261 |
| 6 | -5.344 | -1.915 | -0.472 |
| 6 | -5.091 | -4.102 | -0.376 |
| 6 | 0.268 | 4.928 | 0.506 |
| 6 | -1.249 | 3.048 | 0.087 |
| 6 | -2.280 | 4.003 | 0.267 |
| 6 | -1.917 | 5.315 | 0.555 |
| 6 | -4.106 | 5.073 | 0.464 |
| 1 | -1.729 | -6.264 | 0.755 |
| 1 | -2.322 | -4.679 | 0.329 |
| 1 | 5.145 | -5.357 | 0.505 |
| 1 | -0.795 | -2.946 | 0.138 |
| 1 | 6.314 | -1.727 | -0.641 |
| 1 | 4.711 | -2.325 | -0.309 |
| 1 | 5.374 | 5.142 | -0.389 |
| 1 | 2.964 | -0.807 | -0.212 |
| 1 | -6.314 | 1.727 | -0.640 |
| 1 | -4.711 | 2.325 | -0.309 |
| 1 | -5.374 | -5.142 | -0.390 |
| 1 | -2.963 | 0.807 | -0.211 |
| 1 | 1.729 | 6.264 | 0.754 |
| 1 | 2.322 | 4.679 | 0.328 |
| 1 | -5.145 | 5.357 | 0.505 |
| 1 | 0.795 | 2.946 | 0.137 |
| 1 | -7.003 | -3.218 | -0.691 |
| 1 | 3.213 | -6.961 | 0.886 |
| 1 | 7.004 | 3.218 | -0.690 |
| 1 | -3.213 | 6.961 | 0.886 |

G4∙∙∙K^+^

| 8 | -1.421 | -1.895 | 0.416 |
| --- | --- | --- | --- |
| 8 | -1.827 | 1.349 | 0.166 |
| 8 | 1.827 | -1.349 | 0.168 |
| 8 | 1.422 | 1.894 | 0.414 |
| 7 | 1.579 | -5.127 | -0.866 |
| 7 | -0.644 | -5.699 | -1.026 |
| 7 | 0.006 | -3.555 | -0.233 |
| 7 | -3.655 | -3.890 | -0.305 |
| 7 | -3.060 | -5.893 | -1.074 |
| 7 | -5.332 | -1.591 | 0.562 |
| 7 | -5.898 | 0.641 | 0.469 |
| 7 | -3.628 | -0.046 | 0.329 |
| 7 | -3.919 | 3.615 | 0.162 |
| 7 | -6.065 | 3.056 | 0.361 |
| 7 | 5.332 | 1.591 | 0.560 |
| 7 | 5.898 | -0.641 | 0.468 |
| 7 | 3.628 | 0.045 | 0.329 |
| 7 | 3.919 | -3.616 | 0.162 |
| 7 | 6.065 | -3.056 | 0.361 |
| 7 | -1.579 | 5.126 | -0.867 |
| 7 | 0.644 | 5.699 | -1.025 |
| 7 | -0.006 | 3.554 | -0.235 |
| 7 | 3.655 | 3.890 | -0.304 |
| 7 | 3.059 | 5.893 | -1.073 |
| 6 | 0.287 | -4.816 | -0.708 |
| 6 | -1.269 | -3.048 | -0.021 |
| 6 | -2.279 | -3.986 | -0.360 |
| 6 | -1.890 | -5.231 | -0.838 |
| 6 | -4.083 | -5.044 | -0.738 |
| 6 | -4.975 | -0.305 | 0.443 |
| 6 | -3.062 | 1.220 | 0.235 |
| 6 | -4.045 | 2.244 | 0.243 |
| 6 | -5.379 | 1.876 | 0.368 |
| 6 | -5.144 | 4.063 | 0.234 |
| 6 | 4.975 | 0.305 | 0.442 |
| 6 | 3.062 | -1.220 | 0.235 |
| 6 | 4.045 | -2.244 | 0.243 |
| 6 | 5.379 | -1.876 | 0.367 |
| 6 | 5.144 | -4.063 | 0.234 |
| 6 | -0.287 | 4.816 | -0.709 |
| 6 | 1.270 | 3.048 | -0.022 |
| 6 | 2.279 | 3.986 | -0.360 |
| 6 | 1.890 | 5.231 | -0.838 |
| 6 | 4.083 | 5.045 | -0.737 |
| 1 | 1.774 | -6.073 | -1.147 |
| 1 | 2.337 | -4.568 | -0.473 |
| 1 | -5.116 | -5.337 | -0.836 |
| 1 | 0.775 | -2.897 | -0.061 |
| 1 | -6.322 | -1.768 | 0.541 |
| 1 | -4.706 | -2.355 | 0.302 |
| 1 | -5.438 | 5.099 | 0.198 |
| 1 | -2.961 | -0.825 | 0.336 |
| 1 | 6.322 | 1.768 | 0.539 |
| 1 | 4.705 | 2.355 | 0.301 |
| 1 | 5.438 | -5.099 | 0.199 |
| 1 | 2.961 | 0.825 | 0.335 |
| 1 | -1.774 | 6.072 | -1.147 |
| 1 | -2.337 | 4.568 | -0.474 |
| 1 | 5.116 | 5.338 | -0.833 |
| 1 | -0.775 | 2.896 | -0.063 |
| 1 | 3.151 | 6.832 | -1.427 |
| 1 | -7.064 | 3.164 | 0.436 |
| 1 | -3.151 | -6.831 | -1.430 |
| 1 | 7.064 | -3.164 | 0.435 |
| 19 | 0.000 | 0.000 | 1.652 |

G4∙∙∙Na^+^∙∙∙G4

| 8 | -2.171 | 0.570 | -1.322 |
| --- | --- | --- | --- |
| 8 | 0.570 | 2.170 | -1.322 |
| 8 | -0.570 | -2.170 | -1.322 |
| 8 | 2.171 | -0.571 | -1.322 |
| 7 | -4.393 | -3.435 | -1.735 |
| 7 | -5.710 | -1.554 | -1.728 |
| 7 | -3.345 | -1.383 | -1.522 |
| 7 | -4.935 | 1.928 | -1.426 |
| 7 | -6.734 | 0.636 | -1.628 |
| 7 | -3.435 | 4.393 | -1.734 |
| 7 | -1.554 | 5.709 | -1.727 |
| 7 | -1.383 | 3.345 | -1.522 |
| 7 | 1.928 | 4.934 | -1.427 |
| 7 | 0.637 | 6.734 | -1.627 |
| 7 | 3.435 | -4.393 | -1.732 |
| 7 | 1.554 | -5.710 | -1.726 |
| 7 | 1.383 | -3.345 | -1.521 |
| 7 | -1.928 | -4.934 | -1.427 |
| 7 | -0.637 | -6.734 | -1.627 |
| 7 | 4.393 | 3.435 | -1.734 |
| 7 | 5.710 | 1.553 | -1.728 |
| 7 | 3.345 | 1.383 | -1.521 |
| 7 | 4.935 | -1.928 | -1.427 |
| 7 | 6.734 | -0.637 | -1.629 |
| 6 | -4.510 | -2.106 | -1.655 |
| 6 | -3.269 | 0.002 | -1.438 |
| 6 | -4.556 | 0.604 | -1.500 |
| 6 | -5.668 | -0.217 | -1.628 |
| 6 | -6.238 | 1.907 | -1.502 |
| 6 | -2.105 | 4.509 | -1.655 |
| 6 | 0.002 | 3.268 | -1.438 |
| 6 | 0.605 | 4.555 | -1.500 |
| 6 | -0.217 | 5.668 | -1.628 |
| 6 | 1.908 | 6.237 | -1.502 |
| 6 | 2.106 | -4.510 | -1.654 |
| 6 | -0.002 | -3.268 | -1.438 |
| 6 | -0.604 | -4.555 | -1.500 |
| 6 | 0.217 | -5.668 | -1.627 |
| 6 | -1.908 | -6.237 | -1.502 |
| 6 | 4.510 | 2.105 | -1.655 |
| 6 | 3.269 | -0.003 | -1.438 |
| 6 | 4.556 | -0.605 | -1.501 |
| 6 | 5.668 | 0.217 | -1.628 |
| 6 | 6.238 | -1.908 | -1.503 |
| 1 | -5.253 | -3.955 | -1.741 |
| 1 | -3.509 | -3.924 | -1.574 |
| 1 | -6.884 | 2.770 | -1.472 |
| 1 | -2.440 | -1.862 | -1.454 |
| 1 | -3.954 | 5.253 | -1.740 |
| 1 | -3.924 | 3.509 | -1.573 |
| 1 | 2.770 | 6.883 | -1.473 |
| 1 | -1.862 | 2.439 | -1.453 |
| 1 | 3.955 | -5.253 | -1.738 |
| 1 | 3.924 | -3.509 | -1.572 |
| 1 | -2.770 | -6.883 | -1.473 |
| 1 | 1.862 | -2.440 | -1.452 |
| 1 | 5.253 | 3.954 | -1.740 |
| 1 | 3.510 | 3.924 | -1.573 |
| 1 | 6.884 | -2.771 | -1.474 |
| 1 | 2.440 | 1.861 | -1.453 |
| 1 | -0.377 | -7.704 | -1.701 |
| 1 | -7.705 | 0.376 | -1.702 |
| 1 | 0.377 | 7.704 | -1.701 |
| 1 | 7.705 | -0.377 | -1.702 |
| 11 | 0.000 | 0.000 | 0.000 |
| 8 | -1.937 | -1.132 | 1.321 |
| 8 | -1.132 | 1.938 | 1.321 |
| 8 | 1.132 | -1.937 | 1.322 |
| 8 | 1.937 | 1.132 | 1.321 |
| 7 | -0.676 | -5.535 | 1.734 |
| 7 | -2.938 | -5.136 | 1.727 |
| 7 | -1.386 | -3.343 | 1.521 |
| 7 | -4.852 | -2.126 | 1.425 |
| 7 | -5.211 | -4.312 | 1.627 |
| 7 | -5.536 | 0.676 | 1.734 |
| 7 | -5.137 | 2.938 | 1.729 |
| 7 | -3.344 | 1.386 | 1.521 |
| 7 | -2.127 | 4.852 | 1.427 |
| 7 | -4.312 | 5.211 | 1.629 |
| 7 | 5.536 | -0.676 | 1.733 |
| 7 | 5.137 | -2.937 | 1.728 |
| 7 | 3.344 | -1.386 | 1.521 |
| 7 | 2.126 | -4.851 | 1.428 |
| 7 | 4.312 | -5.210 | 1.629 |
| 7 | 0.676 | 5.536 | 1.734 |
| 7 | 2.938 | 5.137 | 1.728 |
| 7 | 1.386 | 3.344 | 1.521 |
| 7 | 4.852 | 2.126 | 1.426 |
| 7 | 5.211 | 4.312 | 1.627 |
| 6 | -1.699 | -4.678 | 1.655 |
| 6 | -2.312 | -2.310 | 1.437 |
| 6 | -3.648 | -2.794 | 1.499 |
| 6 | -3.853 | -4.161 | 1.627 |
| 6 | -5.759 | -3.062 | 1.500 |
| 6 | -4.678 | 1.699 | 1.656 |
| 6 | -2.310 | 2.312 | 1.437 |
| 6 | -2.794 | 3.648 | 1.500 |
| 6 | -4.162 | 3.854 | 1.629 |
| 6 | -3.063 | 5.759 | 1.502 |
| 6 | 4.678 | -1.698 | 1.655 |
| 6 | 2.310 | -2.311 | 1.438 |
| 6 | 2.794 | -3.647 | 1.501 |
| 6 | 4.162 | -3.853 | 1.629 |
| 6 | 3.063 | -5.758 | 1.503 |
| 6 | 1.699 | 4.678 | 1.655 |
| 6 | 2.312 | 2.310 | 1.437 |
| 6 | 3.647 | 2.794 | 1.500 |
| 6 | 3.853 | 4.162 | 1.628 |
| 6 | 5.759 | 3.063 | 1.501 |
| 1 | -0.917 | -6.511 | 1.740 |
| 1 | 0.294 | -5.256 | 1.574 |
| 1 | -6.825 | -2.909 | 1.471 |
| 1 | -0.408 | -3.041 | 1.453 |
| 1 | -6.511 | 0.917 | 1.740 |
| 1 | -5.256 | -0.294 | 1.573 |
| 1 | -2.909 | 6.825 | 1.474 |
| 1 | -3.042 | 0.408 | 1.453 |
| 1 | 6.512 | -0.916 | 1.740 |
| 1 | 5.256 | 0.295 | 1.572 |
| 1 | 2.909 | -6.825 | 1.475 |
| 1 | 3.042 | -0.407 | 1.453 |
| 1 | 0.917 | 6.511 | 1.740 |
| 1 | -0.294 | 5.256 | 1.573 |
| 1 | 6.825 | 2.909 | 1.472 |
| 1 | 0.407 | 3.042 | 1.453 |
| 1 | 5.182 | -5.712 | 1.704 |
| 1 | -5.713 | -5.182 | 1.701 |
| 1 | -5.182 | 5.713 | 1.703 |
| 1 | 5.713 | 5.182 | 1.701 |

G4∙∙∙K^+^∙∙∙G4

| 8 | 0.696 | -2.220 | -1.532 |
| --- | --- | --- | --- |
| 8 | -2.219 | -0.702 | -1.528 |
| 8 | 2.217 | 0.696 | -1.543 |
| 8 | -0.698 | 2.215 | -1.538 |
| 7 | 5.270 | -1.792 | -1.748 |
| 7 | 4.463 | -3.943 | -1.670 |
| 7 | 2.977 | -2.087 | -1.618 |
| 7 | 1.137 | -5.267 | -1.514 |
| 7 | 3.229 | -6.025 | -1.566 |
| 7 | -1.793 | -5.277 | -1.713 |
| 7 | -3.943 | -4.469 | -1.626 |
| 7 | -2.087 | -2.983 | -1.597 |
| 7 | -5.266 | -1.142 | -1.492 |
| 7 | -6.024 | -3.234 | -1.522 |
| 7 | 1.792 | 5.271 | -1.711 |
| 7 | 3.942 | 4.463 | -1.623 |
| 7 | 2.085 | 2.977 | -1.604 |
| 7 | 5.265 | 1.135 | -1.505 |
| 7 | 6.023 | 3.227 | -1.523 |
| 7 | -5.271 | 1.785 | -1.741 |
| 7 | -4.465 | 3.937 | -1.672 |
| 7 | -2.978 | 2.081 | -1.617 |
| 7 | -1.139 | 5.263 | -1.531 |
| 7 | -3.231 | 6.019 | -1.581 |
| 6 | 4.237 | -2.640 | -1.668 |
| 6 | 1.789 | -2.808 | -1.563 |
| 6 | 2.021 | -4.210 | -1.555 |
| 6 | 3.333 | -4.664 | -1.598 |
| 6 | 1.894 | -6.332 | -1.518 |
| 6 | -2.640 | -4.244 | -1.635 |
| 6 | -2.807 | -1.795 | -1.547 |
| 6 | -4.209 | -2.026 | -1.531 |
| 6 | -4.664 | -3.338 | -1.560 |
| 6 | -6.331 | -1.898 | -1.484 |
| 6 | 2.639 | 4.238 | -1.636 |
| 6 | 2.806 | 1.788 | -1.559 |
| 6 | 4.207 | 2.020 | -1.541 |
| 6 | 4.662 | 3.332 | -1.562 |
| 6 | 6.329 | 1.891 | -1.493 |
| 6 | -4.238 | 2.634 | -1.666 |
| 6 | -1.790 | 2.802 | -1.568 |
| 6 | -2.022 | 4.204 | -1.566 |
| 6 | -3.335 | 4.658 | -1.607 |
| 6 | -1.896 | 6.327 | -1.538 |
| 1 | 6.182 | -2.207 | -1.679 |
| 1 | 5.181 | -0.783 | -1.616 |
| 1 | 1.546 | -7.352 | -1.488 |
| 1 | 2.869 | -1.066 | -1.613 |
| 1 | -2.207 | -6.190 | -1.637 |
| 1 | -0.782 | -5.188 | -1.596 |
| 1 | -7.350 | -1.550 | -1.452 |
| 1 | -1.066 | -2.875 | -1.596 |
| 1 | 2.206 | 6.184 | -1.632 |
| 1 | 0.780 | 5.182 | -1.601 |
| 1 | 7.349 | 1.543 | -1.462 |
| 1 | 1.064 | 2.869 | -1.604 |
| 1 | -6.184 | 2.200 | -1.672 |
| 1 | -5.183 | 0.776 | -1.606 |
| 1 | -1.549 | 7.347 | -1.513 |
| 1 | -2.870 | 1.060 | -1.606 |
| 1 | -6.680 | -3.999 | -1.535 |
| 1 | -3.996 | 6.674 | -1.605 |
| 1 | 6.679 | 3.992 | -1.532 |
| 1 | 3.994 | -6.680 | -1.589 |
| 19 | 0.001 | -0.001 | -0.020 |
| 8 | 1.894 | -1.362 | 1.477 |
| 8 | -1.363 | -1.888 | 1.486 |
| 8 | 1.364 | 1.894 | 1.472 |
| 8 | -1.893 | 1.369 | 1.470 |
| 7 | 5.316 | 1.697 | 1.713 |
| 7 | 5.947 | -0.513 | 1.618 |
| 7 | 3.650 | 0.100 | 1.546 |
| 7 | 4.051 | -3.554 | 1.550 |
| 7 | 6.186 | -2.925 | 1.572 |
| 7 | 1.696 | -5.312 | 1.704 |
| 7 | -0.515 | -5.941 | 1.634 |
| 7 | 0.099 | -3.645 | 1.548 |
| 7 | -3.555 | -4.043 | 1.592 |
| 7 | -2.927 | -6.179 | 1.617 |
| 7 | -1.695 | 5.319 | 1.685 |
| 7 | 0.516 | 5.947 | 1.630 |
| 7 | -0.098 | 3.651 | 1.533 |
| 7 | 3.556 | 4.048 | 1.611 |
| 7 | 2.928 | 6.184 | 1.634 |
| 7 | -5.314 | -1.690 | 1.726 |
| 7 | -5.945 | 0.519 | 1.628 |
| 7 | -3.648 | -0.094 | 1.549 |
| 7 | -4.050 | 3.561 | 1.547 |
| 7 | -6.185 | 2.931 | 1.580 |
| 6 | 4.992 | 0.403 | 1.615 |
| 6 | 3.121 | -1.186 | 1.529 |
| 6 | 4.138 | -2.178 | 1.567 |
| 6 | 5.464 | -1.766 | 1.586 |
| 6 | 5.291 | -3.963 | 1.551 |
| 6 | 0.402 | -4.987 | 1.618 |
| 6 | -1.187 | -3.115 | 1.542 |
| 6 | -2.179 | -4.131 | 1.595 |
| 6 | -1.768 | -5.457 | 1.616 |
| 6 | -3.965 | -5.282 | 1.604 |
| 6 | -0.401 | 4.993 | 1.606 |
| 6 | 1.188 | 3.121 | 1.534 |
| 6 | 2.180 | 4.136 | 1.601 |
| 6 | 1.769 | 5.463 | 1.620 |
| 6 | 3.966 | 5.287 | 1.629 |
| 6 | -4.990 | -0.397 | 1.623 |
| 6 | -3.120 | 1.193 | 1.527 |
| 6 | -4.136 | 2.184 | 1.567 |
| 6 | -5.462 | 1.772 | 1.593 |
| 6 | -5.289 | 3.969 | 1.553 |
| 1 | 6.297 | 1.910 | 1.674 |
| 1 | 4.640 | 2.458 | 1.627 |
| 1 | 5.614 | -4.991 | 1.534 |
| 1 | 2.958 | 0.857 | 1.522 |
| 1 | 1.907 | -6.293 | 1.658 |
| 1 | 2.456 | -4.638 | 1.594 |
| 1 | -4.993 | -5.606 | 1.600 |
| 1 | 0.856 | -2.953 | 1.516 |
| 1 | -1.906 | 6.300 | 1.647 |
| 1 | -2.456 | 4.645 | 1.579 |
| 1 | 4.995 | 5.610 | 1.636 |
| 1 | -0.855 | 2.960 | 1.497 |
| 1 | -6.296 | -1.902 | 1.684 |
| 1 | -4.640 | -2.451 | 1.625 |
| 1 | -5.613 | 4.997 | 1.535 |
| 1 | -2.956 | -0.850 | 1.525 |
| 1 | -3.003 | -7.183 | 1.634 |
| 1 | -7.189 | 3.007 | 1.596 |
| 1 | 3.005 | 7.188 | 1.654 |
| 1 | 7.191 | -3.000 | 1.583 |

Cartesian coordinates of optimized structures in the water phase (COSMO) by CCSD/aug-cc-pVDZ.

A-T

| 7 | 0.995 | 0.393 | 0.000 |
| --- | --- | --- | --- |
| 6 | 1.617 | 1.582 | 0.000 |
| 7 | 2.918 | 1.828 | 0.000 |
| 6 | 3.632 | 0.689 | 0.000 |
| 6 | 3.130 | -0.610 | 0.000 |
| 6 | 1.724 | -0.741 | 0.000 |
| 7 | 4.990 | 0.536 | 0.000 |
| 6 | 5.240 | -0.809 | 0.000 |
| 7 | 4.151 | -1.538 | 0.000 |
| 7 | 1.098 | -1.919 | 0.000 |
| 1 | 0.954 | 2.441 | 0.000 |
| 1 | 5.680 | 1.273 | 0.000 |
| 1 | 6.249 | -1.190 | 0.000 |
| 1 | 1.635 | -2.769 | 0.000 |
| 1 | 0.084 | -1.954 | 0.000 |
| 7 | -1.892 | 0.376 | 0.000 |
| 6 | -2.472 | 1.620 | 0.000 |
| 7 | -3.842 | 1.599 | 0.000 |
| 6 | -4.575 | 0.440 | 0.000 |
| 6 | -3.999 | -0.780 | 0.000 |
| 6 | -2.547 | -0.838 | 0.000 |
| 8 | -1.903 | -1.892 | 0.000 |
| 8 | -1.819 | 2.658 | 0.000 |
| 6 | -4.765 | -2.068 | 0.000 |
| 1 | -0.854 | 0.363 | 0.000 |
| 1 | -4.308 | 2.495 | 0.000 |
| 1 | -5.648 | 0.576 | 0.000 |
| 1 | -5.837 | -1.874 | 0.000 |
| 1 | -4.514 | -2.664 | 0.880 |
| 1 | -4.514 | -2.664 | -0.880 |

G-C

| 7 | -2.474 | 0.731 | 0.000 |
| --- | --- | --- | --- |
| 8 | 1.512 | -1.596 | 0.000 |
| 7 | -0.526 | -0.531 | 0.000 |
| 6 | -1.900 | -0.492 | 0.000 |
| 7 | -2.661 | -1.582 | 0.000 |
| 6 | -1.931 | -2.714 | 0.000 |
| 6 | -0.536 | -2.875 | 0.000 |
| 6 | 0.268 | -1.694 | 0.000 |
| 7 | -2.427 | -3.995 | 0.000 |
| 6 | -1.335 | -4.851 | 0.000 |
| 7 | -0.189 | -4.216 | 0.000 |
| 1 | -1.928 | 1.605 | 0.000 |
| 1 | -3.483 | 0.759 | 0.000 |
| 1 | 0.000 | 0.373 | 0.000 |
| 1 | -3.409 | -4.241 | 0.000 |
| 1 | -1.461 | -5.928 | 0.000 |
| 8 | -0.965 | 3.172 | 0.000 |
| 7 | 2.926 | 0.735 | 0.000 |
| 7 | 0.972 | 1.932 | 0.000 |
| 6 | 3.076 | 3.149 | 0.000 |
| 6 | 0.273 | 3.094 | 0.000 |
| 7 | 1.017 | 4.295 | 0.000 |
| 6 | 2.378 | 4.314 | 0.000 |
| 6 | 2.316 | 1.924 | 0.000 |
| 1 | 4.163 | 3.146 | 0.000 |
| 1 | 0.473 | 5.152 | 0.000 |
| 1 | 2.853 | 5.292 | 0.000 |
| 1 | 2.366 | -0.160 | 0.000 |
| 1 | 3.935 | 0.692 | 0.000 |

G-T

| 6 | 4.530 | -2.147 | 0.000 |
| --- | --- | --- | --- |
| 7 | 4.937 | -0.845 | 0.000 |
| 1 | 5.855 | -0.530 | 0.000 |
| 6 | 3.815 | -0.064 | 0.000 |
| 6 | 2.728 | -0.945 | 0.000 |
| 7 | 3.231 | -2.203 | 0.000 |
| 6 | 1.420 | -0.408 | 0.000 |
| 8 | 0.444 | -1.139 | 0.000 |
| 7 | 1.279 | 0.935 | 0.000 |
| 1 | 0.392 | 1.326 | 0.000 |
| 6 | 2.377 | 1.743 | 0.000 |
| 7 | 3.597 | 1.256 | 0.000 |
| 7 | 2.203 | 3.103 | 0.000 |
| 1 | 5.188 | -3.003 | 0.000 |
| 1 | 1.309 | 3.477 | 0.000 |
| 1 | 2.975 | 3.691 | 0.000 |
| 6 | -5.534 | -1.431 | 0.000 |
| 6 | -4.340 | -0.513 | 0.000 |
| 6 | -4.508 | 0.826 | 0.000 |
| 7 | -3.413 | 1.645 | 0.000 |
| 1 | -3.533 | 2.607 | 0.000 |
| 6 | -2.170 | 1.131 | 0.000 |
| 8 | -1.211 | 1.877 | 0.000 |
| 7 | -1.974 | -0.200 | 0.000 |
| 1 | -1.071 | -0.554 | 0.000 |
| 6 | -3.027 | -1.041 | 0.000 |
| 8 | -2.850 | -2.245 | 0.000 |
| 1 | -5.502 | 1.247 | 0.000 |
| 1 | -6.432 | -0.849 | 0.000 |
| 1 | -5.509 | -2.048 | 0.874 |
| 1 | -5.509 | -2.048 | -0.874 |
