# Supplementary material for: A unified computational view of DNA duplex, triplex, quadruplex and their donor–acceptor interactions
Source: Nucleic Acids Res. 2021 Apr 24;49(9):4919–33. doi: 10.1093/nar/gkab285 (PMC8136788; doi:10.1093/nar/gkab285)
Supplement: gkab285_Supplemental_Files [file gkab285_supplemental_files.zip › Revised Supporting Information.docx]

SUPPORTING INFORMATION

A unified computational view of DNA duplex, triplex, quadruplex and their donor-acceptor interactions

Gyuri Park^1^, Byunghwa Kang^1^, Soyeon V. Park^1^, Donghwa Lee^1,2,*^, Seung Soo Oh^1,3,4,**^

^1^Department of Materials Science and Engineering, Pohang University of Science Technology (POSTECH), Pohang, 37673, South Korea

^2^Division of Advanced Materials Science, Pohang University of Science and Technology (POSTECH), Pohang, 37673, South Korea

^3^School of Interdisciplinary Bioscience and Bioengineering, Pohang University of Science and Technology (POSTECH), Pohang, 37673, South Korea

^4^Institute of Convergence Technology, Yonsei University, Incheon, 21983, South Korea

* To whom correspondence should be addressed. Tel: +82 54 279 2160; Email: donghwa96@postech.ac.kr

**Correspondence may also be addressed to Tel: +82 54 279 2144; Email: seungsoo@postech.ac.kr


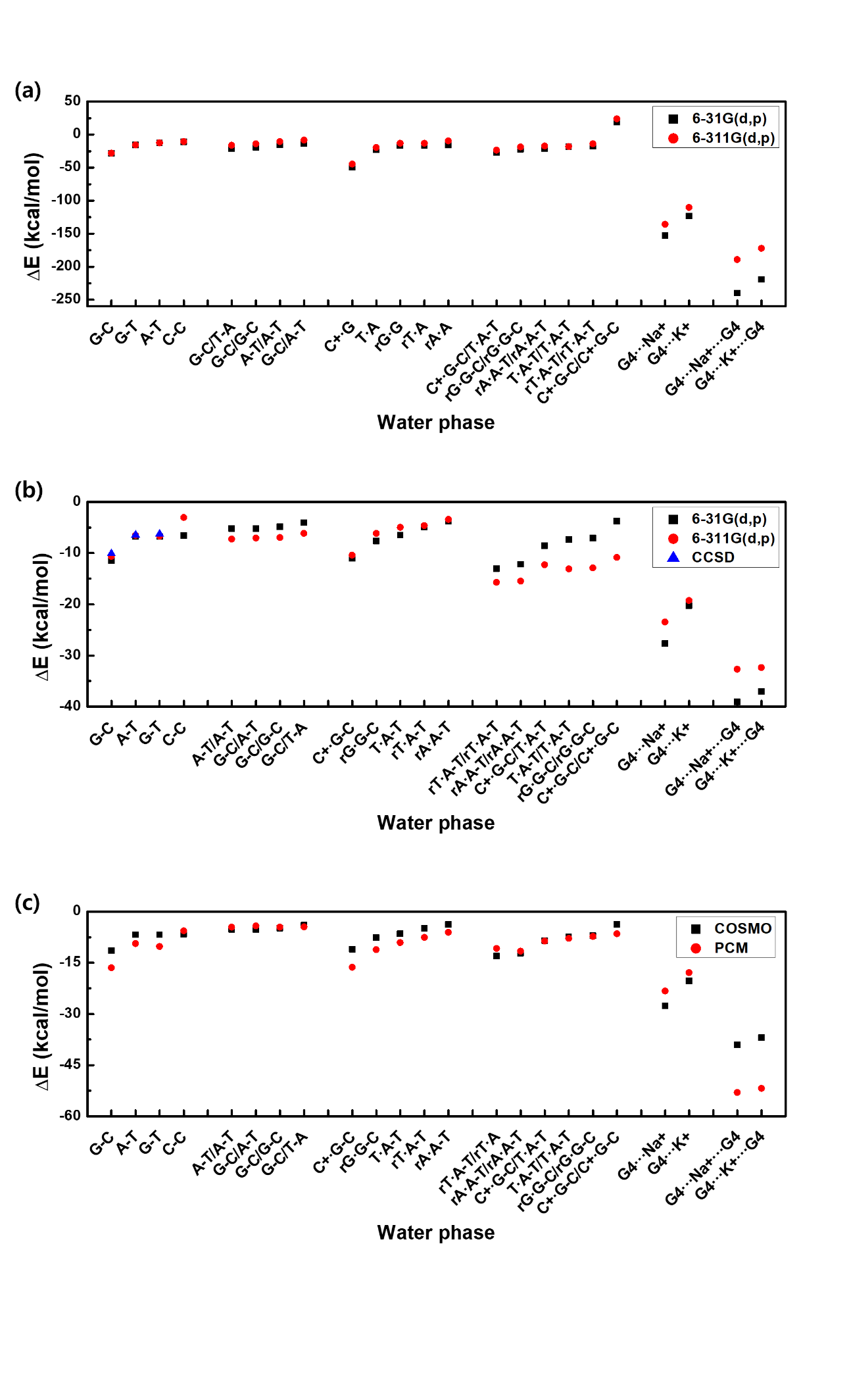
Figure S1. Control calculations (red and blue) to be compared with the calculation results by M05-2X/6-31G(d,p)//M05-2X/6-31G(d,p) (black). (a) Δ*E*s calculated by M05-2X/6-31G(d,p)//M05-2X/6-31G(d,p) and M05-2X/6-311G(d,p)//M05-2X/6-311G(d,p) in vacuum. The completely same energy order was observed at both calculation conditions. (b) Δ*E*s calculated by M05-2X/6-31G(d,p)//M05-2X/6-31G(d,p), M05-2X/6-311G(d,p)//M05-2X/6-311G(d,p) (basis set control), and CCSD(T)/aug-cc-pVDZ (method control) in the COSMO solvation. The CCSD calculation results matched well with the M05-2X results, showing the reliability of our M05-2X calculations. Moreover, the similar Δ*E* trends under double-zeta and triple-zeta polarization basis sets justified that our calculation method (M05-2X/6-31G(d,p)//M05-2X/6-31G(d,p)) would be sufficient to describe various nucleic acid structures. ssTo calculate Δ*E_ionic_* of G-quadruplex structure, we subtracted the Σ*E_G_*, ΣΔ*E_pair (G-G)_*, Σ*E_metal ion_*, and Δ*E_stack_* from *E_G-quadruplex_*, and each component yielded a slightly different energy value under different basis sets, thus leading to relatively wide disparities. However, the stability trend of G-quadruplexs is still maintained. (c) Δ*E*s calculated by M05-2X/6-31G(d,p)//M05-2X/6-31G(d,p) in the COSMO and IEFPCM solvation. They exhibited the similar trend, but the energy order by COSMO is relatively matched well with experimental data (*e.g.*, energy order in IEFPCM was G-C>G-T>A-T).


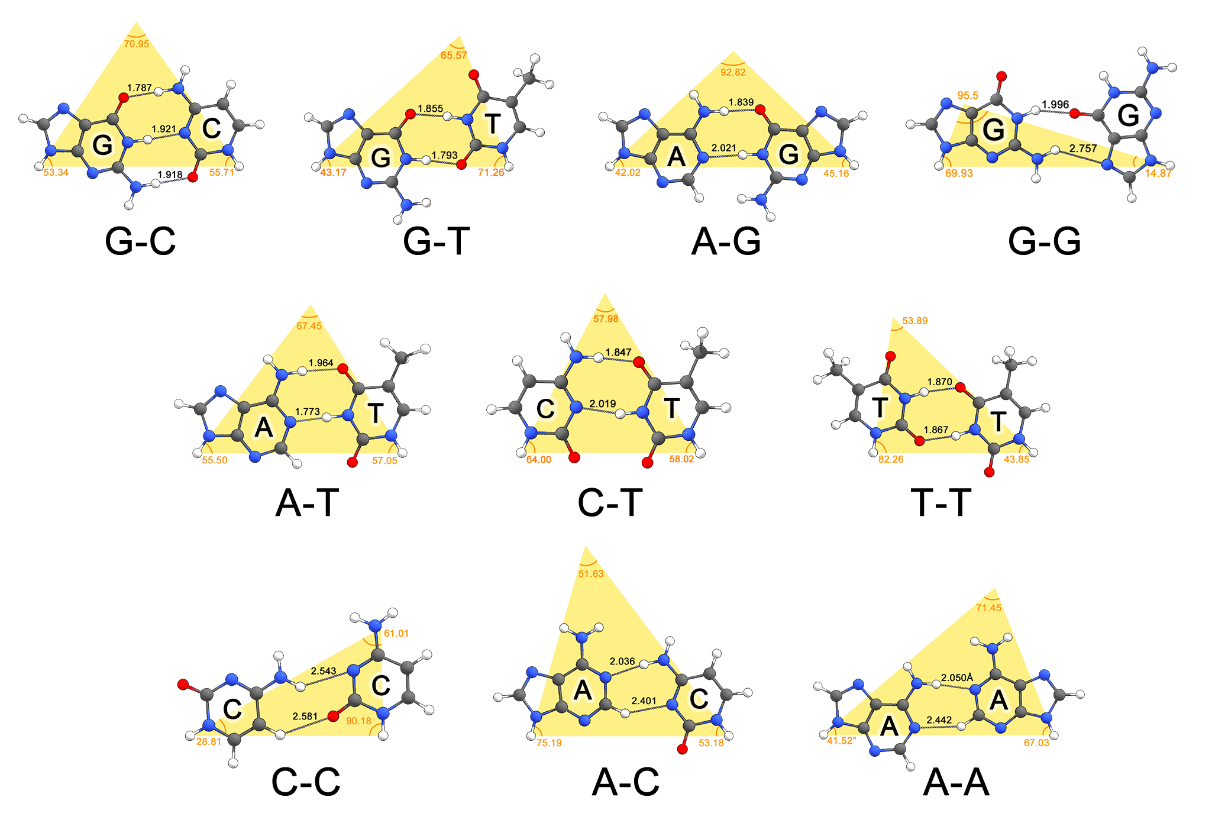
Figure S2. Optimized two-base/one-layer structures with HBLs (black) and angles containing GVAs (orange) in the vacuum.


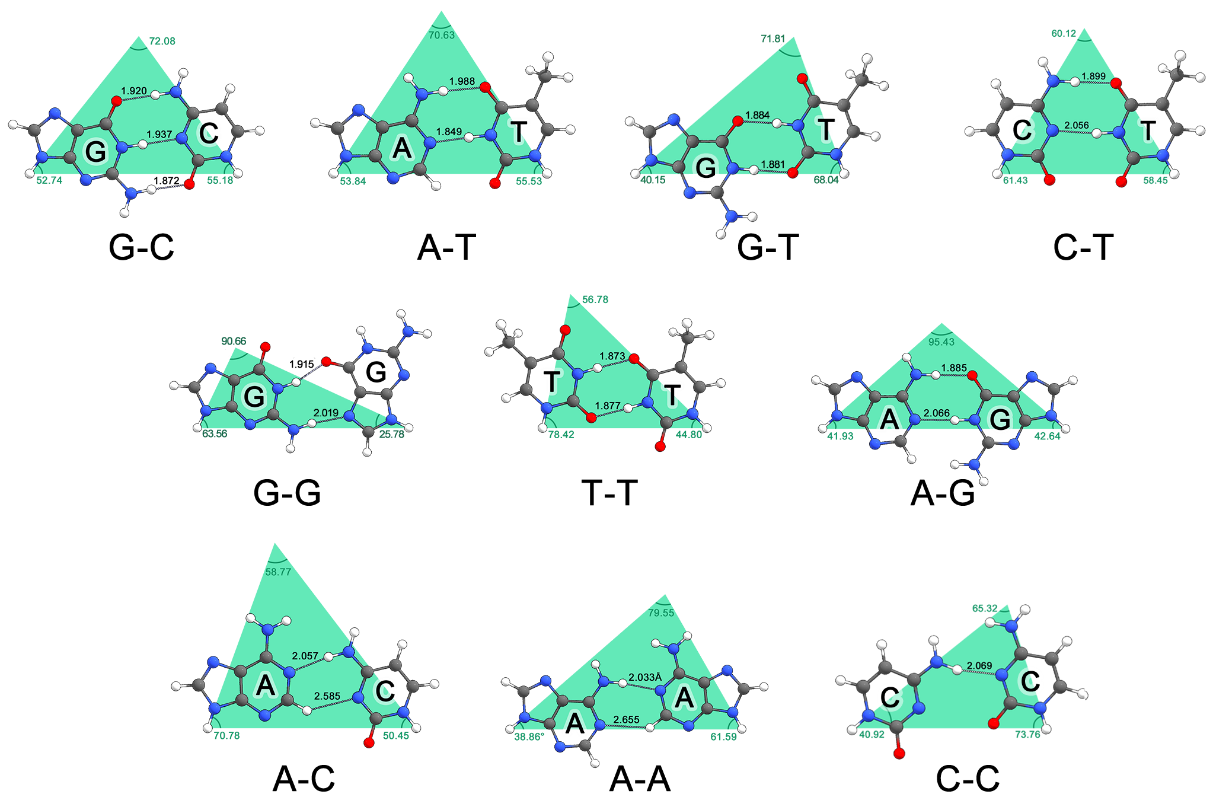


Figure S3. Optimized two-base/one-layer structures with HBLs (black) and angles containing GVAs (green) in the water phase.


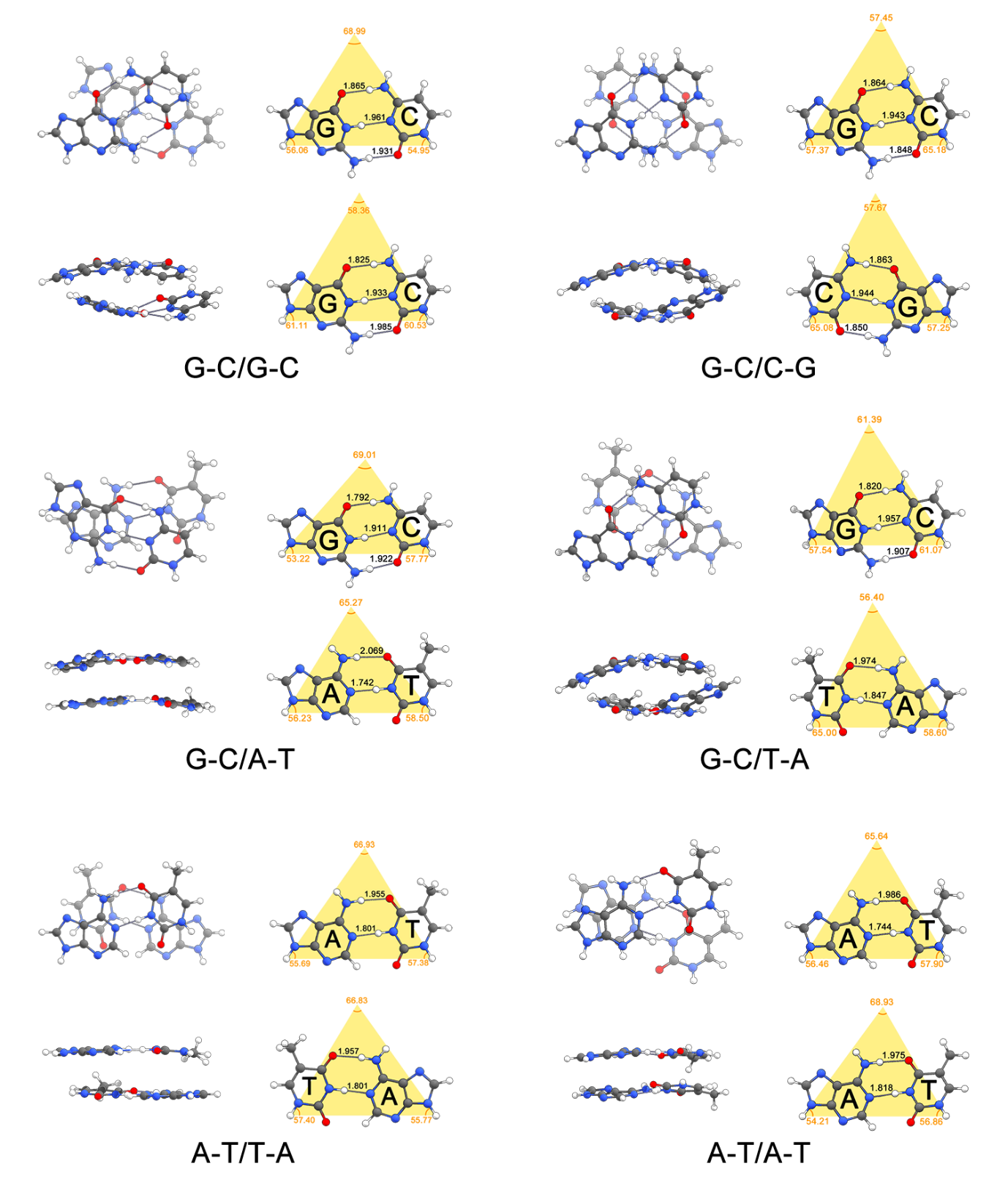


Figure S4. Optimized two-base/two-layer structures with HBLs (black) and angles containing GVAs (orange) in the gas phase. Each structure contains frontal and profile views of whole structures and frontal views of each layer.


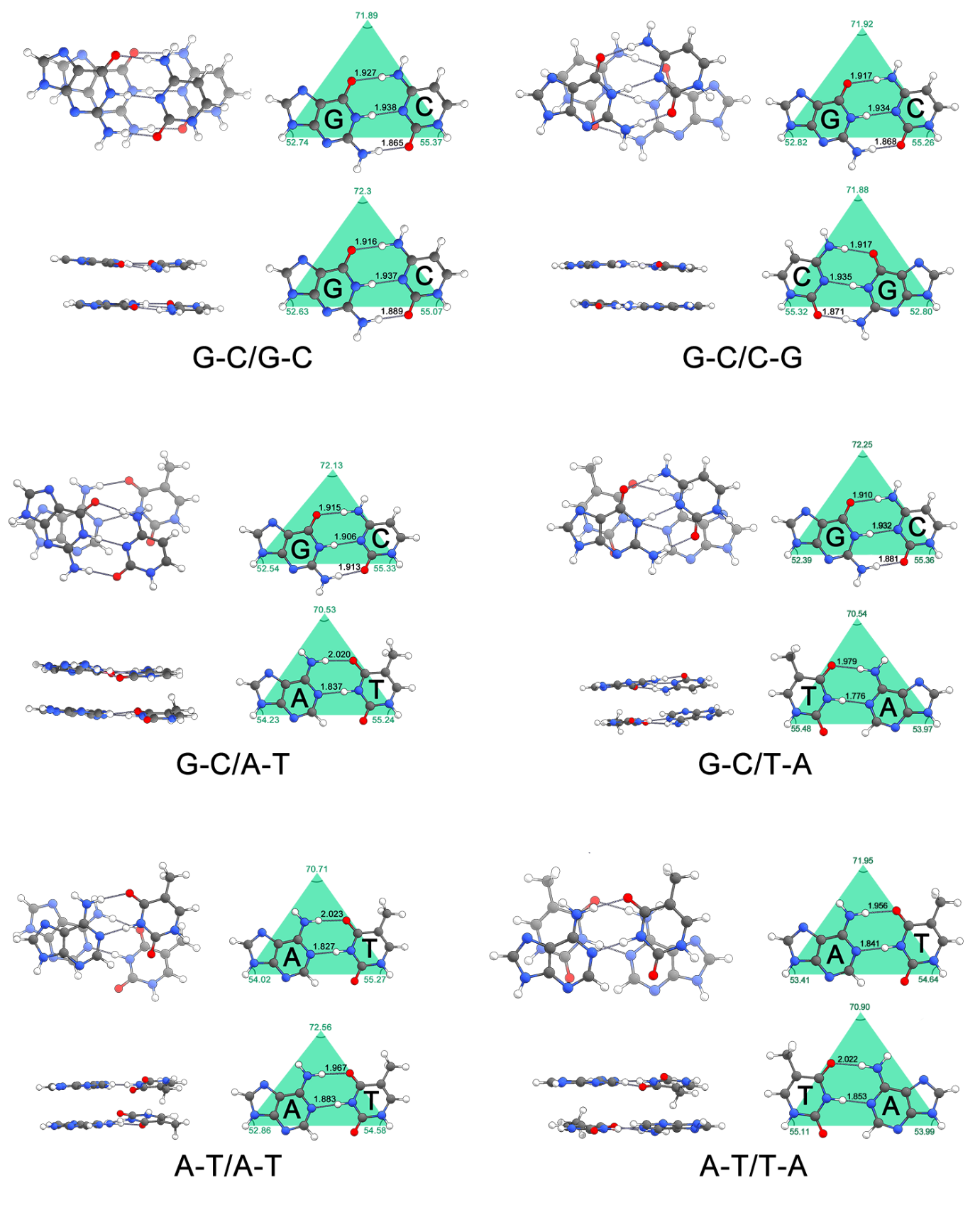


Figure S5. Optimized two-base/two-layer structures with HBLs (black) and angles containing GVAs (green) in the water phase. Each structure contains of frontal and profile views of whole structures and frontal views of each layer.


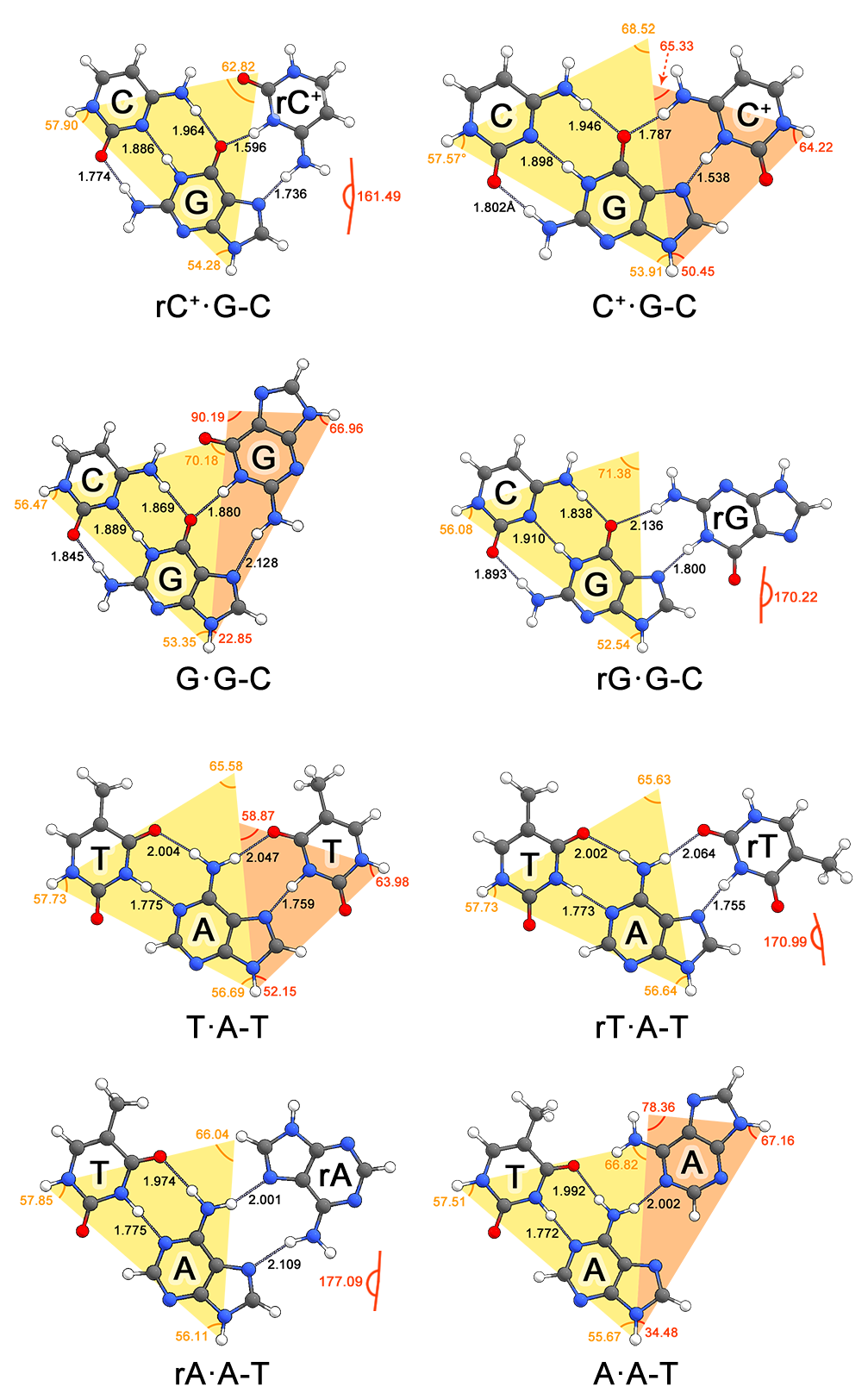
Figure S6. Optimized three-base/one-layer structures with HBLs (black) and angles containing GVAs of Watson-Crick pairs (orange) and that of Hoogsteen pairs (red) in the vacuum.


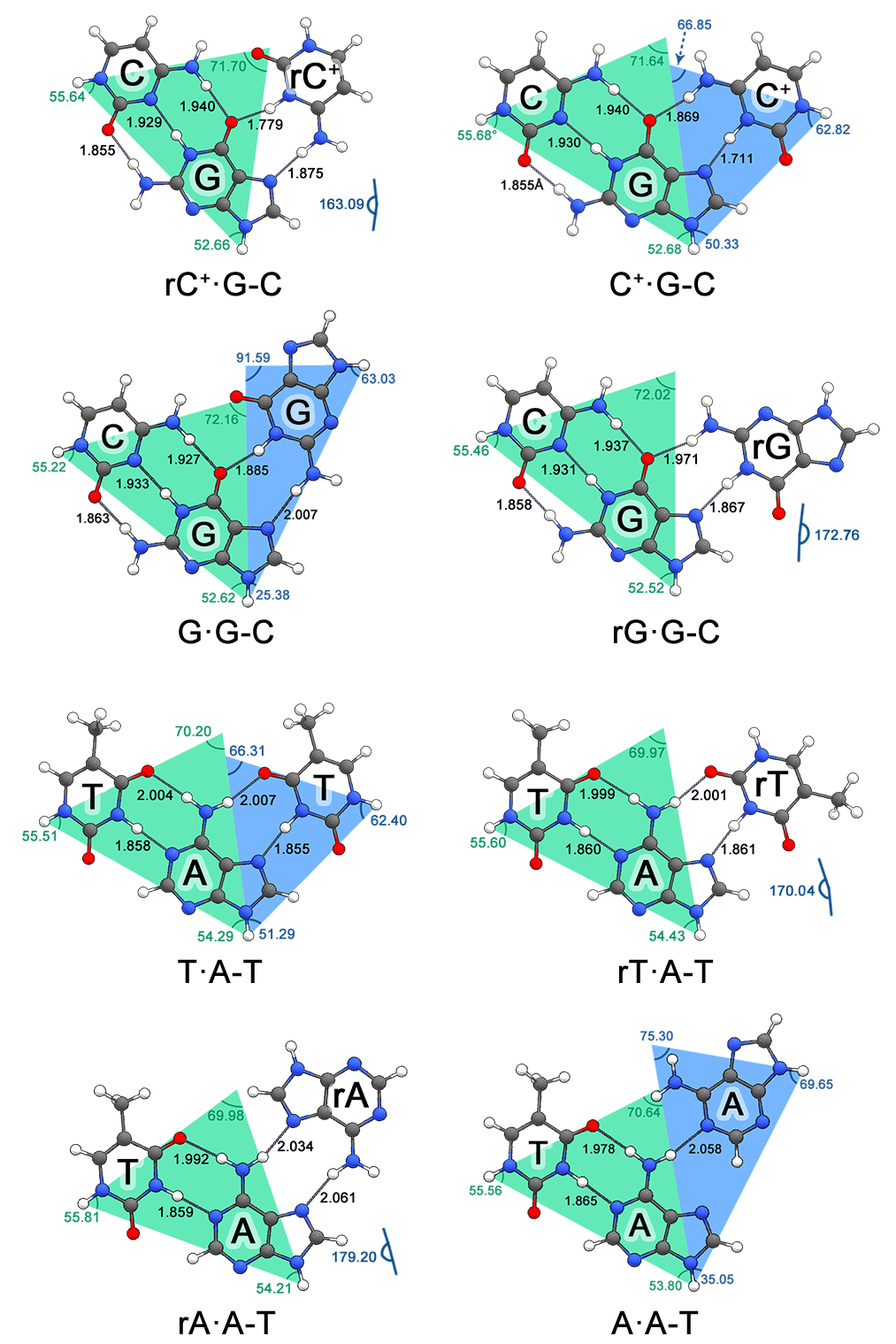
Figure S7. Optimized three-base/one-layer structures with HBLs (black) and angles containing GVAs of Watson-Crick pairs (green) and that of Hoogsteen pairs (blue) in the water phase.


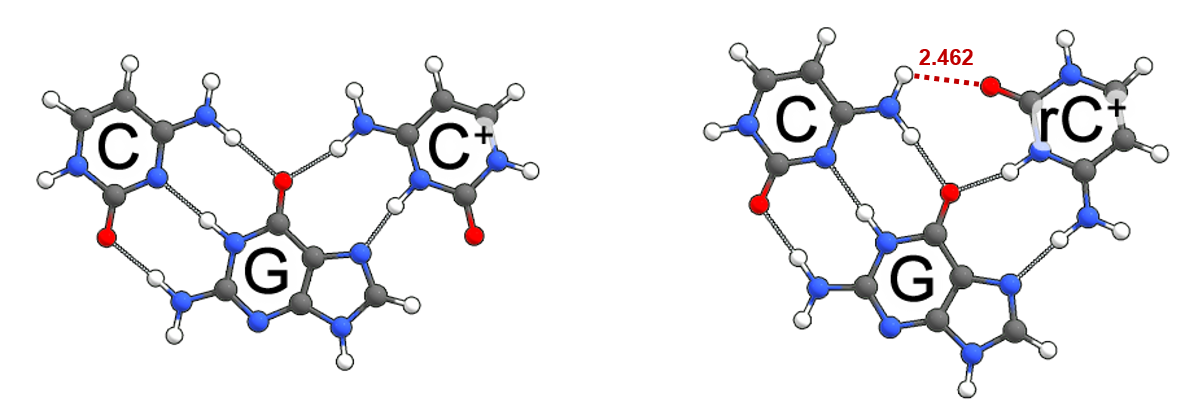

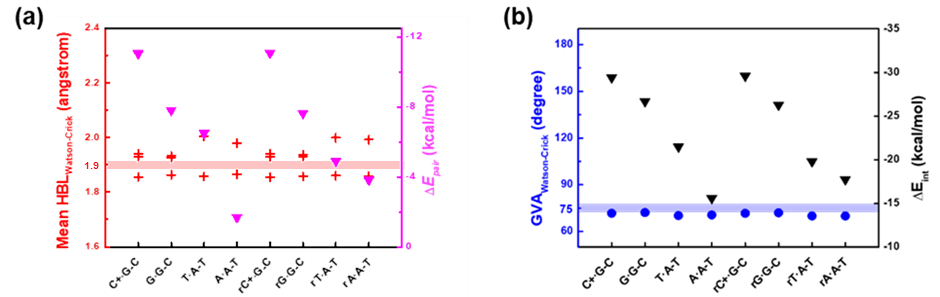
Figure S8. (a) HBL (red square) and (b) GVA (blue circle) of Watson-Crick in three-base/one-layer structures, along with Δ*E_pair_* (magenta inverted triangle) and Δ*E_int_* (black inverted triangle), respectively. Solid lines indicate the mean HBL and GVA of Watson-Crick base pairs obtained from crystallography data.

Figure S9. While C^+^ and C of C^+^•G-C does not have any hydrogen bond additionally, additional hydrogen bond between rC^+^ and C of rC^+^•G-C triad presumably can be formed.


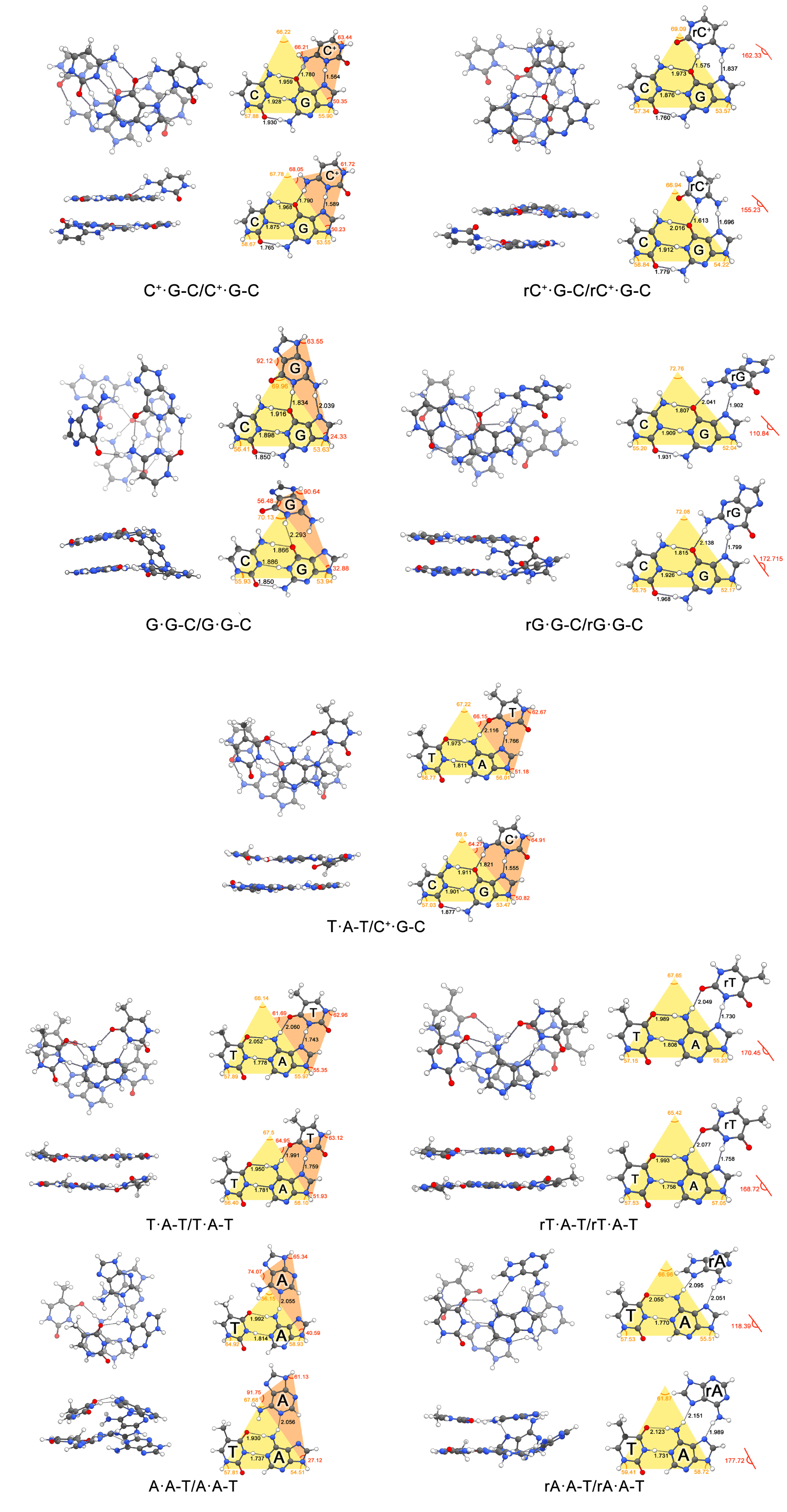


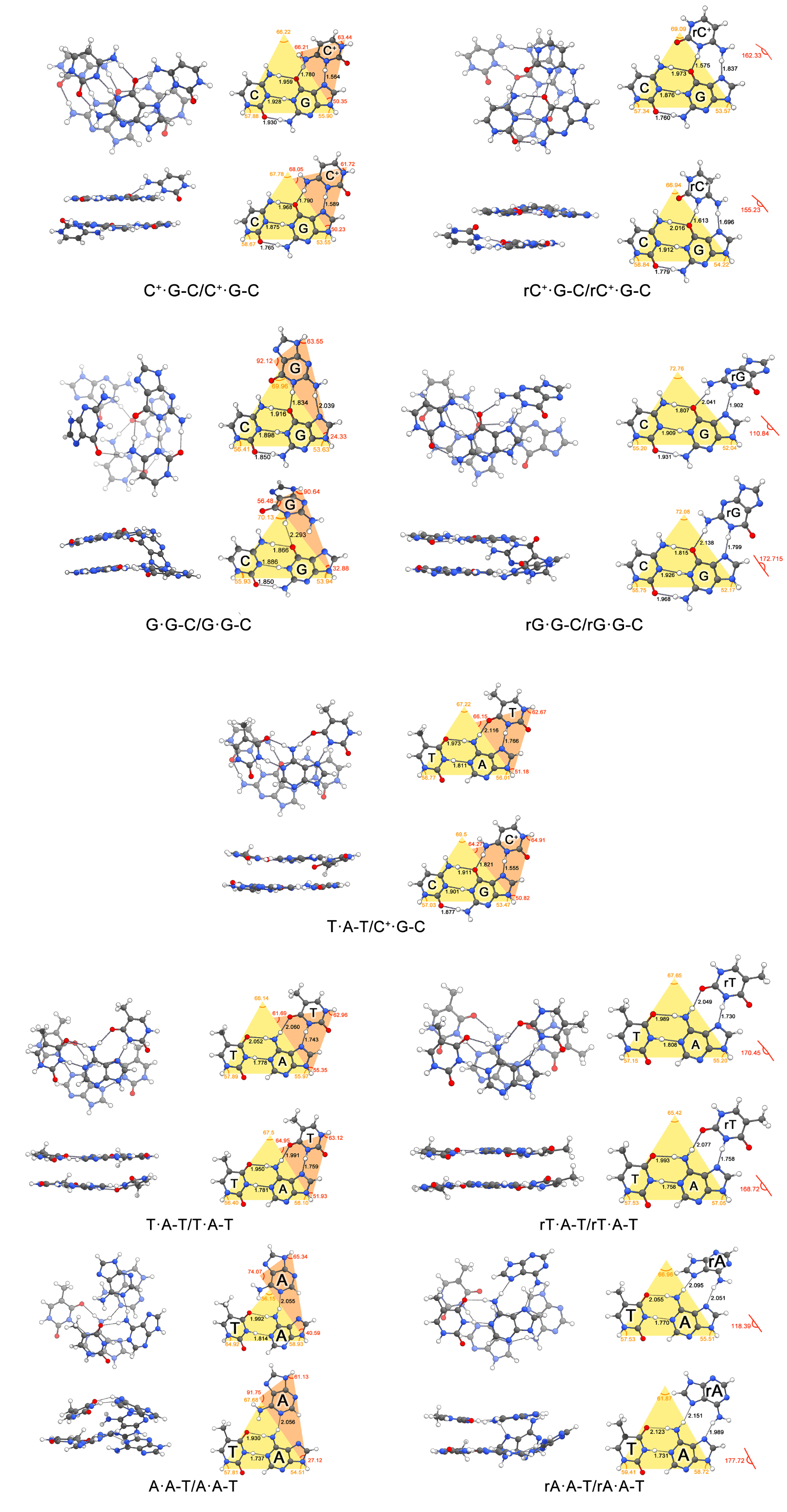
Figure S10. Optimized three-base/two-layer structures with HBLs (black) and angles containing GVAs of Watson-Crick pair (orange) and that of Hoogsteen base pairs (red) in the gas phase. Each structure includes of frontal and profile views of whole structures and frontal views of upper and lower layer.


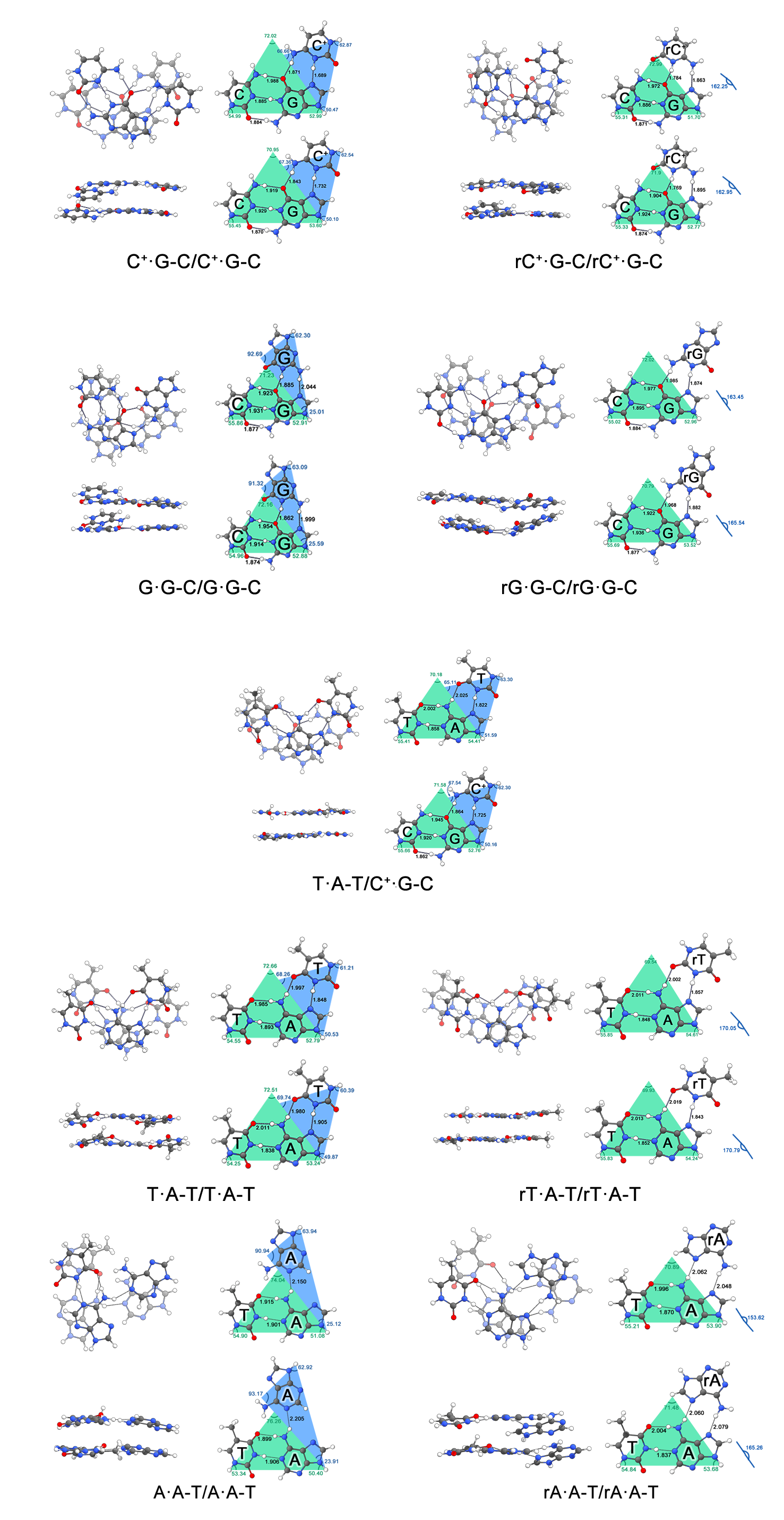


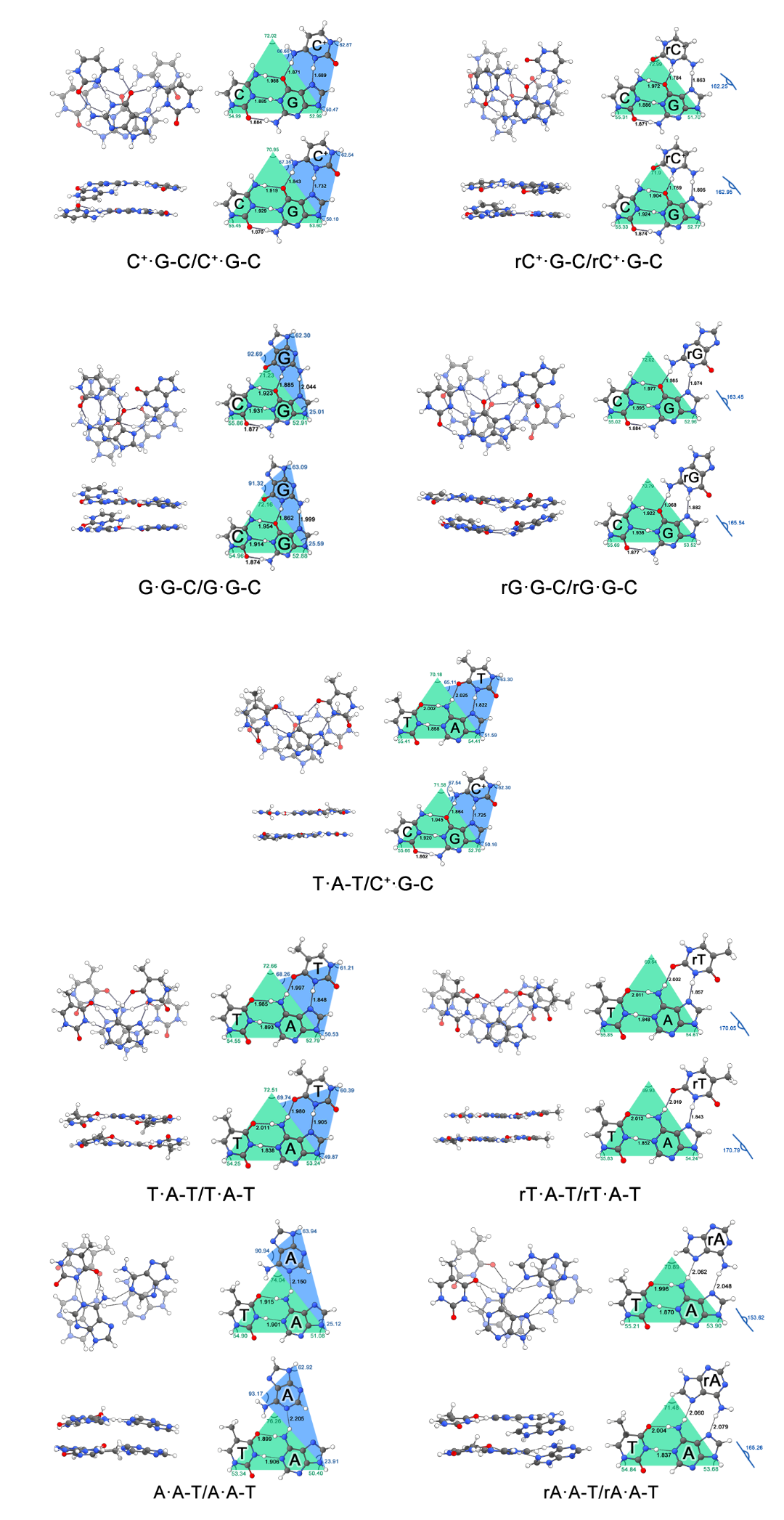
Figure S11. Optimized three-base/two-layer structures with HBLs (black) and angles containing GVAs of Watson-Crick pair (green) and that of Hoogsteen base pairs (blue) in the water phase. Each structure includes of frontal and profile views of whole structures and frontal views of upper and lower layer.


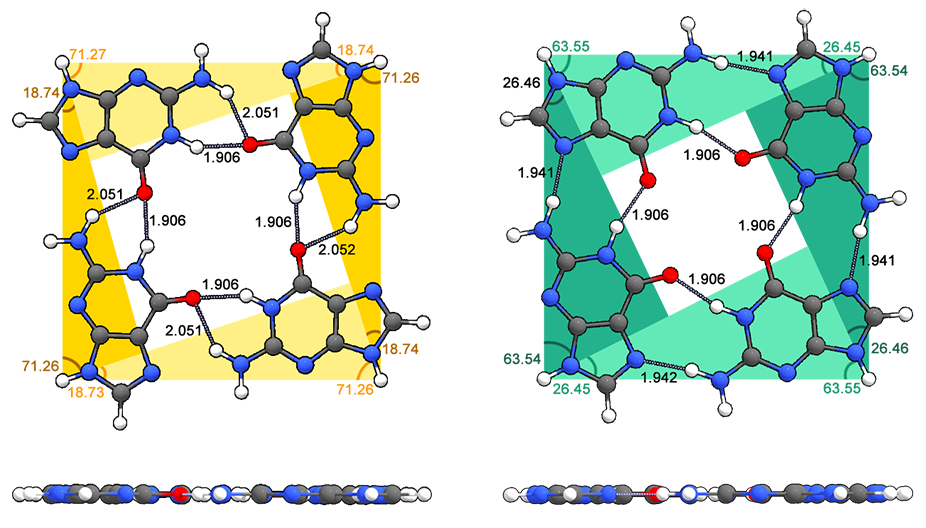


**(a)**

**(b)**

Figure S12. Optimized four-base/one-layer structures without metal ion in the gas (a) and water phase (b). Each structure contains of frontal and profile views. When the metal ion is absent in G-tetrad, the interactional formation of G-tetrad in the vacuum shows not the commonly-known Hoogsteen bonds but bifurcated hydrogen bonds. These bifurcated hydrogen bonds are speculated to be caused because the Van der Waals interaction and electrostatic interaction of Δ*G_solv_* in COSMO does not work. Compare to the G-tetrad in the water phase, pocket for containing metal ion becomes a more square-like structure and longer O···O length by the bifurcated hydrogen bonds. The larger pocket size for location of metal ion indicates more severe distortion of structures when the tetrads accept the metal ion.


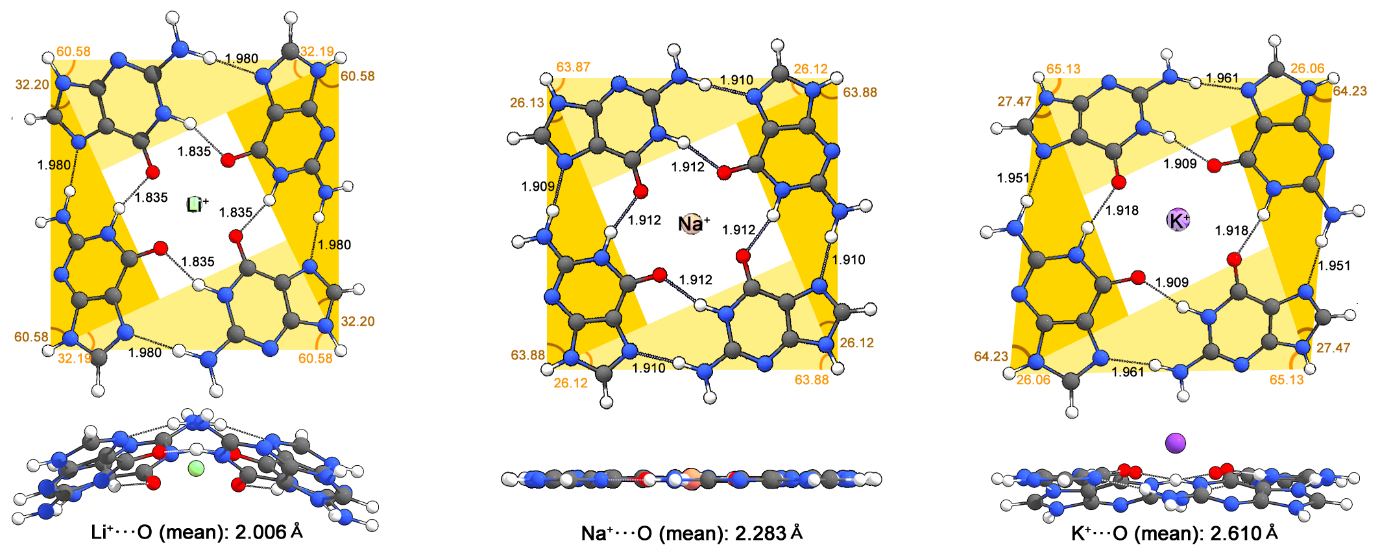
Figure S13. Optimized four-base/one-layer structures with metal ion in the gas phase. Initial models of G-tetrad before optimization are out-of-plane type. Each structure contains of frontal and profile views. The out-of-plane-like structure of G4∙∙Li^+^ is the result of severe distortion of guanines for accepting small Li ion. Thus, the structure of G4∙∙Li^+^ is precisely a distorted in-plane structure. Moreover, as we observed above in the G-tetrads without metal ion, the overall structures, especially G4∙∙K^+^, are severely distorted to be located metal ion in the center of pocket.


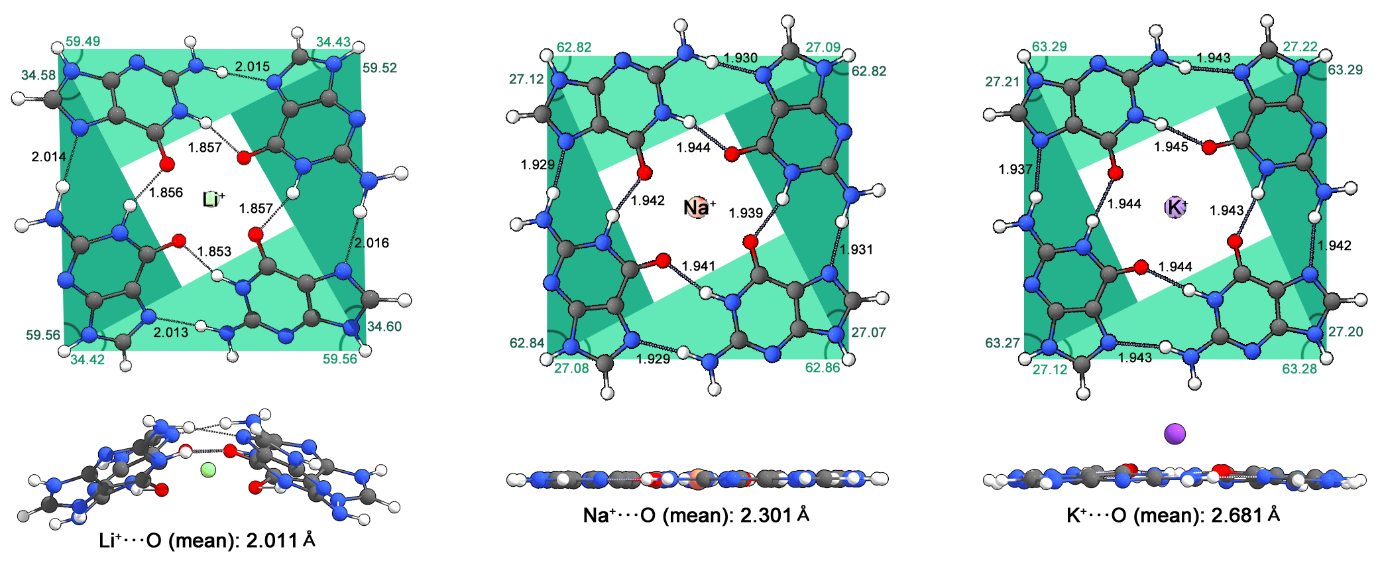


Figure S14. Optimized four-base/one-layer structures with metal ion in the water phase. Initial models of G-tatrad before optimization are out-of-plane type. Each structure contains of frontal and profile views. The distorted in-plane structure of G4∙∙Li^+^ can also observed in the water phase. However, the distortion of G4∙∙K^+^ decreased compared to one in the gas phase due to the water continuum.


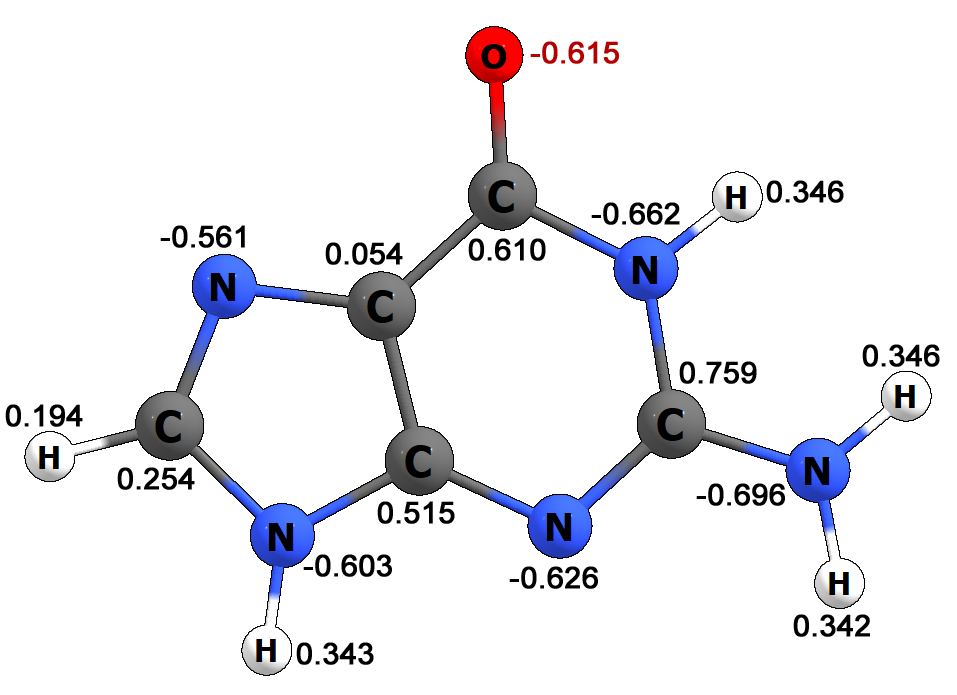
Figure S15. Mulliken charge of guanine in water phase by Gaussian 09.


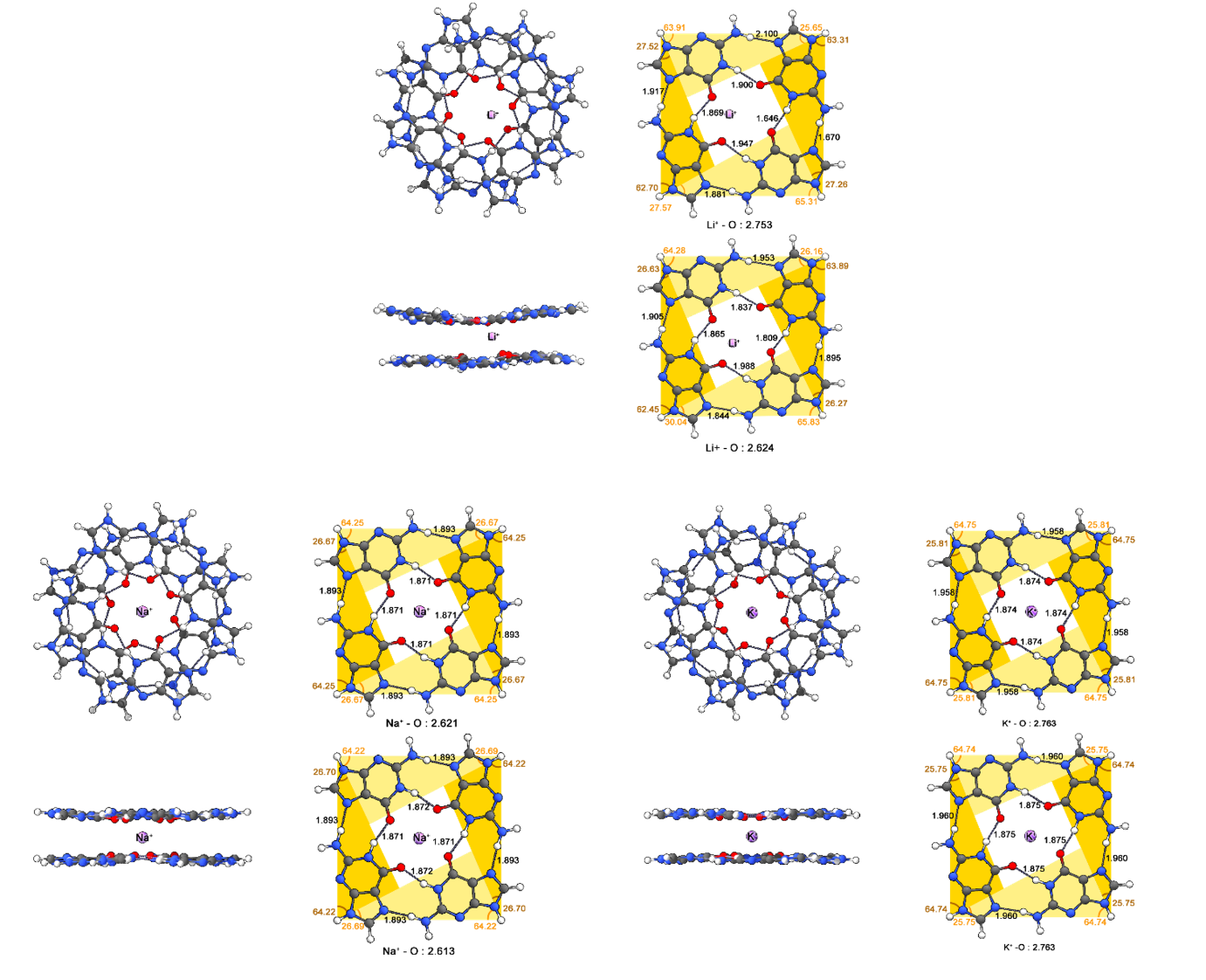


Figure S16. Optimized four-base/two-layer structures with metal ion in the gas phase. Initial position of metal ions before optimization are center of two layers. Each structure contains of frontal and profile views.


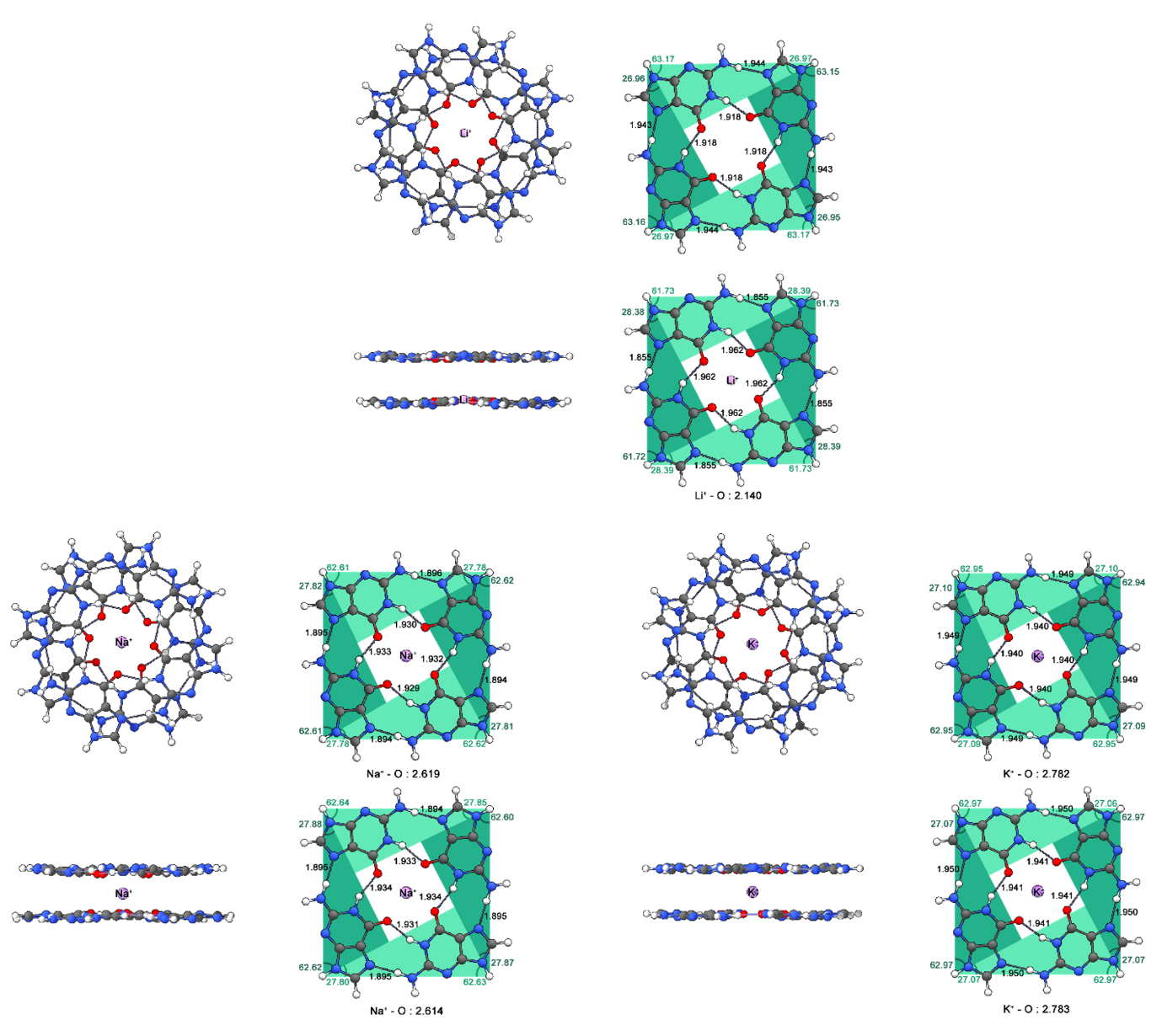
Figure S17. Optimized four-base/two-layer structures with metal ion in the water phase. Initial position of metal ions before optimization are center of two layers. Each structure contains of frontal and profile views.


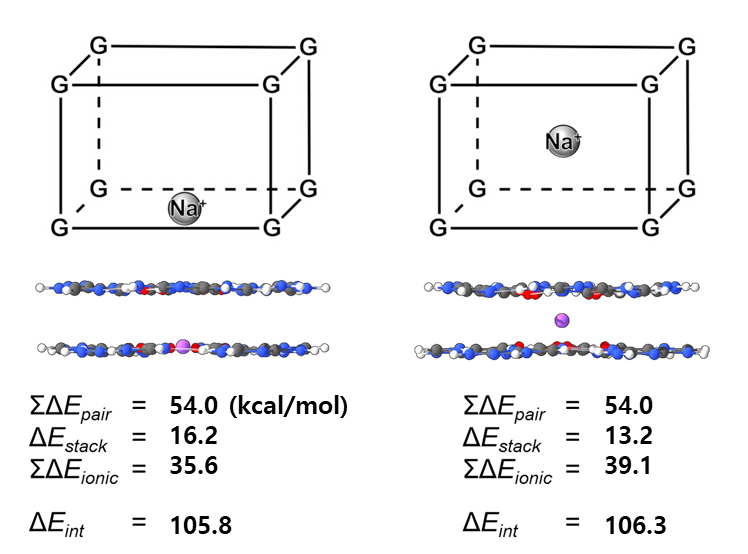
Figure S18. Optimized profile view of in-plane and out-of-plane G4∙∙∙Na^+^∙∙∙G4 structures and related energies.


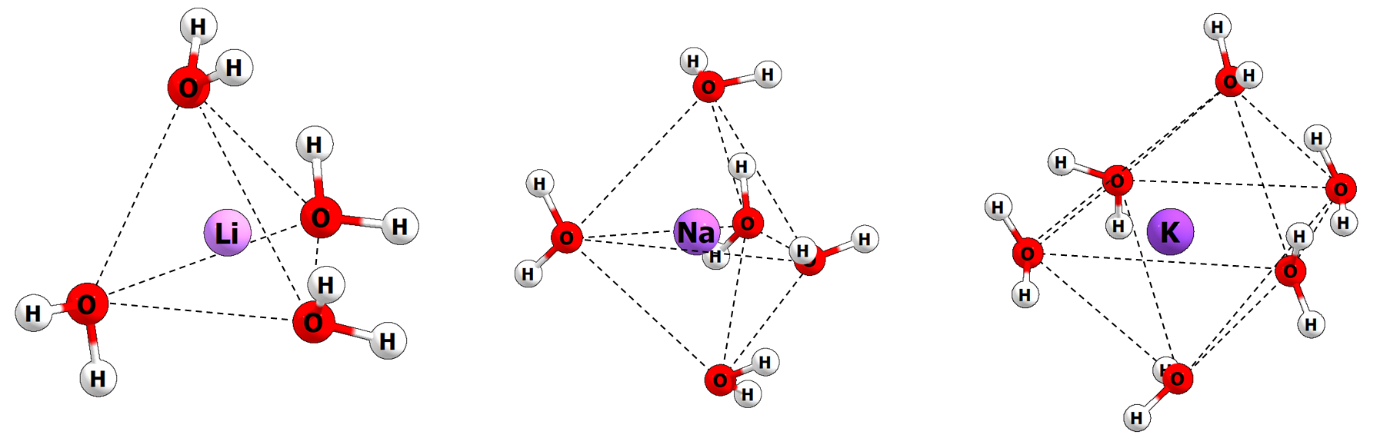


Figure S19. Optimized structure of hydrated metal ions. Each structure having coordination number 4, 5, and 6 displays the tetrahedral, trigonal bipyramidal, and octahedral structure, respectively.

Table S1. Interactions in all options, which are possible number of bases, number of layer, and phases.

|  |  |  | Gas phase | | | | | | | | | |  | | | Water phase | | | | | | | | | | |  |
| --- | --- | --- | --- | --- | --- | --- | --- | --- | --- | --- | --- | --- | --- | --- | --- | --- | --- | --- | --- | --- | --- | --- | --- | --- | --- | --- | --- |
|  |  |  | ΣΔ*E_pair_* | | ΣΔ*E_Hoogsteen_* | | ΣΔ*E_ionic_* | | ΣΔ*E_stack_* | | Σ*E_int_* | |  | | ΣΔ*E_pair_* | | | ΣΔ*E_Hoogsteen_* | | ΣΔ*E_ionic_* | | ΣΔ*E_stack_* | | Σ*E_int_* | |  |  |
| Duplex | One-layer | G-C | | -28.4 | |  | |  | |  | | -28.4 | |  | | | -11.4 | |  | |  | |  | | -11.4 | | |
|  |  | A-T | | -12.6 | |  | |  | |  | | -12.6 | |  | | | -6.80 | |  | |  | |  | | -6.8 | | |
|  |  | G-T | | -16.1 | |  | |  | |  | | -16.1 | |  | | | -6.77 | |  | |  | |  | | -6.8 | | |
|  |  | C-T | | -12.2 | |  | |  | |  | | -12.2 | |  | | | -6.7 | |  | |  | |  | | -6.7 | | |
|  |  | G-G | | -15.0 | |  | |  | |  | | -15.0 | |  | | | -6.6 | |  | |  | |  | | -6.6 | | |
|  |  | T-T | | -11.6 | |  | |  | |  | | -11.6 | |  | | | -6.43 | |  | |  | |  | | -6.43 | | |
|  |  | A-G | | -16.0 | |  | |  | |  | | -16.0 | |  | | | -6.41 | |  | |  | |  | | -6.41 | | |
|  |  | A-C | | -6.8 | |  | |  | |  | | -6.8 | |  | | | -3.4 | |  | |  | |  | | -3.4 | | |
|  |  | A-A | | -6.5 | |  | |  | |  | | -6.5 | |  | | | -3.25 | |  | |  | |  | | -3.2 | | |
|  |  | C-C | | -10.9 | |  | |  | |  | | -10.9 | |  | | | -3.16 | |  | |  | |  | | -3.2 | | |
|  | Two-layer | G-C/C-G | | -56.8 | |  | |  | | -24.1 | | -80.9 | |  | | | -22.9 | |  | |  | | -5.5 | | -28.3 | | |
|  |  | G-C/G-C | | -56.8 | |  | |  | | -19.3 | | -76.1 | |  | | | -22.9 | |  | |  | | -4.9 | | -27.8 | | |
|  |  | G-C/A-T | | -41.1 | |  | |  | | -13.8 | | -54.8 | |  | | | -18.3 | |  | |  | | -5.21 | | -23.5 | | |
|  |  | G-C/T-A | | -41.1 | |  | |  | | -21.3 | | -62.4 | |  | | | -18.3 | |  | |  | | -4.04 | | -22.3 | | |
|  |  | A-T/A-T | | -25.3 | |  | |  | | -15.4 | | -40.7 | |  | | | -13.6 | |  | |  | | -5.22 | | -18.83 | | |
|  |  | A-T/T-A | | -25.3 | |  | |  | | -12.1 | | -37.3 | |  | | | -13.6 | |  | |  | | -5.05 | | -18.65 | | |
| Triplex | One-layer | rC^+^•G-C | | -28.4 | | -50.2 | |  | |  | | -78.6 | |  | | | -11.4 | | -11.08 | |  | |  | | -22.5 | | |
|  |  | C^+^•G-C | | -28.4 | | -49.5 | |  | |  | | -77.9 | |  | | | -11.4 | | -11.04 | |  | |  | | -22.5 | | |
|  |  | G•G-C | | -28.4 | | -28.6 | |  | |  | | -57.0 | |  | | | -11.4 | | -7.81 | |  | |  | | -19.3 | | |
|  |  | rG•G-C | | -28.4 | | -23.3 | |  | |  | | -51.7 | |  | | | -11.4 | | -7.64 | |  | |  | | -19.1 | | |
|  |  | T•A-T | | -12.6 | | -16.8 | |  | |  | | -29.4 | |  | | | -6.8 | | -6.50 | |  | |  | | -13.3 | | |
|  |  | rT•A-T | | -12.6 | | -16.8 | |  | |  | | -29.4 | |  | | | -6.8 | | -4.90 | |  | |  | | -11.7 | | |
|  |  | rA•A-T | | -12.6 | | -15.8 | |  | |  | | -28.4 | |  | | | -6.8 | | -3.83 | |  | |  | | -10.6 | | |
|  |  | A•A-T | | -12.6 | | -12.3 | |  | |  | | -24.9 | |  | | | -6.8 | | -1.69 | |  | |  | | -8.5 | | |
|  | Two-layer | rC^+^•G-C/rC^+^•G-C | | -56.8 | | -100.3 | |  | | 17.7 | | -139.5 | |  | | | -22.9 | | -22.2 | |  | | -4.1 | | -49.2 | | |
|  |  | C^+^•G-C/C^+^•G-C | | -56.8 | | -99.0 | |  | | 18.4 | | -137.4 | |  | | | -22.9 | | -22.1 | |  | | -3.8 | | -48.8 | | |
|  |  | G•G-C/G•G-C | | -56.8 | | -57.2 | |  | | -22.6 | | -136.6 | |  | | | -22.9 | | -15.6 | |  | | -7.8 | | -46.3 | | |
|  |  | rG•G-C/rG•G-C | | -56.8 | | -46.6 | |  | | -22.1 | | -125.5 | |  | | | -11.4 | | -15.3 | |  | | -7.1 | | -33.8 | | |
|  |  | C^+^•G-C/T•A-T | | -41.1 | | -66.3 | |  | | -27.5 | | -134.9 | |  | | | -18.3 | | -17.5 | |  | | -8.6 | | -44.4 | | |
|  |  | rT•A-T/rT•A-T | | -25.3 | | -33.5 | |  | | -17.6 | | -76.4 | |  | | | -13.6 | | -9.8 | |  | | -13.1 | | -36.5 | | |
|  |  | T•A-T/T•A-T | | -25.3 | | -33.6 | |  | | -18.7 | | -77.5 | |  | | | -13.6 | | -13.0 | |  | | -7.4 | | -34.0 | | |
|  |  | rA•A-T/rA•A-T | | -25.3 | | -31.5 | |  | | -16.3 | | -73.1 | |  | | | -13.6 | | -7.7 | |  | | -12.2 | | -33.5 | | |
|  |  | A•A-T/A•A-T | | -25.3 | | -24.5 | |  | | -19.6 | | -69.4 | |  | | | -13.6 | | -3.4 | |  | | -11.9 | | -28.8 | | |
| Quadruplex | One-layer | G4∙∙∙Li^+^ | | -60.0 | |  | | -173.3 | |  | | -233.4 | |  | | | -26.3 | |  | | -31.3 | |  | | -57.6 | | |
|  |  | G4∙∙∙Na^+^ | | -60.0 | |  | | -153.0 | |  | | -213.0 | |  | | | -38.5 | |  | | -27.7 | |  | | -66.1 | | |
|  |  | G4∙∙∙K^+^ | | -60.0 | |  | | -123.3 | |  | | -183.3 | |  | | | -38.5 | |  | | -20.3 | |  | | -58.7 | | |
|  | Two-layer | G4∙∙∙Na^+^∙∙∙G4 | | -120.1 | |  | | -240.2 | | -11.2 | | -371.5 | |  | | | -52.7 | |  | | -39.1 | | -13.2 | | -152.2 | | |
|  |  | G4∙∙∙K^+^∙∙∙G4 | | -137.7 | |  | | -219.2 | | -11.2 | | -368.1 | |  | | | -52.7 | |  | | -37.0 | | -13.2 | | -151.7 | | |
|  |  | G4∙∙∙Li^+^∙∙∙G4 | | -137.7 | |  | | -234.2 | | -11.2 | | -383.2 | |  | | | -52.7 | |  | | -32.8 | | -13.2 | | -145.4 | | |

Table S2. The raw data of HBLs, GVAs, TA, their mean values, and standard deviations(s.d.) in the vacuum and the water phase. The GVA values are obtained by scalar product with glycosidic vectors (nitrogen and hydrogen atoms) of two nucleobases. The TA values also can be provided by scalar product of vectors. However, there is a difference that the two vectors along the hydrogen atoms on glycosidic vectors of upper and lower stacks are analyzed.

| **Gas phase** | **Duplex** | **One-layer** |  | G-C | A-T | G-T | C-T | G-G | T-T | A-G | A-C | A-A | C-C |  |  |  |  |  |  |  |  |
| --- | --- | --- | --- | --- | --- | --- | --- | --- | --- | --- | --- | --- | --- | --- | --- | --- | --- | --- | --- | --- | --- |
|  |  |  | HBL (WC) | 1.79 | 1.96 | 1.79 | 1.85 | 2.76 | 1.87 | 1.84 | 2.40 | 2.05 | 2.54 |  |  |  |  |  |  |  |  |
|  |  |  |  | 1.92 | 1.77 | 1.86 | 2.02 | 2.00 | 1.87 | 2.02 | 2.04 | 2.44 | 2.58 |  |  |  |  |  |  |  |  |
|  |  |  |  | 1.92 |  |  |  |  |  |  |  |  |  |  |  |  |  |  |  |  |  |
|  |  |  | mean HBL (WC) | 1.88 | 1.87 | 1.82 | 1.93 | 2.38 | 1.87 | 1.93 | 2.22 | 2.25 | 2.56 |  |  |  |  |  |  |  |  |
|  |  |  | HBL (WC) s.d. | 0.08 | 0.14 | 0.04 | 0.12 | 0.54 | 0.00 | 0.13 | 0.26 | 0.28 | 0.03 |  |  |  |  |  |  |  |  |
|  |  |  | GVA (WC) | 70.95 | 67.45 | 65.57 | 57.98 | 95.50 | 53.89 | 92.82 | 51.63 | 71.45 | 61.01 |  |  |  |  |  |  |  |  |
|  |  |  | Planarity | 0.00 | 0.00 | 0.07 | 0.00 | 0.00 | 0.00 | 0.00 | 0.00 | 0.01 | 0.00 |  |  |  |  |  |  |  |  |
|  |  | **Two-layer** |  | G-C/C-G | | G-C/G-C | | G-C/A-T | | G-C/T-A | | A-T/A-T | | A-T/T-A | |  |  |  |  |  |  |
|  |  |  | HBL (WC) | 1.86 | 1.86 | 1.87 | 1.83 | 1.79 | 2.07 | 1.82 | 1.97 | 1.99 | 1.98 | 1.96 | 1.96 |  |  |  |  |  |  |
|  |  |  |  | 1.94 | 1.94 | 1.96 | 1.93 | 1.91 | 1.74 | 1.96 | 1.85 | 1.74 | 1.82 | 1.80 | 1.80 |  |  |  |  |  |  |
|  |  |  |  | 1.85 | 1.85 | 1.93 | 1.99 | 1.92 |  | 1.91 |  |  |  |  |  |  |  |  |  |  |  |
|  |  |  | mean HBL (WC) | 1.89 | | 1.92 | | 1.89 | | 1.90 | | 1.88 | | 1.88 | |  |  |  |  |  |  |
|  |  |  | HBL (WC) s.d. | 0.05 | | 0.06 | | 0.13 | | 0.07 | | 0.12 | | 0.09 | |  |  |  |  |  |  |
|  |  |  | GVA (WC) | 57.45 | 57.67 | 68.99 | 58.36 | 69.01 | 65.27 | 61.39 | 56.40 | 65.64 | 68.93 | 66.93 | 66.83 |  |  |  |  |  |  |
|  |  |  | mean GVA (WC) | 57.56 | | 63.68 | | 67.14 | | 58.90 | | 67.29 | | 66.88 | |  |  |  |  |  |  |
|  |  |  | GVA (WC) s.d. | 0.16 | | 7.52 | | 2.64 | | 3.53 | | 2.33 | | 0.07 | |  |  |  |  |  |  |
|  |  |  | Planarity | 0.64 | 0.65 | 0.65 | 0.63 | 0.31 | 0.29 | 0.82 | 0.55 | 0.34 | 0.24 | 0.12 | 0.16 |  |  |  |  |  |  |
|  |  |  | mean planarity | 0.64 | | 0.64 | | 0.30 | | 0.69 | | 0.29 | | 0.14 | |  |  |  |  |  |  |
|  |  |  | planarity s.d. | 0.01 | | 0.02 | | 0.01 | | 0.19 | | 0.07 | | 0.03 | |  |  |  |  |  |  |
|  |  |  | TA | 55.54 | | 53.00 | | 36.14 | | 54.87 | | 46.48 | | 25.10 | |  |  |  |  |  |  |
|  | **Triplex** | **One-layer** |  | rC^+^•G-C | | C^+^•G-C | | G•G-C | | rG•G-C | | T•A-T | | rT•A-T | | rA•A-T | | A•A-T | |  |  |
|  |  |  | HBL (WC) | 1.96 | | 1.95 | | 1.87 | | 1.84 | | 2.00 | | 2.00 | | 1.97 | | 1.99 | |  |  |
|  |  |  |  | 1.89 | | 1.90 | | 1.89 | | 1.91 | | 1.78 | | 1.77 | | 1.78 | | 1.77 | |  |  |
|  |  |  |  | 1.77 | | 1.80 | | 1.85 | | 1.89 | |  | |  |  |  |  |  |  |  |  |
|  |  |  | mean HBL (WC) | 1.87 | | 1.88 | | 1.87 | | 1.88 | | 1.89 | | 1.89 | | 1.87 | | 1.88 | |  |  |
|  |  |  | HBL (WC) s.d. | 0.10 | | 0.07 | | 0.02 | | 0.04 | | 0.16 | | 0.16 | | 0.14 | | 0.16 | |  |  |
|  |  |  | HBL (HG) | 1.60 | | 1.79 | | 1.88 | | 2.14 | | 2.05 | | 2.06 | | 2.00 | | 2.00 | |  |  |
|  |  |  |  | 1.74 | | 1.54 | | 2.13 | | 1.80 | | 1.76 | | 1.76 | | 2.11 | |  |  |  |  |
|  |  |  | mean HBL (HG) | 1.67 | | 1.66 | | 2.00 | | 1.97 | | 1.90 | | 1.91 | | 2.06 | | 2.00 | |  |  |
|  |  |  | HBL (HG) s.d. | 0.10 | | 0.18 | | 0.18 | | 0.24 | | 0.20 | | 0.22 | | 0.08 | |  |  |  |  |
|  |  |  | GVA (WC) | 62.82 | | 68.52 | | 70.18 | | 71.66 | | 65.58 | | 65.63 | | 66.04 | | 66.82 | |  |  |
|  |  |  | GVA (HG) | 161.51 | | 65.33 | | 90.19 | | 170.22 | | 58.87 | | 170.95 | | 176.91 | | 78.36 | |  |  |
|  |  |  | Planarity | 0.00 | | 0.00 | | 0.00 | | 0.00 | | 0.00 | | 0.00 | | 0.00 | | 0.51 | |  |  |
|  |  | **Two-layer** |  | rC^+^•G-C  /rC^+^•G-C | | C^+^•G-C  /C^+^•G-C | | G•G-C  /G•G-C | | rG•G-C  /rG•G-C | | C^+^•G-C  /T•A-T | | rT•A-T  /rT•A-T | | T•A-T  /T•A-T | | rA•A-T  /rA•A-T | | A•A-T  /A•A-T | |
|  |  |  | HBL (WC) | 1.97 | 2.02 | 1.96 | 1.97 | 1.92 | 1.82 | 1.81 | 1.82 | 1.97 | 1.91 | 1.99 | 1.99 | 2.05 | 1.95 | 1.73 | 1.77 | 1.74 | 1.81 |
|  |  |  |  | 1.88 | 1.91 | 1.93 | 1.88 | 1.93 | 1.88 | 1.91 | 1.93 | 1.81 | 1.90 | 1.81 | 1.76 | 1.78 | 1.78 | 2.12 | 2.06 | 1.93 | 1.99 |
|  |  |  |  | 1.76 | 1.78 | 1.93 | 1.77 | 1.89 | 1.88 | 1.93 | 1.97 |  | 1.88 |  |  |  |  |  |  |  |  |
|  |  |  | mean HBL (WC) | 1.89 | | 1.90 | | 1.88 | | 1.89 | | 1.89 | | 1.89 | | 1.89 | | 1.92 | | 1.87 | |
|  |  |  | HBL (WC) s.d. | 0.10 | | 0.08 | | 0.04 | | 0.07 | | 0.06 | | 0.12 | | 0.13 | | 0.20 | | 0.11 | |
|  |  |  | HBL (HG) | 1.58 | 1.61 | 1.78 | 1.79 | 1.86 | 1.93 | 2.04 | 2.14 | 2.12 | 1.82 | 2.05 | 2.08 | 2.06 | 1.99 | 1.99 | 2.05 | 3.19 | 2.97 |
|  |  |  |  | 1.84 | 1.70 | 1.56 | 1.59 | 2.14 | 2.16 | 1.90 | 1.80 | 1.77 | 1.56 | 1.73 | 1.76 | 1.74 | 1.76 | 2.15 | 2.10 | 2.06 | 2.06 |
|  |  |  | mean HBL (HG) | 1.68 | | 1.68 | | 2.02 | | 1.97 | | 1.81 | | 1.90 | | 1.89 | | 2.07 | | 2.57 | |
|  |  |  | HBL (HG) s.d. | 0.12 | | 0.12 | | 0.15 | | 0.15 | | 0.23 | | 0.18 | | 0.16 | | 0.07 | | 0.60 | |
|  |  |  | GVA (WC) | 69.09 | 66.94 | 66.22 | 67.78 | 70.57 | 72.63 | 72.76 | 72.08 | 67.22 | 69.50 | 67.65 | 65.42 | 66.14 | 67.50 | 61.87 | 66.96 | 67.68 | 56.15 |
|  |  |  | mean GVA (WC) | 68.02 | | 67.00 | | 71.60 | | 72.42 | | 68.36 | | 66.54 | | 66.82 | | 64.42 | | 61.92 | |
|  |  |  | GVA (WC) s.d. | 1.52 | | 1.10 | | 1.46 | | 0.48 | | 1.61 | | 1.58 | | 0.96 | | 3.60 | | 8.15 | |
|  |  |  | GVA (HG) | 162.33 | 155.23 | 66.21 | 68.05 | 66.30 | 68.67 | 110.85 | 172.72 | 66.15 | 64.27 | 170.45 | 168.72 | 61.69 | 64.95 | 177.72 | 118.39 | 91.75 | 74.07 |
|  |  |  | mean GVA (HG) | 158.78 | | 67.13 | | 67.49 | | 141.78 | | 65.21 | | 169.59 | | 63.32 | | 148.06 | | 82.91 | |
|  |  |  | GVA (HG) s.d. | 5.02 | | 1.30 | | 1.68 | | 43.75 | | 1.33 | | 1.22 | | 2.31 | | 41.95 | | 12.50 | |
|  |  |  | Planarity | 0.47 | 0.16 | 0.58 | 0.93 | 1.10 | 0.30 | 1.08 | 0.56 | 0.35 | 0.20 | 0.21 | 0.21 | 0.42 | 0.17 | 0.63 | 1.41 | 0.93 | 1.04 |
|  |  |  | mean planarity | 0.31 | | 0.76 | | 0.70 | | 0.82 | | 0.27 | | 0.21 | | 0.30 | | 1.02 | | 0.99 | |
|  |  |  | planarity s.d. | 0.22 | | 0.24 | | 0.56 | | 0.37 | | 0.10 | | 0.00 | | 0.17 | | 0.55 | | 0.08 | |
|  |  |  | TA | 70.85 | | 17.13 | | 36.80 | | 40.38 | | 35.20 | | 25.63 | | 38.23 | | 52.59 | | 50.98 | |
|  | **Quadruplex** | **One-layer** |  | G4∙∙∙Li+ | | G4∙∙∙Na+ | | G4∙∙∙K+ | |  |  |  |  |  |  |  |  |  |  |  |  |
|  |  |  | NH-O | 1.84 | | 1.91 | | 1.91 | |  |  |  |  |  |  |  |  |  |  |  |  |
|  |  |  |  | 1.84 | | 1.91 | | 1.92 | |  |  |  |  |  |  |  |  |  |  |  |  |
|  |  |  |  | 1.84 | | 1.91 | | 1.91 | |  |  |  |  |  |  |  |  |  |  |  |  |
|  |  |  |  | 1.84 | | 1.91 | | 1.92 | |  |  |  |  |  |  |  |  |  |  |  |  |
|  |  |  | mean NH-O | 1.84 | | 1.91 | | 1.91 | |  |  |  |  |  |  |  |  |  |  |  |  |
|  |  |  | NH-O s.d. | 0.00 | | 0.00 | | 0.01 | |  |  |  |  |  |  |  |  |  |  |  |  |
|  |  |  | NH-N | 1.98 | | 1.91 | | 1.96 | |  |  |  |  |  |  |  |  |  |  |  |  |
|  |  |  |  | 1.98 | | 1.91 | | 1.95 | |  |  |  |  |  |  |  |  |  |  |  |  |
|  |  |  |  | 1.98 | | 1.91 | | 1.96 | |  |  |  |  |  |  |  |  |  |  |  |  |
|  |  |  |  | 1.98 | | 1.91 | | 1.95 | |  |  |  |  |  |  |  |  |  |  |  |  |
|  |  |  | mean NH-N | 1.98 | | 1.91 | | 1.96 | |  |  |  |  |  |  |  |  |  |  |  |  |
|  |  |  | NH-N s.d. | 0.00 | | 0.00 | | 0.01 | |  |  |  |  |  |  |  |  |  |  |  |  |
|  |  |  | GVA | 90.00 | | 90.01 | | 90.00 | |  |  |  |  |  |  |  |  |  |  |  |  |
|  |  |  |  | 90.00 | | 90.00 | | 90.00 | |  |  |  |  |  |  |  |  |  |  |  |  |
|  |  |  |  | 90.00 | | 90.00 | | 90.00 | |  |  |  |  |  |  |  |  |  |  |  |  |
|  |  |  |  | 89.98 | | 89.99 | | 90.00 | |  |  |  |  |  |  |  |  |  |  |  |  |
|  |  |  | mean GVA | 90.00 | | 90.00 | | 90.00 | |  |  |  |  |  |  |  |  |  |  |  |  |
|  |  |  | GVA s.d. | 0.01 | | 0.01 | | 0.00 | |  |  |  |  |  |  |  |  |  |  |  |  |
|  |  |  | Planarity | 0.94 | | 0.00 | | 0.49 | |  |  |  |  |  |  |  |  |  |  |  |  |
|  |  | **Two-layer** |  | G4∙∙∙Na^+^∙∙∙G4 | | G4∙∙∙K^+^∙∙∙G4 | | G4∙∙∙Li^+^∙∙∙G4 | |  |  |  |  |  |  |  |  |  |  |  |  |
|  |  |  | NH-O | 1.87 | 1.87 | 1.87 | 1.88 | 1.90 | 1.84 |  |  |  |  |  |  |  |  |  |  |  |  |
|  |  |  |  | 1.87 | 1.87 | 1.87 | 1.88 | 1.65 | 1.81 |  |  |  |  |  |  |  |  |  |  |  |  |
|  |  |  |  | 1.87 | 1.87 | 1.87 | 1.88 | 1.95 | 1.99 |  |  |  |  |  |  |  |  |  |  |  |  |
|  |  |  |  | 1.87 | 1.87 | 1.87 | 1.88 | 1.87 | 1.87 |  |  |  |  |  |  |  |  |  |  |  |  |
|  |  |  | mean NH-O | 1.87 | | 1.87 | | 1.86 | |  |  |  |  |  |  |  |  |  |  |  |  |
|  |  |  | NH-O s.d. | 0.00 | | 0.00 | | 0.12 | |  |  |  |  |  |  |  |  |  |  |  |  |
|  |  |  | NH-N | 1.89 | 1.89 | 1.96 | 1.96 | 2.10 | 1.95 |  |  |  |  |  |  |  |  |  |  |  |  |
|  |  |  |  | 1.89 | 1.89 | 1.96 | 1.96 | 1.67 | 1.90 |  |  |  |  |  |  |  |  |  |  |  |  |
|  |  |  |  | 1.89 | 1.89 | 1.96 | 1.96 | 1.88 | 1.84 |  |  |  |  |  |  |  |  |  |  |  |  |
|  |  |  |  | 1.89 | 1.89 | 1.96 | 1.96 | 1.92 | 1.91 |  |  |  |  |  |  |  |  |  |  |  |  |
|  |  |  | mean NH-N | 1.89 | | 1.96 | | 1.90 | |  |  |  |  |  |  |  |  |  |  |  |  |
|  |  |  | NH-N s.d. | 0.00 | | 0.00 | | 0.12 | |  |  |  |  |  |  |  |  |  |  |  |  |
|  |  |  | GVA | 89.08 | 89.09 | 89.44 | 89.51 | 90.44 | 89.56 |  |  |  |  |  |  |  |  |  |  |  |  |
|  |  |  |  | 89.08 | 89.08 | 89.44 | 89.51 | 89.11 | 89.84 |  |  |  |  |  |  |  |  |  |  |  |  |
|  |  |  |  | 89.08 | 89.09 | 89.44 | 89.51 | 87.12 | 84.13 |  |  |  |  |  |  |  |  |  |  |  |  |
|  |  |  |  | 89.08 | 89.08 | 89.44 | 89.51 | 89.78 | 90.92 |  |  |  |  |  |  |  |  |  |  |  |  |
|  |  |  | mean GVA | 89.08 | | 89.48 | | 88.86 | |  |  |  |  |  |  |  |  |  |  |  |  |
|  |  |  | GVA s.d. | 0.00 | | 0.04 | | 2.22 | |  |  |  |  |  |  |  |  |  |  |  |  |
|  |  |  | Planarity | 0.30 | 0.21 | 0.15 | 0.15 | 0.11 | 0.10 |  |  |  |  |  |  |  |  |  |  |  |  |
|  |  |  | mean planarity | 0.26 | | 0.15 | | 0.10 | |  |  |  |  |  |  |  |  |  |  |  |  |
|  |  |  | planarity s.d. | 0.06 | | 0.00 | | 0.00 | |  |  |  |  |  |  |  |  |  |  |  |  |
|  |  |  | TA | 45.00 | | 44.95 | | 45.78 | |  |  |  |  |  |  |  |  |  |  |  |  |

| **Water phase** | **Duplex** | **One-layer** |  | G-C | A-T | **G-T** | C-T | **G-G** | T-T | A-G | A-C | A-A | C-C |  |  |  |  |  |  |  |  |
| --- | --- | --- | --- | --- | --- | --- | --- | --- | --- | --- | --- | --- | --- | --- | --- | --- | --- | --- | --- | --- | --- |
|  |  |  | HBL (WC) | 1.92 | 1.99 | 1.88 | 1.90 | 1.92 | 1.87 | 1.89 | 2.06 | 2.03 | 2.07 |  |  |  |  |  |  |  |  |
|  |  |  |  | 1.94 | 1.85 | 1.88 | 2.06 | 2.02 | 1.88 | 2.07 | 2.59 | 2.66 |  |  |  |  |  |  |  |  |  |
|  |  |  |  | 1.87 |  |  |  |  |  |  |  |  |  |  |  |  |  |  |  |  |  |
|  |  |  | mean HBL (WC) | 1.91 | 1.92 | 1.88 | 1.98 | 1.97 | 1.88 | 1.98 | 2.32 | 2.34 | 2.07 |  |  |  |  |  |  |  |  |
|  |  |  | HBL (WC) s.d. | 0.03 | 0.10 | 0.00 | 0.11 | 0.07 | 0.00 | 0.13 | 0.37 | 0.44 |  |  |  |  |  |  |  |  |  |
|  |  |  | GVA (WC) | 72.08 | 70.63 | 71.81 | 60.12 | 90.66 | 56.78 | 95.43 | 58.77 | 79.55 | 65.32 |  |  |  |  |  |  |  |  |
|  |  |  | Planarity | 0.00 | 0.00 | 0.00 | 0.00 | 0.00 | 0.00 | 0.00 | 0.00 | 0.01 | 0.00 |  |  |  |  |  |  |  |  |
|  |  | **Two-layer** |  | G-C/C-G | | G-C/G-C | | G-C/A-T | | G-C/T-A | | A-T/A-T | | A-T/T-A | |  |  |  |  |  |  |
|  |  |  | HBL (WC) | 1.92 | 1.92 | 1.92 | 1.90 | 1.92 | 2.02 | 1.91 | 1.98 | 2.02 | 1.97 | 2.02 | 1.96 |  |  |  |  |  |  |
|  |  |  |  | 1.93 | 1.94 | 1.94 | 1.92 | 1.91 | 1.84 | 1.94 | 1.78 | 1.83 | 1.88 | 1.85 | 1.84 |  |  |  |  |  |  |
|  |  |  |  | 1.87 | 1.87 | 1.92 | 1.91 | 1.91 |  | 1.88 |  |  |  |  |  |  |  |  |  |  |  |
|  |  |  | mean HBL (WC) | 1.91 | | 1.92 | | 1.92 | | 1.90 | | 1.93 | | 1.92 | |  |  |  |  |  |  |
|  |  |  | HBL (WC) s.d. | 0.03 | | 0.01 | | 0.07 | | 0.08 | | 0.09 | | 0.09 | |  |  |  |  |  |  |
|  |  |  | GVA (WC) | 71.92 | 71.88 | 72.12 | 73.04 | 72.13 | 70.53 | 72.25 | 70.52 | 70.71 | 72.56 | 70.90 | 71.95 |  |  |  |  |  |  |
|  |  |  | mean GVA (WC) | 71.90 | | 72.58 | | 71.33 | | 71.68 | | 71.64 | | 71.43 | |  |  |  |  |  |  |
|  |  |  | GVA (WC) s.d. | 0.03 | | 0.65 | | 1.13 | | 0.33 | | 1.31 | | 0.74 | |  |  |  |  |  |  |
|  |  |  | Planarity | 0.19 | 0.22 | 0.21 | 0.27 | 0.23 | 0.27 | 0.19 | 0.15 | 0.23 | 0.17 | 0.18 | 0.20 |  |  |  |  |  |  |
|  |  |  | mean planarity | 0.20 | | 0.24 | | 0.25 | | 0.17 | | 0.20 | | 0.19 | |  |  |  |  |  |  |
|  |  |  | planarity s.d. | 0.02 | | 0.04 | | 0.02 | | 0.03 | | 0.04 | | 0.02 | |  |  |  |  |  |  |
|  |  |  | TA | 20.37 | | 33.63 | | 36.81 | | 22.92 | | 37.44 | | 23.73 | |  |  |  |  |  |  |
|  | **Triplex** | **One-layer** |  | rC^+^•G-C | | C^+^•G-C | | G•G-C | | rG•G-C | | T•A-T | | rT•A-T | | rA•A-T | | A•A-T | |  |  |
|  |  |  | HBL (WC) | 1.94 | | 1.94 | | 1.93 | | 1.94 | | 2.00 | | 2.00 | | 1.99 | | 1.98 | |  |  |
|  |  |  |  | 1.93 | | 1.93 | | 1.93 | | 1.93 | | 1.86 | | 1.86 | | 1.86 | | 1.87 | |  |  |
|  |  |  |  | 1.86 | | 1.86 | | 1.86 | | 1.86 | |  | |  | |  | |  | |  |  |
|  |  |  | mean HBL (WC) | 1.91 | | 1.91 | | 1.91 | | 1.91 | | 1.93 | | 1.93 | | 1.93 | | 1.92 | |  |  |
|  |  |  | HBL (WC) s.d. | 0.05 | | 0.05 | | 0.04 | | 0.04 | | 0.10 | | 0.10 | | 0.09 | | 0.08 | |  |  |
|  |  |  | HBL (HG) | 1.78 | | 1.87 | | 1.89 | | 1.97 | | 2.01 | | 2.00 | | 2.03 | | 2.06 | |  |  |
|  |  |  |  | 1.88 | | 1.71 | | 2.01 | | 1.87 | | 1.89 | | 1.86 | | 2.06 | |  | |  |  |
|  |  |  | mean HBL (HG) | 1.83 | | 1.79 | | 1.95 | | 1.92 | | 1.95 | | 1.93 | | 2.05 | | 2.06 | |  |  |
|  |  |  | HBL (HG) s.d. | 0.07 | | 0.11 | | 0.09 | | 0.07 | | 0.09 | | 0.10 | | 0.02 | |  | |  |  |
|  |  |  | GVA (WC) | 71.70 | | 71.64 | | 72.16 | | 72.02 | | 70.20 | | 69.97 | | 69.98 | | 70.64 | |  |  |
|  |  |  | GVA (HG) | 163.09 | | 66.85 | | 91.59 | | 172.76 | | 66.31 | | 170.04 | | 179.20 | | 75.30 | |  |  |
|  |  |  | Planarity | 0.00 | | 0.00 | | 0.00 | | 0.00 | | 0.06 | | 0.00 | | 0.00 | | 0.00 | |  |  |
|  |  | **Two-layer** |  | rC^+^•G-C/rC^+^•G-C | | C^+^•G-C/C^+^•G-C | | G•G-C/G•G-C | | rG•G-C/rG•G-C | | C^+^•G-C/T•A-T | | rT•A-T/rT•A-T | | T•A-T/T•A-T | | rA•A-T/rA•A-T | | A•A-T/A•A-T | |
|  |  |  | HBL (WC) | 1.97 | 1.90 | 1.99 | 1.92 | 1.92 | 1.95 | 1.98 | 1.92 | 2.00 | 1.95 | 2.01 | 2.01 | 1.99 | 2.01 | 2.00 | 2.00 | 1.91 | 1.92 |
|  |  |  |  | 1.89 | 1.92 | 1.89 | 1.93 | 1.93 | 1.91 | 1.90 | 1.94 | 1.86 | 1.92 | 1.85 | 1.85 | 1.89 | 1.84 | 1.87 | 1.84 | 1.90 | 1.90 |
|  |  |  |  | 1.87 | 1.87 | 1.88 | 1.87 | 1.88 | 1.87 | 1.88 | 1.88 |  | 1.86 |  |  |  |  |  |  |  |  |
|  |  |  | mean HBL (WC) | 1.91 | | 1.91 | | 1.91 | | 1.92 | | 1.92 | | 1.93 | | 1.93 | | 1.93 | | 1.91 | |
|  |  |  | HBL (WC) s.d. | 0.04 | | 0.04 | | 0.03 | | 0.04 | | 0.06 | | 0.09 | | 0.08 | | 0.09 | | 0.01 | |
|  |  |  | HBL (HG) | 1.78 | 1.77 | 1.87 | 1.84 | 1.89 | 1.86 | 1.99 | 1.96 | 2.03 | 1.86 | 2.00 | 2.02 | 2.00 | 1.98 | 2.06 | 2.06 | 2.21 | 2.15 |
|  |  |  |  | 1.86 | 1.90 | 1.69 | 1.73 | 2.04 | 2.00 | 1.87 | 1.88 | 1.82 | 1.73 | 1.86 | 1.84 | 1.85 | 1.91 | 2.05 | 2.08 |  |  |
|  |  |  | mean HBL (HG) | 1.83 | | 1.78 | | 1.95 | | 1.92 | | 1.86 | | 1.93 | | 1.93 | | 2.06 | | 2.18 | |
|  |  |  | HBL (HG) s.d. | 0.06 | | 0.09 | | 0.09 | | 0.06 | | 0.13 | | 0.09 | | 0.07 | | 0.01 | | 0.04 | |
|  |  |  | GVA (WC) | 72.99 | 71.90 | 72.02 | 70.95 | 71.23 | 72.16 | 72.02 | 70.79 | 72.02 | 70.95 | 69.54 | 69.93 | 72.66 | 72.51 | 70.90 | 71.48 | 76.26 | 74.02 |
|  |  |  | mean GVA (WC) | 72.45 | | 71.49 | | 71.70 | | 71.41 | | 71.49 | | 69.74 | | 72.59 | | 71.19 | | 75.14 | |
|  |  |  | GVA (WC) s.d. | 0.77 | | 0.76 | | 0.66 | | 0.87 | | 0.76 | | 0.28 | | 0.11 | | 0.41 | | 1.58 | |
|  |  |  | GVA (HG) | 162.25 | 162.95 | 66.66 | 67.36 | 92.69 | 91.32 | 163.45 | 165.54 | 66.66 | 67.36 | 170.05 | 170.79 | 68.26 | 69.74 | 153.62 | 165.26 | 93.17 | 90.94 |
|  |  |  | mean GVA (HG) | 162.60 | | 67.01 | | 92.01 | | 164.49 | | 67.01 | | 170.42 | | 69.00 | | 159.44 | | 92.06 | |
|  |  |  | GVA (HG) s.d. | 0.49 | | 0.49 | | 0.97 | | 1.48 | | 0.49 | | 0.52 | | 1.05 | | 8.23 | | 1.58 | |
|  |  |  | Planarity | 0.33 | 0.54 | 0.72 | 0.42 | 0.71 | 0.67 | 0.78 | 0.58 | 0.20 | 0.10 | 0.31 | 0.35 | 0.43 | 0.38 | 0.64 | 0.52 | 0.59 | 0.55 |
|  |  |  | mean planarity | 0.43 | | 0.57 | | 0.69 | | 0.68 | | 0.15 | | 0.33 | | 0.40 | | 0.58 | | 0.57 | |
|  |  |  | planarity s.d. | 0.15 | | 0.21 | | 0.03 | | 0.15 | | 0.07 | | 0.03 | | 0.04 | | 0.09 | | 0.03 | |
|  |  |  | TA | 48.40 | | 35.26 | | 28.18 | | 36.52 | | 21.26 | | 3.44 | | 32.88 | | 28.10 | | 24.64 | |
|  | **Quadruplex** | **One-layer** |  | G4∙∙∙Li+ | | G4∙∙∙Na+ | | G4∙∙∙K+ | |  |  |  |  |  |  |  |  |  |  |  |  |
|  |  |  | N...H-O | 1.86 | | 1.94 | | 1.95 | |  |  |  |  |  |  |  |  |  |  |  |  |
|  |  |  |  | 1.86 | | 1.94 | | 1.94 | |  |  |  |  |  |  |  |  |  |  |  |  |
|  |  |  |  | 1.85 | | 1.94 | | 1.94 | |  |  |  |  |  |  |  |  |  |  |  |  |
|  |  |  |  | 1.86 | | 1.94 | | 1.94 | |  |  |  |  |  |  |  |  |  |  |  |  |
|  |  |  | mean N-H...O | 1.86 | | 1.94 | | 1.94 | |  |  |  |  |  |  |  |  |  |  |  |  |
|  |  |  | N-H...O s.d. | 0.00 | | 0.00 | | 0.00 | |  |  |  |  |  |  |  |  |  |  |  |  |
|  |  |  | N...H-N | 2.02 | | 1.93 | | 1.94 | |  |  |  |  |  |  |  |  |  |  |  |  |
|  |  |  |  | 2.02 | | 1.93 | | 1.94 | |  |  |  |  |  |  |  |  |  |  |  |  |
|  |  |  |  | 2.01 | | 1.93 | | 1.94 | |  |  |  |  |  |  |  |  |  |  |  |  |
|  |  |  |  | 2.01 | | 1.93 | | 1.94 | |  |  |  |  |  |  |  |  |  |  |  |  |
|  |  |  | mean N-H...N | 2.01 | | 1.93 | | 1.94 | |  |  |  |  |  |  |  |  |  |  |  |  |
|  |  |  | N-H...N s.d. | 0.00 | | 0.00 | | 0.00 | |  |  |  |  |  |  |  |  |  |  |  |  |
|  |  |  | GVA | 86.08 | | 90.09 | | 89.49 | |  |  |  |  |  |  |  |  |  |  |  |  |
|  |  |  |  | 85.86 | | 90.11 | | 89.52 | |  |  |  |  |  |  |  |  |  |  |  |  |
|  |  |  |  | 86.02 | | 90.06 | | 89.60 | |  |  |  |  |  |  |  |  |  |  |  |  |
|  |  |  |  | 85.88 | | 90.04 | | 89.51 | |  |  |  |  |  |  |  |  |  |  |  |  |
|  |  |  | mean GVA | 85.96 | | 90.08 | | 89.53 | |  |  |  |  |  |  |  |  |  |  |  |  |
|  |  |  | GVA s.d. | 0.11 | | 0.03 | | 0.05 | |  |  |  |  |  |  |  |  |  |  |  |  |
|  |  |  | Planarity | 1.04 | | 0.12 | | 0.12 | |  |  |  |  |  |  |  |  |  |  |  |  |
|  |  | **Two-layer** |  | G4∙∙∙Na^+^∙∙∙G4 | | G4∙∙∙K^+^∙∙∙G4 | | G4∙∙∙Li^+^∙∙∙G4 | |  |  |  |  |  |  |  |  |  |  |  |  |
|  |  |  | N...H-O | 1.93 | 1.93 | 1.94 | 1.94 | 1.92 | 1.96 |  |  |  |  |  |  |  |  |  |  |  |  |
|  |  |  |  | 1.93 | 1.93 | 1.94 | 1.94 | 1.92 | 1.96 |  |  |  |  |  |  |  |  |  |  |  |  |
|  |  |  |  | 1.93 | 1.93 | 1.94 | 1.94 | 1.92 | 1.96 |  |  |  |  |  |  |  |  |  |  |  |  |
|  |  |  |  | 1.93 | 1.93 | 1.94 | 1.94 | 1.92 | 1.96 |  |  |  |  |  |  |  |  |  |  |  |  |
|  |  |  | mean N-H...O | 1.93 | | 1.94 | | 1.94 | |  |  |  |  |  |  |  |  |  |  |  |  |
|  |  |  | N-H...O s.d. | 0.00 | | 0.00 | | 0.02 | |  |  |  |  |  |  |  |  |  |  |  |  |
|  |  |  | N...H-N | 1.90 | 1.87 | 1.95 | 1.95 | 1.94 | 1.86 |  |  |  |  |  |  |  |  |  |  |  |  |
|  |  |  |  | 1.89 | 1.90 | 1.95 | 1.95 | 1.94 | 1.86 |  |  |  |  |  |  |  |  |  |  |  |  |
|  |  |  |  | 1.89 | 1.90 | 1.95 | 1.95 | 1.94 | 1.86 |  |  |  |  |  |  |  |  |  |  |  |  |
|  |  |  |  | 1.90 | 1.90 | 1.95 | 1.95 | 1.94 | 1.86 |  |  |  |  |  |  |  |  |  |  |  |  |
|  |  |  | mean N-H...N | 1.89 | | 1.95 | | 1.90 | |  |  |  |  |  |  |  |  |  |  |  |  |
|  |  |  | N-H...N s.d. | 0.01 | | 0.00 | | 0.05 | |  |  |  |  |  |  |  |  |  |  |  |  |
|  |  |  | GVA | 89.61 | 89.51 | 89.95 | 89.97 | 89.86 | 89.88 |  |  |  |  |  |  |  |  |  |  |  |  |
|  |  |  |  | 89.57 | 89.53 | 89.97 | 89.96 | 89.88 | 89.88 |  |  |  |  |  |  |  |  |  |  |  |  |
|  |  |  |  | 89.60 | 89.57 | 89.96 | 89.96 | 89.86 | 89.88 |  |  |  |  |  |  |  |  |  |  |  |  |
|  |  |  |  | 89.57 | 89.50 | 89.95 | 89.96 | 89.95 | 89.90 |  |  |  |  |  |  |  |  |  |  |  |  |
|  |  |  | mean GVA | 89.56 | | 89.96 | | 89.89 | |  |  |  |  |  |  |  |  |  |  |  |  |
|  |  |  | GVA s.d. | 0.04 | | 0.01 | | 0.03 | |  |  |  |  |  |  |  |  |  |  |  |  |
|  |  |  | Planarity | 0.12 | 0.12 | 0.06 | 0.06 | 0.08 | 0.10 |  |  |  |  |  |  |  |  |  |  |  |  |
|  |  |  | mean planarity | 0.12 | | 0.06 | | 0.09 | |  |  |  |  |  |  |  |  |  |  |  |  |
|  |  |  | planarity s.d. | 0.00 | | 0.00 | | 0.01 | |  |  |  |  |  |  |  |  |  |  |  |  |
|  |  |  | TA | 44.85 | | 45.16 | | 44.11 | |  |  |  |  |  |  |  |  |  |  |  |  |

Table S3. Experimentally-determined HBL data of duplex from Cartesian coordinates of RCSB PDB structures. From PDB files, we extracted the Cartesian coordinates of atoms, which are typically described as four significant figures because the crystal structure analysis cannot provide more than three decimal place accuracy due to the limitation of resolution.

| **PDB ID** |  | **Pair** |  | **HBL (angstrom)** | | |  | **average** | **s.d.** |
| --- | --- | --- | --- | --- | --- | --- | --- | --- | --- |
| **1ZF7** |  | C-G |  | 1.574 | 1.892 | 2.099 |  | 1.86 | 0.26 |
|  |  | G-C |  | 1.91 | 1.918 | 1.837 |  | 1.89 | 0.04 |
|  |  | T-A |  | 1.782 | 1.92 |  |  | 1.85 | 0.10 |
|  |  | C-G |  | 1.892 | 1.898 | 1.875 |  | 1.89 | 0.01 |
|  |  | G-C |  | 1.918 | 1.957 | 1.901 |  | 1.93 | 0.03 |
|  |  | A-T |  | 1.966 | 1.883 |  |  | 1.92 | 0.06 |
|  |  | C-G |  | 1.976 | 1.982 | 1.923 |  | 1.96 | 0.03 |
|  |  | G-C |  | 2.16 | 2.047 | 1.905 |  | 2.04 | 0.13 |
| **1ZFB** |  | G-C |  | 1.962 | 1.958 | 1.905 |  | 1.94 | 0.03 |
|  |  | G-C |  | 1.887 | 1.887 | 1.808 |  | 1.86 | 0.05 |
|  |  | C-G |  | 2.111 | 1.821 | 1.478 |  | 1.80 | 0.32 |
|  |  | G-C |  | 2.076 | 2.035 | 1.935 |  | 2.02 | 0.07 |
|  |  | G-C |  | 1.798 | 1.927 | 2.002 |  | 1.91 | 0.10 |
|  |  | C-G |  | 2.14 | 2.059 | 1.879 |  | 2.03 | 0.13 |
|  |  | C-G |  | 2.068 | 1.987 | 1.834 |  | 1.96 | 0.12 |
|  |  | G-C |  | 1.676 | 1.749 | 1.725 |  | 1.72 | 0.04 |
|  |  | C-G |  | 2.24 | 2.081 | 2.008 |  | 2.11 | 0.12 |
|  |  | C-G |  | 2.25 | 1.904 | 1.571 |  | 1.91 | 0.34 |
| **1ZFC** |  | G-C |  | 1.8 | 2.054 | 2.268 |  | 2.04 | 0.23 |
|  |  | G-C |  | 1.882 | 1.75 | 1.821 |  | 1.82 | 0.07 |
|  |  | C-G |  | 2.26 | 2.136 | 2.179 |  | 2.19 | 0.06 |
|  |  | T-A |  | 2.214 | 1.796 |  |  | 2.01 | 0.30 |
|  |  | A-T |  | 1.954 | 2.424 |  |  | 2.19 | 0.33 |
|  |  | T-A |  | 1.977 | 1.509 |  |  | 1.74 | 0.33 |
|  |  | A-T |  | 1.584 | 1.748 |  |  | 1.67 | 0.12 |
|  |  | G-C |  | 1.813 | 1.775 | 1.672 |  | 1.75 | 0.07 |
|  |  | C-G |  | 2.201 | 2.177 | 2.117 |  | 2.17 | 0.04 |
|  |  | C-G |  | 2.326 | 1.993 | 1.543 |  | 1.95 | 0.39 |
| **1ZFH** |  | G-C |  | 1.737 | 2.235 | 2.973 |  | 2.32 | 0.62 |
|  |  | G-C |  | 1.437 | 1.991 | 2.392 |  | 1.94 | 0.48 |
|  |  | T-A |  | 2.133 | 2.09 |  |  | 2.11 | 0.03 |
|  |  | A-T |  | 1.202 | 1.283 |  |  | 1.24 | 0.06 |
|  |  | T-A |  | 1.585 | 2.211 |  |  | 1.90 | 0.44 |
|  |  | A-T |  | 1.939 | 1.825 |  |  | 1.88 | 0.08 |
|  |  | G-C |  | 2.647 | 2.03 | 1.479 |  | 2.05 | 0.58 |
|  |  | G-C |  | 1.769 | 2.131 | 2.308 |  | 2.07 | 0.27 |
| **1BWT** |  | C-G |  | 1.755 | 1.834 | 1.73 |  | 1.77 | 0.05 |
|  |  | G-C |  | 1.772 | 1.839 | 1.716 |  | 1.78 | 0.06 |
|  |  | C-G |  | 1.755 | 1.827 | 1.726 |  | 1.77 | 0.05 |
|  |  | T-A |  | 1.857 | 1.822 |  |  | 1.84 | 0.02 |
|  |  | T-A |  | 1.794 | 1.797 |  |  | 1.80 | 0.00 |
|  |  | A-T |  | 1.778 | 1.822 |  |  | 1.80 | 0.03 |
|  |  | A-T |  | 1.919 | 1.804 |  |  | 1.86 | 0.08 |
|  |  | G-C |  | 1.759 | 1.825 | 1.716 |  | 1.77 | 0.05 |
|  |  | C-G |  | 1.777 | 1.837 | 1.712 |  | 1.78 | 0.06 |
|  |  | G-C |  | 1.754 | 1.833 | 1.732 |  | 1.77 | 0.05 |

| **PDB ID** |  | **Pair** |  | **HBL (angstrom)** | | |  | **average** | **s.d.** |
| --- | --- | --- | --- | --- | --- | --- | --- | --- | --- |
| **1CS2-1** |  | G-C |  | 1.812 | 1.858 | 1.786 |  | 1.82 | 0.04 |
|  |  | A-T |  | 2.092 | 1.797 |  |  | 1.94 | 0.21 |
|  |  | T-A |  | 2.259 | 1.8 |  |  | 2.03 | 0.32 |
|  |  | G-C |  | 1.849 | 1.857 | 1.751 |  | 1.82 | 0.06 |
|  |  | A-T |  | 1.801 | 1.894 |  |  | 1.85 | 0.07 |
|  |  | C-G |  | 1.809 | 1.853 | 1.771 |  | 1.81 | 0.04 |
|  |  | G-C |  | 1.824 | 1.873 | 1.792 |  | 1.83 | 0.04 |
|  |  | A-T |  | 1.843 | 1.844 |  |  | 1.84 | 0.00 |
|  |  | A-T |  | 1.918 | 1.783 |  |  | 1.85 | 0.10 |
|  |  | A-T |  | 1.99 | 1.805 |  |  | 1.90 | 0.13 |
|  |  | T-A |  | 1.922 | 1.807 |  |  | 1.86 | 0.08 |
|  |  | C-G |  | 1.814 | 1.866 | 1.811 |  | 1.83 | 0.03 |
| **1CS2-2** |  | G-C |  | 1.945 | 1.986 | 1.914 |  | 1.95 | 0.04 |
|  |  | A-T |  | 2.147 | 1.877 |  |  | 2.01 | 0.19 |
|  |  | T-A |  | 2.605 | 1.915 |  |  | 2.26 | 0.49 |
|  |  | G-C |  | 2.014 | 1.959 | 1.875 |  | 1.95 | 0.07 |
|  |  | A-T |  | 1.931 | 1.98 |  |  | 1.96 | 0.03 |
|  |  | C-G |  | 1.925 | 1.976 | 1.915 |  | 1.94 | 0.03 |
|  |  | G-C |  | 1.942 | 1.983 | 1.909 |  | 1.94 | 0.04 |
|  |  | A-T |  | 1.984 | 1.925 |  |  | 1.95 | 0.04 |
|  |  | A-T |  | 2.087 | 1.853 |  |  | 1.97 | 0.17 |
|  |  | A-T |  | 2.115 | 1.875 |  |  | 2.00 | 0.17 |
|  |  | T-A |  | 2.038 | 1.884 |  |  | 1.96 | 0.11 |
|  |  | C-G |  | 1.934 | 1.983 | 1.911 |  | 1.94 | 0.04 |
| **1CS2-3** |  | G-C |  | 1.816 | 1.829 | 1.765 |  | 1.80 | 0.03 |
|  |  | A-T |  | 2.035 | 1.743 |  |  | 1.89 | 0.21 |
|  |  | T-A |  | 1.934 | 1.705 |  |  | 1.82 | 0.16 |
|  |  | G-C |  | 1.841 | 1.837 | 1.715 |  | 1.80 | 0.07 |
|  |  | A-T |  | 2.046 | 1.751 |  |  | 1.90 | 0.21 |
|  |  | C-G |  | 1.731 | 1.846 | 1.78 |  | 1.79 | 0.06 |
|  |  | G-C |  | 1.8 | 1.83 | 1.733 |  | 1.79 | 0.05 |
|  |  | A-T |  | 1.839 | 1.724 |  |  | 1.78 | 0.08 |
|  |  | A-T |  | 1.798 | 1.812 |  |  | 1.81 | 0.01 |
|  |  | A-T |  | 1.882 | 1.85 |  |  | 1.87 | 0.02 |
|  |  | T-A |  | 1.856 | 1.813 |  |  | 1.83 | 0.03 |
|  |  | C-G |  | 1.869 | 1.798 | 1.739 |  | 1.80 | 0.07 |
| **1D68** |  | C-G |  | 1.852 | 1.836 | 1.898 |  | 1.86 | 0.03 |
|  |  | G-C |  | 1.839 | 1.878 | 1.855 |  | 1.86 | 0.02 |
|  |  | C-G |  | 1.85 | 1.88 | 1.814 |  | 1.85 | 0.03 |
|  |  | A-T |  | 1.964 | 1.889 |  |  | 1.93 | 0.05 |
|  |  | T-A |  | 1.978 | 1.873 |  |  | 1.93 | 0.07 |
|  |  | A-T |  | 1.977 | 1.783 |  |  | 1.88 | 0.14 |
|  |  | T-A |  | 1.963 | 1.89 |  |  | 1.93 | 0.05 |
|  |  | G-C |  | 1.847 | 1.88 | 1.816 |  | 1.85 | 0.03 |
|  |  | C-G |  | 1.84 | 1.878 | 1.854 |  | 1.86 | 0.02 |
|  |  | G-C |  | 1.852 | 1.836 | 1.898 |  | 1.86 | 0.03 |

T-able S4. Experimentally-determined GVA data of duplex from Cartesian coordinates of RCSB PDB structures

| **PDB ID** |  | **Pair** |  | **GVA (degree)** |  | **PDB ID** |  | **Pair** |  | **GVA (degree)** |
| --- | --- | --- | --- | --- | --- | --- | --- | --- | --- | --- |
| **1ZF7** |  | C-G |  | 79.15 |  | **1BWT** |  | C-G |  | 71.34 |
|  |  | C-G |  | 78.88 |  |  |  | G-C |  | 69.04 |
|  |  | G-C |  | 73.36 |  |  |  | C-G |  | 70.37 |
|  |  | C-G |  | 80.06 |  |  |  | T-A |  | 66 |
|  |  | C-G |  | 72.92 |  |  |  | T-A |  | 69.08 |
|  |  | G-C |  | 73.66 |  |  |  | A-T |  | 72.25 |
|  |  | G-C |  | 70.55 |  |  |  | A-T |  | 62.27 |
|  |  | C-G |  | 73.42 |  |  |  | G-C |  | 68.92 |
|  |  | G-C |  | 68.33 |  |  |  | C-G |  | 67.87 |
|  |  | G-C |  | 62.95 |  |  |  | G-C |  | 70.89 |
| **1ZFB** |  | C-G |  | 73.29 |  | **1CS2-1** |  | G-C |  | 70.77 |
|  |  | C-G |  | 69.59 |  |  |  | A-T |  | 62.5 |
|  |  | G-C |  | 67.31 |  |  |  | T-A |  | 61.17 |
|  |  | C-G |  | 72 |  |  |  | G-C |  | 70.43 |
|  |  | C-G |  | 74.86 |  |  |  | A-T |  | 72.79 |
|  |  | G-C |  | 70.84 |  |  |  | C-G |  | 70.6 |
|  |  | G-C |  | 74.8 |  |  |  | G-C |  | 71.02 |
|  |  | C-G |  | 73.37 |  |  |  | A-T |  | 70.94 |
|  |  | G-C |  | 70.54 |  |  |  | A-T |  | 67.23 |
|  |  | G-C |  | 65.56 |  |  |  | A-T |  | 66.3 |
| **1ZFC** |  | C-G |  | 68.57 |  |  |  | T-A |  | 67.99 |
|  |  | C-G |  | 72.99 |  |  |  | C-G |  | 71.46 |
|  |  | G-C |  | 82.38 |  | **1CS2-2** |  | G-C |  | 70.18 |
|  |  | A-T |  | 97.08 |  |  |  | A-T |  | 63.54 |
|  |  | T-A |  | 65.65 |  |  |  | T-A |  | 59.68 |
|  |  | A-T |  | 61.5 |  |  |  | G-C |  | 70.66 |
|  |  | T-A |  | 73.63 |  |  |  | A-T |  | 71.99 |
|  |  | C-G |  | 69.89 |  |  |  | C-G |  | 70.69 |
|  |  | G-C |  | 70.31 |  |  |  | G-C |  | 71.09 |
|  |  | G-C |  | 76.73 |  |  |  | A-T |  | 69.99 |
| **1ZFH** |  | C-G |  | 59.85 |  |  |  | A-T |  | 65.42 |
|  |  | C-G |  | 70.14 |  |  |  | A-T |  | 65.15 |
|  |  | T-A |  | 92.67 |  |  |  | T-A |  | 67.24 |
|  |  | A-T |  | 76.42 |  |  |  | C-G |  | 70.84 |
|  |  | A-T |  | 79.33 |  | **1CS2-3** |  | G-C |  | 72.55 |
|  |  | T-A |  | 38.27 |  |  |  | A-T |  | 65.97 |
|  |  | T-A |  | 54.29 |  |  |  | T-A |  | 67.27 |
|  |  | A-T |  | 88.56 |  |  |  | G-C |  | 70.84 |
|  |  | G-C |  | 87.86 |  |  |  | A-T |  | 64.56 |
|  |  | G-C |  | 65.62 |  |  |  | C-G |  | 72.5 |
| **1ZFM** |  | C-G |  | 93.79 |  |  |  | G-C |  | 71.94 |
|  |  | C-G |  | 81.87 |  |  |  | A-T |  | 69.77 |
|  |  | G-C |  | 85.28 |  |  |  | A-T |  | 72.22 |
|  |  | C-G |  | 79.4 |  |  |  | A-T |  | 70.71 |
|  |  | T-A |  | 80.28 |  |  |  | T-A |  | 71.88 |
|  |  | A-T |  | 79.8 |  |  |  | C-G |  | 71.88 |
|  |  | G-C |  | 90.96 |  | **1D68** |  | C-G |  | 73.48 |
|  |  | C-G |  | 61.71 |  |  |  | G-C |  | 70.92 |
|  |  | G-C |  | 79.97 |  |  |  | C-G |  | 70.51 |
|  |  | G-C |  | 85.29 |  |  |  | A-T |  | 74.08 |
|  |  |  |  |  |  |  |  | T-A |  | 72.23 |
|  |  |  |  |  |  |  |  | A-T |  | 72.22 |
|  |  |  |  |  |  |  |  | T-A |  | 74.08 |
|  |  |  |  |  |  |  |  | G-C |  | 70.59 |
|  |  |  |  |  |  |  |  | C-G |  | 70.93 |
|  |  |  |  |  |  |  |  | G-C |  | 73.51 |

Table S5. Experimentally-determined TA data of duplex from Cartesian coordinates of RCSB PDB structures

| **PDB ID** |  | **Duplex** |  | **Twist (degree)** |  | **PDB ID** |  | **Duplex** |  | **Twist (degree)** |
| --- | --- | --- | --- | --- | --- | --- | --- | --- | --- | --- |
| **1ZF7** |  | G-C/G-C |  | 42.1 |  | **1BWT** |  | C-G/G-C |  | 35.9 |
|  |  | G-C/C-G |  | 30.8 |  |  |  | G-C/C-G |  | 33.1 |
|  |  | G-C/T-A |  | 33.1 |  |  |  | C-G/T-A |  | 35.4 |
|  |  | G-C/A-T |  | 42.6 |  |  |  | T-A/T-A |  | 39.1 |
|  |  | G-C/C-G |  | 32.7 |  |  |  | T-A/A-T |  | 31.6 |
|  |  | G-C/A-T |  | 28.9 |  |  |  | A-T/A-T |  | 39.1 |
|  |  | G-C/T-A |  | 45.4 |  |  |  | A-T/G-C |  | 34.6 |
|  |  | G-C/C-G |  | 26.1 |  |  |  | G-C/C-G |  | 33.1 |
| **1ZFB** |  | G-C/G-C |  | 40.1 |  |  |  | C-G/G-C |  | 35.1 |
|  |  | G-C/C-G |  | 36.7 |  | **1CS2-1** |  | G-C/A-T |  | 35.4 |
|  |  | G-C/C-G |  | 36.1 |  |  |  | A-T/T-A |  | 37.4 |
|  |  | G-C/G-C |  | 33.9 |  |  |  | T-A/G-C |  | 33.4 |
|  |  | G-C/C-G |  | 36.0 |  |  |  | G-C/A-T |  | 35.2 |
|  |  | G-C/G-C |  | 36.3 |  |  |  | A-T/C-G |  | 33.0 |
|  |  | G-C/C-G |  | 37.3 |  |  |  | C-G/G-C |  | 35.9 |
|  |  | G-C/C-G |  | 29.5 |  |  |  | G-C/A-T |  | 30.7 |
|  |  | G-C/G-C |  | 39.7 |  |  |  | A-T/A-T |  | 37.6 |
| **1ZFC** |  | G-C/G-C |  | 41.8 |  |  |  | A-T/A-T |  | 31.2 |
|  |  | G-C/C-G |  | 32.9 |  |  |  | A-T/T-A |  | 34.8 |
|  |  | G-C/A-T |  | 37.1 |  |  |  | T-A/C-G |  | 31.2 |
|  |  | A-T/T-A |  | 42.8 |  | **1CS2-2** |  | G-C/A-T |  | 40.4 |
|  |  | A-T/T-A |  | 29.7 |  |  |  | A-T/T-A |  | 38.7 |
|  |  | A-T/T-A |  | 36.9 |  |  |  | T-A/G-C |  | 34.1 |
|  |  | G-C/A-T |  | 35.1 |  |  |  | G-C/A-T |  | 35.9 |
|  |  | G-C/C-G |  | 40.6 |  |  |  | A-T/C-G |  | 33.0 |
|  |  | G-C/G-C |  | 34.9 |  |  |  | C-G/G-C |  | 35.9 |
| **1ZFM** |  | G-C/G-C |  | 40.0 |  |  |  | G-C/A-T |  | 31.8 |
|  |  | G-C/C-G |  | 36.1 |  |  |  | A-T/A-T |  | 36.8 |
|  |  | C-G/G-C |  | 36.9 |  |  |  | A-T/A-T |  | 32.8 |
|  |  | G-C/A-T |  | 26.1 |  |  |  | A-T/T-A |  | 34.7 |
|  |  | A-T/T-A |  | 47.7 |  |  |  | T-A/C-G |  | 32.9 |
|  |  | T-A/C-G |  | 30.0 |  | **1CS2-3** |  | G-C/A-T |  | 37.8 |
|  |  | C-G/G-C |  | 41.2 |  |  |  | A-T/T-A |  | 35.3 |
|  |  | G-C/C-G |  | 38.8 |  |  |  | T-A/G-C |  | 31.2 |
|  |  | C-G/C-G |  | 37.7 |  |  |  | G-C/A-T |  | 35.2 |
| **1ZFH** |  | G-C/G-C |  | 29.5 |  |  |  | A-T/C-G |  | 34.7 |
|  |  | G-C/T-A |  | 42.5 |  |  |  | C-G/G-C |  | 35.9 |
|  |  | A-T/T-A |  | 42.1 |  |  |  | G-C/A-T |  | 34.9 |
|  |  | A-T/A-T |  | 28.5 |  |  |  | A-T/A-T |  | 34.2 |
|  |  | A-T/T-A |  | 33.5 |  |  |  | A-T/A-T |  | 33.1 |
|  |  | A-T/A-T |  | 51.4 |  |  |  | A-T/T-A |  | 30.4 |
|  |  | A-T/T-A |  | 34.9 |  |  |  | T-A/C-G |  | 39.7 |
|  |  | G-C/A-T |  | 40.6 |  | **1D68** |  | C-G/G-C |  | 30.9 |
|  |  | G-C/G-C |  | 41.6 |  |  |  | G-C/C-G |  | 39.9 |
|  |  |  |  |  |  |  |  | C-G/A-T |  | 36.6 |
|  |  |  |  |  |  |  |  | A-T/T-A |  | 46.8 |
|  |  |  |  |  |  |  |  | T-A/A-T |  | 36.1 |
|  |  |  |  |  |  |  |  | A-T/T-A |  | 46.8 |
|  |  |  |  |  |  |  |  | T-A/G-C |  | 36.6 |
|  |  |  |  |  |  |  |  | G-C/C-G |  | 39.9 |
|  |  |  |  |  |  |  |  | C-G/G-C |  | 30.9 |

Table S6. Experimentally-determined TA data of triad from Cartesian coordinates of RCSB PDB structures.

| **PDB ID** |  | **Triad** |  | **HBL (Watson-Crick)** | | | **Average** | **s.d.** |  | **HBL (Hoogsteen)** | | **Average** | **s.d.** |
| --- | --- | --- | --- | --- | --- | --- | --- | --- | --- | --- | --- | --- | --- |
| **149D-1** |  | **C^+^•G-C** |  | 1.769 | 1.960 | 1.791 | 1.83 | 0.10 |  | 1.763 | 1.857 | 1.81 | 0.07 |
|  |  | **T•A-T** |  | 1.842 | 1.829 |  | 1.84 | 0.01 |  | 1.769 | 1.848 | 1.81 | 0.06 |
|  |  | **T•A-T** |  | 1.888 | 1.716 |  | 1.80 | 0.12 |  | 1.835 | 1.882 | 1.86 | 0.03 |
|  |  | **T•A-T** |  | 1.932 | 1.758 |  | 1.85 | 0.12 |  | 1.783 | 2.022 | 1.90 | 0.17 |
|  |  | **C^+^•G-C** |  | 1.831 | 1.864 | 1.717 | 1.80 | 0.08 |  | 1.761 | 1.838 | 1.80 | 0.05 |
|  |  | **C^+^•G-C** |  | 1.798 | 1.915 | 1.731 | 1.81 | 0.09 |  | 1.788 | 1.836 | 1.81 | 0.03 |
| **149D-2** |  | **C^+^•G-C** |  | 1.790 | 1.904 | 1.774 | 1.83 | 0.07 |  | 1.775 | 1.900 | 1.84 | 0.09 |
|  |  | **T•A-T** |  | 1.829 | 1.822 |  | 1.83 | 0.00 |  | 1.784 | 1.846 | 1.82 | 0.04 |
|  |  | **T•A-T** |  | 1.929 | 1.825 |  | 1.88 | 0.07 |  | 1.816 | 1.816 | 1.82 | 0.00 |
|  |  | **T•A-T** |  | 1.920 | 1.841 |  | 1.88 | 0.06 |  | 1.743 | 2.007 | 1.88 | 0.19 |
|  |  | **C^+^•G-C** |  | 1.852 | 1.849 | 1.717 | 1.83 | 0.08 |  | 1.831 | 1.901 | 1.87 | 0.05 |
|  |  | **C^+^•G-C** |  | 1.862 | 1.967 | 1.722 | 1.86 | 0.12 |  | 1.893 | 1.842 | 1.87 | 0.04 |
| **149D-3** |  | **C^+^•G-C** |  | 1.783 | 1.969 | 1.853 | 1.85 | 0.09 |  | 1.754 | 1.898 | 1.83 | 0.10 |
|  |  | **T•A-T** |  | 1.790 | 1.922 |  | 1.86 | 0.09 |  | 1.771 | 1.838 | 1.80 | 0.05 |
|  |  | **T•A-T** |  | 1.890 | 1.851 |  | 1.87 | 0.03 |  | 1.803 | 1.847 | 1.83 | 0.03 |
|  |  | **T•A-T** |  | 1.860 | 1.720 |  | 1.79 | 0.10 |  | 1.718 | 2.024 | 1.87 | 0.22 |
|  |  | **C^+^•G-C** |  | 2.011 | 1.931 | 1.748 | 1.88 | 0.13 |  | 1.798 | 1.903 | 1.85 | 0.07 |
|  |  | **C^+^•G-C** |  | 1.791 | 1.906 | 1.707 | 1.84 | 0.10 |  | 1.890 | 1.900 | 1.90 | 0.01 |
| **149D-4** |  | **C^+^•G-C** |  | 1.832 | 1.854 | 1.744 | 1.82 | 0.06 |  | 1.803 | 1.860 | 1.83 | 0.04 |
|  |  | **T•A-T** |  | 1.813 | 1.795 |  | 1.80 | 0.01 |  | 1.872 | 1.911 | 1.89 | 0.03 |
|  |  | **T•A-T** |  | 1.848 | 1.796 |  | 1.82 | 0.04 |  | 1.744 | 1.891 | 1.82 | 0.10 |
|  |  | **T•A-T** |  | 1.814 | 1.930 |  | 1.87 | 0.08 |  | 1.748 | 2.013 | 1.88 | 0.19 |
|  |  | **C^+^•G-C** |  | 1.993 | 1.969 | 1.754 | 1.89 | 0.13 |  | 1.875 | 1.869 | 1.87 | 0.00 |
|  |  | **C^+^•G-C** |  | 1.792 | 1.970 | 1.728 | 1.84 | 0.13 |  | 1.886 | 1.840 | 1.86 | 0.03 |
| **149D-5** |  | **C^+^•G-C** |  | 1.782 | 1.914 | 1.743 | 1.82 | 0.09 |  | 1.802 | 1.879 | 1.84 | 0.05 |
|  |  | **T•A-T** |  | 1.782 | 1.820 |  | 1.80 | 0.03 |  | 1.928 | 1.786 | 1.86 | 0.10 |
|  |  | **T•A-T** |  | 1.856 | 1.785 |  | 1.82 | 0.05 |  | 1.836 | 1.904 | 1.87 | 0.05 |
|  |  | **T•A-T** |  | 1.858 | 1.789 |  | 1.82 | 0.05 |  | 1.767 | 1.978 | 1.87 | 0.15 |
|  |  | **C^+^•G-C** |  | 2.014 | 1.903 | 1.719 | 1.86 | 0.15 |  | 1.807 | 1.857 | 1.83 | 0.04 |
|  |  | **C^+^•G-C** |  | 1.797 | 1.923 | 1.742 | 1.88 | 0.09 |  | 2.004 | 1.946 | 1.98 | 0.04 |
| **149D-6** |  | **C^+^•G-C** |  | 1.804 | 1.951 | 1.743 | 1.85 | 0.11 |  | 1.782 | 1.972 | 1.88 | 0.13 |
|  |  | **T•A-T** |  | 1.796 | 1.793 |  | 1.79 | 0.00 |  | 1.759 | 1.790 | 1.77 | 0.02 |
|  |  | **T•A-T** |  | 1.841 | 1.769 |  | 1.81 | 0.05 |  | 1.746 | 1.837 | 1.79 | 0.06 |
|  |  | **T•A-T** |  | 1.848 | 1.865 |  | 1.86 | 0.01 |  | 1.742 | 1.995 | 1.87 | 0.18 |
|  |  | **C^+^•G-C** |  | 1.818 | 1.871 | 1.727 | 1.83 | 0.07 |  | 1.816 | 1.931 | 1.87 | 0.08 |
|  |  | **C^+^•G-C** |  | 1.798 | 1.952 | 1.735 | 1.84 | 0.11 |  | 1.837 | 1.880 | 1.86 | 0.03 |
| **149D-7** |  | **C^+^•G-C** |  | 1.816 | 1.910 | 1.732 | 1.83 | 0.09 |  | 1.806 | 1.895 | 1.85 | 0.06 |
|  |  | **T•A-T** |  | 1.818 | 1.861 |  | 1.84 | 0.03 |  | 1.811 | 1.826 | 1.82 | 0.01 |
|  |  | **T•A-T** |  | 1.850 | 1.764 |  | 1.81 | 0.06 |  | 1.740 | 1.835 | 1.79 | 0.07 |
|  |  | **T•A-T** |  | 1.833 | 1.867 |  | 1.85 | 0.02 |  | 1.739 | 1.970 | 1.85 | 0.16 |
|  |  | **C^+^•G-C** |  | 2.016 | 1.943 | 1.754 | 1.88 | 0.14 |  | 1.809 | 1.884 | 1.85 | 0.05 |
|  |  | **C^+^•G-C** |  | 1.787 | 1.916 | 1.760 | 1.85 | 0.08 |  | 1.928 | 1.877 | 1.90 | 0.04 |
| **149D-8** |  | **C^+^•G-C** |  | 1.812 | 1.917 | 1.739 | 1.82 | 0.09 |  | 1.772 | 1.868 | 1.82 | 0.07 |
|  |  | **T•A-T** |  | 1.826 | 1.843 |  | 1.83 | 0.01 |  | 1.748 | 1.824 | 1.79 | 0.05 |
|  |  | **T•A-T** |  | 1.941 | 1.711 |  | 1.83 | 0.16 |  | 1.785 | 1.820 | 1.80 | 0.02 |
|  |  | **T•A-T** |  | 1.897 | 1.909 |  | 1.90 | 0.01 |  | 1.781 | 1.991 | 1.89 | 0.15 |
|  |  | **C^+^•G-C** |  | 1.804 | 1.875 | 1.730 | 1.83 | 0.07 |  | 1.775 | 1.988 | 1.88 | 0.15 |
|  |  | **C^+^•G-C** |  | 1.797 | 1.949 | 1.727 | 1.83 | 0.11 |  | 1.778 | 1.906 | 1.84 | 0.09 |
| **149D-9** |  | **C^+^•G-C** |  | 1.823 | 1.933 | 1.732 | 1.82 | 0.10 |  | 1.808 | 1.820 | 1.81 | 0.01 |
|  |  | **T•A-T** |  | 1.888 | 1.729 |  | 1.81 | 0.11 |  | 1.803 | 1.806 | 1.80 | 0.00 |
|  |  | **T•A-T** |  | 1.817 | 1.735 |  | 1.78 | 0.06 |  | 1.766 | 1.784 | 1.78 | 0.01 |
|  |  | **T•A-T** |  | 1.817 | 1.885 |  | 1.85 | 0.05 |  | 1.178 | 1.998 | 1.59 | 0.58 |
|  |  | **C^+^•G-C** |  | 1.813 | 1.857 | 1.759 | 1.83 | 0.05 |  | 1.750 | 1.968 | 1.86 | 0.15 |
|  |  | **C^+^•G-C** |  | 1.808 | 1.904 | 1.713 | 1.85 | 0.10 |  | 1.802 | 2.010 | 1.91 | 0.15 |

| **PDB ID** |  | **Triad** |  | **HBL (Watson-Crick)** | | | **Average** | **s.d.** |  | **HBL (Hoogsteen)** | | **Average** | **s.d.** |
| --- | --- | --- | --- | --- | --- | --- | --- | --- | --- | --- | --- | --- | --- |
| **149D-10** |  | **C^+^•G-C** |  | 1.791 | 1.948 | 1.731 | 1.82 | 0.11 |  | 1.808 | 1.817 | 1.81 | 0.01 |
|  |  | **T•A-T** |  | 1.785 | 1.843 |  | 1.81 | 0.04 |  | 1.893 | 1.800 | 1.85 | 0.07 |
|  |  | **T•A-T** |  | 1.931 | 1.758 |  | 1.84 | 0.12 |  | 1.764 | 1.878 | 1.82 | 0.08 |
|  |  | **T•A-T** |  | 1.824 | 1.895 |  | 1.86 | 0.05 |  | 1.759 | 2.034 | 1.90 | 0.19 |
|  |  | **C^+^•G-C** |  | 1.804 | 1.901 | 1.767 | 1.83 | 0.07 |  | 1.743 | 1.928 | 1.84 | 0.13 |
|  |  | **C^+^•G-C** |  | 1.782 | 1.909 | 1.726 | 1.82 | 0.09 |  | 1.791 | 1.879 | 1.84 | 0.06 |
| **149D-11** |  | **C^+^•G-C** |  | 1.818 | 1.917 | 1.712 | 1.81 | 0.10 |  | 1.807 | 1.815 | 1.81 | 0.01 |
|  |  | **T•A-T** |  | 1.845 | 1.731 |  | 1.79 | 0.08 |  | 1.807 | 1.802 | 1.80 | 0.00 |
|  |  | **T•A-T** |  | 1.850 | 1.724 |  | 1.79 | 0.09 |  | 1.754 | 1.841 | 1.80 | 0.06 |
|  |  | **T•A-T** |  | 1.806 | 1.905 |  | 1.86 | 0.07 |  | 1.833 | 2.032 | 1.93 | 0.14 |
|  |  | **C^+^•G-C** |  | 1.772 | 1.886 | 1.750 | 1.84 | 0.07 |  | 1.772 | 2.027 | 1.90 | 0.18 |
|  |  | **C^+^•G-C** |  | 1.793 | 1.977 | 1.747 | 1.84 | 0.12 |  | 1.794 | 1.867 | 1.83 | 0.05 |
| **149D-12** |  | **C^+^•G-C** |  | 1.842 | 1.952 | 1.736 | 1.83 | 0.11 |  | 1.793 | 1.807 | 1.80 | 0.01 |
|  |  | **T•A-T** |  | 1.875 | 1.778 |  | 1.83 | 0.07 |  | 1.815 | 1.800 | 1.81 | 0.01 |
|  |  | **T•A-T** |  | 1.837 | 1.770 |  | 1.80 | 0.05 |  | 1.762 | 1.847 | 1.80 | 0.06 |
|  |  | **T•A-T** |  | 1.775 | 1.871 |  | 1.82 | 0.07 |  | 1.781 | 2.056 | 1.92 | 0.19 |
|  |  | **C^+^•G-C** |  | 1.774 | 1.917 | 1.774 | 1.85 | 0.08 |  | 1.740 | 2.022 | 1.88 | 0.20 |
|  |  | **C^+^•G-C** |  | 1.792 | 1.961 | 1.733 | 1.83 | 0.12 |  | 1.776 | 1.874 | 1.83 | 0.07 |
| **149D-13** |  | **C^+^•G-C** |  | 1.889 | 1.951 | 1.768 | 1.84 | 0.09 |  | 1.791 | 1.813 | 1.80 | 0.02 |
|  |  | **T•A-T** |  | 1.891 | 1.781 |  | 1.84 | 0.08 |  | 1.796 | 1.829 | 1.81 | 0.02 |
|  |  | **T•A-T** |  | 1.847 | 1.729 |  | 1.79 | 0.08 |  | 1.773 | 1.834 | 1.80 | 0.04 |
|  |  | **T•A-T** |  | 1.856 | 1.886 |  | 1.87 | 0.02 |  | 1.785 | 2.012 | 1.90 | 0.16 |
|  |  | **C^+^•G-C** |  | 1.792 | 1.876 | 1.751 | 1.82 | 0.06 |  | 1.762 | 1.895 | 1.83 | 0.09 |
|  |  | **C^+^•G-C** |  | 1.782 | 1.933 | 1.725 | 1.85 | 0.11 |  | 1.854 | 1.949 | 1.90 | 0.07 |
| **149D-14** |  | **C^+^•G-C** |  | 1.816 | 1.967 | 1.731 | 1.84 | 0.12 |  | 1.824 | 1.842 | 1.83 | 0.01 |
|  |  | **T•A-T** |  | 1.800 | 1.838 |  | 1.82 | 0.03 |  | 1.843 | 1.827 | 1.84 | 0.01 |
|  |  | **T•A-T** |  | 1.887 | 1.754 |  | 1.82 | 0.09 |  | 1.745 | 1.845 | 1.80 | 0.07 |
|  |  | **T•A-T** |  | 1.820 | 1.865 |  | 1.84 | 0.03 |  | 1.823 | 2.001 | 1.91 | 0.13 |
|  |  | **C^+^•G-C** |  | 1.763 | 1.861 | 1.802 | 1.82 | 0.05 |  | 1.762 | 1.923 | 1.84 | 0.11 |
|  |  | **C^+^•G-C** |  | 1.802 | 1.940 | 1.690 | 1.85 | 0.13 |  | 1.800 | 2.001 | 1.90 | 0.14 |
| **1BWG-1** |  | **C_+_•G-C** |  | 2.069 | 2.010 | 2.026 | 2.059 | 0.031 |  | 1.974 | 2.214 | 2.094 | 0.170 |
|  |  | **T•A-T** |  | 2.099 | 1.877 |  | 1.988 | 0.157 |  | 1.992 | 1.941 | 1.967 | 0.036 |
|  |  | **C^+^•G-C** |  | 1.972 | 2.040 | 2.081 | 1.999 | 0.055 |  | 2.030 | 1.870 | 1.950 | 0.113 |
|  |  | **T•A-T** |  | 2.102 | 1.925 |  | 2.014 | 0.125 |  | 1.995 | 2.059 | 2.027 | 0.045 |
|  |  | **C^+^•G-C** |  | 2.061 | 2.016 | 2.010 | 2.042 | 0.028 |  | 2.030 | 2.095 | 2.063 | 0.046 |
|  |  | **T•A-T** |  | 2.006 | 1.942 |  | 1.999 | 0.045 |  | 1.905 | 2.144 | 2.025 | 0.169 |
| **1BWG-2** |  | **C_+_•G-C** |  | 2.052 | 2.008 | 2.059 | 2.054 | 0.028 |  | 1.939 | 2.212 | 2.076 | 0.193 |
|  |  | **T•A-T** |  | 2.051 | 1.882 |  | 1.967 | 0.120 |  | 1.944 | 1.990 | 1.967 | 0.033 |
|  |  | **C^+^•G-C** |  | 1.974 | 2.043 | 2.116 | 1.997 | 0.071 |  | 1.986 | 1.865 | 1.926 | 0.086 |
|  |  | **T•A-T** |  | 2.026 | 1.956 |  | 1.991 | 0.049 |  | 2.028 | 2.063 | 2.046 | 0.025 |
|  |  | **C^+^•G-C** |  | 2.122 | 2.041 | 2.028 | 2.052 | 0.051 |  | 1.994 | 2.076 | 2.035 | 0.058 |
|  |  | **T•A-T** |  | 1.997 | 1.946 |  | 1.997 | 0.036 |  | 1.903 | 2.142 | 2.023 | 0.169 |
| **1BWG-3** |  | **C_+_•G-C** |  | 2.059 | 2.014 | 2.043 | 2.056 | 0.023 |  | 1.951 | 2.215 | 2.083 | 0.187 |
|  |  | **T•A-T** |  | 2.097 | 1.873 |  | 1.985 | 0.158 |  | 1.932 | 1.990 | 1.961 | 0.041 |
|  |  | **C^+^•G-C** |  | 1.969 | 2.055 | 2.107 | 1.996 | 0.070 |  | 1.983 | 1.868 | 1.926 | 0.081 |
|  |  | **T•A-T** |  | 2.040 | 1.947 |  | 1.994 | 0.066 |  | 2.002 | 2.068 | 2.035 | 0.047 |
|  |  | **C^+^•G-C** |  | 2.125 | 2.031 | 2.013 | 2.045 | 0.060 |  | 1.979 | 2.078 | 2.029 | 0.070 |
|  |  | **T•A-T** |  | 1.987 | 1.979 |  | 2.005 | 0.006 |  | 1.909 | 2.143 | 2.026 | 0.165 |
| **1BWG-4** |  | **C_+_•G-C** |  | 2.043 | 2.000 | 2.052 | 2.049 | 0.028 |  | 1.935 | 2.215 | 2.075 | 0.198 |
|  |  | **T•A-T** |  | 2.047 | 1.881 |  | 1.964 | 0.117 |  | 1.942 | 1.991 | 1.967 | 0.035 |
|  |  | **C^+^•G-C** |  | 1.982 | 2.025 | 2.109 | 1.993 | 0.065 |  | 1.983 | 1.868 | 1.926 | 0.081 |
|  |  | **T•A-T** |  | 2.010 | 1.951 |  | 1.981 | 0.042 |  | 2.026 | 2.060 | 2.043 | 0.024 |
|  |  | **C^+^•G-C** |  | 2.104 | 2.043 | 2.007 | 2.036 | 0.049 |  | 1.959 | 2.065 | 2.012 | 0.075 |
|  |  | **T•A-T** |  | 2.034 | 1.940 |  | 2.002 | 0.066 |  | 1.896 | 2.138 | 2.017 | 0.171 |

| **PDB ID** |  | **Triad** |  | **HBL (Watson-Crick)** | | | **Average** | **s.d.** |  | **HBL (Hoogsteen)** | | **Average** | **s.d.** |
| --- | --- | --- | --- | --- | --- | --- | --- | --- | --- | --- | --- | --- | --- |
| **1BWG-5** |  | **C_+_•G-C** |  | 2.069 | 2.008 | 2.029 | 2.059 | 0.031 |  | 1.969 | 2.218 | 2.094 | 0.176 |
|  |  | **T•A-T** |  | 2.102 | 1.875 |  | 1.989 | 0.161 |  | 1.982 | 1.941 | 1.962 | 0.029 |
|  |  | **C^+^•G-C** |  | 1.955 | 2.039 | 2.092 | 1.992 | 0.069 |  | 2.002 | 1.874 | 1.938 | 0.091 |
|  |  | **T•A-T** |  | 2.030 | 1.943 |  | 1.987 | 0.062 |  | 1.988 | 2.054 | 2.021 | 0.047 |
|  |  | **C^+^•G-C** |  | 2.128 | 2.024 | 2.004 | 2.044 | 0.067 |  | 1.979 | 2.086 | 2.033 | 0.076 |
|  |  | **T•A-T** |  | 1.989 | 1.979 |  | 2.006 | 0.007 |  | 1.912 | 2.144 | 2.028 | 0.164 |
| **1BWG-6** |  | **C_+_•G-C** |  | 2.079 | 2.023 | 2.062 | 2.064 | 0.029 |  | 1.953 | 2.204 | 2.079 | 0.177 |
|  |  | **T•A-T** |  | 2.068 | 1.867 |  | 1.968 | 0.142 |  | 1.955 | 1.986 | 1.971 | 0.022 |
|  |  | **C^+^•G-C** |  | 1.943 | 2.010 | 2.110 | 1.988 | 0.084 |  | 1.988 | 1.887 | 1.938 | 0.071 |
|  |  | **T•A-T** |  | 1.997 | 1.952 |  | 1.975 | 0.032 |  | 1.936 | 2.061 | 1.999 | 0.088 |
|  |  | **C^+^•G-C** |  | 2.116 | 2.001 | 2.003 | 2.045 | 0.066 |  | 2.003 | 2.103 | 2.053 | 0.071 |
|  |  | **T•A-T** |  | 1.988 | 2.000 |  | 2.012 | 0.008 |  | 1.918 | 2.141 | 2.030 | 0.158 |
| **1BWG-7** |  | **C_+_•G-C** |  | 2.048 | 2.005 | 1.976 | 2.012 | 0.036 |  | 1.856 | 2.173 | 2.015 | 0.224 |
|  |  | **T•A-T** |  | 2.065 | 1.892 |  | 1.979 | 0.122 |  | 1.988 | 1.929 | 1.959 | 0.042 |
|  |  | **C^+^•G-C** |  | 1.978 | 1.983 | 2.064 | 1.991 | 0.048 |  | 2.046 | 1.886 | 1.966 | 0.113 |
|  |  | **T•A-T** |  | 2.157 | 1.918 |  | 2.038 | 0.169 |  | 1.971 | 2.036 | 2.004 | 0.046 |
|  |  | **C^+^•G-C** |  | 2.009 | 2.003 | 1.991 | 1.990 | 0.009 |  | 1.867 | 2.081 | 1.974 | 0.151 |
|  |  | **T•A-T** |  | 2.072 | 1.941 |  | 2.014 | 0.093 |  | 1.904 | 2.139 | 2.022 | 0.166 |
| **1BWG-8** |  | **C_+_•G-C** |  | 2.096 | 2.016 | 2.038 | 2.065 | 0.041 |  | 1.963 | 2.210 | 2.087 | 0.175 |
|  |  | **T•A-T** |  | 2.117 | 1.857 |  | 1.987 | 0.184 |  | 1.960 | 1.972 | 1.966 | 0.008 |
|  |  | **C^+^•G-C** |  | 1.966 | 2.050 | 2.085 | 1.995 | 0.061 |  | 2.020 | 1.856 | 1.938 | 0.116 |
|  |  | **T•A-T** |  | 2.106 | 1.905 |  | 2.006 | 0.142 |  | 1.941 | 2.064 | 2.003 | 0.087 |
|  |  | **C^+^•G-C** |  | 2.023 | 2.003 | 2.033 | 2.033 | 0.015 |  | 2.000 | 2.104 | 2.052 | 0.074 |
|  |  | **T•A-T** |  | 1.976 | 1.953 |  | 1.993 | 0.016 |  | 1.897 | 2.146 | 2.022 | 0.176 |
| **1BWG-9** |  | **C_+_•G-C** |  | 2.074 | 2.008 | 2.026 | 2.058 | 0.034 |  | 1.974 | 2.210 | 2.092 | 0.167 |
|  |  | **T•A-T** |  | 2.014 | 1.878 |  | 1.946 | 0.096 |  | 1.994 | 1.938 | 1.966 | 0.040 |
|  |  | **C^+^•G-C** |  | 1.871 | 2.026 | 2.076 | 1.972 | 0.107 |  | 2.023 | 1.862 | 1.943 | 0.114 |
|  |  | **T•A-T** |  | 2.056 | 1.952 |  | 2.004 | 0.074 |  | 2.012 | 2.072 | 2.042 | 0.042 |
|  |  | **C^+^•G-C** |  | 2.119 | 2.057 | 2.030 | 2.052 | 0.046 |  | 1.989 | 2.065 | 2.027 | 0.054 |
|  |  | **T•A-T** |  | 1.987 | 1.939 |  | 1.995 | 0.034 |  | 1.902 | 2.153 | 2.028 | 0.177 |
| **1BWG-10** |  | **C_+_•G-C** |  | 2.067 | 2.008 | 2.023 | 2.057 | 0.031 |  | 1.971 | 2.218 | 2.095 | 0.175 |
|  |  | **T•A-T** |  | 2.098 | 1.880 |  | 1.989 | 0.154 |  | 1.987 | 1.942 | 1.965 | 0.032 |
|  |  | **C^+^•G-C** |  | 1.978 | 2.031 | 2.075 | 1.995 | 0.049 |  | 2.016 | 1.875 | 1.946 | 0.100 |
|  |  | **T•A-T** |  | 2.103 | 1.934 |  | 2.019 | 0.120 |  | 1.995 | 2.045 | 2.020 | 0.035 |
|  |  | **C^+^•G-C** |  | 2.058 | 2.017 | 2.012 | 2.034 | 0.025 |  | 2.003 | 2.078 | 2.041 | 0.053 |
|  |  | **T•A-T** |  | 2.016 | 1.942 |  | 1.998 | 0.052 |  | 1.898 | 2.136 | 2.017 | 0.168 |
| **1D3X-1** |  | **T•A-T** |  | 1.813 | 1.947 |  | 1.888 | 0.095 |  | 1.729 | 2.063 | 1.896 | 0.236 |
|  |  | **C^+^•G-C** |  | 1.657 | 1.913 | 1.972 | 1.896 | 0.167 |  | 1.878 | 2.061 | 1.970 | 0.129 |
|  |  | **T•A-T** |  | 1.608 | 1.990 |  | 1.902 | 0.270 |  | 1.930 | 2.078 | 2.004 | 0.105 |
|  |  | **C^+^•G-C** |  | 1.819 | 1.946 | 1.914 | 1.846 | 0.066 |  | 1.643 | 1.910 | 1.777 | 0.189 |
|  |  | **T•A-T** |  | 1.868 | 1.842 |  | 1.875 | 0.018 |  | 1.736 | 2.053 | 1.895 | 0.224 |
|  |  | **C^+^•G-C** |  | 1.814 | 1.918 | 1.855 | 1.902 | 0.052 |  | 1.871 | 2.051 | 1.961 | 0.127 |
|  |  | **T•A-T** |  | 1.928 | 2.016 |  | 1.941 | 0.062 |  | 1.778 | 2.043 | 1.911 | 0.187 |
|  |  | **T•A-T** |  | 1.861 | 1.928 |  | 1.895 | 0.047 |  | 1.837 | 2.023 | 1.930 | 0.132 |
| **1D3X-2** |  | **T•A-T** |  | 1.943 | 1.944 |  | 1.962 | 0.001 |  | 1.883 | 2.078 | 1.981 | 0.138 |
|  |  | **C^+^•G-C** |  | 1.648 | 1.892 | 1.953 | 1.874 | 0.161 |  | 1.925 | 1.952 | 1.939 | 0.019 |
|  |  | **T•A-T** |  | 1.623 | 1.917 |  | 1.880 | 0.208 |  | 1.883 | 2.095 | 1.989 | 0.150 |
|  |  | **C^+^•G-C** |  | 1.874 | 1.976 | 1.943 | 1.918 | 0.052 |  | 1.790 | 2.005 | 1.898 | 0.152 |
|  |  | **T•A-T** |  | 2.000 | 1.985 |  | 2.008 | 0.011 |  | 1.743 | 2.040 | 2.040 | 0.210 |
|  |  | **C^+^•G-C** |  | 1.685 | 1.930 | 1.967 | 1.903 | 0.153 |  | 1.878 | 2.055 | 1.967 | 0.125 |
|  |  | **T•A-T** |  | 1.792 | 2.011 |  | 1.895 | 0.155 |  | 1.795 | 1.981 | 1.888 | 0.132 |
|  |  | **T•A-T** |  | 1.554 | 1.939 |  | 1.747 | 0.272 |  | 1.830 | 2.026 | 1.928 | 0.139 |
| **1D3X-3** |  | **T•A-T** |  | 1.876 | 1.981 |  | 1.875 | 0.074 |  | 1.578 | 2.064 | 1.821 | 0.344 |
|  |  | **C^+^•G-C** |  | 1.740 | 1.922 | 1.920 | 1.908 | 0.105 |  | 1.929 | 2.030 | 1.980 | 0.071 |
|  |  | **T•A-T** |  | 1.501 | 1.997 |  | 1.848 | 0.351 |  | 1.810 | 2.083 | 1.947 | 0.193 |
|  |  | **C^+^•G-C** |  | 1.676 | 1.980 | 1.989 | 1.810 | 0.178 |  | 1.679 | 1.724 | 1.702 | 0.032 |
|  |  | **T•A-T** |  | 1.907 | 2.035 |  | 1.941 | 0.091 |  | 1.738 | 2.085 | 1.912 | 0.245 |
|  |  | **C^+^•G-C** |  | 1.701 | 1.894 | 1.900 | 1.886 | 0.113 |  | 1.924 | 2.013 | 1.969 | 0.063 |
|  |  | **T•A-T** |  | 1.690 | 2.021 |  | 1.896 | 0.234 |  | 1.823 | 2.048 | 1.936 | 0.159 |
|  |  | **T•A-T** |  | 1.503 | 1.941 |  | 1.722 | 0.310 |  | 1.882 | 2.031 | 1.957 | 0.105 |

Table S7. Experimentally-determined GVA data of triad. WC indicates Watson-Crick base pairs, and HG means Hoogsteen pairs from Cartesian coordinates of RCSB PDB structures.

| **PDB ID** |  | **Triad** |  | **GVA (WC)** | | **Average** | **s.d.** |  | **GVA (HG)** | | **Average** | **s.d.** |
| --- | --- | --- | --- | --- | --- | --- | --- | --- | --- | --- | --- | --- |
| **149D-1** |  | **C^+^•G-C** |  | 55.26 | 47.88 | 76.86 | 5.22 |  | 47.43 | 60.10 | 72.47 | 8.96 |
|  |  | **T•A-T** |  | 55.05 | 56.58 | 68.37 | 1.08 |  | 44.57 | 61.20 | 74.23 | 11.76 |
|  |  | **T•A-T** |  | 59.46 | 55.27 | 65.27 | 2.96 |  | 47.65 | 61.90 | 70.45 | 10.08 |
|  |  | **T•A-T** |  | 58.43 | 56.31 | 65.26 | 1.50 |  | 44.12 | 58.04 | 77.84 | 9.84 |
|  |  | **C+•G-C** |  | 54.95 | 56.20 | 68.85 | 0.88 |  | 44.01 | 62.05 | 73.94 | 12.76 |
|  |  | **C+•G-C** |  | 59.07 | 51.03 | 69.90 | 5.69 |  | 46.90 | 61.50 | 71.60 | 10.32 |
| **149D-2** |  | **C+•G-C** |  | 56.16 | 49.07 | 74.77 | 5.01 |  | 47.63 | 65.71 | 66.66 | 12.78 |
|  |  | **T•A-T** |  | 53.85 | 57.38 | 68.77 | 2.50 |  | 43.12 | 64.47 | 72.41 | 15.10 |
|  |  | **T•A-T** |  | 57.77 | 56.81 | 65.42 | 0.68 |  | 48.09 | 61.92 | 69.99 | 9.78 |
|  |  | **T•A-T** |  | 57.08 | 56.34 | 66.58 | 0.52 |  | 44.75 | 56.51 | 78.74 | 8.32 |
|  |  | **C+•G-C** |  | 53.72 | 57.61 | 68.67 | 2.75 |  | 43.85 | 63.15 | 73.00 | 13.65 |
|  |  | **C+•G-C** |  | 59.57 | 50.08 | 70.35 | 6.71 |  | 48.57 | 57.51 | 73.92 | 6.32 |
| **149D-3** |  | **C+•G-C** |  | 54.77 | 47.10 | 78.13 | 5.42 |  | 45.39 | 66.59 | 68.02 | 14.99 |
|  |  | **T•A-T** |  | 52.74 | 55.37 | 71.89 | 1.86 |  | 44.98 | 63.70 | 71.32 | 13.24 |
|  |  | **T•A-T** |  | 55.03 | 55.30 | 69.67 | 0.19 |  | 48.33 | 60.04 | 71.63 | 8.28 |
|  |  | **T•A-T** |  | 56.88 | 57.49 | 65.63 | 0.43 |  | 42.27 | 54.39 | 83.34 | 8.57 |
|  |  | **C+•G-C** |  | 55.06 | 57.96 | 66.98 | 2.05 |  | 43.29 | 61.58 | 75.13 | 12.93 |
|  |  | **C+•G-C** |  | 58.77 | 51.69 | 69.54 | 5.01 |  | 47.93 | 58.91 | 73.16 | 7.76 |
| **149D-4** |  | **C+•G-C** |  | 58.79 | 54.44 | 66.77 | 3.08 |  | 44.66 | 64.69 | 70.65 | 14.16 |
|  |  | **T•A-T** |  | 57.54 | 60.11 | 62.35 | 1.82 |  | 42.89 | 64.00 | 73.11 | 14.93 |
|  |  | **T•A-T** |  | 57.35 | 57.14 | 65.51 | 0.15 |  | 46.09 | 60.76 | 73.15 | 10.37 |
|  |  | **T•A-T** |  | 53.19 | 56.33 | 70.48 | 2.22 |  | 43.52 | 55.89 | 80.59 | 8.75 |
|  |  | **C+•G-C** |  | 54.68 | 56.86 | 68.46 | 1.54 |  | 44.16 | 62.06 | 73.78 | 12.66 |
|  |  | **C+•G-C** |  | 58.23 | 51.43 | 70.34 | 4.81 |  | 48.66 | 59.76 | 71.58 | 7.85 |
| **149D-5** |  | **C+•G-C** |  | 55.50 | 50.42 | 74.08 | 3.59 |  | 44.24 | 69.24 | 66.52 | 17.68 |
|  |  | **T•A-T** |  | 54.90 | 58.18 | 66.92 | 2.32 |  | 47.73 | 62.08 | 70.19 | 10.15 |
|  |  | **T•A-T** |  | 57.78 | 57.92 | 64.30 | 0.10 |  | 48.42 | 61.49 | 70.09 | 9.24 |
|  |  | **T•A-T** |  | 56.97 | 57.28 | 65.75 | 0.22 |  | 43.54 | 56.15 | 80.31 | 8.92 |
|  |  | **C+•G-C** |  | 55.79 | 58.12 | 66.09 | 1.65 |  | 43.46 | 61.89 | 74.65 | 13.03 |
|  |  | **C+•G-C** |  | 59.04 | 49.99 | 70.97 | 6.40 |  | 51.20 | 57.49 | 71.31 | 4.45 |
| **149D-6** |  | **C+•G-C** |  | 56.21 | 51.02 | 72.77 | 3.67 |  | 42.54 | 66.17 | 71.29 | 16.71 |
|  |  | **T•A-T** |  | 55.00 | 56.49 | 68.51 | 1.05 |  | 47.63 | 61.56 | 70.81 | 9.85 |
|  |  | **T•A-T** |  | 59.21 | 56.05 | 64.74 | 2.23 |  | 47.38 | 62.33 | 70.29 | 10.57 |
|  |  | **T•A-T** |  | 55.19 | 55.42 | 69.39 | 0.16 |  | 44.14 | 56.83 | 79.03 | 8.97 |
|  |  | **C+•G-C** |  | 53.51 | 54.54 | 71.95 | 0.73 |  | 44.12 | 61.79 | 74.09 | 12.49 |
|  |  | **C+•G-C** |  | 58.55 | 50.40 | 71.05 | 5.76 |  | 48.08 | 60.43 | 71.49 | 8.73 |
| **149D-7** |  | **C+•G-C** |  | 55.77 | 51.97 | 72.26 | 2.69 |  | 44.04 | 66.70 | 69.26 | 16.02 |
|  |  | **T•A-T** |  | 53.77 | 56.72 | 69.51 | 2.09 |  | 46.16 | 61.44 | 72.40 | 10.80 |
|  |  | **T•A-T** |  | 61.24 | 54.35 | 64.41 | 4.87 |  | 48.11 | 61.56 | 70.33 | 9.51 |
|  |  | **T•A-T** |  | 55.26 | 56.19 | 68.55 | 0.66 |  | 44.46 | 55.86 | 79.68 | 8.06 |
|  |  | **C+•G-C** |  | 54.56 | 56.29 | 69.15 | 1.22 |  | 44.83 | 59.38 | 75.79 | 10.29 |
|  |  | **C+•G-C** |  | 58.28 | 49.73 | 71.99 | 6.05 |  | 49.52 | 57.35 | 73.13 | 5.54 |
| **149D-8** |  | **C+•G-C** |  | 57.66 | 51.50 | 70.84 | 4.36 |  | 44.64 | 63.21 | 72.15 | 13.13 |
|  |  | **T•A-T** |  | 55.33 | 54.87 | 69.80 | 0.33 |  | 48.17 | 59.99 | 71.84 | 8.36 |
|  |  | **T•A-T** |  | 61.06 | 61.37 | 57.57 | 0.22 |  | 46.38 | 61.95 | 71.67 | 11.01 |
|  |  | **T•A-T** |  | 54.15 | 54.38 | 71.47 | 0.16 |  | 45.22 | 58.19 | 76.59 | 9.17 |
|  |  | **C+•G-C** |  | 54.08 | 53.40 | 72.52 | 0.48 |  | 42.95 | 63.99 | 73.06 | 14.88 |
|  |  | **C+•G-C** |  | 61.58 | 50.28 | 68.14 | 7.99 |  | 46.62 | 65.88 | 67.50 | 13.62 |
| **149D-9** |  | **C+•G-C** |  | 56.33 | 53.12 | 70.55 | 2.27 |  | 44.73 | 65.66 | 69.61 | 14.80 |
|  |  | **T•A-T** |  | 56.73 | 60.84 | 62.43 | 2.91 |  | 45.43 | 63.89 | 70.68 | 13.05 |
|  |  | **T•A-T** |  | 57.83 | 56.59 | 65.58 | 0.88 |  | 48.28 | 62.45 | 69.27 | 10.02 |
|  |  | **T•A-T** |  | 53.62 | 55.26 | 71.12 | 1.16 |  | 44.73 | 56.68 | 78.59 | 8.45 |
|  |  | **C+•G-C** |  | 52.44 | 53.19 | 74.37 | 0.53 |  | 42.57 | 61.02 | 76.41 | 13.05 |
|  |  | **C+•G-C** |  | 58.88 | 51.76 | 69.36 | 5.03 |  | 45.16 | 64.68 | 70.16 | 13.80 |

| **PDB ID** |  | **Triad** |  | **GVA (WC)** | | **Average** | **s.d.** |  | **GVA (HG)** | | **Average** | **s.d.** |
| --- | --- | --- | --- | --- | --- | --- | --- | --- | --- | --- | --- | --- |
| **149D-10** |  | **C+•G-C** |  | 55.39 | 51.39 | 73.22 | 2.83 |  | 46.88 | 64.93 | 68.19 | 12.76 |
|  |  | **T•A-T** |  | 54.57 | 52.47 | 72.96 | 1.48 |  | 47.48 | 64.04 | 68.48 | 11.71 |
|  |  | **T•A-T** |  | 60.54 | 61.26 | 58.20 | 0.51 |  | 45.26 | 59.27 | 75.47 | 9.91 |
|  |  | **T•A-T** |  | 54.19 | 56.02 | 69.79 | 1.29 |  | 43.20 | 56.87 | 79.93 | 9.67 |
|  |  | **C+•G-C** |  | 50.96 | 51.98 | 77.06 | 0.72 |  | 43.68 | 61.86 | 74.46 | 12.86 |
|  |  | **C+•G-C** |  | 58.41 | 51.73 | 69.86 | 4.72 |  | 45.21 | 62.86 | 71.93 | 12.48 |
| **149D-11** |  | **C+•G-C** |  | 60.12 | 53.28 | 66.60 | 4.84 |  | 44.82 | 66.46 | 68.72 | 15.30 |
|  |  | **T•A-T** |  | 56.74 | 60.35 | 62.91 | 2.55 |  | 44.59 | 62.81 | 72.60 | 12.88 |
|  |  | **T•A-T** |  | 59.15 | 57.89 | 62.96 | 0.89 |  | 46.12 | 61.28 | 72.60 | 10.72 |
|  |  | **T•A-T** |  | 53.23 | 55.47 | 71.30 | 1.58 |  | 44.36 | 57.30 | 78.34 | 9.15 |
|  |  | **C+•G-C** |  | 52.72 | 50.25 | 77.03 | 1.75 |  | 44.36 | 61.43 | 74.21 | 12.07 |
|  |  | **C+•G-C** |  | 58.42 | 48.02 | 73.56 | 7.35 |  | 47.91 | 62.91 | 69.18 | 10.61 |
| **149D-12** |  | **C+•G-C** |  | 59.68 | 51.48 | 68.84 | 5.80 |  | 46.61 | 65.28 | 68.11 | 13.20 |
|  |  | **T•A-T** |  | 57.47 | 59.33 | 63.20 | 1.32 |  | 46.75 | 62.51 | 70.74 | 11.14 |
|  |  | **T•A-T** |  | 57.42 | 56.95 | 65.63 | 0.33 |  | 47.29 | 62.76 | 69.95 | 10.94 |
|  |  | **T•A-T** |  | 53.74 | 55.61 | 70.65 | 1.32 |  | 42.58 | 57.28 | 80.14 | 10.39 |
|  |  | **C+•G-C** |  | 53.19 | 51.45 | 75.36 | 1.23 |  | 42.83 | 61.23 | 75.94 | 13.01 |
|  |  | **C+•G-C** |  | 60.93 | 47.99 | 71.08 | 9.15 |  | 48.37 | 59.62 | 72.01 | 7.95 |
| **149D-13** |  | **C+•G-C** |  | 58.81 | 51.38 | 69.81 | 5.25 |  | 47.18 | 66.48 | 66.34 | 13.65 |
|  |  | **T•A-T** |  | 57.13 | 58.90 | 63.97 | 1.25 |  | 45.86 | 61.46 | 72.68 | 11.03 |
|  |  | **T•A-T** |  | 58.01 | 57.84 | 64.15 | 0.12 |  | 47.46 | 61.25 | 71.29 | 9.75 |
|  |  | **T•A-T** |  | 53.65 | 54.40 | 71.95 | 0.53 |  | 45.48 | 58.21 | 76.31 | 9.00 |
|  |  | **C+•G-C** |  | 53.16 | 54.04 | 72.80 | 0.62 |  | 43.27 | 60.73 | 76.00 | 12.35 |
|  |  | **C+•G-C** |  | 59.22 | 49.69 | 71.09 | 6.74 |  | 48.83 | 63.44 | 67.73 | 10.33 |
| **149D-14** |  | **C+•G-C** |  | 55.08 | 49.49 | 75.43 | 3.95 |  | 46.84 | 65.61 | 67.55 | 13.27 |
|  |  | **T•A-T** |  | 52.31 | 57.10 | 70.59 | 3.39 |  | 46.56 | 62.41 | 71.03 | 11.21 |
|  |  | **T•A-T** |  | 58.72 | 58.07 | 63.21 | 0.46 |  | 46.26 | 61.87 | 71.87 | 11.04 |
|  |  | **T•A-T** |  | 55.64 | 57.78 | 66.58 | 1.51 |  | 43.08 | 57.89 | 79.03 | 10.47 |
|  |  | **C+•G-C** |  | 54.47 | 54.50 | 71.03 | 0.02 |  | 41.31 | 61.90 | 76.79 | 14.56 |
|  |  | **C+•G-C** |  | 57.79 | 51.28 | 70.93 | 4.60 |  | 46.27 | 61.69 | 72.04 | 10.90 |
| **1BWG-1** |  | **C+•G-C** |  | 52.64 | 54.87 | 72.49 | 1.58 |  | 41.74 | 52.71 | 85.55 | 7.76 |
|  |  | **T•A-T** |  | 57.00 | 54.36 | 68.64 | 1.87 |  | 45.26 | 63.83 | 70.91 | 13.13 |
|  |  | **C+•G-C** |  | 57.08 | 47.23 | 75.69 | 6.97 |  | 48.87 | 58.42 | 72.71 | 6.75 |
|  |  | **T•A-T** |  | 55.41 | 50.77 | 73.82 | 3.28 |  | 47.03 | 61.15 | 71.82 | 9.98 |
|  |  | **C+•G-C** |  | 56.57 | 50.24 | 73.19 | 4.48 |  | 46.17 | 49.76 | 84.07 | 2.54 |
|  |  | **T•A-T** |  | 49.56 | 55.80 | 74.64 | 4.41 |  | 43.20 | 55.24 | 81.56 | 8.51 |
| **1BWG-2** |  | **C+•G-C** |  | 51.99 | 56.80 | 71.21 | 3.40 |  | 40.79 | 53.32 | 85.89 | 8.86 |
|  |  | **T•A-T** |  | 56.55 | 55.83 | 67.62 | 0.51 |  | 44.43 | 60.35 | 75.22 | 11.26 |
|  |  | **C+•G-C** |  | 57.06 | 46.58 | 76.36 | 7.41 |  | 48.55 | 57.93 | 73.52 | 6.63 |
|  |  | **T•A-T** |  | 51.60 | 49.61 | 78.79 | 1.41 |  | 47.60 | 62.15 | 70.25 | 10.29 |
|  |  | **C+•G-C** |  | 58.17 | 49.47 | 72.36 | 6.15 |  | 45.91 | 50.69 | 83.40 | 3.38 |
|  |  | **T•A-T** |  | 50.53 | 56.31 | 73.16 | 4.09 |  | 43.34 | 55.41 | 81.25 | 8.53 |
| **1BWG-3** |  | **C+•G-C** |  | 53.20 | 54.05 | 72.75 | 0.60 |  | 41.50 | 53.29 | 85.21 | 8.34 |
|  |  | **T•A-T** |  | 57.15 | 54.28 | 68.57 | 2.03 |  | 43.73 | 61.09 | 75.18 | 12.28 |
|  |  | **C+•G-C** |  | 56.80 | 46.25 | 76.95 | 7.46 |  | 48.61 | 57.25 | 74.14 | 6.11 |
|  |  | **T•A-T** |  | 52.73 | 49.79 | 77.48 | 2.08 |  | 47.28 | 60.99 | 71.73 | 9.69 |
|  |  | **C+•G-C** |  | 58.93 | 49.77 | 71.30 | 6.48 |  | 45.56 | 51.21 | 83.23 | 4.00 |
|  |  | **T•A-T** |  | 48.60 | 57.17 | 74.23 | 6.06 |  | 43.87 | 54.87 | 81.26 | 7.78 |
| **1BWG-4** |  | **C+•G-C** |  | 52.02 | 56.53 | 71.45 | 3.19 |  | 40.63 | 53.42 | 85.95 | 9.04 |
|  |  | **T•A-T** |  | 56.48 | 55.64 | 67.88 | 0.59 |  | 44.53 | 60.08 | 75.39 | 11.00 |
|  |  | **C+•G-C** |  | 57.17 | 47.07 | 75.76 | 7.14 |  | 48.27 | 56.60 | 75.13 | 5.89 |
|  |  | **T•A-T** |  | 49.16 | 50.35 | 80.49 | 0.84 |  | 48.60 | 61.39 | 70.01 | 9.04 |
|  |  | **C+•G-C** |  | 57.73 | 49.60 | 72.67 | 5.75 |  | 45.48 | 53.70 | 80.82 | 5.81 |
|  |  | **T•A-T** |  | 52.04 | 57.17 | 70.79 | 3.63 |  | 43.92 | 54.67 | 81.41 | 7.60 |

| **PDB ID** |  | **Triad** |  | **GVA (WC)** | | **Average** | **s.d.** |  | **GVA (HG)** | | **Average** | **s.d.** |
| --- | --- | --- | --- | --- | --- | --- | --- | --- | --- | --- | --- | --- |
| **1BWG-5** |  | **C+•G-C** |  | 52.55 | 54.84 | 72.61 | 1.62 |  | 41.70 | 52.75 | 85.55 | 7.81 |
|  |  | **T•A-T** |  | 56.93 | 54.10 | 68.97 | 2.00 |  | 45.13 | 63.69 | 71.18 | 13.12 |
|  |  | **C+•G-C** |  | 56.40 | 46.77 | 76.83 | 6.81 |  | 48.69 | 57.96 | 73.35 | 6.55 |
|  |  | **T•A-T** |  | 52.78 | 49.93 | 77.29 | 2.02 |  | 47.65 | 60.36 | 71.99 | 8.99 |
|  |  | **C+•G-C** |  | 59.06 | 50.01 | 70.93 | 6.40 |  | 45.48 | 51.17 | 83.35 | 4.02 |
|  |  | **T•A-T** |  | 48.53 | 57.24 | 74.23 | 6.16 |  | 43.95 | 54.97 | 81.08 | 7.79 |
| **1BWG-6** |  | **C+•G-C** |  | 52.14 | 56.79 | 71.07 | 3.29 |  | 41.23 | 53.59 | 85.18 | 8.74 |
|  |  | **T•A-T** |  | 56.86 | 56.02 | 67.12 | 0.59 |  | 44.31 | 61.13 | 74.56 | 11.89 |
|  |  | **C+•G-C** |  | 55.48 | 46.80 | 77.72 | 6.14 |  | 49.00 | 57.25 | 73.75 | 5.83 |
|  |  | **T•A-T** |  | 49.27 | 54.06 | 76.67 | 3.39 |  | 46.39 | 57.96 | 75.65 | 8.18 |
|  |  | **C+•G-C** |  | 58.72 | 50.86 | 70.42 | 5.56 |  | 45.79 | 50.48 | 83.73 | 3.32 |
|  |  | **T•A-T** |  | 46.79 | 58.76 | 74.45 | 8.46 |  | 44.05 | 55.92 | 80.03 | 8.39 |
| **1BWG-7** |  | **C+•G-C** |  | 56.93 | 51.75 | 71.32 | 3.66 |  | 40.44 | 52.80 | 86.76 | 8.74 |
|  |  | **T•A-T** |  | 56.78 | 55.72 | 67.50 | 0.75 |  | 45.83 | 61.85 | 72.32 | 11.33 |
|  |  | **C+•G-C** |  | 56.79 | 49.68 | 73.53 | 5.03 |  | 48.73 | 57.09 | 74.18 | 5.91 |
|  |  | **T•A-T** |  | 54.73 | 49.90 | 75.37 | 3.42 |  | 51.04 | 56.71 | 72.25 | 4.01 |
|  |  | **C+•G-C** |  | 54.64 | 51.43 | 73.93 | 2.27 |  | 45.41 | 53.47 | 81.12 | 5.70 |
|  |  | **T•A-T** |  | 51.29 | 56.96 | 71.75 | 4.01 |  | 43.98 | 54.80 | 81.22 | 7.65 |
| **1BWG-8** |  | **C+•G-C** |  | 52.87 | 55.19 | 71.94 | 1.64 |  | 41.39 | 53.92 | 84.69 | 8.86 |
|  |  | **T•A-T** |  | 57.95 | 54.68 | 67.37 | 2.31 |  | 44.33 | 62.27 | 73.40 | 12.69 |
|  |  | **C+•G-C** |  | 56.39 | 46.68 | 76.93 | 6.87 |  | 49.17 | 60.12 | 70.71 | 7.74 |
|  |  | **T•A-T** |  | 56.18 | 52.18 | 71.64 | 2.83 |  | 45.69 | 58.33 | 75.98 | 8.94 |
|  |  | **C+•G-C** |  | 55.20 | 49.52 | 75.28 | 4.02 |  | 45.80 | 49.30 | 84.90 | 2.47 |
|  |  | **T•A-T** |  | 48.36 | 55.15 | 76.49 | 4.80 |  | 42.65 | 54.78 | 82.57 | 8.58 |
| **1BWG-9** |  | **C+•G-C** |  | 52.73 | 54.75 | 72.52 | 1.43 |  | 41.83 | 52.85 | 85.32 | 7.79 |
|  |  | **T•A-T** |  | 56.84 | 54.30 | 68.86 | 1.80 |  | 45.51 | 63.75 | 70.74 | 12.90 |
|  |  | **C+•G-C** |  | 56.78 | 47.42 | 75.80 | 6.62 |  | 48.92 | 58.80 | 72.28 | 6.99 |
|  |  | **T•A-T** |  | 51.34 | 49.41 | 79.25 | 1.36 |  | 47.22 | 61.99 | 70.79 | 10.44 |
|  |  | **C+•G-C** |  | 57.63 | 48.85 | 73.52 | 6.21 |  | 46.34 | 51.31 | 82.35 | 3.51 |
|  |  | **T•A-T** |  | 50.73 | 56.26 | 73.01 | 3.91 |  | 43.10 | 56.84 | 80.06 | 9.72 |
| **1BWG-10** |  | **C+•G-C** |  | 52.79 | 54.60 | 72.61 | 1.28 |  | 41.69 | 52.56 | 85.75 | 7.69 |
|  |  | **T•A-T** |  | 56.85 | 54.15 | 69.00 | 1.91 |  | 45.24 | 63.62 | 71.14 | 13.00 |
|  |  | **C+•G-C** |  | 57.39 | 47.75 | 74.86 | 6.82 |  | 48.31 | 57.42 | 74.27 | 6.44 |
|  |  | **T•A-T** |  | 54.99 | 50.16 | 74.85 | 3.42 |  | 47.93 | 61.16 | 70.91 | 9.36 |
|  |  | **C+•G-C** |  | 56.53 | 50.34 | 73.13 | 4.38 |  | 45.65 | 51.17 | 83.18 | 3.90 |
|  |  | **T•A-T** |  | 49.68 | 55.72 | 74.60 | 4.27 |  | 43.22 | 55.37 | 81.41 | 8.59 |
| **1D3X-1** |  | **T•A-T** |  | 51.06 | 57.42 | 71.52 | 4.50 |  | 41.88 | 57.92 | 80.20 | 11.34 |
|  |  | **C+•G-C** |  | 47.81 | 49.24 | 82.95 | 1.01 |  | 48.92 | 64.04 | 67.04 | 10.69 |
|  |  | **T•A-T** |  | 47.62 | 40.28 | 92.10 | 5.19 |  | 49.21 | 60.16 | 70.63 | 7.74 |
|  |  | **C+•G-C** |  | 53.23 | 39.76 | 87.01 | 9.52 |  | 49.59 | 54.13 | 76.28 | 3.21 |
|  |  | **T•A-T** |  | 53.60 | 59.04 | 67.36 | 3.85 |  | 40.09 | 60.48 | 79.43 | 14.42 |
|  |  | **C+•G-C** |  | 52.05 | 50.52 | 77.43 | 1.08 |  | 47.20 | 58.44 | 74.36 | 7.95 |
|  |  | **T•A-T** |  | 57.65 | 52.12 | 70.23 | 3.91 |  | 41.61 | 60.70 | 77.69 | 13.50 |
|  |  | **T•A-T** |  | 56.25 | 46.94 | 76.81 | 6.58 |  | 55.99 | 48.91 | 75.10 | 5.01 |
| **1D3X-2** |  | **T•A-T** |  | 52.87 | 62.19 | 64.94 | 6.59 |  | 46.46 | 50.64 | 82.90 | 2.96 |
|  |  | **C+•G-C** |  | 48.64 | 45.78 | 85.58 | 2.02 |  | 54.69 | 60.73 | 64.58 | 4.27 |
|  |  | **T•A-T** |  | 51.46 | 45.99 | 82.55 | 3.87 |  | 49.67 | 53.67 | 76.66 | 2.83 |
|  |  | **C+•G-C** |  | 52.26 | 46.48 | 81.26 | 4.09 |  | 46.65 | 59.60 | 73.75 | 9.16 |
|  |  | **T•A-T** |  | 49.67 | 64.60 | 65.73 | 10.56 |  | 38.62 | 60.71 | 80.67 | 15.62 |
|  |  | **C+•G-C** |  | 50.26 | 43.25 | 86.49 | 4.96 |  | 47.88 | 63.80 | 68.32 | 11.26 |
|  |  | **T•A-T** |  | 53.92 | 47.08 | 79.00 | 4.84 |  | 48.06 | 54.48 | 77.46 | 4.54 |
|  |  | **T•A-T** |  | 47.71 | 42.44 | 89.85 | 3.73 |  | 46.44 | 63.07 | 70.49 | 11.76 |
| **1D3X-3** |  | **T•A-T** |  | 50.31 | 56.89 | 72.80 | 4.65 |  | 38.11 | 54.03 | 87.86 | 11.26 |
|  |  | **C+•G-C** |  | 49.59 | 47.34 | 83.07 | 1.59 |  | 52.20 | 58.56 | 69.24 | 4.50 |
|  |  | **T•A-T** |  | 43.11 | 43.75 | 93.14 | 0.45 |  | 49.85 | 53.48 | 76.67 | 2.57 |
|  |  | **C+•G-C** |  | 46.10 | 42.91 | 90.99 | 2.26 |  | 48.39 | 62.76 | 68.85 | 10.16 |
|  |  | **T•A-T** |  | 47.94 | 56.27 | 75.79 | 5.89 |  | 37.61 | 61.83 | 80.56 | 17.13 |
|  |  | **C+•G-C** |  | 52.85 | 45.58 | 81.57 | 5.14 |  | 49.68 | 61.91 | 68.41 | 8.65 |
|  |  | **T•A-T** |  | 48.69 | 46.36 | 84.95 | 1.65 |  | 48.43 | 53.89 | 77.68 | 3.86 |
|  |  | **T•A-T** |  | 44.84 | 41.80 | 93.36 | 2.15 |  | 53.91 | 50.78 | 75.31 | 2.21 |

| **PDB ID** |  | **Triad** |  | **GVA (WC)** | | **Average** | **s.d.** |  | **GVA (HG)** | | **Average** | **s.d.** |
| --- | --- | --- | --- | --- | --- | --- | --- | --- | --- | --- | --- | --- |
| **1D3X-4** |  | **T•A-T** |  | 56.19 | 62.87 | 60.94 | 4.72 |  | 41.81 | 61.21 | 76.98 | 13.72 |
|  |  | **C+•G-C** |  | 52.25 | 46.14 | 81.61 | 4.32 |  | 50.99 | 69.04 | 59.97 | 12.76 |
|  |  | **T•A-T** |  | 45.63 | 44.71 | 89.66 | 0.65 |  | 48.18 | 55.97 | 75.85 | 5.51 |
|  |  | **C+•G-C** |  | 51.99 | 42.45 | 85.56 | 6.75 |  | 43.67 | 64.49 | 71.84 | 14.72 |
|  |  | **T•A-T** |  | 52.17 | 65.03 | 62.80 | 9.09 |  | 34.88 | 63.17 | 81.95 | 20.00 |
|  |  | **C+•G-C** |  | 48.07 | 47.72 | 84.21 | 0.25 |  | 49.89 | 53.88 | 76.23 | 2.82 |
|  |  | **T•A-T** |  | 51.89 | 46.88 | 81.23 | 3.54 |  | 52.18 | 47.62 | 80.20 | 3.22 |
|  |  | **T•A-T** |  | 48.03 | 43.17 | 88.80 | 3.44 |  | 50.18 | 57.82 | 72.00 | 5.40 |
| **1D3X-5** |  | **T•A-T** |  | 54.92 | 57.74 | 67.34 | 1.99 |  | 44.21 | 43.93 | 91.86 | 0.20 |
|  |  | **C+•G-C** |  | 45.00 | 48.33 | 86.67 | 2.35 |  | 50.27 | 57.36 | 72.37 | 5.01 |
|  |  | **T•A-T** |  | 49.56 | 43.67 | 86.77 | 4.16 |  | 47.24 | 51.25 | 81.51 | 2.84 |
|  |  | **C+•G-C** |  | 48.30 | 42.83 | 88.87 | 3.87 |  | 44.83 | 66.06 | 69.11 | 15.01 |
|  |  | **T•A-T** |  | 55.96 | 57.59 | 66.45 | 1.15 |  | 39.59 | 65.86 | 74.55 | 18.58 |
|  |  | **C+•G-C** |  | 48.27 | 47.84 | 83.89 | 0.30 |  | 50.43 | 55.17 | 74.40 | 3.35 |
|  |  | **T•A-T** |  | 52.39 | 45.44 | 82.17 | 4.91 |  | 46.59 | 56.68 | 76.73 | 7.13 |
|  |  | **T•A-T** |  | 44.77 | 39.59 | 95.64 | 3.66 |  | 51.57 | 62.77 | 65.66 | 7.92 |
| **1D3X-6** |  | **T•A-T** |  | 57.25 | 54.99 | 67.76 | 1.60 |  | 43.29 | 59.80 | 76.91 | 11.67 |
|  |  | **C+•G-C** |  | 47.04 | 47.63 | 85.33 | 0.42 |  | 52.46 | 56.33 | 71.21 | 2.74 |
|  |  | **T•A-T** |  | 47.38 | 42.95 | 89.67 | 3.13 |  | 47.55 | 59.39 | 73.06 | 8.37 |
|  |  | **C+•G-C** |  | 52.46 | 41.74 | 85.80 | 7.58 |  | 50.38 | 59.44 | 70.18 | 6.41 |
|  |  | **T•A-T** |  | 47.27 | 55.97 | 76.76 | 6.15 |  | 37.75 | 61.08 | 81.17 | 16.50 |
|  |  | **C+•G-C** |  | 48.81 | 53.17 | 78.02 | 3.08 |  | 45.13 | 59.99 | 74.88 | 10.51 |
|  |  | **T•A-T** |  | 45.98 | 46.69 | 87.33 | 0.50 |  | 46.85 | 61.69 | 71.46 | 10.49 |
|  |  | **T•A-T** |  | 55.27 | 42.88 | 81.85 | 8.76 |  | 52.37 | 56.45 | 71.18 | 2.88 |
| **1D3X-7** |  | **T•A-T** |  | 50.32 | 53.17 | 76.51 | 2.02 |  | 38.28 | 54.94 | 86.78 | 11.78 |
|  |  | **C+•G-C** |  | 53.96 | 49.98 | 76.06 | 2.81 |  | 48.17 | 57.65 | 74.18 | 6.70 |
|  |  | **T•A-T** |  | 42.08 | 51.96 | 85.96 | 6.99 |  | 45.26 | 55.81 | 78.93 | 7.46 |
|  |  | **C+•G-C** |  | 47.67 | 40.46 | 91.87 | 5.10 |  | 55.77 | 68.46 | 55.77 | 8.97 |
|  |  | **T•A-T** |  | 50.97 | 51.95 | 77.08 | 0.69 |  | 42.40 | 65.07 | 72.53 | 16.03 |
|  |  | **C+•G-C** |  | 53.48 | 45.86 | 80.66 | 5.39 |  | 50.54 | 62.86 | 66.60 | 8.71 |
|  |  | **T•A-T** |  | 58.13 | 47.75 | 74.12 | 7.34 |  | 48.09 | 60.98 | 70.93 | 9.11 |
|  |  | **T•A-T** |  | 47.08 | 43.47 | 89.45 | 2.55 |  | 49.33 | 71.93 | 58.74 | 15.98 |
| **1D3X-8** |  | **T•A-T** |  | 57.13 | 51.89 | 70.98 | 3.71 |  | 43.36 | 45.56 | 91.08 | 1.56 |
|  |  | **C+•G-C** |  | 47.80 | 52.28 | 79.92 | 3.17 |  | 48.04 | 54.99 | 76.97 | 4.91 |
|  |  | **T•A-T** |  | 55.93 | 47.15 | 76.92 | 6.21 |  | 44.95 | 50.90 | 84.15 | 4.21 |
|  |  | **C+•G-C** |  | 42.43 | 43.08 | 94.49 | 0.46 |  | 52.16 | 61.17 | 66.67 | 6.37 |
|  |  | **T•A-T** |  | 45.38 | 58.67 | 75.95 | 9.40 |  | 38.41 | 69.09 | 72.50 | 21.69 |
|  |  | **C+•G-C** |  | 44.22 | 46.27 | 89.51 | 1.45 |  | 50.89 | 53.94 | 75.17 | 2.16 |
|  |  | **T•A-T** |  | 46.05 | 46.68 | 87.27 | 0.45 |  | 50.33 | 49.86 | 79.81 | 0.33 |
|  |  | **T•A-T** |  | 50.76 | 42.05 | 87.19 | 6.16 |  | 51.25 | 58.61 | 70.14 | 5.20 |
| **1D3X-9** |  | **T•A-T** |  | 45.65 | 62.12 | 72.23 | 11.65 |  | 40.39 | 51.54 | 88.07 | 7.88 |
|  |  | **C+•G-C** |  | 53.49 | 45.75 | 80.76 | 5.47 |  | 52.45 | 66.14 | 61.41 | 9.68 |
|  |  | **T•A-T** |  | 48.61 | 41.85 | 89.54 | 4.78 |  | 51.08 | 56.32 | 72.60 | 3.71 |
|  |  | **C+•G-C** |  | 51.01 | 42.49 | 86.50 | 6.02 |  | 47.55 | 62.63 | 69.82 | 10.66 |
|  |  | **T•A-T** |  | 44.07 | 57.60 | 78.33 | 9.57 |  | 39.89 | 59.28 | 80.83 | 13.71 |
|  |  | **C+•G-C** |  | 49.80 | 46.18 | 84.02 | 2.56 |  | 46.24 | 57.09 | 76.67 | 7.67 |
|  |  | **T•A-T** |  | 56.83 | 48.27 | 74.90 | 6.05 |  | 41.35 | 56.39 | 82.26 | 10.63 |
|  |  | **T•A-T** |  | 53.57 | 49.75 | 76.68 | 2.70 |  | 44.57 | 58.03 | 77.40 | 9.52 |
| **1D3X-10** |  | **T•A-T** |  | 56.51 | 55.28 | 68.21 | 0.87 |  | 41.85 | 51.34 | 86.81 | 6.71 |
|  |  | **C+•G-C** |  | 56.55 | 46.02 | 77.43 | 7.45 |  | 51.97 | 58.10 | 69.93 | 4.33 |
|  |  | **T•A-T** |  | 48.83 | 50.44 | 80.73 | 1.14 |  | 47.24 | 55.48 | 77.28 | 5.83 |
|  |  | **C+•G-C** |  | 53.83 | 44.08 | 82.09 | 6.89 |  | 48.29 | 58.95 | 72.76 | 7.54 |
|  |  | **T•A-T** |  | 45.68 | 57.12 | 77.20 | 8.09 |  | 39.08 | 59.78 | 81.14 | 14.64 |
|  |  | **C+•G-C** |  | 57.18 | 48.95 | 73.87 | 5.82 |  | 44.68 | 60.18 | 75.14 | 10.96 |
|  |  | **T•A-T** |  | 50.65 | 47.77 | 81.58 | 2.04 |  | 43.49 | 59.73 | 76.78 | 11.48 |
|  |  | **T•A-T** |  | 49.73 | 45.41 | 84.86 | 3.05 |  | 56.35 | 48.46 | 75.19 | 5.58 |

**Table S8.** Experimentally-determined TA data of triplex. WC indicates Watson-Crick base pairs, and HG means Hoogsteen pairs from Cartesian coordinates of RCSB PDB structures.

| **PDB ID** |  | **Triplex** |  | **TA (WC)** | **TA (HG)** |  | **PDB ID** |  | **Triplex** |  | **TA (WC)** | **TA (HG)** |
| --- | --- | --- | --- | --- | --- | --- | --- | --- | --- | --- | --- | --- |
| **149D-1** |  | **C+•G-C/T•A-T** |  | 26.6 | 33.4 |  | **149D-11** |  | **C+•G-C/T•A-T** |  | 24.1 | 32.3 |
|  |  | **T•A-T/T•A-T** |  | 28.3 | 29.7 |  |  |  | **T•A-T/T•A-T** |  | 30.4 | 28.6 |
|  |  | **T•A-T/C+•G-C** |  | 32.1 | 32.7 |  |  |  | **T•A-T/C+•G-C** |  | 35.2 | 30.4 |
|  |  | **T•A-T/C+•G-C** |  | 34.7 | 31.7 |  |  |  | **T•A-T/C+•G-C** |  | 32.9 | 32.9 |
| **149D-2** |  | **C+•G-C/T•A-T** |  | 26.2 | 30.9 |  | **149D-12** |  | **C+•G-C/T•A-T** |  | 25.3 | 35.1 |
|  |  | **T•A-T/T•A-T** |  | 28.6 | 32.5 |  |  |  | **T•A-T/T•A-T** |  | 28.4 | 25.3 |
|  |  | **T•A-T/C+•G-C** |  | 29.7 | 31.6 |  |  |  | **T•A-T/C+•G-C** |  | 34.5 | 31.1 |
|  |  | **T•A-T/C+•G-C** |  | 36.9 | 33.0 |  |  |  | **T•A-T/C+•G-C** |  | 33.9 | 34.6 |
| **149D-3** |  | **C+•G-C/T•A-T** |  | 26.8 | 35.9 |  | **149D-13** |  | **C+•G-C/T•A-T** |  | 27.8 | 35.5 |
|  |  | **T•A-T/T•A-T** |  | 29.0 | 31.9 |  |  |  | **T•A-T/T•A-T** |  | 29.0 | 28.1 |
|  |  | **T•A-T/C+•G-C** |  | 30.6 | 33.1 |  |  |  | **T•A-T/C+•G-C** |  | 32.9 | 31.0 |
|  |  | **T•A-T/C+•G-C** |  | 35.9 | 33.2 |  |  |  | **T•A-T/C+•G-C** |  | 33.8 | 32.7 |
| **149D-4** |  | **C+•G-C/T•A-T** |  | 27.3 | 32.4 |  | **149D-14** |  | **C+•G-C/T•A-T** |  | 25.8 | 35.0 |
|  |  | **T•A-T/T•A-T** |  | 28.3 | 27.6 |  |  |  | **T•A-T/T•A-T** |  | 29.2 | 29.0 |
|  |  | **T•A-T/C+•G-C** |  | 31.1 | 33.1 |  |  |  | **T•A-T/C+•G-C** |  | 35.3 | 31.3 |
|  |  | **T•A-T/C+•G-C** |  | 35.2 | 33.2 |  |  |  | **T•A-T/C+•G-C** |  | 31.2 | 31.9 |
| **149D-5** |  | **C+•G-C/T•A-T** |  | 25.9 | 37.9 |  | **1BWG-1** |  | **C+•G-C/T•A-T** |  | 35.5 | 38.8 |
|  |  | **T•A-T/T•A-T** |  | 28.7 | 27.2 |  |  |  | **T•A-T/C+•G-C** |  | 36.6 | 32.8 |
|  |  | **T•A-T/C+•G-C** |  | 31.0 | 33.2 |  |  |  | **C+•G-C/T•A-T** |  | 29.6 | 32.3 |
|  |  | **T•A-T/C+•G-C** |  | 35.3 | 34.1 |  |  |  | **T•A-T/C+•G-C** |  | 37.0 | 35.8 |
| **149D-6** |  | **C+•G-C/T•A-T** |  | 25.2 | 36.1 |  |  |  | **C+•G-C/T•A-T** |  | 30.1 | 32.6 |
|  |  | **T•A-T/T•A-T** |  | 30.0 | 27.9 |  | **1BWG-2** |  | **C+•G-C/T•A-T** |  | 36.4 | 40.3 |
|  |  | **T•A-T/C+•G-C** |  | 30.9 | 31.0 |  |  |  | **T•A-T/C+•G-C** |  | 37.9 | 33.6 |
|  |  | **T•A-T/C+•G-C** |  | 35.3 | 34.1 |  |  |  | **C+•G-C/T•A-T** |  | 28.7 | 31.5 |
| **149D-7** |  | **C+•G-C/T•A-T** |  | 27.3 | 34.7 |  |  |  | **T•A-T/C+•G-C** |  | 37.5 | 35.8 |
|  |  | **T•A-T/T•A-T** |  | 30.5 | 29.6 |  |  |  | **C+•G-C/T•A-T** |  | 28.6 | 32.5 |
|  |  | **T•A-T/C+•G-C** |  | 31.1 | 33.1 |  | **1BWG-3** |  | **C+•G-C/T•A-T** |  | 35.5 | 39.1 |
|  |  | **T•A-T/C+•G-C** |  | 35.7 | 33.2 |  |  |  | **T•A-T/C+•G-C** |  | 37.1 | 34.2 |
| **149D-8** |  | **C+•G-C/T•A-T** |  | 27.4 | 34.2 |  |  |  | **C+•G-C/T•A-T** |  | 29.1 | 32.2 |
|  |  | **T•A-T/T•A-T** |  | 27.4 | 30.1 |  |  |  | **T•A-T/C+•G-C** |  | 37.5 | 36.0 |
|  |  | **T•A-T/C+•G-C** |  | 34.1 | 31.2 |  |  |  | **C+•G-C/T•A-T** |  | 28.1 | 33.3 |
|  |  | **T•A-T/C+•G-C** |  | 33.2 | 31.5 |  | **1BWG-4** |  | **C+•G-C/T•A-T** |  | 36.2 | 40.4 |
| **149D-9** |  | **C+•G-C/T•A-T** |  | 25.8 | 35.3 |  |  |  | **T•A-T/C+•G-C** |  | 38.0 | 34.0 |
|  |  | **T•A-T/T•A-T** |  | 29.9 | 26.5 |  |  |  | **C+•G-C/T•A-T** |  | 27.6 | 31.9 |
|  |  | **T•A-T/C+•G-C** |  | 34.2 | 30.5 |  |  |  | **T•A-T/C+•G-C** |  | 37.7 | 34.0 |
|  |  | **T•A-T/C+•G-C** |  | 33.2 | 32.8 |  |  |  | **C+•G-C/T•A-T** |  | 29.1 | 34.6 |
| **149D-10** |  | **C+•G-C/T•A-T** |  | 24.0 | 33.2 |  | **1BWG-5** |  | **C+•G-C/T•A-T** |  | 35.7 | 38.8 |
|  |  | **T•A-T/T•A-T** |  | 29.7 | 30.3 |  |  |  | **T•A-T/C+•G-C** |  | 36.8 | 32.8 |
|  |  | **T•A-T/C+•G-C** |  | 33.7 | 30.7 |  |  |  | **C+•G-C/T•A-T** |  | 29.4 | 32.4 |
|  |  | **T•A-T/C+•G-C** |  | 33.7 | 33.5 |  |  |  | **T•A-T/C+•G-C** |  | 37.6 | 35.8 |
|  |  |  |  |  |  |  |  |  | **C+•G-C/T•A-T** |  | 28.0 | 33.2 |

| **PDB ID** |  | **Triplex** |  | **TA (WC)** | **TA (HG)** |  | **PDB ID** |  | **Triplex** |  | **TA (WC)** | **TA (HG)** |
| --- | --- | --- | --- | --- | --- | --- | --- | --- | --- | --- | --- | --- |
| **1BWG-6** |  | **C+•G-C/T•A-T** |  | 36.1 | 39.9 |  | **1D3X-4** |  | **T•A-T/C+•G-C** |  | 32.1 | 27.5 |
|  |  | **T•A-T/C+•G-C** |  | 38.7 | 34.7 |  |  |  | **C+•G-C/T•A-T** |  | 33.9 | 29.8 |
|  |  | **C+•G-C/T•A-T** |  | 30.4 | 35.5 |  |  |  | **T•A-T/C+•G-C** |  | 35.8 | 29.1 |
|  |  | **T•A-T/C+•G-C** |  | 39.1 | 35.7 |  |  |  | **C+•G-C/T•A-T** |  | 24.8 | 39.8 |
|  |  | **C+•G-C/T•A-T** |  | 28.0 | 33.4 |  |  |  | **T•A-T/C+•G-C** |  | 33.9 | 36.6 |
| **1BWG-7** |  | **C+•G-C/T•A-T** |  | 31.2 | 40.7 |  |  |  | **C+•G-C/T•A-T** |  | 34.1 | 36.0 |
|  |  | **T•A-T/C+•G-C** |  | 36.7 | 33.8 |  |  |  | **T•A-T/T•A-T** |  | 29.7 | 24.7 |
|  |  | **C+•G-C/T•A-T** |  | 30.1 | 33.4 |  | **1D3X-5** |  | **T•A-T/C+•G-C** |  | 33.4 | 29.8 |
|  |  | **T•A-T/C+•G-C** |  | 37.1 | 33.0 |  |  |  | **C+•G-C/T•A-T** |  | 40.0 | 32.6 |
|  |  | **C+•G-C/T•A-T** |  | 30.8 | 34.6 |  |  |  | **T•A-T/C+•G-C** |  | 33.8 | 31.1 |
| **1BWG-8** |  | **C+•G-C/T•A-T** |  | 35.7 | 39.3 |  |  |  | **C+•G-C/T•A-T** |  | 27.3 | 39.0 |
|  |  | **T•A-T/C+•G-C** |  | 37.4 | 34.3 |  |  |  | **T•A-T/C+•G-C** |  | 31.9 | 35.6 |
|  |  | **C+•G-C/T•A-T** |  | 31.3 | 34.3 |  |  |  | **C+•G-C/T•A-T** |  | 37.6 | 31.0 |
|  |  | **T•A-T/C+•G-C** |  | 38.9 | 36.6 |  |  |  | **T•A-T/T•A-T** |  | 27.9 | 27.2 |
|  |  | **C+•G-C/T•A-T** |  | 31.2 | 33.4 |  | **1D3X-6** |  | **T•A-T/C+•G-C** |  | 26.5 | 30.1 |
| **1BWG-9** |  | **C+•G-C/T•A-T** |  | 35.5 | 39.0 |  |  |  | **C+•G-C/T•A-T** |  | 37.7 | 27.8 |
|  |  | **T•A-T/C+•G-C** |  | 36.4 | 32.8 |  |  |  | **T•A-T/C+•G-C** |  | 35.9 | 37.4 |
|  |  | **C+•G-C/T•A-T** |  | 29.0 | 30.8 |  |  |  | **C+•G-C/T•A-T** |  | 28.7 | 31.6 |
|  |  | **T•A-T/C+•G-C** |  | 38.4 | 36.9 |  |  |  | **T•A-T/C+•G-C** |  | 29.1 | 37.6 |
|  |  | **C+•G-C/T•A-T** |  | 28.9 | 32.6 |  |  |  | **C+•G-C/T•A-T** |  | 34.1 | 29.3 |
| **1BWG-10** |  | **C+•G-C/T•A-T** |  | 35.4 | 38.8 |  |  |  | **T•A-T/T•A-T** |  | 33.2 | 35.4 |
|  |  | **T•A-T/C+•G-C** |  | 36.8 | 33.3 |  | **1D3X-7** |  | **T•A-T/C+•G-C** |  | 29.1 | 37.1 |
|  |  | **C+•G-C/T•A-T** |  | 28.9 | 31.8 |  |  |  | **C+•G-C/T•A-T** |  | 29.8 | 28.5 |
|  |  | **T•A-T/C+•G-C** |  | 37.2 | 35.2 |  |  |  | **T•A-T/C+•G-C** |  | 29.5 | 35.2 |
|  |  | **C+•G-C/T•A-T** |  | 30.2 | 33.0 |  |  |  | **C+•G-C/T•A-T** |  | 29.6 | 31.6 |
| **1D3X-1** |  | **T•A-T/C+•G-C** |  | 37.1 | 35.9 |  |  |  | **T•A-T/C+•G-C** |  | 34.2 | 36.9 |
|  |  | **C+•G-C/T•A-T** |  | 32.7 | 24.8 |  |  |  | **C+•G-C/T•A-T** |  | 32.9 | 32.3 |
|  |  | **T•A-T/C+•G-C** |  | 37.4 | 35.5 |  |  |  | **T•A-T/T•A-T** |  | 28.5 | 25.5 |
|  |  | **C+•G-C/T•A-T** |  | 23.9 | 34.7 |  | **1D3X-8** |  | **T•A-T/C+•G-C** |  | 28.4 | 33.7 |
|  |  | **T•A-T/C+•G-C** |  | 35.3 | 37.4 |  |  |  | **C+•G-C/T•A-T** |  | 39.0 | 31.2 |
|  |  | **C+•G-C/T•A-T** |  | 33.9 | 29.7 |  |  |  | **T•A-T/C+•G-C** |  | 30.0 | 35.7 |
|  |  | **T•A-T/T•A-T** |  | 28.5 | 38.2 |  |  |  | **C+•G-C/T•A-T** |  | 24.2 | 28.3 |
| **1D3X-2** |  | **T•A-T/C+•G-C** |  | 35.7 | 29.2 |  |  |  | **T•A-T/C+•G-C** |  | 34.1 | 36.7 |
|  |  | **C+•G-C/T•A-T** |  | 35.3 | 30.2 |  |  |  | **C+•G-C/T•A-T** |  | 35.0 | 34.9 |
|  |  | **T•A-T/C+•G-C** |  | 35.5 | 34.7 |  |  |  | **T•A-T/T•A-T** |  | 30.2 | 26.8 |
|  |  | **C+•G-C/T•A-T** |  | 24.4 | 35.8 |  | **1D3X-9** |  | **T•A-T/C+•G-C** |  | 36.7 | 35.1 |
|  |  | **T•A-T/C+•G-C** |  | 36.3 | 31.1 |  |  |  | **C+•G-C/T•A-T** |  | 34.4 | 28.5 |
|  |  | **C+•G-C/T•A-T** |  | 35.0 | 38.8 |  |  |  | **T•A-T/C+•G-C** |  | 35.2 | 33.0 |
|  |  | **T•A-T/T•A-T** |  | 30.1 | 24.1 |  |  |  | **C+•G-C/T•A-T** |  | 28.1 | 37.5 |
| **1D3X-3** |  | **T•A-T/C+•G-C** |  | 35.5 | 40.4 |  |  |  | **T•A-T/C+•G-C** |  | 34.9 | 32.4 |
|  |  | **C+•G-C/T•A-T** |  | 32.2 | 26.4 |  |  |  | **C+•G-C/T•A-T** |  | 37.9 | 34.9 |
|  |  | **T•A-T/C+•G-C** |  | 34.3 | 34.0 |  |  |  | **T•A-T/T•A-T** |  | 31.2 | 36.2 |
|  |  | **C+•G-C/T•A-T** |  | 28.9 | 33.7 |  | **1D3X-10** |  | **T•A-T/C+•G-C** |  | 32.2 | 34.0 |
|  |  | **T•A-T/C+•G-C** |  | 32.3 | 38.1 |  |  |  | **C+•G-C/T•A-T** |  | 28.3 | 27.7 |
|  |  | **C+•G-C/T•A-T** |  | 34.6 | 33.6 |  |  |  | **T•A-T/C+•G-C** |  | 38.8 | 35.1 |
|  |  | **T•A-T/T•A-T** |  | 28.6 | 30.5 |  |  |  | **C+•G-C/T•A-T** |  | 25.5 | 30.8 |
|  |  |  |  |  |  |  |  |  | **T•A-T/C+•G-C** |  | 32.1 | 32.7 |
|  |  |  |  |  |  |  |  |  | **C+•G-C/T•A-T** |  | 29.0 | 26.6 |
|  |  |  |  |  |  |  |  |  | **T•A-T/T•A-T** |  | 28.2 | 40.9 |

Table S9. Experimentally-determined NH-N, NH-O, M^+^∙∙∙O data of quadruplex from Cartesian coordinates of RCSB PDB structures.

| **PDB ID** |  | **Metal ion** |  | **NH-N** | **NH-O** | **M^+^∙∙∙O** |  | **PDB ID** |  | **Metal ion** |  | **NH-N** | **NH-O** | **M^+^∙∙∙O** |
| --- | --- | --- | --- | --- | --- | --- | --- | --- | --- | --- | --- | --- | --- | --- |
| **1JB7** |  | **Na^+^** |  | 1.82287 | 2.02005 | 2.403 |  | **2AQY-5** |  | **Na^+^** |  | 1.884 | 1.89 |  |
|  |  |  |  | 1.8621 | 1.70483 | 2.727 |  |  |  |  |  | 1.871 | 1.898 |  |
|  |  |  |  | 2.14437 | 1.9345 | 2.465 |  |  |  |  |  | 1.843 | 1.872 |  |
|  |  |  |  | 1.58187 | 1.85046 | 2.253 |  |  |  |  |  | 1.841 | 1.872 |  |
|  |  |  |  | 2.014 | 1.875 | 2.385 |  |  |  |  |  | 1.862 | 1.888 |  |
|  |  |  |  | 1.778 | 1.961 | 2.42 |  |  |  |  |  | 1.833 | 1.841 |  |
|  |  |  |  | 1.831 | 1.858 | 2.134 |  |  |  |  |  | 1.894 | 1.844 |  |
|  |  |  |  | 1.882 | 1.818 | 2.27 |  |  |  |  |  | 1.877 | 1.842 |  |
|  |  |  |  | 1.731 | 1.81 | 2.492 |  |  |  |  |  | 1.941 | 1.854 |  |
|  |  |  |  | 1.892 | 1.857 | 2.348 |  |  |  |  |  | 1.853 | 1.85 |  |
|  |  |  |  | 1.919 | 1.915 | 2.265 |  | **2AQY-6** |  | **Na^+^** |  | 1.867 | 1.849 |  |
|  |  |  |  | 1.888 | 1.736 | 2.487 |  |  |  |  |  | 1.844 | 1.849 |  |
| **2AQY-1** |  | **Na^+^** |  | 1.901 | 1.844 |  |  |  |  |  |  | 1.925 | 1.853 |  |
|  |  |  |  | 1.917 | 1.845 |  |  |  |  |  |  | 1.85 | 1.963 |  |
|  |  |  |  | 1.84 | 1.842 |  |  |  |  |  |  | 1.861 | 1.832 |  |
|  |  |  |  | 1.878 | 1.832 |  |  |  |  |  |  | 1.894 | 1.884 |  |
|  |  |  |  | 1.97 | 1.93 |  |  |  |  |  |  | 1.933 | 1.829 |  |
|  |  |  |  | 1.847 | 1.83 |  |  |  |  |  |  | 1.859 | 1.853 |  |
|  |  |  |  | 1.84 | 1.845 |  |  |  |  |  |  | 1.884 | 1.838 |  |
|  |  |  |  | 1.878 | 1.842 |  |  |  |  |  |  | 1.855 | 1.9 |  |
|  |  |  |  | 1.901 | 1.832 |  |  | **2AQY-7** |  | **Na^+^** |  | 1.869 | 1.861 |  |
|  |  |  |  | 1.917 | 1.844 |  |  |  |  |  |  | 1.843 | 1.913 |  |
| **2AQY-2** |  | **Na^+^** |  | 1.853 | 1.904 |  |  |  |  |  |  | 1.876 | 1.873 |  |
|  |  |  |  | 1.851 | 1.846 |  |  |  |  |  |  | 1.87 | 1.889 |  |
|  |  |  |  | 1.881 | 1.843 |  |  |  |  |  |  | 1.918 | 1.967 |  |
|  |  |  |  | 1.854 | 2.017 |  |  |  |  |  |  | 1.95 | 1.846 |  |
|  |  |  |  | 1.841 | 1.865 |  |  |  |  |  |  | 1.969 | 1.847 |  |
|  |  |  |  | 1.872 | 1.83 |  |  |  |  |  |  | 1.87 | 1.89 |  |
|  |  |  |  | 1.956 | 1.99 |  |  |  |  |  |  | 1.847 | 1.871 |  |
|  |  |  |  | 1.843 | 2.049 |  |  |  |  |  |  | 1.871 | 1.847 |  |
|  |  |  |  | 1.885 | 1.834 |  |  | **2AQY-8** |  | **Na^+^** |  | 1.868 | 1.874 |  |
|  |  |  |  | 1.904 | 1.843 |  |  |  |  |  |  | 1.864 | 1.871 |  |
| **2AQY-3** |  | **Na^+^** |  | 1.849 | 1.949 |  |  |  |  |  |  | 1.817 | 1.996 |  |
|  |  |  |  | 1.838 | 1.898 |  |  |  |  |  |  | 1.875 | 1.859 |  |
|  |  |  |  | 1.842 | 1.849 |  |  |  |  |  |  | 1.884 | 1.857 |  |
|  |  |  |  | 1.858 | 1.898 |  |  |  |  |  |  | 1.879 | 1.906 |  |
|  |  |  |  | 1.85 | 1.889 |  |  |  |  |  |  | 1.867 | 1.836 |  |
|  |  |  |  | 1.871 | 1.815 |  |  |  |  |  |  | 1.914 | 1.841 |  |
|  |  |  |  | 1.893 | 1.837 |  |  | **2AQY-9** |  | **Na^+^** |  | 1.893 | 1.883 |  |
|  |  |  |  | 1.904 | 1.84 |  |  |  |  |  |  | 1.843 | 1.971 |  |
|  |  |  |  | 2.001 | 1.849 |  |  |  |  |  |  | 1.911 | 1.844 |  |
|  |  |  |  | 1.825 | 2.03 |  |  |  |  |  |  | 1.87 | 1.827 |  |
| **2AQY-4** |  | **Na^+^** |  | 1.936 | 1.845 |  |  |  |  |  |  | 1.833 | 1.855 |  |
|  |  |  |  | 1.898 | 1.875 |  |  |  |  |  |  | 1.947 | 1.834 |  |
|  |  |  |  | 1.926 | 1.857 |  |  |  |  |  |  | 1.874 | 1.967 |  |
|  |  |  |  | 1.846 | 1.895 |  |  |  |  |  |  | 1.858 | 1.883 |  |
|  |  |  |  | 1.886 | 1.853 |  |  |  |  |  |  | 1.861 | 1.875 |  |
|  |  |  |  | 1.874 | 1.832 |  |  |  |  |  |  | 1.816 | 2.067 |  |
|  |  |  |  | 1.855 | 1.854 |  |  |  |  |  |  |  |  |  |
|  |  |  |  | 1.857 | 1.869 |  |  |  |  |  |  |  |  |  |
|  |  |  |  | 1.849 | 1.903 |  |  |  |  |  |  |  |  |  |
|  |  |  |  | 1.84 | 1.866 |  |  |  |  |  |  |  |  |  |

| **PDB ID** |  | **Metal ion** |  | **NH-N** | **NH-O** | **M^+^∙∙∙O** |  | **PDB ID** |  | **Metal ion** |  | **NH-N** | **NH-O** | **M^+^∙∙∙O** |
| --- | --- | --- | --- | --- | --- | --- | --- | --- | --- | --- | --- | --- | --- | --- |
| **2AQY-10** |  | **Na^+^** |  | 1.938 | 1.844 |  |  | **143D-4** |  | **Na+** |  | 2.01 | 1.811 |  |
|  |  |  |  | 1.872 | 1.858 |  |  |  |  |  |  | 1.819 | 1.903 |  |
|  |  |  |  | 1.939 | 1.844 |  |  |  |  |  |  | 1.953 | 1.812 |  |
|  |  |  |  | 1.861 | 1.972 |  |  |  |  |  |  | 1.898 | 1.885 |  |
|  |  |  |  | 1.845 | 1.858 |  |  |  |  |  |  | 1.945 | 1.772 |  |
|  |  |  |  | 1.875 | 1.862 |  |  |  |  |  |  | 1.923 | 1.834 |  |
|  |  |  |  | 1.861 | 1.877 |  |  |  |  |  |  | 1.808 | 1.865 |  |
|  |  |  |  | 1.861 | 1.852 |  |  |  |  |  |  | 1.797 | 1.857 |  |
|  |  |  |  | 1.867 | 1.88 |  |  |  |  |  |  | 1.838 | 1.882 |  |
|  |  |  |  | 1.883 | 1.87 |  |  |  |  |  |  | 1.896 | 1.891 |  |
| **143D-1** |  | **Na+** |  | 1.885 | 1.794 |  |  |  |  |  |  | 1.98 | 1.822 |  |
|  |  |  |  | 1.826 | 1.848 |  |  |  |  |  |  | 1.816 | 1.966 |  |
|  |  |  |  | 1.862 | 1.857 |  |  | **143D-5** |  | **Na+** |  | 1.964 | 1.797 |  |
|  |  |  |  | 1.895 | 1.821 |  |  |  |  |  |  | 1.95 | 1.872 |  |
|  |  |  |  | 1.91 | 1.794 |  |  |  |  |  |  | 1.934 | 1.809 |  |
|  |  |  |  | 1.828 | 1.865 |  |  |  |  |  |  | 1.867 | 1.867 |  |
|  |  |  |  | 1.88 | 1.789 |  |  |  |  |  |  | 1.989 | 1.798 |  |
|  |  |  |  | 1.978 | 1.796 |  |  |  |  |  |  | 1.895 | 1.893 |  |
|  |  |  |  | 2.034 | 1.761 |  |  |  |  |  |  | 1.857 | 1.857 |  |
|  |  |  |  | 1.939 | 1.899 |  |  |  |  |  |  | 1.838 | 1.838 |  |
|  |  |  |  | 1.835 | 1.842 |  |  |  |  |  |  | 1.805 | 1.841 |  |
|  |  |  |  | 1.876 | 1.915 |  |  |  |  |  |  | 1.965 | 1.773 |  |
| **143D-2** |  | **Na+** |  | 1.962 | 1.812 |  |  |  |  |  |  | 1.907 | 1.779 |  |
|  |  |  |  | 1.908 | 1.803 |  |  |  |  |  |  | 1.852 | 1.928 |  |
|  |  |  |  | 1.953 | 1.953 |  |  | **143D-6** |  | **Na+** |  | 1.838 | 1.844 |  |
|  |  |  |  | 1.883 | 1.883 |  |  |  |  |  |  | 1.778 | 1.847 |  |
|  |  |  |  | 1.951 | 1.831 |  |  |  |  |  |  | 1.877 | 1.818 |  |
|  |  |  |  | 1.824 | 1.874 |  |  |  |  |  |  | 1.851 | 1.818 |  |
|  |  |  |  | 1.961 | 1.779 |  |  |  |  |  |  | 1.844 | 1.798 |  |
|  |  |  |  | 1.873 | 1.84 |  |  |  |  |  |  | 1.935 | 1.778 |  |
|  |  |  |  | 1.925 | 1.816 |  |  |  |  |  |  | 1.879 | 1.773 |  |
|  |  |  |  | 1.929 | 1.837 |  |  |  |  |  |  | 1.854 | 1.865 |  |
|  |  |  |  | 1.865 | 1.89 |  |  |  |  |  |  | 1.844 | 1.768 |  |
|  |  |  |  | 1.857 | 1.866 |  |  |  |  |  |  | 1.873 | 1.835 |  |
| **143D-3** |  | **Na+** |  | 1.833 | 1.822 |  |  |  |  |  |  | 1.867 | 1.821 |  |
|  |  |  |  | 1.84 | 1.836 |  |  |  |  |  |  | 1.807 | 1.875 |  |
|  |  |  |  | 1.867 | 1.829 |  |  | **186D-1** |  | **Na^+^** |  | 1.963 | 1.917 |  |
|  |  |  |  | 1.875 | 1.818 |  |  |  |  |  |  | 1.932 | 1.96 |  |
|  |  |  |  | 1.833 | 1.785 |  |  |  |  |  |  | 1.965 | 1.921 |  |
|  |  |  |  | 1.890 | 1.808 |  |  |  |  |  |  | 1.942 | 1.984 |  |
|  |  |  |  | 1.872 | 1.802 |  |  |  |  |  |  | 1.938 | 1.939 |  |
|  |  |  |  | 1.862 | 1.834 |  |  |  |  |  |  | 1.927 | 1.996 |  |
|  |  |  |  | 2.071 | 1.806 |  |  |  |  |  |  | 1.952 | 1.94 |  |
|  |  |  |  | 1.845 | 1.863 |  |  |  |  |  |  | 1.949 | 1.935 |  |
|  |  |  |  | 1.914 | 1.801 |  |  |  |  |  |  | 1.921 | 1.958 |  |
|  |  |  |  | 1.839 | 1.917 |  |  |  |  |  |  | 1.959 | 1.937 |  |
|  |  |  |  |  |  |  |  |  |  |  |  | 1.906 | 1.958 |  |
|  |  |  |  |  |  |  |  |  |  |  |  | 1.927 | 1.955 |  |

| **PDB ID** |  | **Metal ion** |  | **NH-N** | **NH-O** | **M^+^∙∙∙O** |  | **PDB ID** |  | **Metal ion** |  | **NH-N** | **NH-O** | **M^+^∙∙∙O** |
| --- | --- | --- | --- | --- | --- | --- | --- | --- | --- | --- | --- | --- | --- | --- |
| **186D-2** |  | **Na^+^** |  | 1.943 | 1.953 |  |  | **186D-6** |  | **Na^+^** |  | 1.948 | 1.914 |  |
|  |  |  |  | 1.907 | 1.957 |  |  |  |  |  |  | 1.939 | 1.966 |  |
|  |  |  |  | 1.928 | 1.954 |  |  |  |  |  |  | 1.963 | 1.93 |  |
|  |  |  |  | 1.951 | 1.944 |  |  |  |  |  |  | 1.931 | 1.99 |  |
|  |  |  |  | 1.937 | 1.926 |  |  |  |  |  |  | 1.937 | 1.93 |  |
|  |  |  |  | 1.946 | 1.962 |  |  |  |  |  |  | 1.938 | 2 |  |
|  |  |  |  | 1.954 | 1.933 |  |  |  |  |  |  | 1.921 | 1.935 |  |
|  |  |  |  | 1.94 | 1.978 |  |  |  |  |  |  | 1.941 | 1.971 |  |
|  |  |  |  | 1.945 | 1.941 |  |  |  |  |  |  | 1.924 | 1.904 |  |
|  |  |  |  | 1.915 | 1.965 |  |  |  |  |  |  | 1.939 | 1.958 |  |
|  |  |  |  | 1.942 | 1.947 |  |  |  |  |  |  | 1.938 | 1.92 |  |
|  |  |  |  | 1.939 | 1.935 |  |  |  |  |  |  | 1.96 | 1.96 |  |
| **186D-3** |  | **Na^+^** |  | 1.935 | 1.936 |  |  | **186D-7** |  | **Na^+^** |  | 1.933 | 1.926 |  |
|  |  |  |  | 1.964 | 1.943 |  |  |  |  |  |  | 1.935 | 1.953 |  |
|  |  |  |  | 1.953 | 1.929 |  |  |  |  |  |  | 1.924 | 1.924 |  |
|  |  |  |  | 1.972 | 1.931 |  |  |  |  |  |  | 1.988 | 1.988 |  |
|  |  |  |  | 1.924 | 1.934 |  |  |  |  |  |  | 1.941 | 1.933 |  |
|  |  |  |  | 1.949 | 1.948 |  |  |  |  |  |  | 1.957 | 1.955 |  |
|  |  |  |  | 1.941 | 1.939 |  |  |  |  |  |  | 1.962 | 1.922 |  |
|  |  |  |  | 1.934 | 1.986 |  |  |  |  |  |  | 1.942 | 1.994 |  |
|  |  |  |  | 1.968 | 1.934 |  |  |  |  |  |  | 1.936 | 1.918 |  |
|  |  |  |  | 1.945 | 1.96 |  |  |  |  |  |  | 1.927 | 1.96 |  |
|  |  |  |  | 1.909 | 1.946 |  |  |  |  |  |  | 1.95 | 1.932 |  |
|  |  |  |  | 1.919 | 1.945 |  |  |  |  |  |  | 1.909 | 1.959 |  |
| **186D-4** |  | **Na^+^** |  | 1.91 | 1.96 |  |  | **201D-1** |  | **Na^+^** |  | 1.97 | 1.924 |  |
|  |  |  |  | 1.972 | 1.961 |  |  |  |  |  |  | 1.942 | 1.984 |  |
|  |  |  |  | 1.939 | 1.95 |  |  |  |  |  |  | 1.972 | 1.927 |  |
|  |  |  |  | 1.941 | 1.962 |  |  |  |  |  |  | 1.956 | 1.967 |  |
|  |  |  |  | 1.941 | 1.939 |  |  |  |  |  |  | 1.936 | 1.952 |  |
|  |  |  |  | 1.937 | 1.939 |  |  |  |  |  |  | 1.938 | 2.024 |  |
|  |  |  |  | 1.943 | 1.93 |  |  |  |  |  |  | 1.936 | 1.949 |  |
|  |  |  |  | 1.949 | 1.93 |  |  |  |  |  |  | 1.94 | 2 |  |
|  |  |  |  | 1.911 | 1.94 |  |  |  |  |  |  | 1.958 | 1.953 |  |
|  |  |  |  | 1.91 | 1.968 |  |  |  |  |  |  | 1.94 | 1.973 |  |
|  |  |  |  | 1.936 | 1.952 |  |  |  |  |  |  | 1.94 | 1.948 |  |
|  |  |  |  | 1.936 | 1.965 |  |  |  |  |  |  | 1.935 | 1.98 |  |
| **186D-5** |  | **Na^+^** |  | 1.957 | 1.919 |  |  |  |  |  |  | 1.972 | 1.929 |  |
|  |  |  |  | 1.94 | 1.949 |  |  |  |  |  |  | 1.945 | 1.989 |  |
|  |  |  |  | 1.958 | 1.928 |  |  |  |  |  |  | 1.967 | 1.926 |  |
|  |  |  |  | 1.935 | 1.966 |  |  |  |  |  |  | 1.958 | 1.975 |  |
|  |  |  |  | 1.943 | 1.925 |  |  | **201D-2** |  | **Na^+^** |  | 1.955 | 1.933 |  |
|  |  |  |  | 1.942 | 2.014 |  |  |  |  |  |  | 1.958 | 1.978 |  |
|  |  |  |  | 1.95 | 1.931 |  |  |  |  |  |  | 1.953 | 1.931 |  |
|  |  |  |  | 1.952 | 1.99 |  |  |  |  |  |  | 1.936 | 1.981 |  |
|  |  |  |  | 1.943 | 1.925 |  |  |  |  |  |  | 1.93 | 1.932 |  |
|  |  |  |  | 1.916 | 1.954 |  |  |  |  |  |  | 1.937 | 2.05 |  |
|  |  |  |  | 1.992 | 1.924 |  |  |  |  |  |  | 1.931 | 1.936 |  |
|  |  |  |  | 1.934 | 1.983 |  |  |  |  |  |  | 1.935 | 2.015 |  |
|  |  |  |  |  |  |  |  |  |  |  |  | 1.949 | 1.952 |  |
|  |  |  |  |  |  |  |  |  |  |  |  | 1.942 | 1.992 |  |
|  |  |  |  |  |  |  |  |  |  |  |  | 1.925 | 1.966 |  |
|  |  |  |  |  |  |  |  |  |  |  |  | 1.93 | 2.033 |  |
|  |  |  |  |  |  |  |  |  |  |  |  | 1.956 | 1.92 |  |
|  |  |  |  |  |  |  |  |  |  |  |  | 1.944 | 1.975 |  |
|  |  |  |  |  |  |  |  |  |  |  |  | 1.975 | 1.924 |  |
|  |  |  |  |  |  |  |  |  |  |  |  | 1.942 | 1.993 |  |

| **PDB ID** |  | **Metal ion** |  | **NH-N** | **NH-O** | **M^+^∙∙∙O** |  | **PDB ID** |  | **Metal ion** |  | **NH-N** | **NH-O** | **M^+^∙∙∙O** |
| --- | --- | --- | --- | --- | --- | --- | --- | --- | --- | --- | --- | --- | --- | --- |
| **201D-3** |  | **Na^+^** |  | 1.976 | 1.917 |  |  | **201D-6** |  | **Na^+^** |  | 1.97 | 1.933 |  |
|  |  |  |  | 1.94 | 1.992 |  |  |  |  |  |  | 1.954 | 1.966 |  |
|  |  |  |  | 1.973 | 1.93 |  |  |  |  |  |  | 1.966 | 1.938 |  |
|  |  |  |  | 1.947 | 1.999 |  |  |  |  |  |  | 1.955 | 1.991 |  |
|  |  |  |  | 1.941 | 1.938 |  |  |  |  |  |  | 1.943 | 1.954 |  |
|  |  |  |  | 1.94 | 2.012 |  |  |  |  |  |  | 1.945 | 1.999 |  |
|  |  |  |  | 1.938 | 1.932 |  |  |  |  |  |  | 1.933 | 1.934 |  |
|  |  |  |  | 1.936 | 2.035 |  |  |  |  |  |  | 1.932 | 2.01 |  |
|  |  |  |  | 1.923 | 1.994 |  |  |  |  |  |  | 1.939 | 1.939 |  |
|  |  |  |  | 1.927 | 2.015 |  |  |  |  |  |  | 1.938 | 1.984 |  |
|  |  |  |  | 1.956 | 1.983 |  |  |  |  |  |  | 1.935 | 1.952 |  |
|  |  |  |  | 1.942 | 1.979 |  |  |  |  |  |  | 1.96 | 1.975 |  |
|  |  |  |  | 1.944 | 1.923 |  |  |  |  |  |  | 1.954 | 1.928 |  |
|  |  |  |  | 1.943 | 1.948 |  |  |  |  |  |  | 1.965 | 1.977 |  |
|  |  |  |  | 1.953 | 1.934 |  |  |  |  |  |  | 1.983 | 1.928 |  |
|  |  |  |  | 1.939 | 1.97 |  |  |  |  |  |  | 1.95 | 1.981 |  |
| **201D-4** |  | **Na^+^** |  | 1.957 | 1.931 |  |  | **230D-1** |  | **Na^+^** |  | 1.912 | 1.992 |  |
|  |  |  |  | 1.952 | 2.012 |  |  |  |  |  |  | 1.933 | 1.993 |  |
|  |  |  |  | 1.975 | 1.911 |  |  |  |  |  |  | 1.877 | 2.004 |  |
|  |  |  |  | 1.962 | 1.999 |  |  |  |  |  |  | 1.969 | 2.014 |  |
|  |  |  |  | 1.948 | 1.93 |  |  |  |  |  |  | 1.856 | 1.989 |  |
|  |  |  |  | 1.965 | 1.968 |  |  |  |  |  |  | 1.903 | 1.991 |  |
|  |  |  |  | 1.945 | 1.932 |  |  |  |  |  |  | 1.888 | 1.969 |  |
|  |  |  |  | 1.932 | 1.99 |  |  |  |  |  |  | 1.922 | 1.975 |  |
|  |  |  |  | 1.96 | 1.993 |  |  |  |  |  |  | 1.919 | 2.008 |  |
|  |  |  |  | 1.948 | 1.992 |  |  |  |  |  |  | 1.93 | 1.963 |  |
|  |  |  |  | 1.924 | 1.987 |  |  |  |  |  |  | 1.936 | 1.988 |  |
|  |  |  |  | 1.93 | 2.019 |  |  |  |  |  |  | 1.893 | 1.98 |  |
|  |  |  |  | 1.939 | 1.924 |  |  | **230D-2** |  | **Na^+^** |  | 1.969 | 2.000 |  |
|  |  |  |  | 1.936 | 1.961 |  |  |  |  |  |  | 1.884 | 1.997 |  |
|  |  |  |  | 1.957 | 1.922 |  |  |  |  |  |  | 1.858 | 1.977 |  |
|  |  |  |  | 1.937 | 1.98 |  |  |  |  |  |  | 1.999 | 1.981 |  |
| **201D-5** |  | **Na^+^** |  | 1.967 | 1.923 |  |  |  |  |  |  | 1.885 | 1.992 |  |
|  |  |  |  | 1.947 | 1.985 |  |  |  |  |  |  | 1.794 | 1.999 |  |
|  |  |  |  | 1.935 | 1.92 |  |  |  |  |  |  | 1.933 | 1.93 |  |
|  |  |  |  | 1.942 | 1.968 |  |  |  |  |  |  | 1.917 | 1.984 |  |
|  |  |  |  | 1.93 | 2.01 |  |  |  |  |  |  | 1.929 | 1.988 |  |
|  |  |  |  | 1.924 | 1.994 |  |  |  |  |  |  | 1.937 | 1.982 |  |
|  |  |  |  | 1.959 | 1.962 |  |  |  |  |  |  | 1.959 | 2.018 |  |
|  |  |  |  | 1.949 | 1.977 |  |  |  |  |  |  | 1.869 | 1.868 |  |
|  |  |  |  | 1.943 | 1.96 |  |  | **230D-3** |  | **Na^+^** |  | 1.921 | 1.975 |  |
|  |  |  |  | 1.92 | 1.985 |  |  |  |  |  |  | 1.934 | 1.968 |  |
|  |  |  |  | 1.925 | 1.966 |  |  |  |  |  |  | 1.787 | 2.003 |  |
|  |  |  |  | 1.935 | 1.993 |  |  |  |  |  |  | 1.959 | 1.991 |  |
|  |  |  |  | 1.967 | 1.92 |  |  |  |  |  |  | 1.894 | 1.968 |  |
|  |  |  |  | 1.955 | 1.995 |  |  |  |  |  |  | 1.91 | 2.002 |  |
|  |  |  |  | 1.949 | 1.936 |  |  |  |  |  |  | 1.929 | 1.913 |  |
|  |  |  |  | 1.951 | 1.967 |  |  |  |  |  |  | 1.878 | 2.001 |  |
|  |  |  |  |  |  |  |  |  |  |  |  | 1.98 | 1.947 |  |
|  |  |  |  |  |  |  |  |  |  |  |  | 1.913 | 1.987 |  |
|  |  |  |  |  |  |  |  |  |  |  |  | 1.928 | 2.001 |  |
|  |  |  |  |  |  |  |  |  |  |  |  | 1.813 | 1.973 |  |

| **PDB ID** |  | **Metal ion** |  | **NH-N** | **NH-O** | **M^+^∙∙∙O** |  | **PDB ID** |  | **Metal ion** |  | **NH-N** | **NH-O** | **M^+^∙∙∙O** |
| --- | --- | --- | --- | --- | --- | --- | --- | --- | --- | --- | --- | --- | --- | --- |
| **230D-4** |  | **Na^+^** |  | 1.944 | 2.013 |  |  | **352D-1** |  | **Na^+^** |  | 1.837 | 1.871 | 2.306 |
|  |  |  |  | 1.906 | 1.918 |  |  |  |  |  |  | 1.873 | 1.879 | 2.288 |
|  |  |  |  | 1.959 | 2.014 |  |  |  |  |  |  | 1.796 | 1.884 | 2.304 |
|  |  |  |  | 1.776 | 1.981 |  |  |  |  |  |  | 1.858 | 1.882 | 2.324 |
|  |  |  |  | 1.793 | 1.98 |  |  |  |  |  |  | 1.905 | 1.841 |  |
|  |  |  |  | 1.94 | 1.954 |  |  |  |  |  |  | 1.775 | 1.864 |  |
|  |  |  |  | 1.902 | 1.97 |  |  |  |  |  |  | 1.804 | 1.879 |  |
|  |  |  |  | 1.929 | 1.962 |  |  |  |  |  |  | 1.788 | 1.849 |  |
|  |  |  |  | 1.952 | 1.991 |  |  |  |  |  |  | 1.842 | 1.848 | 2.554 |
|  |  |  |  | 1.946 | 1.975 |  |  |  |  |  |  | 1.863 | 1.901 | 2.676 |
|  |  |  |  | 1.77 | 2.031 |  |  |  |  |  |  | 1.867 | 1.879 | 2.574 |
|  |  |  |  | 1.932 | 1.932 |  |  |  |  |  |  | 1.798 | 1.93 | 2.93 |
| **230D-5** |  | **Na^+^** |  | 1.944 | 1.959 |  |  |  |  |  |  | 1.807 | 1.881 | 2.896 |
|  |  |  |  | 1.915 | 1.977 |  |  |  |  |  |  | 1.807 | 1.866 | 2.997 |
|  |  |  |  | 1.863 | 2.001 |  |  |  |  |  |  | 1.834 | 1.846 | 2.914 |
|  |  |  |  | 1.917 | 2.004 |  |  |  |  |  |  | 1.8 | 1.85 | 2.779 |
|  |  |  |  | 1.916 | 1.866 |  |  |  |  |  |  | 1.775 | 1.81 | 2.694 |
|  |  |  |  | 1.819 | 2.035 |  |  |  |  |  |  | 1.821 | 1.824 | 2.749 |
|  |  |  |  | 1.913 | 1.931 |  |  |  |  |  |  | 1.783 | 1.804 | 2.757 |
|  |  |  |  | 1.903 | 2.043 |  |  |  |  |  |  | 1.795 | 1.829 | 2.696 |
|  |  |  |  | 1.935 | 2.023 |  |  |  |  |  |  | 1.821 | 1.873 | 2.524 |
|  |  |  |  | 1.909 | 1.951 |  |  |  |  |  |  | 1.797 | 1.876 | 2.518 |
|  |  |  |  | 1.781 | 2.007 |  |  |  |  |  |  | 1.826 | 1.872 | 2.526 |
|  |  |  |  | 1.965 | 1.851 |  |  |  |  |  |  | 1.82 | 1.887 | 2.511 |
| **230D-6** |  | **Na^+^** |  | 1.86 | 1.928 |  |  |  |  |  |  | 1.829 | 1.831 |  |
|  |  |  |  | 1.923 | 2.019 |  |  |  |  |  |  | 1.814 | 1.923 |  |
|  |  |  |  | 1.966 | 1.951 |  |  |  |  |  |  | 1.816 | 1.89 |  |
|  |  |  |  | 1.897 | 2.029 |  |  |  |  |  |  | 1.834 | 1.907 |  |
|  |  |  |  | 1.934 | 1.924 |  |  |  |  |  |  | 1.794 | 1.879 | 2.301 |
|  |  |  |  | 1.894 | 2.006 |  |  |  |  |  |  | 1.894 | 1.88 | 2.302 |
|  |  |  |  | 1.886 | 1.964 |  |  |  |  |  |  | 1.847 | 1.905 | 2.306 |
|  |  |  |  | 1.809 | 2.011 |  |  |  |  |  |  | 1.875 | 1.899 | 2.284 |
|  |  |  |  | 1.838 | 2.027 |  |  | **352D-2** |  | **Na^+^** |  | 1.794 | 1.879 | 2.301 |
|  |  |  |  | 1.968 | 1.978 |  |  |  |  |  |  | 1.894 | 1.88 | 2.302 |
|  |  |  |  | 1.882 | 1.998 |  |  |  |  |  |  | 1.847 | 1.905 | 2.306 |
|  |  |  |  | 1.928 | 1.923 |  |  |  |  |  |  | 1.875 | 1.899 | 2.284 |
| **230D-7** |  | **Na^+^** |  | 1.917 | 2 |  |  |  |  |  |  | 1.829 | 1.831 |  |
|  |  |  |  | 1.854 | 1.972 |  |  |  |  |  |  | 1.814 | 1.923 |  |
|  |  |  |  | 1.846 | 1.994 |  |  |  |  |  |  | 1.816 | 1.89 |  |
|  |  |  |  | 1.968 | 1.972 |  |  |  |  |  |  | 1.834 | 1.907 |  |
|  |  |  |  | 1.839 | 1.842 |  |  |  |  |  |  | 1.826 | 1.872 | 2.526 |
|  |  |  |  | 1.881 | 2.044 |  |  |  |  |  |  | 1.82 | 1.887 | 2.511 |
|  |  |  |  | 1.935 | 1.901 |  |  |  |  |  |  | 1.821 | 1.873 | 2.524 |
|  |  |  |  | 1.955 | 2.046 |  |  |  |  |  |  | 1.797 | 1.876 | 2.518 |
|  |  |  |  | 1.839 | 1.999 |  |  |  |  |  |  | 1.775 | 1.81 | 2.828 |
|  |  |  |  | 1.965 | 1.941 |  |  |  |  |  |  | 1.821 | 1.824 | 2.867 |
|  |  |  |  | 1.832 | 1.923 |  |  |  |  |  |  | 1.783 | 1.804 | 2.724 |
|  |  |  |  | 1.94 | 1.94 |  |  |  |  |  |  | 1.795 | 1.829 | 2.732 |
| **4DIH** |  | **Na+** |  | 3.131 | 2.937 |  |  |  |  |  |  | 1.807 | 1.881 | 2.665 |
|  |  |  |  | 2.661 | 2.712 |  |  |  |  |  |  | 1.807 | 1.866 | 2.733 |
|  |  |  |  | 2.869 | 2.887 |  |  |  |  |  |  | 1.834 | 1.846 | 2.679 |
|  |  |  |  | 2.892 | 2.852 |  |  |  |  |  |  | 1.8 | 1.85 | 2.652 |
|  |  |  |  | 2.871 | 2.903 |  |  |  |  |  |  | 1.867 | 1.879 | 2.574 |
|  |  |  |  | 3.07 | 2.698 |  |  |  |  |  |  | 1.798 | 1.93 | 2.605 |
|  |  |  |  | 2.793 | 2.751 |  |  |  |  |  |  | 1.842 | 1.848 | 2.554 |
|  |  |  |  | 2.745 | 2.749 |  |  |  |  |  |  | 1.863 | 1.901 | 2.676 |
|  |  |  |  |  |  |  |  |  |  |  |  | 1.905 | 1.841 |  |
|  |  |  |  |  |  |  |  |  |  |  |  | 1.775 | 1.864 |  |
|  |  |  |  |  |  |  |  |  |  |  |  | 1.804 | 1.879 |  |
|  |  |  |  |  |  |  |  |  |  |  |  | 1.788 | 1.849 |  |
|  |  |  |  |  |  |  |  |  |  |  |  | 1.837 | 1.871 | 2.306 |
|  |  |  |  |  |  |  |  |  |  |  |  | 1.873 | 1.879 | 2.288 |
|  |  |  |  |  |  |  |  |  |  |  |  | 1.796 | 1.884 | 2.304 |
|  |  |  |  |  |  |  |  |  |  |  |  | 1.858 | 1.882 | 2.324 |

| **PDB ID** | |  | | **Metal ion** |  | | **NH-N** | | **NH-O** | | **M^+^∙∙∙O** | |  | | | **PDB ID** | |  | | **Metal ion** |  | | **NH-N** | | **NH-O** | | **M^+^∙∙∙O** | |
| --- | --- | --- | --- | --- | --- | --- | --- | --- | --- | --- | --- | --- | --- | --- | --- | --- | --- | --- | --- | --- | --- | --- | --- | --- | --- | --- | --- | --- |
| 1jpq | |  | | K^+^ |  | | 2.01 | | 1.909 | | 3.001 | |  | | | 2gku-1 | |  | | K^+^ |  | | 2.031 | | 1.924 | |  | |
|  |  |  | |  |  | | 1.88 | | 1.876 | | 2.873 | |  | | |  |  |  | |  |  | | 1.828 | | 2.012 | |  | |
|  |  |  | |  |  | | 1.70 | | 1.849 | | 2.877 | |  | | |  |  |  | |  |  | | 1.933 | | 1.798 | |  | |
|  |  |  | |  |  | | 1.86 | | 1.799 | | 3.08 | |  | | |  |  |  | |  |  | | 2.016 | | 2.004 | |  | |
|  |  |  | |  |  | | 1.89 | | 2.014 | | 2.886 | |  | | |  |  |  | |  |  | | 1.84 | | 1.997 | |  | |
|  |  |  | |  |  | | 1.83 | | 1.839 | | 2.865 | |  | | |  |  |  | |  |  | | 2.008 | | 1.842 | |  | |
|  |  |  | |  |  | | 1.81 | | 1.846 | | 2.669 | |  | | |  |  |  | |  |  | | 2.003 | | 1.934 | |  | |
|  |  |  | |  |  | | 1.90 | | 1.822 | | 2.986 | |  | | |  |  |  | |  |  | | 1.847 | | 2.046 | |  | |
|  |  |  | |  |  | | 1.88 | | 1.904 | | 2.85 | |  | | |  |  |  | |  |  | | 1.79 | | 2.024 | |  | |
|  |  |  | |  |  | | 1.99 | | 1.87 | | 2.782 | |  | | |  |  |  | |  |  | | 1.91 | | 2.04 | |  | |
|  |  |  | |  |  | | 1.92 | | 1.857 | | 2.728 | |  | | |  |  |  | |  |  | | 1.88 | | 2.011 | |  | |
|  |  |  | |  |  | | 1.96 | | 1.96 | | 2.912 | |  | | |  |  |  | |  |  | | 1.925 | | 2.007 | |  | |
|  |  |  | |  |  | | 1.88 | | 1.849 | | 2.823 | |  | | | 2gku-2 | |  | | K^+^ |  | | 1.869 | | 2.013 | |  | |
|  |  |  | |  |  | | 1.86 | | 1.814 | | 2.753 | |  | | |  |  |  | |  |  | | 1.796 | | 2.055 | |  | |
|  |  |  | |  |  | | 1.85 | | 1.862 | | 2.74 | |  | | |  |  |  | |  |  | | 1.861 | | 1.92 | |  | |
|  |  |  | |  |  | | 1.84 | | 1.849 | | 2.742 | |  | | |  |  |  | |  |  | | 2.004 | | 2.014 | |  | |
| 1jrn-1 | |  | | K^+^ |  | | 1.9 | | 1.977 | | 2.704 | |  | | |  |  |  | |  |  | | 2.025 | | 1.899 | |  | |
|  |  |  | |  |  | | 2.0 | | 1.987 | | 2.752 | |  | | |  |  |  | |  |  | | 1.937 | | 2.015 | |  | |
|  |  |  | |  |  | | 2.2 | | 2.082 | | 2.944 | |  | | |  |  |  | |  |  | | 1.795 | | 2.025 | |  | |
|  |  |  | |  |  | | 1.9 | | 1.925 | | 2.955 | |  | | |  |  |  | |  |  | | 2 | | 2.01 | |  | |
|  |  |  | |  |  | | 2.0 | | 2.011 | | 2.589 | |  | | |  |  |  | |  |  | | 1.843 | | 2.071 | |  | |
|  |  |  | |  |  | | 1.7 | | 1.708 | | 2.647 | |  | | |  |  |  | |  |  | | 1.827 | | 2.022 | |  | |
|  |  |  | |  |  | | 1.8 | | 1.734 | | 2.871 | |  | | |  |  |  | |  |  | | 1.804 | | 2.026 | |  | |
|  |  |  | |  |  | | 1.8 | | 1.836 | | 2.729 | |  | | |  |  |  | |  |  | | 1.832 | | 2.027 | |  | |
|  |  |  | |  |  | | 2.0 | | 1.833 | | 2.869 | |  | | | 2gku-3 | |  | | K^+^ |  | | 1.93 | | 2.006 | |  | |
|  |  |  | |  |  | | 1.7 | | 1.712 | | 2.528 | |  | | |  |  |  | |  |  | | 1.869 | | 2.082 | |  | |
|  |  |  | |  |  | | 1.7 | | 1.893 | | 2.904 | |  | | |  |  |  | |  |  | | 1.783 | | 2.016 | |  | |
|  |  |  | |  |  | | 2.1 | | 1.936 | | 2.961 | |  | | |  |  |  | |  |  | | 1.87 | | 2.005 | |  | |
|  |  |  | |  |  | | 1.9 | | 1.957 | | 2.821 | |  | | |  |  |  | |  |  | | 1.8 | | 2.014 | |  | |
|  |  |  | |  |  | | 1.9 | | 1.93 | | 2.669 | |  | | |  |  |  | |  |  | | 1.998 | | 2.013 | |  | |
|  |  |  | |  |  | | 1.7 | | 2.008 | | 3.069 | |  | | |  |  |  | |  |  | | 2.021 | | 1.933 | |  | |
|  |  |  | |  |  | | 1.9 | | 1.796 | | 2.98 | |  | | |  |  |  | |  |  | | 1.886 | | 2.02 | |  | |
| 1jrn-2 | |  | | K^+^ |  | | 1.70 | | 1.686 | | 2.985 | |  | | | 2gku-4 | |  | | K^+^ |  | | 1.847 | | 2.01 | |  | |
|  |  |  | |  |  | | 1.75 | | 1.585 | | 2.959 | |  | | |  |  |  | |  |  | | 1.797 | | 2.063 | |  | |
|  |  |  | |  |  | | 1.79 | | 1.909 | | 2.627 | |  | | |  |  |  | |  |  | | 1.819 | | 1.939 | |  | |
|  |  |  | |  |  | | 1.80 | | 1.834 | | 2.57 | |  | | |  |  |  | |  |  | | 2 | | 2.021 | |  | |
|  |  |  | |  |  | | 1.70 | | 1.696 | | 2.636 | |  | | |  |  |  | |  |  | | 1.799 | | 2.017 | |  | |
|  |  |  | |  |  | | 2.00 | | 2.034 | | 2.958 | |  | | |  |  |  | |  |  | | 1.96 | | 2.013 | |  | |
|  |  |  | |  |  | | 1.96 | | 1.848 | | 2.6 | |  | | |  |  |  | |  |  | | 2.024 | | 1.925 | |  | |
|  |  |  | |  |  | | 1.87 | | 1.965 | | 2.89 | |  | | |  |  |  | |  |  | | 1.947 | | 2.033 | |  | |
|  |  |  | |  |  | | 1.98 | | 2.062 | | 2.986 | |  | | |  |  |  | |  |  | | 1.98 | | 2.014 | |  | |
|  |  |  | |  |  | | 1.92 | | 1.805 | | 2.966 | |  | | |  |  |  | |  |  | | 1.932 | | 2.061 | |  | |
|  |  |  | |  |  | | 1.85 | | 1.983 | | 2.965 | |  | | |  |  |  | |  |  | | 1.798 | | 1.987 | |  | |
|  |  |  | |  |  | | 2.05 | | 2.073 | | 2.688 | |  | | |  |  |  | |  |  | | 1.934 | | 2.014 | |  | |
|  |  |  | |  |  | | 1.94 | | 1.784 | | 2.733 | |  | | | 2gku-5 | |  | | K^+^ |  | | 1.853 | | 2.044 | |  | |
|  |  |  | |  |  | | 2.02 | | 1.856 | | 3.024 | |  | | |  |  |  | |  |  | | 1.869 | | 1.895 | |  | |
|  |  |  | |  |  | | 2.14 | | 2.096 | | 3.026 | |  | | |  |  |  | |  |  | | 1.893 | | 2.025 | |  | |
|  |  |  | |  |  | | 1.69 | | 1.76 | | 2.843 | |  | | |  |  |  | |  |  | | 1.824 | | 2.016 | |  | |
| 1k8p | |  | | K^+^ |  | | 1.94 | | 1.972 | | 2.801 | |  | | |  |  |  | |  |  | | 1.817 | | 2.015 | |  | |
|  |  |  | |  |  | | 1.75 | | 1.664 | | 2.716 | |  | | |  |  |  | |  |  | | 1.953 | | 2.017 | |  | |
|  |  |  | |  |  | | 1.76 | | 1.737 | | 2.532 | |  | | |  |  |  | |  |  | | 2.019 | | 1.904 | |  | |
|  |  |  | |  |  | | 1.69 | | 1.735 | | 2.439 | |  | | |  |  |  | |  |  | | 1.87 | | 2.009 | |  | |
|  |  |  | |  |  | | 1.78 | | 1.793 | | 2.606 | |  | | |  |  |  | |  |  | | 1.914 | | 1.989 | |  | |
|  |  |  | |  |  | | 1.70 | | 1.628 | | 2.854 | |  | | |  |  |  | |  |  | | 1.87 | | 2.032 | |  | |
|  |  |  | |  |  | | 1.70 | | 1.918 | | 2.965 | |  | | |  |  |  | |  |  | | 1.877 | | 2.008 | |  | |
|  |  |  | |  |  | | 1.74 | | 1.666 | | 2.798 | |  | | |  |  |  | |  |  | | 1.926 | | 2.018 | |  | |
|  |  |  | |  |  | | 1.94 | | 2.055 | | 2.871 | |  | | |  | |  | |  |  | |  | |  | |  | |
|  |  |  | |  |  | | 1.85 | | 1.743 | | 2.721 | |  | | |  | |  | |  |  | |  | |  | |  | |
|  |  |  | |  |  | | 2.10 | | 1.903 | | 2.861 | |  | | |  | |  | |  |  | |  | |  | |  | |
|  |  |  | |  |  | | 1.86 | | 1.845 | | 2.813 | |  | | |  | |  | |  |  | |  | |  | |  | |
| 1kf1 | |  | | K^+^ |  | | 1.80 | | 1.819 | | 2.814 | |  | | |  | |  | |  |  | |  | |  | |  | |
|  |  |  | |  |  | | 1.90 | | 1.88 | | 2.828 | |  | | |  | |  | |  |  | |  | |  | |  | |
|  |  |  | |  |  | | 1.84 | | 1.769 | | 2.62 | |  | | |  | |  | |  |  | |  | |  | |  | |
|  |  |  | |  |  | | 1.85 | | 1.957 | | 2.803 | |  | | |  | |  | |  |  | |  | |  | |  | |
|  |  |  | |  |  | | 1.76 | | 1.951 | | 2.684 | |  | | |  | |  | |  |  | |  | |  | |  | |
|  |  |  | |  |  | | 1.85 | | 1.843 | | 2.657 | |  | | |  | |  | |  |  | |  | |  | |  | |
|  |  |  | |  |  | | 1.70 | | 1.813 | | 2.787 | |  | | |  | |  | |  |  | |  | |  | |  | |
|  |  |  | |  |  | | 1.87 | | 1.77 | | 2.735 | |  | | |  | |  | |  |  | |  | |  | |  | |
|  |  |  | |  |  | | 1.94 | | 1.927 | | 2.594 | |  | | |  | |  | |  |  | |  | |  | |  | |
|  |  |  | |  |  | | 1.80 | | 1.864 | | 2.629 | |  | | |  | |  | |  |  | |  | |  | |  | |
|  |  |  | |  |  | | 1.87 | | 1.838 | | 2.612 | |  | | |  | |  | |  |  | |  | |  | |  | |
|  |  |  | |  |  | | 1.70 | | 1.876 | | 2.434 | |  | | |  | |  | |  |  | |  | |  | |  | |
| **PDB ID** |  | | **Metal ion** | | |  | | **NH-N** | | **NH-O** | | **M^+^∙∙∙O** | |  | **PDB ID** | |  | | **Metal ion** | | |  | | **NH-N** | | **NH-O** | | **M^+^∙∙∙O** |
| 2jpz-1 |  | | K^+^ | | |  | | 1.707 | | 1.789 | |  | |  | 2jpz-6 | |  | | K^+^ | | |  | | 1.684 | | 1.802 | |  |
|  |  | |  |  |  |  | | 1.741 | | 2.035 | |  | |  |  |  |  | |  |  |  |  | | 1.677 | | 2.048 | |  |
|  |  | |  |  |  |  | | 1.957 | | 1.842 | |  | |  |  |  |  | |  |  |  |  | | 1.968 | | 1.791 | |  |
|  |  | |  |  |  |  | | 1.761 | | 2.048 | |  | |  |  |  |  | |  |  |  |  | | 1.862 | | 2.044 | |  |
|  |  | |  |  |  |  | | 1.701 | | 1.919 | |  | |  |  |  |  | |  |  |  |  | | 1.728 | | 1.859 | |  |
|  |  | |  |  |  |  | | 1.708 | | 1.979 | |  | |  |  |  |  | |  |  |  |  | | 1.709 | | 1.965 | |  |
|  |  | |  |  |  |  | | 1.936 | | 1.999 | |  | |  |  |  |  | |  |  |  |  | | 1.792 | | 1.794 | |  |
|  |  | |  |  |  |  | | 1.718 | | 1.99 | |  | |  |  |  |  | |  |  |  |  | | 1.684 | | 2.004 | |  |
|  |  | |  |  |  |  | | 1.806 | | 2.017 | |  | |  |  |  |  | |  |  |  |  | | 1.977 | | 1.784 | |  |
|  |  | |  |  |  |  | | 1.943 | | 2.017 | |  | |  |  |  |  | |  |  |  |  | | 1.715 | | 1.899 | |  |
|  |  | |  |  |  |  | | 1.99 | | 1.819 | |  | |  |  |  |  | |  |  |  |  | | 2.004 | | 1.98 | |  |
|  |  | |  |  |  |  | | 1.755 | | 1.907 | |  | |  |  |  |  | |  |  |  |  | | 1.993 | | 1.793 | |  |
| 2jpz-2 |  | | K^+^ | | |  | | 1.994 | | 1.792 | |  | |  | 2jpz-7 | |  | | K^+^ | | |  | | 1.742 | | 1.897 | |  |
|  |  | |  |  |  |  | | 1.794 | | 2.084 | |  | |  |  |  |  | |  |  |  |  | | 1.901 | | 1.834 | |  |
|  |  | |  |  |  |  | | 1.676 | | 1.834 | |  | |  |  |  |  | |  |  |  |  | | 1.954 | | 1.791 | |  |
|  |  | |  |  |  |  | | 1.733 | | 1.809 | |  | |  |  |  |  | |  |  |  |  | | 1.699 | | 2.081 | |  |
|  |  | |  |  |  |  | | 1.865 | | 1.836 | |  | |  |  |  |  | |  |  |  |  | | 1.733 | | 1.94 | |  |
|  |  | |  |  |  |  | | 1.714 | | 1.983 | |  | |  |  |  |  | |  |  |  |  | | 1.919 | | 1.955 | |  |
|  |  | |  |  |  |  | | 1.893 | | 1.972 | |  | |  |  |  |  | |  |  |  |  | | 1.868 | | 2.008 | |  |
|  |  | |  |  |  |  | | 1.717 | | 1.966 | |  | |  |  |  |  | |  |  |  |  | | 1.812 | | 1.984 | |  |
|  |  | |  |  |  |  | | 1.998 | | 1.796 | |  | |  |  |  |  | |  |  |  |  | | 1.941 | | 1.796 | |  |
|  |  | |  |  |  |  | | 1.809 | | 2.015 | |  | |  |  |  |  | |  |  |  |  | | 1.751 | | 1.822 | |  |
|  |  | |  |  |  |  | | 1.984 | | 1.977 | |  | |  |  |  |  | |  |  |  |  | | 1.866 | | 2.018 | |  |
|  |  | |  |  |  |  | | 1.994 | | 1.799 | |  | |  |  |  |  | |  |  |  |  | | 1.992 | | 1.808 | |  |
| 2jpz-3 |  | | K^+^ | | |  | | 1.959 | | 1.783 | |  | |  | 2jpz-8 | |  | | K^+^ | | |  | | 1.953 | | 1.873 | |  |
|  |  | |  |  |  |  | | 1.788 | | 2.052 | |  | |  |  |  |  | |  |  |  |  | | 1.997 | | 1.788 | |  |
|  |  | |  |  |  |  | | 1.695 | | 1.803 | |  | |  |  |  |  | |  |  |  |  | | 1.915 | | 2.003 | |  |
|  |  | |  |  |  |  | | 1.694 | | 2.051 | |  | |  |  |  |  | |  |  |  |  | | 1.711 | | 1.952 | |  |
|  |  | |  |  |  |  | | 1.728 | | 2.011 | |  | |  |  |  |  | |  |  |  |  | | 1.706 | | 1.835 | |  |
|  |  | |  |  |  |  | | 1.748 | | 1.963 | |  | |  |  |  |  | |  |  |  |  | | 1.711 | | 1.993 | |  |
|  |  | |  |  |  |  | | 1.683 | | 2.028 | |  | |  |  |  |  | |  |  |  |  | | 1.816 | | 1.904 | |  |
|  |  | |  |  |  |  | | 1.692 | | 1.991 | |  | |  |  |  |  | |  |  |  |  | | 1.719 | | 1.985 | |  |
|  |  | |  |  |  |  | | 1.958 | | 1.789 | |  | |  |  |  |  | |  |  |  |  | | 1.81 | | 1.801 | |  |
|  |  | |  |  |  |  | | 1.717 | | 1.856 | |  | |  |  |  |  | |  |  |  |  | | 1.89 | | 2.037 | |  |
|  |  | |  |  |  |  | | 1.988 | | 2.002 | |  | |  |  |  |  | |  |  |  |  | | 1.994 | | 2.01 | |  |
|  |  | |  |  |  |  | | 1.969 | | 1.79 | |  | |  |  |  |  | |  |  |  |  | | 1.737 | | 1.849 | |  |
| 2jpz-4 |  | | K^+^ | | |  | | 1.963 | | 1.832 | |  | |  | 2jpz-9 | |  | | K^+^ | | |  | | 1.659 | | 1.804 | |  |
|  |  | |  |  |  |  | | 1.896 | | 2.034 | |  | |  |  |  |  | |  |  |  |  | | 1.724 | | 2.04 | |  |
|  |  | |  |  |  |  | | 1.671 | | 1.927 | |  | |  |  |  |  | |  |  |  |  | | 1.975 | | 1.785 | |  |
|  |  | |  |  |  |  | | 1.687 | | 2.044 | |  | |  |  |  |  | |  |  |  |  | | 1.815 | | 2.044 | |  |
|  |  | |  |  |  |  | | 1.977 | | 1.826 | |  | |  |  |  |  | |  |  |  |  | | 1.973 | | 1.993 | |  |
|  |  | |  |  |  |  | | 1.678 | | 2.008 | |  | |  |  |  |  | |  |  |  |  | | 1.923 | | 1.974 | |  |
|  |  | |  |  |  |  | | 1.926 | | 2.003 | |  | |  |  |  |  | |  |  |  |  | | 1.724 | | 1.985 | |  |
|  |  | |  |  |  |  | | 1.698 | | 1.944 | |  | |  |  |  |  | |  |  |  |  | | 1.694 | | 1.967 | |  |
|  |  | |  |  |  |  | | 1.724 | | 1.971 | |  | |  |  |  |  | |  |  |  |  | | 1.755 | | 1.831 | |  |
|  |  | |  |  |  |  | | 1.846 | | 2.034 | |  | |  |  |  |  | |  |  |  |  | | 1.958 | | 1.885 | |  |
|  |  | |  |  |  |  | | 1.992 | | 1.894 | |  | |  |  |  |  | |  |  |  |  | | 1.988 | | 1.944 | |  |
|  |  | |  |  |  |  | | 1.965 | | 1.789 | |  | |  |  |  |  | |  |  |  |  | | 1.982 | | 1.783 | |  |
| 2jpz-5 |  | | K^+^ | | |  | | 1.661 | | 1.922 | |  | |  | 2jpz-10 | |  | | K^+^ | | |  | | 1.949 | | 1.797 | |  |
|  |  | |  |  |  |  | | 1.875 | | 2.038 | |  | |  |  |  |  | |  |  |  |  | | 1.903 | | 2.089 | |  |
|  |  | |  |  |  |  | | 1.973 | | 3.238 | |  | |  |  |  |  | |  |  |  |  | | 1.695 | | 1.773 | |  |
|  |  | |  |  |  |  | | 1.795 | | 2.044 | |  | |  |  |  |  | |  |  |  |  | | 1.701 | | 2.018 | |  |
|  |  | |  |  |  |  | | 1.754 | | 1.845 | |  | |  |  |  |  | |  |  |  |  | | 1.912 | | 2.003 | |  |
|  |  | |  |  |  |  | | 1.696 | | 2.004 | |  | |  |  |  |  | |  |  |  |  | | 1.708 | | 1.979 | |  |
|  |  | |  |  |  |  | | 1.769 | | 1.855 | |  | |  |  |  |  | |  |  |  |  | | 1.962 | | 1.89 | |  |
|  |  | |  |  |  |  | | 1.92 | | 1.977 | |  | |  |  |  |  | |  |  |  |  | | 1.714 | | 1.986 | |  |
|  |  | |  |  |  |  | | 1.757 | | 2.011 | |  | |  |  |  |  | |  |  |  |  | | 1.833 | | 1.984 | |  |
|  |  | |  |  |  |  | | 1.981 | | 1.806 | |  | |  |  |  |  | |  |  |  |  | | 1.855 | | 2.013 | |  |
|  |  | |  |  |  |  | | 2.001 | | 1.791 | |  | |  |  |  |  | |  |  |  |  | | 2.005 | | 1.85 | |  |
|  |  | |  |  |  |  | | 1.801 | | 1.848 | |  | |  |  |  |  | |  |  |  |  | | 1.97 | | 1.794 | |  |

Table S10. M^+^∙∙∙O and O∙∙∙O length of optimized monovalent metal ions with water molecules.

| **Hydrated M^+^** | **Length (Å)** | | | | | | | | | | | | |
| --- | --- | --- | --- | --- | --- | --- | --- | --- | --- | --- | --- | --- | --- |
| **Li^+^∙∙∙4H_2_O** | **M^+^∙∙∙O** | 1.957 | 1.957 | 1.954 | 1.955 |  |  |  |  |  |  |  |  |
|  | **O∙∙∙O** | 3.155 | 3.251 | 3.200 | 3.136 | 3.272 | 3.147 |  |  |  |  |  |  |
| **Na^+^∙∙∙5H_2_O** | **M^+^∙∙∙O** | 2.304 | 2.335 | 2.315 | 2.366 | 2.398 |  |  |  |  |  |  |  |
|  | **O∙∙∙O** | 3.398 | 3.276 | 3.439 | 4.296 | 3.902 | 3.817 | 3.480 | 3.365 | 2.791 |  |  |  |
| **K^+^∙∙∙6H_2_O** | **M^+^∙∙∙O** | 2.681 | 2.760 | 2.726 | 2.900 | 2.727 | 2.759 |  |  |  |  |  |  |
|  | **O∙∙∙O** | 3.565 | 4.203 | 4.091 | 4.115 | 4.114 | 4.340 | 2.836 | 4.148 | 4.041 | 4.298 | 3.384 | 2.835 |

Table S11. Energy components of G4∙∙∙M^+^∙∙∙G4. Δ*E_int_* (w/o hydration) is summation of ΣΔ*E_pair_*, ΣΔ*E_stack_*, and ΣΔ*E_ionic_*, and Δ*E_int_* (w/ hydration) is obtained by subtracting Δ*E_hydration_* from Δ*E_int_* (w/o hydration).

| **Quadruplex** | **ΣΔ*E_pair_*** | **ΣΔ*E_stack_*** | **ΣΔ*E_ionic_*** | **Δ*E_hydration_*** | **Δ*E_int_* (w/o hydration)** | **Δ*E_int_* (w/ hydration)** |
| --- | --- | --- | --- | --- | --- | --- |
| **G4∙∙∙Li^+^∙∙∙G4** | -76.91 | -30.02 | -38.48 | -47.79 | -145.42 | -97.62 |
| **G4∙∙∙Na^+^∙∙∙G4** | -76.91 | -30.02 | -45.27 | -43.54 | -152.20 | -108.66 |
| **G4∙∙∙K^+^∙∙∙G4** | -76.91 | -30.02 | -44.77 | -40.37 | -151.71 | -111.34 |
